# Lifestyles Shape the Cytochrome P450 Repertoire of the Bacterial Phylum *Proteobacteria*

**DOI:** 10.3390/ijms23105821

**Published:** 2022-05-22

**Authors:** Siphesihle Msweli, Andiswa Chonco, Lihle Msweli, Puleng Rosinah Syed, Rajshekhar Karpoormath, Wanping Chen, Dominik Gront, Bridget Valeria Zinhle Nkosi, David R. Nelson, Khajamohiddin Syed

**Affiliations:** 1 Department of Biochemistry and Microbiology, Faculty of Science and Agriculture, University of Zululand, KwaDlangezwa 3886, South Africa; siphesihlemsweli2001@gmail.com (S.M.); angelicaandyc@gmail.com (A.C.); lihle.msweli9991@gmail.com (L.M.); brilenhle@gmail.com (B.V.Z.N.); 2 Department of Pharmaceutical Chemistry, College of Health Sciences, University of KwaZulu-Natal, Durban 4000, South Africa; prosinah@gmail.com (P.R.S.); Karpoormath@ukzn.ac.za (R.K.); 3 Department of Molecular Microbiology and Genetics, University of Göttingen, 37077 Göttingen, Germany; chenwanping1@foxmail.com; 4 Biological and Chemical Research Center, Faculty of Chemistry, University of Warsaw, Pasteura 1, 02-093 Warsaw, Poland; dgront@gmail.com; 5 Department of Microbiology, Immunology and Biochemistry, University of Tennessee Health Science Center, Memphis, TN 38163, USA

**Keywords:** *Alphaproteobacteria*, *Betaproteobacteria*, *Gammaproteobacteria*, *Deltaproteobacteria*, *Epsilonproteobacteria*, genome-data mining, P450s, evolution, diversity, secondary metabolism, biosynthetic gene-clusters

## Abstract

For the last six decades, cytochrome P450 monooxygenases (CYPs/P450s), heme thiolate proteins, have been under the spotlight due to their regio- and stereo-selective oxidation activities, which has led to the exploration of their applications in almost all known areas of biology. The availability of many genome sequences allows us to understand the evolution of P450s in different organisms, especially in the Bacteria domain. The phenomenon that “P450s play a key role in organisms’ adaptation vis a vis lifestyle of organisms impacts P450 content in their genome” was proposed based on studies on a handful of individual bacterial groups. To have conclusive evidence, one must analyze P450s and their role in secondary metabolism in species with diverse lifestyles but that belong to the same category. We selected species of the phylum *Proteobacteria* classes, *Alpha*, *Beta*, *Gamma*, *Delta*, and *Epsilon*, to address this research gap due to their diverse lifestyle and ancient nature. The study identified that the lifestyle of alpha-, beta-, gamma-, delta-, and epsilon-proteobacterial species profoundly affected P450 profiles in their genomes. The study determined that irrespective of the species associated with different proteobacterial classes, pathogenic species or species adapted to a simple lifestyle lost or had few P450s in their genomes. On the contrary, species with saprophytic or complex lifestyles had many P450s and secondary metabolite biosynthetic gene clusters. The study findings prove that the phenomenon mentioned above is factual, and there is no link between the number and diversity of P450s and the age of the bacteria.

## 1. Introduction

Cytochrome P450 monooxygenases (CYPs/P450s), heme thiolate proteins, play a key role in organisms’ primary and secondary metabolism. These proteins are present across the species of different biological kingdoms, including in non-living entities such as viruses [1,2]. Due to their regio- and stereo-specific oxidation properties, their biotechnological potentials have been explored in various fields of biology [3]. One of the best examples is their involvement in producing secondary metabolites, organic compounds with potential biotechnological applications [4,5].

Since the identification of P450s dating back to the 1950s and the 1960s in rat liver [6,7,8,9], due to the ongoing genome sequencing rush, many P450s have been identified in species belonging to different biological kingdoms [1]. Due to the advantage of genome sequencing, understanding the evolution of P450s across different species is now gaining momentum. In this direction, recent studies focusing on bacterial P450s demonstrated that the impact of lifestyle profoundly affects the P450 profiles vis a vis P450s playing a role in organisms’ adaptation to diverse ecological niches [10,11,12,13,14,15]. The analysis of P450s in the genera *Streptomyces* and *Mycobacterium* revealed that *Streptomyces* species P450s are highly diverse, producing various secondary metabolites, thus securing the niche area [10,11,16]. P450s in mycobacterial species were found to play a role in utilizing or synthesizing lipids or helping in pathogenesis by producing an array of secondary metabolites [10,11,16,17,18]. The pathogenic or commensal lifestyle influenced P450 content to such an extent that species belonging to the class *Gammaproteobacteria* and the phylum *Firmicutes* have a low number or no P450s in their genomes [12,13]. A study on alphaproteobacterial species P450s revealed that P450s passed from these ancient bacterial species to other bacterial species [15]. The study also revealed that more P450s were involved in secondary metabolism during speciation, indicating that they certainly played a role in adapting species to diverse ecological niches [15]. The study also identified no link between the number and diversity of P450s and the age of bacteria, indicating that the P450 profiles of an organism are strongly related to its lifestyle [13].

Most of the studies indicated above focus on the individual category of organisms. The analysis of P450s and their role in secondary metabolism in species adapted to diverse habitats and belonging to the same phylum will provide conclusive evidence on the impact of lifestyle vis a vis P450s’ role in an organism’s adaptation to ecological niches. To address this research gap, in this study, we performed a comprehensive comparative analysis of P450s and their role in secondary metabolism in the bacterial phylum *Proteobacteria*. 

*Proteobacteria* are considered the largest phylum of Bacteria [19,20]. It consists of phenotypically and metabolically diverse species adapted to different ecological niches [19,20]. *Proteobacteria* are medically, ecologically, and scientifically important [19,20]. Proteobacteria are divided into seven classes: *Alphaproteobacteria*, *Betaproteobacteria*, *Gammaproteobacteria*, *Deltaproteobacteria*, *Epsilonproteobacteria*, *Zetaproteobacteria*, and *Acidithiobacillia* [21]. Brief information on well-known proteobacterial classes is presented below to highlight their lifestyle diversity.

All alphaproteobacterial species are oligotrophic, which means they can survive in an environment with low nutrients, such as deep oceanic sediments, deep undersurface soil, or glacial ice [22]. Alphaproteobacterial species are crucial in carbon, nitrogen, and sulfur cycles [23]. It is well known that alphaproteobacterial species are the original source of mitochondria [24]. Some notable alphaproteobacterial species include plant pathogens belonging to the genus *Agrobacterium* and human pathogens belonging to the genera *Rickettsiaceae*, *Brucellaceae,* and *Bartonellaceae* [25]. Alphaproteobacterial species also produce secondary metabolites with potential human benefits [15]. 

*Betaproteobacteria* has the most heterogeneous species concerning metabolic, morphological, and ecological viewpoints [26]. Some of the most well-known species include human-, animal- and plant-pathogens, especially from the genera, *Bordetella*, *Burkholderia*, *Neisseria*, *Taylorella*, *Acidovorax*, *Ralstonia*, and *Xylophilus* [26]. Apart from pathogens, this class also has species able to fix atmospheric nitrogen, saprophytes, and biotechnologically relevant species that can produce human valuable secondary metabolites [26]. One of the best examples is the production of poly-*β*-hydroxybutyrate (bioplastics) and single-cell protein from *Ralstonia eutropha* [26]. 

*Gammaproteobacteria* is the largest and most diverse class of *Proteobacteria* [20,26]. Most of the bacterial species in this class are commensals with the ability to cause diseases, and some of them are strictly pathogenic [20,26]. Some of the most well-known pathogens include species belonging to the genera *Citrobacter*, *Enterobacter*, *Escherichia*, *Klebsiella*, *Proteus*, *Salmonella*, *Serratia*, *Shigella,* and *Yersinia* [12]. Information on well-known pathogens of this class is presented elsewhere [12].

*Deltaproteobacteria* consists of the most peculiar species in *Proteobacteria* [20,26]. Some species live as predators of other bacteria. For example, species belonging to the genus *Bdellovibrio* have known parasites of other Gram-negative bacteria. Some species belonging to the genera *Desulfovibrio*, *Desulfobacter,* and *Desulfuromonas* are well-known sulfate-reducing bacteria using sulfate as the final electron acceptor in the electron transport chain [20,26]. Species belonging to myxobacteria display complex developmental life cycles, such as forming multicellular structures known as fruiting bodies [20,26]. These species live in the soil and feed on other bacteria or decaying material [20,26]. Apart from predatory behavior, myxobacterial species produce various secondary metabolites, and their biotechnological potentials, including cancer treatment, have been explored [27].

*Epsilonproteobacteria* consist of well-known enteropathogen species of humans and animals [20,26]. Species belonging to the genera *Campylobacter* and *Helicobacter* cause food poisoning, chronic gastritis, stomach ulcers, and duodenum ulcers [20,26]. These species mainly obtain energy from amino acids or tricarboxylic acid cycle intermediates [20,26]. Other species include sulfate or sulfur-reducing bacteria belonging to the genus *Sulfurospirillum* and plant symbiotic nitrogen-fixing bacteria *Arcobacter nitrofigilis* [20,26].

As indicated above, considering the ancient nature, highly diverse lifestyle, and availability of many genomes, proteobacterial species are ideal for studying the evolution and diversity of P450s and their role in secondary metabolism concerning the impact of lifestyle, if any, on the P450 repertoire in organisms.

## 2. Results and Discussion

### 2.1. Deltaproteobacterial Species Have the Highest P450 Diversity

Genome-wide data mining for P450s in 2696 proteobacterial species belonging to four different classes (*Alpha*-, *Beta*-, *Gamma*-, *Delta*-, and *Epsilon-proteobacteria*) revealed the presence of P450s only in 764 species (28%), indicating that most of the proteobacterial species do not have P450s in their genomes (Table 1 and Table S1). Among proteobacterial species, the highest number of species with P450s was found in *Betaproteobacteria* (57%), followed by *Alphaproteobacteria* (38%), *Epsilonproteobacteria* (25%), *Deltaproteobacteria* (21%), and *Gammaproteobacteria* (13%). This indicates that most gammaproteobacterial species have no P450s in their genomes (Table 1 and Table S1). In a bacterial group, a few species having P450s is not unique, as observed in *Firmicutes* [13]. Genera level analysis revealed that some of the species belonging to a particular genus in different classes have no P450s in their genomes (Table S1). Based on the number of species analyzed, we conclude that species belonging to the genera include *Neisseria*, *Taylorella*, and *Kinetoplastibacterium* of *Betaproteobacteria*; *Geobacter*, *Desulfovibrio*, *Pseudodesulfovibrio* of *Deltaproteobacteria,* and *Helicobacter* (except for *Helicobacter winghamensis*) of *Epsilonproteobacteria* does not have P450s (Table S1). A recent study identified that species belonging to the genera *Shewanella*, *Aeromonas*, *Haemophilus* of *Gammaproteobacteria* and *Rickettsia*, *Bartonella*, *Ehrlichia, Wolbachia,* and *Anaplasma* of *Alphaproteobacteria* also do not have P450s in their genomes [12,13]. A comparative analysis of P450s revealed the presence of the highest number of P450s in alphaproteobacterial species (874 P450s), and the lowest number of P450s is in epsilonproteobacterial species (53 P450s) (Table 1). The P450 count in other proteobacterial classes is as follows: beta-, 603 P450s; delta-, 333 P450s; and gamma-proteobacterial species, 277 P450s (Table 1). The average number of P450s was found to be highest in delta- (14 P450s), followed by alpha-species (4 P450s), beta- and gamma- (2 P450s), and epsilon-proteobacterial species (single P450) (Table 1). Among 764 species belonging to the four different proteobacterial classes, the highest number of P450s was found in *Archangium gephyra* (56 P450s), followed by *Archangium violaceum* (43 P450s), *Cystobacter fuscus* (42 P450s), *Melittangium boletus* (28 P450s), *Chondromyces crocatus* (26 P450s) and *Minicystis rosea* (24 P450s) of *Deltaproteobacteria* (Table S1). The P450 count in other *Proteobacteria* classes is as follows: a single to nine P450s in betaproteobacterial species; a single P450 to six P450s in gammaproteobacterial species [12]; a single to seventeen P450s in alphaproteobacterial species [15]; and only one P450 found in epsilonproteobacterial species (Table S1). Detailed information on genera, species, and P450 information for *Beta*-, *Delta*- and *Epsilon-proteobacteria* is presented in Table S1.

### 2.2. Proteobacterial Species Have Highly Diverse P450s

**Figure 1:**
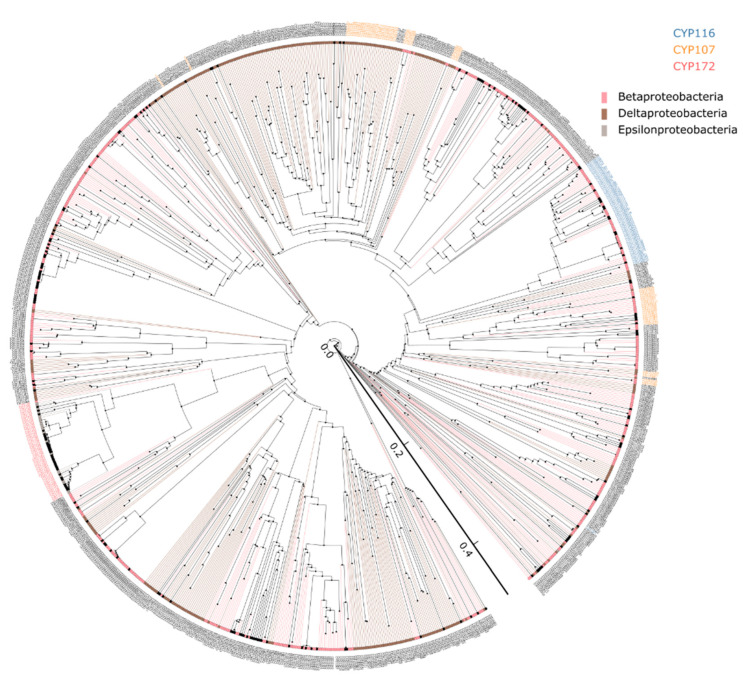
Phylogenetic analysis of P450s from gamma-, delta-, and epsilon-proteobacterial species. P450 families that are expanded in these species and proteobacterial classes were highlighted in different colors and indicated in the figure. P450 protein sequences used for constructing the phylogenetic tree are presented in Table S3. A high-resolution phylogenetic tree is provided in Figure S1.

Annotation and assigning the family and subfamily of the P450s were done as per International P450 Nomenclature Committee rules [28,29,30] that includes phylogenetic analysis (Figure 1). the 2139 P450s from five different proteobacterial classes can be grouped into 292 P450 families and 564 P450 subfamilies (Table S2). As shown in Figure 1, P450s belonging to the same family are grouped, indicating the correct annotation of P450s. However, a few CYP107 P450s are scattered across the evolutionary tree (Figure 1). This phenomenon was also observed previously for *Streptomyces* species P450s [10,11]. It has been hypothesized that the phylogenetic-based annotation of P450s could detect similarity cues beyond a simple percentage identity cutoff [10,11]. Beta-, delta-, and epsilon-proteobacterial species P450s identified in this study, along with their protein sequences and species, are presented in Table S3. 

**Figure 2:**
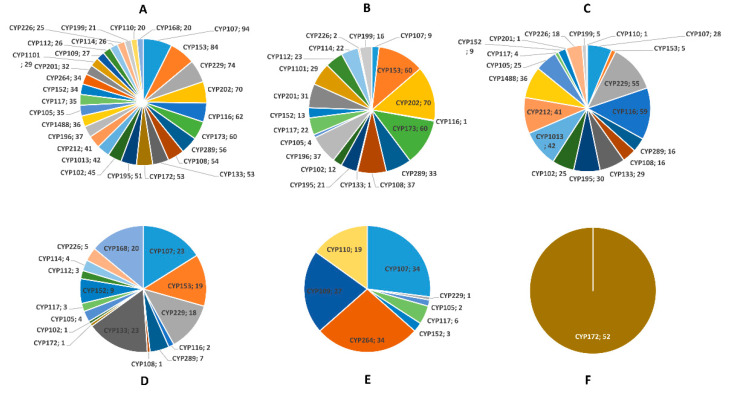
Comparative analysis of P450 families in proteobacterial species. (**A**) Complete set of P450 families in five different classes of proteobacterial species. P450 family analysis in alphaproteobacterial species (**B**), betaproteobacterial species (**C**), gammaproteobacterial species (**D**), deltaproteobacterial species (**E**), and epsilonproteobacterial species (**F**). The P450 families with ≥20 members are presented in the figure. The P450 family name and number of P450s are shown in the figure. Detailed information on P450 families and subfamilies is presented in Table S2.

Among proteobacterial species, alpha- had the highest number of P450 families (143 families), followed by gamma- (81 families), beta- (79 families), and delta- (74 families), indicating high P450 family diversity in alpha-proteobacterial species (Table 1). In contrast, epsilonproteobacterial species have only 2 P450 families (Table 1 and Table S2). Among P450 families, CYP107 has the highest number of members (94 P450s), followed by CYP153 (84 P450s), CYP229 (74 P450s), CYP202 (70 P450s), and CYP116 (62 P450s) (Figure 2A and Table S2). Proteobacterial species belonging to different classes expanded a particular P450 family in their genomes (Figure 2B–F and Table S2), suggesting the important role of this dominant P450 family in their physiology. The dominant P450 families in different proteobacterial species are CYP202 in alpha-*,* CYP116 in beta-, CYP113 and CYP107 in gamma-, CYP264 and CYP107 in delta-, and CYP172 in epsilon-proteobacterial species (Figure 2B–F and Table S2). The analysis of the P450 subfamilies revealed the presence of 564 subfamilies where dominant P450 families have the highest number of subfamilies, indicating further diversity at the subfamily level (Table 1 and Table S2). Interestingly, subfamily level preference was observed in proteobacterial species where a particular subfamily has more members (Table 1 and Table S2), indicating the importance of these P450s in their physiology. The analysis of subfamilies among proteobacterial species revealed the presence of the highest P450 subfamilies in alpha- (214 subfamilies), followed by delta- (171 subfamilies), beta- (119 subfamilies), gamma- (102 subfamilies), and epsilon-proteobacterial species (2 subfamilies). This indicates that the alphaproteobacterial species have the highest number of P450 families and subfamilies in their genomes. 

**Figure 3:**
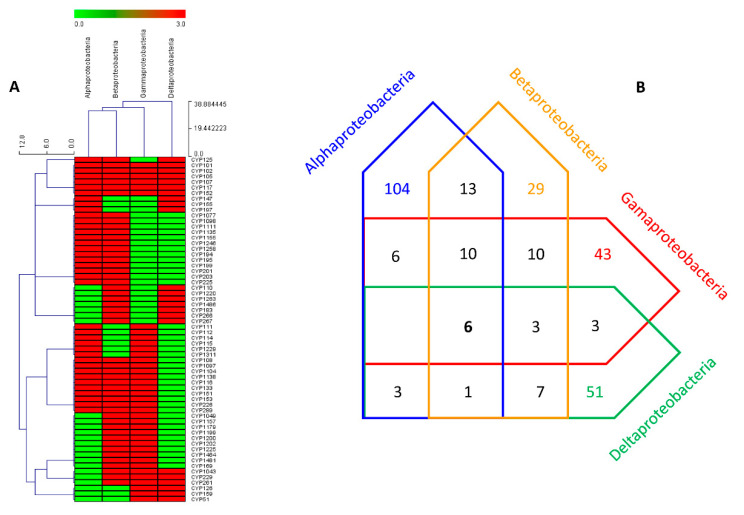
P450 family conservation analysis in proteobacterial species belonging to four different classes. (**A**) The heat map represents the presence (red) or absence (green) of the P450 family in proteobacterial species. P450 families form the vertical axis, and four different proteobacterial classes form the horizontal axis. (**B**) A Venn diagram represents the number of P450 families that are unique and commonly present among proteobacterial species belonging to four different classes. The numbers indicate the number of P450 families. The number of P450 families conserved among four proteobacterial classes is indicated in bold. A detailed analysis of the families, subfamilies, and member count is presented in Table S2.

P450 family conservation across proteobacterial species belonging to four different classes (*Alpha*, *Beta*, *Gamma*, *Delta*, and *Epsilon*) was carried out, except for the epsilonproteobacterial species, as they have only two P450 families. A comparative analysis of P450 families among proteobacterial species revealed the conservation of six P450 families, CYP101, CYP102, CYP105, CYP107, CYP117, and CYP152, across species belonging to four different classes (Figure 3), indicating their common ancestral origin. A moderate number of P450 families were shared among alpha-, beta-, gamma-, and delta-proteobacterial species, except for no shared P450 found between alpha- and delta-proteobacterial species (Figure 3). One of the interesting features was that many P450 families were unique in different proteobacterial species. The number of unique P450 families in proteobacterial species is as follows: 104 families in alpha-, 51 in delta-, 43 in gamma-, and 29 in beta-proteobacterial species (Figure 3). This suggests the high diversity of P450 families in these species, possibly indicating their lifestyle influence on the P450 repertoire, the same as observed in other species [10,11,12,13,14,15]. One of the two P450 families in epsilonproteobacterial species, such as CYP172, was also present in gammaproteobacterial species (Figure 3 and Table S2). Two notable mentions are CYP51, the sterol 14α-demethylase, present in gamma- and delta-proteobacterial species, whereas the CYP125, the cholesterol side-chain oxidase, is present in alpha-, beta- delta-proteobacterial species and not found in gammaproteobacterial species (Figure 3). The presence of CYP51 in proteobacterial species is reported earlier in support of the bacterial origin of CYP51 that ultimately passed to eukaryotes, as described elsewhere [31]. A point to be noted is that a recent study showed that alphaproteobacterial species are capable of cholesterol oxidation [15], and the presence of CYP125 in other proteobacterial species suggests this is a common phenomenon in these species. 

An analysis of the P450 diversity percentage revealed that deltaproteobacterial species have the highest P450 diversity percentage (0.97%), and betaproteobacterial species have the lowest P450 diversity percentage (0.05%) (Table 1). This indicates the presence of highly diverse P450s in deltaproteobacterial species. A point to be noted is that despite having the highest number of P450 families and subfamilies, the P450 diversity percentage in alphaproteobacterial species was found to be lowest compared to deltaproteobacterial species (Table 2). This is partly due to that fact that many alphaproteobacterial species were analyzed in this study compared to deltaproteobacterial species. In the future, the availability of more deltaproteobacterial species genomes will undoubtedly provide more insights into this aspect. A detailed analysis of the families, subfamilies, and member count is presented in Table S2.

### 2.3. More P450s Are Involved in Secondary Metabolism in Delta-, Compared to Alpha-, Gamma-, and Beta-Proteobacterial Species

The analysis of P450s that are part of secondary metabolite biosynthetic gene clusters (smBGCs) revealed that only 22% of proteobacterial species P450s from four different classes (*Alpha*-, *Beta*-, *Gamma*-, and *Delta*-) are involved in the production of secondary metabolites (Table 1). P450s from epsilonproteobacterial species were not part of smBGCs (Table S4), indicating their role was confined only to primary metabolism. Most of the epsilonproteobacterial species did not have the smBGCs in their genomes, and the ones that had only had a single smBGCs in their genome (Table S4). In contrast, species belonging to classes *Alpha*, *Gamma*, *Beta*, and *Delta* have many smBGCs in their genomes (Table S4). The percentage of P450s part of smBGCs in proteobacterial species was found to be highest compared to other bacterial species such as *Cyanobacteria* (8%) [14], mycobacterial species (11%) [10], and *Firmicutes* species (18%) [13] and lowest with only one percent compared to *Streptomyces* species (23%) [10,11]. The analysis of smBGCs P450s revealed that betaproteobacterial species have the highest number of P450s part of smBGCs (107 P450s), followed by delta- (69 P450s), gamma- (49 P450s), and alpha-proteobacterial species (21 P450s) (Table 2). However, when the percentage of P450s part of BGCs was calculated, deltaproteobacterial species had the highest percentage of P450s as part of smBGCs (21%). In contrast, it was 18% for beta- and gamma-proteobacterial species, and it was only 2% for alphaproteobacterial species (Table 1), indicating deltaproteobacterial species P450s play a prominent role in secondary metabolism. Furthermore, deltaproteobacterial species had the highest number of P450 families as part of smBGCs (37 families) compared to gamma- (22 families), beta- (18 families), and alpha-proteobacterial species, which had the lowest number of P450 families (16 families) as part of smBGCs (Table 1). An interesting pattern, such as no correlation was observed between the dominant P450 family in species and the dominant P450 family as part of smBGCs in the same group of species (Table 1 and Table 2). This means a P450 family can be dominantly present in species, but it may not play a dominant role in secondary metabolism. CYP202, CYP116, CYP113, and CYP264 are prevalent in the alpha-, beta-, gamma- and delta-proteobacterial species (Table 1), but they are not dominant concerning being part of smBGCs except for CYP107, which is prevalent in smBGCs in gamma-, and delta-proteobacterial species (Table 2).

### 2.4. P450 Repertoire of Proteobacterial Species Shaped by Their Lifestyle

To understand the effect of lifestyle on P450 profiles vis a vis P450s’ role in organism adaptation to particular ecological niches, one should look at an organism’s lifestyle and its evolutionary position in the tree of life. Based on the available literature, the evolutionary order from old to young is *Proteobacteria*, *Firmicutes*, *Actinobacteria*, *Planctomycetacia*, *Cyanobacteria*, *Chloroflexi*, *Bacteroidetes* [32,33]. Within *Proteobacteria*, from old to young, are *Epsilon*-, *Delta*-, *Alpha*-, *Gamma*, *Beta-proteobacteria* [32,33]. This means that proteobacterial species are ancient bacterial species compared to the other bacteria mentioned here. An analysis of P450s’ profiles in these species should provide insights on P450s’ evolutionary pattern and reflect the phenomenon discussed above, considering these species display extreme diversity concerning their lifestyle and ecological niches. 

Among epsilonproteobacterial genera, *Helicobacter* and *Campylobacter* have the highest number of known species (Table S1). These species are enteropathogens or commensals and extract energy from amino acids and tricarboxylic acid cycle intermediates [20,26]. Due to this lifestyle, such as surviving on more straightforward carbon sources, these species have no P450s, as observed in other species [34]. Furthermore, these species have the lowest number of P450s and only two P450 families (Table 1). They also have the lowest number of smBGCs, indicating they hardly produce secondary metabolites, and none of the P450s were found to be part of smBGCs (Table S4). 

Deltaproteobacterial species have complex lifestyles, such as living in soil with a predatory or saprophytic behavior (living on decaying materials) [20,26]. They form multicellular fruiting bodies resembling the eukaryotic lifestyle [20,26]. Due to this, they have the highest average number of P450s in their genome compared to other proteobacterial species (Table 1). Furthermore, the P450s part of smBGCs is the highest among proteobacterial species, suggesting these species produce a diverse array of secondary metabolites that help them survive in their ecological niches. Some of these metabolites target cellular structures and help them prey on other bacteria [20,26,27]. The number of P450s in deltaproteobacterial species and the percentage of P450s part of smBGCs is comparable with species belonging to the genera *Streptomyces* and *Mycobacterium* [10,11]. This strongly indicates that irrespective of evolutionary order of origin, the lifestyle certainly influences the P450s profiles *vis a vis* P450s helping organisms adapt to diverse ecological niches.

Alphaproteobacterial species are adapted to diverse ecological niches and can survive in a low-nutrient environment. In this class, human pathogenic species belonging to the genera *Rickettsiaceae* and *Bartonellaceae* have no P450s, and species belonging to the genus *Brucellaceae* have a single P450s, albeit some of the species have no P450s [15]. Furthermore, species belonging to the genus *Acetobacter* have no P450s [15]. These species are well known for their fermentation ability and adaptation to the utilization of simple sugars. Thus, they have no P450s. Loss of P450s/having few P450s due to organisms’ adaptation to simpler organic nutrients previously been reported [34]. Nonetheless, other species with diverse lifestyles do have P450s in their genome in this class (Table 1 and Table S1). However, alphaproteobacterial species have very few P450s that are part of smBGCs (Table 1), indicating most of the alphaproteobacterial P450s play a role in primary metabolism.

The most striking example of loss of P450s or having few P450s in pathogenic species was reported in *Gammaproteobacteria* [12]. The study revealed that most pathogenic species have no P450s, mainly *Citrobacter*, *Enterobacter*, *Escherichia*, *Klebsiella*, *Proteus*, *Salmonella*, *Serratia*, *Shigella,* and *Yersinia* [12]. On the contrary, species of environmental importance had P450s, and 18% of P450s were part of smBGCs, indicating they are involved in producing secondary metabolites [12].

*Betaproteobacteria* species are one of the most heterogeneous species in *Proteobacteria*. Clear evidence of a pathogenic lifestyle leading to having no or fewer P450s can be found in the species belonging to the genera *Bordetella*, *Burkholderia*, *Neisseria*, *Taylorella*, *Acidovorax*, *Ralstonia*, and *Xylophilus* (Table S1). On the other hand, biotechnologically valuable species that produce secondary metabolites or degrade various xenobiotics have more P450s in their genome (Table S1). Due to this type of lifestyle, *Betaproteobacteria* has the highest number of species with P450s and the highest number of P450s part of smBGCs in *Proteobacteria* (Table 1).

Considering the above facts, based on the literature published on bacterial P450s and following the evolutionary order, we conclude that the lifestyle of organisms profoundly impacts P450 repertoire *vis a vis* P450s’ key role in organisms’ adaptation to ecological niches.

## 3. Materials and Methods

### 3.1. Species and Their Genome Database Information

Proteobacterial species belonging to the *Beta*- (513 species), *Delta*- (107 species), and *Epsilon* (216 species) classes that are available for public use in the Kyoto Encyclopedia of Genes and Genomes (KEGG) [35] database were used. Information on genera, species names, and species codes are presented in Table S1.

### 3.2. Genome Data Mining and Annotation of P450s

P450 data mining and annotation were carried out following the standard procedure described previously by our laboratory [12,13,15]. Briefly, proteomes of each bacterial species were downloaded from the KEGG and subjected to the NCBI Batch Web CD-Search Tool [36]. The result was analyzed, and proteins that belong to the P450 superfamily were selected and searched for the presence of characteristic P450 motifs, EXXR, and CXG [37,38]. Proteins that were short in amino acid length and lacked both motifs were regarded as P450 fragments, and these P450 fragments were not considered for further analysis. Proteins having both motifs were selected for the assignment of the family and subfamilies. Following the International P450 Nomenclature Committee rule [28,29,30], proteins with >40% identity and >55% identity were grouped under the same family and subfamily, respectively. P450s with less than 40% identity were assigned to a new P450 family. Beta-, delta-, and epsilon-proteobacterial species P450s identified in this study, along with their protein sequences and species, are presented in Table S3.

### 3.3. Phylogenetic Analysis of P450s

Phylogenetic analysis of P450s was carried out following the procedure described recently by our laboratory [12,13]. The phylogenetic tree of P450s was constructed using protein sequences (Table S3). Firstly, the MAFFT v6.864 [39] was used to align the Trex web server’s protein sequences [40]. The alignments were then used to interpret the best tree by the Trex web server [40]. Lastly, a web-based tool, VisuaLife, was used to create, visualize, and color the tree [41].

### 3.4. Generation of P450 Profile Heat-Maps

The generation of the heat map profile was carried out using the method previously reported by our laboratory [12,13]. The data were represented as (−3) for P450 family/subtype absence (green) and (3) for P450 family/subtype presence (red). A tab-delimited file was imported into Mev (Multi-experiment viewer) [42]. Hierarchical clustering using a Euclidean distance metric was used to cluster the data. P450 families formed the vertical axis, and proteobacterial classes formed the horizontal axis. The P450 families that are shared between four different proteobacterial classes such as *Alpha*, *Beta*, *Gama*, and *Delta,* are presented in the figure.

### 3.5. Identification of P450s Part of smBGCs

P450s part of the smBGCs was identified using the procedure described by our laboratory [13,15]. Briefly, genome ID of beta, delta, and epsilon-proteobacterial species was submitted to anti-SMASH (antibiotics & Secondary Metabolite Analysis Shell) [43,44] to identify smBGCs. Anti-SMASH results were downloaded in gene cluster sequences and Excel spreadsheets representing species-wise cluster information. P450s that formed part of a specific gene cluster were identified by manual data mining of gene cluster sequences. Standard gene cluster abbreviation terminology available at the anti-SMASH database [43] was maintained in this study.

### 3.6. P450 Key Features Analysis

All calculations were carried out following the procedure reported previously by our laboratory [12]. The average number of P450s was calculated using the formula: Average number of P450s = Number of P450s/Number of species. The P450 diversity percentage was calculated using the formula: P450 diversity percentage = 100 × Total number of P450 families/Total number of P450s × Number of species with P450s. The percentage of P450s that formed part of B.G.C.s was calculated using the formula: Percentage of P450s part of B.G.C.s = 100 × Number of P450s part of BGCs/Total number of P450s present in species.

### 3.7. Comparative Analysis of P450s and smBGCs Data

P450s and smBGCs data for alpha- and gamma-proteobacterial species were retrieved from published articles [12,15] and used for comparative analysis.

## 4. Conclusions

Organisms change their gene pool as per their necessity to adapt to diverse ecological niches. Specific genes, amplification, expansion, gain, or loss, entirely depend on the organism’s lifestyle. Here, we provide conclusive evidence of such a phenomenon concerning cytochrome P450 monooxygenases (CYPs/P450s). P450s are ubiquitously present in organisms due to their important role in primary and secondary metabolism. P450s analysis in the ancient and diverse bacterial phylum *Proteobacteria* revealed that pathogenic species or species adapted to living on simple carbon sources lost or have fewer P450s than saprophytes or species with complex lifestyles. In addition to this, most proteobacterial species, especially pathogens, are facultative or obligate anaerobes. This kind of lifestyle may also be the reason for them not having P450s in their genomes. Furthermore, P450s were found to play a role in secondary metabolism in saprophytes or species with complex lifestyles, and thus, these species expanded P450s in their genomes.

## Figures and Tables

**Table 1:** Comparative analysis of key features of P450s and their association with secondary metabolism in different proteobacterial species. Abbreviation: No or no., number of; BGCs: biosynthetic gene clusters.

Category	*Alphaproteobacteria*	*Betaproteobacteria*	*Gammaproteobacteria*	*Deltaproteobacteria*	*Epsilonproteobacteria*
Species analyzed	599	513	1261	107	216
Species without P450s	370	223	1091	84	163
Species with P450s	229	290	169	23	53
Percentage of species with P450s	38	57	13	21	25
No. of P450s	873	603	277	333	53
No. of families	143	79	81	74	2
No. of subfamilies	214	119	102	171	2
Dominant P450 family	CYP202	CYP116	CYP133 & CYP107	CYP107	CYP172
Average no. of P450s	4	2	2	14	1
P450 diversity percentage	0,07	0,05	0,17	0,97	0,07
No. of P450s part of BGCs	21	107	49	69	0
No. of P450 families part of BGCs	16	18	22	37	0
Percentage of P450s part of BGCs	2	18	18	21	0
Reference	[15]	This study	[12]	This study	This study

**Table 2:** Comparative analysis of P450s involved in secondary metabolism in proteobacterial species. P450 family name and number of members that are part of the secondary metabolite biosynthetic gene cluster are shown in the table. Detailed information on secondary metabolite clusters, species, and P450s are presented in Table S4.

*Alphaproteobacteria*		*Betaproteobacteria*		*Gammaproteobacteria*		*Deltaproteobacteria*	
P450 Family	Count	P450 Family	Count	P450 Family	Count	P450 Family	Count
CYP206	5	CYP1013	39	CYP107	18	CYP107	6
CYP1101	2	CYP1488	37	CYP1465	4	CYP1011	4
CYP2334	1	CYP107	4	CYP105	3	CYP262	4
CYP199	1	CYP116	4	CYP126	3	CYP264	4
CYP173	1	CYP117	4	CYP134	2	CYP110	3
CYP153	1	CYP133	4	CYP153	2	CYP120	3
CYP152	1	CYP1486	3	CYP159	2	CYP253	3
CYP1302	1	CYP1464	2	CYP116	1	CYP263	3
CYP127	1	CYP1104	1	CYP1200	1	CYP1069	2
CYP1246	1	CYP1200	1	CYP1247	1	CYP109	2
CYP1138	1	CYP1246	1	CYP1278	1	CYP126	2
CYP1104	1	CYP1318	1	CYP1414	1	CYP1329	2
CYP1138	1	CYP1481	1	CYP1475	1	CYP1486	2
CYP108	1	CYP1686	1	CYP163	1	CYP183	2
CYP107	1	CYP183	1	CYP289	1	CYP251	2
CYP1326	1	CYP2308	1	CYP1201	1	CYP51	2
		CYP261	1	CYP1468	1	CYP105	1
		CYP267	1	CYP1469	1	CYP1224	1
				CYP1472	1	CYP1298	1
				CYP1477	1	CYP1347	1
				CYP1779	1	CYP1448	1
				CYP2242	1	CYP147	1
						CYP1489	1
						CYP1490	1
						CYP1491	1
						CYP1494	1
						CYP1497	1
						CYP1498	1
						CYP1499	1
						CYP1503	1
						CYP1504	1
						CYP152	1
						CYP167	1
						CYP209	1
						CYP229	1
						CYP242	1

## Data Availability

Not applicable.

## Supplementary Materials

The following are available online at https://www.mdpi.com/article/10.3390/ijms23105821/s1.

## References

[1] Nelson D.R. (2018). Cytochrome P450 diversity in the tree of life. Biochim. Biophys. Acta Proteins Proteom..

[2] Lamb D.C., Follmer A.H., Goldstone J.V., Nelson D.R., Warrilow A.G., Price C.L., True M.Y., Kelly S.L., Poulos T.L., Stegeman J.J. (2019). On the occurrence of cytochrome P450 in viruses. Proc. Natl. Acad. Sci. USA.

[3] Yamazaki H. (2014). Fifty Years of Cytochrome P450 Research.

[4] Podust L.M., Sherman D.H. (2012). Diversity of P450 enzymes in the biosynthesis of natural products. Nat. Prod. Rep..

[5] Greule A., Stok J.E., De Voss J.J., Cryle M.J. (2018). Unrivalled diversity: The many roles and reactions of bacterial cytochromes P450 in secondary metabolism. Nat. Prod. Rep..

[6] Klingenberg M. (1958). Pigments of rat liver microsomes. Arch. Biochem. Biophys..

[7] Garfinkel D. (1958). Studies on pig liver microsomes. I. Enzymic and pigment composition of different microsomal fractions. Arch. Biochem. Biophys..

[8] Omura T., Sato R. (1962). A new cytochrome in liver microsomes. J. Biol. Chem..

[9] Omura T., Sato R. (1964). The carbon monoxide-binding pigment of liver microsomes. I. Evidence for its hemoprotein nature. J. Biol. Chem..

[10] Senate L.M., Tjatji M.P., Pillay K., Chen W., Zondo N.M., Syed P.R., Mnguni F.C., Chiliza Z.E., Bamal H.D., Karpoormath R. (2019). Similarities, variations, and evolution of cytochrome P450s in *Streptomyces* versus *Mycobacterium*. Sci. Rep..

[11] Mnguni F.C., Padayachee T., Chen W., Gront D., Yu J.-H., Nelson D.R., Syed K. (2020). More P450s are involved in secondary metabolite biosynthesis in *Streptomyces* compared to *Bacillus*, *Cyanobacteria* and *Mycobacterium*. Int. J. Mol. Sci..

[12] Msomi N.N., Padayachee T., Nzuza N., Syed P.R., Kryś J.D., Chen W., Gront D., Nelson D.R., Syed K. (2021). In silico analysis of P450s and their role in secondary metabolism in the bacterial class Gammaproteobacteria. Molecules.

[13] Padayachee T., Nzuza N., Chen W., Nelson D.R., Syed K. (2020). Impact of lifestyle on cytochrome P450 monooxygenase repertoire is clearly evident in the bacterial phylum Firmicutes. Sci. Rep..

[14] Khumalo M.J., Nzuza N., Padayachee T., Chen W., Yu J.-H., Nelson D., Syed K. (2020). Comprehensive analyses of cytochrome P450 monoxygenases and secondary metabolite biosynthetic gene clusters in *Cyanobacteria*. Int. J. Mol. Sci..

[15] Nzuza N., Padayachee T., Syed P.R., Kryś J.D., Chen W., Gront D., Nelson D.R., Syed K. (2021). Ancient Bacterial Class Alphaproteobacteria Cytochrome P450 Monooxygenases Can Be Found in Other Bacterial Species. Int. J. Mol. Sci..

[16] Parvez M., Qhanya L.B., Mthakathi N.T., Kgosiemang I.K., Bamal H.D., Pagadala N.S., Xie T., Yang H., Chen H., Theron C.W. (2016). Molecular evolutionary dynamics of cytochrome P450 monooxygenases across kingdoms: Special focus on mycobacterial P450s. Sci. Rep..

[17] Syed P.R., Chen W., Nelson D.R., Kappo A.P., Yu J.H., Karpoormath R., Syed K. (2019). Cytochrome P450 Monooxygenase CYP139 Family Involved in the Synthesis of Secondary Metabolites in 824 Mycobacterial Species. Int. J. Mol. Sci..

[18] van Wyk R., van Wyk M., Mashele S.S., Nelson D.R., Syed K. (2019). Comprehensive comparative analysis of cholesterol catabolic genes/proteins in mycobacterial species. Int. J. Mol. Sci..

[19] Berman J.J. (2019). Taxonomic Guide to Infectious Diseases: Understanding the Biologic Classes of Pathogenic Organisms.

[20] Kersters K., De Vos P., Gillis M., Swings J., Vandamme P., Stackebrandt E. (2006). Introduction to the Proteobacteria. The Prokaryotes: A Handbook on the Biology of Bacteria.

[21] Williams K.P., Kelly D.P. (2013). Proposal for a new class within the phylum Proteobacteria, Acidithiobacillia classis nov., with the type order Acidithiobacillales, and emended description of the class Gammaproteobacteria. Int. J. Syst. Evol. Microbiol..

[22] Phung N.T., Lee J., Kang K.H., Chang I.S., Gadd G.M., Kim B.H. (2004). Analysis of microbial diversity in oligotrophic microbial fuel cells using 16S rDNA sequences. FEMS Microbiol. Lett..

[23] Li T., Tang K., Zhang L., Zhao Z., Xie X., Chen C.T.A., Wang D., Jiao N., Zhang Y. (2018). Coupled carbon, sulfur, and nitrogen cycles mediated by microorganisms in the water column of a shallow-water hydrothermal ecosystem. Front. Microbiol..

[24] Wang Z., Wu M. (2015). An integrated phylogenomic approach toward pinpointing the origin of mitochondria. Sci. Rep..

[25] Mukhopadhya I., Hansen R., El-Omar E.M., Hold G.L. (2012). IBD-what role do Proteobacteria play?. Nat. Rev. Gastroenterol. Hepatol..

[26] Dworkin M., Falkow S., Rosenberg E., Schleifer K.-H., Stackebrandt E. (2006). The Prokaryotes: Volume 5: Proteobacteria: Alpha and Beta Subclasses.

[27] Bhat M.A., Mishra A.K., Bhat M.A., Banday M.I., Bashir O., Rather I.A., Rahman S., Shah A.A., Jan A.T. (2021). Myxobacteria as a Source of New Bioactive Compounds: A Perspective Study. Pharmaceutics.

[28] Nelson D.R., Kamataki T., Waxman D.J., Guengerich F.P., Estabrook R.W., Feyereisen R., Gonzalez F.J., Coon M.J., Gunsalus I.C., Gotoh O. (1993). The P450 superfamily: Update on new sequences, gene mapping, accession numbers, early trivial names of enzymes, and nomenclature. DNA Cell Biol..

[29] Nelson D.R. (2006). Cytochrome P450 nomenclature, 2004. Methods Mol. Biol..

[30] Nelson D.R. (1998). Cytochrome P450 nomenclature. Methods Mol. Biol..

[31] Lamb D.C., Hargrove T.Y., Zhao B., Wawrzak Z., Goldstone J.V., Nes W.D., Kelly S.L., Waterman M.R., Stegeman J.J., Lepesheva G.I. (2021). Concerning P450 Evolution: Structural Analyses Support Bacterial Origin of Sterol 14α-Demethylases. Mol. Biol. Evol..

[32] Marin J., Battistuzzi F.U., Brown A.C., Hedges S.B. (2016). The timetree of prokaryotes: New insights into their evolution and speciation. Mol. Biol. Evol..

[33] Battistuzzi F.U., Hedges S.B. (2009). A major clade of prokaryotes with ancient adaptations to life on land. Mol. Biol. Evol..

[34] Kgosiemang I.K.R., Syed K., Mashele S.S. (2014). Comparative genomics and evolutionary analysis of cytochrome P450 monooxygenases in fungal subphylum *Saccharomycotina*. J. Pure Appl. Microbiol..

[35] Kanehisa M., Sato Y., Furumichi M., Morishima K., Tanabe M. (2019). New approach for understanding genome variations in KEGG. Nucleic Acids Res..

[36] Lu S., Wang J., Chitsaz F., Derbyshire M.K., Geer R.C., Gonzales N.R., Gwadz M., Hurwitz D.I., Marchler G.H., Song J.S. (2020). CDD/SPARCLE: The conserved domain database in 2020. Nucleic Acids Res..

[37] Graham S.E., Peterson J.A. (1999). How similar are P450s and what can their differences teach us?. Arch. Biochem. Biophys..

[38] Syed K., Mashele S.S. (2014). Comparative analysis of P450 signature motifs EXXR and CXG in the large and diverse kingdom of fungi: Identification of evolutionarily conserved amino acid patterns characteristic of P450 family. PLoS ONE.

[39] Katoh K., Kuma K., Toh H., Miyata T. (2005). MAFFT version 5: Improvement in accuracy of multiple sequence alignment. Nucleic Acids Res..

[40] Boc A., Diallo A.B., Makarenkov V. (2012). T-REX: A web server for inferring, validating and visualizing phylogenetic trees and networks. Nucleic Acids Res..

[41] Kryś J.D., Gront D. (2021). VisuaLife: Library for interactive visualization in rich web applications. Bioinformatics.

[42] Howe E.A., Sinha R., Schlauch D., Quackenbush J. (2011). RNA-Seq analysis in MeV. Bioinformatics.

[43] Blin K., Pascal Andreu V., de Los Santos E.L.C., Del Carratore F., Lee S.Y., Medema M.H., Weber T. (2019). The antiSMASH database version 2: A comprehensive resource on secondary metabolite biosynthetic gene clusters. Nucleic Acids Res..

[44] Blin K., Shaw S., Steinke K., Villebro R., Ziemert N., Lee S.Y., Medema M.H., Weber T. (2019). antiSMASH 5.0: Updates to the secondary metabolite genome mining pipeline. Nucleic Acids Res..

