# Supplementary material for: Lifestyles Shape the Cytochrome P450 Repertoire of the Bacterial Phylum Proteobacteria
Source: Int J Mol Sci. 2022 May 22;23(10):5821. doi: 10.3390/ijms23105821 (PMC9148083; doi:10.3390/ijms23105821)
Supplement: Supplementary file 1 [file ijms-23-05821-s001.zip › Supplementary Information/Figure S1.pdf]

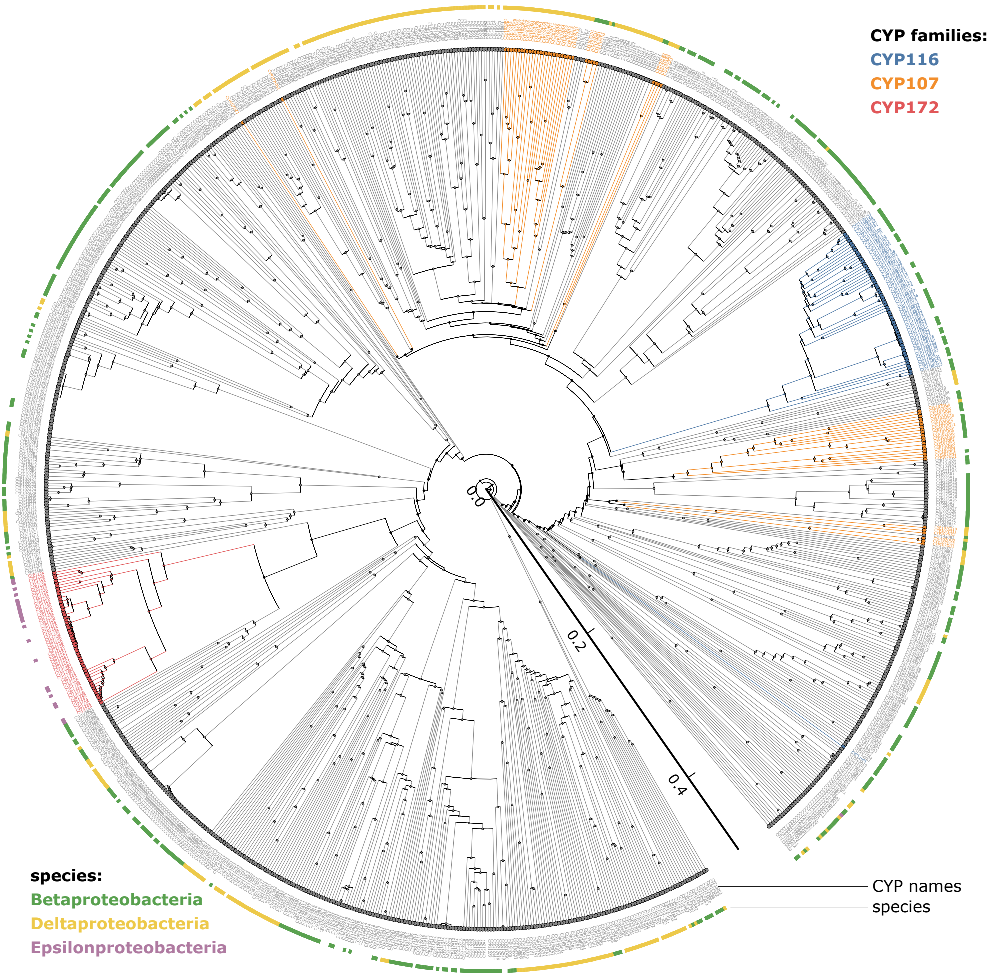

CYP families:
CYP116
CYP107
CYP172
0.0
0.2
0.4
CYP names
species
species:
Betaproteobacteria
Deltaproteobacteria
Epsilonproteobacteria

Figure S1. Information on proteobacterial species and their respective genera was used in the study. Species abbreviations and the P450 count of different species were also presented in the table. Species information is presented as per their class.
