# Supplementary material for: Lifestyles Shape the Cytochrome P450 Repertoire of the Bacterial Phylum Proteobacteria
Source: Int J Mol Sci. 2022 May 22;23(10):5821. doi: 10.3390/ijms23105821 (PMC9148083; doi:10.3390/ijms23105821)
Supplement: Supplementary file 1 [file ijms-23-05821-s001.zip › Supplementary Information/Supplementary Information.pdf]

Article

Lifestyles Shape the Cytochrome P450 Repertoire of the Bacterial Phylum *Proteobacteria*

Siphesihle Msweli [1], Andiswa Chonco [1], Lihle Msweli [1], Puleng Rosinah Syed [2], Rajshekhar Karpoormath [2], Wanping Chen [3], Dominik Gront [4], Bridget Valeria Zinhle Nkosi [1], David R Nelson [5,*] and Khajamohiddin Syed [1,*]

1 Department of Biochemistry and Microbiology, Faculty of Science and Agriculture, University of Zululand, KwaDlangezwa 3886, South Africa; siphesihlemsweli2001@gmail.com (S.M.); angelicaandyc@gmail.com (A.C.); lihle.msweli9991@gmail.com (L.M.); brilenhle@gmail.com (B.V.Z.N.)
2 Department of Pharmaceutical Chemistry, College of Health Sciences, University of KwaZulu-Natal, Durban 4000, South Africa; prosinah@gmail.com (P.R.S.); Karpoormath@ukzn.ac.za (R.K.)
3 Department of Molecular Microbiology and Genetics, University of Göttingen, 37077 Göttingen, Germany; chenwanping1@foxmail.com
4 Biological and Chemical Research Center, Faculty of Chemistry, University of Warsaw, Pasteura 1, 02-093 Warsaw, Poland; dgront@gmail.com
5 Department of Microbiology, Immunology and Biochemistry, University of Tennessee Health Science Center, Memphis, TN 38163, USA
* Correspondence: drnelson1@gmail.com (D.R.N.); khajamohiddinsyed@gmail.com (K.S.); Tel.: +1-901-448-8303 (D.R.N.); +27-035-902-6857 (K.S.)

Citation: Msweli, S.; Chonco, A.; Msweli, L.; Syed, P.R.; Karpoormath, R.; Chen, W.; Gront, D.; Nkosi, B.V.Z.; Nelson, D.R. and Syed, K. Lifestyles Shape the Cytochrome P450 Repertoire of the Bacterial Phylum *Proteobacteria*. *Int. J. Mol. Sci.* **2022**, *23*, 5812. https://doi.org/10.3390/ijms23105821

Academic Editor: Patrick M. Dansette

Received: 30 March 2022
Accepted: 7 May 2022
Published: 22 May 2022

Publisher's Note: MDPI stays neutral with regard to jurisdictional claims in published maps and institutional affiliations.

Copyright: © 2022 by the authors. Submitted for possible open access publication under the terms and conditions of the Creative Commons Attribution (CC BY) license (https://creativecommons.org/licenses/by/4.0/).

Table of Contents

Table S1. Information on proteobacterial species and their respective genera was used in the study. Species abbreviations and the P450 count of different species were also presented in the table. Species information is presented as per their class. 3

Betaproteobacteria 3

Deltaproteobacteria 21

Epsilonproteobacteria 25

Table S2. Comparative analysis of P450 families and subfamilies in different classes of *Proteobacteria*. P450 family and subfamily data for *Alphaproteobacteria* and *Gammaproteobacteria* is retrieved from the published articles [1,2] and used for comparative analysis. 34

Table S3. P450 identified and annotated in this study is presented as per their class. A header follows each P450 sequence consisting of protein ID, database, P450 name, species name, species code, and an abbreviation for the class, such as BP for *Betaproteobacteria*, DP for *Deltaproteobacteria,* and EP for *Epsilonproteobacteria*. 54

Betaproteobacteria 54

Deltaproteobacteria 296

Epsilonproteobacteria 392

Table S4. Identification of P450s that are part of the secondary metabolite biosynthetic gene clusters (sm-BGCs) in alphaproteobacterial species. Reference cluster information was obtained by performing BLAST at the anti-SMASH database [3], as indicated in the materials and methods section. The cluster type and most similar known cluster names available in the anti-SMASH database[3], were listed in the table. 411

Betaproteobacteria 411

Deltaproteobacteria 417

Epsilonproteobacteria 422

Table S1. Information on proteobacterial species and their respective genera was used in the study. Species abbreviations and the P450 count of different species were also presented in the table. Species information is presented as per their class.

Betaproteobacteria

**Genus**	**Number of species**	**Number of species having P450s**	**Species name**	**Species codes**	**P450 count**
Neisseria	40	0	*Neisseria meningitidis* MC58 (serogroup B)	nme	0
			*Neisseria meningitidis alpha*710 (serogroup B)	nmp	0
			Neisseria meningitidis H44/76 (serogroup B)	nmh	0
			Neisseria meningitidis G2136 (serogroup B)	nmd	0
			Neisseria meningitidis M01-240149 (serogroup B)	nmm	0
			Neisseria meningitidis M01-240355 (serogroup B)	nms	0
			Neisseria meningitidis M04-240196 (serogroup B)	nmq	0
			Neisseria meningitidis NZ-05/33 (serogroup B)	nmz	0
			Neisseria meningitidis Z2491 (serogroup A)	nma	0
			Neisseria meningitidis WUE 2594 (serogroup A)	nmw	0
			Neisseria meningitidis 510612 (serogroup A)	nmx	0
			Neisseria meningitidis FAM18 (serogroup C)	nmc	0
			Neisseria meningitidis 053442 (serogroup C)	nmn	0
			Neisseria meningitidis 8013 (serogroup C)	nmt	0
			Neisseria meningitidis alpha14 (cnl strain)	nmi	0
			Neisseria gonorrhoeae FA 1090	ngo	0
			Neisseria gonorrhoeae NCCP11945	ngk	0
			Neisseria lactamica	nla	0
			Neisseria elongata	nel	0
			Neisseria weaveri	nwe	0
			Neisseria mucosa FDAARGOS_260	nsi	0

			Neisseria mucosa ATCC 19696	nmj	0
			Neisseria chenwenguii	nei	0
			Neisseria sp. KEM232	nek	0
			Neisseria flavescens	nfv	0
			Neisseria subflava	nsf	0
			Neisseria zalophi	nzl	0
			Neisseria animalis	naq	0
			Neisseria brasiliensis	nbl	0
			Neisseria zoodegmatis	nzo	0
			Neisseria canis	nci	0
			Neisseria cinerea	ncz	0
			Neisseria animaloris	nani	0
			Neisseria bacilliformis	nbc	0
			Neisseria wadsworthii	nwd	0
			Neisseria shayeganii	nsg	0
			Neisseria musculi	nmus	0
			Neisseria sicca	nsc	0
			Neisseria perflava	npf	0
			Neisseria dentiae	ndn	0
Snodgrassella	1	0	*Snodgrassella alvi*	salv	0
Kingella	2	0	*Kingella kingae*	kki	0
			Kingella oralis	koa	0
Vitreoscilla	2	2	*Vitreoscilla filiformis*	vff	2
			Vitreoscilla sp. C1	vit	1
Eikenella	2	0	*Eikenella corrodens*	ecor	0
			Eikenella exigua	eex	0
Simonsiella	1	0	*Simonsiella muelleri*	smur	0
Alysiella	1	0	*Alysiella filiformis*	aff	0
Conchiformibius	1	0	*Conchiformibius steedae*	cste	0

Aquella	1	0	*Aquella oligotrophica*	nba	0
Paralysiella	1	0	*Paralysiella testudinis*	ptes	0
Chromobacterium	8	7	*Chromobacterium violaceum*	cvi	1
			Chromobacterium vaccinii	cvc	1
			Chromobacterium sp. ATCC 53434	chro	0
			Chromobacterium phragmitis IIBBL 112-1	chri	2
			Chromobacterium phragmitis IIBBL 274-1	chrb	1
			Chromobacterium rhizoryzae	crz	1
			Chromobacterium paludis	chrm	2
			Chromobacterium haemolyticum	chae	1
Iodobacter	2	1	*Iodobacter ciconiae*	iod	0
			Iodobacter fluviatilis	ifl	1
Laribacter	1	0	*Laribacter hongkongensis*	lhk	0
Pseudogulbenkiania	1	1	*Pseudogulbenkiania* sp. NH8B	pse	1
Jeongeupia	1	0	*Jeongeupia* sp. USM3	jeu	0
Aquaspirillum	1	0	*Aquaspirillum* sp. LM1	aql	0
Aquitalea	2	1	*Aquitalea magnusonii*	amah	1
			Aquitalea sp. USM4	aqs	0
Deefgea	1	0	*Deefgea* sp. D17	dee	0
Chitinibacter	2	0	*Chitinibacter bivalviorum*	chiz	0
			Chitinibacter fontanus	cfon	0
Vogesella	1	0	*Vogesella* sp. XCS3	vog	0
Ralstonia	14	13	*Ralstonia solanacearum* GMI1000	rso	0
			Ralstonia solanacearum CFBP2957	rsc	1
			Ralstonia solanacearum PSI07	rsl	3
			Ralstonia solanacearum Po82	rsn	4
			Ralstonia solanacearum CMR15	rsm	3
			Ralstonia solanacearum FQY_4	rse	1
			Ralstonia solanacearum UY031	rsy	2

			Ralstonia pickettii 12J	rpi	2
			Ralstonia pickettii 12D	rpf	2
			Ralstonia pickettii DTP0602	rpj	2
			Ralstonia mannitolilytica	rmn	2
			Ralstonia insidiosa	rin	3
			Ralstonia pseudosolanacearum	rpu	1
			Ralstonia syzygii subsp. *celebesensis*	rsg	2
Cupriavidus	13	13	*Cupriavidus necator* H16	reh	4
			Cupriavidus necator N-1	cnc	7
			Cupriavidus necator NH9	cuh	3
			Cupriavidus pinatubonensis JMP134	reu	2
			Cupriavidus metallidurans	rme	5
			Cupriavidus taiwanensis	cti	3
			Cupriavidus basilensis	cbw	7
			Cupriavidus gilardii	cgd	2
			Cupriavidus sp. USMAHM13	ccup	1
			Cupriavidus malaysiensis	cup	1
			Cupriavidus sp. USMAA2-4	cuu	2
			Cupriavidus pauculus	cpau	6
			Cupriavidus oxalaticus	cox	4
Burkholderia	90	82	*Burkholderia mallei* ATCC 23344	bma	3
			Burkholderia mallei SAVP1	bmv	3
			Burkholderia mallei NCTC 10229	bml	3
			Burkholderia mallei NCTC 10247	bmn	3
			Burkholderia mallei 23344	bmal	2
			Burkholderia mallei 6	bmae	1
			Burkholderia mallei BMQ	bmaq	2
			Burkholderia mallei 2000031063	bmai	3
			Burkholderia mallei FMH 23344	bmaf	2

			Burkholderia mallei NCTC 10247	bmaz	2
			Burkholderia mallei 2002734299	bmab	2
			Burkholderia pseudomallei K96243	bps	2
			Burkholderia pseudomallei 1710b	bpm	2
			Burkholderia pseudomallei 1106a	bpl	3
			Burkholderia pseudomallei 668	bpd	2
			Burkholderia pseudomallei MSHR346	bpr	0
			Burkholderia pseudomallei MSHR305	bpse	2
			Burkholderia pseudomallei MSHR511	bpsm	3
			Burkholderia pseudomallei MSHR146	bpsu	3
			Burkholderia pseudomallei MSHR520	bpsd	3
			Burkholderia pseudomallei 1026b	bpz	3
			Burkholderia pseudomallei BPC006	bpq	3
			Burkholderia pseudomallei NCTC 13179	bpk	3
			Burkholderia pseudomallei HBPUB10134a	bpsh	2
			Burkholderia pseudomallei NAU35A-3	bpsa	2
			Burkholderia pseudomallei A79A	bpso	2
			Burkholderia pseudomallei TSV202	but	2
			Burkholderia thailandensis E264	bte	4
			Burkholderia thailandensis 2002721723	btq	4
			Burkholderia thailandensis E444	btj	4
			Burkholderia thailandensis H0587	btz	3
			Burkholderia thailandensis MSMB121	btd	3
			Burkholderia thailandensis MSMB59	btv	3
			Burkholderia thailandensis E254	bthe	3
			Burkholderia thailandensis USAMRU Malaysia #20	bthm	3
			Burkholderia thailandensis 2003015869	btha	4
			Burkholderia thailandensis 2002721643	bthl	3
			Burkholderia oklahomensis E0147	bok	0

			Burkholderia oklahomensis C6786	boc	2
			Burkholderia mayonis	buu	2
			Burkholderia savannae	bsav	0
			Burkholderia vietnamiensis G4	bvi	1
			Burkholderia vietnamiensis LMG 10929	bve	1
			Burkholderia lata	bur	4
			Burkholderia cenocepacia AU 1054	bcn	1
			Burkholderia cenocepacia HI2424	bch	1
			Burkholderia cenocepacia MC0-3	bcm	5
			Burkholderia cenocepacia J2315	bcj	2
			Burkholderia cenocepacia DDS 22E-1	bcen	2
			Burkholderia cenocepacia DWS 37E-2	bcew	1
			Burkholderia cenocepacia H111	bceo	2
			Burkholderia ambifaria AMMD	bam	1
			Burkholderia ambifaria MC40-6	bac	1
			Burkholderia multivorans ATCC 17616 (Tohoku)	bmj	3
			Burkholderia multivorans ATCC 17616 (JGI)	bmu	4
			Burkholderia multivorans DDS 15A-1	bmk	5
			Burkholderia multivorans ATCC BAA-247	bmul	2
			Burkholderia cepacia GG4	bct	2
			Burkholderia cepacia DDS 7H-2	bced	2
			Burkholderia cepacia ATCC 25416	bcep	0
			Burkholderia dolosa	bdl	1
			Burkholderia pyrrocinia	bpyr	2
			Burkholderia contaminans	bcon	4
			Burkholderia ubonensis	bub	3
			Burkholderia diffusa	bdf	1
			Burkholderia latens	blat	0
			Burkholderia territorii	btei	2

			Burkholderia seminalis	bsem	1
			Burkholderia pseudomultivorans	bpsl	1
			Burkholderia metallica	bmec	0
			Burkholderia stagnalis	bstg	1
			Burkholderia stabilis	bstl	1
			Burkholderia anthina	bann	2
			Burkholderia glumae BGR1	bgl	1
			Burkholderia glumae LMG 2196 = ATCC 33617	bgu	1
			Burkholderia sp. CCGE1001	bug	3
			Burkholderia sp. CCGE1003	bgf	4
			Burkholderia gladioli BSR3	bgd	4
			Burkholderia gladioli ATCC 10248	bgo	3
			Burkholderia sp. YI23	byi	7
			Burkholderia sp. KJ006	buk	1
			Burkholderia sp. RPE67	bue	7
			Burkholderia sp. 2002721687	bul	3
			Burkholderia sp. HB1	buq	2
			Burkholderia plantarii PG1	bgp	2
			Burkholderia plantarii ATCC 43733	bpla	3
			Burkholderia sp. Bp5365	bud	3
			Burkholderia sp. PAMC 26561	bum	0
			Burkholderia sp. PAMC 28687	bui	0
			Burkholderia sp. JP2-270	burk	1
Paraburkholderia	24	23	*Paraburkholderia xenovorans* LB400	bxe	6
			Paraburkholderia xenovorans LB400	bxb	5
			Paraburkholderia phymatum	bph	4
			Paraburkholderia atlantica CCGE1002	bge	2
			Paraburkholderia phenoliruptrix	bpx	3
			Paraburkholderia phytofirmans PsJN	bpy	2

			Paraburkholderia phytofirmans OLGA172	buz	0
			Paraburkholderia fungorum	bfn	6
			Paraburkholderia caribensis	bcai	3
			Paraburkholderia sprentiae	pspw	5
			Paraburkholderia sp. SOS3	para	2
			Paraburkholderia aromaticivorans	parb	1
			Paraburkholderia hospita	phs	7
			Paraburkholderia terrae	pter	9
			Paraburkholderia graminis	pgp	2
			Paraburkholderia caledonica	pcj	4
			Paraburkholderia terricola	pts	3
			Paraburkholderia caffeinilytica	pcaf	2
			Paraburkholderia megapolitana	pmeg	5
			Paraburkholderia tropica	ptro	4
			Paraburkholderia ginsengisoli	pgis	2
			Paraburkholderia acidiphila	pacp	5
			Paraburkholderia acidisoli	pacs	2
			Paraburkholderia edwinii	pew	9
Mycetohabitans	1	1	*Mycetohabitans rhizoxinica*	brh	1
Polynucleobacter	4	0	*Polynucleobacter asymbioticus*	pnu	0
			Polynucleobacter necessarius	pne	0
			Polynucleobacter duraquae	pdq	0
			Polynucleobacter paneuropaeus	poh	0
Pandoraea	14	6	*Pandoraea pnomenusa* 3kgm	ppk	1
			Pandoraea pnomenusa RB38	ppno	0
			Pandoraea pnomenusa DSM 16536	ppnm	0
			Pandoraea pnomenusa	prb	0
			Pandoraea pulmonicola	ppul	1
			Pandoraea sputorum	pspu	0

			Pandoraea apista	papi	1
			Pandoraea vervacti	pve	0
			Pandoraea oxalativorans	pox	0
			Pandoraea thiooxydans	ptx	0
			Pandoraea faecigallinarum	pfg	0
			Pandoraea norimbergensis	pnr	1
			Pandoraea sp. XY-2	pand	1
			Pandoraea fibrosis	pfib	2
Paucimonas	1	0	*Paucimonas lemoignei*	plg	0
Ephemeroptericola	1	0	*Ephemeroptericola cinctiostellae*	hyf	0
Lautropia	1	0	*Lautropia mirabilis*	lmir	0
Mycoavidus	1	0	*Mycoavidus cysteinexigens*	mcys	0
Caballeronia	2	2	*Caballeronia* sp. SBC2	caba	2
			Caballeronia insecticola	buo	2
Limnobacter	1	1	*Limnobacter* sp. SAORIC-580	limn	1
Chitinimonas	1	1	*Chitinimonas arctica*	cari	1
Vallotia	3	0	*Candidatus Vallotia tarda*	vtr	0
			Candidatus Vallotia lariciata	vla	0
			Candidatus Vallotia cooleyia	vcv	0
Bordetella	23	15	*Bordetella pertussis Tohama* I	bpe	1
			Bordetella pertussis CS	bpc	1
			Bordetella pertussis 18323	bper	1
			Bordetella pertussis B1917	bpet	1
			Bordetella pertussis 137	bpeu	1
			Bordetella parapertussis Bpp5	bpar	1
			Bordetella parapertussis 12822	bpa	1
			Bordetella bronchiseptica 253	bbh	1
			Bordetella bronchiseptica RB50	bbr	1
			Bordetella bronchiseptica MO149	bbm	1

			Bordetella bronchiseptica S798	bbx	1
			Bordetella petrii	bpt	1
			Bordetella avium	bav	0
			Bordetella holmesii ATCC 51541	bho	0
			Bordetella holmesii 44057	bhm	0
			Bordetella hinzii	bhz	1
			Bordetella trematum	btrm	1
			Bordetella bronchialis	bbro	0
			Bordetella flabilis	bfz	0
			Bordetella pseudohinzii	bpdz	0
			Bordetella sp. H567	boh	0
			Bordetella genomosp. 13	bgm	0
			Bordetella sp. J329	boj	1
Achromobacter	10	6	*Achromobacter xylosoxidans* A8	axy	4
			Achromobacter xylosoxidans NH44784-1996	axo	1
			Achromobacter xylosoxidans NBRC 15126 = ATCC 27061	axn	1
			Achromobacter xylosoxidans NCTC10807	axx	0
			Achromobacter denitrificans	adt	0
			Achromobacter insolitus	ais	0
			Achromobacter spanius	asw	0
			Achromobacter sp. AONIH1	achr	3
			Achromobacter sp. B7	achb	1
			Achromobacter deleyi	ady	1
Taylorella	5	0	*Taylorella equigenitalis* MCE9	teq	0
			Taylorella equigenitalis ATCC 35865	tea	0
			Taylorella equigenitalis 14/56	teg	0
			Taylorella asinigenitalis MCE3	tas	0
			Taylorella asinigenitalis 14/45	tat	0
Pusillimonas	3	3	*Pusillimonas* sp. T7-7	put	2

			Pusillimonas thiosulfatoxidans	pus	1
			Pusillimonas sp. DMV24BSW_D	pud	2
Advenella	2	1	*Advenella kashmirensis*	aka	1
			Advenella mimigardefordensis	amim	0
Castellaniella	1	1	*Castellaniella defragrans*	cdn	1
Basilea	1	0	*Basilea psittacipulmonis*	bpsi	0
Alcaligenes	4	3	*Alcaligenes faecalis* ZD02	afa	0
			Alcaligenes faecalis JQ135	afq	1
			Alcaligenes aquatilis	aaqu	1
			Alcaligenes ammonioxydans	aamm	1
Paenalcaligenes	1	0	*Paenalcaligenes hominis*	phn	0
Orrella	2	2	*Orrella dioscoreae*	odi	1
			Orrella marina	boz	1
Oligella	2	1	*Oligella urethralis*	our	0
			Oligella ureolytica	oue	1
Pigmentiphaga	2	1	*Pigmentiphaga* sp. H8	pig	3
			Pigmentiphaga aceris	pacr	0
Kerstersia	1	0	*Kerstersia gyiorum*	kgy	0
Paralcaligenes	1	1	*Paralcaligenes* sp. KSB-10	park	2
Rhodoferax	6	3	*Rhodoferax ferrireducens*	rfr	2
			Rhodoferax saidenbachensis	rsb	0
			Rhodoferax antarcticus	rac	0
			Rhodoferax koreense	rhy	0
			Rhodoferax sediminis CHu59-6-5	rhf	3
			Rhodoferax sediminis Gr-4	rhg	1
Polaromonas	5	4	*Polaromonas* sp. JS666	pol	4
			Polaromonas naphthalenivorans	pna	1
			Polaromonas sp. SP1	pos	2
			Polaromonas sp. Pch-P	poo	2

			Polaromonas vacuolata	pvac	0
Acidovorax	13	6	*Acidovorax citrulli*	aav	0
			Acidovorax sp. JS42	ajs	2
			Acidovorax ebreus	dia	1
			Acidovorax avenae	aaa	0
			Acidovorax sp. KKS102	ack	1
			Acidovorax sp. RAC01	acra	1
			Acidovorax carolinensis NA2	acid	0
			Acidovorax carolinensis P4	acip	0
			Acidovorax carolinensis NA3	acin	0
			Acidovorax carolinensis P3	acis	0
			Acidovorax sp. 1608163	acio	1
			Acidovorax monticola	amon	0
			Acidovorax antarcticus	aant	1
Verminephrobacter	1	0	*Verminephrobacter eiseniae*	vei	0
Delftia	5	3	*Delftia acidovorans*	dac	1
			Delftia sp. Cs1-4	del	0
			Delftia tsuruhatensis	dts	0
			Delftia sp. HK171	dhk	1
			Delftia lacustris	dla	1
Variovorax	6	6	*Variovorax paradoxus* S110	vap	2
			Variovorax paradoxus EPS	vpe	2
			Variovorax paradoxus B4	vpd	3
			Variovorax sp. PAMC 28711	vaa	1
			Variovorax boronicumulans	vbo	1
			Variovorax sp. PMC12	vam	3
Comamonas	7	5	*Comamonas thiooxydans*	ctt	1
			Comamonas testosteroni TK102	ctes	3
			Comamonas kerstersii	cke	0

			Comamonas serinivorans	cser	3
			Comamonas sp. NLF-7-7	cof	1
			Comamonas aquatica	caqt	0
			Comamonas odontotermitis	codo	1
Alicycliphilus	2	2	*Alicycliphilus denitrificans* BC	adn	1
			Alicycliphilus denitrificans K601	adk	1
Ramlibacter	1	1	*Ramlibacter tataouinensis*	rta	2
Symbiobacter	1	0	*Candidatus Symbiobacter mobilis*	cbx	0
Ottowia	2	0	*Ottowia* sp. oral taxon 894	oto	0
			Ottowia oryzae	otk	0
Limnohabitans	2	2	*Limnohabitans* sp. 103DPR2	lim	1
			Limnohabitans sp. 63ED37-2	lih	2
Hydrogenophaga	6	5	*Hydrogenophaga* sp. RAC07	hyr	2
			Hydrogenophaga sp. PBC	hyb	0
			Hydrogenophaga sp. LPB0072	hyl	1
			Hydrogenophaga sp. PAMC20947	hyc	1
			Hydrogenophaga pseudoflava	hpse	2
			Hydrogenophaga sp. BPS33	hyn	3
Diaphorobacter	4	1	*Diaphorobacter polyhydroxybutyrativorans*	dpy	0
			Diaphorobacter sp. HDW4A	dih	0
			Diaphorobacter aerolatus	daer	0
			Diaphorobacter ruginosibacter	drg	2
Simplicispira	1	1	*Simplicispira suum*	simp	1
Melaminivora	3	0	*Pulveribacter suum*	melm	0
			Melaminivora sp. SC2-9	mela	0
			Melaminivora jejuensis	mje	0
Serpentinomonas	2	2	*Serpentinomonas raichei*	cbaa	1
			Serpentinomonas mccroryi	cbab	1
Schlegelella	2	2	*Schlegelella thermodepolymerans*	sthm	3

			Schlegelella brevitalea	pbh	2
Kinneretia	1	1	*Kinneretia* sp. DAIF2	kia	2
Methylibium	2	1	*Methylibium petroleiphilum*	mpt	0
			Methylibium sp. Pch-M	metp	1
Herminiimonas	1	0	*Herminiimonas arsenicoxydans*	har	0
Janthinobacterium	9	7	*Janthinobacterium* sp. Marseille	mms	0
			Janthinobacterium agaricidamnosum	jag	1
			Janthinobacterium sp. B9-8	jab	0
			Janthinobacterium sp. 1_2014MBL_MicDiv	jaz	2
			Janthinobacterium sp. LM6	jal	1
			Janthinobacterium svalbardensis	jsv	1
			Janthinobacterium sp. 17J80-10	jaj	3
			Janthinobacterium tructae	jas	1
			Janthinobacterium lividum	jlv	1
Herbaspirillum	7	5	*Herbaspirillum seropedicae* SmR1	hse	1
			Herbaspirillum seropedicae Z67	hsz	1
			Herbaspirillum hiltneri	hht	0
			Herbaspirillum rubrisubalbicans	hrb	1
			Herbaspirillum sp. meg3	hee	0
			Herbaspirillum huttiense	hhf	1
			Herbaspirillum frisingense	hfr	1
Zinderia	1	0	*Candidatus Zinderia insecticola*	zin	0
Collimonas	3	3	*Collimonas fungivorans*	cfu	1
			Collimonas arenae	care	1
			Collimonas pratensis	cpra	2
Massilia	10	6	*Massilia* sp. NR 4-1	mnr	0
			Massilia sp. WG5	masw	0
			Massilia violaceinigra	mass	0
			Massilia armeniaca	masz	2

			Massilia oculi	mtim	0
			Massilia sp. YMA4	masy	3
			Massilia albidiflava	mali	2
			Massilia umbonata	mum	6
			Massilia flava	mfla	2
			Massilia plicata	mpli	1
Duganella	1	1	*Duganella* sp. AF9R3	dug	2
Oxalobacter	1	0	*Oxalobacter formigenes*	ofo	0
Undibacterium	2	2	*Undibacterium parvum*	upv	2
			Undibacterium piscinae	upi	1
Noviherbaspirillum	1	1	*Noviherbaspirillum* sp. UKPF54	nok	1
Glaciimonas	1	1	*Glaciimonas* sp. PAMC28666	glc	1
Sutterella	3	0	*Sutterella megalosphaeroides*	sutt	0
			Sutterella faecalis	sutk	0
			Sutterella wadsworthensis	sws	0
Leptothrix	1	1	*Leptothrix cholodnii*	lch	1
Thiomonas	2	1	*Thiomonas intermedia*	tin	0
			Thiomonas arsenitoxydans	thi	1
Rubrivivax	2	1	*Rubrivivax gelatinosus*	rge	1
			Rubrivivax benzoatilyticus	rbn	0
Roseateles	1	0	*Roseateles depolymerans*	rdp	0
Paucibacter	1	0	*Paucibacter* sp. KCTC 42545	pkt	0
Mitsuaria	1	0	*Mitsuaria* sp. 7	miu	0
Rhizobacter	1	1	*Rhizobacter gummiphilus*	rgu	1
Aquabacterium	1	1	*Aquabacterium olei*	aon	1
Sphaerotilus	1	1	*Sphaerotilus natans* subsp. *sulfidivorans*	snn	2
Xylophilus	1	0	*Xylophilus rhododendri*	xyk	0
Tepidimonas	1	0	*Tepidimonas taiwanensis*	ttw	0
Ideonella	1	1	*Ideonella dechloratans*	idc	1

unclassified Burkholderiales	2	1	*Burkholderiales bacterium* GJ-E10	bbag	1
			Burkholderiales bacterium YL45	bbay	0
Nitrosomonas	7	3	*Nitrosomonas europaea*	neu	0
			Nitrosomonas eutropha	net	0
			Nitrosomonas sp. AL212	nit	1
			Nitrosomonas sp. Is79A3	nii	1
			Nitrosomonas communis	nco	1
			Nitrosomonas ureae	nur	0
			Nitrosomonas stercoris	nst	0
Nitrosospira	2	2	*Nitrosospira multiformis*	nmu	1
			Nitrosospira lacus	nlc	1
Sulfuritalea	1	0	*Sulfuritalea hydrogenivorans*	shd	0
Methyloversatilis	1	1	*Methyloversatilis* sp. RAC08	metr	1
Denitratisoma	1	1	*Denitratisoma oestradiolicum*	doe	2
Thiobacillus	1	0	*Thiobacillus denitrificans*	tbd	0
Methylobacillus	1	1	*Methylobacillus flagellatus*	mfa	1
Methylotenera	2	0	*Methylotenera mobilis*	mmb	0
			Methylotenera versatilis	meh	0
Methylovorus	2	0	*Methylovorus glucosetrophus*	mei	0
			Methylovorus sp. MP688	mep	0
Methylopumilus	2	1	*Candidatus Methylopumilus turicensis*	mbac	1
			Candidatus Methylopumilus planktonicus	mbat	0
Methylophilus	1	1	*Methylophilus* sp. TWE2	meu	1
Methyloradius	1	1	*Methyloradius palustris*	mpau	1
Sideroxydans	1	0	*Sideroxydans lithotrophicus*	slt	0
Gallionella	1	0	*Gallionella capsiferriformans*	gca	0
Ferriphaselus	1	0	*Ferriphaselus amnicola*	fam	0
Nitrotoga	1	0	*Candidatus Nitrotoga* sp. AM1P	nim	0

Ferrigenium	1	0	*Ferrigenium kumadai*	fku	0
Sulfuricella	1	0	*Sulfuricella denitrificans*	sdr	0
Sulfuriferula	3	0	*Sulfuriferula* sp. AH1	sulf	0
			Sulfuriferula plumbiphila	splb	0
			Sulfuriferula nivalis	sniv	0
Sulfurimicrobium	1	0	*Sulfurimicrobium lacus* skT11	slac	0
Usitatibacter	2	0	*Usitatibacter rugosus*	uru	0
			Usitatibacter palustris	upl	0
Azospira	3	0	*Azospira oryzae*	dsu	0
			Azospira restricta	ares	0
			Azospira inquinata	aiq	0
Rugosibacter	1	0	*Rugosibacter aromaticivorans*	rbu	0
Oryzomicrobium	1	0	*Oryzomicrobium terrae*	otr	0
Niveibacterium	1	0	*Niveibacterium microcysteis*	niv	0
Aromatoleum	4	3	*Aromatoleum aromaticum*	eba	2
			Aromatoleum bremense	abre	0
			Aromatoleum petrolei	apet	3
			Aromatoleum pumilum	atw	1
unclassified Rhodocyclaceae	1	0	*Rhodocyclaceae bacterium Thauera-like*	rbh	0
Dechloromonas	2	0	*Dechloromonas aromatica*	dar	0
			Dechloromonas sp. HYN0024	dey	0
Quatrionicoccus	1	0	*Quatrionicoccus australiensis*	qau	0
Ferribacterium	1	0	*Ferribacterium limneticum*	fle	0
Azoarcus	7	5	*Azoarcus olearius* BH72	azo	1
			Azoarcus olearius DQS4	aoa	1
			Azoarcus sp. KH32C	aza	0
			Azoarcus sp. CIB	azi	1
			Azoarcus communis	acom	2

			Azoarcus sp. DN11	azd	3
			Azoarcus sp. DD4	azr	0
Thauera	5	4	*Thauera* sp. MZ1T	tmz	1
			Thauera humireducens	thu	0
			Thauera chlorobenzoica	tcl	1
			Thauera sp. K11	thk	2
			Thauera aromatica	tak	1
Nitrogeniibacter	1	0	*Nitrogeniibacter mangrovi*	azq	0
unclassified Zoogloeaceae	1	0	*Zoogloeaceae bacteirum* Par-f-2	zpa	0
Fluviibacter	1	0	*Fluviibacter phosphoraccumulans*	fpho	0
Ferrovum	1	0	*Ferrovum myxofaciens*	fmy	0
Accumulibacter	1	0	*Accumulibacter phosphatis*	app	0
Tremblaya	3	0	*Candidatus Tremblaya princeps* PCIT	tpn	0
			Candidatus Tremblaya princeps PCVAL	tpq	0
			Candidatus Tremblaya phenacola	tpj	0
Kinetoplastibacterium	8	0	*Candidatus Kinetoplastibacterium crithidii (ex Angomonas deanei* ATCC 30255)	kci	0
			Candidatus Kinetoplastibacterium crithidii TCC036E	kct	0
			Candidatus Kinetoplastibacterium blastocrithidii (ex Strigomonas culicis)	kbl	0
			Candidatus Kinetoplastibacterium blastocrithidii TCC012E	kbt	0
			Candidatus Kinetoplastibacterium desouzaii	kde	0
			Candidatus Kinetoplastibacterium galatii	kga	0
			Candidatus Kinetoplastibacterium oncopeltii	kon	0
			Candidatus Kinetoplastibacterium sorsogonicusi	kso	0
Profftella	1	0	*Candidatus Profftella armatura*	ssdc	0
Nasuia	1	0	*Candidatus Nasuia deltocephalinicola*	ndl	0
Vidania	1	0	*Candidatus Vidania fulgoroideae*	vfg	0
Desulfobacillus	1	0	*Candidatus Desulfobacillus denitrificans*	ddz	0

unclassified Betaproteobacteria	3	0	*Beta proteobacterium* CB	bprc	0
			Betaproteobacteria bacterium UKL13-2	beb	0
137			*Betaproteobacteria bacterium* GR16-43	beba	0

Deltaproteobacteria

Genus	Number of species	Number of species having P450s	Species name	Species codes	Number of p450s
Geobacter	7		*Geobacter sulfurreducens* PCA	gsu	0
			Geobacter sulfurreducens KN400	gsk	0
			Geobacter metallireducens	gme	0
			Geobacter sp. M21	gem	0
			Geobacter sp. M18	geb	0
			Geobacter pickeringii	gpi	0
			Geobacter anodireducens	gao	0
Geotalea	2		*Geobacter uraniireducens*	gur	0
			Geobacter daltonii FRC-32	geo	0
Trichlorobacter	1		*Trichlorobacter lovleyi*	glo	0
Citrifermentans	2		*Citrifermentans bemidjiense*	gbm	0
			Citrifermentans bremense	gbn	0
Geoalkalibacter	1		*Geoalkalibacter subterraneus*	gsb	0

Geomonas	3		*Geomonas oryzisoli*	ger	0
			Geomonas subterranea	gsub	0
			Geomonas nitrogeniifigens	gnt	0
Syntrophotalea	3		*Pelobacter carbinolicus*	pca	0
			Syntrophotalea acetylenica	pace	0
			Syntrophotalea acetylenivorans	pef	0
Pelobacter	1		*Pelobacter propionicus*	ppd	0
Desulfuromonas	3		*Desulfuromonas soudanensis*	des	0
			Desulfuromonas sp. DDH964	deu	0
			Desulfuromonas versatilis	dve	0
Desulfovibrio	11		*Desulfovibrio vulgaris* Hildenborough	dvu	0
			Desulfovibrio vulgaris DP4	dvl	0
			Desulfovibrio vulgaris Miyazaki F	dvm	0
			Desulfovibrio vulgaris RCH1	dvg	0
			Desulfovibrio desulfuricans ATCC 27774	dds	0
			Desulfovibrio fairfieldensis	dfi	0
			Desulfovibrio piger	dpg	0
			Desulfovibrio sp. G11	def	0
			Candidatus Desulfovibrio trichonymphae	dtr	0
			Desulfovibrio ferrophilus	dfl	0
			Desulfovibrio sulfodismutans	dsd	0
Solidesulfovibrio	2		*Solidesulfovibrio magneticus*	dma	0
			Solidesulfovibrio carbinolicus	dcb	0
Megalodesulfovibrio	1		*Megalodesulfovibrio gigas*	dgg	0
Oleidesulfovibrio	1		*Oleidesulfovibrio alaskensis*	dde	0
Oceanidesulfovibrio	1		*Oceanidesulfovibrio marinus*	dms	0
Maridesulfovibrio	2		*Maridesulfovibrio salexigens*	dsa	0
			Desulfovibrio hydrothermalis	dhy	0
Desulfocurvibacter	1	1	*Desulfocurvibacter africanus subsp. africanus*	daf	1

Pseudodesulfovibrio	6		*Pseudodesulfovibrio aespoeensis*	das	0
			Pseudodesulfovibrio piezophilus	dpi	0
			Pseudodesulfovibrio indicus	dej	0
			Pseudodesulfovibrio profundus	pprf	0
			Pseudodesulfovibrio cashew	psel	0
			Pseudodesulfovibrio mercurii	ddn	0
Lawsonia	2		*Lawsonia intracellularis* PHE/MN1-00	lip	0
			Lawsonia intracellularis N343	lir	0
Desulfolutivibrio	1		*Desulfolutivibrio sulfoxidireducens*	dsx	0
Paradesulfovibrio	1		*Paradesulfovibrio bizertensis*	pbiz	0
Desulfomicrobium	2		*Desulfomicrobium baculatum*	dba	0
			Desulfomicrobium orale	doa	0
Desulfohalobium	1		*Desulfohalobium retbaense*	drt	0
Desulfotalea	1		*Desulfotalea psychrophila*	dps	0
Desulfocapsa	1		*Desulfocapsa sulfexigens*	dsf	0
Desulfurivibrio	1		*Desulfurivibrio alkaliphilus*	dak	0
Desulfobulbus	3		*Desulfobulbus propionicus*	dpr	0
			Desulfobulbus oralis	deo	0
			Desulfobulbus oligotrophicus	dog	0
Desulfomarina	1		*Desulfomarina profundi*	dbk	0
Desulfococcus	2		*Desulfococcus oleovorans*	dol	0
			Desulfococcus multivorans	dml	0
Desulfatibacillum	1		*Desulfatibacillum aliphaticivorans*	dal	0
Desulforapulum	1		*Desulforapulum autotrophicum*	dat	0
Desulfobacula	1		*Desulfobacula toluolica*	dto	0
Desulfosarcina	3		*Desulfosarcina ovata* subsp. *sediminis*	dov	0
			Desulfosarcina widdelii	dwd	0
			Desulfosarcina alkanivorans	dalk	0
Desulfonema	2		*Desulfonema limicola*	dli	0

			Desulfonema magnum	dmm	0
Desulfoluna	1		*Desulfoluna* sp. ASN36	dek	0
Anaeromyxobacter	4		*Anaeromyxobacter dehalogenans* 2CP-C	ade	0
			Anaeromyxobacter dehalogenans 2CP-1	acp	0
			Anaeromyxobacter sp. Fw109-5	afw	0
			Anaeromyxobacter sp. K	ank	0
Myxococcus	4	4	*Myxococcus xanthus*	mxa	7
			Myxococcus stipitatus	msd	11
			Myxococcus hansupus	mym	4
			Myxococcus fulvus	mfb	2
Corallococcus	3	4	*Corallococcus coralloides*	ccx	10
			Corallococcus macrosporus HW-1	mfu	2
			Corallococcus macrosporus DSM 14697	mmas	1
Stigmatella	1	1	*Stigmatella aurantiaca*	sur	17
Archangium	2	2	*Archangium gephyra*	age	56
			Archangium violaceum	avm	43
Melittangium	1	1	*Melittangium boletus*	mbd	28
Cystobacter	1	1	*Cystobacter fuscus*	cfus	42
Vulgatibacter	1		*Vulgatibacter incomptus*	vin	0
Sorangium	2	2	*Sorangium cellulosum* So ce56	scl	19
			Sorangium cellulosum So0157-2	scu	8
Chondromyces	1	1	*Chondromyces crocatus*	ccro	26
Sandaracinus	1	1	*Sandaracinus amylolyticus*	samy	6
Labilithrix	1	1	*Labilithrix luteola*	llu	5
Minicystis	1	1	*Minicystis rosea*	mrm	24
Haliangium	1	1	*Haliangium ochraceum*	hoh	17
Syntrophus	1		*Syntrophus aciditrophicus*	sat	0
Desulfobacca	1		*Desulfobacca acetoxidans*	dao	0
Desulfomonile	1		*Desulfomonile tiedjei*	dti	0

Syntrophobacter	1		*Syntrophobacter fumaroxidans*	sfu	0
Desulfoglaeba	1		*Desulfoglaeba alkanexedens*	dax	0
Desulfarculus	1		*Desulfarculus baarsii*	dbr	0
Hippea	1		*Hippea maritima*	hmr	0
Desulfurella	1		*Desulfurella acetivorans*	dav	0
Bradymonas	1	1	*Bradymonas sediminis*	bsed	1
Persicimonas	1	1	*Persicimonas caeni*	pcay	3

Epsilonproteobacteria

**Genus**	**Number of species**	**Number of species having P450s**	**Species name**	**Species code**	**Number of p450s**
Helicobacter	78	1	*Helicobacter pylori* 26695	hpy	0
			Helicobacter pylori 26695	heo	0
			Helicobacter pylori J99	hpj	0
			Helicobacter pylori HPAG1	hpa	0
			Helicobacter pylori Shi470	hps	0
			Helicobacter pylori Shi112	hhp	0
			Helicobacter pylori Shi169	hhq	0
			Helicobacter pylori Shi417	hhr	0
			Helicobacter pylori G27	hpg	0

			Helicobacter pylori P12	hpp	0
			Helicobacter pylori B38	hpb	0
			Helicobacter pylori B8	hpl	0
			Helicobacter pylori PeCan4	hpc	0
			Helicobacter pylori PeCan18	hca	0
			Helicobacter pylori SJM180	hpm	0
			Helicobacter pylori ELS37	hpe	0
			Helicobacter pylori 35A	hpo	0
			Helicobacter pylori 908	hpi	0
			Helicobacter pylori 2017	hpq	0
			Helicobacter pylori 2018	hpw	0
			Helicobacter pylori Cuz20	hpu	0
			Helicobacter pylori F16	hef	0
			Helicobacter pylori F30	hpf	0
			Helicobacter pylori F32	heq	0
			Helicobacter pylori F57	hex	0
			Helicobacter pylori Sat464	hpt	0
			Helicobacter pylori 52	hpz	0
			Helicobacter pylori v225d	hpv	0
			Helicobacter pylori 83	hpx	0
			Helicobacter pylori SNT49	hen	0
			Helicobacter pylori Lithuania75	hph	0
			Helicobacter pylori Gambia94/24	heg	0
			Helicobacter pylori India7	hpn	0
			Helicobacter pylori Puno120	hep	0
			Helicobacter pylori Puno135	heu	0
			Helicobacter pylori SouthAfrica7	hes	0
			Helicobacter pylori SouthAfrica20	hpys	0
			Helicobacter pylori HUP-B14	hcn	0

			Helicobacter pylori 51	hpd	0
			Helicobacter pylori XZ274	hey	0
			Helicobacter pylori Rif1	her	0
			Helicobacter pylori Rif2	hei	0
			Helicobacter pylori Aklavik117	hpya	0
			Helicobacter pylori Aklavik86	hpyk	0
			Helicobacter pylori OK113	hpyo	0
			Helicobacter pylori OK310	hpyl	0
			Helicobacter pylori oki102	hpyb	0
			Helicobacter pylori oki112	hpyc	0
			Helicobacter pylori oki128	hpyd	0
			Helicobacter pylori oki154	hpye	0
			Helicobacter pylori oki422	hpyf	0
			Helicobacter pylori oki673	hpyg	0
			Helicobacter pylori oki828	hpyh	0
			Helicobacter pylori oki898	hpyj	0
			Helicobacter pylori UM032	hpyr	0
			Helicobacter pylori UM037	hpyi	0
			Helicobacter pylori UM066	hpyu	0
			Helicobacter pylori UM299	hpym	0
			Helicobacter pylori UM298	hem	0
			Helicobacter pylori BM012A	heb	0
			Helicobacter pylori BM012S	hez	0
			Helicobacter hepaticus	hhe	0
			Helicobacter acinonychis	hac	0
			Helicobacter mustelae	hms	0
			Helicobacter felis	hfe	0
			Helicobacter bizzozeronii	hbi	0
			Helicobacter cetorum MIT 00-7128	hce	0

			Helicobacter cetorum MIT 99-5656	hcm	0
			Helicobacter cinaedi PAGU611	hcp	0
			Helicobacter cinaedi CCUG 18818 = ATCC BAA-847	hcb	0
			Helicobacter heilmannii	hhm	0
			Helicobacter typhlonius	hty	0
			Helicobacter bilis	hbl	0
			Helicobacter apodemus	had	0
			Helicobacter enhydrae	het	0
			Helicobacter cholecystus	hcl	0
			Helicobacter winghamensis	hwi	1
			Helicobacter suis	hsh	0
Wolinella	1	1	*Wolinella succinogenes*	wsu	0
Sulfurimonas	9	9	*Sulfurimonas denitrificans*	tdn	0
			Sulfurimonas autotrophica	sua	0
			Sulfurimonas xiamenensis	suln	0
			Sulfurimonas lithotrophica	sulg	0
			Sulfurimonas sp. CVO	sulc	0
			Sulfurimonas paralvinellae	spal	0
			Sulfurimonas sediminis	ssei	0
			Candidatus Sulfurimonas baltica	sbal	0
			Candidatus Sulfurimonas marisnigri	smas	0
Sulfuricurvum	2	2	*Sulfuricurvum kujiense*	sku	0
			Candidatus Sulfuricurvum sp. RIFRC-1	sulr	0
Campylobacter	78	51	*Campylobacter jejuni* subsp. *jejuni* NCTC 11168 = ATCC 700819	cje	1
			Campylobacter jejuni subsp. *jejuni* NCTC 11168-BN148	cjb	1
			Campylobacter jejuni subsp. *jejuni* 81-176	cjj	1
			Campylobacter jejuni subsp. *jejuni* 81116	cju	1

			Campylobacter jejuni subsp. *jejuni* ICDCCJ07001	cjn	1
			Campylobacter jejuni subsp. *jejuni* IA3902	cji	1
			Campylobacter jejuni subsp. *jejuni* M1	cjm	1
			Campylobacter jejuni subsp. *jejuni* S3	cjs	1
			Campylobacter jejuni subsp. *jejuni* PT14	cjp	1
			Campylobacter jejuni subsp. *jejuni* 00-2426	cjej	1
			Campylobacter jejuni subsp. *jejuni* 00-2538	cjeu	1
			Campylobacter jejuni subsp. *jejuni* 00-2544	cjen	1
			Campylobacter jejuni subsp. *jejuni* 00-2425	cjei	1
			Campylobacter jejuni subsp. *jejuni* R14	cjer	1
			Campylobacter jejuni subsp. *jejuni* MTVDSCj20	cjv	1
			Campylobacter jejuni subsp. *jejuni* YH001	cjy	0
			Campylobacter jejuni subsp. *jejuni* 35925B2	cjq	0
			Campylobacter jejuni subsp. *jejuni* 00-1597	cjl	1
			Campylobacter jejuni subsp. *jejuni* 00-6200	cjw	1
			Campylobacter jejuni RM1221	cjr	1
			Campylobacter jejuni subsp. *doylei* 269.97	cjd	1
			Campylobacter jejuni 32488	cjz	1
			Campylobacter jejuni 4031	cjx	1
			Campylobacter fetus subsp. *fetus* 82-40	cff	0
			Campylobacter fetus subsp. *fetus* 04/554	cft	0
			Campylobacter fetus subsp. *venerealis* cfvi03/293	cfv	0
			Campylobacter fetus subsp. *venerealis* 97/608	cfx	0
			Campylobacter fetus subsp. *venerealis* 84-112	cfz	0
			Campylobacter fetus subsp. *testudinum* 03-427	camp	0
			Campylobacter fetus subsp. *testudinum* pet-3	cfp	0
			Campylobacter curvus	ccv	0
			Campylobacter hominis	cha	0
			Campylobacter concisus 13826	cco	0

			Campylobacter concisus ATCC 33237	ccoc	0
			Campylobacter lari RM2100	cla	1
			Campylobacter lari RM16701	clr	1
			Campylobacter lari RM16712	clm	1
			Campylobacter lari CCUG 22395	clq	0
			Campylobacter lari NCTC 11845	cln	1
			Campylobacter lari subsp. *concheus* LMG 11760	cll	1
			Campylobacter coli 76339	ccol	1
			Campylobacter coli CVM N29710	ccc	1
			Campylobacter coli 15-537360	ccq	1
			Campylobacter coli RM1875	ccf	1
			Campylobacter coli RM4661	ccy	1
			Campylobacter coli RM5611	ccoi	1
			Campylobacter coli FB1	ccof	1
			Campylobacter coli OR12	ccoo	1
			Campylobacter iguaniorum	caj	0
			Campylobacter insulaenigrae	cis	1
			Campylobacter volucris	cvo	1
			Campylobacter peloridis	cpel	1
			*Campylobacte*r sp. RM16704	camr	1
			Campylobacter subantarcticus LMG 24374	csm	1
			Campylobacter subantarcticus LMG 24377	csf	1
			Campylobacter gracilis	cgra	0
			Campylobacter ureolyticus	cure	0
			Campylobacter hyointestinalis	chyo	0
			Campylobacter helveticus	chv	1
			Campylobacter sputorum	cspf	0
			Campylobacter pinnipediorum	cpin	0
			Campylobacter cuniculorum	ccun	1

			Campylobacter lanienae	clx	1
			Campylobacter avium	cavi	1
			Campylobacter hepaticus	chw	1
			Campylobacter sp. RM12175	camz	1
			Campylobacter sp. RM6137	camy	1
			Campylobacter ornithocola	coj	1
			Campylobacter upsaliensis	cux	1
			Campylobacter rectus	crx	0
			Campylobacter geochelonis	cgeo	0
			Campylobacter blaseri	cbla	0
			Campylobacter corcagiensis	ccor	0
			Campylobacter armoricus	carm	1
			Campylobacter mucosalis	cmuc	0
			Campylobacter showae	csho	0
			Candidatus Campylobacter infans	cinf	0
			Campylobacter novaezeelandiae	cnv	1
Sulfurospirillum	7	7	*Sulfurospirillum deleyianum*	sdl	0
			Sulfurospirillum barnesii	sba	0
			Sulfurospirillum multivorans	smul	0
			Sulfurospirillum halorespirans	shal	0
			Sulfurospirillum sp. SL2-1	suls	0
			Sulfurospirillum sp. JPD-1	sulj	0
			Sulfurospirillum sp. ACSTCE	sult	0
Aliiarcobacter	11	11	*Aliarcobacter butzleri* RM4018	abu	0
			Aliarcobacter butzleri ED-1	abt	0
			Aliarcobacter butzleri 7h1h	abl	0
			Aliarcobacter skirrowii	ask	0
			Aliarcobacter trophiarum	atp	0
			Aliarcobacter cibarius	acib	0

			Aliarcobacter cryaerophilus	acre	0
			Aliarcobacter lanthieri	alan	0
			Arcobacter porcinus	apoc	0
			Aliarcobacter faecis	afc	0
			Aliarcobacter thereius	athr	0
Arcobacter	7	7	*Arcobacter nitrofigilis*	ant	0
			Arcobacter ellisii	aell	0
			Arcobacter aquimarinus	aaqi	0
			Arcobacter suis	asui	0
			Arcobacter cloacae	aclo	0
			Arcobacter venerupis	avp	0
			Arcobacter defluvii	adz	0
Poseidonibacter	2	2	*Poseidonibacter lekithochrous*	alk	0
			Poseidonibacter parvus	alp	0
Malaciobacter	6	1	*Malaciobacter halophilus*	ahs	0
			Malaciobacter mytili	amyt	1
			Malaciobacter marinus	amar	0
			Malaciobacter canalis	acaa	0
			Malaciobacter molluscorum	amol	0
			Malaciobacter pacificus	apai	0
Halarcobacter	3	3	*Halarcobacter bivalviorum*	hbv	0
			Halarcobacter ebronensis	hebr	0
			Halarcobacter anaerophilus	aana	0
Pseudoarcobacter	1	1	*Pseudoarcobacter acticola*	paco	0
unclassified Arcobacter	1	1	*Arcobacter* sp. L	arc	0
Hydrogenimonas	1	1	*Hydrogenimonas* sp. MAG	hyo	0
Nitratifractor	1	1	*Nitratifractor salsuginis*	nsa	0
Sulfurovum	3	3	*Sulfurovum* sp. NBC37-1	sun	0

			Sulfurovum lithotrophicum	slh	0
			Sulfurovum indicum	sinu	0
Nitratiruptor	1	1	*Nitratiruptor* sp. SB155-2	nis	0
Nautilia	2	2	*Nautilia profundicola*	nam	0
			Nautilia sp. PV-1	nap	0
Caminibacter	2	2	*Caminibacter mediatlanticus*	cmed	0
			Caminibacter pacificus	cpaf	0

Table S2. Comparative analysis of P450 families and subfamilies in different classes of *Proteobacteria*. P450 family and subfamily data for *Alphaproteobacteria* and *Gammaproteobacteria* are retrieved from the published articles [1, 2] and used for comparative analysis.

**Family**	**Subfamily**	***Alphaproteobacteria***	***Betaproteobacteria***	***Gammaproteobacteria***	***Deltaproteobacteria***	***Epsilonproteobacteria***
CYP101	M			1		
	B	1				
	C	3				
	D	2				
	E	2				
	Q	1				
	R	1				
	F		2			
	N		2			
	P				1	
CYP1007	B				1	
	C				2	
	D				1	
	E				1	
	F				1	
	G				1	
CYP1010	A	1				
CYP1011	E				3	
	F				1	
CYP1013	A		42			
CYP1017	A	1				
CYP102	J		5	1	1	
	A	10	6			

	E		14			
	K	1				
	AC	1				
	M				6	
CYP103	A	4				
CYP1036	B	1				
	C	1				
CYP104	A	13				
CYP1043	C			1		
	A		1		1	
CYP1049	A		3	1		
CYP105	BQ			4		
	BF	3				
	CX	1				
	B		1			
	CV		4			
	CW		4			
	T		16			
	AC				1	
	EK				1	
CYP1061	B	2				
CYP1068	A	4				
CYP1069	A				6	
	B				1	
	C				2	
	D				1	
	E				1	
	F				1	
	G				1	

	H				1	
	J				2	
CYP107	DJ			3		
	E			4		
	HT			1		
	S			15		
	AN	8				
	JE	1				
	DG		12			
	DN		5			
	DQ		3			
	NN		1			
	NP		5			
	NQ		1			
	NX		1			
	AM				1	
	CJ				2	
	DL				7	
	DP				9	
	EN				2	
	HU				2	
	HV				4	
	HW				1	
	HX				3	
	HY				1	
	HZ				1	
	JA				1	
CYP1075	A	1				
CYP1076	A	1				

CYP1077	A	1	1			
CYP1078	A	7				
	C	1				
CYP108	R			1		
	A	2				
	D	6				
	G	18				
	L	1				
	P	2				
	U	1				
	V	1				
	W	1				
	X	5				
	AB		1			
	E		2			
	S		12			
	Z		1			
CYP1081	B	2				
CYP1082	A	1				
CYP1083	A	3				
	C	2				
CYP1086	B	1				
CYP1088	B	2				
CYP1089	A	1				
CYP109	AK				3	
	C				5	
	D				2	
	N				4	
	P				1	

	Q				4	
	R				7	
	S				1	
CYP1090	C	2				
CYP1091	A	3				
CYP1094	A	3				
	B	1				
CYP1097	B			1		
	A		16			
	C		1			
CYP1098	A	6				
	B		8			
	D		1			
CYP110	J		1		2	
	V				1	
	AD				1	
	AU				1	
	AV				1	
	H				3	
	R				5	
	S				1	
	T				1	
	U				2	
	W				1	
CYP1101	A	29				
CYP1104	B		1	3		
	A	4				
	C	3				
	E	3				

CYP1107	A	12				
	B	3				
	C	1				
CYP1108	A		1			
CYP111	A	1		1		
	B	1				
CYP1110	A		2			
CYP1111	A	5	2			
	B	1				
	C	1				
CYP1112	A		4			
CYP1116	B	3				
CYP1118	B	1				
CYP112	A	23		3		
CYP1135	A	1				
	B		1			
CYP1137	A	1				
CYP1138	A		3	1		
	B	2				
CYP114	A	22		4		
CYP1141	A	1				
CYP1142	A			1		
CYP1145	A	1				
CYP115	A	1		3		
CYP1155	B	1				
	A		1			
CYP1157	C			1		
	D		5			
CYP116	B	1	58	2		

	F		1			
CYP1164	A			1		
CYP1165	A			1		
	B			1		
CYP117	A	22		3		
	C		4			
	B				1	
	D				1	
	E				4	
CYP1170	A	7				
CYP1171	A	1				
CYP1172	A			1		
CYP1173	A	1				
CYP1174	A	2				
CYP1175	A	1				
CYP1176	B		1			
CYP1179	A			1		
	B		4			
CYP1180	A		1			
CYP1181	A	1				
CYP1182	A	1				
CYP1183	A		1			
CYP1186	A	2				
CYP1187	A	1				
CYP1199	A		1	2		
	C		1			
CYP120	F				1	
	G				5	
	H				1	

CYP1200	B			1		
	A		1			
CYP1201	A			1		
	B			1		
CYP1202	A			1		
	B		1			
CYP1204	A	2				
CYP1206	B	1				
CYP1220	A		13			
	C				1	
CYP1221	B	2				
CYP1224	J				3	
CYP1225	A		2	1		
CYP1229	A	1		1		
	B	2				
	C	1				
CYP1232	F		1			
CYP1234	A			5		
	B			1		
CYP1236	A	1				
CYP1246	A	2	1			
CYP1247	A			9		
CYP1248	B				2	
CYP125	U		1			
	E				2	
	N				1	
CYP125	P	1				
CYP1250	A	1				
CYP1258	A	1				

	B		1			
CYP126	C			4	1	
	B				2	
CYP1261	B			1		
CYP1263	B		1			
	C				1	
CYP127	A	18				
	C	1				
CYP1275	B	1				
CYP1278	A			1		
CYP1281	A	1				
CYP1284	B				7	
CYP1286	A				2	
	B				1	
CYP1291	B	3				
	C	2				
CYP1295	B		1			
CYP1298	A				1	
CYP1302	A	2				
CYP1311	A	2		1		
CYP1312	A	1				
CYP1315	B				1	
CYP1318	A		1			
CYP1319	A		3			
CYP1326	B	2				
CYP1329	A				2	
	C				1	
CYP133	B		10	22		
	C		14			

	D			1		
	E		4			
	F	1				
	J		1			
CYP1330	A	1				
CYP1335	A				2	
CYP1337	A	1				
CYP134	B			2		
CYP1347	B				6	
	C				1	
	E				1	
CYP1349	A	1				
CYP1350	A	1				
CYP1352	B				1	
CYP1355	B		4			
CYP136	F			2		
CYP1366	A				1	
CYP1371	B	1				
CYP1373	B				2	
	C				2	
	D				1	
CYP1376	B	1				
	C	1				
CYP1384	A	1				
CYP1387	A				1	
	B				1	
CYP1396	A	1				
CYP1405	B	1				
CYP1406	B	2				

	C	1				
CYP1413	A			3		
	B			1		
CYP1414	A			2		
	B			1		
CYP1415	A			3		
CYP1448	B				1	
CYP1460	C	1				
CYP1464	A		4	2		
CYP1465	A			4		
CYP1466	A			1		
CYP1467	A			1		
CYP1468	A			1		
CYP1469	A			2		
CYP147	D	16				
	A				1	
	L				1	
CYP1470	A			3		
CYP1471	A			1		
CYP1472	A			1		
CYP1473	A			1		
CYP1474	A			3		
	B			2		
	C			2		
CYP1475	A			1		
CYP1476	A			1		
CYP1477	A			1		
CYP1478	A			1		
CYP1479	A			1		

CYP1480	A			1		
CYP1481	A		1	2		
CYP1482	A			1		
CYP1483	A			1		
CYP1484	A		1			
CYP1485	A		6			
CYP1486	A		3			
	B				2	
CYP1487	A		2			
CYP1488	A		36			
CYP1489	A				2	
CYP1490	A				1	
CYP1491	A				1	
CYP1492	A				3	
CYP1493	A				1	
CYP1494	A				1	
CYP1495	A				1	
CYP1496	A				1	
	B				1	
CYP1497	A				1	
CYP1498	A				1	
CYP1499	A				1	
CYP1500	A				1	
CYP1501	A				1	
CYP1502	A				1	
CYP1503	A				1	
CYP1504	A				1	
CYP1506	A				5	
CYP151	A		1	2		

	C	2				
CYP1515	A	2				
CYP152	G			3		
	R			2		
	W			2		
	X			1		
	Y			1		
	B	4	3			
	C	3				
	E	2	1		2	
	P	1				
	AA	1				
	AB	2				
	AR		3			
	S		2			
	Z				1	
CYP153	A	44	5	16		
	C	2				
	D	14				
	E			3		
CYP155	J	3				
	K	1				
	H				1	
CYP159	B			4		
	A				1	
CYP1591	A	1				
CYP1597	A	1				
CYP163	K			1		
CYP167	B				11	

	A				1	
	C				1	
	D				1	
	E				2	
CYP168	A			18		
	B			1		
	C			1		
CYP1686	A		1			
CYP169	A		1	13		
CYP172	B			1		
	A					**52**
CYP173	A	45				
	B	8				
	C	3				
	G	1				
	H	1				
	J	2				
CYP1732	A	3				
CYP1733	A	1				
	B	1				
CYP1734	A	1				
CYP1735	A	1				
CYP1736	A	1				
CYP1737	A	2				
CYP1738	A	1				
CYP1739	A	2				
CYP1740	A	1				
CYP1741	A	1				
CYP1742	A	1				

CYP1743	A	1				
CYP1744	A	1				
CYP1745	A	1				
CYP1746	A	1				
CYP1747	A	1				
CYP1748	A	1				
CYP1749	A	1				
	B	1				
CYP1750	A	1				
CYP1751	A	1				
CYP1752	A	4				
CYP1753	A	1				
CYP1754	A	1				
CYP1755	A	1				
CYP177	E			2		
CYP1779	A			1		
CYP180	D	1				
CYP183	AG		1			
	AH				2	
	BE				1	
CYP1833	B				1	
CYP186	K	7				
CYP191	B	1				
CYP192	A	3				
CYP193	A	17				
CYP194	A	10				
	C		2			
CYP195	A	18	29			
	D	1				

	E	2				
	C		1			
CYP196	A	34				
	B	2				
	C	1				
CYP1962	A		1			
CYP197	R	2				
	AA				2	
	AB				2	
	F				4	
	G				1	
	H				1	
	J				1	
	K				1	
CYP198	A			3		
CYP1982	B		3			
CYP199	A	11	2			
	B	1				
	J	1				
	K	1				
	L	1				
	M	1				
	H		1			
	V		2			
CYP200	A	5				
	B	2				
	C	1				
CYP201	A	25				
	B	3				

	C	3				
	E		1			
CYP202	A	46				
	B	24				
CYP203	A	12	6			
	B	1				
CYP204	A	2				
CYP206	A	9				
	B	1				
	C	3				
	D	6				
CYP209	A				1	
CYP210	A				1	
	B				1	
CYP212	A		34			
	B		6			
	E		1			
CYP2140	A	1				
CYP2159	A		1			
CYP219	A	2				
CYP220	A		2			
CYP221	A			2		
CYP223	A	2				
	E	1				
CYP224	A	1				
CYP2242	A			1		
CYP225	A	7	1			
CYP226	A		18	5		
	C	1				

	D	1				
CYP229	A			7		
	D		1	7		
	E			4		
	B		4			
	F		39			
	G		1			
	L		4			
	M		4			
	N		1			
	P		1			
	H				1	
CYP2308	A		1			
CYP2334	A	2				
CYP234	A			1		
CYP2353	A		1			
	B		2			
CYP236	A			3		
CYP238	A			1		
CYP240	A		15			
CYP242	A				4	
	B				1	
	C				1	
CYP251	V				2	
CYP2528	A				1	
CYP253	D				5	
	G				6	
	H				2	
	J				1	

	K				1	
	L				1	
	M				1	
	S				1	
CYP259	A				1	
CYP260	A				1	
	B				1	
CYP261	D		1	1		
	A				1	
CYP262	B				2	
	A				1	
	C				1	
	D				1	
	F				1	
CYP2624	B				1	
CYP263	A				3	
CYP264	A				6	
	B				1	
	C				1	
	D				14	
	E				2	
	F				1	
	G				1	
	H				1	
	J				3	
	K				3	
	L				1	
CYP265	A				1	
CYP266	A		1		1	

	B				1	
CYP267	C		1			
	A				4	
CYP2724	A					**1**
CYP2730	A		1			
CYP2733	B		1			
CYP2742	A		1			
CYP2743	A		1			
CYP278	A	1				
	C	1				
CYP286	C	1				
CYP288	B	2				
CYP289	A	33	14	6		
	D			1		
	E		1			
	F		1			
CYP290	A	5				
	B	1				
CYP51	B			2	2	
**292**	**564**	**873**	**603**	**277**	**333**	**53**

Table S3. P450 identified and annotated in this study is presented as per their class. A header follows each P450 sequence consisting of protein ID, database, P450 name, species name, species code, and an abbreviation for the class, such as BP for *Betaproteobacteria*, DP for *Deltaproteobacteria*, and EP for *Epsilonproteobacteria*.

Betaproteobacteria

>VITFI_CDS3214|KEGG|CYP1098B3|[Vitreoscilla filiformis]vff|BP

MSRFCPYYPRPVSDKTPAWRMFFSKRRSWLDGLYERSYHMKMGEVHFPGVDLYMVNDPAL
VREVMDNADRFPKHEMLGEALRPLLGDSIFTTNGEVWQRQRDLMDPSFEAARVKLVFGRM
RDAAQAMVERLRQVPEGAVYDLEVEMTHVAADIILRTILSKSLDSEGARLIFDAFARYQD
MAPRLMLPAFFGLRWLRPWWQIRRSRRAAEDIRKLIVQLIRPRYDAYQTALASGNTEGVE
TGGDILATLLRVRNAETGQGFTFDELVDQVAMLFLAGHETSASALSWSLHLLANAPDVQE
RLHQESLDKLGAVGTDPSGLKDLELSRNVFRESLRLFPPVGFLVRASETGCPLGDKTVKP
GGSVMISPWLIQRHRQFWARPDEFDPDRYTHTEDTTPMTTRESLRKAYLPFGMGPRVCIG
AAFALQEATLILSTLARHFRFEPVPGHRPEPVGRLTLRAENGIPLKVFARDR

>VITFI_CDS3233|KEGG|CYP203A11|[Vitreoscilla filiformis]vff|BP
MFSFDPYSPAVDADPFPYYQRLRDEFPCFWSPEAQMWVLSRYSDIVTALNDWQTYSSASGNLMTELPGRAGATLGSSDPPRHDRLRALVQHAFMKR
NLLALETPIREVARDVFGTLKGVSEFDFKEVSSKFTVKVLMAALGLPMGDEALVPEQEVRDNAVLMVQSDARTRTKGPEHIAAYNWMQDYAAKVIA
LRRAEPRDDLISHFAQAEIDGDKLDEREVLLTTTTLIMAGVESMGGFMMMFAHNLATFADARRAVVANPALLPDAIEESLRFNTSAQRFRRRLMKD
VSLHGQTMKQGDFVCLAYGSGNRDERQFPNPEVYDITRKPRGHLGFGGGVHACLGTAIARMAVKIAFEEFHRVVPDYRRVADQLAWMPSSTFRSPL
VLLLKAQ

>ADP71_22770|KEGG|CYP151A16|[Vitreoscilla sp.]vit|BP

MDNSLIVEVPFLNFADPSFSIRSDAVKQAQDVSWYAKTPYGLAILRYDEVNALVRDQRLR
QGSYAWPKHCNATGAFADWWTRMLLSKEGPNHSRLRRLANPAFSPKLVRLMQPVFFQLAN
DLINQFIDQGKCEFVGDFAAPYATQVICCLLGIPLSEAKRLAELAVDMGVALGVTYERDQ
NLVNGATDEMFEYCYEAVRRLKEEGLGDNFLSMLVKANENQADLSDEELFDMMVLAIFGG
IDTTRNQLSLAMDTFVQNPAQWQILGEQPDLAANAVEEIMRFRPTVTWVTREALEDFEFQ

GVFIKQGTTVHLISQAADNDPRAFGDVAFDITQKRKAHFGFGAGAHHCIGHFIARGDMKE
ALALLAQRLHNVQYAAEPSYMPDSGNTGLNHLHIRFDAVKG

>CV_2656|KEGG|CYP212A1|[Chromobacterium violaceum]cvi|BP
MKTPPQSSCPFHAVGRPPTPPRSSAGRWPPGPESGLTGWGLLKLMSRDLMGTLAGWQREFGDLVHVRTWPEHQVIVSDPQLARELLVNQADALQRW
ERALTVYRRVHGHSVLIAEGQVWREKRQALQPDFTRKSVQAFSPSIVEAARRAFEQWPARHAAWPIESELTSVTMEVILRMMFSSGVGSEAQQAEE
AVHTLMVASTEELWRPASLPDWVPWQRKRRRARLLMNGLIERHLQARLAMPQDAWPEDLLSRLLRLHLQQPQSWPLQAVRDECKTAFLAGHETVAT
SLTWWAWCMASHPEIQERAREEALAALSGGGQADPAALQYVSQTLLETMRLYPAVPLLMSRRALKPVTLGDWTFPAKTVFMVPMQLMQHDERWFPE
PRSYRPERFGPDAARPQQGAYLPFGGGPRVCLGQHLAMAEMALVAAQLLLRYRLSAPEGAEPPRPVFHVSQRPSQPLTLGIARI

>BKX93_03785|KEGG|CYP212A8|[Chromobacterium vaccinii]cvc|BP
MKTPPQSSCPFHAGGHPPASPKPSSGSWPPGPEAGLTGWGLLTQLSRDLMGTLAGWQREFGDLVHVRTWPEHQVIVSDPHLARELLVNQADSLRRW
ERALSVYGRVHGHSVLIAEGEAWREKRQALQPDFTRKSVQAFSPSIEAAARRAFEQWPDRRDAWPIESELTSVTMEVILRMMFSSGVGSEAREAEE
AVHTLMVASTEELWRPASLPDWVPWQRKRRRARLLMNGLIERHLQARLRLPADAWPEDLLSRLLRLHLQQPKSWPLQAVRDECKTAFLAGHETVAA
SLSWWAWCMASHPDIQEKARAEALAAWPGEESADADLSALQYVSQTLLETMRLHPAVPLLMSRRAVKPVTLGDWTFPAKTVFMVPMQLMQHDERWF
PEPLSYRPERFDPDAARPQQGAYLPFGGGPRVCLGQHLAVAEMTLVAAQLLRRYRLSAPAGMAPPRAVFHVSQRPSAPLALDIARI

>DK842_20225|KEGG|CYP212A20|[Chromobacterium phragmitis]chri|BP

MKTTSSSCPFRPGGQPASQPNPQAGAWPPGPKPGLMGWRLLSRMSRDLMGTLADWQREFG
DLVHVRTWPEHQIIVCHPQWARELLVSQADALQRWERALFVYGRVHGHSVLIAEGEAWHQ
KRQALQPDFTRKSVQAFSPSIIDAGKQAFAKWPERDDAWPIERELTSLTMEVILRMMFSS
GVGEEAQQAEDAVHTLMVASTDELWRPFSLPDWMPWQRKRRRARLVMGELIERHLQARLA
VPPDAWPEDLLSRLLRLHLEQPEEWPLQAVRDECKTAFLAGHETIAASLGWWSWSMASHP
DIQQRAREEAIAAWPGDEHGEMDLSALQFVSQTLLESMRLYPAVPLLMSRRAIKPVAIGD
WTFPARTVFMVPMQLMQHDERWFPEPRRYRPERFAPEAGRPQLGSYLPFGGGPRVCLGQH
LAMAEMTLVAAQLLRRFRLSIPAGAQPPRPVFHVSQRPDKPLTLAIARL

>DK842_05860|KEGG|CYP261D1|[Chromobacterium phragmitis]chri|BP

MFATRGPPRGYMDGLICGRTDRTREFRFFIVWKEVLTEKDAWSETEAVAHRLSTRSETKAIVLLDQMQRNNMKRFNDLPAPPSLPFLGNAHQLDKK
GLHLTLEKWAREYRQEPFRFQLGPLKFIVLSRPEDVRCVLKERPTQFRRMREMSKIFDEVGFSSVFTKEGEAWRRQRRMMQPAFTKHTLVPFVPQI
ARKALQLRDRLQKSNGEPLEIRHDFRRFTVDVGCLLVFGVDLNNLADDDNHVLHTFHNVVHTMNKRLRSLVPYWRLVKMPVDRRFDGSIQELRDMV
TGIAHDVRARMKRGEDLGEHCILHTMLAASEGDDATLTDDELFANMMILVLGSEGSTAAMLSWICFYLSQDEVLQQRLREELCDFDLETVDYDAIE
SLPLTEAVIEETFRIRSTVPALVLEALEDTEVGELHVDRGTRVFALTRVNGIMDTARDMVFDPRRWLNEDGSLASLKDARQTQFPFGYGPRVCPGA
PLSNIELKLAIAMLVKHFKLEIVDGEAVEEVFMASAVPDNFRVKAIPLPKRPRESGSLVFGREEEREDARSPA

>DK843_03200|KEGG|CYP212A20|[Chromobacterium phragmitis]chrb|BP
MKTTSSSCPFRPGGQPASQPNPQAGAWPPGPKPGLMGWRLLSRMSRDLMGTLADWQREFGDLVHVRTWPEHQIIVCHPQWARELLVSQADALQRWE
RALFVYGRVHGHSVLIAEGEAWHQKRQALQPDFTRKSVQAFSPSIIDAGKQAFAKWPERDDAWPIERELTSLTMEVILRMMFSSGVGEEAQQAEDA
VHTLMVASTDELWRPFSLPDWMPWQRKRRRARLVMGELIERHLQACLAVPPDAWPEDLLSRLLRLHLEQPEEWPLQAVRDECKTAFLAGHETIAAS
LGWWSWSMASHPDIQQRAREEAIAAWPGDEHGEMDLSALQFVSQTLLESMRLYPAVPLLMSRRAIKPVAIGDWTFPARTVFMVPMQLMQHDERWFP
EPRRYRPERFAPEAGRPQQGSYLPFGGGPRVCLGQHLAMAEMTLVAAQLLRRFRLSIPAGAQPPRPVFHVSQRPDKPLALAIARL

>D1345_00555|KEGG|CYP2353B2|[Chromobacterium rhizoryzae]crz|BP
MSAWPLDPLAAVVHAHPYPYYRDLAQRQPLYFDAGLGLWVAAGHAAVQAALASATCRVRPVEQPAPSQLGDGGLARTFCLWARMSEAPRHPALKAA
LNRALATVAGAEAGGLAESLARDWLAREPLNGGLLDRMAQGLPVLTLAGLLGLPMEDAPGLVAEAAGLARALAADADAEALESGERALEALQARVA
KALPRSGGLLAVLRREWMEEEEALTANVIGLLFQSLDAGAGLFAAAVWHQAQGRPLAAGAAADWAWLALHDPVLHNTRRFVARDWHHGGQTVPAGA
SLLVVLAAAALDPAGPGEGLMFGAGRHACPGQALALEIARGGLAALAAMRPDWKTLTAAARFRRLPNARVRIYDEEGSAE

>FYK34_06710|KEGG|CYP212A21|[Chromobacterium paludis]chrm|BP
MKTPHTSSCPLHADTKASTTANPGTSAWPPGPKAGLTGLRLVARMSRDLPGALAEWQSEFGDLVHVRTWPEHQVVVSEPRLVRELLVSQAGALVRW
QRARRIFSRIHGHSVLTAEGEAWRNKRQALQADFTRQAAQAFSPAILAATEQALSAWPEQDDRWPIERALTSLTMDVIMRMLFSNGIGEDARRTEQ
AAHALMVASNAEFYQPFSLPDWMPWQRGRRQARQVLERLIEDHLQTRLRLAREAWPEDLLSRLLRLHLQQPQAWPLQAVRDECKTTFLAGHETVAA
TLTWWAWCMASHPEAQAAARAEARQFAPEQAMGQAGAPWRYLVQTLQETLRLHPAAPILISRRALQPVTLGDWRLPAGTMYLVPVLLMHHDARWFP
QPRSFRPERFAPEAARPPLGAYLPFGSGPRVCLGQHLAMSEMTLIAAQLLRRYELSVPEGQAAPEPAFYITRRPREPLRLRIRRR

>FYK34_16325|KEGG|CYP2353A8|[Chromobacterium paludis]chrm|BP
MNAWPEHALAAASHRDPYPYYLELARQGWARDQRLGLWLAASHAAASAVLACPACRVRPLDEPAPAALAGLALGETFGCWLRMSEAPIHAPLRAAV
AASLAAQEDERAQRRARDLARGFLAGAAPDGARLDALVDFLPAATLASLLGWPERSLLGWPERSLLGLSGQVAAWCRAVAADAGAAEREAGEAACL

ALHAELAALADAGWLARWRQEFGELGEDALRANLLGVLFQSRDAGAGLLAAGISWRCEGPWDWRAAAEWRRRLRADPVLHHTRRFVAEDATLAGQP
LKAGDQVLVLLAAAARDPAGPGESMDFGRGRHGCPGERLALAIARGALAALEEIGPDWRALGGGLVFRGPPNVRMRRFGAGDGR

>CH06BL_01070|KEGG|CYP2353B5|[Chromobacterium haemolyticum] chae|BP

MSAWPLDPLAAVVHAHPYPYYRDLAQRRPLYFDAGLGLWVAAGHAAAQAVLASAACRVRP
LEQPAPPLLGDGGLARTFCLWARMSEAPSHPALKAALNRALATVAGAEAGRLAESLARDW
LAREPLSGGLLDRMAQGLPVLTLAGLLGLEVEDEPALVAEVAALAQALAADADAETLARG
EQALAALQVRVAEALPRSDGLLAELRREWTVEEAALTANVIGLLFQSLDAGAGLFAAAVW
HQAQGRPLAAGAAADWAWLALHDPVLHNTRRFVARDWHHGGQTVPAGASLLVVLAAAALD
PAGPGEGLMFGAGRHACPGQALALEIARGGLAALAAMRPDWKTLTAAARFRRLPNARVRI
YDEEGSAE

>C1H71_18930|KEGG|CYP107NX1|[Iodobacter fluviatilis]ifl|BP

MITTKPLSFYDPAFYQNPYASFQRLMEKGEIHYVKFPSGVCGWLVTGYDAAMKTLTHPNI
GKNHKLGNAHWRSLASIMPEPQHTTLQSHLLHQDPPRHTALRALIMEAFSASRIDRFRER
IALIAQRLLEPLQRAGECDLIADFASQLPLLVLSEVIGLSDKHRIQFKPVWCKVVQPVGP
NDVGRAAYIGLLTELQSYIDTVIVESRGGDSDRLIVQLIAAYDGKKISRDELTSVLFQLL
VAGQEPMTNQIGNAMLALLQHPEQLKQIREDSTRIDRAMTELLRYDGAFALSTWRFFTET
TQWLGATVFAGDSVIVGLNAANHDSAQFGCPHKLDFAREPNKPLSFGYDHHYCPASALAR
LELEVSIQAILEYLPNIQLACTDRELRWINAVLARGLLALPVRYTLGASRS

>NH8B_0727|KEGG|CYP289A13|[Pseudogulbenkiania sp.]pse|BP

MNTKQTPPRDAGAQRPSPDWDARAPAVLQDQIRAYDDMRHRCPVAYSEYLNWSLFRHDDV
MQALTDPKTFSNAVSSYPSIPNGMDPPEHTAYRMLIDPYFAPEAMARFEPVCRRIAVDLV
RELPSAGEVELMGQFAEDFALQIQCAFMGWPVALHDPLRRWTHRNRAATLAADRAAMAAV
ALEFDGYIRAQLDARRAAGDQAPDDVTRRLLGETLQGRTLSDAEIVCIVRNWTVGELSTI

SSCVGILTQYLAERAELQQRLRQQPELLPAAIDEILRIHAPLIANRRIATRDVEIGGCQL
KAGDRVTLIWASANRDETVFGDADEFRLDRDPADNLLYGAGIHVCPGAPLARLELKIVME
ALLSQTERIGTVAGKTPVRASYPASGYASLPLWIERAAGPAPQA

>DLM_1347|KEGG|CYP1200A5|[Aquitalea magnusonii]amah|BP
MNAPVSALAPVWVGDIPQLDAAAVYHATSPANGMARIILPSGHVAYHLTRYHEVLQVLTDARFVRRPCNTEDGPSFLPTITPNELLLNNDGASHAR
LRKVVAKDFSAAGVATLRDTVIQATHARLDALSSQTGVIDLLDLVLECIPAEVDCHLLGIPLADRSYYRPLTHTVQIADPRDVPELLRQFWLVYDY
LMDLVTGRRTTAPHGLIRRFVAARHESVPPLNDEELVGILLGILIGGDQNILTVLCKVVYTLLAAPALWQSLRAQPATLPAMVEELLRLIPLGTTS
TFPRIASEGVHGSFGFIPAGSVVYADVFAANRDPAVFADPLRIDPQRNASKHLQFGYGMHHCMGAALARMEITTVLEILLQRMPDLQLAQPAASVP
WTHGTILRRPASLPVCGLTLR

>RCFBP_20388|KEGG|CYP1179B1|[Ralstonia solanacearum]rsc|BP

MSEIENMETLNLSSSDIEPHRYRLYEAHRQNAPALWYPSANSWLIFRHGDAASLARSPLV
RTDYLVREKISDAMLEADPALREIVATISRWMIYNEAPIHGRLRTFMSRTFDRGYIERVR
GRIASILAPRIEALKGRTEFDFVEALAHPVPAEILAAMLGLDEIDIVDFLRWSDAIADFM
QDFVISPVPNRQIARATAEQLLEMKAALREAIAVRRRQPKEDLLTDLACATGDDGSTITE
EELMLQLIHLIFGGHKIPQFVLANTLHLLFKNPQYMLHGEEATFDKVSKLVDESMRVESP
IQFITRHAVDDFELNGQRIKKGDSIYLMLGSANRDPEVYSDPDTFQPYAPKRKGIHYGTG
PHVCIGAALAGVTIAEILCSFLQATRSVEALYDLDHPEWTRNDTFHGVARMPVKAEYR

>RPSI07_mp0406|KEGG|CYP1464A2|[Ralstonia solanacearum]rsl|BP
MGDMQDTAAVAERDTVAGQQRDRATVDLLEVARDGKPASVARPVAGNLAMKDGFPVMPGK
VPLLGHVHRISQDALGELRKAEAACGPMFWTYFGARLPVLQILDEEGLAILQNKYTDNSF
LREQMPVITGAAMNAFDGPRHRNARHASSAAFSPTGLTRAQVGQFVVESIEQRLKRWTRE
SKLAIFPETKDIALEVVFRILGIKTHELAQWRRQYEEFFLGMIPLKINLPGFPAWRCRKA
RGWLEARVAQIVATARADDDHDSLVGAMIFGRDDNGNGMSEVELVHNILGLGFAGSETTA
SVMAWSALMLAQHPDVWRQLCRQVAGMDSVPSTQEELVRQAPLAEAIFRETMRLYPPAPF
EMRKVHTEFEMMGHKIPAGVMVGVSLLHVSRNPERYPDPDSWKPERWLGVDRALTPVETC
GFGGGPHACLGRHVAALEITLFITMLARELGPKGIVPRLAGKLPAPAYLPFLRPSNKAFL

DMTGR

>RPSI07_mp0607|KEGG|CYP133B5|[Ralstonia solanacearum]rsl|BP

MKLADLKLADLSTPSFLENPYPLYETLREQGPFVRIGPNGLMTGRYSIVDALLHNRQMGK
NYMDSIRLRYGDKGPDMPLFQGFSRMFLLINPPAHTRLRSLMMQAFNARQIESMRELATA
TAHQLIDGFEQKQSADLVAEFAFPLPVRIICRMLDVDIDDAPALGVAASKLAKVFDPAPM
SADELVKTSAAYEELAQYFTKVIEARRAQPGTDLISMLLRVEEDGQKLTHDEIVSNVILL
FIAGHETTSNMIGNALIALHRNPRQLDLLKRDPSRMPNAVLECLRYDGSVQVTIRAAMED
VEVEGEVVPRGTTVFLMLGAANRDPAQFTDPDQLDIGRQQGRPQTFGAGIHHCLGYRLAL
IELESALNALFERLPHLRLTNLDQLSWNQRGNLRGVNALMAAW

>RPSI07_mp1116|KEGG|CYP229F11|[Ralstonia solanacearum]rsl|BP

MDIALDRPADPLQAVTHTDPAPYYTGLAAARPFHYDAALGWWIAAGAEAVGAVLGSLACT
VRPADEPVPRHIASSPTGLFFSRLVRQTDGPGHAECKATVMAALGRLDLSAVAARAHRLA
YMALPDATALGPALADAMHLRIPLATMTHALLGDDADAAACQRDIATYLAALSPGAPPAA
IHHAQTAVARLSERVAAAPLAAHADETIVANLAGLLAQTHDACAGLLGNAIVTLARQPGL
AAHLRSHPHAIDPFLYEVLRLDAPVQNTRRFVSRDTTLCGESLRAGDRILVLLAAANVDT
AANPDPLRFDLARVRRRVWTFGAGAHRCPAESLAVAVARETVQHLLSLPGIARWLAGAGE
VRYQASPNARIPVFSPSHQEAP

>RSPO_c02265|KEGG|CYP1179B1|[Ralstonia solanacearum]rsn|BP
MSEIENMETLNLSSSEIEPHRYRLYEAHRQNAPALWYPAANSWLIFRHGDAASLARSPLVRTDYLVREKISDAMLEADPALREIVATISRWMIYNE
APIHGRLRTFMSRTFDRGYIERVRGRIASILAPRIEALKGRTEFDFVEALAHPVPAEILAAMLGLDEIDIVDFLRWSDAIADFMQDFVISPVPNRQ
IAQATAEQLLEMKAALREAIAVRRRLPKEDLLTDLACATGDDGSTITEEELMLQLIHLIFGGHKIPQFVLANTLHLLFKNPQYMLHGEEATFDKVS
KLVDESMRVESPIQFITRHAVDDFELNGQTIKKGDSIYLMLGSANRDPEVYSDPDTFQPYAPKRKGIHYGTGPHVCIGAALAGVTIAEILCSFLQA
TRSVEALYDLDHPEWTRNDTFHGVARMPVKAEYR

>RSPO_m00920|KEGG|CYP229F10|[Ralstonia solanacearum]rsn|BP
MARDRGGGAFENEPSYALRTAMDIALDLPADPLQAVTHAGPAHYYTALAAARPFHYDAALGWWIAASAAAVDAVLGHTACTVRPADEPVPRHLAAS
PAGVFFGRLVRQIDGPDHGARRAAVLTALGRLDVSSVSAHAHRLAKMALPDATALGPALADAMHLRIPLATLAHALLGDDADAAACRQDIAAYLAA
LSPEAPAAAIHRADTAVARLSERFATAPLAAHADEALVANLAGLLAQTHDACAGLLGNAVVALARRPGLAGHLRTHPHAIDPFLYEVLRLDAPVPN
TRRFVSRDTTLCGESLRAGDRILVLLAAANVDASANPDPLRFDLARAPRRVWTFGAGAHRCPGEALAVAIARETVRHLLNLPGIDRWLAGVAMVRY
QASPNARIPVSAPSHQEAP

>RSPO_m01039|KEGG|CYP133B5|[Ralstonia solanacearum]rsn|BP
MKLADLSTPSFLENPYPLYETLRAQGPFVRIGPNALMTGRYSIVDALLHNRQMGKNYMDSIRLRYGEEGPKMPLFQGFSRMFLLINPPAHTRLRSM
MMQAFNARQIESMRELATVTAHQLIDAFEQKRSADLVAEFAFPLPVRIICQMLDIDVDDAMVLGAAASKLAKVFDPAPMSAGELVETSAAYEELAQ
YFTKVIEARRAQSGTDLISMLLRVEEDGHKLTHDEIVSNVILLFMAGHETTSNMIGNALIALHRNPQQLDLLRRDPSRMPNAVLECLRYDGSVQVT
IRAAMEDVEVEGEVVPRGTTVFLMLGAANRDPDQFTEPDRLDIGRQDGRLQTFGAGIHHCLGYRLALIELETALSALFTRLPDLRLTNLDQLGWNQ
RGNLRGVNALMAAW

>RSPO_m01214|KEGG|CYP1464A2|[Ralstonia solanacearum]rsn|BP
MTANMMSDQQETATVAQSDTAQRQSRATPPEVARAGEPARVARPVADNLTMKDGFPIMPG
KAPLLGHVHKISQDALGELRRAEAACGPMFWTYFGTKLPVLQIMDEAGLAILQNKYTDNS
FLREQMPVITGEAMNAFDGPRHRNARHASSAAFSPTGLTRAQVGQFVVDTIEQRLKRWTR
ESKLAIFPETKEIALEVVFRILGIETHELAQWRHQYEEFFLGMIPLKIDLPGFPAWRCRK
ARRWLEARVARIVATARANGDHDSLVGAMIFGRDDNGNGMSEVELVHNILGLGFAGSETT
ASVMAWSALMLSQHPEIWRQLCQQVAGLHSMPLTHEELVSQVPLAEAIFRETMRLYPPAP
FEMRKVHTEFDMMGHRVPAGAMVGVSLLHVSRNPERYPDPDNWRPERWLGIDRALTPVET
CQFGGGPHACLGRHVAALEITLFIAMLARELGPKGITPRLAGKLPPPAYLPFLRPSNKAF
LDLTGR

>CMR15_20043|KEGG|CYP212A14|[Ralstonia solanacearum]rsm|BP
MSRDLLGSLAAWQREFGDVVHLHFWPEHEIVVTDPALARELLVSHHDALVRWERGIRVFAQVHGHSVLIAEGEAWRGKRHALQPSFSPKAVQAFVP
TIAAATSRALLQWPRQDVRWPIENAITSLTMDVITRMMFSSEIGEEARAAEKAIRVVSAAADAEFYWPASAPDWMPWKRAKRRALAELHGLIDRHV
QTRLAQPHEVWPDDLLTRLLQLHQEEASAWPLHAVRDECMTAFLAGHETTAAALTWWAWCMACNPAAQTTARKEVQAVLQGRTPDADMLASLPYLT
QTIKETLRLYPAAPVLISRRATRSIALGPWQFPARTMFLVPVQLMHHDPRWFPQPLSFRPERFAQDAPEIPRGAYAPFGAGPRVCLGQHLAMSEMT
VIAAMVLQRFSLSVPDGMQAPRPVMRVTLRPDQPMHLAIAPI

>CMR15_mp10680|KEGG|CYP133B5|[Ralstonia solanacearum]rsm|BP

MKLADLSTPSFLANPYPLYETLRAQGPFVRIGPNALMTGHYSIVDALLHNRQMGKSYMDS
IRLRYGEDGPNMPLFQGFSRMFLMLNPPMHTRLRGLMMQVFNARQIEAMREVATATAHQL
IDAFAQKPSADLVAEFAFPLPVRIICQMMDLDIDDAMALGVGVSKLAKVFDPAPMSADEL
VETSAAYEELAQYFTKVIEARRAQPGTDLISMLMRAEENGETLTHDEIVSNVILLFIAGH
ETTSNMIGNSLIALHRNPQQLDLLKRDPSRMPNAVMECLRYDGSVQVTIRAAMEDVEVEG
EVLPRGTTVFLMLGAANRDPAQFTDPDQLDIGRQQGRLQTFGAGIHHCLGYRLALIELEA
ALGALFERLPNLRLTNLDQLSWNQRGNLRGVNTLMAAW

>CMR15_mp10892|KEGG|CYP1179B1|[Ralstonia solanacearum]rsm|BP

METLNLSSADIEPVRYKLYESRRQNTPALWYPEANSWLVFRHQDVASLARSPMVRTDYLV
REKISDAMLDADPSLREIVATISRWMVYNEAPIHGRLRTFMNRTFDRGYIERVRDRIASI
LAPRIEALKGREEFDFVETLAHPVPAEILAAMLGLDEIEIVDFLRWSDAIADFMQDFVIS
PVPNRVIAQATAKQLLEMKAALREAIAVRRRQPKEDLLTDLACAAGDGGDAITEEELMLQ
LIHLIFGGHKIPQFVLANTLHLLFKNPQYLVHGEEATFDKISKIVDESMRVESPIQFITR
HAVDDFMLHGERIKKGQSIYLMLGSANRDAEVFGDPDTFQPDAPKRKGIHYGTGPHVCIG
AALAGVTIANILCQFLQATRSVEPLYDLNNPEWTRNDTFHGVARMPIKVTYR

>F504_4509|KEGG|CYP133B5|[Ralstonia solanacearum]rse|BP

MKLADLSTPSFLENPYPLYETLRSQGPFVRIGPNALMTGHYSIVDALLHNRQMGKSYMES
IRLRYGDEGPNMPLFQGFSRMFLMLNPPMHTRLRGLMMQVFNARQIESMREVATATAHQL
IDDFEQKPSADLVAEFAFPLPVRIICQMMDLDIDDAMALGVGVSKLAKVFDPAPMSADAL
VETSAAYEELAQYFTKVIEARRAQPGTDLISMLMRAEENGETLTHDEIVSNVILLFIAGH
ETTSNMIGNALIALHRNPQQLDLLKREPSRMPNAVLECLRYDGSVQVTIRAALEDVEVEG
EVLPRGTTVFLMLGAANRDPAQFTDPDQLDIGRQQGRLQTFGAGIHHCLGYRLALIELES
ALGALFERLPNLRLTNLDQLSWNQRGNLRGVNALMAAW

>RSUY_15200|KEGG|CYP1179B1|[Ralstonia solanacearum]rsy|BP

MSEIENMETLNLSSSEIEPHRYRLYEAHRQNAPALWYPAANSWLIFRHSDAASLARSPLV
RTDYLVREKISDAMLEADPALREIVATISRWMIYNEAPIHGRLRTFMSRTFDRGYIERVR
GRIASILAPRIEALKGRTEFDFVEALAHPVPAEILAAMLGLDEIDIVDFLRWSDAIADFM
QDFVISPVPNRQIARATAAQLLEMKAALREAIAVRRRQPKEDLLTDLACATGDDGSTITE
EELMLQLIHLIFGGHKIPQFVLANTLHLLFKNPQYMLHGEEATFDKVSKLVDESMRVESP
IQFITRHAVDDFELNGQTIKKGDSIYLMLGSANRDPEVYSDPDTFQPYAPKRKGIHYGTG
PHVCIGAALAGVTIAEILCSFLQATRSVEALYDLDHPEWTRNDTFHGVARMPVKAEYR

>RSUY_42850|KEGG|CYP229F10|[Ralstonia solanacearum]rsy|BP
MDIALDLPADPLQAITHADPAPYYAALAAARPFHYDAALGWWIAASAAAVDAVLGHTACT
VRPADEPVPRHLAASPTGVFFGRLVRQIDGPDHSARRATILTALGRLDVSSVAAQAHRLA
KMALPDATALDPALADAMHLRIPLETLAHALLGDDADAAACRQDIAAYLAALSPDAPAGA
IHRADTAVACLSARFATAPLAAHADEALVANLAGLLVQTHDACAGLLGNAVVALARRPGL
AGHLHTRPHAIDPFLYEVLRLDAPVQNTRRFVSRDTALCGESLRAGDRILVLLAAANVDA
SANPDPLRFDLARAPRRVWTFGAGAHRCPGESLAVAIARETVQHLLNLPGIDRWLAGVAM
VRYQASPNARIPVFAPSHQEAP

>Rpic_4258|KEGG|CYP102E4|[Ralstonia pickettii]rpi|BP

MRSASELPANASPAAAEPIPQPKGLPWLGNLLQLPKDRLAQTLLETSRQFPQGLYQLDFA
GRRVPFVYSADLVAELCDETRFRKLIGPPLSFLRAGAGDGLFTAHQDEPNWGKAHRILLP
AFSQRAMKGYFDVMLDVANALADKWARQGPDADIPVADDMTRLTLDTISLAGFGYRFDSF
NTPELHPFLAAMVGVLSEAMGKLTRLPLKDRFMREHHRRFEHDVAAMHQLVDEVIRARRQ
AKDGGIGASDLLGLMLNARDPLSDQPLDDTNIRFQVITFLIAGHETTSGLLTFALYMLLR
HPAVLAQAYAEVDRVLPGDTVPQYAHLAQLDVIERVLKETLRLWPTAPSFGVAPYEDTRI
GGRYAIRKDQRVVTVLLALHRDPAVWDRPEAFDIDRFLPENEAKLHPHAYKPFGNGERAC
IGRQFALTEAKLALAVILQRFALSDPYDYGFHIKETLTLKPDGFRLRARLRHARERLSVA
TPSRTEAVTQDDAEGALAGGGEPMHVLYGGSLGTCQDIAEQLAATASRAGFDAKVAPLDT

IADALPQRGTLVVVAATYNGRAPDSARTLEARLDAADALTRQATGLRYAVLGCGNSQWPA
FQAFPKRVEAMLAAAGAHAIVPRGEADGNAGFDAAVDAWTRSLWSALGARQTHADGPSVR
VDYVAPDALRAAMLPPAARAMTVLANEELVSDATGLWDFTREAPRGPTRHLTVQLPDDVT
YATGDHLAVYPRNADDRVDAAIARLALEADALVTLTARHAHVRHLPLNQPVSVRQLLRDF
VELQDTATVRDITALHAATRCPFTRGQLAVWLEGDDAAERFDQDIQAPHVSVLDLLIRLP
AIELTLEAFLARLGPMRPRFYSIASSARVSPGVAALTVGTVSGPAWSGVGTYRGTASNYL
MTLRAGAEIAAAVRTPNPPFAPDVDAHKPMVLIGAGTGIAPFRGFLEERAAQQAAGEAVA
TSLLCFGCRHPEHDFLYRDTLRAWEDAGLVRVFPAYSCVAEHPHRFVQHALWDAREAVWA
AFDAGATLYVCGDGRAMAPAVRDTLIRMHQARYGSDLATASGWLTAQMQSGRYRQDVFN

>Rpic_4262|KEGG|CYP229F16|[Ralstonia pickettii]rpi|BP
MTALDTAPPADVLQAIVHADPAPYYAELAATRPFFFDATLGWWVAASAEAVDAVLGSRAC
RVRPADEPVPKAVAATPTGAFFGRLVRQIDGPDHSTRKAKVMAALGRLDVSALAGQALRH
ARTALPDAASLDAALDADAHFRVPLATMAQTLLGDHADAPACQADIAAYLAALGPGASAG
VVAQADAAVGRLQQRFADSPFAGGDDDSDADIANLAALLAQTYDACAGLLGNCIVALSRH
AGLTARLRAQPLAIEPFVREVLRRDAPVQNTRRFVAHDIALLGQPLRAGDKVLVVLAAAN
VDGTTNPDPLRFDSDRTAPRLWTFGGGTHRCPADALATAIARETVRHLLSLPDVDRWLAG
LGEVAYRPSPNARIPRFQA

>Rpic12D_4368|KEGG|CYP102E4|[Ralstonia pickettii]rpf|BP
MRSASELPANASPAAAEPIPQPKGLPWLGNLLQLPKDRLAQTLLETSRQFPQGLYQLDFAGRRVPFVYSADLVAELCDETRFRKLIGPPLSFLRAG
AGDGLFTAHQDEPNWGKAHRILLPAFSQRAMKGYFDVMLDVANALADKWARQGPDADIPVADDMTRLTLDTISLAGFGYRFDSFNTPELHPFLAAM
VGVLSEAMGKLTRLPLKDRFMREHHRRFEHDVAAMHQLVDEVIRARRQAKDGGIGASDLLGLMLNARDPLSDQPLDDTNIRFQVITFLIAGHETTS
GLLTFALYMLLRHPAVLAQAYAEVDRVLPGDTVPQYAHLAQLDVIERVLKETLRLWPTAPSFGVAPYEDTRIGGRYAIRKDQRVVTVLLALHRDPA
VWDRPEAFDIDRFLPENEAKLHPHAYKPFGNGERACIGRQFALTEAKLALAVILQRFALSDPYDYGFHIKETLTLKPDGFRLRARLRHARERLSVA
TPSRTEAVTQDDAEGALAGGGEPMHVLYGGSLGTCQDIAEQLAATASRAGFDAKVAPLDTIADALPQRGTLVVVAATYNGRAPDSARTLEARLDAA
DALTRQATGLRYAVLGCGNSQWPAFQAFPKRVEAMLAAAGAHAIVPRGEADGNAGFDAAVDAWTRSLWSALGARQTHADGPSVRVDYVAPDALRAA
MLPPAARAMTVLANEELVSDATGLWDFTREAPRGPTRHLTVQLPDDVTYATGDHLAVYPRNADDRVDAAIARLALEADALVTLTARHAHVRHLPLN
QPVSVRQLLRDFVELQDTATVRDITALHAATRCPFTRGQLAVWLEGDDAAERFDQDIQAPHVSVLDLLIRLPAIELTLEAFLARLGPMRPRFYSIA
SSARVSPGVAALTVGTVSGPAWSGVGTYRGTASNYLMTLRAGAEIAAAVRTPNPPFAPDVDAHKPMVLIGAGTGIAPFRGFLEERAAQQAAGEAVA
TSLLCFGCRHPEHDFLYRDTLRAWEDAGLVRVFPAYSCVAEHPHRFVQHALWDAREAVWAAFDAGATLYVCGDGRAMAPAVRDTLIRMHQARYGSD
LATASGWLTAQMQSGRYRQDVFN

>Rpic12D_4372|KEGG|CYP229F16|[Ralstonia pickettii]rpf|BP
MTALDTAPPADVLQAIVHADPAPYYAELAATRPFFFDATLGWWVAASAEAVDAVLGSRACRVRPADEPVPKAVAATPTGAFFGRLVRQIDGPDHST
RKAKVMAALGRLDVSALAGQALRHARTALPDAASLDAALDADAHFRVPLATMAQTLLGDHADAPACQADIAAYLAALGPGASAGVVAQADAAVGRL
QQRFADSPFAGGDDDSDADIANLAALLAQTYDACAGLLGNCIVALSRHAGLTARLRAQPLAIEPFVREVLRRDAPVQNTRRFVAHDIALLGQPLRA
GDKVLVVLAAANVDGTTNPDPLRFDSDRTAPRLWTFGGGTHRCPADALATAIARETVRHLLSLPDVDRWLAGLGEVAYRPSPNARIPRFQA

>N234_33520|KEGG|CYP229F9|[Ralstonia pickettii]rpj|BP
MLTATLPDTGIDPLSAVIHPDPYPYYRELAARRPFFRDERLGLWVAAGPREVAAVLAHPDCRVRPPAQPVPPALAGTAAGDLFGRLIRMNDGAAHA
PLKQILMPMLAGIDPAAAALRAAAMATVIDAGAATGTPGERVNRWLFTLPVVTVADLLGLPVAEGDGATAVAQRVAAFAAAQSPLADAHTVRAGAE
AAQWLGAWLADTTPGDGPLPALHQAAHAAGIDAQAVAANTIGLLVQACEATAALAGNTLLRLGRGASDLALQAVVARVAQDDPPVQNTRRFLAADA
QLCGHAVKAGDAVLVLLAAASCSGLAGGARPWTFGHGRHACPGERLAQALAAATVAALLERGADPAALAQAFRYRPSLNARMPHFL

>N234_37805|KEGG|CYP108S1|[Ralstonia pickettii]rpj|BP
MNHTDFDLPGDVAQVLVDPSAYADPARLHAAYTWARENNPLGRAVNDGFDPFWVVTKHADVTAISRDHNLFHNGDYSVVCRPKASIDHVIATTGRP
NIVKALVHVDGDEHKGLRALTQSWFMPNSILKLDERIRGLARQIVDKLQQSDGNTIDFAKDIALHYPLHVIMDILGVPPEDEPRMLLLTQELFGGE
DPELKRAKQEASANDGALAKNLMAVVADFRDYFENVTAERRVNPRNDVATLLANAVVNGEPISEVSRLGYYVIIATAGHDTTSSSSAVAMWALSQY
PELLPRLQADSSLIPQFIDEAVRIASPVRHFMRTATADTEIRGRTIRKGDWLMLCYGSANRDEEVFDRPFDFSIDRKPNRHLAFGFGAHVCLGQHL
AKMEMRILFEEMIPRLRRVEAAGEMEMIASCFVGGPKHLPVRCVMQ

>TK49_21945|KEGG|CYP102E4|[Ralstonia mannitolilytica]rmn|BP
MRSASELPANVSAMVDPKAEPIPQPKGLPWLGNLLQLPKDRLAQTLLETSRHFPQGLYQLDFAGRRVPFVYAADLVAELSDETRFRKLIGPPLSFL
RAGAGDGLFTAHHDEPNWGKAHRILLPAFSQRAMKGYFDVMLEVANALADKWARQGPEADIAVADDMTRLTLDTISLAGFGYRFDSFNTPELHPFL
AAMVGVLSEAMGKLTRLPLKDRFMREHHRRFAQDVAAMHALVDEVIRARRHAPDGAAAASDLLGLMLNARDPVTDEPLDDTNIRFQVITFLIAGHE
TTSGLLTFALYMLLRNPAVLAQAYAEVDRVLPGDTVPQYAHLARLDVLERVLKETLRLWPTAPSFAVAPYEDTRIGGRYVIRKDQRVVTLLLGLHR
DPAVWARPEVFDIDRFLPENEARLHPHAYKPFGNGERACIGRQFALTEAKLALAVILQRFALSDPYDYGFRIKETLTLKPDGFRLRARRRNAHERL
SVAMPARVDAAKQPDAEAALAGGGEPMHVLYGGSLGTCQDIAEQLAATASRAGFDAKVAPLDTIADALPQHGTLIVVAATYNGRAPDSARTLEARL
DASGARVPQASALRYAVLGCGNSQWPAFQAFPKRVEAMLAASGAQAIVPRGEADGNAGFDAAVDAWIRGLWSALGAREAHAEGASVSVRYVAPDAV
RAATLPPTARPVTVLGNDELVSDPTGLWDFTQEAPRGPTRHITVRLPDGETYATGDHLAVYPRNADDRVDAAIARLGLEADALVTLTARHAHVRHL
PLEQPVSVRQLLRDFVELQDTATVRDIAALHAATRCPVTRGQLAVWLEGDEAARRFDQDVQAAHVSVLDLLIRFPAIELSLEAFLARLGAMRPRFY
SIASSARVSPQVVSLTVGMVSGPAWSGAGMYRGTASNYLMQLRPGAEIAAAVRTPNPPFAPEADASTPMVLIGAGTGIAPFRGFLEERAARQAAGH
AVATSLLCFGCRHPEHDFLYRDTLRAWQDAGVVRVFPAYSCVPGHPHRFVQHALWDARDAVWAAFDAGATLYVCGDGRAMAPAVRDTLIRMHQARH
GSDLATASDWLAAQMQAGRYRQDVFN

>TK49_21965|KEGG|CYP229F17|[Ralstonia mannitolilytica]rmn|BP
MDIAIAPPIDALQAVTHADPAPYYAQLAATRAFFFDAALGWWVAASAKAVDTVLGSDACR
VRPADEPVPNAVAASPAGAFFGRLVRQIDGPEHGMRKAIVMAVLGKLDTSALAARARRQA
YAALPDTAQLDAALAGDAHFRVPLATMAQTLLGDAADAPACHADIAAYLAALGPAPDAAS
VAQADAAVRRLHGRFEGSPLVAGNDDAEVDNLAALLAQTYDACAGLLGNCIVALSRHAAL
RTRLRAQPGAVEPFVREVLRHDAPVQNTRRFVQRDVQLLGQHVQAGQRILVLLAAANVDA
AVNPAPLRFDIDRPAPRLWTFGAGVHRCPAETLATIIARETVRHLLGLPGIDRWLGNLGE
VTYRASPNARIPVFSA

>ACS15_2481|KEGG|CYP212A11|[Ralstonia insidiosa]rin|BP

MSRDLLGTLAAWQREFGDVVHLRFWPEHEIVVSDPSLVRELLVTHHDALVRWERGMHVFA
QLHGHSVLIAEGEAWRGKRHAMQPNFSPKAVQAFVPTISAATEKALAQWPKHDVRWPIES
AITSLAMDVITRMMFSSEIGEEARIAEQAIRVVSAAANAEFYWPASAPDWVPWKRAKRRA
LAALHGLIDHHLQARLAQPQDAWPDDLLTRLLRLHRDAPTAWPLHAVRDECMTAFLAGHE
TTAATLIWWAWCMASNPAAQALARREVQQTLQGHAPSPDTLASLPYLTQTIKETLRLYPA
APVLVSRRSTRPITLGTWQLPARTMFLIPVQVMHHDPRWFPEPLAFRPERFALDALELPR
ARGAYLPFGTGPRVCLGQHLAMSEMTVIAAMLLQRFTLSVPDNMQPPRPVMNVTLRPDQQ
LHLAITPA
>ACS15_4201|KEGG|CYP102E4|[Ralstonia insidiosa]rin|BP
MRSASELPSNASSAAAEPIPQPKGLPWLGNLLQLPKDRVAQTFLEISRQFPQGLYQLDFVGRCVPFVYSADLVAELCDETRFRKLIGPPLSFLRAG
AGDGLFTAHQDEPNWGKAHRILLPAFSQRAMKGYFDVMLEVASALADKWTRQGPDADISVADDMTRLTLDTISLAGFGYRFDSFKTPALHPFLEAM
AGVLSEAMGKLTRLPFKDRFMHEHHRRFERDVAAMHQLVDEVIRKRRHAQDGGTGASDLLGLMLNARDPVTDLPLDDTNIRFQVITFLIAGHETTS
GLLTFALYMLLRHPAVLAQAYAEVDRILPGDTVPQYAHLAQLDVIERVLKETLRLWPTAPSFGVAPYEDTRIGGRYAIRKDQRVVTLLMALHRDPA
VWDRPEVFDIDRFLPENEARIHPHAYKPFGNGERACIGRQFALTEAKLALAVILQRFALSDPHDYAFHIKETLTLKPDGFRLRARLRNARERLLVS
VPSQADLTVDADTQTTLAGGGEPMHVLYGGSLGTCQDIAEQIAATASRAGFAAKVAPLDAIADALPQQGTLVVVAATYNGRAPDSARTLEGRLDSA
DALVRLAPGLRYAVLGCGNSQWPAFQAFPKRVDAMLAAAGAQAVVPRGEADGNGGFDTAVDAWTRSLWAALGARQVQADAAAVSVQYLAPDAVRAT
VLPAAARSMTVLANEELVCDPTGLWDFTREAPRGPTRHLTVRLPQGVTYATGDHLAVYPRNADDRVEAAIARLGLEGEALVTLTAQHAHVRHLPLN
QPVTVRQLLRDFVELQDTATARDITALHAITRCPFTRSQLAVWIDGEAAAQRFDQDIQAAHVSVLDLLIRFPAIELTLEGFLARLGAMRPRFYSIA
SSARVSPDVAALTVGTVSGPAWSGVGTYRGTASNYLTQLPAGAEIAATVRTPNPPFAPDVDASKPMVLICAGTGIAPFRGFLEERAAQQAAGEAVA
TSLLCFGCRHPEHDFLYRDALRAWEDAGVVQVLPAYSCVDSHPHRFVQHALWDPREAVWAAFDAGATLYVCGDGRAMAPAVRDTLIRMHQARYGSD
LATASDWLTEQMQSGRYRQDVFN

>ACS15_4205|KEGG|CYP229F6|[Ralstonia insidiosa]rin|BP

MNITSAPPADPLQAIVHADPTPYYAELAATRPFFFDAALGWWVAASADAVEAVLCSSACR
VRPAEEPVPKAVAASPTGTFFGRLVRQIDGPDHAARKATVMAALGRLDVAALANRAHQQA
HAALPDATALDAVLSTDAHFRVPLATIAQTLLADQADTHACQNDITAYLAALGPHADASV
VERADAAVGRLQQRFADSPFATSDDDADISNLAALLAQTYDACAGLLGNSIVTLARHPEL
VERLHAAPEAIEPFVREVLRLDAPVQNTRRFVAQDTELLGQPMRAGDRILVLLAAANVDA
AANPNPLRFDVDRAQPRLWTFGGGAHRCPAETLAVVIARETVQHLLRLPGIARWLANLGE
ITYRPSPNARIPVFQA

>CDC45_21165|KEGG|CYP133B5|[Ralstonia pseudosolanacearum]rpu|BP
MKLADLSTPSFLENPYPLYETLRSQGPFVRIGPNALMTGHYSIVDALLHNRQMGKSYMESIRLRYGDEGPNMPLFQGFSRMFLMLNPPMHTRLRGL
MMQVFNARQIESMREVATATAHQLIDDFEQKPSADLVAEFAFPLPVRIICQMMDLDIDDAMALGVGVSKLAKVFDPAPMSADALVETSAAYEELAQ
YFTKVIEARRAQPGTDLISMLMRAEENGETLTHDEIVSNVILLFIAGHETTSNMIGNALIALHRNPQQLDLLKREPSRMPNAVLECLRYDGSVQVT
IRAALEDVEVEGEVLPRGTTVFLMLGAANRDPAQFTDPDQLDIGRQQGRLQTFGAGIHHCLGYRLALIELESALGALFERLPNLRLTNLDQLSWNQ
RGNLRGVNALMAAW

>JK151_04820|KEGG|CYP133B5|[Ralstonia syzygii subsp. Celebesensis]rsg|BP

MKLADLSTPSFLENPYPLYETLREQGPFVRIGPNGLMTGRYSIVDALLHNRQMGKNYMDS
IRLRYGDKGPDMPLFQGFSRMFLLINPPAHTRLRSLMMQAFNARQIESMRELATATAHQL
IDGFEQKQSADLVAEFAFPLPVRIICRMLDVDIDDAPALGVAASKLAKVFDPAPMSADEL
VKTSAAYEELAQYFTKVIEARRAQPGTDLISMLLRVEEDGQKLTHDEIVSNVILLFIAGH
ETTSNMIGNALIALHRNPRQLDLLKRDPSRMPNAVLECLRYDGSVQVTIRAAMEDVEVEG
EVVPRGTTVFLMLGAANRDPAQFTDPDQLDIGRQQGRPQTFGAGIHHCLGYRLALIELES
ALNALFERLPHLRLTNLDQLSWNQRGNLRGVNALMAAW

>JK151_14030|KEGG|CYP1464A2|[Ralstonia syzygii subsp. Celebesensis]rsg|BP

MGDMQDTAAVAERDTVAGQQRNRATVDPLEVARDGKPASVARPVAGNLAMKDGFPVMPGK
VPLLGHVHRISQDALGELRKAEAACGPMFWTYFGARLPVLQILDEEGLAILQNKYTDNSF

LREQMPVITGAAMNAFDGPRHRNARHASSAAFSPTGLTRAQVGQFVVESIEQRLKRWTRE
SKLAIFPKTKDIALEVVFRILGIKTHELAQWRRQYEEFFLGMIPLKINLPGFPAWRCRKA
RGWLEARVAQIVATARADDDHDSLVGAMIFGRDDNGNGMSEVELVHNILGLGFAGSETTA
SVMAWSALMLAQHPDVWRQLCRQVAGMDSVPSTQEELVRQAPLAEAIFRETMRLYPPAPF
EMRKVHTEFEMMGHKIPAGVMVGVSLLHVSRNPERYPDPDSWKPERWLGVDRALTPVETC
GFGGGPHACLGRHVAALEITLFITMLARELGPKGIVPRLAGKLPAPAYLPFLRPSNKAFL
DMTGR

>H16_B1009|KEGG|CYP102E2|[Cupriavidus necator]reh|BP
MPPPIELSSPDQASDAPHQAPARSMHAPIPEPIPRDPGWPLVGNLLQITPGALGQHLLAR
SRHHDGIFELNFAGRRVPFVTSVALASELCDAAQFRKYIGPPVSYLRGMAGDGLFTARSD
EANWGKAHRILMPAFSQRAMKGYFDVMLRVANRLVDKWDQQGPDADIAVADDMTRLTLDT
IALSGFGYDFESFASTELHPFIEAMVGALEEAMSKLTRFALQDRFMHAAHQKFDQDTRFM
RDLVDDVIRRRRAGDAAERPGGTANDLLGLMLEARDPDTDQRLDDENIRNQVITFLIAGH
ETTSGLLTFALYELLRNPGVMAQAYAEVDAVLPGDAAPVYADLARLPVLDRVLKETLRLW
PTAPAFAVAPFEDTLLGGRYLIRKDRRLSVVLTALHRDPKVWADPERFDIDRFLPEQEAK
LPRHAYMPFGNGERACIGRQFALTEAKLALALMLRNFQFTDAHDYQFRIKETLTIKPDGF
TVRARRRRPHERIAAAPLAGTAQAPRAGPDVQGRGRTLAVLCGSSLGTARELAEQVHAGA
LAAGFDATLRDLDDVADALPTTGLAVIIAATYNGRAPDSARRLEALLDSGAASGYRAEAL
SVAVLGCGNSQWPTYQAFPRRVYEFFTNAGASPLLPRGEADGNGDFDQAVERWLALLWQA
LQAGDGVGGPAHGTPPVRVQVKDVAEIRASTLPANTEAFTVLANTELVNDPSGLWDFSHE
APRTSTRDIRLRLPDGVRYATGDHLAVYPQNQPGMVAALCERIGIDPDAIVTLSASGGAA
RGLPLGEALSVRQLLTHFVELQDVVSRHTLRLLSQSSRCPVTRQALRQLASDDAGSGYAA
QVAPRRLGLADVLDRFPAIEVDLAGLLACTVPMRPRFYSIASSPLVSPGVATITVGTVWS
PALSGRGLFRGVASTWLQGLAPGAMVAGAIRTPNPTFAPAADPATPMILIGPGTGIAPFR
GFLEERAAQQAAGQPVAPVQLYYGCRHPAHDWLYRNNVERWQAQGVVQVHLACSVVDGEP
RYVQDLLWQRRADVWARLNQGAIIYVCGDGRHMAPAVRQVLIQIGAEQGGMTPEAASDWL
ADLVSAGRYRQDVFN

>H16_B1279|KEGG|CYP116B137|[Cupriavidus necator]reh|BP

MANPSPSAARSGCPIDHSALTAPNGCPVSHNAAQFDPFGDGYQQDPPEYVRWSREQEPVF
YSPRLGYWVVTRYEDIKAIFRDNLTFSPSIALEKITPTGDEANAVLASYGYAMNRTLVNE
DEPAHMPRRRALMAPFTPAELAHHEPLVRRLAREYVDRFIDDGRADLVDQMLWEVPLTVA
LHFLGVPEEDMDLLRQYSIAHTVNTWGRPKPEEQVAVAHAVGNFWQLAGRILDKMREDPS
GPGWMQYGLRKQKELPDVVTDSYLHSMMMAGIVAAHETTANASANAIKLLLQHPDAWREI
CDDPSLIPNAVEECLRHNGSVAAWRRLVTRDAEVGGIRLPAGSKLLIVTSSANHDERHFA
DADLFDIRRDNASEQLTFGYGSHQCMGKNLARMEMQVFLEELTRRLPHMRLAEQTFTYVP
NTSFRGPEHLLVEWDPAQNPERRDPALLEVHQPVRIGEPSAHTIARTVVVERVTPAADGV
VRLRLAAPDGKPLPRWAPGSHIDVECGDTGLSRQYSLCGDPDDTGALEIAVLRDPDSRGG
SAWVHGSVQAGDRLRIRGPRNHFRFDEGCGRAIFIAGGIGITPVSAMARRARALGIDYEF
HYCGRSRQAMAMLDELQALHGARLHVHASDEGQRADFGKLLARPDPRTQIYACGPQRMLD
ALAESCAAWPEDALRVEHFVSKLGTLDASKELPFSVELKDSGLVMEVPAGQTLLSALRGA
NIDVQSDCEEGLCGSCEVRVLAGQVDHRDVVLTRSEREANQRMMACCSRACGGGRLVLEL

>H16_B1743|KEGG|CYP194C2|[Cupriavidus necator]reh|BP
MTDTNQHALLHDGYDLLSDHYVQEAHALWRDIRSSGCPVAHSEKWGGSWLPTTYDDIHHVAQNPAVFSSRAAEIAGEVPPQGSGLVLPPLTSDPPD
HKIHRDLLEPYFTPARVAAIEPYAQSLARDLARRVAVKGEADLGEDYSKPFVLSLLTRFLDVPDDRQERFMDWAIRVLKYGPFDQELRKAAFDEAF
ADLEQLLKEREQDPGEDLVSHIALATIDGKPISRKHRIGSLLLAVLAGADTTWNALNASLNHLADHPADRATLINEPGLLRTTAVEELLRFYAPLS
IARVTTEEVELKGRCIGAGERVILAYPAANRDPAVFENPDEVQLDRKRNRHLTFGVGVHRCLGSHLARMEMRVAIEEWLKAIPNFERISGAVKWSA
GNARGPENVRIRVV

>H16_B2406|KEGG|CYP229F14|[Cupriavidus necator]reh|BP
MPDTDIDPLSAVTHPDPYPYYRELAASQPFFRDDRLGLWVAAGPQEVADVLAHSDCRVRPPAQPVPPALAGTAAGELFGRLVRMNDGAAHAPLKAL
LMPMLAGIDPAAAAQRATVLAAVLDAGEASWAAMSGECINRWLFTLPVVTVADLLGLPVANEGSSAAEAAQRVAAFAGAQSPLADAPAVRAGAEAA
QWLGHWLADAADGAGPLPALRQAARAAGIDAQAVAANIIGLLVQACEATAALAGNTLLRLGRDTTQSGLPLDAVVARVAREDPPVQNTRRFLAADA
QLCGHAVKAGDAVLVLLAAASCSGAAASERPWTFGHGRHACPGDRLAQALAAATVAALRARGADPAALAQAFRYRPSLNARIPHFL

>CNE_2c09660|KEGG|CYP102E2|[Cupriavidus necator]cnc|BP
MPPPIELSSPDQASDAPHPSPARPMHAPNPEPIPRDPGWPLVGNLLQITPGALGQHLLAR
SRHHDGIFELNFAGRRVPFVTSVALASELCDAARFRKYIGPPLSYLRGMAGDGLFTARSD
EANWGKAHRILMPAFSQRAMKGYFDVMLRVANRLVDKWDRQGPDADIAVADDMTRLTLDT
IALSGFGYDFESFASANLHPFIEAMVGALEEAMSKLTRFALQDRFMHAAHQKFDQDTRFM
RDLVDDVIRRRRAGNAAQRRGGTANDLLGLMLEARDPDTDQRLDDENIRNQVITFLIAGH

ETTSGLLTFALYELLRNPGVMAQAYAEVDAVLPGDAAPVYADLARLPVLDRVLKETLRLW
PTAPAFAVAPFEDTLLGGRYLIRKDRRLSVVLTALHRDPKVWADPERFDIDRFLPEQEAK
LPRHAYMPFGNGERACIGRQFALTEAKLALALMLRNFQFTDAHDYQFRIKETLTIKPDGF
TVRARRRRPHERIAAAPVAGTAQAPQAGPDVQGGGRPLAVLCGSSLGTARELAEQVHAGA
LAAGFDATLRDLDDVADALPTTGLAVIIAATYNGRAPDSARRLEALLDSGAASGYRAEGL
SVAVLGCGNSQWPTYQAFPRRVYEFFTNAGASPLLPRGEADGNGDFDQAAERWLASLWQA
LQAGDSVGGPAHGTPPVRVQMRDVAEIRASTLPANTQGFTVLANTELVNDPSGLWDFSRE
APRTSTRDIRLRLPAGVRYATGDHLAVYPQNQPGMVAALCERIGIDPDAIVTLSASGGAA
RGLPLGEALSVRQLLTHFVELQDVVSRQTLRLLSQSSRCPVTRQALQQLASDDAESGYAA
QVAPRRLGLADVLDRFPAIELDLAGLLACTVPMRPRFYSIASSPLVSPDVATITVGTVWS
AALSGRGLFRGVASAWLQGLAPGAMVAGAIRTPNPAFAPAADPATPMILIGPGTGIAPFR
GFLEERAAQQAAGQPVAPVQLYYGCRHPAHDWLYRDDVARWQAQGVVQVHLACSVVDGEP
RYVQDLLWQRRADVWARLNQGAIVYVCGDGRHMAPAVRQVLIQIGAEQGGMTPEAASDWL
AELVSTGRYRQDVFN

>CNE_2c12500|KEGG|CYP116B156|[Cupriavidus necator]cnc|BP

MAKPSPSAARSGCPIDHSALTAPNGCPVSHNAAQFDPFGDGYQQDPPEYVRWSREQEPVF
YSPSLGYWVVTRYQDIKAIFRDNLTFSPSIALEKITPTGDEANAVLASYGYAMNRTLVNE
DEPAHMPRRRALMAPFTPAELAHHEPLVRRLAREYVDRFIDDGRADLVDQMLWEVPLTVA
LHFLGVPEEDMDLLRQYSIAHTVNTWGRPKPEEQVAVAHAVGNFWQLAGRILDKMREDPS
GPGWMQYGLRKQKDLPEVVTDSYLHSMMMAGIVAAHETTANASANAIKLLLQHPDAWREI
CDDPSLIPNAVEECLRHNGSVAAWRRLVTRDAEVGGIRLPAGSKLLIVTSSANHDERHFA
DADLFDIRRDNASEQLTFGYGSHQCMGKNLARMEMQVFLEELTRRLPHMRLAGQTFTYVP
NTSFRGPEHLLVEWDPAQNPERRDPALLSVHQPVRIGEPSAHTIARTVVVECVTPAADGV
VRLRLAAPDGKPLPRWAPGSHIDVECGDTGLSRQYSLCGDPDDAAALEIAVLRDPDSRGG
SAWVHGSVQAGDRLRIRGPRNHFRFDEGCKRAIFIAGGIGITPVSAMARRARALGTDYEF
HYCGRSRQAMAMLDELQALHGARLQVHASDEGHRADFGKLLAQPDPRTQIYACGPQRMLD
ALAECCAAWPEDALRVEHFVSKLGTLDASKEQPFTVELKDSGLVMEVPAGQTLLSALRGA
NIDVQSDCEEGLCGSCEVRVLAGQVDHRDVVLTRSEREANQRMMACCSRACGGGRLVLEL

>CNE_2c23570|KEGG|CYP229F9|[Cupriavidus necator]cnc|BP

MKRSPPRTRQTGSTSSPQRAAMQTATLPDTGIDPLRAVTDPDPYPYYRELATRQPFYRDD
RLGLWVAAGPREVAAVLAHPDCRVRPPAQPVPPALAGTAAGDLFGRLIRMNDGAAHAPLK
QILMPMLAGLDAAATARRAAAMATMIDAGAAAGTSGERVNRWLFTLPVVTVADLLGLPIA
EGGKAAEVAQRVAAFAAAQSQLADAHTVRAGADAAQWLGAWLAGATPGDGPLPALHQAAH
AAGIDAQAVAANIIGLLVQACEATAALAGNTLLRLGRDTPQSELPLDAVVARVAREDPPV
QNTRRFLAADAQLCGHAVKAGDAVLVLLAAASCSGAAAGERPWTFGHGRHACPGDRVAQA
LAAATVAALRARGADPAALAQAFRYRPSLNARIPHFL

>CNE_BB1p04290|KEGG|CYP1077A2|[Cupriavidus necator]cnc|BP

MDLHDNSPAKATLSFDPAGSGHDAWSENFATSRKECPVFWSEAHGGYWTVANHESATRVA
RDWQTFSSQKSWDPATGHHEGGNVIPSIPGPAFIPVETDPPLWKRYRLLLNPFFAPKAAE
RFRPIAEEIATALMDRVIEKGSFDIVKDFSNPVPALTTLRILGIPFDADNWERFADPFHK
LAYARELPEFPEVLANLEWIRGKLADLADACHQHPGDGLFSTLCHAEAEGASLSKQELVD
IGMMLLVGGVGTTNALFANTMIYLDKHRAILERLVSNPDMLPIAIEEFVRFFSPVSGQAR
LVKCPVDVGSQHMRPGEPLLLALSSANRDESVFRNPDEVMVDRMPNPHIGFGGGNHRCLG
SFFAREMFTAMFREMTKRMPDYAVDHDRASRYPCVASVNGWISAPARFTPGKKVGSAVKL

>CNE_BB1p08730|KEGG|CYP108S1|[Cupriavidus necator]cnc|BP
MNHNDFDLPGDVASVLVDPRAYADPVRLHAAYTWARENNPLGRAVNDGFDPFWVVTKHADVTAISRDHNLFHNGDYSVVCRPKAMIDHVIATTGSP
NVVRALVHVDGDEHKGLRGLTQSWFMPNSILKLDERIRALARQIVDKLQKSDGNTIDFAKDIALHYPLHVIMDILGVPPEDEARMLMLTQELFGGE
DPELKRAKQEQNPNANVSAQSMMAVVADFKDYFERVTADRRVNPRNDVATLLANAVVNGEPISEVSRLGYYVIIATAGHDTTSSSSAVAMWALSQF
PELLPRLQADPSLISQFIDEAVRFASPVRHFMRTATADTEIRGRKIRKGDWLMLCYGSANRDEEVFEKPFDFNIDRKPNRHLAFGFGAHVCLGQHL
AKMEMRILFEEMIPRLKRVEPAGQMELIASTFVGGPKHLPVRCVMQ

>CNE_BB1p09050|KEGG|CYP107DG4|[Cupriavidus necator]cnc|BP
MNTATLSVRANPGPDTAIELRDPCPPILDAAFLRNPYPAYHRLRAAGPIHWSEEFFGGAW
LLTRHEDVEAVLRDPLYSARRTGGWVMRGSEDERQALCPFQRLFSRAMLFLDAPDHMRLR
QALNAGFRPAALRAFAGTIEQMATGLMDRLEGTEQFDFMEGIAKPLPAMVIASLLGADAG
DQPDFLAWSEDLAAFIGSPAPDQGLKRRAQASLLKMSAAFEALLARRRAQHATRPQTEQT
DLIGHLLHAEAEGRIEAGPELLAQCAMLLFAGHETTRNLLGNGLHALLSHPEQWQRLRQS

PELLPNALRELLRYESPVQYTGRRVTTDMVLNGRRMQRGDLVVPLIGAANRDPARYRNPD
ELDITRREGSHLSFGHGPHVCIGAALTLMEAEIAFRALLTRWPGLELVESEPHWNSNPVY
RGLASLQVRHLS

>CNE_BB1p09240|KEGG|CYP108S6|[Cupriavidus necator]cnc|BP
MQTVFATTRNPGRKTKIYSGDTMKLDEFTLPAHIAETVVDPKAHADLDRLHAAYTWARAN
NPLGCAEVEGYDPFWVVTKHADVALIGRDNQTFHSADRSVTCLPKEALDHIKSTTGNAEL
IKSLVRLDDPEHKKLRALTMAWFMPNSILKLDERMRALAKSTVDKMAAQSGGVVDFANDV
AVHYPLHVIMDILGVPPEDEPRMLMLTQQLVGSDDPELGRDKGAAKGSKEKMDSRREVVA
DFAQYFNGITADRRANPRNDVASLLANAVIDGEPLSDTLRLGYYILIATAGHDTTSSSTA
TAMWQLSQSPELLSRLKADPSLISGFVDEAVRFISPVRHFMRTATTDVDLRGRRIRKGDW
LMLCYASANRDEEVFDDPFVFNIDRKPNRHLGFGFGPHVCLGQHLAKMEMRILFEAMIPR
LQWVEPAGEMNLVESNLVTGPKRLPVRYEMN

>BJN34_26235|KEGG|CYP102E2|[Cupriavidus necator]cuh|BP

MPPPIELSSTEQAADTPSPSPAGTAREPIPVDPGLPLVGNLLQIKPGALGQHLLARSRHH
DGIFELNFAGRRVPFVTSVALASEVCDATRFRKYIGPPLSYLRGMAGDGLFTAHSDEANW
GKAHRILMPAFSQRAMKGYFDVMLRVANRLVDKWDRQGPDADIAVADDMTRLTLDTIALS
GFGYDFESFARADLHPFIEAMVGALEEAMGKLTRFALQDRFMHAAHRKFDEDTRFMRDLV
DDVIRRRRADGAAGPGHGTKNDLLGLMLEARDPDTDQRLDDENIRNQVITFLIAGHETTS
GLLTFALYELLRNPGVMAQAYAEVDAVLPGDAAPIYADLARLPVLDRVLKETLRLWPTAP
AFAVAPFEDTLLGGRYLIRKDRRMSVVLTALHRDPKVWADPERFDIDRFLPEREAQLPRH
AYMPFGNGERACIGRQFALTEAKLALALMLRNFQFTDAHDYQFRIKETLTIKPDGLTVRA
RRRRPHERIAAAQATATTQAVQAGPEVLGGGRPLAVLCGSSLGTARELAEQVHAGAVAAG
FDATLRDLDEVADALPATGLAVIIAATYNGRAPDSARRFEALLDSGAASGYRAQGLRVAV
LGCGNSQWPTYQAFPRRVYDFFTQAGATPLLPRGEADGNGDFDQAAERWLALLWQALQAG
DGADGPAHGTPAIRVQVKDVAEIRASTLPANTQGFTVLANAELVNDPTGLWDFAREAPRT
STRDIRLRLPEGVRYATGDHLAVYPQNQPEIVTALCERIDIDPDAIVTLSAPGGTARGLP
LGEALSARQLLTHFVELQDVVSRQTLRLLSQHSRCPVTRQALQQLASDEAETGYAAQVAS
RRLGLADVLDRFPAIELDLAGLLACAVPMRPRFYSIASSPLVSPDVATITVGMVWSPALS
GRGLFRGVASTWLQGLAPGAMVAGTIRTPNPAFAPAADPATPMILIGPGTGIAPFRGFLE
ERAAQQAAGQAVAPVQLYYGCRHPLHDWLYRDDVERWRAQGVAQLHLACSVVDGEPRYVQ

DLLWQRRADVWTRLGEGAIVYVCGDGRHMAPAVRQVLLQIGAEQGGMTPEAASDWLAELV
STGRYRQDVFN

>BJN34_28050|KEGG|CYP116B155|[Cupriavidus necator]cuh|BP
MANPSPGTARGCPIDHSAFTAPNGCPVSHNAAEFDPFGDGYQQDPPEYVRWSREQEPVFY
SPKLGYWVVTRYDDIKAIFRDNLTFSPSIALEKITPTGDEANAVLASYGYAMNRTLVNED
EPAHMPRRRALMAPFTPAELAHHEPLVRRLAREYVDRFIDDGRADLVDQMLWEVPLTVAL
HFLGVPEEDMDLLRQYSIAHTVNTWGRPKPEEQVAVAHAVGNFWQLAGRILDKMREEPSG
PGWMQYGLRKQKDLPEVVTDSYLHSMMMAGIVAAHETTANASANAIKLLLQHPDAWREIC
EDPSLIPNAVEECLRHNGSVAAWRRLVTRDAEVGGIRLPAGSKLLIVTSSANHDERHFAD
ADLFDIRRDNASEQLTFGYGSHQCMGKNLARMEMQVFLEELTRRLPHMRLAGQTFTYVPN
TSFRGPEHLLVEWDPAQNPERREPALLDVHQPVRIGEPSAHTIARAVVVERVTPAADGVV
RLRLAAPDGKPLPRWAPGSHIDVECGDTGLSRQYSLCGDPDDAGALEIAVLHDPDSRGGS
AWVHGSVNAGDRLRIRGPRNHFRFDEGCGRAIFIAGGIGITPVSAMARRARALGIDYEFH
YCGRSRQAMAMLDELQALHGARLHVHASDEGRRADFGKLLAQPDPRTQIYACGPQRMLDA
LTDCCAAWPEDALRVEHFVSKLGTLDASKEQPFTVELKDSGLVMEVPAGQTLLSALRGAN
IDVQSDCEEGLCGSCEVRVLAGQVDHRDVVLTRSEREANQRMMACCSRACGGGRLVLEL

>BJN34_31475|KEGG|CYP108S1|[Cupriavidus necator]cuh|BP
MNHTDLDLPGDVAKVLVDPRAYADPARLHAAYAWARANNPLGRAVNDGFDPFWVVTKHAD
VTAISRDHNLFHNGDYSVVCRPKASIDHVIATTGRPNIVKALVHVDGDEHKGLRALTQSW
FMPNSILKLDERIRGLARQTVDKLQQSDGSTIDFAKDIALHYPLHVIMDILGVPPEDESR
MLLLTQELFGGEDPELKRAKQEKSADDGAMAKSLMAVVADFRDYFEKVTAERRVNPRNDV
ATLLANAVVNGEPISEISRLGYYVIIATAGHDTTSSSSAVAMWALSQYPELLPRLQADPS
LIPQFIDEAVRIASPVRHFMRTATADTEIRGRKIRKGDWLMLCYGSANRDEEVFDQPFDF
NIDRKPNRHLAFGFGAHVCLGQHLAKMEMRILFEELIPRLQRVEPAGEMEMIASCFVGGP
KHLPVRCVM

>Reut_B5115|KEGG|CYP229F13|[Cupriavidus pinatubonensis]reu|BP
MMPADPREAVTAPDPYPYYRALATRQPFGFDERLGLWVAAGRAEVAAVLAHPDCRVRPLS
QPVPTALAGTAAGELFGRLIRMNDGAAHAPLKAILMRLMASMDAATAKTRSAGLAGVLDL
VPDMAPLNGATINRWLFTLPVVTVADLLGLPVRAGADAMAVAARVAAYAAAQSPLATAGD
VEAGADAARWLGQWLKMPAGRPCPAEDLGLLPALRQAAHADDIAPEVVAANVIGLMVQAC

EATAGLAGNMLVRLGRSSPTTRGLDELAVRVAIDDPPVQNTRRFLAADARVCGVPVRAGD
AVLVLLAAASCAAPVEAGARPWTFGAGRHACPGDALARALAAATVDALLARGAQPTALAQ
AFRYRPSHNARIPHFL

>Reut_B5278|KEGG|CYP116B160|[Cupriavidus pinatubonensis]reu|BP

MTAATPETTTQKCPFSHGAQPAAAAPNGCPVSAEAAAFDPFGDGYQQDPPEYVRWSREQE
PVFYSPQLGYWVVTRYDDIKAIFRDNLTFSPAIALEKITPTGPEANAVLASYGYAMNRTL
VNEDEPAHMPRRRALMEPFTPEQLKHHEPLVRKLAREYVDRFIDDGKADLVDQMLWEVPL
TVALHFLGVPEEDMDLLRQYSIAHTVNTWGRPKPEEQVAVAHAVGNFWQLAGRILDKMRA
DPSAPGWMQYGIQKQKELPEVVTDSYLHSMMMAGIVAAHETTANASANAIKLLLQHPDVW
REICEDPALIPNAVEECLRHNGSVAAWRRVVTRDTEVGGIPIAAGSKLLIVTSSANHDEA
HFADADLFDIRRENAADQLTFGYGSHQCMGKNLARMEMQIFLEELTRRLPHMRLAQQSFT
YLPNTSFRGPEHLWVEWDPAQNPERTQPSLLAQQTPVRIGEPSGQAISRPVVVQSVTQAA
DGIVRLRLASPDGKAMPRWSPGSHIDVDCGETGLSRQYSLCGDPAETGVLEIAVLREPES
RGGSAWIHDHVKPGDRLKIRGPRNHFRMDEGCRKAIFIAGGIGITPVSAMARRALALGID
YEIHYSGRTRTGMAMIDELRALHGERLHLYIKEEGGRNDLDALLARTDRNAQIYACGPAR
MVEALEQACTGWPDDALRVEHFATTLGTLDPSKEQPFEVELKDSGLTVTVPADQTLLTTL
RCAGIDIQSDCEEGLCGSCEVRVLSGSVDHRDVVLTRGEREANTKMMTCCSRALPGSRLI
LEL

>Rmet_3467|KEGG|CYP102E1|[Cupriavidus metallidurans]rme|BP
MSTATPAAALEPIPRDPGWPIFGNLFQITPGEVGQHLLARSRHHDGIFELDFAGKRVPFV
SSVALASELCDATRFRKIIGPPLSYLRDMAGDGLFTAHSDEPNWGCAHRILMPAFSQRAM
KAYFDVMLRVANRLVDKWDRQGPDADIAVADDMTRLTLDTIALAGFGYDFASFASDELDP
FVMAMVGALGEAMQKLTRLPIQDRFMGRAHRQAAEDIAYMRNLVDDVIRQRRVSPTSGMD
LLNLMLEARDPETDRRLDDANIRNQVITFLIAGHETTSGLLTFALYELLRNPGVLAQAYA
EVDTVLPGDALPVYADLARMPVLDRVLKETLRLWPTAPAFAVAPFDDVVLGGRYRLRKDR
RISVVLTALHRDPKVWANPERFDIDRFLPENEAKLPAHAYMPFGQGERACIGRQFALTEA
KLALALMLRNFAFQDPHDYQFRLKETLTIKPDQFVLRVRRRRPHERFVTRQASQAVADAA
QTDVRGHGQAMTVLCASSLGTARELAEQIHAGAIAAGFDAKLADLDDAVGVLPTSGLVVV
VAATYNGRAPDSARKFEAMLDADDASGYRANGMRLALLGCGNSQWATYQAFPRRVFDFFI
TAGAVPLLPRGEADGNGDFDQAAERWLAQLWQALQADGAGTGGLGVDVQVRSMAAIRAET

LPAGTQAFTVLSNDELVGDPSGLWDFSIEAPRTSTRDIRLQLPPGITYRTGDHIAVWPQN
DAQLVSELCERLDLDPDAQATISAPHGMGRGLPIDQALPVRQLLTHFIELQDVVSRQTLR
ALAQATRCPFTKQSIEQLASDDAEHGYATKVVARRLGILDVLVEHPAIALTLQELLACTV
PMRPRLYSIASSPLVSPDVATLLVGTVCAPALSGRGQFRGVASTWLQHLPPGARVSASIR
TPNPPFAPDPDPAAPMLLIGPGTGIAPFRGFLEERALRKMAGNAVTPAQLYFGCRHPQHD
WLYREDIERWAGQGVVEVHPAYSVVPDAPRYVQDLLWQRREQVWAQVRDGATIYVCGDGR
RMAPAVRQTLIEIGMAQGGMTDKAASDWFGGLVAQGRYRQDVFN

>Rmet_3820|KEGG|CYP229F12|[Cupriavidus metallidurans]rme|BP
MPPNASLAPADPIAAVTHADPYPYYRDLASTRPFYFDPALKLWVAASAAAVAEVLDHADG
RVRPAAQPVPPSLAGSAAGDLFGRLIRMNDGVAHAPLKAIVARQMAALDLSDARSRARAL
AAALPRVDHDDAHEANRWMFTLPVLAIADLLGLPLERDTDGHVSTRVAGFVATFAAAMSP
LASPADVEAGVAAAQWLTRLAQTAEDRETADIGVLAALAQAGGDSKMDPATLTANAIGLM
IQSCEATAGLIGNTLVYLGRSDWQGADIGASVDRVARIDPPVQNTRRFMAADAMLCGQQV
KEGDAVLVLLAAAAHDPSSAAEERAWTFGAGRHGCPGDALARVLATCTVDALLARDVHPD
RLAAGVRYRPSVNARIPSFSSNN

>Rmet_3868|KEGG|CYP212A2|[Cupriavidus metallidurans]rme|BP

MTTESQAQCPFQTAKSPPPAGSPLPHPYGTWPPGPAAGLTGWHLLRRMSRDLLGTLGEWQ
QTYGDVVHLRMWPEHAVVVTDPQLVRELLVTHHDSLIRWERGTRVFSRVHGHSVLTAEGD
AWSRKRQALQPGFMPKAVHGFVPGIVEIVDKGLATWPTRVADWPVESALTSLTMDVIVRM
MFSDEIGEDARVAECAVRAISEAANADFYWPASLPDWVPWKRARRRALHTLRDLIERHLQ
ARLRMRTDTWPDDLLSRLLCLHRDDATAWPLQAVRDECMTTFLAGHETTAATLTWWAWCM
ASNPSAQDAARAEVTHVLRGQAPTADSRQALRQVVQTITESMRLYPVAPVLISRRAVRPI
TLGPWRLPARTLFMLPLQLMHHDPRLFPEPERFQPDRFSTGSPQAPRGAYMPFGTGPRVC
LGQHLATAEMTVIAAMLLQRYKLSVPEGAAHPRPLLNVTLRPDQPLWLAVTPI

>Rmet_4932|KEGG|CYP116B1|[Cupriavidus metallidurans]rme|BP

MPQTNAPASSGSCPIDHSALRAPNGCPISHQAAAFDPFEDGYQQDPPEYVRWSRAQEPVF
YSPKLGYWVVTRYDDIKAIFRDNITFSPSIALEKITPTGEAANAVLASYGYAMNRTLVNE

DEPAHMPRRRALMEPFTPAELAHHEPMVRKLTREYVDRFIDTGRADLVDEMLWEVPLTVA
LHFLGVPEEDMDLLRQYSIAHTVNTWGRPKPEEQVAVAHAVGNFWQLAGRILDKMREDPS
GPGWMQYGLRKQRELPEVVTDSYLHSMMMAGIVAAHETTANASANAIKLLLQHPDVWREI
CEDPALIPNAVEECLRHNGSVAAWRRLVTRDTEVGGMSLAAGSKLLIVTSSANHDEHHFA
DADLFDIHRDNASDQLTFGYGSHQCMGKNLARMEMQIFLEELTSRLPHMRLAGQRFTYVP
NTSFRGPEHLWVEWDPARNPERTDPTVLAPRDAVRIGEPTGGTTGRTLIVERVETAAQGV
SRIRLVSPDGRALPRWSPGSHIDIECGHTGISRQYSLCGDPADTSAFEIAVLREPESRGG
SAWIHASLRAGDKLKVRGPRNHFRLDETCRRAIFIAGGIGVTPVSAMARRAKELGVDYTF
HYCGRSRASMAMIDELRALHGDRVRIHAADEGQRADLAQVLGAPDANTQIYACGPARMIE
ALEALCATWPEDSLRVEHFSSKLGTLDPSREQPFAVELKDSGLTLEVPPDQTLLATLRAA
NIDVQSDCEEGLCGSCEVRVLAGEIDHRDVVLTRGERDANNRMMACCSRAAKGGKIVLGL

>Rmet_5516|KEGG|CYP108E1|[Cupriavidus metallidurans]rme|BP
MTIASDFDTELASHEIYSDPERMHEMFETLRREDPVHWTTAPGHPPFWAVTKQADVIEVG
KHPDVFIASPKSFLMNDVEQRVRIEETAATGGKLVRTMIHMDDPDHKKYRGLTQSYFMPA
NIKRLESVIQERARALVGRLIEKGTSEFCSEIAVWYPLQIVMTLLDVPESEHPYLLKLTQ
QFLAPKDPTLRRDGPDERGKGAVAKEYFAYFGKMLAERRAAPLKEDLGSLIAHATVDGEP
LPLMEAVSYYVILATAGHDTTSSSMCSGLYYLLTQPGELDRLRARPELMPSAIEEMFRHG
SPVKHFVRTATRDFELRGKKIQAGDEVALMYHSASFDEEVFDEPRSFRIDRGPNKHVAFG
FGIHACLGQNLARASMRTFFTELLARTESIEVVGKAEFIASNQVGGMKTLNIRVTPSKQS
TTDRIEVAA

>RALTA_B1182|KEGG|CYP116B170|[Cupriavidus taiwanensis]cti|BP

MTTPSPGAARGCPIDHSMLTAPNGCPVSHNAAQFDPFGDGYQQDPPEYVRWSREQEPVFY
SPQLGYWVVTRYDDIKAIFRDNLTFSPSIALEKITPTGDEANAVLASYGYAMNRTLVNED
EPAHMPRRRALMAPFTPAELAHHEPLVRRLTREYVDRFIDDGRADLVDQMLWEVPLTVAL
HFLGVPEEDMDLLRQYSIAHTVNTWGRPKPEEQVAVAHAVGNFWQLAGRILDKMREDPSG
PGWMQYGLRKQKELPEVVTDSYLHSMMMAGIVAAHETTANASANAIKLLLQHPDAWRELC
EDPALIPNAVEECLRHNGSVAAWRRLVTRDAEVGGIRLPAGSKLLIVTSSANHDERHFAD
ADLFDIRRDNASEQLTFGYGSHQCMGKNLARMEMQVFLEELTRRLPHMRLAEQTFTYVPN
TSFRGPEHLLVEWDPAQNPERRDPALLAVRQPVRIGEPSAHTIARTVVVERATPAADGVL

RLRLAAPDGKPLPRWAPGSHIDVECGDTGLSRQYSLCGDPDDAAALEIAVLRDPASRGGS
AWVHDHVRAGDRLRIRGPRNHFRFDEQCRRAIFIAGGIGITPVSAMARRARALGIDYTFH
YCGRSRQAMAMLDELQALHGARLHVHASNEGRRANFGKLLGQPDADTQIYACGPQRLLDA
LAECCMAWPADALRVEHFVSRLGSLDASKEQAFTVELKDSGLVLEVPAGQTLLGALRGAN
IDVQSDCEEGLCGSCEVRVLAGQVDHRDVVLTRAERDANHRMMACCSRACGGGRLVLEL

>RALTA_B2169|KEGG|CYP229F15|[Cupriavidus taiwanensis]cti|BP

MPETITDPFADIDPFSAVTHPDPYPYYRELAATRPFFREQRLGLWIAAGSSEVAAVLAHP
DCRVRPPAQPVPPALAGTAAGDLFGRLIRMNDGAAHAPLKALLMPMLAAVAPGVAAQKAA
AMAQVLDAGEGAHVAMPAASVNRWLFALPVVTVAAVLGLPVARDHGTAVEVAQQVAAFAA
AQSPLADADTVRAGAEAARWLGTWLASSLASSVAADGPLSALQHAAQAAGIDAQAVAANI
IGLLVQACEATAGLAGNTLLWLGRNPSASGMPLQGVVARVAQDDPPVQNTRRFLAADTQL
CGHAVKAGDTVLVLLAAASTSGLADDARAWTFGQGRHACPGDRLAQALAGATVAALCARG
ADPAALARAFRYRPSQNARIPHFL

>pRALTA_0449|KEGG|CYP108S3|[Cupriavidus taiwanensis]cti|BP
MFILAPITTTSNFDLPDDIAKLLVDPKTYSDRELLHSVYTWARANNPFGRAVIEGFDPFW
VATKYADVSAISRDSSLFRNGDYSVVCRPKAAIDYIISVTGSPHIVKALVQFDGDEHKKM
RALAQSWFMPDSLLNLDERIRRLARCCVDKLAHYEGEVIDFSRNIALYYPLHVIMDILGI
PSEDEAKMLVLTQELFGGEDPELNRGKAEVASGKDVLAKNLLAVVEDFRHYFDKLTSDKR
TSPRNDMATLLSNAVVNGEPISDANRLGYYIIIATAGHDTTSSSSAIAMWALSQFPDLLP
RLQADAALIPQFVDEAVRFASPVSHFMRTASADTEIRGRTVHKGDWIMLCYGSANRDEDV
FDKPFEFSIDRKEVHHLAFGTGPHICLGQHLAKMEMRILFEEMIPRLERVESAGELEMVA
SSFVGGPKHLPLRCFMR

>RR42_m2044|KEGG|CYP107DG5|[Cupriavidus basilensis]cbw|BP

MSAPARLATFARLGMHAQMEAEAQTEAVIEKPASCRPFVDATFLHDPYPLYRELREAGPI
HWSEEFFGGAWLLTRHDDVEAVLRDPLYSARRTGGWVMRGNEESRQTLCPFQRLFSRAML
FLDAPDHTRLRQVLNAGFRPSTLRASAGAIEHMVAGLMSGIDEAGPFDFMERIARPLPAM
VIARLLGMDAADQPEFLAWSEDLAAFIGAPDPDQGLKRQAQVSLLKLSAAFEAMLARRRE
QGATVLDAAGDDLIGHLLRAEASGEVESGAELLAQCAMLLFAGHETTRNLLGNGLYALLS

HPDQWRRLQRAPELLPNALRELLRYESPVQYTGRRVTTDVVLHGRRLRRGELVVPLIGAA
NRDPARYSNPDSLDISRREGSHLSFGHGPHFCIGAALTLMEAEIVFRALSARWPELALLD
ASPHWNGNPVYRGLASLQIRCLP

>RR42_m3629|KEGG|CYP116B151|[Cupriavidus basilensis]cbw|BP

MANATPNPHASGCPVDHRLLTAQRSPTGCPVSPGAASFDPFEDGYQQDPPEYVRWAREQE
PVFYSPKLGYWVVTRYDDIKAIFRDNLTFSPSIALEKITPTGPQANAVLASYGYAMNRTL
VNEDEPAHMPRRRVLMAPFTPEELKHHEPMVRKLAREYVDRFVDDGRADLVDQMLWEVPL
TVALHFLGVPEEDMDLLRKYSIAHTVNTWGRPRPEEQVEVAHAVGNFWQLAGKILDKMRQ
DPSGPGWMRYGIRKQVEYPEIVTDSYLHSMMMAGIVAAHETTANATANAMKLLLQHPRAW
QEICEDPGLIPNAVEECLRHNGSVAAWRRLATRDVQVGGVDIPAGAKLLIVTSSANHDER
HFADADLFDIRRENASDQLTFGYGSHQCMGKNLARMEMQVFLEELTRRLPHMRLAEQSFS
YVPNTSFRGPEHMWVEWDPKQNPERANPALLEVQVPVRIGEPLGHAISRPVVVASATRAA
EGIVRLRLVSPDGKPMPRWAPGAHIDVECGGTGLSRQYSLCGDPDDNSALEIAVLREAQG
RGGSAWVHDSAKAGDRLKIRGPRNHFRLDEAAGRLILIAGGIGITPVSAMARRAKALGMD
YALHYSGRSRASMALLDELIALHGDRLHVYVKDEGRRNDFASLLGQPQADTQIYACGPAR
MLAALQQQCAGWPGDALRIEHFESTLATLDPGQEQAFEAELKDSGIVVAVAPGQTLLAAL
RAANIDMQSDCEEGLCGSCEVRVLAGEIDHRDVVLTRAEREANTKMMACCSRAKCQRIVL
EL

>RR42_m4058|KEGG|CYP102E3|[Cupriavidus basilensis]cbw|BP

MQRASTPASAAPAATSATIPAAQPARSLEPIPRDPGWPLVGNLLQVTPGKVGQHLLARSR
AFDGIFELDFAGRRVAFVSDAALVGELSDGQHFRKIVGPPLSYLRDLAGDGLFTAHAEEE
NWGRAHRILMPAFSQRAMKGYFDVMLRVANRLVDKWDRQGADTDIPIADDMTRLTLDTIA
LSGFGYDFDSFSREQLHPFIDAMVGALEEAMGKLTRFALQDKFMHKAHRKFADDIQYMRE
LVDDVVRQRRAAAGNHAGQPPAHDLLNLMLEARDPETDQRLDDVNIRNQVITFLIAGHET
TSGLLTFALYELLRHPGVLAQAYAEVDAVLPGDAQPVYADLAKLQVIDRVLKETLRLWPT
APAFAVAPLEDTVIGGRYLIRKDRRVSVVLTALHRDPKVWADPETFDIDRFLPENEARIH
PHAYKPFGNGERACIGRQFALTEAKLALALMLRNFQFSDPHDYQFRIKETLTLKPDAFTL
RARRRRAHERIAAAPAPGAASVQSQPPAVRGNGQPLAVLCGSSLGTARELAEQIHAGALA

AGFAATLRDLDEAVGALPTDGLAVIVAATYNGRAPDSARRFEAMLDSGDAGGYRAQGLRF
ALLGCGNSQWATYQAFPKRLHDFFSAAGAAPLLARGEADGNGDFDQAAESWLAALWQSLQ
ALRVEQGAAPDAAGASQIEVRLRDIATIRASTLPPSAQAFTVLANTELVNDPAGLWDFAL
EAPRTATRDIQLRLPEGASYATGDHLAVYPRNHPATVRDLCERLDLDPDAMVTLSAAPGC
AARGLPLDEALPLRELLTHFIELQDVVSRQTLRTLLQYTRCPFTRAALERLGSDHAQEGY
AARVASQRLGLVDVLVSHPAIALTLPGLLACTVPMRPRFYSIASSPLVSPDVATITVGTV
WSPALSGRGMFRGVASTWLQGLAPGAVVAAAIRTPNPAFAPDPDPARPMILVGPGTGIAP
FRGFLEERAAQKAAGRAVAASQFYYGCRHPGHDWLYRDDVARWQAEGVVQAHAAYSTTPA
ADQRYVQHLLWRDRQAIWAMLQDGATIYVCGDGRQMAPAVRRTLIEIAAQQGGMSEAAAS
DWLAGMVAQGRYRQDVFN

>RR42_s0745|KEGG|CYP116F1|[Cupriavidus basilensis]cbw|BP

MSENKAQVSRCPFSAAAKNFDPFDPTFLEQPFSTLKELRESEPVFFDEKLGYWVVTRMDD
AKAILRNQKDFSADIVLDPIVPLYQSTIENFMRSGFHGGGGSLVNDVNPEHDERRKWLAK
SLTPQRVKELEPFVREAVVKCIDGFVKDGEVDLVKMLGNQVPAVVLLNFLAIPQEDLERV
KAWAEPTILFSWGHPTESDQNHMADMLGEFWHYCKAHIAKLRDNLGDDVVSEAIRGQQEK
SLWTDDELTRMVLNFTFAGHDTTTNAAANMFETLMKHRESWDDLCRNPALIPNAVEECIR
FAPSVISHRRRAVNNITIGSAEIQANDKILIYFAAANRDDATFANGDTFDIHRDGPNRHV
TFGFGWHACYGAPLARLELRIMLEEMTKRLPHIHLKPHQRFEYAPLNTTMRGPSHVYVQW
NPALNPLPLDRPSN

>RR42_s1885|KEGG|CYP105CW1|[Cupriavidus basilensis]cbw|BP

MHAGGDSGIPDFPPERVDPLSPPPVYFQMQQGSGLGQARLWDGKIAWLITRYDDVRSVLS
DPRFSSDITNPGYPTVSAAMKVARGSNRTFITMDAPKHAEHRRMLTGEFSIRKIEALRPR
IQAIVDKLLDDFATKPQPSDLVSAFTLATPALAISELLGVPYEDHDFFQELAMVLTSSSA
TLEEAIAANHELCEVYLKGLVAKRSENPGEDILSRLIVNHVRKGDITETDVVSLARLLLI
AGHETTANTTAMGVLFLLQRPDIWNELRQDTSLVPNAVEEILRYLDVTHSGKRRVATEDI
VVNGQMIQAGDPVVVLSVSANRDSSKFEDPNVFDLRRDSRTQVSFGYGPHQCIGQPLARL
EMQIMFTALLERFPNLELAVPVESLEFKGDSLMYGVKELPVRW

>RR42_s1901|KEGG|CYP105CW2|[Cupriavidus basilensis]cbw|BP

MTESPAPTPSSNPSSAVEGRCPFDAAANEPKSACPFHAEGDSGGIPDFPPKRVDPLAPPP
VFFQMQQKGELAQGKLWDGKIAWLVTRYDDVRAVLSDSRFSSDVSHPGFPSSSAAVKVVR
SSNRTFITMDEPKHSEQRRMLTSEFTIRKIENLRPRLQAIVDKLLEDFAMGPQPSDLVSA
FTLATPALAISELLGVPYDDHDFFQEQAMIMTSSTATREEASAANQALCEYLRELVAKRS
ESPGEDILSRLIVNHVRKGDITETDVVSLGRLLLIAGHETTANTTSMGVLFLLQRPDIWE
ELRQNPSLVPNAVEEMLRFLDVIQSGTRRVALEDVVVNGQLIRAGDPVVVLSISANRDQE
QFKDPHVFDLRRDARTQIAFGYGPHQCMGQVLARVEMQIMFTALLKRLPNLRLAVPIESL
AFKSDSLMYGVRALPVSW

>RR42_s3363|KEGG|CYP1263B1|[Cupriavidus basilensis]cbw|BP

MPLPTAMPMPSDPLCAVTHPDPAPYYRILAAQRPFYRDEALGLWVASGAAEVQAVLAHPA
CRVRPPAQAVPPALAGSPAGTLFGGLIRMNDGDWHAPLKGVLRKRMACLALAGMGARVLA
LAQDLPFDAVRPGAAVNHWMFTLPVLAVADALGLPMTGKPSVAGQVVAFARAMSPLATAQ
EIAAGSEAATWLQAWMRASLSSAPPLDALVHEALAAGVAREIIAANAIGLLIQACEATAG
LVGNMLLCLGRDTSHGRERPALDCVAARVLREDPPVQNTRRFLAADVRLCGAQLQAGDTV
LVVLAAASLGQREAATPAPWTFGRGHHACPGDALASTVAQAVVAALLARGADPASLAHAF
RYRPSVNARIPYFL

>CR3_4046|KEGG|CYP116B11|[Cupriavidus gilardii]cgd|BP

MSERTEPARAQGCPFHVQPHGQANASADAVATPLSPTGCPVSARAADFDPFEDGYQQDPP
DYVRWAREQEPVFYSPRLGYWVVTRYDDIKAIFRDNLTFSPSIALEKITPTGDEANAVLA
SYGYAMNRTLVNEDEPAHMPRRRVLMEPFTPEQLQHHEPMVRRLTREYVDRFIDDGKADL
VDQILWEVPLTVALHFLGVPEEDMDLLRKYSIAHTVNTWGRPKPEEQVEVAHAVGNFWQL
AGRILDKMRQDPSGPGWMQYGIRKQKEYPDVVTDSYLHSMMMAGIVAAHETTANASANAI
KLLLQHPDAWRELCEDPSLIPNAVEECLRHNGSVAAWRRLATRDVQIGGVDIPAGAKLLI
VTSSANHDERHFADADLFDIRRANASDQLTFGYGAHQCMGKNLARMEMQIFLEEFTRRLP
HMRLSEQKFTYVPNTSFRGPEHLWVEWDPSQNPERRNPALLQVRTPVRIGEPSSHAINRT
VVVESVTRVADDIVRLRLVSPDGKPLPRWSPGSHIDVECGDTGMSRQYSLCGDPADSRAF
EIAVLREADSRGGSAWIHRELKAGDRLRIRGPRNHFRLDESARRLILIAGGIGITPISAM

ARRAKELGIDYQLHYCGRGRASMALLDELAALHGDRLHVYAKDEGARADLGALLGQPDAE
TQIYACGPARMLDALAACCASWPQDALRVEHFTSTLGALDPSKEQAFEAELKDSGIIVTV
RADQTLLAALRAANIDVQSDCEEGLCGSCEVRVLAGEIDHRDVVLTRAEREANGKMMSCC
SRARCGRIVLEL

>CR3_4532|KEGG|CYP229D7|[Cupriavidus gilardii]cgd|BP

MTHRAPYRYYRELARQQPFHRDAMLMLWVAAGPAEVAAVLSHPACRVRPPGQPVPPPLAQ
SPAGTLFARLIRMNDGQRHAPLKAVLQRQLATLDPEDVRAAASASARLLPVTDDHAATAN
RWTGALPVIAIAHLLGFTAVDDDVTGCRIATQIASFAAAMSPLAGPAEIEAGIEAAASLQ
TLPLRADTPWAKALCAEARAAGVDDDAIAANLLGLMVQSCEATAGLIGNTLLWLAHDAAA
ADDLPTTVATVLRADPPIHNTRRFMAEDARVCGVPLRRGDGILVLLAAAAAAASEVRIDN
VSASVNTPGKRGERPWTFGHGAHRCPGDALAAALAEATVAALLARGIEPAVLAQAYRYRP
SLNARIPHFL

>BKK81_01125|KEGG|CYP102E5|[Cupriavidus sp.]ccup|BP

MTQAATSSSQAPVAGRPAASLQPIPRDPGWPLVGNILQIKPGAFAQHLMARARAHEGIFE
LDFAGRRVPFVTSAALAAELADAARFRKFIGPPLSFLRDVAGDGLFTAHTDEANWGRAHR
ILMPAFSQRAMKGYFDVMLRVAMRLVDKWDRQGPDADIAVSDDMTRLTLDTIALSGFGYD
FDSFASQQLHPFIDAMVGALEEAMGKLTRFPLQDRFMHAAHRKFAEDTRFMRELVDDVIR
QRRQSPTPARDLLNLMLEARDPETDQRLDDVNIRNQVITFLIAGHETTSGLLTFALYELM
RHPGVLAQAYAEVDAVLPGDAPPAYADLARLQVLDRVLKETLRLWPTAPAFSVAPFEDTV
IGGRYRIHKDRRVAVVLTALHRDPAVWDDPERFDIDRFLPENEARIHPHAYKPFGNGERA
CIGRQFALTEAKLALALMLRHFQLHDPHDYQFRIKETLTLKPDHFLLRARRRPHERIVV
AAPASGAVTAAAARPVVAAGGETLAVLCASSLGTARELAEQVQAGALAAGYEATLHDLDD
ALGKLPTQGLAVIVAATYNGRAPDNARRFEAMLDAAGDAPGDWRAGGLRYAVLGCGNSQW
ATYQAFPKRLDTFFAAAGARPLLARGEADGNADFDQAAEQWLAALWSALAQQQPHAPGPA
PGAEGAPPLAVEVALRGIDEIRASVLPSNTQPFTVLANRELVSDPAGLWDFAVEAPRTST
RDIRLRLPEGARYAAGDHLAVYPQNRPALVHDLCERLDLDPEAVVTLSAPRAGAARGLPL
GQPLPLRALLTHFVELQDVVSRQTLRLLAQRTRCPFTRQSLERLGSDDAAQGYAAQVAAH
RLGLPDLLARFPAIELDLAGLLACVVPMRPRFYSIASSPLVSPDVATLTVGTVLAPALSG
RGQFRGVASNWLQGLAPGATVAAAIRTPNPPFAPEADPAQAMILIGPGTGIAPFRGFLEE

RAAQQAAGQAVAPVELYFGCRHPQHDWLYRDEMLAWQAQGVARLHVAHSATGAPARFVQD
LLWQQRAELWRQLSAGAVAYLCGDGRQMAPAVRKTLMDIAVAEGGLDGDAASAWFAGLVA
EGRYRQDVFN

>BKK80_00460|KEGG|CYP102E5|[Cupriavidus malaysiensis]cup|BP

MTQAATSSSQAPVAGRPAASLQPIPRDPGWPLVGNILQIKPGAFAQHLMARARAHEGIFE
LDFAGRRVPFVTSAALAAELADAARFRKFIGPPLSFLRDVAGDGLFTAHTDEANWGRAHR
ILMPAFSQRAMKGYFDVMLRVAMRLVDKWDRQGPDADIAVSDDMTRLTLDTIALSGFGYD
FDSFASQQLHPFIDAMVGALEEAMGKLTRFPLQDRFMHAAHRKFAEDTRFMRELVDDVIR
QRRQSPTPARDLLNLMLEARDPETDQRLDDVNIRNQVITFLIAGHETTSGLLTFALYELM
RHPGVLAQAYAEVDAVLPGDAPPAYADLARLQVLDRVLKETLRLWPTAPAFSVAPFEDTV
IGGRYRIHKDRRVAVVLTALHRDPAVWDDPERFDIDRFLPENEARIHPHAYKPFGNGERA
CIGRQFALTEAKLALALMLRHFQLHDPHDYQFRIKETLTLKPDHFLLRARRRRPHERIVV
AAPASGAVTAAAARPVVAAGGETLAVLCASSLGTARELAEQVQAGALAAGYEATLHDLDD
ALGKLPTQGLAVIVAATYNGRAPDNARRFEAMLDAAGDAPGDWRAGGLRYAVLGCGNSQW
ATYQAFPKRLDTFFAAAGARPLLARGEADGNADFDQAAEQWLAALWSALAQQQPHAPGPA
PGAEGAPPLAVEVALRGIDEIRASVLPSNTQPFTVLANRELVSDPAGLWDFAVEAPRTST
RDIRLRLPEGARYAAGDHLAVYPQNRPALVHDLCERLDLDPEAVVTLSAPRAGAARGLPL
GQPLPLRALLTHFVELQDVVSRQTLRLLAQRTRCPFTRQSLERLGSDDAAQGYAAQVAAH
RLGLPDLLARFPAIELDLAGLLACVVPMRPRFYSIASSPLVSPDVATLTVGTVLAPALSG
RGQFRGVASNWLQGLAPGATVAAAIRTPNPPFAPEADPAQAMILIGPGTGIAPFRGFLEE
RAAQQAAGQAVAPVELYFGCRHPQHDWLYRDEMLAWQAQGVARLHVAHSATGAPARFVQD
LLWQQRAELWRQLSAGAVVYLCGDGRQMAPAVRKTLMDIAVAEGGLDGDAASAWFAGLVA
EGRYRQDVFN

>BKK79_11700|KEGG|CYP102E5|[Cupriavidus sp.]cuu|BP

MTQAATSSSQAPVAGRPAASLQPIPRDPGWPLVGNILQIKPGAFAQHLMARARAHEGIFE
LDFAGRRVPFVTSAALAAELADAARFRKFIGPPLSFLRDVAGDGLFTAHTDEANWGRAHR
ILMPAFSQRAMKGYFDVMLRVAMRLVDKWDRQGPDADIAVSDDMTRLTLDTIALSGFGYD
FDSFASQQLHPFIDAMVGALEEAMGKLTRFPLQDRFMHAAHRKFAEDTRFMRELVDDVIR

QRRQSPTPARDLLNLMLEARDPETDQRLDDVNIRNQVITFLIAGHETTSGLLTFALYELM
RHPGVLAQAYAEVDAVLPGDAPPAYADLARLQVLDRVLKETLRLWPTAPAFSVAPFEDTV
IGGRYRIHKDRRVAVVLTALHRDPAVWDDPERFDIDRFLPENEARIHPHAYKPFGNGERA
CIGRQFALTEAKLALALMLRHFQLHDPHDYQFRIKETLTLKPDHFLLRARRRRPHERIVV
AAPASGAVTAAAARPVVAAGGETLAVLCASSLGTARELAEQVQAGALAAGYEATLHDLDD
ALGKLPTQGLAVIVAATYNGRAPDNARRFEAMLDAAGDAPGDWRAGGLRYAVLGCGNSQW
ATYQAFPKRLDTFFAAAGARPLLARGEADGNADFDQAAEQWLAALWSALAQQQPHAPGPA
PGAEGAPPLAVEVALRGIDEIRASVLPSNTQPFTVLANRELVSDPAGLWDFAVEPPRTST
RDIRLRLPEGARYAAGDHLAVYPQNRPALVHDLCERLDLDPEAVVTLSAPRAGAARGLPL
GQPLPLRALLTHFVELQDVVSRQTLRLLAQRTRCPFTRQSLERLGSDDAAQGYAAQVAAH
RLGLPDLLARFPAIELDLAGLLACVVPMRPRFYSIASSPLVSPDVATLTVGTVLAPALSG
RGQFRGVASNWLQGLAPGATVAAAIRTPNPPFAPEADPAQAMILIGPGTGIAPFRGFLEE
RAAQQAAGQAVAPVELYFGCRHPQHDWLYRDEMLAWQAQGVARLHVAHSATGAPARFVQD
LLWQQRAELWRQLSAGAVAYLCGDGRQMAPAVRKTLMDIAVAEGGLDGDAASAWFAGLVA
EGRYRQDVFN

>BKK79_19265|KEGG|CYP212A18|[Cupriavidus sp.]cuu|BP

MPTRLPSSCPVHASAPSRPASPAPQDAWPPGPAPGLTGWGLLRHMSRDLLGALAGWKQAH
GDIVHLAIWPEHQVIVSDPELARELLVARHADLVRWERAMHVFSGLQGRSVLIVEGEAWH
GKRTALQPAFAPRPVQSLVPTIVAACDKALAQWPTRAPAWPVESALTGLAMDIIMQTLFS
SGIGDDARLAEHAVHAALVAADKEFYWPASWPDWMPWKRRKRNAVAALRALVERHLLARL
RLPQDAWPDDLLTRLLGLHRQDATAWPLQAVRDECMTAFLAGHETSAATLAWWAWCMAAN
PAAQATARAEVERVLQGRAPGADTLPGLTYLTQTVKETLRLYPTAPVLISRRSKRALTLG
PWTFPERTLFMLPVQLMHHDARWFDEPLAFRPERFAAGAPDFPRGAYLPFGAGPRVCLGQ
HLAMAELTVVAAMVLQRFSLSLPPDAEQLDTVLHITLRPAQPMRLGIAPAAATAPAAAAR
PTVRG

>EHF44_06115|KEGG|CYP102E1|[Cupriavidus pauculus]cpau|BP

MPPAMSSVPAPDALEPIPRDPGWPIVGNLFQITPGEVGQHLLARSRHHQGIFELDFAGRR
VPFVSSVALAAEVCDPARFRKIIGPPLSYLRDMAGDGLFTAHSDEPNWGCAHRILMPAFS

QRAMKGYFDVMLRVANRLVDKWDRNGPDADIAVADDMTRLTLDTIALAGFGYDFRSFASD
APDPFIVAMVGALEEAMRKLTRLPIQDRFMGRAHRRFADDVATMRNLVDDVIRQRRATPR
PGMDLLNLMLEARDPETDRQLDDANIRNQVITFLIAGHETTSGLLTFTLYELLRNPAVLA
QAYAEVDAVLPGDAPPVHADLARMPVLDRVLKETLRLWPTAPAFAVAPFEDVVLGGRYRL
RQNRRISVVLTALHRDPAVWDNPERFDIDRFLPEHEAKLPAHAYLPFGHGERACIGRQFA
LTEAKLALALMLRNFAFHDPYDYQFRLKETLTIKPDNLVLRARRRPHERIAAPAATPAV
TDAAQADVRGHGQPMAVLCASSLGTARELAEQIHGGAVAAGFDATLADLDDVADALPTSG
LLVVVAATYNGHAPDSGRRFEAMLDAGGADGYRADGLRLALLGCGNSQWATYQAFPRRVF
DVFTQAGAVPLLPRGEADGNGDFDQAAERWLAALWQALQADGAGAGGIGVDVDVRSVAAI
RADTLPAGTRTFTVLANEELVGDPAGLWDFAVEPPRTSTRAIRLQLPPGMTYETGDHVAV
WPRNADALVDDLCARLDLDADAMVTLSAPHGTGRGLPIGQPLPLRQLLTHFVELQDVVSR
QTLRALAQATRCPATRAALERLASDDEADGYATQVAARRLNLCDVLAAHPAVTPTLPQLL
ACTVPMRPRFYSIASSPRVSPDVATLLVGTVWAPALSGRGQFRGVASTWLQGLAPGATVS
ASIRTPNPPFAPLADPSAPMLLVGPGTGIAPFRGFLEERAAEQAAGQRVTPVQLYFGCRH
PRHDWLFHDEIARWAAAGVAEVHTAYSVVPGAARYVQDLLWQRRAQVWTQLQAGATVYVC
GDGRRMAPAVRQTLIDIGADQGGMAPEGASDWFAALVRQGRYRQDVFN

>EHF44_13900|KEGG|CYP105CW2|[Cupriavidus pauculus]cpau|BP

MTESPAPTPSSNPSSAVEGRCPFDAAANEPKSACPFHAEGDSGGIPDFPPKRVDPLAPPP
VFFQMQQKGELAQGKLWDGKIAWLVTRYDDVRAVLSDSRFSSDVSHPGFPSSSAAVKVVR
SSNRTFITMDEPKHSEQRRMLTSEFTIRKIENLRPRLQAIVDKLLEDFAMGPQPSDLVSA
FTLATPALAISELLGVPYDDHDFFQEQAMIMTSSTATREEASAANQALCEYLRELVAKRS
ESPGEDILSRLIVNHVRKGDITETDVVSLGRLLLIAGHETTANTTSMGVLFLLQRPDIWE
ELRQNPSLVPNAVEEMLRFLDVIQSGTRRVALEDVVVNGQLIRAGDPVVVLSISANRDQE
QFKDPHVFDLRRDARTQIAFGYGPHQCMGQVLARVEMQIMFTALLKRLPNLRLAVPIESL
AFKSDSLMYGVRALPVSW

>EHF44_13985|KEGG|CYP105CW1|[Cupriavidus pauculus]cpau|BP

MHAGGDSGIPDFPPERVDPLSPPPVYFQMQQGSGLGQARLWDGKIAWLITRYDDVRSVLS
DPRFSSDITNPGYPTVSAAMKVARGSNRTFITMDAPKHAEHRRMLTGEFSIRKIEALRPR

IQAIVDKLLDDFATKPQPSDLVSAFTLATPALAISELLGVPYEDHDFFQELAMVLTSSSA
TLEEAIAANHELCEVYLKGLVAKRSENPGEDILSRLIVNHVRKGDITETDVVSLARLLLI
AGHETTANTTAMGVLFLLQRPDIWNELRQDTSLVPNAVEEILRYLDVTHSGKRRVATEDI
VVNGQMIQAGDPVVVLSVSANRDSSKFEDPNVFDLRRDSRTQVSFGYGPHQCIGQPLARL
EMQIMFTALLERFPNLELAVPVESLEFKGDSLMYGVKELPVRW

>EHF44_20660|KEGG|CYP229F18|[Cupriavidus pauculus]cpau|BP

MQPADPVAAVTHPDPYPYYRALARTRPFHFDQGLKLWVAAGAEAVAAVLAHPDARVRPPG
QPVPPALAGGPAGDLFGRLVRMNDGPAHAALKPAILRGLAGLDLGLVRVRAHALAAALPR
LGTDDAHEAMQWMFTLPVLTVADLLGLPLARDADGHVLTRIAGFVTTFAAAMSPLASPAD
VAAGAAAAQWLTRCVADQADHADPAPPDGLLPTLWRDAAHVEPAARVANAVGLMIQACEA
TAGLIGNTLVHLGRDAAGRPVEDFDAIDATVGRVADLDPPVQNTRRFLAADAVVCGQPLR
AGDAVLVLLAAAAHDPSAPSGGRPWTFGAGRHGCPGDALARVLAACTVQALLARGVQPAR
LAAGVRYRPSVNARIPQFTSQP

>EHF44_24585|KEGG|CYP116B300|[Cupriavidus pauculus]cpau|BP

MTQPTPPAPRAGCPIDHTALRAPNGCPVSHNAAAFDPFEDGYQQDPPDYVRWSREQEPVF
YSPRLGYWVVTRYDDIKAIFRDNLTFSPAIALEKITPTGDAANAVLASYGYAMNRTLVNE
DEPAHMPRRRALMEPFTPAELMHHEPLVRRLAREYVDRFIDDGRADLVDQMLWEVPLTVA
LHFLGVPEEDMDLLRQYSIAHTVNTWGRPRPEEQVAVAHAVGNFWQLAGRILDKMRADPS
GPGWMQYGIRKQQELPDVVTDSYLHSMMMAGIVAAHETTANASANAIKLLLQHPDVWREI
CDDPALIPNAVEECLRHNGSVAAWRRVVTQDTEVGGIPLAAGSKLLIMTASANHDERHFA
DADLFDIHRDNASDQLTFGYGAHQCMGKNLARMEMQVFLEELTSRLPHLRLAEQTFTYVP
NTSFRGPEHLWVEWDPARNPERTNPAVLAPRDAVRIGEPTGNIGRTLVVASVTPAAQDVL
RIRLVAPDGRPLPRWSPGSHIDVECGSTGISRQYSLCGDPADAGAFEIAVLREAESRGGS
AWIHASLRAGNRLKVRGPRNHFRLDEGCRRALFIAGGIGITPVSAMARRARELGIDYAIH
YCGRSRAAMAMLDELAALHGSRLHIHARDEGSRADLAAIVGAPDGITQVYACGPARMIEG
LEAACAAWPPDTLRVEHFSSQLGTLDPSKEQPFTVELKDSGLTLEVPADQTLLTALRCAN
IDVQSDCEEGLCGSCEVRVLAGDIDHRDVVLTRTEREANDRMMSCCSRAKTGGRIVLAL

>EHF44_26030|KEGG|CYP133C12|[Cupriavidus pauculus]cpau|BP

MKFESFATPAFFENPYPLYEQIRAAGPLLPLGPNVWVTGRFHVIDALLRDRRMGKTYLQS
VQARYGEAATQAPVFQAISRTFLMINPPAHTRLRALLMQAFNRRQVEGLARIVDTTTRSL
LDRVGHRTEFDVVADFALPLPVEIICRLLDIPVEDGVRLGSAASRMATALDLAPLDERQL
ADANDAALTLEAYFRDVVARRRTQPGDDLISSLIGVEQDGATLTEAEIISNVILLFVAGH
ETTSNMIGNTLIGLFRAPEALQQLKAGLSDPAMVSRAVAECLRHDGAVQMVVRTAFEDIA
LDDVVVPRGSIVFMLLGAANRDPAVFPQPDQLDLSRADAPSLSFGGGIHYCLGARLATLE
LESAITGLLTRFPNLQPVDLAGLSWHRRNNLRGVQALPVRTGA

>E0W60_00125|KEGG|CYP1098D1|[Cupriavidus oxalaticus]cox|BP

MNFQPPYPKPHTRKSSFLLRFLRGWNSWIDVLFERSYSMKMGKISQPGMDIFMVNDADWV
RKILGDTHEYPKHKLMHRMLEPLLGTSIFTTNGKVWERQRRLVEPAFAQARLKLVFSLMA
DSVAGMTRRLDAVADGRSYEVDGEMTYVTADIIFRTILSENLEESAAKEIYDAFLEFQHH
AQRAMILMIYRLPPVISAWRSKRAAKKIRVILSEIIARRLAERDSGQGSAKQDILAGMMD
AVDPVNGDRFTYEELVDQVCMLFLAGHETSASALTWALYLISQCPHLQDKMLAEIQEAVG
DRDFELSDIKSLPAVGNVFRETLRLYPPVGFFVREAAQSQCIRDKSVKEGSPILISPWLL
HRHRTLWERPDEFDPDRFDTQAGKESSKCAYIPFSKGPRVCIGAAYATQEAVLILASIVR
RYRVEADPGHVPKVVGRVTIRSGNGVRVRLHRR

>E0W60_00515|KEGG|CYP116B161|[Cupriavidus oxalaticus]cox|BP

MAIPSPDTARGGCPVDHSAFTAPNGCPVSHKAAEFDPFGDGYQQDPPEYVRWSREQEPVF
YSPKLGYWVVTRYDDIKAIFRDNITFSPSIALEKITPTGDEANAVLASYGYAMNRTLVNE
DEPAHMPRRRALMDPFTPAELAHHEPMVRRLAREYVDQFIDDGRADLVDQMLWEVPLAVA
LHFLGVPEEDMDLLRQYSIAHTVNTWGRPRPDEQVAVAHAVGNFWQLAGRILDKMREDPS
GPGWMQYGLRKQKALPEVVTDSYLHSMMMAGIVAAHETTANASANAIKLLLQHPDTWREI
CEDPSLIPNAVEECLRHNGSVAAWRRLVTRDTEVGGISLATGSKLLIVTSSANHDERHFA
DADLFDIRRDNASEQLTFGYGSHQCMGKNLARMEMQIFLEELTRRLPHMRLAEQSFTYVP
NTSFRGPEHLLVEWDPAQNPERRDPALLAQRQPVRIGEPSAHTSARTVVVESIAPAAAGV
VRLRLAAPDGKPLPRWAPGSHIDVECGDTGLSRQYSLCGDPDDAAALEIAVLREPDSRGG
SAWVHGSVTAGDRLRIRGPRNHFRFDEGCARAIFIAGGIGITPVSAMARRARQLGIDYEI
HYCGRSRGSMAMLDALQALHGARLHVYAGDEGRRADFAAMLATPQPRTHVYACGPQRLLD

ALADACAAWPEDALRVEHFVSRLGTLDASKEQPFTVELKDSGLVMEVPAGQTLLAALRGA
NIDVQSDCEEGLCGSCEVRVLAGQVDHRDVVLTRTERDANDRMMACCSRAAGGCRLVLEL

>E0W60_05550|KEGG|CYP229F19|[Cupriavidus oxalaticus]cox|BP

MLPAAESHANDANHALDPLSAVTHPDPYPYYRELATHRPFFRDQRLGLWVAAGAREVATV
LSHPDCRVRPPAQPVPPALAGTAAGDLFGRLIRMNDGAAHAPLKDLLMPLLAAIDPAAAA
RRAAALAELMDAGTAWSAERINGVLSTLPVVTLADLLGLPVAVDRATAAAVAAHVAAFAA
AQSPLADANAVQAGAVAAQWLGNWLANAVPAAGPLPSLQQAACAAGIDAQAVAANTIGLL
VQACEATAALAGNSLLHLGRGKALPGISPDQLVTEVAVSDPPVQNTRRFLAADATLCGHD
VKAGDVVLVLLAAASCGGMADGERPWTFGHGRHACPGDRLAQALAGATVTALLARGAAPA
VLARAFRYRPSLNARIPHFC

>E0W60_09205|KEGG|CYP102E6|[Cupriavidus oxalaticus]cox|BP

MPQPTVSPGTEALAQPAHPAAPTAPEPIPRDPGWPLVGNLLQIKPGTVGQHLLARSRHHD
GIFELDFAGRCVPFVTSVALAAEVCDAARFRKYIGPPLSYLRGMAGDGLFTAHTDEANWG
KAHRILMPAFSQRAMKGYFEVMLRVANRLVDKWDRQGPDADIAVADDMTRLTLDTIALSG
FGYDFDSFASAQLHPFIEAMVGALGEAMSRLTRLGLQDRFMHAAHRKFDEDTRFMRELVD
DVIRRRRAADEGGRQGTAMDLLGLMLEARDPDTDQRLDDENIRNQVITFLIAGHETTSGL
LTFALYELLRNPGVMAQAYAEVDAVLPGDAAPVYADLARLPVLDRVLKETLRLWPTAPAF
AVAPYADTLLGGRYLIRKDRRVSVVLTALHRDPRVWADPERFDIDRFLPDNEARLPRHAY
MPFGNGERACIGRQFALTEAKLALALMLRNFQFTDAHDYQFRIKETLTIKPDGFTVRARR
RRPHERIVGMPVAGAEQAPPAGAEVRGAGRALAVLCGSSLGTARELAEQVHAGALAAGFD
ATLRDLDEIADALPASGLAVIVAATYNGRAPDSARRFEDLLNSGAARDYRAPDLRVAVLG
CGNSQWPTYQAFPRRVFEFFASAGATPLLPRGEADGNGDFDQAAERWLALLWQALQGGDA
ASALPQVPAVQVQLRGVAEIRATTLPPNAQGFTVLSNTELVNDPDGLWDFAREAPRTSTR
DIRLRLPEGVRYHTGDHLAVYPQNQPETVTAICERIDVDPDAIVTLSAAPGAARGLPVGE
ALSARQLLTHFVELQDVVSRQTLRSLAQATPCPFTRQSLQQLASDDEAAGYAAQLVQRRL
GLGDILDRFPAIGLDLAGLLACTVPMRPRFYSIASSPLVSPDVATITVGTVCAPALSGRG

LFRGVASTWLQGLAPGAVVAGVIRTPHPPFAPEAPDVPMILVGPGTGIAPFRGFLEERAA
QQAAGEAVRPVQLYYGCRHPAHDWLYRDDVARWQEQGVAEVHAAYSVVDGAPCFVQDLLW
QRQADVWAQLNAGAVFYVCGDGRHMAPAVRQTLIRIAAEQGGMTEAAASDWLAALVAAGR
YRQDVFN

>BMAA0023|KEGG|CYP1488A1|[Burkholderia mallei]bma|BP

MNGLPLSLPRVDSTDSLFADPLAFLARARAQCGDVFVLREHGPIFSRASDCSGVIAVFGE
HRLRQVLTETDTFALPMSAAAKMALPKNLVNLNRGLHSMREPEHGRHKRLLTGTANRALF
DAHRSGMQTDLSRFCEMLNGDGRISVVSRMRELTAKLASRLFLGPQCEEDAELTFLLSAY
FTLRREASSPNAHDPLQYRDALIGAGRQLDRALRARIRRYRKAPAGECAGLLQRLATAGQ
PGSPALSEDEIVGHANVMFVSSTEPVAISLAWLLLVLSQLPDLRRALRAESAARASSPAS
PYDAPLLERVINETLRLLTPNALMVRATTQAVSLQGVALPARCEIVVCPFLVHREANAFA
RPHAFLPSRWETARPSPYEYFPFGAGGHFCAGRNLALSLIGEVLSTLLSRFDFVLDIEQF
IDWRIHIMLMPKGDPTLTAHPVDERRDTPSPKWRGPVAELFHFAPGLS

>BMAA1669|KEGG|CYP116B23|[Burkholderia mallei]bma|BP

MTTHSASTPPALPAAQCPFRPVSDDAAAHPAGCPASAGAAAFDPFDDAYQQDPPEYVRWA
REREPVFYSPKLGYWVVARYDDIKTIFRDNRTFSPSIALEKITPTGPDANAALASYGYAM
GRTLVNEDEPTHMARRRLLIEPFTPDALRAHEPMVRRLAREYVDRFVDDGRADLVDQMLW
EVPLTVALHFLGVPEADMDTLRAYSIAHTVNTWGRPRPDEQVAVAHAVGQFWQYAGKVLE
QMREDPSGPGWMQFGIRMQREHPDIVTDSYLHSMMMAGIVAAHETTANATANALKLLLEH
PRAWRELCDDPALIPNAVDECLRHSGSIVAWRRIATRDATVGGVAIPAGAKLLIVTSSAN
RDERRFADPDLFDIHRDNPADHLTFGYGSHQCMGKHLARMEMQIFIDELSRRLPHMRLAE
QRFTYVPNTSFRGPEHLWVEWEPAENPQRRGHGARRCALAVRIGESNARRAGRALRVDSV
MPVAERVLRVRLVAPDGAPLPRWSAGAHIDVECGDTGLSRQYSLCGDPAERGVYEIAVLR
EPASRGGSAWMHERLRPGMRVKIRGPRNHFRLDGHAARAILIAGGIGITPIGAIAREARA
RGIDYALHYCGRSRRTMAFVDELAALHGERLRLHVSDEGTRHDFSALDVAAGTQVYACGP
ARMLDALAACAAHWPAHALRVEHFAAAAPRVDPRTDQPFEVELKDSGLTLTVPANRTLLA
TLRAANVDVQSDCEEGLCGSCEVRVLAGEVDHRDSVLSADERALNTRMMACCSRARHGRL
VLEL

>BMAA2088|KEGG|CYP1013A3|[Burkholderia mallei]bma|BP

MSKHRQAPGPRGGLVMGNLAAYKRNPITMLLRLHQQYGDVARNRLGPFVTHALAHPDHIQ
YVLQENHRNYVRGRFYDNFKMFFGDGLLTTDGEFWRRHRRVVQPLFHKKQVDAHTAAVGD
AALALAYRWSALPPGKALDVVEEMMHLSLRMLGLMVFNTDVSSHAEAAGPAVRFGIEAMM
PQGNLNDFIPRWAPTRFNRRIAHARRAIDTIVAKIIADHREARCEPSDVISLLLNARDPD
TGAPMTQQEVHDEVMTVFLAGHETTGAGLAWALYALAQHPAVLRQLRDELDARLGGRAPT
VQDFEQLPYLSQVVDEVLRVYPPIWGFTRDLVEDDEIGGYRIPARSSVFMSPYVTHRHPA
FWRNPDAFDPENFASDAPARHRFVYFPFGGGMRKCIGFQTALLQMRVLVAVVAQHFDLNA
LPGHPIELGATISLRPVHGIRLIVKPRERQQSHLARVREHESARALRPLGDTATTAGAAC
PMTAAREPGASAAALAAVEAGALDAAHRGGAAAAAAGEPCPPRDPAEAAPLPLAAAPAPA
SPAVDADASARGVPAWRFTWRPVPVDPVPDEPSPALSGRRIALVNGRGGTAERVAAALAR
TCAKVSVFAPAASADHAEAARVFVNEAGPLDGIVDLGLEAPFALDDAGAWEAPMRRTVEL
LKACYGDWAAEDDTSRLFYVALTWMDGQMGYGETAGAQPLGGLWAGLAKTLPQELPNCNV
RVLDIAPDETGRVDRLVVNELYRWGLFEVGYRGGVRYTLHAKRNELPPHAVDARTTLGAG
DTVLFSGGARGIGLLCARSLAERHGCTVIVTGRETPPDGGEPWMALDEPGFKHYGLEQLR
AATPEHPPKAIRAALQRLKRRRELRTALDEMAALGLPVHYRVCDVTDRAAVRALCDELGD
SLRMVIHNAGVDRPVRLIQKRAEDFADTVRTKVLGFANLCDAVRARPRLVQFCNVGSLTG
RWGGMTGETDYAAANEALARLGLWAARHAFSPACGVKTLVWPTWEGVGMITNFAVTQRYV
SPMNVDEGVRHWLRELAAPGSGEVMFMGAVGRAPTPMQIKGFVPIRELPNIGELVTRRHH
VGEPVRFRPFKRFESRYAIDPAVAPYAHAFRFNGRAVLPASLVLEHACAVGDWVMPPTGE
PRELSEMTNVVLRLDALPDVPAGGAPVELASEAAGYMIGDVWCVDVRCSDARTQAELLSM
TLVYRPQPEQIGAAFARAPDTTPEPAPLTSAPHAAWNGELLPAGDWSVPDDASAGIVRIG
RVPSAAVLSLWALPFPLELRLPVNHVEHVLRVLLAEWGAARADAPAVWRIGGAKLARLPA
ASADGVVEYAAGRFAVLDARGYVLLRIDDARVDGEPAQAALPETGALADPLTA

>BMASAVP1_1115|KEGG|CYP1013A3|[Burkholderia mallei]bmv|BP

MSKHRQAPGPRGGLVMGNLAAYKRNPITMLLRLHQQYGDVARNRLGPFVTHALAHPDHIQ
YVLQENHRNYVRGRFYDNFKMFFGDGLLTTDGEFWRRHRRVVQPLFHKKQVDAHTAAVGD
AALALAYRWSALPPGKALDVVEEMMHLSLRMLGLMVFNTDVSSHAEAAGPAVRFGIEAMM
PQGNLNDFIPRWAPTRFNRRIAHARRAIDTIVAKIIADHREARCEPSDVISLLLNARDPD

TGAPMTQQEVHDEVMTVFLAGHETTGAGLAWALYALAQHPAVLRQLRDELDARLGGRAPT
VQDFEQLPYLSQVVDEVLRVYPPIWGFTRDLVEDDEIGGYRIPARSSVFMSPYVTHRHPA
FWRNPDAFDPENFASDAPARHRFVYFPFGGGMRKCIGFQTALLQMRVLVAVVAQHFDLNA
LPGHPIELGATISLRPVHGIRLIVKPRERQQSHLARVREHESARALRPLGDTATTAGAAC
PMTAAREPGASAAALAAVEAGALDAAHRGGAAAAAAGEPCPPRDPAEAAPLPLAAAPAPA
SPAVDADASARGVPAWRFTWRPVPVDPVPDEPSPALSGRRIALVNGRGGTAERVAAALAR
TCAKVSVFAPAASADHAEAARVFVNEAGPLDGIVDLGLEAPFALDDAGAWEAPMRRTVEL
LKACYGDWAAEDDTSRLFYVALTWMDGQMGYGETAGAQPLGGLWAGLAKTLPQELPNCNV
RVLDIAPDETGRVDRLVVNELYRWGLFEVGYRGGVRYTLHAKRNELPPHAVDARTTLGAG
DTVLFSGGARGIGLLCARSLAERHGCTVIVTGRETPPDGGEPWMALDEPGFKHYGLEQLR
AATPEHPPKAIRAALQRLKRRRELRTALDEMAALGLPVHYRVCDVTDRAAVRALCDELGD
SLRMVIHNAGVDRPVRLIQKRAEDFADTVRTKVLGFANLCDAVRARPRLVQFCNVGSLTG
RWGGMTGETDYAAANEALARLGLWAARHAFSPACGVKTLVWPTWEGVGMITNFAVTQRYV
SPMNVDEGVRHWLRELAAPGSGEVMFMGAVGRAPTPMQIKGFVPIRELPNIGELVTRRHH
VGEPVRFRPFKRFESRYAIDPAVAPYAHAFRFNGRAVLPASLVLEHACAVGDWVMPPTGE
PRELSEMTNVVLRLDALPDVPAGGAPVELASEAAGYMIGDVWCVDVRCSDARTQAELLSM
TLVYRPQPEQIGAAFARAPDTTPEPAPLTSAPHAAWNGELLPAGDWSVPDDASAGIVRIG
RVPSAAVLSLWALPFPLELRLPVNHVEHVLRVLLAEWGAARADAPAVWRIGGAKLARLPA
ASADGVVEYAAGRFAVLDARGYVLLRIDDARVDGEPAQAALSETGALADPLTA

>BMASAVP1_1176|KEGG|CYP1488A1|[Burkholderia mallei]bmv|BP

MNGLPLSLPRVDSTDSLFADPLAFLARARAQCGDVFVLREHGPIFSRASDCSGVIAVFGE
HRLRQVLTETDTFALPMSAAAKMALPKNLVNLNRGLHSMREPEHGRHKRLLTGTANRALF
DAHRSGMQTDLSRFCEMLNGDGRISVVSRMRELTAKLASRLFLGPQCEEDAELTFLLSAY
FTLRREASSPNAHDPLQYRDALIGAGRQLDRALRARIRRYRKAPAGECAGLLQRLATAGQ
PGSPALSEDEIVGHANVMFVSSTEPVAISLAWLLLVLSQLPDLRRALRAESAARASSPAS
PYDAPLLERVINETLRLLTPNALMVRATTQAVSLQGVALPARCEIVVCPFLVHREANAFA
RPHAFLPSRWETARPSPYEYFPFGAGGHFCAGRNLALSLIGEVLSTLLSRFDFVLDIEQF
IDWRIHIMLMPKGDPTLTAHPVDERRDTPSPKWRGPVAELFHFAPGLS

>BMASAVP1_1707|KEGG|CYP116B23|[Burkholderia mallei]bmv|BP

MTTHSASTPPALPAAQCPFRPVSDDAAAHPAGCPASAGAAAFDPFDDAYQQDPPEYVRWA
REREPVFYSPKLGYWVVARYDDIKTIFRDNRTFSPSIALEKITPTGPDANAALASYGYAM
GRTLVNEDEPTHMARRRLLIEPFTPDALRAHEPMVRRLAREYVDRFVDDGRADLVDQMLW
EVPLTVALHFLGVPEADMDTLRAYSIAHTVNTWGRPRPDEQVAVAHAVGQFWQYAGKVLE
QMREDPSGPGWMQFGIRMQREHPDIVTDSYLHSMMMAGIVAAHETTANATANALKLLLEH
PRAWRELCDDPALIPNAVDECLRHSGSIVAWRRIATRDATVGGVAIPAGAKLLIVTSSAN
RDERRFADPDLFDIHRDNPADHLTFGYGSHQCMGKHLARMEMQIFIDELSRRLPHMRLAE
QRFTYVPNTSFRGPEHLWVEWEPAENPQRRGHGARRCALAVRIGESNARRAGRALRVDSV
MPVAERVLRVRLVAPDGAPLPRWSAGAHIDVECGDTGLSRQYSLCGDPAERGVYEIAVLR
EPASRGGSAWMHERLRPGMRVKIRGPRNHFRLDGHAARAILIAGGIGITPIGAIAREARA
RGIDYALHYCGRSRRTMAFVDELAALHGERLRLHVSDEGTRHDFSALDVAAGTQVYACGP
ARMLDALAACAAHWPAHALRVEHFAAAAPRVDPRTDQPFEVELKDSGLTLTVPANRTLLA
TLRAANVDVQSDCEEGLCGSCEVRVLAGEVDHRDSVLSADERALNTRMMACCSRARHGRL
VLEL

>BMA10229_1393|KEGG|CYP1013A3|[Burkholderia mallei]bml|BP

MSKHRQAPGPRGGLVMGNLAAYKRNPITMLLRLHQQYGDVARNRLGPFVTHALAHPDHIQ
YVLQENHRNYVRGRFYDNFKMFFGDGLLTTDGEFWRRHRRVVQPLFHKKQVDAHTAAVGD
AALALAYRWSALPPGKALDVVEEMMHLSLRMLGLMVFNTDVSSHAEAAGPAVRFGIEAMM
PQGNLNDFIPRWAPTRFNRRIAHARRAIDTIVAKIIADHREARCEPSDVISLLLNARDPD
TGAPMTQQEVHDEVMTVFLAGHETTGAGLAWALYALAQHPAVLRQLRDELDARLGGRAPT
VQDFEQLPYLSQVVDEVLRVYPPIWGFTRDLVEDDEIGGYRIPARSSVFMSPYVTHRHPA
FWRNPDAFDPENFASDAPARHRFVYFPFGGGMRKCIGFQTALLQMRVLVAVVAQHFDLNA
LPGHPIELGATISLRPVHGIRLIVKPRERQQSHLARVREHESARALRPLGDTATTAGAAC
PMTAAREPGASAAALAAVEAGALDAAHRGGAAAAAAGEPCPPRDPAEAAPLPLAAAPAPA
SPAVDADASARGVPAWRFTWRPVPVDPVPDEPSPALSGRRIALVNGRGGTAERVAAALAR

TCAKVSVFAPAASADHAEAARVFVNEAGPLDGIVDLGLEAPFALDDAGAWEAPMRRTVEL
LKACYGDWAAEDDTSRLFYVALTWMDGQMGYGETAGAQPLGGLWAGLAKTLPQELPNCNV
RVLDIAPDETGRVDRLVVNELYRWGLFEVGYRGGVRYTLHAKRNELPPHAVDARTTLGAG
DTVLFSGGARGIGLLCARSLAERHGCTVIVTGRETPPDGGEPWMALDEPGFKHYGLEQLR
AATPEHPPKAIRAALQRLKRRRELRTALDEMAALGLPVHYRVCDVTDRAAVRALCDELGD
SLRMVIHNAGVDRPVRLIQKRAEDFADTVRTKVLGFANLCDAVRARPRLVQFCNVGSLTG
RWGGMTGETDYAAANEALARLGLWAARHAFSPACGVKTLVWPTWEGVGMITNFAVTQRYV
SPMNVDEGVRHWLRELAAPGSGEVMFMGAVGRAPTPMQIKGFVPIRELPNIGELVTRRHH
VGEPVRFRPFKRFESRYAIDPAVAPYAHAFRFNGRAVLPASLVLEHACAVGDWVMPPTGE
PRELSEMTNVVLRLDALPDVPAGGAPVELASEAAGYMIGDVWCVDVRCSDARTQAELLSM
TLVYRPQPEQIGAAFARAPDTTPEPAPLTSAPHAAWNGELLPAGDWSVPDDASAGIVRIG
RVPSAAVLSLWALPFPLELRLPVNHVEHVLRVLLAEWGAARADAPAVWRIGGAKLARLPA
ASADGVVEYAAGRFAVLDARGYVLLRIDDARVDGEPAQAALPETGALADPLTA

>BMA10229_1455|KEGG|CYP1488A1|[Burkholderia mallei]bml|BP

MLREHGPIFSRASDCSGVIAVFGEHRLRQVLTETDTFALPMSAAAKMALPKNLVNLNRGL
HSMREPEHGRHKRLLTGTANRALFDAHRSGMQTDLSRFCEMLNGDGRISVVSRMRELTAK
LASRLFLGPQCEEDAELTFLLSAYFTLRREASSPNAHDPLQYRDALIGAGRQLDRALRAR
IRRYRKAPAGECAGLLQRLATAGQPGSPALSEDEIVGHANVMFVSSTEPVAISLAWLLLV
LSQLPDLRRALRAESAARASSPASPYDAPLLERVINETLRLLTPNALMVRATTQAVSLQG
VALPARCEIVVCPFLVHREANAFARPHAFLPSRWETARPSPYEYFPFGAGGHFCAGRNLA
LSLIGEVLSTLLSRFDFVLDIEQFIDWRIHIMLMPKGDPTLTAHPVDERRDTPSPKWRGP
VAELFHFAPGLS

>BMA10229_1913|KEGG|CYP116B23|[Burkholderia mallei]bml|BP

MTTHSASTPPALPAAQCPFRPVSDDAAAHPAGCPASAGAAAFDPFDDAYQQDPPEYVRWA
REREPVFYSPKLGYWVVARYDDIKTIFRDNRTFSPSIALEKITPTGPDANAALASYGYAM
GRTLVNEDEPTHMARRRLLIEPFTPDALRAHEPMVRRLAREYVDRFVDDGRADLVDQMLW
EVPLTVALHFLGVPEADMDTLRAYSIAHTVNTWGRPRPDEQVAVAHAVGQFWQYAGKVLE
QMREDPSGPGWMQFGIRMQREHPDIVTDSYLHSMMMAGIVAAHETTANATANALKLLLEH

PRAWRELCDDPALIPNAVDECLRHSGSIVAWRRIATRDATVGGVAIPAGAKLLIVTSSAN
RDERRFADPDLFDIHRDNPADHLTFGYGSHQCMGKHLARMEMQIFIDELSRRLPHMRLAE
QRFTYVPNTSFRGPEHLWVEWEPAENPQRRGHGARRCALAVRIGESNARRAGRALRVDSV
MPVAERVLRVRLVAPDGAPLPRWSAGAHIDVECGDTGLSRQYSLCGDPAERGVYEIAVLR
EPASRGGSAWMHERLRPGMRVKIRGPRNHFRLDGHAARAILIAGGIGITPIGAIAREARA
RGIDYALHYCGRSRRTMAFVDELAALHGERLRLHVSDEGTRHDFSALDVAAGTQVYACGP
ARMLDALAACAAHWPAHALRVEHFAAAAPRVDPRTDQPFEVELKDSGLTLTVPANRTLLA
TLRAANVDVQSDCEEGLCGSCEVRVLAGEVDHRDSVLSADERALNTRMMACCSRARHGRL
VLEL

>BMA10247_A0029|KEGG|CYP1488A1|[Burkholderia mallei]bmn|BP

MNGLPLSLPRVDSTDSLFADPLAFLARARAQCGDVFVLREHGPIFSRASDCSGVIAVFGE
HRLRQVLTETDTFALPMSAAAKMALPKNLVNLNRGLHSMREPEHGRHKRLLTGTANRALF
DAHRSGMQTDLSRFCEMLNGDGRISVVSRMRELTAKLASRLFLGPQCEEDAELTFLLSAY
FTLRREASSPNAHDPLQYRDALIGAGRQLDRALRARIRRYRKAPAGECAGLLQRLATAGQ
PGSPALSEDEIVGHANVMFVSSTEPVAISLAWLLLVLSQLPDLRRALRAESAARASSPAS
PYDAPLLERVINETLRLLTPNALMVRATTQAVSLQGVALPARCEIVVCPFLVHREANAFA
RPHAFLPSRWETARPSPYEYFPFGAGGHFCAGRNLALSLIGEVLSTLLSRFDFVLDIEQF
IDWRIHIMLMPKGDPTLTAHPVDERRDTPSPKWRGPVAELFHFAPGLS

>BMA10247_A0588|KEGG|CYP116B23|[Burkholderia mallei]bmn|BP

MTTHSASTPPALPAAQCPFRPVSDDAAAHPAGCPASAGAAAFDPFDDAYQQDPPEYVRWA
REREPVFYSPKLGYWVVARYDDIKTIFRDNRTFSPSIALEKITPTGPDANAALASYGYAM
GRTLVNEDEPTHMARRRLLIEPFTPDALRAHEPMVRRLAREYVDRFVDDGRADLVDQMLW
EVPLTVALHFLGVPEADMDTLRAYSIAHTVNTWGRPRPDEQVAVAHAVGQFWQYAGKVLE
QMREDPSGPGWMQFGIRMQREHPDIVTDSYLHSMMMAGIVAAHETTANATANALKLLLEH
PRAWRELCDDPALIPNAVDECLRHSGSIVAWRRIATRDATVGGVAIPAGAKLLIVTSSAN
RDERRFADPDLFDIHRDNPADHLTFGYGSHQCMGKHLARMEMQIFIDELSRRLPHMRLAE
QRFTYVPNTSFRGPEHLWVEWEPAENPQRRGHGARRCALAVRIGESNARRAGRALRVDSV
MPVAERVLRVRLVAPDGAPLPRWSAGAHIDVECGDTGLSRQYSLCGDPAERGVYEIAVLR
EPASRGGSAWMHERLRPGMRVKIRGPRNHFRLDGHAARAILIAGGIGITPIGAIAREARA

RGIDYALHYCGRSRRTMAFVDELAALHGERLRLHVSDEGTRHDFSALDVAAGTQVYACGP
ARMLDALAACAAHWPAHALRVEHFAAAAPRVDPRTDQPFEVELKDSGLTLTVPANRTLLA
TLRAANVDVQSDCEEGLCGSCEVRVLAGEVDHRDSVLSADERALNTRMMACCSRARHGRL
VLEL

>BMA10247_A2379|KEGG|CYP1013A3|[Burkholderia mallei]bmn|BP
MSKHRQAPGPRGGLVMGNLAAYKRNPITMLLRLHQQYGDVARNRLGPFVTHALAHPDHIQ
YVLQENHRNYVRGRFYDNFKMFFGDGLLTTDGEFWRRHRRVVQPLFHKKQVDAHTAAVGD
AALALAYRWSALPPGKALDVVEEMMHLSLRMLGLMVFNTDVSSHAEAAGPAVRFGIEAMM
PQGNLNDFIPRWAPTRFNRRIAHARRAIDTIVAKIIADHREARCEPSDVISLLLNARDPD
TGAPMTQQEVHDEVMTVFLAGHETTGAGLAWALYALAQHPAVLRQLRDELDARLGGRAPT
VQDFEQLPYLSQVVDEVLRVYPPIWGFTRDLVEDDEIGGYRIPARSSVFMSPYVTHRHPA
FWRNPDAFDPENFASDAPARHRFVYFPFGGGMRKCIGFQTALLQMRVLVAVVAQHFDLNA
LPGHPIELGATISLRPVHGIRLIVKPRERQQSHLARVREHESARALRPLGDTATTAGAAC
PMTAAREPGASAAALAAVEAGALDAAHRGGAAAAAAGEPCPPRDPAEAAPLPLAAAPAPA
SPAVDADASARGVPAWRFTWRPVPVDPVPDEPSPALSGRRIALVNGRGGTAERVAAALAR
TCAKVSVFAPAASADHAEAARVFVNEAGPLDGIVDLGLEAPFALDDAGAWEAPMRRTVEL
LKACYGDWAAEDDTSRLFYVALTWMDGQMGYGETAGAQPLGGLWAGLAKTLPQELPNCNV
RVLDIAPDETGRVDRLVVNELYRWGLFEVGYRGGVRYTLHAKRNELPPHAVDARTTLGAG
DTVLFSGGARGIGLLCARSLAERHGCTVIVTGRETPPDGGEPWMALDEPGFKHYGLEQLR
AATPEHPPKAIRAALQRLKRRRELRTALDEMAALGLPVHYRVCDVTDRAAVRALCDELGD
SLRMVIHNAGVDRPVRLIQKRAEDFADTVRTKVLGFANLCDAVRARPRLVQFCNVGSLTG
RWGGMTGETDYAAANEALARLGLWAARHAFSPACGVKTLVWPTWEGVGMITNFAVTQRYV
SPMNVDEGVRHWLRELAAPGSGEVMFMGAVGRAPTPMQIKGFVPIRELPNIGELVTRRHH
VGEPVRFRPFKRFESRYAIDPAVAPYAHAFRFNGRAVLPASLVLEHACAVGDWVMPPTGE
PRELSEMTNVVLRLDALPDVPAGGAPVELASEAAGYMIGDVWCVDVRCSDARTQAELLSM
TLVYRPQPEQIGAAFARAPDTTPEPAPLTSAPHAAWNGELLPAGDWSVPDDASAGIVRIG
RVPSAAVLSLWALPFPLELRLPVNHVEHVLRVLLAEWGAARADAPAVWRIGGAKLARLPA
ASADGVVEYAAGRFAVLDARGYVLLRIDDARVDGEPAQAALPETGALADPLTA

>DM55_3675|KEGG|CYP1488A1|[Burkholderia mallei]bmal|BP

MNGLPLSLPRVDSTDSLFADPLAFLARARAQCGDVFVLREHGPIFSRASDCSGVIAVFGE

HRLRQVLTETDTFALPMSAAAKMALPKNLVNLNRGLHSMREPEHGRHKRLLTGTANRALF
DAHRSGMQTDLSRFCEMLNGDGRISVVSRMRELTAKLASRLFLGPQCEEDAELTFLLSAY
FTLRREASSPNAHDPLQYRDALIGAGRQLDRALRARIRRYRKAPAGECAGLLQRLATAGQ
PGSPALSEDEIVGHANVMFVSSTEPVAISLAWLLLVLSQLPDLRRALRAESAARASSPAS
PYDAPLLERVINETLRLLTPNALMVRATTQAVSLQGVALPARCEIVVCPFLVHREANAFA
RPHAFLPSRWETARPSPYEYFPFGAGGHFCAGRNLALSLIGEVLSTLLSRFDFVLDIEQF
IDWRIHIMLMPKGDPTLTAHPVDERRDTPSPKWRGPVAELFHFAPGLS

>DM78_4548|KEGG|CYP1488A1|[Burkholderia mallei]bmae|BP

MNGLPLSLPRVDSTDSLFADPLAFLARARAQCGDVFVLREHGPIFSRASDCSGVIAVFGE
HRLRQVLTETDTFALPMSAAAKMALPKNLVNLNRGLHSMREPEHGRHKRLLTGTANRALF
DAHRSGMQTDLSRFCEMLNGDGRISVVSRMRELTAKLASRLFLGPQCEEDAELTFLLSAY
FTLRREASSPNAHDPLQYRDALIGAGRQLDRALRARIRRYRKAPAGECAGLLQRLATAGQ
PGSPALSEDEIVGHANVMFVSSTEPVAISLAWLLLVLSQLPDLRRALRAESAARASSPAS
PYDAPLLERVINETLRLLTPNALMVRATTQAVSLQGVALPARCEIVVCPFLVHREANAFA
RPHAFLPSRWETARPSPYEYFPFGAGGHFCAGRNLALSLIGEVLSTLLSRFDFVLDIEQF
IDWRIHIMLMPKGDPTLTAHPVDERRDTPSPKWRGPVAELFHFAPGLS

>DM76_4371|KEGG|CYP1488A1|[Burkholderia mallei]bmaq|BP
MNGLPLSLPRVDSTDSLFADPLAFLARARAQCGDVFVLREHGPIFSRASDCSGVIAVFGE
HRLRQVLTETDTFALPMSAAAKMALPKNLVNLNRGLHSMREPEHGRHKRLLTGTANRALF
DAHRSGMQTDLSRFCEMLNGDGRISVVSRMRELTAKLASRLFLGPQCEEDAELTFLLSAY
FTLRREASSPNAHDPLQYRDALIGAGRQLDRALRARIRRYRKAPAGECAGLLQRLATAGQ
PGSPALSEDEIVGHANVMFVSSTEPVAISLAWLLLVLSQLPDLRRALRAESAARASSPAS
PYDAPLLERVINETLRLLTPNALMVRATTQAVSLQGVALPARCEIVVCPFLVHREANAFA
RPHAFLPSRWETARPSPYEYFPFGAGGHFCAGRNLALSLIGEVLSTLLSRFDFVLDIEQF
IDWRIHIMLMPKGDPTLTAHPVDERRDTPSPKWRGPVAELFHFAPGLS

>AIO61053.1|NBI| CYP1013A3|[Burkholderia mallei]bmaq|BP
MSKHRQAPGPRGGLVMGNLAAYKRNPITMLLRLHQQYGDVARNRLGPFVTHALAHPDHIQ
YVLQENHRNYVRGRFYDNFKMFFGDGLLTTDGEFWRRHRRVVQPLFHKKQVDAHTAAVGD

AALALAYRWSALPPGKALDVVEEMMHLSLRMLGLMVFNTDVSSHAEAAGPAVRFGIEAMM
PQGNLNDFIPRWAPTRFNRRIAHARRAIDTIVAKIIADHREARCEPSDVISLLLNARDPD
TGAPMTQQEVHDEVMTVFLAGHETTGAGLAWALYALAQHPAVLRQLRDELDARLGGRAPT
VQDFEQLPYLSQVVDEVLRVYPPIWGFTRDLVEDDEIGGYRIPARSSVFMSPYVTHRHPA
FWRNPDAFDPENFASDAPARHRFVYFPFGGGMRKCIGFQTALLQMRVLVAVVAQHFDLNA
LPGHPIELGATISLRPVHGIRLIVKPRERQQSHLARVREHESARALRPLGDTATTAGAAC
PMTAAREPGASAAALAAVEAGALDAAHRGGAAAAAAGEPCPPRDPAEAAPLPLAAAPAPA
SPAVDADASARGVPAWRFTWRPVPVDPVPDEPSPALSGRRIALVNGRGGTAERVAAALAR
TCAKVSVFAPAASADHAEAARVFVNEAGPLDGIVDLGLEAPFALDDAGAWEAPMRRTVEL
LKACYGDWAAEDDTSRLFYVALTWMDGQMGYGETAGAQPLGGLWAGLAKTLPQELPNCNV
RVLDIAPDETGRVDRLVVNELYRWGLFEVGYRGGVRYTLHAKRNELPPHAVDARTTLGAG
DTVLFSGGARGIGLLCARSLAERHGCTVIVTGRETPPDGGEPWMALDEPGFKHYGLEQLR
AATPEHPPKAIRAALQRLKRRRELRTALDEMAALGLPVHYRVCDVTDRAAVRALCDELGD
SLRMVIHNAGVDRPVRLIQKRAEDFADTVRTKVLGFANLCDAVRARPRLVQFCNVGSLTG
RWGGMTGETDYAAANEALARLGLWAARHAFSPACGVKTLVWPTWEGVGMITNFAVTQRYV
SPMNVDEGVRHWLRELAAPGSGEVMFMGAVGRAPTPMQIKGFVPIRELPNIGELVTRRHH
VGEPVRFRPFKRFESRYAIDPAVAPYAHAFRFNGRAVLPASLVLEHACAVGDWVMPPTGE
PRELSEMTNVVLRLDALPDVPAGGAPVELASEAAGYMIGDVWCVDVRCSDARTQAELLSM
TLVYRPQPEQIGAAFARAPDTTPEPAPLTSAPHAAWNGELLPAGDWSVPDDASAGIVRIG
RVPSAAVLSLWALPFPLELRLPVNHVEHVLRVLLAEWGAARADAPAVWRIGGAKLARLPA
ASADGVVEYAAGRFAVLDARGYVLLRIDDARVDGEPAQAALPETGALADPLTA

>DM57_05130|KEGG|CYP1488A1|[Burkholderia mallei]bmai|BP

MNGLPLSLPRVDSTDSLFADPLAFLARARAQCGDVFVLREHGPIFSRASDCSGVIAVFGE
HRLRQVLTETDTFALPMSAAAKMALPKNLVNLNRGLHSMREPEHGRHKRLLTGTANRALF
DAHRSGMQTDLSRFCEMLNGDGRISVVSRMRELTAKLASRLFLGPQCEEDAELTFLLSAY
FTLRREASSPNAHDPLQYRDALIGAGRQLDRALRARIRRYRKAPAGECAGLLQRLATAGQ
PGSPALSEDEIVGHANVMFVSSTEPVAISLAWLLLVLSQLPDLRRALRAESAARASSPAS

PYDAPLLERVINETLRLLTPNALMVRATTQAVSLQGVALPARCEIVVCPFLVHREANAFA
RPHAFLPSRWETARPSPYEYFPFGAGGHFCAGRNLALSLIGEVLSTLLSRFDFVLDIEQF
IDWRIHIMLMPKGDPTLTAHPVDERRDTPSPKWRGPVAELFHFAPGLS

>DM57_07635|KEGG|CYP116B23|[Burkholderia mallei]bmai|BP

MTTHSASTPPALPAAQCPFRPVSDDAAAHPAGCPASAGAAAFDPFDDAYQQDPPEYVRWA
REREPVFYSPKLGYWVVARYDDIKTIFRDNRTFSPSIALEKITPTGPDANAALASYGYAM
GRTLVNEDEPTHMARRRLLIEPFTPDALRAHEPMVRRLAREYVDRFVDDGRADLVDQMLW
EVPLTVALHFLGVPEADMDTLRAYSIAHTVNTWGRPRPDEQVAVAHAVGQFWQYAGKVLE
QMREDPSGPGWMQFGIRMQREHPDIVTDSYLHSMMMAGIVAAHETTANATANALKLLLEH
PRAWRELCDDPALIPNAVDECLRHSGSIVAWRRIATRDATVGGVAIPAGAKLLIVTSSAN
RDERRFADPDLFDIHRDNPADHLTFGYGSHQCMGKHLARMEMQIFIDELSRRLPHMRLAE
QRFTYVPNTSFRGPEHLWVEWEPAENPQRRGHGARRCALAVRIGESNARRAGRALRVDSV
MPVAERVLRVRLVAPDGAPLPRWSAGAHIDVECGDTGLSRQYSLCGDPAERGVYEIAVLR
EPASRGGSAWMHERLRPGMRVKIRGPRNHFRLDGHAARAILIAGGIGITPIGAIAREARA
RGIDYALHYCGRSRRTMAFVDELAALHGERLRLHVSDEGTRHDFSALDVAAGTQVYACGP
ARMLDALAACAAHWPAHALRVEHFAAAAPRVDPRTDQPFEVELKDSGLTLTVPANRTLLA
TLRAANVDVQSDCEEGLCGSCEVRVLAGEVDHRDSVLSADERALNTRMMACCSRARHGRL
VLEL

>DM57_14575|KEGG|CYP1013A3|[Burkholderia mallei]bmai|BP

MSKHRQAPGPRGGLVIGNLAAYKRNPITMLLRLHQQYGDVARNRLGPFVTHALAHPDHIQ
YVLQENHRNYVRGRFYDNFKMFFGDGLLTTDGEFWRRHRRVVQPLFHKKQVDAHTAAVGD
AALALAYRWSALPPGKALDVVEEMMHLSLRMLGLMVFNTDVSSHAEAAGPAVRFGIEAMM
PQGNLNDFIPRWAPTRFNRRIAHARRAIDTIVAKIIADHREARCEPSDVISLLLNARDPD
TGAPMTQQEVHDEVMTVFLAGHETTGAGLAWALYALAQHPAVLRQLRDELDARLGGRAPT
VQDFEQLPYLSQVVDEVLRVYPPIWGFTRDLVEDDEIGGYRIPARSSVFMSPYVTHRHPA
FWRNPDAFDPENFASDAPARHRFVYFPFGGGMRKCIGFQTALLQMRVLVAVVAQHFDLNA
LPGHPIELGATISLRPVHGIRLIVKPRERQQSHLARVREHESARALRPLGDTATTAGAAC
PMTAAREPGASAAALAAVEAGALDAAHRGGAAAAAAGEPCPPRDPAEAAPLPLAAAPAPA

SPAVDADASARGVPAWRFTWRPVPVDPVPDEPSPALSGRRIALVNGRGGTAERVAAALAR
TCAKVSVFAPAASADHAEAARVFVNEAGPLDGIVDLGLEAPFALDDAGAWEAPMRRTVEL
LKACYGDWAAEDDTSRLFYVALTWMDGQMGYGETAGAQPLGGLWAGLAKTLPQELPNCNV
RVLDIAPDETGRVDRLVVNELYRWGLFEVGYRGGVRYTLHAKRNELPPHAVDARTTLGAG
DTVLFSGGARGIGLLCARSLAERHGCTVIVTGRETPPDGGEPWMALDEPGFKHYGLEQLR
AATPEHPPKAIRAALQRLKRRRELRTALDEMAALGLPVHYRVCDVTDRAAVRALCDELGD
SLRMVIHNAGVDRPVRLIQKRAEDFADTVRTKVLGFANLCDAVRARPRLVQFCNVGSLTG
RWGGMTGETDYAAANEALARLGLWAARHAFSPACGVKTLVWPTWEGVGMITNFAVTQRYV
SPMNVDEGVRHWLRELAAPGSGEVMFMGAVGRAPTPMQIKGFVPIRELPNIGELVTRRHH
VGEPVRFRPFKRFESRYAIDPAVAPYAHAFRFNGRAVLPASLVLEHACAVGDWVMPPTGE
PRELSEMTNVVLRLDALPDVPAGGAPVELASEAAGYMIGDVWCVDVRCSDARTQAELLSM
TLVYRPQPEQIGAAFARAPDTTPEPAPLTSAPHAAWNGELLPAGDWSVPDDASAGIVRIG
RVPSAAVLSLWALPFPLELRLPVNHVEHVLRVLLAEWGAARADAPAVWRIGGAKLARLPA
ASADGVVEYAAGRFAVLDARGYVLLRIDDARVDGEPAQAALPETGALADPLTA

>DM51_3802|KEGG|CYP1488A1|[Burkholderia mallei]bmaf|BP

MNGLPLSLPRVDSTDSLFADPLAFLARARAQCGDVFVLREHGPIFSRASDCSGVIAVFGE
HRLRQVLTETDTFALPMSAAAKMALPKNLVNLNRGLHSMREPEHGRHKRLLTGTANRALF
DAHRSGMQTDLSRFCEMLNGDGRISVVSRMRELTAKLASRLFLGPQCEEDAELTFLLSAY
FTLRREASSPNAHDPLQYRDALIGAGRQLDRALRARIRRYRKAPAGECAGLLQRLATAGQ
PGSPALSEDEIVGHANVMFVSSTEPVAISLAWLLLVLSQLPDLRRALRAESAARASSPAS
PYDAPLLERVINETLRLLTPNALMVRATTQAVSLQGVALPARCEIVVCPFLVHREANAFA
RPHAFLPSRWETARPSPYEYFPFGAGGHFCAGRNLALSLIGEVLSTLLSRFDFVLDIEQF
IDWRIHIMLMPKGDPTLTAHPVDERRDTPSPKWRGPVAELFHFAPGLS

>AIP73255.1|NCBI|CYP1013A3|[Burkholderia mallei]bmaf|BP

MSKHRQAPGPRGGLVMGNLAAYKRNPITMLLRLHQQYGDVARNRLGPFVTHALAHPDHIQ
YVLQENHRNYVRGRFYDNFKMFFGDGLLTTDGEFWRRHRRVVQPLFHKKQVDAHTAAVGD
AALALAYRWSALPPGKALDVVEEMMHLSLRMLGLMVFNTDVSSHAEAAGPAVRFGIEAMM
PQGNLNDFIPRWAPTRFNRRIAHARRAIDTIVAKIIADHREARCEPSDVISLLLNARDPD

TGAPMTQQEVHDEVMTVFLAGHETTGAGLAWALYALAQHPAVLRQLRDELDARLGGRAPT
VQDFEQLPYLSQVVDEVLRVYPPIWGFTRDLVEDDEIGGYRIPARSSVFMSPYVTHRHPA
FWRNPDAFDPENFASDAPARHRFVYFPFGGGMRKCIGFQTALLQMRVLVAVVAQHFDLNA
LPGHPIELGATISLRPVHGIRLIVKPRERQQSHLARVREHESARALRPLGDTATTAGAAC
PMTAAREPGASAAALAAVEAGALDAAHRGGAAAAAAGEPCPPRDPAEAAPLPLAAAPAPA
SPAVDADASARGVPAWRFTWRPVPVDPVPDEPSPALSGRRIALVNGRGGTAERVAAALAR
TCAKVSVFAPAASADHAEAARVFVNEAGPLDGIVDLGLEAPFALDDAGAWEAPMRRTVEL
LKACYGDWAAEDDTSRLFYVALTWMDGQMGYGETAGAQPLGGLWAGLAKTLPQELPNCNV
RVLDIAPDETGRVDRLVVNELYRWGLFEVGYRGGVRYTLHAKRNELPPHAVDARTTLGAG
DTVLFSGGARGIGLLCARSLAERHGCTVIVTGRETPPDGGEPWMALDEPGFKHYGLEQLR
AATPEHPPKAIRAALQRLKRRRELRTALDEMAALGLPVHYRVCDVTDRAAVRALCDELGD
SLRMVIHNAGVDRPVRLIQKRAEDFADTVRTKVLGFANLCDAVRARPRLVQFCNVGSLTG
RWGGMTGETDYAAANEALARLGLWAARHAFSPACGVKTLVWPTWEGVGMITNFAVTQRYV
SPMNVDEGVRHWLRELAAPGSGEVMFMGAVGRAPTPMQIKGFVPIRELPNIGELVTRRHH
VGEPVRFRPFKRFESRYAIDPAVAPYAHAFRFNGRAVLPASLVLEHACAVGDWVMPPTGE
PRELSEMTNVVLRLDALPDVPAGGAPVELASEAAGYMIGDVWCVDVRCSDARTQAELLSM
TLVYRPQPEQIGAAFARAPDTTPEPAPLTSAPHAAWNGELLPAGDWSVPDDASAGIVRIG
RVPSAAVLSLWALPFPLELRLPVNHVEHVLRVLLAEWGAARADAPAVWRIGGAKLARLPA
ASADGVVEYAAGRFAVLDARGYVLLRIDDARVDGEPAQAALPETGALADPLTA

>BM44_3635|KEGG|CYP1488A1|[Burkholderia mallei]bmaz|BP

MNGLPLSLPRVDSTDSLFADPLAFLARARAQCGDVFVLREHGPIFSRASDCSGVIAVFGE
HRLRQVLTETDTFALPMSAAAKMALPKNLVNLNRGLHSMREPEHGRHKRLLTGTANRALF
DAHRSGMQTDLSRFCEMLNGDGRISVVSRMRELTAKLASRLFLGPQCEEDAELTFLLSAY
FTLRREASSPNAHDPLQYRDALIGAGRQLDRALRARIRRYRKAPAGECAGLLQRLATAGQ
PGSPALSEDEIVGHANVMFVSSTEPVAISLAWLLLVLSQLPDLRRALRAESAARASSPAS
PYDAPLLERVINETLRLLTPNALMVRATTQAVSLQGVALPARCEIVVCPFLVHREANAFA
RPHAFLPSRWETARPSPYEYFPFGAGGHFCAGRNLALSLIGEVLSTLLSRFDFVLDIEQF
IDWRIHIMLMPKGDPTLTAHPVDERRDTPSPKWRGPVAELFHFAPGLS

>ABM99991.2|NCBI|CYP1013A3|[Burkholderia mallei]bmaz|BP

MSKHRQAPGPRGGLVMGNLAAYKRNPITMLLRLHQQYGDVARNRLGPFVTHALAHPDHIQ
YVLQENHRNYVRGRFYDNFKMFFGDGLLTTDGEFWRRHRRVVQPLFHKKQVDAHTAAVGD
AALALAYRWSALPPGKALDVVEEMMHLSLRMLGLMVFNTDVSSHAEAAGPAVRFGIEAMM
PQGNLNDFIPRWAPTRFNRRIAHARRAIDTIVAKIIADHREARCEPSDVISLLLNARDPD
TGAPMTQQEVHDEVMTVFLAGHETTGAGLAWALYALAQHPAVLRQLRDELDARLGGRAPT
VQDFEQLPYLSQVVDEVLRVYPPIWGFTRDLVEDDEIGGYRIPARSSVFMSPYVTHRHPA
FWRNPDAFDPENFASDAPARHRFVYFPFGGGMRKCIGFQTALLQMRVLVAVVAQHFDLNA
LPGHPIELGATISLRPVHGIRLIVKPRERQQSHLARVREHESARALRPLGDTATTAGAAC
PMTAAREPGASAAALAAVEAGALDAAHRGGAAAAAAGEPCPPRDPAEAAPLPLAAAPAPA
SPAVDADASARGVPAWRFTWRPVPVDPVPDEPSPALSGRRIALVNGRGGTAERVAAALAR
TCAKVSVFAPAASADHAEAARVFVNEAGPLDGIVDLGLEAPFALDDAGAWEAPMRRTVEL
LKACYGDWAAEDDTSRLFYVALTWMDGQMGYGETAGAQPLGGLWAGLAKTLPQELPNCNV
RVLDIAPDETGRVDRLVVNELYRWGLFEVGYRGGVRYTLHAKRNELPPHAVDARTTLGAG
DTVLFSGGARGIGLLCARSLAERHGCTVIVTGRETPPDGGEPWMALDEPGFKHYGLEQLR
AATPEHPPKAIRAALQRLKRRRELRTALDEMAALGLPVHYRVCDVTDRAAVRALCDELGD
SLRMVIHNAGVDRPVRLIQKRAEDFADTVRTKVLGFANLCDAVRARPRLVQFCNVGSLTG
RWGGMTGETDYAAANEALARLGLWAARHAFSPACGVKTLVWPTWEGVGMITNFAVTQRYV
SPMNVDEGVRHWLRELAAPGSGEVMFMGAVGRAPTPMQIKGFVPIRELPNIGELVTRRHH
VGEPVRFRPFKRFESRYAIDPAVAPYAHAFRFNGRAVLPASLVLEHACAVGDWVMPPTGE
PRELSEMTNVVLRLDALPDVPAGGAPVELASEAAGYMIGDVWCVDVRCSDARTQAELLSM
TLVYRPQPEQIGAAFARAPDTTPEPAPLTSAPHAAWNGELLPAGDWSVPDDASAGIVRIG
RVPSAAVLSLWALPFPLELRLPVNHVEHVLRVLLAEWGAARADAPAVWRIGGAKLARLPA
ASADGVVEYAAGRFAVLDARGYVLLRIDDARVDGEPAQAALPETGALADPLTA

>BM45_4412|KEGG|CYP1488A1|[Burkholderia mallei]bmab|BP

MNGLPLSLPRVDSTDSLFADPLAFLARARAQCGDVFVLREHGPIFSRASDCSGVIAVFGE
HRLRQVLTETDTFALPMSAAAKMALPKNLVNLNRGLHSMREPEHGRHKRLLTGTANRALF

DAHRSGMQTDLSRFCEMLNGDGRISVVSRMRELTAKLASRLFLGPQCEEDAELTFLLSAY
FTLRREASSPNAHDPLQYRDALIGAGRQLDRALRARIRRYRKAPAGECAGLLQRLATAGQ
PGSPALSEDEIVGHANVMFVSSTEPVAISLAWLLLVLSQLPDLRRALRAESAARASSPAS
PYDAPLLERVINETLRLLTPNALMVRATTQAVSLQGVALPARCEIVVCPFLVHREANAFA
RPHAFLPSRWETARPSPYEYFPFGAGGHFCAGRNLALSLIGEVLSTLLSRFDFVLDIEQF
IDWRIHIMLMPKGDPTLTAHPVDERRDTPSPKWRGPVAELFHFAPGLS

>AJX04323.1|NCBI|CYP1013A3|[Burkholderia mallei]bmab|BP

MSKHRQAPGPRGGLVMGNLAAYKRNPITMLLRLHQQYGDVARNRLGPFVTHALAHPDHIQ
YVLQENHRNYVRGRFYDNFKMFFGDGLLTTDGEFWRRHRRVVQPLFHKKQVDAHTAAVGD
AALALAYRWSALPPGKALDVVEEMMHLSLRMLGLMVFNTDVSSHAEAAGPAVRFGIEAMM
PQGNLNDFIPRWAPTRFNRRIAHARRAIDTIVAKIIADHREARCEPSDVISLLLNARDPD
TGAPMTQQEVHDEVMTVFLAGHETTGAGLAWALYALAQHPAVLRQLRDELDARLGGRAPT
VQDFEQLPYLSQVVDEVLRVYPPIWGFTRDLVEDDEIGGYRIPARSSVFMSPYVTHRHPA
FWRNPDAFDPENFASDAPARHRFVYFPFGGGMRKCIGFQTALLQMRVLVAVVAQHFDLNA
LPGHPIELGATISLRPVHGIRLIVKPRERQQSHLARVREHESARALRPLGDTATTAGAAC
PMTAAREPGASAAALAAVEAGALDAAHRGGAAAAAAGEPCPPRDPAEAAPLPLAAAPAPA
SPAVDADASARGVPAWRFTWRPVPVDPVPDEPSPALSGRRIALVNGRGGTAERVAAALAR
TCAKVSVFAPAASADHAEAARVFVNEAGPLDGIVDLGLEAPFALDDAGAWEAPMRRTVEL
LKACYGDWAAEDDTSRLFYVALTWMDGQMGYGETAGAQPLGGLWAGLAKTLPQELPNCNV
RVLDIAPDETGRVDRLVVNELYRWGLFEVGYRGGVRYTLHAKRNELPPHAVDARTTLGAG
DTVLFSGGARGIGLLCARSLAERHGCTVIVTGRETPPDGGEPWMALDEPGFKHYGLEQLR
AATPEHPPKAIRAALQRLKRRRELRTALDEMAALGLPVHYRVCDVTDRAAVRALCDELGD
SLRMVIHNAGVDRPVRLIQKRAEDFADTVRTKVLGFANLCDAVRARPRLVQFCNVGSLTG
RWGGMTGETDYAAANEALARLGLWAARHAFSPACGVKTLVWPTWEGVGMITNFAVTQRYV
SPMNVDEGVRHWLRELAAPGSGEVMFMGAVGRAPTPMQIKGFVPIRELPNIGELVTRRHH
VGEPVRFRPFKRFESRYAIDPAVAPYAHAFRFNGRAVLPASLVLEHACAVGDWVMPPTGE
PRELSEMTNVVLRLDALPDVPAGGAPVELASEAAGYMIGDVWCVDVRCSDARTQAELLSM

TLVYRPQPEQIGAAFARAPDTTPEPAPLTSAPHAAWNGELLPAGDWSVPDDASAGIVRIG
RVPSAAVLSLWALPFPLELRLPVNHVEHVLRVLLAEWGAARADAPAVWRIGGAKLARLPA
ASADGVVEYAAGRFAVLDARGYVLLRIDDARVDGEPAQAALPETGALADPLTA

>BPSS1654|KEGG|CYP116B23|[Burkholderia pseudomallei]bps|BP

MTTHSASTPPALPAAQCPFRPVSDDAAAHPARCPASAGAAAFDPFDDAYQQDPPEYVRWA
REREPVFYSPKLGYWVVARYDDIKTIFRDNRTFSPSIALEKITPTGPDANAALASYGYAM
GRTLVNEDEPTHMARRRLLIEPFTPDALRAHEPMVRRLAREYVDRFVDDGRADLVDQMLW
EVPLTVALHFLGVPEADMDTLRAYSIAHTVNTWGRPRPDEQVAVAHAVGQFWQYAGKVLG
KMREDPSGPGWMQFGIRMQREHPDIVTDSYLHSMMMAGIVAAHETTANATANALKLLLEH
PRAWRELCDDPALIPNAVDECLRHSGSIVAWRRIATRDATVGGVAIPAGAKLLIVTSSAN
RDERRFADPDLFDIHRDNPADHLTFGYGSHQCMGKHLARMEMQIFIDELSRRLPHMRLAE
QRFTYVPNTSFRGPEHLWVEWAPAENPQRRGHGARRCALAVRIGESNARRAGRALRVDSV
MPVAERVLRVRLVAPDGAPLPRWSAGAHIDVECGDTGLSRQYSLCGDPAERGVYEIAVLR
EPASRGGSAWLHERLRPGMRVKIRGPRNHFRLDGHAARAILIAGGIGVTPIGAIAREARA
RGIDYALHYCGRSRRTMAFVDELAALHGERLRLHVSDEGTRHDFSALDVAAGTQVYACGP
ARMLDALAACAAHWPAHALRVEHFAAAAPRVDPRTDQPFEVELKDSGLTLTVPANRTLLA
TLRAANVDVQSDCEEGLCGSCEVRVLAGEVDHRDSVLSADERALNTRMMACCSRARRGRL
VLEL

>BPSS2327|KEGG|CYP1013A3|[Burkholderia pseudomallei]bps|BP

MSKHRQAPGPRGGLVMGNLAAYKRNPITMLLRLHQQYGDVARNRLGPFVTHALAHPDHIQ
YVLQENHRNYVRGRFYDNFKMFFGDGLLTTDGEFWRRHRRVVQPLFHKKQVDAHTAAVGD

AALALAHRWSALPPGKALDVVEEMMHLSLRMLGLMVFNTDVSSHAEAVGPAVRFGIEAMM
PQGNLNDFIPRWAPTRFNRRIAHARRAIDTIVAKIIADHREARCEPSDVISLLLNARDPD
TGAPMTQQEVHDEVMTVFLAGHETTGAGLAWALYALAQHPAVLRQLRDELDARLGGRAPT
VQDFEQLPYLSQVVDEVLRVYPPIWGFTRDLVEDDEIGGYRIPARSSVFMSPYVTHRHPA
FWRNPDAFDPENFASDAPARHRFVYFPFGGGMRKCIGFQTALLQMRVLVAVVAQHFDLNA
LPGHPIELGATISLRPVHGIRLIVKPRERQQSHLARVREHESARALRPLGDTATTAGAAC
PMTAAREPGASAAALAAVEAGALDAAHRGGAAAAAAGEPCPPRDPAEAAPLPLAASPAPA
SPAVDADASARGVPAWRFTWRPVPVDPVPDEPSPALSGRRIALVNGRGGTAERVAAALAR
TCAKVSVFAPAASADHAEAARVFVNEAGPLDGIVDLGLEAPFALDDAGAWEAPMRRTVEL
LKACYGDWAAEDDTSRLFYVALTWMDGQMGYGETAGAQPLGGLWAGLAKTLPQELPNCNV
RVLDIAPDETGRVDRLVVNELYRWGLFEVGYRGGVRYTLHAKRNELPPHAVDARTTLGAG
DTVLFSGGARGIGLLCARALAERHGCTVIVTGRETPPDGGEPWMALDEPGFKHYGLEQLR
AATPEHPPKAIRAALQRLKRRRELKTALDEMAALGLPVHYRVCDVTDRAAVRALCDELGD
SLRMVIHNAGVDRPVRLIQKRAEDFADTVRTKVLGFANLCDAVRARPRLVQFCNVGSLTG
RWGGMTGETDYAAANEALARLGLWAARHAFSPACGVKTLVWPTWEGVGMITNFAVTQRYV
SPMNVDEGVRHWLRELATPGSGEVMFMGAVGRAPTPVQIKGFVPIRELPNIGELVTRRHH
VGEPVRFRPFKRFESRYAIDPAVAPYAHAFRFNGRAVLPASLVLEHACAVGDWVMPPTGE
PRELSEMTNVVLRLDALPDVPAGGAPVELASEAAGYMIGDVWCVDVRCSDARTQAELLSM
TLVYRPQPEQIGAAFARAPDTTPEPAPLTSAPHAAWNGELLPAGDWSVPDDASAGIVRIG
RVPPAAVLSLWALPFPLELRLPVNHVEHVLRVLLAEWGAARADAPAVWRIGGAKLARLPA
ASADGVVEYAAGRFAVLDARGYVLLRIDDARVDGEPAQAALPETGALADPLTA

>BURPS1710b_A0717|KEGG|CYP116B23|[Burkholderia pseudomallei]bpm|BP

MTTHSASTPPALPAAQCPFRPVSDDAAAHPARCPASAGAAAFDPFDDAYQQDPPEYVRWA
REREPVFYSPKLGYWVVARYDDIKTIFRDNRTFSPSIALEKITPTGPDANAALASYGYAM
GRTLVNEDEPAHMARRRLLIEPFTPDALRAHEPMVRRLAREYVDRFVDDGRADLVDQMLW
EVPLTVALHFLGVPEADMDTLRAYSIAHTVNTWGRPRPDEQVAVAHAVGQFWQYAGKVLE
KMREDPSGPGWMQFGIRMQREHPDIVTDSYLHSMMMAGIVAAHETTANATANALKLLLEH
PRAWRELCDDPALIPNAVDECLRHSGSIVAWRRIATRDATVGGVAIPAGAKLLIVTSSAN
RDERRFADPDLFDIHRDNPADHLTFGYGSHQCMGKHLARMEMQIFIDELSRRLPHMRLAE
QRFTYVPNTSFRGPEHLWVEWAPAENPQRRGHGARRCALAVRIDESNARRAGRALRVDSV
MPVAERVLRVRLVAPDGAPLPRWSAGAHIDVECGDTGLSRQYSLCGDPAERGVYEIAVLR

EPASRGGSAWLHERLRPGMRVKIRGPRNHFRLDGHAARAILIAGGIGITPIGAIAREARA
RGIDYALHYCGRSRRTMAFVDELAALHGERLRLHVSDEGTRHDFSALDVAAGTQVYACGP
ARMLDALAACAAHWPAHALRVEHFAAAAPRVDPRTDQPFEVELKDSGLTLTVPANRTLLA
TLRAANVDVQSDCEEGLCGSCEVRVLAGEVDHRDSVLSADERALNTRMMACCSRARHGRL
VLEL

>BURPS1710b_A1475|KEGG|CYP1013A3|[Burkholderia pseudomallei]bpm|BP

MSKHRQAPGPRGGLVMGNLAAYKRNPITMLLRLHQQYGDVARNRLGPFVTHALAHPDHIQ
YVLQENHRNYVRGRFYDNFKMFFGDGLLTTDGEFWRRHRRVVQPLFHKKQVDAHTAAVGD
AALALAHRWSALPPGKALDVVEEMMHLSLRMLGLMVFNTDVSSHAEAAGPAVRFGIEAMM
PQGNLNDFIPRWAPTRFNRRIAHARRAIDTIVAKIIADHREARCEPSDVISLLLNARDPD
TGAPMTQQEVHDEVMTVFLAGHETTGAGLAWALYALAQHPAVLRQLRDELDARLGGRAPT
VQDFEQLPYLSQVVDEVLRVYPPIWGFTRDLVEDDEIGGYRIPARSSVFMSPYVTHRHPA
FWRNPDAFDPENFASDAPARHRFVYFPFGGGMRKCIGFQTALLQMRVLVAVVAQHFDLNA
LPGHPIELGATISLRPVHGIRLIVKPRERQQSHLARVREHESARALRPLGDTATTAGAAC
PMTAAREPGASAAALAAVEAGALDAAHRGGAAAAAAGEPCPPRDPAEAAPLPLAAAPAPA
SPAVDADASARGVPAWRFTWRPVPVDPVPDEPSPALSGRRIALVNGRGGTAERVAAALAR
TCAKVSVFAPAASADHAEAARVFVNEAGPLDGIVDLGLEAPFALDDAGAWEAPMRRTVEL
LKACYGDWAAEDDTSRLFYVALTWMDGQMGYGETAGAQPLGGLWAGLAKTLPQELPNCNV
RVLDIAPDETGRVDRLVVNELYRWGLFEVGYRGGVRYTLHAKRNELPPHAVDARTTLGAG
DTVLFSGGARGIGLLCARALAERHGCTVIVTGRETPPDGGEPWMALDEPGFKHYGLEQLR
AATPEHPPKAIRAALQRLKRRRELKTALDEMAALGLPVHYRVCDVTDRAAVRALCDELGD
SLRMVIHNAGVDRPVRLIQKRAEDFADTVRTKVLGFANLCDAVRAQPRLVQFCNVGSLTG
RWGGMTGETDYAAANEALARLGLWAARHAFSPACGVKTLVWPTWEGVGMITNFAVTQRYV
SPMNVDEGVRHWLRELATPGSGEVMFMGAVGRAPTPVQIKGFVPIRELPNIGELVTRRHH
VGEPVRFRPFKRFESRYAIDPAVAPYAHAFRFNGRAVLPASLVLEHACAVGDWVMPPTGE
PRELSEMTNVVLRLDALPDVPAGGAPVELASEAAGYMIGDVWCVDVRCSDARTQAELLSM
TLVYRPQPEQIGAAFARAPDTTPEPAPLTSAPHAAWNGELLPAGDWSVPDDASAGIVRIG
RVPPAAVLSLWALPFPLELRLPVNHVEHVLRVLLAEWGAARADAPAVWRIGGAKLARLPA
ASADGVVEYAAGRFAVLDARGYVLLRIDDARVDGEPAQAALPETGALADPLTA

>BURPS1106A_A0029|KEGG|CYP1488A1|[Burkholderia pseudomallei]bpl|BP

MNGLPLSLPRVDSTDSLFADPLAFLARARAQCGDVFVLREHGPIFSRASDCSGVIAVFGE
HRLRQVLTETDTFALPMSAAAKMALPKNLVNLNRGLHSMREPEHGRHKRLLTGTANRALF
DAHRSGMQTDLSRFCEMLNGDGRISVVSRMRELTAKLASRLFLGPQCEEDAELTFLLSAY
FTLRREASSPNAHDPLQYRDALIGAGRQLDRALRARIRRYRKAPAGECAGLLQRLATAGQ
PGSPALSEDEIVGHANVMFVSSTEPVAISLAWLLLVLSQLPDLRRALRAESAARASSPAS
PYDAPLLERVINETLRLLTPNALMVRATTQAVSLQGVALPARCEIVVCPFLVHREANAFA
RPHAFLPSRWETARPSPYEYFPFGAGGHFCAGRNLALSLIGEVLSTLLSRFDFVLDIEQF
IDWRIHIMLMPKGDPTLTAHPVDERRDTPSPKWRGPVAELFHFAPGLS

>BURPS1106A_A2242|KEGG|CYP116B23|[Burkholderia pseudomallei]bpl|BP

MTTHSASTPPALPAAQCPFRPVSDDAAAHPAGCPASAGAAAFDPFDDAYQQDPPEYVRWA
REREPVFYSPKLGYWVVARYDDIKTIFRDNRTFSPSIALEKITPTGPDANAALASYGYAM
GRTLVNEDEPAHMARRRLLIEPFTPDALRAHEPMVRRLAREYVDRFVDDGRADLVDQMLW
EVPLTVALHFLGVPEADMDTLRAYSIAHTVNTWGRPRPDEQVAVAHAVGQFWQYAGKVLE
QMREDPSGPGWMQFGIRMQREHPDIVTDSYLHSMMMAGIVAAHETTANATANALKLLLEH
PRAWRELCDDPALIPNAVDECLRHSGSIVAWRRIATRDATVGGVAIPAGAKLLIVTSSAN
RDERRFADPDLFDIHRDNPADHLTFGYGSHQCMGKHLARMEMQIFIDELSRRLPHMRLAE
QRFTYVPNTSFRGPEHLWVEWAPAENPQRRGHGARRCALAVRIDESNARRAGRALRVDSV
MPVAERVLRVRLVAPDGAPLPRWSAGAHIDVECGDTGLSRQYSLCGDPAERGVYEIAVLR
EPASRGGSAWLHERLRPGMRVKIRGPRNHFRLDGHAARAILIAGGIGITPIGAIAREARA
RGIDYALHYCGRSRRTMAFVDELAALHGERLRLHVSDEGTRHDFSALDVAAGTQVYACGP
ARMLDALAACAAHWPAHALRVEHFAAAAPRVDPRTDQPFEVELKDSGLTLTVPANRTLLA
TLRAANVDVQSDCEEGLCGSCEVRVLAGEVDHRDSVLSADERALNTRMMACCSRARHGRL
VLEL

>BURPS1106A_A3146|KEGG|CYP1013A3|[Burkholderia pseudomallei]bpl|BP

MSKHRQAPGPRGGLVMGNLAAYKRNPITMLLRLHQQYGDVARNRLGPFVTHALAHPDHIQ
YVLQENHRNYVRGRFYDNFKMFFGDGLLTTDGEFWRRHRRVVQPLFHKKQVDAHTAAVGD
AALALAHRWSALPPGKALDVVEEMMHLSLRMLGLMVFNTDVSSHAEAVGPAVRFGIEAMM
PQGNLNDFIPRWAPTRFNRRIAHARRAIDTIVAKIIADHREARCEPSDVISLLLNARDPD
TGAPMTQQEVHDEVMTVFLAGHETTGAGLAWALYALAQHPAVLRQLRDELDARLGGRAPT
VQDFEQLPYLSQVVDEVLRVYPPIWGFTRDLVEDDEIGGYRIPARSSVFMSPYVTHRHPA
FWRNPDAFDPENFASDAPARHRFVYFPFGGGMRKCIGFQTALLQMRVLVAVVAQHFDLNA
LPGHPIELGATISLRPVHGIRLIVKPRERQQSHLARVREHESARALRPLGDTATTAGAAC
PMTAAREPGASAAALAAVEAGALDAAHRGGAAAAAAGEPCPPRDPAEAAPLPLAASPAPA
SPAVDADASARGVPAWRFTWRPVPVDPVPDEPSPALSGRRIALVNGRGGTAERVAAALAR
TCAKVSVFAPAASADHAEAARVFVNEAGPLDGIVDLGLEAPFALDDAGAWEAPMRRTVEL
LKACYGDWAAEDDTSRLFYVALTWMDGQMGYGETAGAQPLGGLWAGLAKTLPQELPNCNV
RVLDIAPDETGRVDRLVVNELYRWGLFEVGYRGGVRYTLHAKRNELPPHAVDARTTLGAG
DTVLFSGGARGIGLLCARALAERHGCTVIVTGRETPPDGGEPWMALDEPGFKHYGLEQLR
AATPEHPPKAIRAALQRLKRRRELRTALDEMAALGLPVHYRVCDVTDRAAVRALCDELGD
SLRMVIHNAGVDRPVRLIQKRAEDFADTVRTKVLGFANLCDAVRARPRLVQFCNVGSLTG
RWGGMTGETDYAAANEALARLGLWAARHAFSPACGVKTLVWPTWEGVGMITNFAVTQRYV
SPMNVDEGVRHWLRELAAPGSGEVMFMGAVGRAPTPVQIKGFVPIRELPNIGELVTRRHH
VGEPVRFRPFKRFESRYAIDPAVAPYAHAFRFNGRAVLPASLVLEHACAVGDWVMPPTGE
PRELSEMTNVVLRLDALPDVPAGGAPVELASEAAGYMIGDVWCVDVRCSDARTQAELLSM
TLVYRPQPEQIGAAFARAPDTTPEPAPLTSAPHAAWNGELLPAGDWSVPDDASAGIVRIG
RVPPAAVLSLWALPFPLELRLPVNHVEHVLRVLLAEWGAARADAPAVWRIGGAKLARLPA
ASADGVVEYAAGRFAVLDARGYVLLRIDDARVDGEPAQAALPETGALADPLTA

>BURPS668_A3260|KEGG|CYP1013A3|[Burkholderia pseudomallei]bpd|BP

MSKHRQAPGPRGGLVMGNLAAYKRNPITMLLRLHQQYGDVARNRLGPFVTHAVAHPDHIQ
YVLQENHRNYVRGRFYDNFKMFFGDGLLTTDGEFWRRHRRVVQPLFHKKQVDAHTAAVGD

AALALAHRWSALPPGKALDVVEEMMHLSLRMLGLMVFNTDVSSHAEAVGPAVRFGIEAMM
PQGNLNDFIPRWAPTRFNRRIAHARRAIDTIVAKIIADHREARCEPSDVISLLLNARDPD
TGAPMTQQEVHDEVMTVFLAGHETTGAGLAWALYALAQHPAVLRQLRDELDARLGGRAPT
VQDFEQLPYLSQVVDEVLRVYPPIWGFTRDLVEDDEIGGYRIPARSSVFMSPYVTHRHPA
FWRNPDAFDPENFASDAPARHRFVYFPFGGGMRKCIGFQTALLQMRVLVAVVAQHFDLNA
LPGHPIELGATISLRPVHGIRLIVKPRERQQSHLARVREHESARALRPLGDTATTAGAAC
PMTAAREPGASAAALAAVEAGALDAAHRGGAAAAAAGEPCPPRDPAEAAPLPLAAAPAPA
SPAVDADASARGVPAWRFTWRPVPVDPVPDEPSPALSGRRIALVNGRGGTAERVAAALAR
TCAKVSVFAPAASADHAEAARVFVNEAGPLDGIVDLGLEAPFALDDAGAWEAPMRRTVEL
LKACYGDWAAEDDTSRLFYVALTWMDGQMGYGETAGAQPLGGLWAGLAKTLPQELPNCNV
RVLDIAPDETGRVDRLVVNELYRWGLFEVGYRGGVRYTLHAKRNELPPHAVDARTTLGAG
DTVLFSGGARGIGLLCARALAERHGCTVIVTGRETPPDGGEPWMALDEPGFKHYGLEQLR
AATPEHPPKAIRAALQRLKRRRELRTALDEMAALGLPVHYRVCDVTDRAAVRALCDELGD
SLRMVIHNAGVDRPVRLIQKRAEDFADTVRTKVLGFANLCDAVRARPRLVQFCNVGSLTG
RWGGMTGETDYAAANEALARLGLWAARHAFSPACGVKTLVWPTWEGVGMITNFAVTQRYV
SPMNVDEGVRHWLRELAAPGSGEVMFMGAVGRAPTPVQIKGFVPIRELPNIGELVTRRHH
VGEPVRFRPFKRFESRYVIDPAVAPYAHAFRFNGRAVLPASLVLEHACAVGDWVMPPTGE
PRELSEMTNVVLRLDALPDVPAGGAPVELASEAAGYMIGDVWCVDVRCSDARTQAELLSM
TLVYRPQPEQIGAAFARAPDTTPEPAPLTSAPHAAWNGELLPAGDWSVPDDASAGIVRIG
RVPPAAVLSLWALPFPLELRLPVNHVEHVLRVLLAEWGAARADAPAVWRIGGAKLARLPA
ASADGVVEYAAGRFAVLDARGYVLLRIDDARVDGEPAQAALPETGALADPLTA

>ABN86800.1|NCBI|CYP1488A1|[Burkholderia pseudomallei]bpd|BP

MNGLPLSLPRVDSTDSLFADPLAFLARARAQCGDVFVLREHGPIFSRASDCSGVIAVFGE
HRLRQVLTETDTFALPMSAAAKMALPKNLVNLNRGLHSMREPEHGRHKRLLTGTANRALF
DAHRSGMQTDLSRFCEMLNGDGRISVVSRMRELTAKLASRLFLGPQCEEDAELTFLLSAY
FTLRREASSPNAHDPLQYRDALIGAGRQLDRALRARIRRYRKAPAGECAGLLQRLATAGQ

PGSPALSEDEIVGHANVMFVSSTEPVAISLAWLLLVLSQLPDLRRALRAESAARASSPAS
PYDAPLLERVINETLRLLTPNALMVRATTQAVSLQGVALPARCEIVVCPFLVHREANAFA
RPHAFSPSRWETARPSPYEYFPFGAGGHFCAGRNLALSLIGEVLSTLLSRFDFVLDIEQF
IDWRIHIMLMPKGDPTLMAHPVDERRDTPSPKWRGPVAELFHFAPGLS

>BDL_5855|KEGG|CYP1488A1|[Burkholderia pseudomallei]bpse|BP

MNGLPLSLPRVDSTDSLFADPLAFLARARAQCGDVFVLREHGPIFSRASDCSGVIAVFGE
HRLRQVLTETDTFALPMSAAAKMALPKNLVNLNRGLHSMREPEHGRHKRLLTGTANRALF
DAHRSGMQTDLSRFCEMLNGDGRISVVSRMRELTAKLASRLFLGPQCEEDAELTFLLSAY
FTLRREASSPNAHDPLQYRDALIGAGRQLDRALRARIRRYRKAPAGECAGLLQRLATAGQ
PGSPALSEDEIVGHANVMFVSSTEPVAISLAWLLLVLSQLPDLRRALRAESAARASSPAS
PYDAPLLERVINETLRLLTPNALMVRATTQAVSLQGVALPARCEIVVCPFLVHREANAFA
RPHAFLPSRWETARPSPYEYFPFGAGGHFCAGRNLALSLIGEVLSTLLSRFDFVLDIEQF
IDWRIHIMLMPKGDPTLTAHPVDERRDTPSPKWRGPVAELFHFAPGLS

>AGR69572.1|NCBI|CYP1013A3|[Burkholderia pseudomallei]bpse|BP

MSKHRQAPGPRGGLVMGNLAAYKRNPITMLLRLHQQYGDVARNRLGPFVTHALAHPDHIQ
YVLQENHRNYVRGRFYDNFKMFFGDGLLTTDGEFWRRHRRVVQPLFHKKQVDAHTAAVGD
AALALAHRWSALPPGKALDVVEEMMHLSLRMLGLMVFNTDVSSHAEAVGPAVRFGIEAMM
PQGNLNDFIPRWAPTRFNRRIAHARRAIDTIVAKIIADHREARCEPSDVISLLLNARDPD
TGAPMTQQEVHDEVMTVFLAGHETTGAGLAWALYALAQHPAVLRQLRDELDARLGGRAPT
VQDFEQLPYLSQVVDEVLRVYPPIWGFTRDLVEDDEIGGYRIPARSSVFMSPYVTHRHPA
FWRNPDAFDPENFASDAPARHRFVYFPFGGGMRKCIGFQTALLQMRVLVAVVAQHFDLNA
LPGHPIELGATISLRPVHGIRLIVKPRERQQSHLARVREHESARALRPLGDTATTAGAAC
PMTAAREPGASAAALAAVEAGALDAAHRGGAAAAAAGEPCPPRDPAEAAPLPLAAAPAPA

SPAVDADASARGVPAWRFTWRPVPVDPVPDEPSPALSGRRIALVNGRGGTAERVAAALAR
TCAKVSVFAPAASADHAEAARVFVNEAGPLDGIVDLGLEAPFALDDAGAWEAPMRRTVEL
LKACYGDWAAEDDTSRLFYVALTWMDGQMGYGETAGAQPLGGLWAGLAKTLPQELPNCNV
RVLDIAPDETGRVDRLVVNELYRWGLFEVGYRGGVRYTLHAKRNELPPHAVDARTTLGAG
DTVLFSGGARGIGLLCARALAERHGCTVIVTGRETPPDGGEPWMALDEPGFKHYGLEQLR
AATPEHPPKAIRAALQRLKRRRELRTALDEMAALGLPVHYRVCDVTDRAAVRALCDELGD
SLRMVIHNAGVDRPVRLIQKRAEDFADTVRTKVLGFANLCDAVRARPRLVQFCNVGSLTG
RWGGMTGETDYAAANEALARLGLWAARHAFSPACGVKTLVWPTWEGVGMITNFAVTQRYV
SPMNVDEGVRHWLRELAAPGSGEVMFMGAVGRAPTPVQIKGFVPIRELPNIGELVTRRHH
VGEPVRFRPFKRFESRYAIDPAVAPYAHAFRFNGRAVLPASLVLEHACAVGDWVMPPTGE
PRELSEMTNVVLRLDALPDVPAGGAPVELASEAAGYMIGDVWCVDVRCSDARTQAELLSM
TLVYRPQPEQIGAAFARAPDTTPEPAPLTSAPHAAWNGELLPAGDWSVPDDASAGIVRIG
RVPPAAVLSLWALPFPLELRLPVNHVEHVLRVLLAEWGAARADAPAVWRIGGAKLARLPA
ASADGVVEYAAGRFAVLDARGYVLLRIDDARVDGEPAQAALPETGALADPLTA

>BBQ_3721|KEGG|CYP1488A1|[Burkholderia pseudomallei]bpsm|BP

MNGLPLSLPRVDSTDSLFADPLAFLARARAQCGDVFVLREHGPIFSRASDCSGVIAVFGE
HRLRQVLTETDTFALPMSAAAKMALPKNLVNLNRGLHSMREPEHGRHKRLLTGTANRALF
DAHRSGMQTDLSRFCEMLNGDGRISVVSRMRELTAKLASRLFLGPQCEEDAELTFLLSAY
FTLRREASSPNAHDPLQYRDALIGAGRQLDRALRARIRRYRKAPAGECAGLLQRLATAGQ
PGSPALSEDEIVGHANVMFVSSTEPVAISLAWLLLVLSQLPDLRRALRAESAARASSPAS
PYDAPLLERVINETLRLLTPNALMVRATTQAVSLQGIALPARCEIVVCPFLVHREANAFA
RPHAFLPSRWETARPSPYEYFPFGAGGHFCAGRNLALSLIGEVLSTLLSRFDFVLDIEQF
IDWRIHIMLMPKGDPTLTAHPVDERRDTPSPKWRGPVAELFHFAPGLS

>BBQ_4488|KEGG|CYP116B23|[Burkholderia pseudomallei]bpsm|BP

MTTHSASTPPALPAAQCPFRPVSDDAAAHPAGCPASAGAAAFDPFDDAYQQDPPEYVRWA
REREPVFYSPKLGYWVVARYDDIKTIFRDNRTFSPSIALEKITPTGPDANAALASYGYAM

GRTLVNEDEPAHMARRRLLIEPFTPDALRAHEPMVRRLAREYVDRFVDDGRADLVDQMLW
EVPLTVALHFLGVPEADMDTLRAYSIAHTVNTWGRPRPDEQVAVAHAVGQFWQYAGKVLE
KMREDPSGPGWMQFGIRMQREHPDIVTDSYLHSMMMAGIVAAHETTANATANALKLLLEH
PRAWRELCDDPALIPNAVDECLRHSGSIVAWRRIATRDATVGGVAIPAGAKLLIVTSSAN
RDERRFADPDLFDIHRDNPADHLTFGYGSHQCMGKHLARMEMQIFIDELSRRLPHMRLAE
QRFTYVPNTSFRGPEHLWVEWEPAENPQRRGHDARRCALAVRIGESNARRAGRALRVDSV
MPVAERVLRVRLVAPDGAPLPRWSAGAHIDVECGDTGLSRQYSLCGDPAERGVYEIAVLR
EPASRGGSAWLHERLRPGMRVKIRGPRNHFRLDGHAARAILIAGGIGVTPIGAIAREARA
RGIDYALHYCGRSRRTMAFVDELAALHGERLRLHVSDEGTRHDFSALDVAAGTQVYACGP
ARMLDALAACAAHWPAHALRVEHFAAAAPRVDPRTDQPFEVELKDSGLTLTVPANRTLLA
TLRAANVDVQSDCEEGLCGSCEVRVLAGEVDHRDSVLSADERALNTRMMACCSRARHGRL
VLEL

>AHG37152.1|NCBI|CYP1013A3|[Burkholderia pseudomallei]bpsm|BP

MSKHRQAPGPRGGLVMGNLAAYKRNPITMLLRLHQQYGDVARNRLGPFVTHALAHPDHIQ
YVLQENHRNYVRGRFYDNFKMFFGDGLLTTDGEFWRRHRRVVQPLFHKKQVDAHTAAVGD
AALALAHRWSALPPGKALDVVEEMMHLSLRMLGLMVFNTDVSSHAEAVGPAVRFGIEAMM
PQGNLNDFIPRWAPTRFNRRIAHARRAIDTIVAKIIADHREARCEPSDVISLLLNARDPD
TGAPMTQQEVHDEVMTVFLAGHETTGAGLAWALYALAQHPAVLRQLRDELDARLGGRAPT
VQDFEQLPYLSQVVDEVLRVYPPIWGFTRDLVEDDEIGGYRIPARSSVFMSPYVTHRHPA
FWRNPDAFDPENFASDAPARHRFVYFPFGGGMRKCIGFQTALLQMRVLVAVVAQHFDLNA
LPGHPIELGATISLRPVHGIRLIVKPRERQQSHLARVREHESARALRPLGDTATTAGAAC
PMTAAREPGASAAALAAVEAGALDAAHRGGAAAARGEPCRRAIRPTAAPLPLAASPAVDA
PDASARGVPAWRFTWRPVPVDPVPDEPSPALSGRRIALVNGRGGTAERVAAALARTCAKV
SVFAPAASADHAEAARVFVNEAGPLDGIVDLGLEVPFALDDAGAWEAPMRRTVELLKACY
GDWAAEDDTSRLFYVALTWMDGQMGYGETAGAQPLGGLWAGLAKTLPQELPNCNVRVLDI
APDETGRVDRLVVNELYRWGLFEVGYRGGVRYTLHAKRNELPPHAVDARTTLGAGDTVLF
SGGARGIGLLCARALAERHGCTVIVTGRETPPDGGEPWMALDEPGFKHYGLEQLRAATPE
HPPKAIRAALQRLKRRELRTALDEMAALGLPVHYRVCDVTDRAAVRALCDELGDSLRMV
IHNAGVDRPVRLIQKRAEDFADTVRTKVLGFANLCDAVRARPRLVQFCNVGSLTGRWGGM
TGETDYAAANEALARLGLWAARHAFSPACGVKTLVWPTWEGVGMITNFAVTQRYVSPMNV

DEGVRHWLRELATPGSGEVMFMGAVGRAPTPVQIKGFVPIRELPNIGELVTRRHHVGEPV
RFRPFKRFESRYAIDPAVAPYAHAFRFNGRAVLPASLVLEHACAVGDWVMPPTGEPRELS
EMTNVVLRLDALPDVPAGGAPVELASEAAGYMIGDVWCVDVRCSDARTQAELLSMTLVYR
PQPEQIGAAFARAPDTTPEPAPLTSAPHAAWNGELLPAGDWSVPDDASAGIVRIGRVPPA
AVLSLWALPFPLELRLPVNHVEHVLRVLLAEWGAARADAPAVWRIGGAKLARLPAASADG
VVEYAAGRFAVLDARGYVLLRIDDARVDGEPAQAALPETGALADPLTA

>BBN_5106|KEGG|CYP116B23|[Burkholderia pseudomallei]bpsu|BP

MTTHSASTPPALPAAQCPFRPVSDDAAAHPAGCPASAGAAAFDPFDDAYQQDPPEYVRWA
REREPVFYSPKLGYWVVARYDDIKTIFRDNRTFSPSIALEKITPTGPDANAALASYGYAM
GRTLVNEDEPAHMARRRLLIEPFTPDALRAHEPMVRRLAREYVDRFVDDGRADLVDQMLW
EVPLTVALHFLGVPEADMDTLRAYSIAHTVNTWGRPRPDEQVAVAHAVGQFWQYAGKVLE
KMREDPSGPGWMQFGIRMQREHPDIVTDSYLHSMMMAGIVAAHETTANATANALKLLLEH
PRAWRELCDDPALIPNAVDECLRHSGSIVAWRRIATRDATVGGVAIPAGAKLLIVTSSAN
RDERRFADPDLFDIHRDNPADHLTFGYGSHQCMGKHLARMEMQIFIDELSRRLPHMRLAE
QRFTYVPNTSFRGPEHLWVEWEPAENPQRRGHDARRCALAVRIGESNARRAGRALRVDSV
MPVAERVLRVRLVAPDGAPLPRWSAGAHIDVECGDTGLSRQYSLCGDPAERGVYEIAVLR
EPASRGGSAWLHERLRPGMRVKIRGPRNHFRLDGHAARAILIAGGIGVTPIGAIAREARA
RGIDYALHYCGRSRRTMAFVDELAALHGERLRLHVSDEGTRHDFSALDVAAGTQVYACGP
ARMLDALAACAAHWPAHALRVEHFAAAAPRVDPRTDQPFEVELKDSGLTLTVPANRTLLA
TLRAANVDVQSDCEEGLCGSCEVRVLAGEVDHRDSVLSADERALNTRMMACCSRARHGRL
VLEL

>BBN_5879|KEGG|CYP1488A1|[Burkholderia pseudomallei]bpsu|BP

MNGLPLSLPRVDSTDSLFADPLAFLARARAQCGDVFVLREHGPIFSRASDCSGVIAVFGE
HRLRQVLTETDTFALPMSAAAKMALPKNLVNLNRGLHSMREPEHGRHKRLLTGTANRALF

DAHRSGMQTDLSRFCEMLNGDGRISVVSRMRELTAKLASRLFLGPQCEEDAELTFLLSAY
FTLRREASSPNAHDPLQYRDALIGAGRQLDRALRARIRRYRKAPAGECAGLLQRLATAGQ
PGSPALSEDEIVGHANVMFVSSTEPVAISLAWLLLVLSQLPDLRRALRAESAARASSPAS
PYDAPLLERVINETLRLLTPNALMVRATTQAVSLQGIALPARCEIVVCPFLVHREANAFA
RPHAFLPSRWETARPSPYEYFPFGAGGHFCAGRNLALSLIGEVLSTLLSRFDFVLDIEQF
IDWRIHIMLMPKGDPTLTAHPVDERRDTPSPKWRGPVAELFHFAPGLS

>AHG69705.1|NCBI|CYP1013A3|[Burkholderia pseudomallei]bpsu|BP

MSKHRQAPGPRGGLVMGNLAAYKRNPITMLLRLHQQYGDVARNRLGPFVTHALAHPDHIQ
YVLQENHRNYVRGRFYDNFKMFFGDGLLTTDGEFWRRHRRVVQPLFHKKQVDAHTAAVGD
AALALAHRWSALPPGKALDVVEEMMHLSLRMLGLMVFNTDVSSHAEAVGPAVRFGIEAMM
PQGNLNDFIPRWAPTRFNRRIAHARRAIDTIVAKIIADHREARCEPSDVISLLLNARDPD
TGAPMTQQEVHDEVMTVFLAGHETTGAGLAWALYALAQHPAVLRQLRDELDARLGGRAPT
VQDFEQLPYLSQVVDEVLRVYPPIWGFTRDLVEDDEIGGYRIPARSSVFMSPYVTHRHPA
FWRNPDAFDPENFASDAPARHRFVYFPFGGGMRKCIGFQTALLQMRVLVAVVAQHFDLNA
LPGHPIELGATISLRPVHGIRLIVKPRERQQSHLARVREHESARALRPLGDTATTAGAAC
PMTAAREPGASAAALAAVEAGALDAAHRGGAAAAAAGEPCPPRDPAEAAPLPLAASPAVD
ADASARGVPAWRFTWRPVPVDPVPDEPSPALSGRRIALVNGRGGTAERVAAALARTCAKV
SVFAPAASADHAEAARVFVNEAGPLDGIVDLGLEVPFALDDAGAWEAPMRRTVELLKACY

GDWAAEDDTSRLFYVALTWMDGQMGYGETAGAQPLGGLWAGLAKTLPQELPNCNVRVLDI
APDETGRVDRLVVNELYRWGLFEVGYRGGVRYTLHAKRNELPPHAVDARTTLGAGDTVLF
SGGARGIGLLCARALAERHGCTVIVTGRETPPDGGEPWMALDEPGFKHYGLEQLRAATPE
HPPKAIRAALQRLKRRRELRTALDEMAALGLPVHYRVCDVTDRAAVRALCDELGDSLRMV
IHNAGVDRPVRLIQKRAEDFADTVRTKVLGFANLCDAVRARPRLVQFCNVGSLTGRWGGM
TGETDYAAANEALARLGLWAARHAFSPACGVKTLVWPTWEGVGMITNFAVTQRYVSPMNV
DEGVRHWLRELATPGSGEVMFMGAVGRAPTPVQIKGFVPIRELPNIGELVTRRHHVGEPV
RFRPFKRFESRYAIDPAVAPYAHAFRFNGRAVLPASLVLEHACAVGDWVMPPTGEPRELS
EMTNVVLRLDALPDVPAGGAPVELASEAAGYMIGDVWCVDVRCSDARTQAELLSMTLVYR
PQPEQIGAAFARAPDTTPEPAPLTSAPHAAWNGELLPAGDWSVPDDASAGIVRIGRVPPA
AVLSLWALPFPLELRLPVNHVEHVLRVLLAEWGAARADAPAVWRIGGAKLARLPAASADG
VVEYAAGRFAVLDARGYVLLRIDDARVDGEPAQAALPETGALADPLTA

>BBX_4173|KEGG|CYP116B23|[Burkholderia pseudomallei]bpsd|BP

MTTHSASTPPALPAAQCPFRPVSDDAAAHPAGCPASAGAAAFDPFDDAYQQDPPEYLRWA
REREPVFYSPKLGYWVVARYDDIKTIFRDNRTFSPSIALEKITPTGPDANAALASYGYAM
GRTLVNEDEPAHMARRRLLIEPFTPDALRAHEPMVRRLAREYVDRFVDDGRADLVDQMLW
EVPLTVALHFLGVPEADMDTLRAYSIAHTVNTWGRPRPDEQVAVAHAVGQFWQYAGKVLE
KMREDPSGPGWMQFGIRMQREHPDIVTDSYLHSMMMAGIVAAHETTANATANALKLLLEH
PRAWRELCDDPALIPNAVDECLRHSGSIVAWRRTATRDATVGGVAIPAGAKLLIVTSSAN
RDERRFADPDLFDIHRDNPADHLTFGYGSHQCMGKHLARMEMQIFIDELSRRLPHMRLAE
QRFTYVPNTSFRGPEHLWVEWEPAENPQRRGHGARRCALAVRIGESNARRAGRALRVDSV
MPVAERVLRVRLVAPDGAPLPRWSAGAHIDVECGDTGLSRQYSLCGDPAERGVYEIAVLR
EPASRGGSAWMHERLRPGMRVKIRGPRNHFRLDGHAARAILIAGGIGITPIGAIAREARA
RGIDYALHYCGRSRRTMAFVDELAALHGERLRLHVSDEGTRHDFSALDVAAGTQVYACGP

ARMLDALAACAAHWPAHALRVEHFAAAAPRVDPRTDQPFEVELKDSGLTLTVPANRTLLA
TLRAANVDVQSDCEEGLCGSCEVRVLAGEVGHRDSVLSADERALNTRMMACCSRARHGRL
VLEL

>BBX_5024|KEGG|CYP1488A1|[Burkholderia pseudomallei]bpsd|BP

MNGLPLSLPRVDSTDSLFADPLAFLARARAQCGDVFVLREHGPIFSRASDCSGVIAVFGE
HRLRQVLTETDTFALPMSAAAKMALPKNLVNLNRGLHSMREPEHGRHKRLLTGTANRALF
DAHRSGMQTDLSRFCEMLNGDGRISVVSRMRELTAKLASRLFLGPQCEEDAELTFLLSAY
FTLRREASSPNAHDPLQYRDALIGAGRQLDRALRARIRRYRKAPAGECAGLLQRLATAGQ
PGSPALSEDEIVGHANVMFVSSTEPVAISLAWLLLVLSQLPDLRRALRAESAARASSPAS
PYDAPLLERVINETLRLLTPNALMVRATTQAVSLQGVALPARCEIVVCPFLVHREANAFA
RPHAFLPSRWETARPSPYEYFPFGAGGHFCAGRNLALSLIGEVLSTLLSRFDFVLDIEQF
IDWRIHIMLMPKGDPTLTAHPVDERRDTPSPKWRGPVAELFHFAPGLS

>AHK69290.1|NCBI|CYP1013A3|[Burkholderia pseudomallei]bpsd|BP

MSKHRQAPGPRGGLVMGNLAAYKRNPITMLLRLHQQYGDVARNRLGPFVTHALAHPDHIQ
YVLQENHRNYVRGRFYDNFKMFFGDGLLTTDGEFWRRHRRVVQPLFHKKQVDAHTAAVGD
AALALAHRWSALPPGKALDVVEEMMHLSLRMLGLMVFNTDVSSHAEAVGPAVRFGIEAMM
PQGNLNDFIPRWAPTRFNRRIAHARRAIDTIVAKIIADHREARCEPSDVISLLLNARDPD
TGAPMTQQEVHDEVMTVFLAGHETTGAGLAWALYALAQHPAVLRQLRDELDARLGGRAPT
VQDFEQLPYLSQVVDEVLRVYPPIWGFTRDLVEDDEIGGYRIPARSSVFMSPYVTHRHPA
FWRNPDAFDPENFASDAPARHRFVYFPFGGGMRKCIGFQTALLQMRVLVAVVAQHFDLNA
LPGHPIELGATISLRPVHGIRLIVKPRERQQSHLARVREHESARALRPLGDTATTAGAAC
PMTAAREPGASAAALAAVEAGALDAAHRGGAAAAAAGEPCPPRDPAEAAPLPLAAAPAPA
SPAVDADASARGVPAWRFTWRPVPVDPVPDEPSPALSGRRIALVNGRGGTAERVAAALAR
TCAKVSVFAPAASADHAEAARVFVNEAGPLDGIVDLGLEAPFALDDAGAWEAPMRRTVEL
LKACYGDWAAEDDTSRLFYVALTWMDGQMGYGETAGAQPLGGLWAGLAKTLPQELPNCNV
RVLDIAPDETGRVDRLVVNELYRWGLFEVGYRGGVRYTLHAKRNELPPHAVDARTTLGAG
DTVLFSGGARGIGLLCARALAERHGCTVIVTGRETPPDGGEPWMALDEPGFKHYGLEQLR

AATPEHPPKAIRAALQRLKRRRELRTALDEMAALGLPVHYRVCDVTDRAAVRALCDELGD
SLRMVIHNAGVDRPVRLIQKRAEDFADTVRTKVLGFANLCDAVRARPRLVQFCNVGSLTG
RWGGMTGETDYAAANEALARLGLWAARHAFSPACGVKTLVWPTWEGVGMITNFAVTQRYV
SPMNVDEGVRHWLRELAAPGSGEVMFMGAVGRAPTPVQIKGFVPIRELPNIGELVTRRHH
VGEPVRFRPFKRFESRYAIDPAVAPYAHAFRFNGRAVLPASLVLEHACAVGDWVMPPTGE
PRELSEMTNVVLRLDALPDVPAGGAPVELASEAAGYMIGDVWCVDVRCSDARTQAELLSM
TLVYRPQPEQIGAAFARAPDTTPEPAPLTSAPHAAWNGELLPAGDWSVPDDASAGIVRIG
RVPPAAVLSLWALPFPLELRLPVNHVEHVLRVLLAEWGAARADAPAVWRIGGAKLARLPA
ASADGVVEYAAGRFAVLDARGYVLLRIDDARVDGEPAQAALPETGALADPLTA

>BP1026B_II1768|KEGG|CYP116B23|[Burkholderia pseudomallei]bpz|BP

MTTHSASTPPALPAAQCPFRPVSDDAAAHPAGCPASAGAAAFDPFDDAYQQDPPEYVRWA
REREPVFYSPKLGYWVVARYDDIKTIFRDNRTFSPSIALEKITPTGPDANAALASYGYAM
GRTLVNEDEPAHMARRRLLIEPFTPDALRAHEPMVRRLAREYVDRFVDDGRADLVDQMLW
EVPLTVALHFLGVPEADMDTLRAYSIAHTVNTWGRPRPDEQVAVAHAVGQFWQYAGKVLE
KMREDPSGPGWMQFGIRMQREHPDIVTDSYLHSMMMAGIVAAHETTANATANALKLLLEH
PRAWRELCDDPALIPNAVDECLRHSGSIVAWRRTATRDATVGGVAIPAGAKLLIVTSSAN
RDERRFADPDLFDIHRDNPADHLTFGYGSHQCMGKHLARMEMQIFIDELSRRLPHMRLAE
QRFTYVPNTSFRGPEHLWVEWAPAENPQRRGHDARRCALAVRIGESNARRAGRALRVDSV
MPVAERVLRVRLVAPDGAPLPRWSAGAHIDVECGDTGLSRQYSLCGDPAERGVYEIAVLR
EPASRGGSAWLHERLRPGMRVKIRGPRNHFRLDGHAARAILIAGGIGVTPIGAIAREARA
RGIDYALHYCGRSRRTMAFVDELAALHGERLRLHVSDEGTRHDFSALDVAAGTQVYACGP
ARMLDALAACAAHWPAHALRVEHFAAAAPRVDPRTDQPFEVELKDSGLTLTVPANRTLLA

TLRAANVDVQSDCEEGLCGSCEVRVLAGEVDHRDSVLSADERALNTRMMACCSRARRGRL
VLEL

>BP1026B_II2507|KEGG|CYP1013A3|[Burkholderia pseudomallei]bpz|BP

MSKHRQAPGPRGGLVMGNLAAYKRNPITMLLRLHQQYGDVARNRLGPFVTHALAHPDHIQ
YVLQENHRNYVRGRFYDNFKMFFGDGLLTTDGEFWRRHRRVVQPLFHKKQVDAHTAAVGD
AALALAHRWSALPPGKALDVVEEMMHLSLRMLGLMVFNTDVSSHAEAVGPAVRFGIEAMM
PQGNLNDFIPRWAPTRFNRRIAHARRAIDTIVAKIIADHREARCEPSDVISLLLNARDPD
TGAPMTQQEVHDEVMTVFLAGHETTGAGLAWALYALAQHPAVLRQLRDELDARLGGRAPT
VQDFEQLPYLSQVVDEVLRVYPPIWGFTRDLVEDDEIGGYRIPARSSVFMSPYVTHRHPA
FWRNPDAFDPENFASDAPARHRFVYFPFGGGMRKCIGFQTALLQMRVLAVVAQHFDLNA
LPGHPIELGATISLRPVHGIRLIVKPRERQQSHLARVREHESARALRPLGDTATTAGAAC
PMTAAREPGASAAALAAVEAGALDAAHRGGVAAAAAGEPCPPRDPAEAAPLPLAASPAPA
SPAVDADASARGVPAWRFTWRPVPVDPVPDEPSPALSGRRIALVNGRGGTAERVAAALAR
TCAKVSVFAPAASADHAEAARVFVNEAGPLDGIVDLGLEAPFALDDAGAWEAPMRRTVEL
LKACYGDWVAEDDTSRLFYVALTWMDGQMGYGETAGAQPLGGLWAGLAKTLPQELPNCNV
RVLDIAPDETGRVDRLVVNELYRWGLFEVGYRGGVRYTLHAKRNELPPHAVDARTTLGAG
DTVLFSGGARGIGLLCARALAERHGCTVIVTGRETPPDGGEPWMALDEPGFKHYGLEQLR
AATPEHPPKAIRAALQRLKRRRELRTALDEMAALGLPVHYRVCDVTDRAAVRALCDELGD
SLRMVIHNAGVDRPVRLIQKRAEDFADTVRTKVLGFANLCDAVRARPRLVQFCNVGSLTG
RWGGMTGETDYAAANEALARLGLWAARHAFSPACGVKTLVWPTWEGVGMITNFAVTQRYV
SPMNVDEGVRHWLRELATPGSGEVMFMGAVGRAPTPVQIKGFVPIRELPNIGELVTRRHH
VGEPVRFRPFKRFESRYAIDPAVAPYAHAFRFNGRAVLPASLVLEHACAVGDWVMPPTGE
PRELSEMTNVVLRLDALPDVPAGGAPVELASEAAGYMIGDVWCVDVRCSDARTQAELLSM
TLVYRPQPEQIGAAFARAPDTTPEPAPLTSAPHAAWNGELLPAGDWSVPDDASAGIVRIG
RVPPAAVLSLWALPFPLELRLPVNHVEHVLRVLLAEWGAARADAPAVWRIGGAKLARLPA
ASADGVVEYAAGRFAVLDARGYVLLRIDDARVDGEPAQAALPETGALADPLTA

>BP1026B_II0024|KEGG|CYP1488A1|[Burkholderia pseudomallei]bpz|BP

MSAAAKMALPKNLVNLNRGLHSMREPEHGRQKRLLTGTANRALFDAHRSGMQTDLSRFCE
MLNGDGRISVVSRMRELTAKLASRLFLGPQCEEDAELTFLLSAYFTLRREASSPNAHDPL
QYRDALIGAGRQLDRALRARIRRYRKAPAGECAGLLQRLATAGQPGSPALSEDEIVGHAN
VMFVSSTEPVAISLAWLLLVLSQLPDLRRALRAESAARASSPASPCGAPLLERVINETLR
LLTPNALMVRATTQAVSLQGVALPARCEIVVCPFLVHREANAFARPHAFSPSRWETARPS
PYEYFPFGAGGHFCAGRNLALSLIGEVLSTLLSRFDFVLDIEQFIDWRIHIMLMPKGDPT
LTAHPVDERRDTPSPKWRGPVAELFHFAPGLS

>BPC006_II0029|KEGG|CYP1488A1|[Burkholderia pseudomallei]bpq|BP
MNGLPLSLPRVDSTDSLFADPLAFLARARAQCGDVFVLREHGPIFSRASDCSGVIAVFGE
HRLRQVLTETDTFALPMSAAAKMALPKNLVNLNRGLHSMREPEHGRHKRLLTGTANRALF
DAHRSGMQTDLSRFCEMLNGDGRISVVSRMRELTAKLASRLFLGPQCEEDAELTFLLSAY
FTLRREASSPNAHDPLQYRDALIGAGRQLDRALRARIRRYRKAPAGECAGLLQRLATAGQ
PGSPALSEDEIVGHANVMFVSSTEPVAISLAWLLLVLSQLPDLRRALRAESAARASSPAS
PYDAPLLERVINETLRLLTPNALMVRATTQAVSLQGVALPARCEIVVCPFLVHREANAFA
RPHAFLPSRWETARPSPYEYFPFGAGGHFCAGRNLALSLIGEVLSTLLSRFDFVLDIEQF
IDWRIHIMLMPKGDPTLTAHPVDERRDTPSPKWRGPVAELFHFAPGLS

>BPC006_II2219|KEGG|CYP116B23|[Burkholderia pseudomallei]bpq|BP

MTTHSASTPPALPAAQCPFRPVSDDAAAHPAGCPASAGAAAFDPFDDAYQQDPPEYVRWA
REREPVFYSPKLGYWVVARYDDIKTIFRDNRTFSPSIALEKITPTGPDANAALASYGYAM
GRTLVNEDEPAHMARRRLLIEPFTPDALRAHEPMVRRLAREYVDRFVDDGRADLVDQMLW
EVPLTVALHFLGVPEADMDTLRAYSIAHTVNTWGRPRPDEQVAVAHAVGQFWQYAGKVLE
QMREDPSGPGWMQFGIRMQREHPDIVTDSYLHSMMMAGIVAAHETTANATANALKLLLEH
PRAWRELCDDPALIPNAVDECLRHSGSIVAWRRIATRDATVGGVAIPAGAKLLIVTSSAN
RDERRFADPDLFDIHRDNPADHLTFGYGSHQCMGKHLARMEMQIFIDELSRRLPHMRLAE
QRFTYVPNTSFRGPEHLWVEWAPAENPQRRGHGARRCALAVRIDESNARRAGRALRVDSV
MPVAERVLRVRLVAPDGAPLPRWSAGAHIDVECGDTGLSRQYSLCGDPAERGVYEIAVLR
EPASRGGSAWLHERLRPGMRVKIRGPRNHFRLDGHAARAILIAGGIGITPIGAIAREARA
RGIDYALHYCGRSRRTMAFVDELAALHGERLRLHVSDEGTRHDFSALDVAAGTQVYACGP

ARMLDALAACAAHWPAHALRVEHFAAAAPRVDPRTDQPFEVELKDSGLTLTVPANRTLLA
TLRAANVDVQSDCEEGLCGSCEVRVLAGEVDHRDSVLSADERALNTRMMACCSRARHGRL
VLEL

>BPC006_II3092|KEGG|CYP1013A3|[Burkholderia pseudomallei]bpq|BP

MSKHRQAPGPRGGLVMGNLAAYKRNPITMLLRLHQQYGDVARNRLGPFVTHALAHPDHIQ
YVLQENHRNYVRGRFYDNFKMFFGDGLLTTDGEFWRRHRRVVQPLFHKKQVDAHTAAVGD
AALALAHRWSALPPGKALDVVEEMMHLSLRMLGLMVFNTDVSSHAEAVGPAVRFGIEAMM
PQGNLNDFIPRWAPTRFNRRIAHARRAIDTIVAKIIADHREARCEPSDVISLLLNARDPD
TGAPMTQQEVHDEVMTVFLAGHETTGAGLAWALYALAQHPAVLRQLRDELDARLGGRAPT
VQDFEQLPYLSQVVDEVLRVYPPIWGFTRDLVEDDEIGGYRIPARSSVFMSPYVTHRHPA
FWRNPDAFDPENFASDAPARHRFVYFPFGGGMRKCIGFQTALLQMRVLVAVVAQHFDLNA
LPGHPIELGATISLRPVHGIRLIVKPRERQQSHLARVREHESARALRPLGDTATTAGAAC
PMTAAREPGASAAALAAVEAGALDAAHRGGAAAAAAGEPCPPRDPAEAAPLPLAASPAPA
SPAVDADASARGVPAWRFTWRPVPVDPVPDEPSPALSGRRIALVNGRGGTAERVAAALAR
TCAKVSVFAPAASADHAEAARVFVNEAGPLDGIVDLGLEAPFALDDAGAWEAPMRRTVEL
LKACYGDWAAEDDTSRLFYVALTWMDGQMGYGETAGAQPLGGLWAGLAKTLPQELPNCNV
RVLDIAPDETGRVDRLVVNELYRWGLFEVGYRGGVRYTLHAKRNELPPHAVDARTTLGAG
DTVLFSGGARGIGLLCARALAERHGCTVIVTGRETPPDGGEPWMALDEPGFKHYGLEQLR
AATPEHPPKAIRAALQRLKRRRELRTALDEMAALGLPVHYRVCDVTDRAAVRALCDELGD
SLRMVIHNAGVDRPVRLIQKRAEDFADTVRTKVLGFANLCDAVRARPRLVQFCNVGSLTG
RWGGMTGETDYAAANEALARLGLWAARHAFSPACGVKTLVWPTWEGVGMITNFAVTQRYV
SPMNVDEGVRHWLRELAAPGSGEVMFMGAVGRAPTPVQIKGFVPIRELPNIGELVTRRHH
VGEPVRFRPFKRFESRYAIDPAVAPYAHAFRFNGRAVLPASLVLEHACAVGDWVMPPTGE
PRELSEMTNVVLRLDALPDVPAGGAPVELASEAAGYMIGDVWCVDVRCSDARTQAELLSM
TLVYRPQPEQIGAAFARAPDTTPEPAPLTSAPHAAWNGELLPAGDWSVPDDASAGIVRIG
RVPPAAVLSLWALPFPLELRLPVNHVEHVLRVLLAEWGAARADAPAVWRIGGAKLARLPA
ASADGVVEYAAGRFAVLDARGYVLLRIDDARVDGEPAQAALPETGALADPLTA

>BBK_4312|KEGG|CYP116B23|[Burkholderia pseudomallei]bpk|BP

MTTHSASTPPALPAAQCPFRPVSDDAAAHPAGCPASAGAAAFDPFDDAYQQDPPEYVRWA

REREPVFYSPKLGYWVVARYDDIKTIFRDNRTFSPSIALEKITPTGPDANAALASYGYAM
GRTLVNEDEPAHMARRRLLIEPFTPDALRAHEPMVRRLAREYVDRFVDDGRADLVDQMLW
EVPLTVALHFLGVPEADMDTLRAYSIAHTVNTWGRPRPDEQVAVAHAVGQFWQYAGKVLE
KMREDPSGPGWMQFGIRMQREHPDIVTDSYLHSMMMAGIVAAHETTANATANALKLLLEH
PRAWRELCDDPALIPNAVDECLRHSGSIVAWRRTATRDATVGGVAIPAGAKLLIVTSSAN
RDERRFADPDLFDIHRDNPADHLTFGYGSHQCMGKHLARMEMQIFIDELSRRLPHMRLAE
QRFTYVPNTSFRGPEHLWVEWEPAENPQRRGHDARRCALAVRIGESNARRAGRALRVDSV
MPVAERVLRVRLVAPDGAPLPRWSAGAHIDVECGDTGLSRQYSLCGDPAERGVYEIAVLR
EPASRGGSAWMHERLRPGMRVKIRGPRNHFRLDGHAARAILIAGGIGITPIGAIAREARA
RGIDYALHYCGRSRRTMAFVDELAALHGERLRLHVSDEGTRHDFSALDVAAGTQVYACGP
ARMLDALAACAAHWPAHALRVEHFAAAAPRVDPRTDQPFEVELKDSGLTLTVPANRTLLA
TLRAANVDVQSDCEEGLCGSCEVRVLAGEVDHRDSVLSADERALNTRMMACCSRARHGRL
VLEL

>BBK_5113|KEGG|CYP1013A3|[Burkholderia pseudomallei]bpk|BP

MSKHRQAPGPRGGLVMGNLAAYKRNPITMLLRLHQQYGDVARNRLGPFVTHALAHPDHIQ
YVLQENHRNYVRGRFYDNFKMFFGDGLLTTDGEFWRRHRRVVQPLFHKKQVDAHTAAVGD
AALALAHRWSALPPGKALDVVEEMMHLSLRMLGLMVFNTDVSSHAEAVGPAVRFGIEAMM
PQGNLNDFIPRWAPTRFNRQIAHARRAIDTIVAKIIADHREARCEPSDVISLLLNARDPD
TGAPMTQQEVHDEVMTVFLAGHETTGAGLAWALYALAQHPAVLRRLRDELDARLGGRAPT
VQDFEQLPYLSQVVDEVLRVYPPIWGFTRDLVEDDEIGGYRIPARSSVFMSPYVTHRHPA
FWRNPDAFDPENFASDAPARHRFVYFPFGGGMRKCIGFQTALLQMRVLVAVVAQHFDLNA
LPGHPIELGATISLRPVHGIRLIVKPRERQQSHLARVREHESARALRPLGDTATTAGAAC
PMTAAREPGASAAALAAVEAGALDAAHRGGAAAAAAGEPCPPRDPAEAAPLPLAASPAPA
SPAVDADASARGVPAWRFTWRPVPVDPVPDEPSPALSGRRIALVNGRGGTAERVAAALAR
TCAKVSVFAPAASADHAEAARVFVNEAGPLDGIVDLGLEAPFALDDAGAWEAPMRRTVEL
LKACYGDWAAEDDTSRLFYVALTWMDGQMGYGETAGAQPLGGLWPGSRRRCRRSCRTATC
ACSTSRPTRRAASIGSSSTSSIAGGCSKSAIAAACATRCTRSATNCRRTPSTRAPRSARA
TPCCSRAARAASACCARARSPSVMAAR

>BBK_5162|KEGG|CYP1488A1|[Burkholderia pseudomallei]bpk|BP

MNGLPLSLPRVDSTDSLFADPLAFLARARAQCGDVFVLREHGPIFSRASDCSGVIAVFGE
HRLRQVLTETDTFALPMSAAAKMALPKNLVNLNRGLHSMREPEHGRHKRLLTGTANRALF
DAHRSGMQTDLSRFCEMLNGDGRISVVSRMRELTAKLASRLFLGPQCEEDAELTFLLSAY
FTLRREASSPNAHDPLQYRDALIGAGRQLDRALRARIRRYRKAPAGECAGLLQRLATAGQ
PGSPALSEDEIVGHANVMFVSSTEPVAISLAWLLLVLSQLPDLRRALRAESAARASSPAS
PYDAPLLERVINETLRLLTPNALMVRATMQAVSLQGIALPARCEIVVCPFLVHREANAFA
RPHAFLPSRWETARPSPYEYFPFGAGGHFCAGRNLALSLIGEVLSTLLSRFDFVLDIEQF
IDWRIHIMLMPKGDPTLTAHPVDERRDTPSPKWRGPVAELFHFAPGLS

>DR55_5348|KEGG|CYP1488A1|[Burkholderia pseudomallei]bpsh|BP

MNGLPLSLPRVDSTDSLFADPLAFLARARAQCGDVFVLREHGPIFSRASDCSGVIAVFGE
HRLRQVLTETDTFALPMSAAAKMALPKNLVNLNRGLHSMREPEHGRHKRLLTGTANRALF
DAHRSGMQTDLSRFCEMLNGDGRISVVSRMRELTAKLASRLFLGPQCEEDAELTFLLSAY
FTLRREASSPNAHDPLQYRDALIGAGRQLDRALRARIRRYRKAPAGECAGLLQRLATAGQ
PGSPALSEDEIVGHANVMFVSSTEPVAISLAWLLLVLSQLPDLRRALRAESAARASSPAS
PCGAPLLERVINETLRLLTPNALMVRATTQAASLQGVALPARCEIVVCPFLVHREANAFA
RPHAFLPSRWETARPSPYEYFPFGAGGHFCAGRNLALSLIGEVLSTLLSRFDFVLDIEQF
IDWRIHIMLMPKGDPTLTAHPVDERRDTPSPKWRGPVAELFHFAPGLS

>AIP54282.1|NCBI|CYP1013A3|[Burkholderia pseudomallei]bpsh|BP

MSKHRQAPGPRGGLVMGNLAAYKRNPITMLLRLHQQYGDVARNRLGPFVTHALAHPDHIQ
YVLQENHRNYVRGRFYDNFKMFFGDGLLTTDGEFWRRHRRVVQPLFHKKQVDAHTAAVGD
AALALAHRWSALPPGKALDVVEEMMHLSLRMLGLMVFNTDVSSHAEAVGPAVRFGIEAMM
PQGNLNDFIPRWAPTRFNRRIAHARRAIDTIVAKIIADHREARCEPSDVISLLLNARDPD
TGAPMTQQEVHDEVMTVFLAGHETTGAGLAWALYALAQHPAVLRQLRDELDARLGGRAPT
VQDFEQLPYLSQVVDEVLRVYPPIWGFTRDLVEDDEIGGYRIPARSSVFMSPYVTHRHPA

FWRNPDAFDPENFASDAPARHRFVYFPFGGGMRKCIGFQTALLQMRVLVAVVAQHFDLNA
LPGHPIELGATISLRPVHGIRLIVKPRERQQSHLARVREHESARALRPLGDTATTAGAAC
PMTAAREPGASAAALAAVEAGALDAAHRGGAAAAAAGEPCPPRDPAEAAPLPLAASPAPA
SPAVDADASARGVPAWRFTWRPVPVDPVPDEPSPALSGRRIALVNGRGGTAERVAAALAR
TCAKVSVFAPAASADHAEAARVFVNEAGPLDGIVDLGLEAPFALDDAGAWEAPMRRTVEL
LKACYGDWAAEDDTSRLFYVALTWMDGQMGYGETAGAQPLGGLWAGLAKTLPQELPNCNV
RVLDIAPDETGRVDRLVVNELYRWGLFEVGYRGGVRYTLHAKRNELPPHAVDARTTLGAG
DTVLFSGGARGIGLLCARALAERHGCTVIVTGRETPPDGGEPWMALDEPGFKHYGLEQLR
AATPEHPPKAIRAALQRLKRRRELKTALDEMAALGLPVHYRVCDVTDRAAVRALCDELGD
SLRMVIHNAGVDRPVRLIQKRAEDFADTVRTKVLGFANLCDAVRAQPRLVQFCNVGSLTG
RWGGMTGETDYAAANEALARLGLWAARHAFSPACGVKTLVWPTWEGVGMITNFAVTQRYV
SPMNVDEGVRHWLRELATPGSGEVMFMGAVGRAPTPVQIKGFVPIRELPNIGELVTRRHH
VGEPVRFRPFKRFESRYAIDPAVAPYAHAFRFNGRAVLPASLVLEHACAVGDWVMPPTGE
PRELSEMTNVVLRLDALPDVPAGGAPVELASEAAGYMIGDVWCVDVRCSDARTQAELLSM
TLVYRPQPEQIGAAFARAPDTTPEPAPLTSAPHAAWNGELLPAGDWSVPDDASAGIVRIG
RVPPAAVLSLWALPFPLELRLPVNHVEHVLRVLLAEWGAARADAPAVWRIGGAKLARLPA
ASADGVVEYAAGRFAVLDARGYVLLRIDDARVDGEPAQAALPETGALADPLTA

>BBU_3609|KEGG|CYP1488A1|[Burkholderia pseudomallei]bpsa|BP

MNGLPLSLPRVDSTDSLFADPLAFLARARAQCGDVFVLREHGPIFSRASDCSGVIAVFGE
HRLRQVLTETDTFALPMSAAAKMALPKNLVNLNRGLHSMREPEHGRHKRLLTGTANRALF
DAHRSGMQTDLSRFCEMLNGDGRISVVSRMRELTAKLASRLFLGPQCEEDAELTFLLSAY
FTLRREASSPNAHDPLQYRDALIGAGRQLDRALRARIRRYRKAPAGECAGLLQRLATAGQ
PGSPALSEDEIVGHANVMFVSSTEPVAISLAWLLLVLSQLPDLRRALRAESAARASSPAS
PYDAPLLERVINETLRLLTPNALMVRATTQAVSLQGIALPARCEIVVCPFLVHREANAFA
RPHAFLPSRWETARPSPYEYFPFGAGGHFCAGRNLALSLIGEVLSTLLSRFDFVLDIEQF
IDWRIHIMLMPKGDPTLMAHPVDERRDTPSPKWRGPVAELFHFAPGLS

>AIS91404.1|NCBI|CYP1013A3|[Burkholderia pseudomallei]bpsa|BP

MSKHRQAPGPRGGLVMGNLAAYKRNPITMLLRLHQQYGDVARNRLGPFVTHALAHPDHIQ
YVLQENHRNYVRGRFYDNFKMFFGDGLLTTDGEFWRRHRRVVQPLFHKKQVDAHTAAVGD
AALALAHRWSALPPGKALDVVEEMMHLSLRMLGLMVFNTDVSSHAEAVGPAVRFGIEAMM
PQGNLNDFIPRWAPTRFNRRIAHARRAIDTIVAKIIADHREARCEPSDVISLLLNARDPD
TGAPMTQQEVHDEVMTVFLAGHETTGAGLAWALYALAQHPAVLRQLRDELDARLGGRAPT
VQDFEQLPYLSQVVDEVLRVYPPIWGFTRDLVEDDEIGGYRIPARSSVFMSPYVTHRHPA
FWRNPDAFDPENFASDAPARHRFVYFPFGGGMRKCIGFQTALLQMRVLVAVVAQHFDLNA
LPGHPIELGATISLRPVHGIRLIVKPRERQQSHLARVREHESARALRPLGDTATTAGAAC
PMTAAREPGASAAALAAVEAGALDAAHRGGAAAAAAGEPCPPRDPAEAAPLPLAASPAPA
SPAVDADASARGVPAWRFTWRPVPVDPVPDEPSPALSGRRIALVNGRGGTAERVAAALAR
TCAKVSVFAPAASADHAEAARVFVNEAGPLDGIVDLGLEAPFALDDAGAWEAPMRRTVEL
LKACYGDWAAEDDTSRLFYVALTWMDGQMGYGETAGAQPLGGLWAGLAKTLPQELPNCNV
RVLDIAPDETGRVDRLVVNELYRWGLFEVGYRGGVRYTLHAKRNELPPHAVDARTTLGAG
DTVLFSGGARGIGLLCARALAERHGCTVIVTGRETPPDGGEPWMALDEPGFKHYGLEQLR
AATPEHPPKAIRAALQRLKRRRELKTALDEMAALGLPVHYRVCDVTDRAAVRALCDELGD
SLRMVIHNAGVDRPVRLIQKRAEDFADTVRTKVLGFANLCDAVRAQPRLVQFCNVGSLTG
RWGGMTGETDYAAANEALARLGLWAARHAFSPACGVKTLVWPTWEGVGMITNFAVTQRYV
SPMNVDEGVRHWLRELAAPGSGEVMFMGAVGRAPTPVQIKGFVPIRELPNIGELVTRRHH
VGEPVRFRPFKRFESRYAIDPAVAPYAHAFRFNGRAVLPASLVLEHACAVGDWVMPPTGE
PRELSEMTNVVLRLDALPDVPAGGAPVELASEAAGYMIGDVWCVDVRCSDARTQAELLSM
TLVYRPQPEQIGAAFARAPDTTPEPAPLTSAPHAAWNGELLPAGDWSVPDDASAGIVRIG
RVPPAAVLSLWALPFPLELRLPVNHVEHVLRVLLAEWGAARADAPAVWRIGGAKLARLPA
ASADGVVEYAAGRFAVLDARGYVLLRIDDARVDGEPAQAALPETGALADPLTA

>X996_5118|KEGG|CYP1488A1|[Burkholderia pseudomallei]bpso|BP

MNGLPLSLPRVDSTDSLFADPLAFLARARAQCGDVFVLREHGPIFSRASDCSGVIAVFGE
HRLRQVLTETDTFALPMSAAAKMALPKNLVNLNRGLHSMREPEHGRHKRLLTGTANRALF
DAHRSGMQTDLSRFCEMLNGDGRISVVSRMRELTAKLASRLFLGPQCEEDAELTFLLSAY
FTLRREASSPNAHDPLQYRDALIGAGRQLDRALRARIRRYRKAPAGECAGLLQRLATAGQ
PGSPALSEDEIVGHANVMFVSSTEPVAISLAWLLLVLSQLPDLRRVLRAESAARASSPAS
PYDAPLLERVINETLRLLTPNALMVRATTQAVSLQGVALPARCEIVVCPFLVHREANAFA
RPHAFLPSRWETARPSPYEYFPFGAGGHFCAGRNLALSLIGEVLSTLLSRFDFVLDIEQF
IDWRIHIMLMPKGDPTLMAHPVDERRDTPSPKWRGPVAELFHFAPGLS

>AIV93005.1|NCBI|CYP1013A3|[Burkholderia pseudomallei]bpso|BP

MSKHRQAPGPRGGLVMGNLAAYKRNPITMLLRLHQQYGDVARNRLGPFVTHALAHPDHIQ
YVLQENHRNYVRGRFYDNFKMFFGDGLLTTDGEFWRRHRRVVQPLFHKKQVDAHTAAVGD
AALALAHRWSALPPGKALDVVEEMMHLSLRMLGLMVFNTDVSSHAEAVGPAVRFGIEAMM

PQGNLNDFIPRWAPTRFNRRIAHARRAIDTIVAKIIADHREARCEPSDVISLLLNARDPD
TGAPMTQQEVHDEVMTVFLAGHETTGAGLAWALYALAQHPAVLRQLRDELDARLGGRAPT
VQDFEQLPYLSQVVDEVLRVYPPIWGFTRDLVEDDEIGGYRIPARSSVFMSPYVTHRHPA
FWRNPDAFDPENFASDAPARHRFVYFPFGGGMRKCIGFQTALLQMRVLVAVVAQHFDLNA
LPGHPIELGATISLRPVHGIRLIVKPRERQQSHLARVREHESARALRPLGDTATTAGAAC
PMTAAREPGASAAALAAVEAGALDAAHRGGVAAAAAGEPCPPRDPAEAAPLPLAAAPAPA
SPAVDADASARGVPAWRFTWRPVPVDPVPDEPSPALSGRRIALVNGRGGTAERVAAALAR
TCAKVSVFAPAASADHAEAARVFVNEAGPLDGIVDLGLEAPFALDDAGAWEAPMRRTVEL
LKACYGDWAAEDDTSRLFYVALTWMDGQMGYGETAGAQPLGGLWAGLAKTLPQELPNCNV
RVLDIAPDETGRVDRLVVNELYRWGLFEVGYRGGVRYTLHAKRNELPPHAVDARTTLGAG
DTVLFSGGARGIGLLCARSLAERHGCTVIVTGRETPPDGGEPWMALDEPGFKHYGLEQLR
AATPEHPPKAIRAALQRLKRRRELRTALDEMAALGLPVHYRVCDVTDRAAVRALCDELGD
SLRMVIHNAGVDRPVRLIQKRAEDFADTVRTKVLGFANLCDAVRARPRLVQFCNVGSLTG
RWGGMTGETDYAAANEALARLGLWAARHAFSPACGVKTLVWPTWEGVGMITNFAVTQRYV
SPMNVDEGVRHWLRELAAPGSGEVMFMGAVGRAPTPVQIKGFVPIRELPNIGELVTRRHH
VGEPVRFRPFKRFESRYAIDPAVAPYAHAFRFNGRAVLPASLVLEHACAVGDWVMPPTGE
PRELSEMTNVVLRLDALPDVPAGGAPVELASEAAGYMIGDVWCVDVRCSDARTQAELLSM
TLVYRPQPEQIGAAFARAPDTTPEPAPLTSAPHAAWNGELLPAGDWSVPDDASAGIVRIG
RVPPAAVLSLWALPFPLELRLPVNHVEHVLRVLLAEWGAARADAPAVWRIGGAKLARLPA
ASADGVVEYAAGRFAVLDARGYVLLRIDDARVDGEPAQAALPETGALADPLTA

>X994_4668|KEGG|CYP1488A1|[Burkholderia pseudomallei]but|BP

MNGLPLSLPRVDSTDSLFADPLAFLARARAQCGDVFVLREHGPIFSRASDCSGVIAVFGE
HRLRQVLTETDTFALPMSAAAKMALPKNLVNLNRGLHSMREPEHGRHKRLLTGTANRALF
DAHRSGMQTDLSRFCEMLNGDGRISVVSRMRELTAKLASRLFLGPQCEEDAELTFLLSAY

FTLRREASSPNAHDPLQYRDALIGAGRQLDRALRARIRRYRKAPAGECAGLLQRLATAGQ
PGSPALSEDEIVGHANVMFVSSTEPVAISLAWLLLVLSQLPDLRRALRAESAARASSPAS
PYDAPLLERVINETLRLLTPNALMVRATTQAVSLQGVALPARCEIVVCPFLVHREANAFA
HPHAFSPSRWETARPSPYEYFPFGAGGHFCAGRNLALSLIGEVLSTLLSRFDFVLDIEQF
IDWRIHIMLMPKGDPTLTAHPVDERRDTPSPKWRGPVAELFHFAPGLS

>AIV75465.1|NCBI|CYP1013A3|[Burkholderia pseudomallei]but|BP

MSKHRQAPGPRGGLVMGNLAAYKRNPITMLLRLHQQYGDVARNRLGPFVTHALAHPDHIQ
YVLQENHRNYVRGRFYDNFKMFFGDGLLTTDGEFWRRHRRVVQPLFHKKQVDAHTAAVGD
AALALAHRWSALPPGKALDVVEEMMHLSLRMLGLMVFNTDVSSHAEAVGPAVRFGIEAMM
PQGNLNDFIPRWAPTRFNRRIAHARRAIDTIVAKIIADHREARCEPSDVISLLLNARDPD
TGAPMTQQEVHDEVMTVFLAGHETTGAGLAWALYALAQHPAVLRQLRDELDARLGGRAPT
VQDFEQLPYLSQVVDEVLRVYPPIWGFTRDLVEDDEIGGYRIPARSSVFMSPYVTHRHPA
FWRNPDAFDPENFASDAPARHRFVYFPFGGGMRKCIGFQTALLQMRVLAVVAQHFDLNA
LPGHPIELGATISLRPVHGIRLIVKPRERQQSHLARVREHESARALRPLGDTATTAGAAC
PMTAAREPGASAAALAAVEAGALDAAHRGGAAAAAAGEPCPPRDPAEAAPLPLAAAPAPA
SPAVDADASARGVPAWRFTWRPVPVDPVPDEPSPALSGRRIALVNGRGGTAERVAAALAR
TCAKVSVFAPAASADHAEAARVFVNEAGPLDGIVDLGLEAPFALDDAGAWEAPMRRTVEL
LKACYGDWAAEDDTSRLFYVALTWMDGQMGYGETAGAQPLGGLWAGLAKTLPQELPNCNV
RVLDIAPDETGRVDRLVVNELYRWGLFEVGYRGGVRYTLHAKRNELPPHAVDARTTLGAG
DTVLFSGGARGIGLLCARALAERHGCTVIVTGRETPPDGGEPWMALDEPGFKHYGLEQLR

AATPEHPPKAIRAALQRLKRRRELRTALDEMAALGLPVHYRVCDVTDRAAVRALCDELGD
SLRMVIHNAGVDRPVRLIQKRAEDFVDTVRTKVLGFANLCDAVRARPRLVQFCNVGSLTG
RWGGMTGETDYAAANEALARLGLWAARHAFSPACGVKTLVWPTWEGVGMITNFAVTQRYV
SPMNVDEGVRHWLRELAAPGSGEVMFMGAVGRAPTPVQIKGFVPIRELPNIGELVTRRHH
VGEPVRFRPFKRFESRYAIDPAVAPYAHAFRFNGRAVLPASLVLEHACAVGDWVMPPTGE
PRELSEMTNVVLRLDALPDVPAGGAPVELASEAAGYMIGDVWCVDVRCSDARTQAELLSM
TLVYRPQPEQIGAAFARAPDTTPEPAPLTSAPHAAWNGELLPAGDWSVPDDASAGIVRIG
RVPPAAVLSLWALPFPLELRLPVNHVEHVLRVLLAEWGAARADAPAVWRIGGAKLARLPA
ASADGVVEYAAGRFAVLDARGYVLLRIDDARVDGEPAQAALPETGALADPLTA

>BTH_II0026|KEGG|CYP1488A2|[Burkholderia thailandensis]bte|BP

MNGLPPSLPRVDSTESLFAEPLAFLAQARSRHGDVFVMREHGPIFSRASDCSGVIAVFGE
HRLRQILTDIDNFALPMSAAAKMALPKNLVNLNRGLHSMREPEHGRHKRLLTGTINRELF
DAHRFEIRAALNRFCEMLKVDRRISVVSRMRELTVEMASHIFLGAQCQEDDELAFLLSAY
FTLRREASSLNARDPLLYRDELIGVGQQLDRTLRERIRRYRKRPVDARAGLLQRLATAGP
PGSPALSEDEIVGHANVMFVSSTEPVAMSLTWLLLVLSQLPDLRRALRAEIADRASMPAS
TNGASWLENVVNETLRLLTPNALMVRATTRAVSLQGVALPARCEIVVCPFLAHREAKPFP
DPHAFSPSRWETARPSPYEYFPFGAGGHFCAGRNLALSLIREVLSTLLSRFDFVLDGEQS
IDWRIHIMLMPKGDPALIAHPVDERGDTPSPKWRGPITDLFHFAPGLS

>BTH_II0434|KEGG|CYP212A12|[Burkholderia thailandensis]bte|BP

MSRDLPGTLAAWQKAYGDIVHLRIWPEHDVVVADPQLARALLVTHHDALIRWERGIRVLS
QVHGHSVLTAEGEAWRTRRHALVPNFSPKAVQGFSPTIVKAATRAFAQWPREDADWHIES
ALTMLTMDVILQMVFSSTLGEDVRVAEQAIRVASAAANAEFYWPTSMPDWMPWKREKRQA
LRTLKDLIERHLQRRLKLACDAWPDDLLSCLLQSREDASALSLQNVRDECMTIFLAGHET
TAATLVWWAWCMASNPEAQRVARDEVQRVLQGNAPVAETLPELGYLVQTIKETLRRYPVA
PLLISRRATRAIQLGDWALPARTLFMIPAHLMHHDARWFPDPEAFRPERFAPDAPEVPRG
AYIPFGAGPRVCLGQHLAMAEMTVIAAMLLQRFVLSAPDGSAPPHPVLNVTLRPAQPMHL

AITQVDPRIA

>BTH_II0725|KEGG|CYP116B128|[Burkholderia thailandensis]bte|BP

MTHSASTPPASPAAQCPLHPVSDDAAAHPAGCPASAGAAAFDPFDDAYQQDPPEYVRWAR
EQEPVFYSPKLGYWIVTRYDDIKAIFRDNRTFSPSIALEKITPTGPDANAVLASYGYAMN
RTLVNEDEPAHMARRRLLIEPFTPDALRAHEPMVRRLAREYVDRFVDDGRADLVDQMLWE
VPLTVALHFLGVPEEDMDTLRKYSIAHTVNTWGRPRPDEQVAVAHAVGQFWRYAGKVLEK
MREDPSGPGWMQFGIRMQREHPDIVTDSYLHSMMMAGIVAAHETTANATANALKLLLQHP
RAWRELCDDPSLIPNAVDECLRHNGSIAAWRRIATCDATVGGVAIPAGAKLLIVTSSANR
DERRFADADLFDIHRDDCAEHLTFGYGAHQCMGKHLARMEMQIFIDELTRRLPHMRLAEQ
RFTYVPNTSFRGPEHLWVEWDPAANPERRRADARRPALAVRIGESNARGAGRALRVDSMT
PVAERVLRVRLVAPGGEPLPRWSAGAHIDIECGDTGLSRQYSLCGDPAERGAYEIAVLRE
PASRGGSAWMHERLRPGMRVKIRGPRNHFRLDAHASRAILIAGGIGITPIGAIARQARAG
GIDYTLHYCGRSRRTMAFVDELAALHGERLRLHVSDEGTRADFSALDVAPGTQVYACGPA
RMLDALAARAAHWPAHALRVEHFAAAAPRVDPAAEQPFEVELKDSGLTLTVPANRTLLAT
LRAANVDLQSDCEEGLCGSCEVRVLAGEIDHRDSVLSAGERAANTRMMACCSRAKRGRIV
LEL

>BTH_II2347|KEGG|CYP1013A3|[Burkholderia thailandensis]bte|BP

MSKHRQAPGPRGSLVMGNLAAYKRNPITMLLRLHQQYGDVARNRLGPFVTHALAHPDHIQ
YVLQENHRNYVRGRFYDNFKMFFGDGLLTTDGEFWRRHRRAVQPLFHKKQVDAHTAAVGD
AALALAHRWSALPPGKAVDVVEEMMHLSLRMLGLMVFNTDVSSHAEAVGPAVRFGIEAMM
PQGNMNDFIPRWAPTRFNRRIAHARRAIDTIIAKIIADHREARCEPSDVISLLLNARDPD
TGAPMTQQEVHDEVMTVFLAGHETTGAGLAWALYALAQHPAVLRQLRDELDARLGGRAPT
VQDFEQLPYLSQVVDEVLRVYPPIWGFTRDLVEDDEIGGYRIPARSSVFMSPYVTHRHPA
FWRNPDAFDPENFASDAPARHRFVYFPFGGGMRKCIGFQTALLQMRVLVAVVAQHFDLNA
LPGHPIELGAAISLRPVHGIRLIVKPRERQQSHLARVREHESARALRPLGDTAEAAGAVC
PMTDAAHEICVSADAAMVEAGAPGDANAGGAAAAVERAAARQHEPAAAAPLPFATSAATG
APPAASPVAAAAAGASARSVPAWRFTWRPVPVDPVPDEPSPALSGKRIALVNGRGGTIER
VAAALARTCAKVSVFAPAANADHAEAARAFVNEAGPLDGIVDLGLEAPFALDDASAWEAP

MRRTVELLKACYGDWAAEDDTSRLFYVALTWMDGQMGYGDDAGAQPLGGLWAGLAKTLPQ
ELPNCNVRVLDIAPDETGRVDRLVVNELYRWGLFEVGYRNGVRYTLHAKRDELPPLAVDA
RAALGPGDTVLFSGGARGIGLLCARALAERHGCTVLVTGRETPPDGGEPWMALDEAGFKR
YGLEQLRAATPERPPKAIRASLQRLKHRRELKAALDEMAALGLPVHYRVCDVTDRAAVRA
LCDELGDSLRIVIHNAGVDQPVRLIQKRADDFAHTVRTKVLGFANLCDAVRARPRLAQFC
NVGSLTGRWGGMTGETDYAAANEALARLGLWAAKHALPPACGVKTLVWPTWEGVGMITNF
AVTQRYVSPMNVEEGVRHWLRELASPGGGEVMFMGAVGRAPTPVQIKGFQPIRELPNIGE
LVTRRHHVGEPVRFRPFKRFEGRYAIDPAVAPYARAFRFNGRAVLPASLVLEHACAVGDW
VMPPTGEPRELSEMTNVVLRLDALPDVPAGDAPVEIASEAAGYMIGDVWCVDVRCSDART
QAELLSMTLVYRPQPEQIGAAYARAPDTKPEPAPLTSAPHAAWDGELLPAGDWSVPDDAS
AGIVRIGRVPPAAVLSLWTLPFPLELRLPVNHVEHVLRVLLAEWGAAQTDAPSVWRIGAA
KLARLPAASADGVVEYATGRFAVLDARGYVLLRVDDTRVDSDAAHAAPAETVALADPLTA

>BTQ_3316|KEGG|CYP1488A2|[Burkholderia thailandensis]btq|BP

MNGLPPSLPRVDSTESLFAEPLAFLAQARSRHGDVFVMREHGPIFSRASDCSGVIAVFGE
HRLRQILTDIDNFALPMSAAAKMALPKNLVNLNRGLHSMREPEHGRHKRLLTGTINRELF
DAHRFEIRAALNRFCEMLKVDRRISVVSRMRELTVEMASHIFLGAQCQEDDELAFLLSAY
FTLRREASSLNARDPLLYRDELIGVGQQLDRTLRERIRRYRKRPVDARAGLLQRLATAGP
PGSPALSEDEIVGHANVMFVSSTEPVAMSLTWLLLVLSQLPDLRRALRAEIADRASMPAS
TNGASWLENVVNETLRLLTPNALMVRATTRAVSLQGVALPARCEIVVCPFLAHREAKPFP
DPHAFSPSRWETARPSPYEYFPFGAGGHFCAGRNLALSLIREVLSTLLSRFDFVLDGEQS
IDWRIHIMLMPKGDPALIAHPVDERGDTPSPKWRGPITDLFHFAPGLS

>BTQ_3727|KEGG|CYP212A12|[Burkholderia thailandensis]btq|BP

MSRDLPGTLAAWQKAYGDIVHLRIWPEHDVVVADPQLARALLVTHHDALIRWERGIRVLS
QVHGHSVLTAEGEAWRTRRHALVPNFSPKAVQGFSPTIVKAATRAFAQWPREDADWHIES
ALTMLTMDVILQMVFSSTLGEDVRVAEQAIRVASAAANAEFYWPTSMPDWMPWKREKRQA
LRTLKDLIERHLQRRLKLACDAWPDDLLSCLLQSREDASALSLQNVRDECMTIFLAGHET
TAATLVWWAWCMASNPEAQRVARDEVQRVLQGNAPVAETLPELGYLVQTIKETLRRYPVA
PLLISRRATRAIQLGDWALPARTLFMIPAHLMHHDARWFPDPEAFRPERFAPDAPEVPRG
AYIPFGAGPRVCLGQHLAMAEMTVIAAMLLQRFVLSAPDGSAPPHPVLNVTLRPAQPMHL

AITQVDPRIA

>BTQ_4014|KEGG|CYP116B128|[Burkholderia thailandensis]btq|BP

MTHSASTPPASPAAQCPLHPVSDDAAAHPAGCPASAGAAAFDPFDDAYQQDPPEYVRWAR
EQEPVFYSPKLGYWIVTRYDDIKAIFRDNRTFSPSIALEKITPTGPDANAVLASYGYAMN
RTLVNEDEPAHMARRRLLIEPFTPDALRAHEPMVRRLAREYVDRFVDDGRADLVDQMLWE
VPLTVALHFLGVPEEDMDTLRKYSIAHTVNTWGRPRPDEQVAVAHAVGQFWRYAGKVLEK
MREDPSGPGWMQFGIRMQREHPDIVTDSYLHSMMMAGIVAAHETTANATANALKLLLQHP
RAWRELCDDPSLIPNAVDECLRHNGSIAAWRRIATCDATVGGVAIPAGAKLLIVTSSANR
DERRFADADLFDIHRDDCAEHLTFGYGAHQCMGKHLARMEMQIFIDELTRRLPHMRLAEQ
RFTYVPNTSFRGPEHLWVEWDPAANPERRRADARRPALAVRIGESNARGAGRALRVDSMT
PVAERVLRVRLVAPGGEPLPRWSAGAHIDIECGDTGLSRQYSLCGDPAERGAYEIAVLRE
PASRGGSAWMHERLRPGMRVKIRGPRNHFRLDAHASRAILIAGGIGITPIGAIARQARAG
GIDYTLHYCGRSRRTMAFVDELAALHGERLRLHVSDEGTRADFSALDVAPGTQVYACGPA
RMLDALAARAAHWPAHALRVEHFAAAAPRVDPAAEQPFEVELKDSGLTLTVPANRTLLAT
LRAANVDLQSDCEEGLCGSCEVRVLAGEIDHRDSVLSAGERAANTRMMACCSRAKRGRIV
LEL

>BTQ_5633|KEGG|CYP1013A3|[Burkholderia thailandensis]btq|BP

MSKHRQAPGPRGSLVMGNLAAYKRNPITMLLRLHQQYGDVARNRLGPFVTHALAHPDHIQ
YVLQENHRNYVRGRFYDNFKMFFGDGLLTTDGEFWRRHRRAVQPLFHKKQVDAHTAAVGD
AALALAHRWSALPPGKAVDVVEEMMHLSLRMLGLMVFNTDVSSHAEAVGPAVRFGIEAMM
PQGNMNDFIPRWAPTRFNRRIAHARRAIDTIIAKIIADHREARCEPSDVISLLLNARDPD
TGAPMTQQEVHDEVMTVFLAGHETTGAGLAWALYALAQHPAVLRQLRDELDARLGGRAPT
VQDFEQLPYLSQVVDEVLRVYPPIWGFTRDLVEDDEIGGYRIPARSSVFMSPYVTHRHPA
FWRNPDAFDPENFASDAPARHRFVYFPFGGGMRKCIGFQTALLQMRVLVAVVAQHFDLNA
LPGHPIELGAAISLRPVHGIRLIVKPRERQQSHLARVREHESARALRPLGDTAEAAGAVC
PMTDAAHEICVSADAAMVEAGAPGDANAGGAAAAVERAAARQHEPAAAAPLPFATSAATG
APPAASPVAAAAAGASARSVPAWRFTWRPVPVDPVPDEPSPALSGKRIALVNGRGGTIER
VAAALARTCAKVSVFAPAANADHAEAARAFVNEAGPLDGIVDLGLEAPFALDDASAWEAP
MRRTVELLKACYGDWAAEDDTSRLFYVALTWMDGQMGYGDDAGAQPLGGLWAGLAKTLPQ

ELPNCNVRVLDIAPDETGRVDRLVVNELYRWGLFEVGYRNGVRYTLHAKRDELPPLAVDA
RAALGPGDTVLFSGGARGIGLLCARALAERHGCTVLVTGRETPPDGGEPWMALDEAGFKR
YGLEQLRAATPERPPKAIRASLQRLKHRRELKAALDEMAALGLPVHYRVCDVTDRAAVRA
LCDELGDSLRIVIHNAGVDQPVRLIQKRADDFAHTVRTKVLGFANLCDAVRARPRLAQFC
NVGSLTGRWGGMTGETDYAAANEALARLGLWAAKHALPPACGVKTLVWPTWEGVGMITNF
AVTQRYVSPMNVEEGVRHWLRELASPGGGEVMFMGAVGRAPTPVQIKGFQPIRELPNIGE
LVTRRHHVGEPVRFRPFKRFEGRYAIDPAVAPYARAFRFNGRAVLPASLVLEHACAVGDW
VMPPTGEPRELSEMTNVVLRLDALPDVPAGDAPVEIASEAAGYMIGDVWCVDVRCSDART
QAELLSMTLVYRPQPEQIGAAYARAPDTKPEPAPLTSAPHAAWDGELLPAGDWSVPDDAS
AGIVRIGRVPPAAVLSLWTLPFPLELRLPVNHVEHVLRVLLAEWGAAQTDAPSVWRIGAA
KLARLPAASADGVVEYATGRFAVLDARGYVLLRVDDTRVDSDAAHAAPAETVALADPLTA

>BTJ_4293|KEGG|CYP1013A3|[Burkholderia thailandensis]btj|BP

MSKHRQAPGPRGSLVMGNLAAYKRNPITMLLRLHQQYGDVARNRLGPFVTHALAHPDHIQ
YVLQENHRNYVRGRFYDNFKMFFGDGLLTTDGEFWRRHRRAVQPLFHKKQVDAHTAAVGD
AALALAHRWSALPPGKAVDVVEEMMHLSLRMLGLMVFNTDVSSHAEAVGPAVRFGIEAMM
PQGNMNDFIPRWAPTRFNRRIAHARRAIDTIIAKIIADHREARCEPSDVISLLLNARDPD
TGAPMTQQEVHDEVMTVFLAGHETTGAGLAWALYALAQHPAVLRQLRDELDARLGGRAPT
VQDFEQLPYLSQVVDEVLRVYPPIWGFTRDLVEDDEIGGYRIPARSSVFMSPYVTHRHPA
FWRNPDAFDPENFASDAPARHRFVYFPFGGGMRKCIGFQTALLQMRVLVAVVAQHFDLNA
LPGHPIELGAAISLRPVHGIRLIVKPRERQQSHLARVREHESARALRPLGDTAEAAGAVC
PMTDAAHEICVSADAAMVEAGAPGDANAGGAAAAVERAAARQHEPAAAAPLPFATSAATG
APPAASPVAAAAGASARSVPAWRFTWRPVPVDPVPDEPSPALSGKRIALVNGRGGTIERV
AAALARTCAKVSVFAPAANADHAEAARAFVNEAGPLDGIVDLGLEAPFALDDASAWEAPM
RRTVELLKACYGDWAAEDDTSRLFYVALTWMDGQMGYGDDAGAQPLGGLWAGLAKTLPQE
LPNCNVRVLDIAPDETGRVDRLVVNELYRWGLFEVGYRNGVRYTLHAKRDELPPLAVDAR
AALGPGDTVLFSGGARGIGLLCARALAERHGCTVLVTGRETPPDGGEPWMALDEAGFKRY
GLEQLRAATPERPPKAIRASLQRLKHRRELKAALDEMAALGLPVHYRVCDVTDRAAVRAL
CDELGDSLRIVIHNAGVDQPVRLIQKRADDFAHTVRTKVLGFANLCDAVRARPRLAQFCN
VGSLTGRWGGMTGETDYAAANEALARLGLWAAKHALPPACGVKTLVWPTWEGVGMITNFA
VTQRYVSPMNVEEGVRHWLRELASPGGGEVMFMGAVGRAPTPVQIKGFQPIRELPNIGEL
VTRRHHVGEPVRFRPFKRFEGRYAIDPAVAPYARAFRFNGRAVLPASLVLEHACAVGDWV

MPPTGEPRELSEMTNVVLRLDALPDVPAGDAPVEIASEAAGYMIGDVWCVDVRCSDARTQ
AELLSMTLVYRPQPEQIGAAYARAPDTKPEPAPLTSAPHAAWDGELLPAGDWSVPDDASA
GIVRIGRVPPAAVLSLWTLPFPLELRLPVNHVEHVLRVLLAEWGAAQTDAPSVWRIGAAK
LARLPAASADGVVEYATGRFAVLDARGYVLLRVDDTRVDSDAAHAAPAETVALADPLTA

>BTJ_4348|KEGG|CYP1488A2|[Burkholderia thailandensis]btj|BP

MNGLPPSLPRVDSTESLFAEPLAFLAQARSRHGDVFVMREHGPIFSRASDCSGVIAVFGE
HRLRQILTDIDNFALPMSAAAKMALPKNLVNLNRGLHSMREPEHGRHKRLLTGTINRELF
DAHRFEIRAALNRFCEMLKVDRRISVVSRMRELTVEMASHIFLGAQCQEDDELAFLLSAY
FTLRREASSLNARDPLLYRDELIGVGQQLDRTLRERIRRYRKRPVDARAGLLQRLATAGP
PGSPALSEDEIVGHANVMFVSSTEPVAMSLTWLLLVLSQLPDLRRALRAEIADRASMPAS
TNGASWLENVVNETLRLLTPNALMVRATTRAVSLQGVALPARCEIVVCPFLAHREAKPFP
DPHAFSPSRWETARPSPYEYFPFGAGGHFCAGRNLALSLIREVLSTLLSRFDFVLDGEQS
IDWRIHIMLMPKGDPALIAHPVDERGDTPSPKWRGPITDLFHFAPGLS

>BTJ_4761|KEGG|CYP212A12|[Burkholderia thailandensis]btj|BP

MSRDLPGTLAAWQKAYGDIVHLRIWPEHDVVVADPQLARALLVTHHDALIRWERGIRVLS
QVHGHSVLTAEGEAWRTRRHALVPNFSPKAVQGFSPTIVKAATRAFAQWPREDADWHIES
ALTMLTMDVILQMVFSSTLGEDVRVAEQAIRVASAAANAEFYWPASMPDWMPWKREKRQA
LRTLKDLIERHLQRRLKLACDAWPDDLLSCLLQSREDASALSLQNVRDECMTIFLAGHET
TAATLVWWAWCMASNPEAQRVARDEVQRVLQGNAPVAETLPELGYLVQTIKETLRRYPVA
PLLISRRATRAIQLGDWALPARTLFMIPAHLMHHDARWFPDPEAFRPERFAPDAPEVPRG
AYIPFGAGPRVCLGQHLAMAEMTVIAAMLLQRFVLSAPDGSAPPHPVLNVTLRPAQPMHL
AITQVDPRIA

>BTJ_5045|KEGG|CYP116B128|[Burkholderia thailandensis]btj|BP

MTHSASTPPASPAAQCPLHPVSDDAAAHPAGCPASAGAAAFDPFDDAYQQDPPEYVRWAR
EQEPVFYSPKLGYWIVTRYDDIKAIFRDNRTFSPSIALEKITPTGPDANAVLASYGYAMN
RTLVNEDEPAHMARRRLLIEPFTPDALRAHEPMVRRLAREYVDRFVDDGRADLVDQMLWE
VPLTVALHFLGVPEEDMDTLRKYSIAHTVNTWGRPRPDEQVAVAHAVGQFWRYAGKVLEK

MREDPSGPGWMQFGIRMQREHPDIVTDSYLHSMMMAGIVAAHETTANATANALKLLLQHP
RAWRELCDDPSLIPNAVDECLRHNGSIAAWRRIATCDATVGGVAIPAGAKLLIVTSSANR
DERRFADADLFDIHRDDCAEHLTFGYGAHQCMGKHLARMEMQIFIDELTRRLPHMRLAEQ
RFTYVPNTSFRGPEHLWVEWDPAANPERRRADARRPALAVRIGESNARGAGRALRVDSMT
PVAERVLRVRLVAPGGEPLPRWSAGAHIDIECGDTGLSRQYSLCGDPAERGAYEIAVLRE
PASRGGSAWMHERLRPGMRVKIRGPRNHFRLDAHASRAILIAGGIGITPIGAIARQARAG
GIDYTLHYCGRSRRTMAFVDELAALHGERLRLHVSDEGTRADFSALDVAPGAQVYACGPA
RMLDALAARAAHWPAHALRVEHFAAAAPRVDPAAEQPFEVELKDSGLTLTVPANRTLLAT
LRAANVDLQSDCEEGLCGSCEVRVLAGEIDHRDSVLSAGERAANTRMMACCSRAKRGRIV
LEL

>BTL_3516|KEGG|CYP116B128|[Burkholderia thailandensis]btz|BP

MTHSASTPPALPAAQCPLHPVSDDAAAHPAGCPASAGAAAFDPFDDAYQQDPPEYVRWAR
EQEPVFYSPKLGYWIVTRYDDIKAIFRDNRTFSPSIALEKITPTGPDANAALASYGYAMN
RTLVNEDEPAHMARRRLLIEPFTPDALRAHEPMVRRLAREYVDRFVDDGRADLVDQMLWE
VPLTVALHFLGVPEEDMDTLRKYSIAHTVNTWGRPRPDEQVAVAHAVGQFWRYAGKVLEK
MREDPSGPGWMQFGIRMQREHPDIVTDSYLHSMMMAGIVAAHETTANAIANALKLLLQHP
RAWRELCDDPSLIPNAVDECLRHNGPIAAWRRIATCDATVGGVAIPAGAKLLIVTSSANR
DERRFADADLFDIHRDDCAEHLTFGYGAHQCMGKHLARMEMQIFIDELTRRLPHMRLAEQ
RFTYVPNTSFRGPEHLWVEWDPAANPERRRADARRPALAVRIGESNARGAGRALRVDSMT
PVAERVLRVRLVAPGGEPLPRWSAGAHIDIECGDTGLSRQYSLCGDPAERGAYEIAVLRE
PASRGGSAWMHERLRPGMRVKIRGPRNHFRLDAHASRAILIAGGIGITPIGAIARQARAG
GIDYTLHYCGRSRRTMAFVDELAALHGERLRLHVSDEGTRADFSALDVAPGTQVYACGPA
RMLDALAARAAHWPAHALRVEHFAAAAPRVDPAAEQPFEVELKDSGLTLTVPANRTLLAT
LRAANVDLQSDCEEGLCGSCEVRVLAGEIDHRDSVLSAGERAANTRMMACCSRAKRGRIV
LEL

>BTL_5148|KEGG|CYP1488A2|[Burkholderia thailandensis]btz|BP

MNGLPPSLPRVDSTESLFAEPLAFLAQARSRHGDVFVMREHGPIFSRASDCSGVIAVFGE
HRLRQILTDIDNFALPMSAAAKMALPKNLVNLNRGLHSMREPEHGRHKRLLTGTINRELF

DAHRFEIRAALNRFCEMLKVDRRISVVSRMRELTVEMASHIFLGAQCQEDDELAFLLSAY
FTLRREASSLNARDPLLYRDELIGVGQQLDRTLRERIRRYRKRPVDARAGLLQRLATAGP
PGSPALSEDEIVGHANVMFVSSTEPVAMSLTWLLLVLSQLPDLRRALRAEIADRASMPAS
TNGAPWLENVVNETLRLLTPNALMVRATTRAVSLQGVALPARCEIVVCPFLAHREAKPFP
DPHAFSPSRWETARPSPYEYFPFGAGGHFCAGRNLALSLIREVLSTLLSRFDFVLDGEQS
IDWRIHIMLMPKGDPALIAHPVDERGDTPSPKWRGPITDLFHFAPGLS

>BTL_5546|KEGG|CYP212A12|[Burkholderia thailandensis]btz|BP

MSRDLPGTLAAWQKAYGDIVHLRIWPEHDVVVADPQLARELLVTHHDALIRWERGIRVLS
QVHGHSVLTAEGEAWRTRRHALVPNFSPKAVQGFSPTIVKAATRAFAQWPREDADWHIES
ALTMLTMDVILQMVFSSTLGEDVRLAEQAIRVASAAANAEFYWPASMPDWMPWKREKRQA
LRTLKDLIERHLQRRLKLACDAWPDDLLSCLLQSREDASALSLQNVRDECMTIFLAGHET
TAATLVWWAWCMASNPEAQRVARDEVQRVLQGNAPVAETLPELGYLVQTIKETLRLYPVA
PLLISRRATRAIQLGDWSLPARTLFMIPVHLMHHDARWFPDPEAFRPERFAPDAPEVPRG
AYIPFGAGPRVCLGQHLAMAEMTVIAAMLLQRFVLSAPDGSAPPHPVLNVTLRPAQPMHL
AITQVDPRIA

>BTI_4127|KEGG|CYP116B103|[Burkholderia thailandensis]btd|BP

MSTPPASPAAQCPFHPASADVSPHPAGCPASAAAAAFDPFDDAYQQDPPEYVRWAREQEP
VFYSPKLGYWVVARYDDIKAIFRDNRTFSPSIALEKIMPTGADANAVLASYGYAMNRTLV
NEDEPAHMARRRLLIEPFTPDALRAHEPMVRRLAREYVDRFVDDGRADLVDQMLWEVPLT
VALHFLGVPEEDMDTLRQYSIAHTVNTWGRPRPDEQVAVAHAVGQFWQYAGKVLERMRED
PSGPGWMQFGIRMQREHPDIVTDSYLHSMMMAGIVAAHETTANATANALKLLLQHPHAWR
ELCEAPSLIPNAVDECLRHNGSIAAWRRIATCDTMVGGVAIPAGAKLLIVTSSANRDERR
FADPDLFDIHRDDCADHLTFGYGSHRCMGKHLARMEMQIFIDELTRRLPHMRLAEQRFTY
MPNTSFRGPEHLWVEWDPAANPERRRADARRSALAVRIGESNARSAGRALCVDSVTPAAE

RVLRVRLVAPDGAALPRWSAGAHIDVECGDTGLSRQYSLCGDPVERGAYEIAVLREPASR
GGSAWMHERLRPGMRVTIRGPRNHFRLDAHAGRTILIAGGIGITPIGAIAREARALGIDY
TLHYCGRSRRTMAFVDALAALHGGRLRLHVSDEGTRHDFSALDVAPGTQVYACGPARMLD
ALAARAAHWPADALRVEHFAAAAPRVDPAAEQPFEVELKDSGLTLTVPANRSLLVTLRAA
NVDVQSDCEEGLCGSCEVRVLAGEVDHRDSVLSAGERAANTRMMACCSRAKRGRLVLEL

>BTI_4272|KEGG|CYP1486A1|[Burkholderia thailandensis]btd|BP

MRDMSKVEYDAAKIRPFNIDPQVAAIIKNPERPEAFPPLAKVPKSIPGTSGFIAGMKSLF
AYKREGLQFFANQANIHGKVFRNQLGPYSIVGVVDHEVISQILKNGDRCWSTALAVERVT
RGLSFDIDLKKKSRLMSLDFDAHRQVRTLVQPAFSDRALASYLAIIQQEFTRNIEAWPRD
GEVPFRVEARQIFSQLAARLFFGIEDSSEAARLEGATRDYWNAIPVVLKNRHLNERWRRG
LDGVATLSSIMYDLIPKRRDEGGTDLFSQLCKSREDTGGLDDDQLVDLMLNIVFAAFDTT
TMGITSMAYLLAKHPHWQEVLRAESMRLPEQAALADFNGMERTTQVWKETLRMYPVSAIT
PRVSLGECQVGPYVVPPRTLVFNFTGLLGQDSDLWDHPQRFDPERFSPERAEYQRHRGQY
LPFGHGVHTCVGAALANLEAKVFWSIVLTRYKFSLAKDYDAVHCMNPLGSVSGEVALKVE
RL

>BTI_4729|KEGG|CYP1488A2|[Burkholderia thailandensis]btd|BP

MRERGPIFSRASDCSGVIAVFGEHRLRQILTEIDDFALPMSAAAKLALPKNLINLNRGLH
SMREPEHGRHKRLLTGTINRELFDAHRNGIRAALDRLGERLKEDGRIAVVSRMRELTSEM
ASHVFLGPRCHEEDAELAFLLSAYFTLRREASSLNARDPHVYRAELIGVGQQLDRTLRER
IRRYRNGPADDRAGLLRRLATAGLPGSPALSEDEIVGHANVMFVSSTEPVAISLTWLLLV
LSQLPGLRRALRAEIAGRASSPTSPGGAPLLENVVNETLRLLTPNALMVRATTRAVSLQG
VALPAHCEIVVCPFLAHREAGPFPDPLAFSPSRWETARPSPYEYFPFGAGGHFCAGRNLA
LLLIREVLSTLLLRFDFVLDGEQSIDWRIHIMLMPTGDPTLIAHPADERRDTPSPKWRGP
ATDLFHFAPGLS

>BTHA_4656|KEGG|CYP212A12|[Burkholderia thailandensis]btv|BP

MSRDLPGTLAAWQKAYGDIVHLRIWPEHDVVVADPQLARALLVTHHDALIRWERGIRVLS

QVHGHSVLTAEGEAWRTRRHALVPNFSPKAVQGFSPTIVKAATRAFAQWPREDADWHIES
ALTMLTMDVILQMVFSSTLGEDVRVAEQAIRVASAAANAEFYWPTSMPDWMPWKREKRQA
LRTLKDLIERHLQRRLKLACDAWPDDLLSCLLQSREDASALSLQNVRDECMTIFLAGHET
TAATLVWWAWCMASNPEAQRVARDEVQRVLQGNAPVAETLPELGYLVQTIKETLRRYPVA
PLLISRRATRAIQLGDWALPARTLFMIPAHLMHHDARWFPDPEAFRPERFAPDAPEVPRG
AYIPFGAGPRVCLGQHLAMAEMTVIAAMLLQRFVLSAPDGSAPPHPVLNVTLRPAQPMHL
AITQVDPRIA

>BTHA_5068|KEGG|CYP1488A2|[Burkholderia thailandensis]btv|BP

MSAAAKMALPKNLVNLNRGLHSMREPEHGRHKRLLTGTINRELFDAHRFEIRAALNRFCE
MLKVDRRISVVSRMRELTVEMASHIFLGAQCQEDDELAFLLSAYFTLRREASSLNARDPL
LYRDELIGVGQQLDRTLRERIRRYRKRPVDARAGLLQRLATAGPPGSPALSEDEIVGHAN
VMFVSSTEPVAMSLTWLLLVLSQLPDLRRALRAEIADRASMPASTNGASWLENVVNETLR
LLTPNALMVRATTRAVSLQGVALPARCEIVVCPFLAHREAKPFPDPHAFSPSRWETARPS
PYEYFPFGAGGHFCAGRNLALSLIREVLSTLLSRFDFVLDGEQSIDWRIHIMLMPKGDPA
LIAHPVDERGDTPSPKWRGPITDLFHFAPGLS

>BTHA_5121|KEGG|CYP1013A3|[Burkholderia thailandensis]btv|BP

MSKHRQAPGPRGSLVMGNLAAYKRNPITMLLRLHQQYGDVARNRLGPFVTHALAHPDHIQ
YVLQENHRNYVRGRFYDNFKMFFGDGLLTTDGEFWRRHRRAVQPLFHKKQVDAHTAAVGD
AALALAHRWSALPPGKAVDVVEEMMHLSLRMLGLMVFNTDVSSHAEAVGPAVRFGIEAMM
PQGNMNDFIPRWAPTRFNRRIAHARRAIDTIIAKIIADHREARCEPSDVISLLLNARDPD
TGAPMTQQEVHDEVMTVFLAGHETTGAGLAWALYALAQHPAVLRQLRDELDARLGGRAPT
VQDFEQLPYLSQVVDEVLRVYPPIWGFTRDLVEDDEIGGYRIPARSSVFMSPYVTHRHPA
FWRNPDAFDPENFASDAPARHRFVYFPFGGGMRKCIGFQTALLQMRVLVAVVAQHFDLNA
LPGHPIELGAAISLRPVHGIRLIVKPRERQQSHLARVREHESARALRPLGDTAEAAGAVC
PMTDAAHEICVSADAAMVEAGAPGDANAGGAAAAVERAAARQHEPAAAAPLPFATSAATG
APPAASPVAAAAAGASARSVPAWRFTWRPVPVDPVPDEPSPALSGKRIALVNGRGGTIER
VAAALARTCAKVSVFAPAANADHAEAARAFVNEAGPLDGIVDLGLEAPFALDDASAWEAP
MRRTVELLKACYGDWAAEDDTSRLFYVALTWMDGQMGYGDDAGAQPLGGLWAGLAKTLPQ

ELPNCNVRVLDIAPDETGRVDRLVVNELYRWGLFEVGYRNGVRYTLHAKRDELPPLAVDA
RAALGPGDTVLFSGGARGIGLLCARALAERHGCTVLVTGRETPPDGGEPWMALDEAGFKR
YGLEQLRAATPERPPKAIRASLQRLKHRRELKAALDEMAALGLPVHYRVCDVTDRAAVRA
LCDELGDSLRIVIHNAGVDQPVRLIQKRADDFAHTVRTKVLGFANLCDAVRARPRLAQFC
NVGSLTGRWGGMTGETDYAAANEALARLGLWAAKHALPPACGVKTLVWPTWEGVGMITNF
AVTQRYVSPMNVEEGVRHWLRELASPGGGEVMFMGAVGRAPTPVQIKGFQPIRELPNIGE
LVTRRHHVGEPVRFRPFKRFEGRYAIDPAVAPYARAFRFNGRAVLPASLVLEHACAVGDW
VMPPTGEPRELSEMTNVVLRLDALPDVPAGDAPVEIASEAAGYMIGDVWCVDVRCSDART
QAELLSMTLVYRPQPEQIGAAYARAPDTKPEPAPLTSAPHAAWDGELLPAGDWSVPDDAS
AGIVRIGRVPPAAVLSLWTLPFPLELRLPVNHVEHVLRVLLAEWGAAQTDAPSVWRIGAA
KLARLPAASADGVVEYATGRFAVLDARGYVLLRVDDTRVDSDAAHAAPAETVALADPLTA

>BTN_4756|KEGG|CYP1013A3|[Burkholderia thailandensis]bthe|BP

MSKHRQAPGPRGSLVMGNLAAYKRNPITMLLRLHQQYGDVARNRLGPFVTHALAHPDHIQ
YVLQENHRNYVRGRFYDNFKMFFGDGLLTTDGEFWRRHRRAVQPLFHKKQVDAHTAAVGD
AALALAHRWSALPPGKAVDVVEEMMHLSLRMLGLMVFNTDVSSHAEAVGPAVRFGIEAMM
PQGNMNDFIPRWAPTRFNRRIAHARRAIDTIIAKIIADHREARCEPSDVISLLLNARDPD
TGAPMTQQEVHDEVMTVFLAGHETTGAGLAWALYALAQHPAVLRQLRDELDARLGGRAPT
VQDFEQLPYLSQVVDEVLRVYPPIWGFTRDLVEDDEIGGYRIPARSSVFMSPYVTHRHPA
FWRNPDAFDPENFASDAPARHRFVYFPFGGGMRKCIGFQTALLQMRVLVAVVAQHFDLNA
LPGHPIELGAAISLRPVHGIRLIVKPRERQQSHLARVREHESARALRPLGDTAEAAGAVC
PMTDAAHEICVSADAAMVEAGAPGDANAGGAAAAVERAAARQHEPAAAAPLPFATSAATG
APPAASPVAAAAAGASARSVPAWRFTWRPVPVDPVPDEPSPALSGKRIALVNGRGGTIER
VAAALARTCAKVSVFAPAANADHAEAARAFVNEAGPLDGIVDLGLEAPFALDDASAWEAP
MRRTVELLKACYGDWAAEDDTSRLFYVALTWMDGQMGYGDDAGAQPLGGLWAGLAKTLPQ
ELPNCNVRVLDIAPDETGRVDRLVVNELYRWGLFEVGYRNGVRYTLHAKRDELPPLAVDA
RAALGPGDTVLFSGGARGIGLLCARALAERHGCTVLVTGRETPPDGGEPWMALDEAGFKR
YGLEQLRAATPERPPKAIRASLQRLKHRRELKAALDEMAALGLPVHYRVCDVTDRAAVRA
LCDELGDSLRIVIHNAGVDQPVRLIQKRADDFAHTVRTKVLGFANLCDAVRARPRLAQFC
NVGSLTGRWGGMTGETDYAAANEALARLGLWAAKHALPPACGVKTLVWPTWEGVGMITNF
AVTQRYVSPMNVEEGVRHWLRELASPGGGEVMFMGAVGRAPTPVQIKGFQPIRELPNIGE

LVTRRHHVGEPVRFRPFKRFEGRYAIDPAVAPYARAFRFNGRAVLPASLVLEHACAVGDW
VMPPTGEPRELSEMTNVVLRLDALPDVPAGDAPVEIASEAAGYMIGDVWCVDVRCSDART
QAELLSMTLVYRPQPEQIGAAYARAPDTKPEPAPLTSAPHAAWDGELLPAGDWSVPDDAS
AGIVRIGRVPPAAVLSLWTLPFPLELRLPVNHVEHVLRVLLAEWGAAQTDAPSVWRIGAA
KLARLPAASADGVVEYATGRFAVLDARGYVLLRVDDTRVDSDAAHAAPAETVALADPLTA

>BTN_4810|KEGG|CYP1488A2|[Burkholderia thailandensis]bthe|BP

MNGLPPSLPRVDSTESLFAEPLAFLAQARSRHGDVFVMREHGPIFSRASDCSGVIAVFGE
HRLRQILTDIDNFALPMSAAAKMALPKNLVNLNRGLHSMREPEHGRHKRLLTGTINRELF
DAHRFEIRAALNRFCEMLKVDRRISVVSRMRELTVEMASHIFLGAQCQEDDELAFLLSAY
FTLRREASSLNARDPLLYRDELIGVGQQLDRTLRERIRRYRKRPVDARAGLLQRLATAGP
PGSPALSEDEIVGHANVMFVSSTEPVAMSLTWLLLVLSQLPDLRRALRAEIADRASMPAS
TNGASWLENVVNETLRLLTPNALMVRATTRAVSLQGVALPARCEIVVCPFLAHREAKPFP
DPHAFSPSRWETARPSPYEYFPFGAGGHFCAGRNLALSLIREVLSTLLSRFDFVLDGEQS
IDWRIHIMLMPKGDPALIAHPVDERGDTPSPKWRGPITDLFHFAPGLS

>BTN_5218|KEGG|CYP212A12|[Burkholderia thailandensis]bthe|BP

MSRDLPGTLAAWQKAYGDIVHLRIWPEHDVVVADPQLARALLVTHHDALIRWERGIRVLS
QVHGHSVLTAEGEAWRTRRHALVPNFSPKAVQGFSPTIVKAATRAFAQWPREDADWHIES
ALTMLTMDVILQMVFSSTLGEDVRVAEQAIRVASAAANAEFYWPASMPDWMPWKREKRQA
LRTLKDLIERHLQRRLKLACDAWPDDLLSCLLQSREDASALSLQNVRDECMTIFLAGHET
TAATLVWWAWCMASNPEAQRVARDEVQRVLQGNAPVAETLPELGYLVQTIKETLRRYPVA
PLLISRRATRAIQLGDWALPARTLFMIPAHLMHHDARWFPDPEAFRPERFAPDAPEVPRG
AYIPFGAGPRVCLGQHLAMAEMTVIAAMLLQRFVLSAPDGSAPPHPVLNVTLRPAQPMHL
AITQVDPRIA

>BTRA_5023|KEGG|CYP1013A3|[Burkholderia thailandensis]bthm|BP

MSKHRQAPGPRGSLVMGNLAAYKRNPITMLLRLHQQYGDVARNRLGPFVTHALAHPDHIQ
YVLQENHRNYVRGRFYDNFKMFFGDGLLTTDGEFWRRHRRAVQPLFHKKQVDAHTAAVGD
AALALAHRWSALPPGKAVDVVEEMMHLSLRMLGLMVFNTDVSSHAEAVGPAVRFGIEAMM

PQGNMNDFIPRWAPTRFNRRIAHARRAIDTIIAKIIADHREARCEPSDVISLLLNARDPD
TGAPMTQQEVHDEVMTVFLAGHETTGAGLAWALYALAQHPAVLRQLRDELDARLGGRAPT
VQDFEQLPYLSQVVDEVLRVYPPIWGFTRDLVEDDEIGGYRIPARSSVFMSPYVTHRHPA
FWRNPDAFDPENFASDAPARHRFVYFPFGGGMRKCIGFQTALLQMRVLVAVVAQHFDLNA
LPGHPIELGAAISLRPVHGIRLIVKPRERQQSHLARVREHESARALRPLGDTAEAAGAVC
PMTDAAHEICVSADAAMVEAGAPGDANAGGAAAAVERAAARQHEPAAAAPLPFATSAATG
APPAASPVAAAAAGASARSVPAWRFTWRPVPVDPVPDEPSPALSGKRIALVNGRGGTIER
VAAALARTCAKVSVFAPAANADHAEAARAFVNEAGPLDGIVDLGLEAPFALDDASAWEAP
MRRTVELLKACYGDWAAEDDTSRLFYVALTWMDGQMGYGDDAGAQPLGGLWAGLAKTLPQ
ELPNCNVRVLDIAPDETGRVDRLVVNELYRWGLFEVGYRNGVRYTLHAKRDELPPLAVDA
RAALGPGDTVLFSGGARGIGLLCARALAERHGCTVLVTGRETPPDGGEPWMALDEAGFKR
YGLEQLRAATPERPPKAIRASLQRLKHRRELKAALDEMAALGLPVHYRVCDVTDRAAVRA
LCDELGDSLRIVIHNAGVDQPVRLIQKRADDFAHTVRTKVLGFANLCDAVRARPRLAQFC
NVGSLTGRWGGMTGETDYAAANEALARLGLWAAKHALPPACGVKTLVWPTWEGVGMITNF
AVTQRYVSPMNVEEGVRHWLRELASPGGGEVMFMGAVGRAPTPVQIKGFQPIRELPNIGE
LVTRRHHVGEPVRFRPFKRFEGRYAIDPAVAPYARAFRFNGRAVLPASLVLEHACAVGDW
VMPPTGEPRELSEMTNVVLRLDALPDVPAGDAPVEIASEAAGYMIGDVWCVDVRCSDART
QAELLSMTLVYRPQPEQIGAAYARAPDTKPEPAPLTSAPHAAWDGELLPAGDWSVPDDAS
AGIVRIGRVPPAAVLSLWTLPFPLELRLPVNHVEHVLRVLLAEWGAAQTDAPSVWRIGAA
KLARLPAASADGVVEYATGRFAVLDARGYVLLRVDDTRVDSDAAHAAPAETVALADPLTA

>BTRA_5078|KEGG|CYP1488A2|[Burkholderia thailandensis]bthm|BP

MNGLPPSLPRVDSTESLFAEPLAFLAQARSRHGDVFVMREHGPIFSRASDCSGVIAVFGE
HRLRQILTDIDNFALPMSAAAKMALPKNLVNLNRGLHSMREPEHGRHKRLLTGTINRELF
DAHRFEIRAALNRFCEMLKVDRRISVVSRMRELTVEMASHIFLGAQCQEDDELAFLLSAY
FTLRREASSLNARDPLLYRDELIGVGQQLDRTLRERIRRYRKRPVDARAGLLQRLATAGP
PGSPALSEDEIVGHANVMFVSSTEPVAMSLTWLLLVLSQLPDLRRALRAEIADRASMPAS
TNGASWLENVVNETLRLLTPNALMVRATTRAVSLQGVALPARCEIVVCPFLAHREAKPFP
DPHAFSPSRWETARPSPYEYFPFGAGGHFCAGRNLALSLIREVLSTLLSRFDFVLDGEQS
IDWRIHIMLMPKGDPALIAHPVDERGDTPSPKWRGPITDLFHFAPGLS

>BTRA_5488|KEGG|CYP212A12|[Burkholderia thailandensis]bthm|BP

MSRDLPGTLAAWQKAYGDIVHLRIWPEHDVVVADPQLARALLVTHHDALIRWERGIRVLS
QVHGHSVLTAEGEAWRTRRHALVPNFSPKAVQGFSPTIVKAATRAFAQWPREDADWHIES
ALTMLTMDVILQMVFSSTLGEDVRVAEQAIRVASAAANAEFYWPASMPDWMPWKREKRQA
LRTLKDLIERHLQRRLKLACDAWPDDLLSCLLQSREDASALSLQNVRDECMTIFLAGHET
TAATLVWWAWCMASNPEAQRVARDEVQRVLQGNAPVAETLPELGYLVQTIKETLRRYPVA
PLLISRRATRAIQLGDWALPARTLFMIPAHLMHHDARWFPDPEAFRPERFAPDAPEVPRG
AYIPFGAGPRVCLGQHLAMAEMTVIAAMLLQRFVLSAPDGSAPPHPVLNVTLRPAQPMHL
AITQVDPRIA

>DR62_5346|KEGG|CYP212A12|[Burkholderia thailandensis]btha|BP

MTMLPEHQCPFHRNSPTQARPVGSSGRWPPGPEAGLTGWRLLGAMSRDLPGTLAAWQKAY
GDIVHLRIWPEHDVVVADPQLARELLVTHHDALIRWERGIRVLSQVHGHSVLTAEGEAWR
TRRHALVPNFSPKAVQGFSPTIVKAATRAFAQWPREDADWHIESALTMLTMDVILQMVFS
STLGEDVRLAEQAIRVASAAANAEFYWPASMPDWMPWKREKRQALRTLKDLIERHLQRRL
KLACDAWPDDLLSCLLQSREDASALSLQNVRDECMTIFLAGHETTAATLVWWAWCMASNP
EAQRVARDEVQRVLQGNAPVAETLPELGYLVQTIKETLRLYPVAPLLISRRATRAIQLGD
WSLPARTLFMIPVHLMHHDARWFPDPEAFRPERFAPDAPEVPRGAYIPFGAGPRVCLGQH
LAMAEMTVIAAMLLQRFVLSAPDGSAPPHPVLNVTLRPAQPMHLAITQVDPRIA

>DR62_5680|KEGG|CYP116B128|[Burkholderia thailandensis]btha|BP

MTHSASTPPALPAAQCPLHPVSDDAAAHPAGCPASAGAAAFDPFDDAYQQDPPEYVRWAR
EQEPVFYSPKLGYWIVTRYDDIKAIFRDNRTFSPSIALEKITPTGPDANAALASYGYAMN
RTLVNEDEPAHMARRRLLIEPFTPDALRAHEPMVRRLAREYVDRFVDDGRADLVDQMLWE
VPLTVALHFLGVPEEDMDTLRKYSIAHTVNTWGRPRPDEQVAVAHAVGQFWRYAGKVLEK
MREDPSGPGWMQFGIRMQREHPDIVTDSYLHSMMMAGIVAAHETTANATANALKLLLQHP
RAWRELCDDPSLIPNAVDECLRHNGPIAAWRRIATCDATVGGVAIPAGAKLLIVTSSANR
DERRFADADLFDIHRDDCAEHLTFGYGAHQCMGKHLARMEMQIFIDELTRRLPHMRLAEQ
RFTYVPNTSFRGPEHLWVEWDPAANPERRRADARRPALAVRIGESNARGAGRALRVDSMT
PVAERVLRVRLVAPGGEPLPRWSAGAHIDIECGDTGLSRQYSLCGDPAERAAYEIAVLRE
PASRGGSAWMHERLRPGMRVKIRGPRNHFRLDAHASRAILIAGGIGITPIGAIARQARAG

GIDYTLHYCGRSRRTMAFVDELAALHGERLRLHVSDEGTRADFSALDVAPGTQVYACGPA
RMLDALAARAAHWPAHALRVEHFAAAAPRVDPAAEQPFEVELKDSGLTLTVPANRTLLAT
LRAANVDLQSDCEEGLCGSCEVRVLAGEIDHRDSVLSAGERAANTRMMACCSRAKRGRIV
LEL

>DR62_4913|KEGG|CYP1488A2|[Burkholderia thailandensis]btha|BP

MNGLPPSLPRVDSTESLFAEPLAFLAQARSRHGDVFVMREHGPIFSRASDCSGVIAVFGE
HRLRQILTDIDNFALPMSAAAKMALPKNLVNLNRGLHSMREPEHGRHKRLLTGTINRELF
DAHRFEIRAALNRFCEMLKVDRRISVVSRMRELTVEMASHIFLGAQCQEDDELAFLLSAY
FTLRREASSLNARDPLLYRDELIGVGQQLDRTLRERIRRYRKRPVDARAGLLQRLATAGP
PGSPALSEDEIVGHANVMFVSSTEPVAMSLTWLLLVLSQLPDLRRALRAEIADRASMPAS
TNGAPWLENVVNETLRLLTPNALMVRATTRAVSLQGVALPARCEIVVCPFLAHREAKPFP
DPHAFSPSRWETARPSPYEYFPFGAGGHFCAGRNLALSLIREVLSTLLSRFDFVLDGEQS
IDWRIHIMLMPKGDPALIAHPVDERGDTPSPKWRGPITDLFHFAPGLS

>DR62_07990|KEGG|CYP1013A3|[Burkholderia thailandensis]btha|BP
MPVDPVPDEPSPALSGKRIALVNGRGGTIERVAAALARTCAKVSVFAPAANADHAEAARAFVNEAGPLDGIVDLGLEAPFALDDASAWEAPMRRTV
ELLKACYGDWAAEDDTSRLFYVALTWMDGQMGYGDAAGAQPLGGLWAGLAKTLPQELPNCNVRVLDIAPDETGRVDRLVVNELYRWGLFEVGYRNG
VRYTLHAKRDELPPLAVDARAALGPGDTVLFSGGARGIGLLCARALAERHGCTVLVTGRETPPDGGEPWMALDEAGFKRYGLEQLRAATPERPPKA
IRASLQRLKHRRELKAALDEMAALGLPVHYRVCDVTDRAAVRALCDELGDSLRIVIHNAGVDQPVRLIQKRADDFAHTVRTKVLGFANLCDAVRAR
PRLAQFCNVGSLTGRWGGMTGETDYAAANEALARLGLWAAKHALPPACGVKTLVWPTWEGVGMITNFAVTQRYVSPMNVEEGVRHWLRELASPGGG
EVMFMGAVGRAPTPVQIKGFQPIRELPNIGELVTRRHHVGEPVRFRPFKRFEGRYAIDPAVAPYARAFRFNGRAVLPASLVLEHACAVGDWVMPPT
GEPRELSEMTNVVLRLDALPDVPAGDAPVEIASEAAGYMIGDVWCVDVRCSDARTQAELLSMTLVYRPQPEQIGAAYARAPDTKPEPAPLTSAPHA
AWDGELLPAGDWSVPDDASAGIVRIGRVPPAAVLSLWALPFPLELRLPVNHVEHVLRVLLAEWGAAQTDAPSVWRIGAAKLARLPAASADGVVEYA
TGRFAVLDARGYVLLRVDDTRVDSDAAHAAPAETVALADPLTA

>BG87_5090|KEGG|CYP1013A3|[Burkholderia thailandensis]bthl|BP
MSKHRQAPGPRGSLVMGNLAAYKRNPITMLLRLHQQYGDVARNRLGPFVTHALAHPDHIQ
YVLQENHRNYVRGRFYDNFKMFFGDGLLTTDGEFWRRHRRAVQPLFHKKQVDAHTAAVGD
AALALAHRWSALPPGKAVDVVEEMMHLSLRMLGLMVFNTDVSSHAEAVGPAVRFGIEAMM
PQGNMNDFIPRWAPTRFNRRIAHARRAIDTIIAKIIADHREARCEPSDVISLLLNARDPD
TGAPMTQQEVHDEVMTVFLAGHETTGAGLAWALYALAQHPAVLRQLRDELDARLGGRAPT

VQDFEQLPYLSQVVDEVLRVYPPIWGFTRDLVEDDEIGGYRIPARSSVFMSPYVTHRHPA
FWRNPDAFDPENFASDAPARHRFVYFPFGGGMRKCIGFQTALLQMRVLVAVVAQHFDLNA
LPGHPIELGAAISLRPVHGIRLIVKPRERQQSHLARVREHESARALRPLGDTAEAAGAVC
PMTDAAHEICVSADAAMVEAGAPGDANAGGAAAAVERAAARQHEPAAAAPLPFATSAATG
APPAASPVAAAAAGASARSVPAWRFTWRPVPVDPVPDEPSPALSGKRIALVNGRGGTIER
VAAALARTCAKVSVFAPAANADHAEAARAFVNEAGPLDGIVDLGLEAPFALDDASAWEAP
MRRTVELLKACYGDWAAEDDTSRLFYVALTWMDGQMGYGDDAGAQPLGGLWAGLAKTLPQ
ELPNCNVRVLDIAPDETGRVDRLVVNELYRWGLFEVGYRNGVRYTLHAKRDELPPLAVDA
RAALGPGDTVLFSGGARGIGLLCARALAERHGCTVLVTGRETPPDGGEPWMALDEAGFKR
YGLEQLRAATPERPPKAIRASLQRLKHRRELKAALDEMAALGLPVHYRVCDVTDRAAVRA
LCDELGDSLRIVIHNAGVDQPVRLIQKRADDFAHTVRTKVLGFANLCDAVRARPRLAQFC
NVGSLTGRWGGMTGETDYAAANEALARLGLWAAKHALPPACGVKTLVWPTWEGVGMITNF
AVTQRYVSPMNVEEGVRHWLRELASPGGGEVMFMGAVGRAPTPVQIKGFQPIRELPNIGE
LVTRRHHVGEPVRFRPFKRFEGRYAIDPAVAPYARAFRFNGRAVLPASLVLEHACAVGDW
VMPPTGEPRELSEMTNVVLRLDALPDVPAGDAPVEIASEAAGYMIGDVWCVDVRCSDART
QAELLSMTLVYRPQPEQIGAAYARAPDTKPEPAPLTSAPHAAWDGELLPAGDWSVPDDAS
AGIVRIGRVPPAAVLSLWTLPFPLELRLPVNHVEHVLRVLLAEWGAAQTDAPSVWRIGAA
KLARLPAASADGVVEYATGRFAVLDARGYVLLRVDDTRVDSDAAHAAPAETVALADPLTA

>BG87_5145|KEGG|CYP1488A2|[Burkholderia thailandensis]bthl|BP

MNGLPPSLPRVDSTESLFAEPLAFLAQARSRHGDVFVMREHGPIFSRASDCSGVIAVFGE
HRLRQILTDIDNFALPMSAAAKMALPKNLVNLNRGLHSMREPEHGRHKRLLTGTINRELF
DAHRFEIRAALNRFCEMLKVDRRISVVSRMRELTVEMASHIFLGAQCQEDDELAFLLSAY
FTLRREASSLNARDPLLYRDELIGVGQQLDRTLRERIRRYRKRPVDARAGLLQRLATAGP
PGSPALSEDEIVGHANVMFVSSTEPVAMSLTWLLLVLSQLPDLRRALRAEIADRASMPAS
TNGASWLENVVNETLRLLTPNALMVRATTRAVSLQGVALPARCEIVVCPFLAHREAKPFP
DPHAFSPSRWETARPSPYEYFPFGAGGHFCAGRNLALSLIREVLSTLLSRFDFVLDGEQS
IDWRIHIMLMPKGDPALIAHPVDERGDTPSPKWRGPITDLFHFAPGLS

>BG87_5559|KEGG|CYP212A12|[Burkholderia thailandensis]bthl|BP

MSRDLPGTLAAWQKAYGDIVHLRIWPEHDVVVADPQLARALLVTHHDALIRWERGIRVLS

QVHGHSVLTAEGEAWRTRRHALVPNFSPKAVQGFSPTIVKAATRAFAQWPREDADWHIES
ALTMLTMDVILQMVFSSTLGEDVRVAEQAIRVASAAANAEFYWPTSMPDWMPWKREKRQA
LRTLKDLIERHLQRRLKLACDAWPDDLLSCLLQSREDASALSLQNVRDECMTIFLAGHET
TAATLVWWAWCMASNPEAQRVARDEVQRVLQGNAPVAETLPELGYLVQTIKETLRRYPVA
PLLISRRATRAIQLGDWALPARTLFMIPAHLMHHDARWFPDPEAFRPERFAPDAPEVPRG
AYIPFGAGPRVCLGQHLAMAEMTVIAAMLLQRFVLSAPDGSAPPHPVLNVTLRPAQPMHL
AITQVDPRIA

>BG90_1030|KEGG|CYP266A2|[Burkholderia oklahomensis]boc|BP

MDSAAVNKEEAVALNILAPENLRNPYPMFQYLLSEAPLFFDENSKFYVVSRHEDIERLMK
DPRVSADRYTTLAADTPAEDRDMNDAIVRYLSMFLLNLEGERHTRLRNLTNKSFLPKHME
SLGPFAQEVTDELLDAVEKKGELEIISDLAFPMPIRFICRILGLPNADVQLIKTLSDYVS
VYVGSAGKAPGCIPLAHRGFTELADLFYPVVQERRKNPTDDLVSSLINVRIGDDVLSDEE
VVANCILFLVSGFETTTNLIAAGTLALLEHPDQLSLLKNDPTLIDGAIEEMLRFYPPVNR
TARVLTDDVELHGVTLKKGQIIILMLGAGNRDPRVFAHPDSFDIWRPRTGKILSFGSGHH
FCVGSHLARLEAKIAIQSLLHRFPALKLNHESISWRGESRFRGLQSMTISL

>BG90_4037|KEGG|CYP1013A3|[Burkholderia oklahomensis]boc|BP

MSKHRQAPGPRGSLVMGNLAAYKRNPITMLLRLHQQYGDVARNRLGPFITHALAHPDHVQ
YVLQENHRNYVRGRFYDNFKMFFGDGLLTTDGEFWRRHRRAVQPLFHKKQVDAHTAAVGD
AALALAHRWSALPPGKAVDVVEEMMHLSLRMLGLMVFNTDVSTHAEAVGPAVRFGIEAMM
PQGNMNDFIPRWAPTRFNRRIAHARRAIDTIIAKIIADHRDERCEPSDVISLLLNARDPD
TGAPMTQQEVHDEVMTVFLAGHETTGAGLAWALYALAQHPAVLRQLRDELDARLGGRAPT
VQDFEQLPYLSQVVDEVLRVYPPIWGFTRDLVEDDEIGGYRIPARSSVFMSPYVTHRHPA
FWRNPDAFDPENFASDAAARHRFVYFPFGGGMRKCIGFQTALLQMRVLVAVIAQHFDLNV
LPGHPIELGATISLRPMHGIRLIVKPRERQQSHLARVREHEAARALRPLGDTIEPTGAAC
PMAAAGASAAAGADAAAGADAAAESAQALDVAHASGAAVADERAAARQHADAARLSLATP
SDAAADASARGVPAWRFTWRPAPVDPVPDAPSPALSGKRIALVNGRGGTVERIAAALART
CAKVSVFAPAANADDAEAARTFVNEAGPLDGIVDLGLEAPFALDDASAWEAPMRRTVELL
KACYGDWAAEDDTSRLFYVALTWMDGMMGYGDAAAGAQPLGGLWAGLAKTLPQELPNCNV

RVLDISPDETGRVDRLVVNELYRWGLFEVGYRRGVRYTLHARRNELPPLAVDARTTLTAG
DTVLFSGGARGIGLLCARALAERHGCAVIVTGREAPPDGSEPWMALDDAGFKRYSLEQLR
AATPDNPPKAIRATLQRLKRRRELKAALDEMAALDLPVRYRVCDVTDRAAVRALCDELGD
SLRMVIHNAGVDQPVRLIQKGADDFARTVRTKVLGFANLCDAVRARPRLVQFCNVGSLTG
RWGGMTGETDYAAANEALARLGLWAAKHVLPPACGVKTLVWPTWEGVGMITNFSVTQRYI
TPMNVDEGVRHWLRELASPGSGEVMFMGAVGRAPTPVQIKGFQPIRELPNIGELVTRRHH
VGEPLRFRPFKRFEGRYAIDPAVAPYAYAFRFNGRAVLPASLVLEHACAVGDWVMPPTGE
PRDLSEMTNVVLRLDALPDVPNGGAPVEIGSEAAGYMIGDVWCVDVRCSDARTQTELLSM
TLVYRPEPEQIGAAYARPPGATPEPPRLTSAPHAAWNGDLLPAGDWSVPDDASAGIVRIG
RVPPAAVLSLWTLPFPLELRLPVNHVEHVLRVLLAEWGAAQSDAPAVWRIGSAKLARLPA
SSADGVVEYATGRFAVVDARGYVLLRLDDTRVDAARTMQPEAGALADSMTA

>WS70_22015|KEGG|CYP117C2|[Burkholderia mayonis]buu|BP

MLLNPLNRRVREQQGMPVMAGAFPVLGHLPAVMSSLTDLLRYAEQRHGTHFWLEMGTFGR
MLVCTQPEAFALFKNKHVLTDLLGEVAPPLKDTMATQDGDSHRSTRAWANPAFQPQGLTG
AKVGDLFADIMLARIGQWPVSAPIRLVDEVSDMSLTMIFRMLGVDEQAIAEWTRFYSDYF
LVSAWPVDLPGTRFRRSRHAQTWIDANLQTLIDDARARPDAGGLLAMLVHSIVSSNVALT
DASLLANLRALIHAAHKTTAVTMAQLVIKIAQHPEYWALLCAEAKSIGEVPRNPANLVSF
PYAEALFRETLRYHPAFPLALRRTEEAIDLGGRSIEAGTRFIIPLVLLSRNAELYPQPDV
FMPGRWMGRNEGLKPIDTMQFGNGPHRCLGYHTAWMQLVQFAVALALTLSARGVRPRLVS
SDAGRHRFFPLTYPPRTTRIAFE

>AOJ05625.1|NCBI|CYP1013A3|[Burkholderia mayonis]buu|BP

MSKHRQAPGPRGSLVMGNLAAYKRNPITMLLRLHQQYGDVARNRLGPFVTHALAHPDHVQ
YVLQENHRNYVRGRFYDNFKMFFGDGLLTTDGEFWRRHRRAVQPLFHKKQVDAHTAAVGD
AALALAHRWSALPPGKSIDVVEEMMHLSLRMLGLMVFNTDVSTHAEAVGPAVRFGIEAMM

PQGNMNDFIPRWAPTRFNRRIAHARRAIDTIIAKIIADHRDERCEPSDVISLLLNARDPD
TGAPMTQQEVHDEVMTVFLAGHETTGAGLAWALYALAQHPAVLRQLRDELDARLGGRAPT
VQDFEQLPYLSQVVDEVLRVYPPIWGFTRDLVEDDEIGGYRIPARSSVFMSPYVTHRHPA
FWRNPDAFDPENFASEAAARHRFVYFPFGGGMRKCIGFQTALLQMRVLVAVVAQHFDLNA
LPGHPIELGATISLRPVHGIRLVVKPRERQQSHLARVREHEAARALRPLGDTVEPTGAAC
PMAAAGEIGASAAAGAAAESAQALDVAHASGPAVADERAANRQHADAARLSLATPSTAAP
RDAAADASARGVPAWRFTWRPAPVDPVPDAPSPALSGKRIALVNGRGGTVERITAALART
CTKVSVFAPAANADYAEAARTFVNEAGPFDGIVDLGLEAPFTLDDASAWEAPMRRTVELL
KACYGDWAAEDDTSRLFYVALTWMDGMMGYGDAAAGAQPLGGLWAGLAKTLPQELPNCNV
RVLDIAPDETGRVDRLVVNELYRWGFFEVGYRQGVRYTLHARRNELPPLAVDARASLAPG
DTVLFSGGARGIGLLCARALAERHGCTVIVTGREASPDGSEPWMALDDEGFKRYGLEQLR
AATPDNPPKAIRAMLQRLKRRRELKAALDEMAALDLPVRYRVCDVTDRAAVRALCDELGD
SLRMVIHNAGVDQPVRLIQKGADDFARTVRTKVLGFANLCDAVRTRPRLVQFCNVGSLTG
RWGGMTGETDYAAANEGLARLGLWAAKHALPPACGVKTLVWPTWEGVGMITNFAVTQRYI
TPMNVDEGVRHWLRELASPGSGEVMFMGAVGRAPTPVQIKGFLPIRELPNIGELVTRRHH
VGEPLRFRPFKRFEGRYAIDPAVAPYAHAFRFNGRAVLPASLVLEHACAVGDWVMPPTGE
PRDLSEMTNVVLRLDALPDVSNGGAPVEIGSEAAGYMIGDAWCVDVRCSDARTQAELLSM
TLVYRTEPEQIGAAYARPPDATLEPSRLTSAPHAAWNGDLLPAGDWSVPDDASAGIVRIG
RVPPAAVLSLWTLPFPLELRLPVNHVEHVLRVLLAEWGAAQSDAPAVWRIGGAKLARLPA
SSADGVVEYATGRFAMIDARGYVLLRLDDTRVDAAQATQPEAGALADPLTA

>Bcep1808_5445|KEGG|CYP229F5|[Burkholderia vietnamiensis]bvi|BP

MNPTDPIAAVTHRDPYPYYATLVDGPPLAFDATLGLWVASRAASVAAVLGHPACRVRPLD
APVPPALRGTTAGALFGDLIRMNDGALRHDVPKQALRAAFASIDAHALRERTASLAGRRL
RAPLDAEALNAWCLTVPVCAVADLLGFDEAQLDDIAALVVAFVAALSPLSDDAALARASD
AARHLLERMTERVARTRARDGSLVAAVQDAARAAGWQAGGALVANLVGLLSQTCEASAAW
LGNVLIAWEESARASADGGRAIPDAAALDAFVAEVGRFDSPVQNTRRFVASHTTIEGVSV
DAGAAILVVLAAANRDPAVHREPHRLLPGRAPAPTFGFGTGPHGCPGERIARAVTTGAFD
AWLHASSGELPSGLVWDYRASTNIRMPNFGIASQA

>AK36_3459|KEGG|CYP229F5|[Burkholderia vietnamiensis]bve|BP

MNPTDPIAAVTHRDPYPYYATLVDGPPLAFDATLGLWVASRAASVAAVLGHPTCRVRPLD
APVPPALRGTTAGALFGDLIRMNDGALRERTASLAGRRLRAPLDAEALNAWCLTVPVCAV
ADLLGFDEAQLDDIAALVVAFVAALSPLSDDATLARASDAARRLLERMTERVARTRARDG
SLVAAVQDAARAAGWQAGGALVTNLVGLLSQTCEASAAWLGNALIAWEESARASADGGRA
IPDAAALDAFVAEVGRFDSPVQNTRRFVASHTTIEGVSVDAGAAILVVLAAANRDPAVHR
EPHRLLPGRAPAPTFGFGKGPHGCPGERIARAVTTGAFDVWLHASSGELPSGLVWDYRAS
TNIRMPNFGIASQA

>Bcep18194_B0775|KEGG|CYP229F2|[Burkholderia lata]bur|BP

MNPTDPIAAVTHRDPYPYYAALVDGPPLAFDATLGLWVASRAAAVTAVLGHPACRVRPLD
APVPPALRGTTAGALFGELVRMNDGALRHDVPKQALRTAFAPIDTVALRDRVTQLAARRL
PTPGDAGALNAWCMTVPVCAVADLLGFDDTQLDDIAGLVVDFVAALSPLSDAVALARASD
AARQLLDRMTARVAQSHAHDGTLVAAVQQAARVSGWQASGGLVANLVGLLSQTCEATAAW
LGNTLVAWSGDASASAARVVPDDAALDAFVAEVGRFDSPVQNTRRFVASRTTIEGVTVEA
GDAILVVLAAANRDPAVHRDPHRFMPGRAAGPNFGFGTGPHGCPGERIARAVTAGAFGAW
LRAGGVAPHDGLAWDYRASTNVRMPKFDAAR

>Bcep18194_B1689|KEGG|CYP212A7|[Burkholderia lata]bur|BP

MSRDLLGTLAGWQRTYGDVVHLRVRPEHAVVVTDPALVRELLVTHHDALGRWERAIRVFA
QVHGHSVLVAEGDAWRDKRQALQPNFMPKPVQAFVPSIAATAGHALAQWPARDPDWPIES
ALTSLAMDVIMRTMFSDAIGDDARAAEEAVRTVSAAANADFYWPASTPDWVPWKRGKARA
MAVLNGLIDRQLHARLDLPARAWPDDLLSRLLRLHRDDARRWPLQAVHDECMTAFLAGHE
TTAGALTWWAWCMATNPAAQAAARDEVSRVLGGCAPSAETRSSLRYLTHTLEETLRLYPS
APILISRRASRPIVLGPWQFPARTLFMLPLQLMQRDARWFAEPDVFRPERFADGAPAAPR
GTYMPFGTGPRVCLGQHLAMTEMTVVAAMILQRYELSAQPGMMPPRPVLNVTLRPERLLH
LAIAPAADGGTV

>Bcep18194_C6803|KEGG|CYP102J1|[Burkholderia lata]bur|BP

MKSSSLVPQPPLKPVIGHLMEVLGPSPLAKMMDLARTYGPVYWFEVFGQGYYVVSGQTLV
DEVCDETRFQKCVHQSLLELRPAIGDGLFTAFGDEPNWAKAHRVLMQAFGPLSIWSMFDK
MVDIADQMFLHWERFGPETPVDVSDHMTRLTLDTIALCAFDCRFNSFYREDQHPFVDAMV
NTLSEAGKRELRPKLVSKLMVKRSRQFDADIEVMRSLATKMIEDRRKNPHVNEESMDLLD
RMLNGIDPVTGEKLDDENIVFQMITFLIAGHETTSGLLSFATYFLLKNPDILQKARDMVD
EVVGSETPRIEHLARLRYVEQILMETLRIWPTAPGFAVKPLADTTFGGKYAVSPDDIIMI
LTPMLHRDVSVWGEDVEAFRPERFAPENAEQLPPNSWKPFGNGARACIGRPFAMQEAHLV
LIMLLQRFDFSFADPDYELDVAETLTLKPAGFRVNVKPRARGALKVPDTALHARSNAQSP
SVAPVQSIAPGEDLSNLLVLFGGNTGSAESFARRIAGDASRHGFHATCAPLDDFAGKLGG
YPAIVIVTASYEGQPPDNAKSFVPYVEALDEGALDGVHFSVLGCGNKQWARTYQAIPKRV
DEALEKAGATRVHFRGELDSGGDFFGEFDRWYTEMWNRFAISAGKEIPVIQHDDVALKVS
FAGSSREKVLNLGDMAHASIVDNRELVDISVAGSRSKKHIELKLPEAMTYRSGDYLAVLP
RNAKNNVDRVLRRFRVSWDTQVVIEGTSSNPRLPLGQAIGCGELFSSYVELAVPATRSQV
SSLAAATRCPPEKVELERLSADGFECEILGKRTTVMDLLERFGSVDLSLEKFLDMLPALK
ARQYSISSSPLWKADHVTLTVAVVDAPALSGNGRHEGVASSYLARLNTGDSLSVAVRPSN
ARFRPPAEPDLPMILICAGSGIAPFRGFLQERALQKQRGENVGTSLLFFGIDDPDVDFLY
RDELDEWARCGVVEVMPAYSNRPEEGARFVQDKVWLERERISALFSQGATVFVCGDGKNM
APAVRATLGRIYQETTGENDESASAWIDTMEREHGRYVADVFA

>Bcep18194_C7583|KEGG|CYP1135B1|[Burkholderia lata]bur|BP

MKHRIDTYRDAEKTMKDKTLRQAMYDPALFFMKDTVVNLHGDAHLRKRQALNSLFTREFF
RDYQNRVLPAALDQVLKPVVAEGRGDVAILAFQALVNLTSDVTGIDRVGTEEETNRILKI
IRGLAHVSTVEQLLSGSVDDANRKIREALVEFERDFYVPSARRRQALIDQGAELPKDVMT
VMLVNYPNGELPHDVMVKDAALFLLASAATQANTLCFIMLDILTWCRDHPGTRETLLAEP
ATLQKFIWEAIRLHPPQVVSLRRASCPMHIPDGGEVAAGDVIEFDMATANRNPDIFGPDA
DTFNPYRVPPGRIAAPGLSFGAGPHACVGRVLAAGVPITAENADNEDTEFGTLHMITHAL
LELGVDLDRDHPPIVDEATVRRHIVSLPFVLRPELAAAAGREVAGGGCPFTRS

>Bcen_3450|KEGG|CYP229F2|[Burkholderia cenocepacia]bcn|BP

MNPTDPIAAVTHRDPYPYYAALVDGPPLAFDATLGLWVASRAAAVTAVLCHPACRVRPLD
APVPPALRGTTAGALFGELVRMNDGASRHDVPKQALRAAFAPIDTEALRDRAEVLAARRL
PAPGDADALNAWCMTVPVCAVADLLGFDETQLDDIAARVVEFVAALSPLSDAAALARASD
AARHLLDRMTERVAQSRAHDGTLVAAVQQADRASGWHASGALVANLVGLLSQTCEATAAW
LGNALVAWNGDVGDSGGVPDAVALDAFVAEVGRFDTPVQNTRRFVAARTTIEGVSVEAGD
AILVVLAAANRDPAVHRDPHRLTPGRPAGPNFGFGTGPHGCPGERIARAVTAGAFAARLR
AGGWPPRDALKWDYRASTNVRMPKFDANR

>Bcen2424_4916|KEGG|CYP229F2|[Burkholderia cenocepacia]bch|BP

MNPTDPIAAVTHRDPYPYYAALVDGPPLAFDATLGLWVASRAAAVTAVLCHPACRVRPLD
APVPPALRGTTAGALFGELVRMNDGASRHDVPKQALRAAFAPIDTEALRDRAEVLAARRL
PAPGDADALNAWCMTVPVCAVADLLGFDETQLDDIAARVVEFVAALSPLSDAAALARASD
AARHLLDRMTERVAQSRAHDGTLVAAVQQADRASGWHASGALVANLVGLLSQTCEATAAW
LGNALVAWNGDVGDSGGVPDAVALDAFVAEVGRFDTPVQNTRRFVAARTTIEGVSVEAGD
AILVVLAAANRDPAVHRDPHRLTPGRPAGPNFGFGTGPHGCPGERIARAVTAGAFAARLR
AGGWPPRDALKWDYRASTNVRMPKFDANR

>Bcenmc03_5370|KEGG|CYP229F2|[Burkholderia cenocepacia]bcm|BP

MNPTDPIAAVTHRDPYPYYAALVDGPPLAFDATLGLWVASRAAAVTAVLCHPACRVRPLD
APVPPALRGTTAGALFGELVRMNDGASRHDVPKQALRASFAPIDTDALRDRAEVLAARRL

PASGDADALNAWCMTVPVCAVADLLGFDETQLDDIAARVVEFVAALSPLSDAAALARASD
AARHLLDRMTERVAQSRAHDGTLVAAVQQAARVSGWHASGALVANLVGLLSQTCEATAAW
LGNVLVAWNGDVGDSGGVPDAAALDAFVAEVGRFDTPVQNTRRFVAARTTIEGVSVEAGD
AILVVLAAANRDPAVHRDPHRLTPGRPAGPNFGFGTGPHGCPGERIARAVTAGAFAARLR
AGGWPPRDALKWDYRASTNVRMPKFDANR

>Bcenmc03_5892|KEGG|CYP1484A1|[Burkholderia cenocepacia]bcm|BP

MTPMFDETLAHKTLFALLTDATDDRDAFYDAMPALPGAYRDVSGTWMVGRYRLACAVLDH
PALTTRSPLVECDVVRDEAGLLDTMLFADDSMHERMRKTFAPLFAKPRLAALRTHLASDL
DALLEQIPDPRRFDLVEHLAQRLPALAACRLLGIDPVHAPRLQAQSMGPIRLISAMPIGP
ADHALAVAQTRRFVDELDVHLDTVCAVLQAGGTRPALARRQLLANVLLTFIAGYGTTMLS
LGNTFAQALARRDLWQALVQEPRRVPLALRELMRIEPAVHVMIRFARADVELDGLRMAKG
DVVAVVAAAANRDPGEFAAPGDIRLDRAHGGLVFGAGSHGCLGAALARMQLAVVFETLLA
RMPGLMLEPGAHPRLQEGAFRGYRSLRVYDAARA

>Bcenmc03_6858|KEGG|CYP101N1|[Burkholderia cenocepacia]bcm|BP

MSAVGHGATPEQAPVPSHVSPERVFDIDIYHPPGAEGDYHLSLKKLHAEGIPDIFWTPRN
GGHWVATRGDDIYNILKDYENFSSKQLTVPLVEHQPLPPIFFDPPQHTAYRALIAPAFMP
QAIGKLEEKARELAIELIEDFYPAGEVEFVSRFAQHLPIAIFMNMVALPAEDREYLLGLA
DKMVRPVDANEKLAALGAIFQYLGAKIAERRANPGDDLISKIVNSTIDGRALTDDEVRGL
CGLVLIGGLDTVASAMGFIANFLAGSPAHRKQLIDNPALTQKAVDELLRRFPVVNQGRRI
THDFEYKGVQLKAGDMIILPTTLHGLDERKFEDPLTVDFSRTPPIHSTFGNGPHRCPGSF
LARVELKVFLQEWLKRIPDFQVKPGAKAGVHGGVNGTLYYLPLVWKVTR

>Bcenmc03_6889|KEGG|CYP1111A3|[Burkholderia cenocepacia]bcm|BP

MAGALRLEDFDDSTFNPFFEDSLAYGSDINPYPRIAELREKGTVQEGDYRVLMGGAADLT
MSDVAHYCVFGYDEVSHVLGDPATFSNKSYERNLGISFGRSISTMDAPEHPRYRRIFQKA
FLPHTVAAWGDTLVDPVVSELMSKFVDRGHADLVNEFTLHYPFNIIYRQLALPPEDVKIF
HKLAIAQTLVSIDVAHGMEASTKLGAYFAKLIDDRRRTPGDDLVSMLATAEVEGERLPDE
VVISFLRQLVNAGGDTTYRATSVLLLALLRNPEQLEAVRNDRTLVAQAIEEALRWDGPVL

MQSRMATRDVTLGGVEIPAGAFLDVVAGSANRDPSRFPDPDKFDIYRKPAHRHFAFAYGP
HVCIGQHLARVEMTRALNAILDRLPNLRLDPGAPAPEINGIMMRVPHSIPVVFG

>Bcenmc03_6896|KEGG|CYP101F1|[Burkholderia cenocepacia]bcm|BP

MEGDTKMNRAKDWTVPDHVPRELVVDFDYMAPPGAQDDVHLAWKSLHAGPDIVWSPYYGG
HWIATRGEDIEAMQKDHTRFSHRNVNIPPNNEMQIVPLELDPPEHTAYRNLITPAFLPQA
IASLENDVRSLAVELLDKIAPRGECEFVAEFAKILPIVIFLRLAELPVSDREQLLEWAEW
AVRGDFERRNDSQRALVGYISTWVEKRKADAGGDLVSLIANAQIEGKLISQERMFGMFIV
VLFGGLDTVASMMGFIARFLAENPAHRRQLIENPELMVSSVDELIRRFGVANTARLITQD
MEYKGIRFRKGDQIQLPNALHGLDDRKFSNPLAVDLTRKPVIHAAFGNGPHRCPGSFLAR
TEIKVFLQEWLKRIPDFHIREGDKPRCGSGSVNGMLYLPLAWDVAP

>BCAM2153|KEGG|CYP229F2|[Burkholderia cenocepacia]bcj|BP

MNPTDPIAAVTHRDPYPYYAALVDGPPLAFDATLGLWVASRATAVTAVLGDPACRVRPLD
APVPPALRGTTAGALFGELVRMNEGASRHDVPKQVLRAAFAPLDPAALRDRAEVIAARRL
SAPGDADALNAWCMTVPVCAVAELLGFDETQLDDIAARVVEFVAALSPLSDAAALARASD
AARQLLDRMTERVAQSHAQDGTLVAAVQQAARASGWQAGGALVANLVGLLSQTCEATAAW
LGNALVAWNGEAREVPDAAAIDAFVAEVGRFDSPVQNTRRFVAARTTIEGVSVEAGDAIL
VVLAAANRDPAVHREPHRLMPGRTAGPNFGFGTGPHGCPGERIARAVTAGAFAARLHAGG
WPPRDALKWDYRASTNVRMPKFDASR

>BCAM2591|KEGG|CYP116B109|[Burkholderia cenocepacia]bcj|BP

MSAIEHHASSKCPFHPAPAAATAPNGCPVSAAAAAFDPFDDGYQQDPPEYVRWAREREPV
FYSPKLGYWIVTRYDDIKAIFRDNLTFSPSIALEKITPTGAEANAVLASYGFALNRTLVN
EDEPAHMPRRRALLDPFTPEALKRHEPMVRRLAREYVDRFIDDGRADLVDQMLWEVPLTV
ALHFLGVPEEDMDTLREYSIAHTINTWGRPKPEEQVAVAHAVGKFWQFAGKVLDKMREDP

SGPGWMQYGIRKQKTLPDVITDSYLHSMMMAGIVAAHETTANATANAMKLLLQHPDAWRD
ICDDPSLIPNAVEECLRHNGSVAAWRRLATKDVTIGGVAVPAGSKLLIVTSSANHDEARF
ADADLFDIRRENASDQLTFGYGSHQCMGKNLARMEMQIFLEEFTRRLPHMKLAEQRFTYV
PNTSFRGPEHLYVEWDPAANPERRDPSALRARTAVRIGEPSSHAVSRVVKVQRVSRCADR
VAQVRLVSGDGRMLPRWTPGAHIDVICGDTGITRQYSLCGDPAERDAYEIAVLHEAQGRG
GSAWVHANLRAGDVLKIRGPRNHFRFATDAGKAIFVAGGIGITPISAMAREAKVRGIDYE
IHYSGARRASMALLDALTALHGERLHVYVSDEGSRNDFRALFAQPADDTRIYACGPARML
DALEASCAHWPRDALRVEHFVSTRAALDPSKERPFEVELKDSGITLTVAANQTVLDALQH
ANIDVQSDCREGLCGSCEVRVLAGRVDHRDSVLTRAERDAHDRMMTCCSRACGGERLVLE
L

>DM39_4072|KEGG|CYP1487A1|[Burkholderia cenocepacia]bcen|BP

MNSSVQTNGEAGRCPFHSAFDPLEPAQIADPFPILAKMREESPVFYMEKYDLWVVTRRED
VLAVYKDMVSFSNGNAHKPLSPKSQAVIDRVGEDWPLPVDGSLNAMDPPEHLPLKRLFMS
VFHKALPGMDEWLDEKLNNLVDRFVDAGEADLVPTFFWPTTVGAVAHLIGAADSETERFN
EWAENWFELTGSTLLSPERAEACWMAFIDFEQWAYALLEARRLNPQHDLISKLIEAQQSG
AAITDRQIVTNMVGMVAAGTDTTANTIAQMIYTLLKHPDQLQRVRENPELRPQMIEEIIR
LRLPIRGVVRTMTQDVEMSGVTLPKGAKVYIHLGSANHDSSMFANPDEFDIERTNAHKHV
SFGAFNRACVGAPLARLEIAKALDIILDRLPGLRLVDESEQLRYTESMIVPSLKSLRVRW
DVPRKTV

>DM39_5695|KEGG|CYP229F7|[Burkholderia cenocepacia]bcen|BP

MNPTDPIAAVTHRDPYPYYATLVDGPPLAFDASLGLWVASRAASVAAVLGHPVCRVRPLD
MPVPPALRGTTAGALFGELVRMNDGALRHDVPKQALRTALASVDARALRDRAARIAAGRL
PARLDADALNAWCMTTPVCAVAAQLGFDESQLDGIAASVVDFVAALSPLSDAAALARASD
AARQLLDRMTERVANAHAQDGTLVDAIRQAARETGWHASGALVANLVGLLSQTCEATAAW
LGNAVVAWHQTNGAAETARDDAALDAFVAEVGRFDSPVQNTRRFVAARTTIEGTSVEAGD
AILVVLAAANRDPAVHRDPQRLMPGRTAGPNFGFGTGPHACPGERIARAVTVGAFGALLR
AGGGPPRDALKWGYRASTNVRMPKFEARP

>DM40_4874|KEGG|CYP229F1|[Burkholderia cenocepacia]bcew|BP

MNPTDPIAAVTHRDPYPYYATLVDGPPLAFDAALGLWVASRAASVTAVLGHPACRVRPLD
APVPPALRGTTAGALFGDLVRMNDGALRHDVPKQALRAAFAPLDADALRERAARLAGDRL
RAPHDAHALNAWCMTVPVCAVADLLGFDDAQLDDIAAWVVDFVAALSPLSDAAALAQASE
GARHLLDRMTERVAHTRAQDGSLVAAVQQAARAAGWHASGALVANLVGLLSQTCEATAAW
LGNVLVAWDTTKPGGARAMPDAAALDAFVAEVGRFDAPVQNTRRFVASRTMIEGVTVDAG
AAILVVLAAANRDPSAHRDPHRLLPARAPGPNFGFGTGPHGCPGERIARAVTAGAFGAWL
RAAGHEPRDRLVWDYRASANVRMPKFMPAR

>I35_5939|KEGG|CYP102J5|[Burkholderia cenocepacia]bceo|BP

MFDKMVDIADQMFLYWERFGPETAVDVSDHMTRLTLDTIALCAFDCRFNSFYREDQHPFV
DAMVNTLSEAGKRELRPKLVSKLMLNRSRKFDADIELMRSLATKMIEDRRKNPHDNEEAM
DLLDRMLNGVDPVTGEKLDDENIVFQMITFLIAGHETTSGLLSFATYFLLKNPDVLQRAR
DMVDEVVGNDTPRIEHLARLRYIEQILMETLRIWPTAPAFAVTPLADTKFGGKYPVSPND
IIMILTPMLHRDVSVWGDDVEAFRPERFEPENAEKLPPNSWKPFGNGARACIGRPFAMQE
AHLVLIMLLQRFDFSFADPNYELQVAETLTLKPTGLRINVRPRARGVRKVSNDNLRVRAS
IETPNVDQAPAVSDKDASKMLVLFGGNTGSSESFARRIAGDAGRHGFHATCAPLDAFAGK
IDGFPAIVVVTASYEGQPPDNAISFLPYIEAMSEGALDGVKFSVLGCGNKQWARTYQAIP
KRVDAAFEKAGATRVHARGELDSGGDFFGEFDRWYAELWNRFAISFGKDTSVAQNNNAAL
NVSFLNNVREKLLSVDDMTHATVIDNRELVDISAPGSRSKKHMELKLPETMTYRSGDYLA
VLPRNSKRNVDRVLRRFRLSWDTQVVIDGTSSNPRLPLGQAISCGELLSSYVELAVPATR
SQVSILATATRCPPEKVDLERLSTGDFECEILEKRTTVMDLLERFGSVDLSFDKFLDMLP
ALKARQYSISSSPLWKADHVTLTVAVVDAPALSGNGRHEGVASSYLARLDAGDSVSVSVR
PSNARFRPPAESNLPMILICAGSGIAPFRGFLQERAIQKQRGENVGPSLLFFGIDDPDVD
FLYRDELEEWANLGVVEVMPAYSNRPEEGARFVQDKVWLEREKIAALFAQGATVYVCGDG
KNMAPAVRATLGRIYQDSTGANDESAHAWIDTMEREHGRYVADVFA

>I35_6489|KEGG|CYP116B109|[Burkholderia cenocepacia]bceo|BP

MSAIEHHASSKCPFHPAPAAATAPNGCPVSAAAAAFDPFDDGYQQDPPEYVRWAREREPV
FYSPKLGYWIVTRYDDIKAIFRDNLTFSPSIALEKITPTGAEANAVLASYGFALNRTLVN
EDEPAHMPRRRALLDPFTPEALKHHEPMVRRLAREYVDRFIDDGRADLVDQMLWEVPLTV

ALHFLGVPEEDMDTLREYSIAHTINTWGRPKPEEQVAVAHAVGKFWQFAGKVLDKMREDP
SGPGWMQYGIRKQKALPDVITDSYLHSMMMAGIVAAHETTANATANAMKLLLQHPDAWRD
ICDDPSLIPNAVEECLRHNGSVAAWRRLATKDVTIGGVAVPAGSKLLIVTSSANHDEARF
ADADLFDIRRENASDQLTFGYGSHQCMGKNLARMEMQIFLEEFTRRLPHMKLAEQRFTYV
PNTSFRGPEHLYVEWDPAANPERRDPSALRARTAVRIGEPSSHAVSRVVKVQRVSRCADR
VAQVRLVSGDGRMLPRWTPGAHIDVICGDTGITRQYSLCGDPDERDAYEIAVLHEAQGRG
GSAWVHANLRAGDVLKIRGPRNHFRFATDAGKAIFVAGGIGITPISAMAREAKARGIDYE
IHYSGARRASMALLDTLAALHGERLHVYVSEEGSRNDFRALFAQPADDTHIYACGPARML
DALEASCAHWPRDALRVEHFVSTRAALDPSNERPFEVELRDSGITLTVAANQTVLDALQH
ANIDVQSDCREGLCGSCEVRVLAGRVDHRDSVLTRAERDAHDRMMTCCSRACGGERLVLE
L

>Bamb_4351|KEGG|CYP229F1|[Burkholderia ambifaria]bam|BP

MNPTDPIAAVTHRDPYPYYATLVDGPPLAFDATLGLWVASRAASVTAVLGHPACRVRPLD
APVPPALRGTTAGALFGELVRMNDGALRHDVPKQALRAACAPIDAGALRERAAQLAGSRL
RAPHDADALNAWCLSVPVCAVADLLGFDEMQLDDIAALVVDFVAALSPLSDAAALARASD
AARQLLDRMTERVARTRAQDGSWVAAIQQAAAAAGWHASDALVANLVGLLSQTCEATAAW
LGNVLVAWDGTKDDAAEGERAMPDTAALEAFVAEVGRFDSPVQNTRRFVASRTTIEGVTV
DAGAAILAVLAAANRDPAVHREPHRLLPGRAPGPNFGFGTGPHGCPGEQIARAVTAGAFG
AWLRAGGEAPRDRLVWDYRASTNVRMPKFGPAR

>BamMC406_4867|KEGG|CYP229F1|[Burkholderia ambifaria]bac|BP

MNPTDPIAAVTHRDPYPYYATLVDGPPLAFDATLGLWVASRAASVTAVLGHPACRVRPLD
APVPPALRGTTAGALFGELVRMNDGALRHDVPKQALRAACAPIDAGALRERAAQLAGSRL
RAPDDADALNAWCLSVPVCAVADLLGFDEMQLDDIAALVVDFVAALSPLSDDAALARASD
AARQLLDRMTERVARTRAQDGSWVAAIQQAAAAAGWHASGALVANLVGLLSQTCEATAAW
LGNVLVAWDGTTDDAADGERAMPDTAALEAFVAEVGRFDAPVQNTRRFVASRTTIEGVTV
DAGAAILVVLAAANRDPAVHREPHRLLPGRASAPNFGFGTGPHGCPGERIARAVTAGAFG
AWLRAGGEATRGKLVWDYRGSTNVRMPKFGPAR

>BMULJ_05568|KEGG|CYP116B113|[Burkholderia multivorans]bmj|BP

MSVTQRDASSSQCPFHPAPAAAAAPNGCPVSAAAAAFDPFEDGYQQDPPEYVRWAREQEP
VFYSPKLGYWVVTRYDDIKAIFRDNLTFSPSIALEKITPTGPEANAVLASYGFALNRTLV
NEDEPAHMPRRRALLDPFTPDALKHHEPMVRRLAREYVDRFIDDGHADLVDQMLWEVPLT
VALHFLGVPEEDMDTLREYSIAHTINTWGRPKPEEQVAVAHAVGKFWQFAGKVLDKMRED
PSGPGWMQYGIRKQKELPDVITDSYLHSMMMAGIVAAHETTANATANAMKLLLQHPDAWR
DICEDPSLIPNAVEECLRHNGSLAAWRRLATKDVTIGGVDIPAGAKLLIVTSSANHDEAR
FADADLFDIRRENASDQLTFGYGSHQCMGKNLARMEMQIFLDEFTRRLPHMKLAEQRFTY
VPNTSFRGPEHLYVEWDPALNPERRDRSVLRPRAAVRIGEPSSHAVSRAVKVERVSICAD
RVAHVRLVSADGRPLPRWTAGSHIDVICGDTGITRQYSLCGDPDERDAYEIAVLHEAQSR
GGSAWVHANLRAGDVLKIRGPRNHFRFVTDAGKAIFVAGGIGITPISAMAREAKARGIDY
EIHYSGARRASMAMLDTLAALHGERLHVYVSEEGTRNDFHALFAQPADDAHIYACGPARM
LDALDASCAHWPRDALRVEHFVSTKAALDPSKEQQFEVELKDSGITLTVAANQTVLDALQ
HANIDVQSDCREGLCGSCEVRVLAGRVDHRDSVLTRAERDAHDRMMTCCSRACGGERLVL
EL

>BMULJ_05578|KEGG|CYP117C1|[Burkholderia multivorans]bmj|BP

MLLNPLSRRVRMQQGMPVLPGAFPVVGHLPAVARALPDLLAYGERRWGTHFWLDMGAMGR
MLVCTRPEAFELFNNKDVLADLLGDVAPAMRDTMATQDGELHRRTRAWANPSFQPQGLSA
AKVGELFADIMMAWIRAWPARGTINLVDETSALTLTMIFRMLGIAEDDIATWETHYRHYA
LVSLLPVDLPGTPLRRSRRAQVWIDARLSALVNELGRSPGDSGLLATLIRSPAAPDASSP
ESNLLGNLRALIHAAHKTTAVTMAHLAIRLAEQPDHWDALVAEARAAGTVPRNPSELARF
PFAEALFLETLRSEPAFPLVFRRTRERIELGGRELAAGTRLIIPLILLSRAPSLYPQPDT
FLPQRWLEHRGAHKPIQTLQFGSGPHRCLGFHTACMQLVQFAVTLALVLDERKMRPQLVP
QRTCGRRRFPINYPPRDTQVAFRSVSSNHDNRSEIPS

>BAG43225.1|NCBI|CYP1013A4|[Burkholderia multivorans]bmj|BP

MSRKAIAPGPRGSLVMGNLAAYKRNPVTMLLKLQQQYGDVVRNRLGPFVTHALAHPDGVQ
HVLQDNHRNYVRGRFYDNFRMFFGDGLLTTDGEFWRRHRRVVQPLFHRKQVDAHAAAVGD
AALALVEQWFARPDADAPFDVVEATMHVSLRMLGLMLFNADLSRHAHDVGPAVRFGIEAM
MPQGNLNDFVPRWMPTPFNRRIAHARRAIDTIVDAIVAEHRAGRCAPSDVISLLLAARDP
ETGAPLTEREVHDEVMTVLLAGHETTGSGMAWGLYALAQHPDVLRRLRDELDAVLGGRAP
APDDLERLPYLLQTVDEMLRVYPPIWGFTRDLVDDDEIGGYHVPAGSSVFLSPYVTHRHP
ALWSHPDAFDPERFASHAPARHKYAYFPFGGGMRKCIGYQTALLQMRVLIAVVAQHVDLS
TVPGQSLDTGATISLRPRDGIRMIAKPRVRMHAAQRARTPRADARADACPFGGTRAPDAA
PLTRDAQPQGGADDGRAVMQPHTTQTSHAASDASPVASPLASPAAGPVLPDDLLAHLRRV
TGARTEHRAAPPDDAPPTRRFTWRAIEADPLPEMPAAALSGKRVAIVNGRASTVERVVSA
LTRACAKAAVFAPPDGADAAAAAQTFAVQHGPFDAIVDLGIEAAFALDAHDAWDAPLRRT
IALLHACYEDWSREESTSRLFYLAVTWMDGRMGYGDAPPVQPLGGLWAGLAKTLPQELPN
CNVRVLDIAPDETGRAHALIARELYRGGRFEIGHQGGRRYTLTAAHDPLPADATPDWGPD
DIVLFSGGARGIGLLAACALAKRCGCTVVVTGREPLPAGDEPWLALDEAGFRRYGFEQLR
QATRSDTPKMIRQRLRQLERRRDMHASLAAAAALGVPLQYRVCDVTDAAAVRALCDSLGA
RLRAVVHNAGIDQPVRLPAKQADEFLGTVRVKVRGFMNLYAAVRDRAHVTGFFNVGSLTG
RWGGMTGEADYAAANEALARLGLWARHDAHGRVVKTLVWPTWDGVGMITHLAVTRRYVSP
MRIDDGVRYWLEELADGRSGEAMYMGAVGRAVTPVQLRGFGPVDGLPNLHELATRALHAG
EPRRFRPFARFSTHYRTVGAPYARAFRYDGRAAAPASLLLEHARVAADWVAPPDGEPVAL
HRIDDVTIRLDALSDTLDASGDVHVDTDVAGGAADDGWRVHARCTTVGGRTVLEATFVYR
RGTPARTTVVLPPDDCDAPAAPCAPVAWRGDLLPAARWHGLLGQVRAAEPDALWQTLDPA
AGAPGAALWLPVNHLENTLRALFGAWPAERGLPHTWRIGRIDVGAPCADDAELIVRYSGE
RFAIADRAGARIAELRDTALVGDAPAGASAAEPVAAFDTNGGDGR

>Bmul_1952|KEGG|CYP1013A4|[Burkholderia multivorans]bmu|BP

MSRKAIAPGPRGSLVMGNLAAYKRNPVTMLLKLQQQYGDVVRNRLGPFVTHALAHPDGVQ
HVLQDNHRNYVRGRFYDNFRMFFGDGLLTTDGEFWRRHRRVVQPLFHRKQVDAHAAAVGD
AALALVEQWFARPDADAPFDVVEATMHVSLRMLGLMLFNADLSRHAHDVGPAVRFGIEAM
MPQGNLNDFVPRWMPTPFNRRIAHARRAIDTIVDAIVAEHRAGRCAPSDVISLLLAARDP
ETGAPLTEREVHDEVMTVLLAGHETTGSGMAWGLYALAQHPDVLRRLRDELDAVLGGRAP
APDDLERLPYLLQTVDEMLRVYPPIWGFTRDLVDDDEIGGYHVPAGSSVFLSPYVTHRHP
ALWSHPDAFDPERFASHAPARHKYAYFPFGGGMRKCIGYQTALLQMRVLIAVVAQHVDLS
TVPGQSLDTGATISLRPRDGIRMIAKPRVRMHAAQRARTPRADARADACPFGGTRAPDAA
PLTRDAQPQGGADDGRAVMQPHTTQTSHAASDASPVASPLASPAAGPVLPDDLLAHLRRV
TGARTEHRAAPPDDAPPTRRFTWRAIEADPLPEMPAAALSGKRVAIVNGRASTVERVVSA
LTRACAKAAVFAPPDGADAAAAAQTFAVQHGPFDAIVDLGIEAAFALDAHDAWDAPLRRT
IALLHACYEDWSREESTSRLFYLAVTWMDGRMGYGDAPPVQPLGGLWAGLAKTLPQELPN
CNVRVLDIAPDETGRAHALIARELYRGGRFEIGHQGGRRYTLTAAHDPLPADATPDWGPD
DIVLFSGGARGIGLLAACALAKRCGCTVVVTGREPLPAGDEPWLALDEAGFRRYGFEQLR
QATRSDTPKMIRQRLRQLERRRDMHASLAAAAALGVPLQYRVCDVTDAAAVRALCDSLGA
RLRAVVHNAGIDQPVRLPAKQADEFLGTVRVKVRGFMNLYAAVRDRAHVTGFFNVGSLTG
RWGGMTGEADYAAANEALARLGLWARHDAHGRVVKTLVWPTWDGVGMITHLAVTRRYVSP
MRIDDGVRYWLEELADGRSGEAMYMGAVGRAVTPVQLRGFGPVDGLPNLHELATRALHAG
EPRRFRPFARFSTHYRTVGAPYARAFRYDGRAAAPASLLLEHARVAADWVAPPDGEPVAL
HRIDDVTIRLDALSDTLDASGDVHVDTDVAGGAADDGWRVHARCTTVGGRTVLEATFVYR
RGTPARTTVVLPPDDCDAPAAPCAPVAWRGDLLPAARWHGLLGQVRAAEPDALWQTLDPA

AGAPGAALWLPVNHLENTLRALFGAWPAERGLPHTWRIGRIDVGAPCADDAELIVRYSGE
RFAIADRAGARIAELRDTALVGDAPAGASAAEPVAAFDTNGGDGR

>Bmul_3696|KEGG|CYP229F3|[Burkholderia multivorans]bmu|BP

MNPTDPIAAVTHRDPYPYYAALLDGPPLAYDASLGLWVATRAASVAAVLGHPACRVRPLD
LPVPPALRGTSAGALFGELVRMNDGALRHQLPKQALHAALAQIDVQALRGRAATLAAAQR
PALDDPDALNAWCIATPVRAVADLLGFDEPQLDDIAASVVDFVAALSPLSDAASLARASD
AARQLLERMTVRVAQSHARDGTLVAAVKDAAAQAGWHASGALVANLVGLLSQTCEATAAW
LANTIVAWHRQSAESASSADVPDDATLDAFVAEVARFDTPVQNTRRFVAARTTLEGVTLD
AGAAILVVLAAANRDPAVHRDPHRFEPARTPGPDFGFGTGPHQCPGARIARAVTVGAFGA
LLRAGGWPPREALKWDYRASANIRMPKFDAAR

>Bmul_5948|KEGG|CYP117C1|[Burkholderia multivorans]bmu|BP

MLLNPLSRRVRMQQGMPVLPGAFPVVGHLPAVARALPDLLAYGERRWGTHFWLDMGAMGR
MLVCTRPEAFELFNNKDVLADLLGDVAPAMRDTMATQDGELHRRTRAWANPSFQPQGLSA
AKVGELFADIMMAWIRAWPARGTINLVDETSALTLTMIFRMLGIAEDDIATWETHYRHYA
LVSLLPVDLPGTPLRRSRRAQVWIDARLSALVNELGRSPGDSGLLATLIRSPAAPDASSP
ESNLLGNLRALIHAAHKTTAVTMAHLAIRLAEQPDHWDALVAEARAAGTVPRNPSELARF
PFAEALFLETLRSEPAFPLVFRRTRERIELGGRELAAGTRLIIPLILLSRAPSLYPQPDT
FLPQRWLEHRGAHKPIQTLQFGSGPHRCLGFHTACMQLVQFAVTLALVLDERKMRPQLVP
QRTCGRRRFPINYPPRDTQVAFRSVSSNHDNRSEIPS

>Bmul_5958|KEGG|CYP116B113|[Burkholderia multivorans]bmu|BP

MARERIMSVTQRDASSSQCPFHPAPAAAAAPNGCPVSAAAAAFDPFEDGYQQDPPEYVRWAREQEPVFYSPKLGYWVVTRYDDIKAIFRDNLTFSP
SIALEKITPTGPEANAVLASYGFALNRTLVNEDEPAHMPRRRALLDPFTPDALKHHEPMVRRLAREYVDRFIDDGHADLVDQMLWEVPLTVALHFL
GVPEEDMDTLREYSIAHTINTWGRPKPEEQVAVAHAVGKFWQFAGKVLDKMREDPSGPGWMQYGIRKQKELPDVITDSYLHSMMMAGIVAAHETTA
NATANAMKLLLQHPDAWRDICEDPSLIPNAVEECLRHNGSLAAWRRLATKDVTIGGVDIPAGAKLLIVTSSANHDEARFADADLFDIRRENASDQL
TFGYGSHQCMGKNLARMEMQIFLDEFTRRLPHMKLAEQRFTYVPNTSFRGPEHLYVEWDPALNPERRDRSVLRPRAAVRIGEPSSHAVSRAVKVER

VSICADRVAHVRLVSADGRPLPRWTAGSHIDVICGDTGITRQYSLCGDPDERDAYEIAVLHEAQSRGGSAWVHANLRAGDVLKIRGPRNHFRFVTD
AGKAIFVAGGIGITPISAMAREAKARGIDYEIHYSGARRASMAMLDTLAALHGERLHVYVSEEGTRNDFHALFAQPADDAHIYACGPARMLDALDA
SCAHWPRDALRVEHFVSTKAALDPSKEQQFEVELKDSGITLTVAANQTVLDALQHANIDVQSDCREGLCGSCEVRVLAGRVDHRDSVLTRAERDAH
DRMMTCCSRACGGERLVLEL

>DM80_293|KEGG|CYP1013A4|[Burkholderia multivorans]bmk|BP

MSRKAIAPGPRGSLVMGNLAAYKRNPVTMLLKLQQQYGDVVRNRLGPFVTHALAHPDGVQ
HVLQDNHRNYVRGRFYDNFRMFFGDGLLTTDGEFWRRHRRVVQPLFHRKQVDAHAAAVGD
AALALVEQWFARPDADAPFDVVEAMMHVSLRMLGLMLFNADLSRHADDVGPAVRFGIAAM
MPQGNLNDFVPRWMPTPFNRRIAHARRAIDTIVDAIVAEHRAGRCAPSDVISLLLAARDS
ETGAPLTEREVHDEVMTVLLAGHETTGSGMAWGLYALAQHPDVLRRLRDELDAVLGGRAP
APDDLERLPYLLQTVDEILRVYPPIWGFTRDLVDDDEIGGYHVPAGSSVFLSPYVTHRHP
ALWSHPDAFDPERFASHAPARHKYAYFPFGGGMRKCIGYQTALLQMRVLIAVVAQHVDLS
AVPGQSLDTGATISLRPRDGIRMIAKPRARTHAAQSARTPRAGARADACPFGGARAPDAA
PLARDMQPQGGADDGRAVMQPHAKQAPHAASDASPVASPPASPAAGPVLPDELLAHLRRV
TGASTEHRAAPPDDAPPTRRFTWRAIEADAVPEMPAGELSGKRVAIVNGRASTVERVVSA
LTRACAKPAVFAPPDGADAAAAAQTFAVQHGPFDAIVDLGIEAAFALDAHDAWDAPLRRT
IALLHACYDDWSREESTSRLFYLAVTWMDGRMGYGDAPPVQPLGGLWAGLAKTLPQELPN
CNVRVLDIAPDETGRVHALIARELYRGGRFEIGHQGGRRYTLTAAHDPLPADAAPDWGPD
DIVLFSGGARGIGLLAACALAKRCGCTVVVTGRDPLPAGDEPWLALDEAGFRRYGFEQLR
HATRSDTPKMIRQRLRQLERRRDMHASLAAAAALGVPLQYRVCDVTDATAVRALCDSLGA
RLRAVVHNAGIDQPVRLPAKQADEFLGTVRVKVRGFMNLYAAVRDRAHVTGFFNVGSLTG
RWGGMTGEADYAAANEALARLGLWARHDAHGRVVKTLVWPTWDGVGMITHLAVTRRYVSP
MRIDDGVRYWLEELADGRSGETMYMGAVGRAVTPVQLRGFGPVDGLPNLHELATRALHAG
EPRRFRPFARFSTHYRTVGAPYARAFRYDGRAAAPASLLLEHARVAADWVAPPDGEPVAL
HRIDDVTIRLDALADTLDASGDVHVDTDVAGGAADDGWRVHARCTTVGGRTVLAATFVYR
RGTPARTTVVLPPDDCDAPAAPCAPVAWRGDLLPAARWHGLLGQVRAAEPDALWQTLDPA
AGTHGAALWLPVNHLENILRALFGAWPAARGLPHTWRIGRIDVGAPCADDAELIVRYSGE
RFAIADRAGARVAELRDTALVGDAPAGAPAAEPVAAFDTNGGDGR

>DM80_1416|KEGG|CYP195C1|[Burkholderia multivorans]bmk|BP

MSTTEMMARPTAPGSRFDLFNLPDAYYRDPAPFYKALRDNEPLLWNSDGSVLLTRYDDVK
AVWRDLSGRVNRAEAYAKKFGEGPLLEHHTSSMLFRDPPDHDRLRDIVNPFFTPSSIERL
RTAVREIVERLIEEVREKREIDFVAEFSSRIPIQVICDIFGVPRSDGPLIRDLGAQVLFP
LNPAVSAEAIAAGHEATRKFKDYLSEHFNYWRRQPVEDMPTNIISALVAAEKRGNPPISE
VEMAHMCILVLNGGHETTTNLIGLSTLALMKHPDQMALLAKEPSVIGPGVEECLRYTTPL
QLQGRRTTQEVQVPSGTIPADTEVIFAQVSANRDERVFADPDRLQLTRQPNAHLAFGAGI
HVCLGRPLARMEAAEALPALARHFPSMQLIDEPVFARSARFRGIQSMRVRLY

>DM80_5078|KEGG|CYP229F3|[Burkholderia multivorans]bmk|BP

MNPTDPIAAVTHRDPYPYYAALLDGPPLAYDASLGLWVATRATSVAAVLGHPACRVRPLD
LPVPPALRGTSAGALFGELVRMNDGALRHQVPKQALHAALAQIDVQALRGRAATLAAAQL
PALDDPDALNAWCIATSVRAVADLLGFDEPQLDDIAASVVDFVAALSPLSDAASLARASA
AARQLLDRMTVRVAQSHARDGTLVAVVKDAAAQAGWHASGALVANLVGLLSQTCEATAAW
LANTIVAWHRQSASSADVPDDATLDAFVAEVARFDTPVQNTRRFVAARTTLEGVTLDAGA
AILLVLAAANRDPAVHRDPHRFVPARTPGPDFGFGTGPHQCPGARIARAVTVGAFGALLR
AGGWPPREALKWDYRASANIRMPKFDAAR

>DM80_6397|KEGG|CYP117C1|[Burkholderia multivorans]bmk|BP

MLLNPLSRRVRMQQGMPVLPGAFPVVGHLPAVARALPDLLAYGERRWGTHFWLDMGAMGR
MLVCTRPEAFELFNNKDVLADLLGDVAPAMRDTMATQDGELHRRTRAWANPSFQPQGLSA
AKVGELFADIMMAWIRAWPARGTINLVDETSALTLTMIFRMLGIAEDDIATWETHYRHYA
LVSLLPVDLPGTPLRRSRRAQVWIDARLSALVNELGRSPGDSGLLATLIRSPAAPDASSP
ESNLLGNLRALIHAAHKTTAVTMAHLAIRLAEQPDHWDALVAEARAAGTVPRNPSELARF
PFAEALFLETLRSEPAFPLVFRRTRERIELGGRELAAGTRLIIPLILLSRAPSLYPQPDT
FLPQRWLEHRGAHKPIQTLQFGSGPHRCLGFHTACMQLVQFAVTLALVLDERKMRPQLVP
QRTCGRRRFPINYPPRDTQVAFRSVSSNHDNRSEIPS

>AIO71674.1|NCBI|CYP116B113|[Burkholderia multivorans]bmk|BP

MSVTQRDASSSQCPFHPAPAAAAAPNGCPVSAAAAAFDPFEDGYQQDPPEYVRWAREQEP
VFYSPKLGYWVVTRYDDIKAIFRDNLTFSPSIALEKITPTGPEANAVLASYGFALNRTLV
NEDEPAHMPRRRALLDPFTPDALKHHEPMVRRLAREYVDRFIDDGHADLVDQMLWEVPLT
VALHFLGVPEEDMDTLREYSIAHTINTWGRPKPEEQVAVAHAVGKFWQFAGKVLDKMRED
PSGPGWMQYGIRKQKELPDVITDSYLHSMMMAGIVAAHETTANATANAMKLLLQHPDAWR
DICEDPSLIPNAVEECLRHNGSLAAWRRLATKDVTIGGVDIPAGAKLLIVTSSANHDEAR
FADADLFDIRRENASDQLTFGYGSHQCMGKNLARMEMQIFLDEFTRRLPHMKLAEQRFTY
VPNTSFRGPEHLYVEWDPALNPERRDRSVLRPRAAVRIGEPSSHAVSRAVKVERVSICAD
RVAHVRLVSADGRPLPRWTAGSHIDVICGDTGITRQYSLCGDPDERDAYEIAVLHEAQSR
GGSAWVHANLRAGDVLKIRGPRNHFRFVTDAGKAIFVAGGIGITPISAMAREAKARGIDY
EIHYSGARRASMAMLDTLAALHGERLHVYVSEEGTRNDFHALFAQPADDAHIYACGPARM
LDALDASCAHWPRDALRVEHFVSTKAALDPSKEQQFEVELKDSGITLTVAANQTVLDALQ
HANIDVQSDCREGLCGSCEVRVLAGRVDHRDSVLTRAERDAHDRMMTCCSRACGGERLVL
EL

>NP80_4852|KEGG|CYP229F3|[Burkholderia multivorans]bmul|BP

MNPTDPIAAVMHRDPYPYYAALLDGPPLAYDASLGLWVATRAASVRAVLGHPACRVRPLD
LPVPPALRGTSAGALFGELVRMNDGALRHQVPKQALHAALAQIDVQASRGRTATLAAAQL
PALDDPGALNAWCIATPVRAVADLLGFDESQLDDIAASVVDFVAALSPLSDAASLARASD
AARQLLDRMTVRVAQSHARDGTLVSAVKDAAAEAGWHASGALVANLVGLLSQTCEATAAW
LANAIVAWHRQSAESVSPADVPDDATLDAFVAEVARFETPVQNTRRFVAARTTLEGVTLD

AGAAILVVLAAANRDPAVHRDPHRFVPARTPGPDFGFGTGPHQCPGARIARAVTVGAFGA
LLRAGGWPPREALKWSYRASANIRMPKFDAAR

>NP80_5652|KEGG|CYP116B113|[Burkholderia multivorans]bmul|BP

MSVTERDASSSKCPFHPAPAAATAPNGCPVSAAAAAFDPFEDGYQQDPPEYVRWAREQEPVFYSPKLGYWVVTRYDDIKAIFRDNLTFSPSIALEK
ITPTGPEANAVLASYGFALNRTLVNEDEPAHMPRRRALLDPFTPDALKHHEPMVRRLAREYVDRFIDDGHADLVDQMLWEVPLTVALHFLGVPEED
MDTLREYSIAHTINTWGRPKPEEQVAVAHAVGKFWQFAGKVLDKMREDPSGPGWMQYGIRKQKELPDVITDSYLHSMMMAGIVAAHETTANATANA
MKLLLQHPDAWRDICEDPSLIPNAVEECLRHNGSVAAWRRLATKNVTIGGVDIPAGAKLLIVTSSANHDEARFADADLFDIRRENASDQLTFGYGS
HQCMGKNLARMEMQIFLDEFTRRLPHMKLAEQRFTYVPNTSFRGPEHLYVEWDPALNPERRDRSVLRPRAAVRIGEPSSHAVSRAVKVERVSICAD
RVAHVRLVSADGRPLPRWTAGSHIDVICGDTGITRQYSLCGDPDERDAYEIAVLHEAQSRGGSAWVHANLRAGDVLKIRGPRNHFRFATDAGKAIF
VAGGIGITPISAMAREAQARGIDYEIHYSGARRASMAMLDTLAALHGERLHVYVSEEGTRNDFHALFAQPADDTHVYACGPARMLDALEASCAHWP
RDALRVEHFVSTKAVLDPSKEQPFEVELKDSGITLTVAANQTVLDALQHANIDVQSDCREGLCGSCEVRVLAGRVDHRDSVLTRGERDAHDRMMTC
CSRACGGERLVLEL

>GEM_3522|KEGG|CYP229F1|[Burkholderia cepacia]bct|BP

MNPTDPIAAVTHRDPYPYYATLVDGPPLAFDATLGLWVTSRAASVTAVLGHPACRVRPLD
APVPPALRGTTAGALFGELVRMNDGALRHDVPKQALRAAFAPIDADALRERAAQLAGRRL
RAPYDAAALNAWCLTVPVCAVADLLGFDDAQLDPIAGLVVDFVAALSPLSDAAALARASD
GARQLLDRMTERVAHTRAQDGSLVAAIQQAARAAGWQASGALVANLVGLLSQTCEATAAW
LGNALVAWDGTTADGAGAERAMPDAAALDAFVAEVGRFDAPVQNTRRFVASRTTIEGVTV
EAGAAILVVLAAANRDPAVHREPQRLLPGRAPGPNFGFGTGPHGCPGERIARAVTAGAFG
AWLRAGGGPSRDRLVWDYRASTNVRMPKFVAGR

>GEM_4303|KEGG|CYP212A9|[Burkholderia cepacia]bct|BP

MAAPVLHPPGVWPPGPRAGLTGWQLLRAMSRDLPGTLAGWQRAHGDVVHLRMWPEHAVVV
TDPALVRELLVTHHDAFVRWERGIRVFSQVHGRSVLIAEGDAWRDKRHALQPNFMPKPVQ
AFVPAIAATAGHALAQWPARDAHWPVESALTSLAMDVIMRTMFSDAIGADARIAEEAVRA
VSAAANAEFYQPASAPDWMPWKRGKARALAVLNGLIDRQLHARLDLPAHGWPDDLLSRLL
RLHRADAYAWPLRAVHDECMTAFLAGHETAAATLTWWAWNMAANPAAQAAARDEVVRLLA
GRAPTADTRPALRYLTQTLEETMRLYPAAPILISRRALRPVVLGPWQFPARTLFMLPVQL

MHHDARWFPEPDAFRPERFAADAPTLPRGAYMPFGTGPRVCLGQHLAMTEMTVVAAMILQ
RLVLAVPPGTPPPRPVLNVTLRPEQPLHLAIAPTGLTAVTVSA

>DM42_5842|KEGG|CYP229F2|[Burkholderia cepacia]bced|BP

MNPTDPIAAVTHRDPYPYYAALVDGPPLAFDTTLGLWVASRATAVTAVLGDPACRVRPLD
APVPPALRGTTAGALFGELVRMNEGASRHDVPKQALRAAFAPLDPAALRDRAEAIAARRL
SAPGDADALNAWCMTVPVCAVADLLGFDETQLDDIAARVVEFVAALSPLSDAAALARASD
AARQLLDRMTERVAQSHAQDGTLVAAVQQAARASGWQAGGALVANLVGLLSQTCEATAAW
LGNALVAWNGEAREVPDAAAIDAFVAEVGRFDSPVQNTRRFVAARTTIEGVSVEAGDAIL
VVLAAANRDPAVHREPHRLMPGRTAGPNFGFGTGPHGCPGERIALAVTAGAFAARLHAGG
WPPRDALKWDYRASTNVRMPKFDASR
>DM42_5902|KEGG|CYP102J5|[Burkholderia cepacia]bced|BP
MKSSALVPQPPLKPIIGHLMEVLGPSPLAKMMDLARTYGPVYWFEVFGQGYYVVSGQALV
DEVCDESRFQKSVHQSLLELRPAIGDGLFTAFSDEPNWAKAHRVLMQSFGPLSIWSMFDK
MVDIADQMFLYWERFGPETAVDVSDHMTRLTLDTIALCAFDCRFNSFYREDQHPFVDAMV
NTLSEAGKRELRPKLVSKLMLNRSRKFDADIELMRSLATKMIEDRRKNPHDNEEAMDLLD
RMLNGVDPVTGEKLDDENIVFQMITFLIAGHETTSGLLSFATYFLLKNPDVLQRARDMVD
EVVGNDTPRIEHLARLRYIEQILMETLRIWPTAPAFAVTPLADTKFGGKYPVSPNDIIMI
LTPMLHRDVSVWGDDVEAFRPERFEPENAEKLPPNSWKPFGNGARACIGRPFAMQEAHLV
LIMLLQRFDFSFADPNYELQVAETLTLKPTGLRINVRPRARGVRKVSNDNLRVRASIETP
NVDQAPAVSDKDASKMLVLFGGNTGSSESFARRIAGDAGRHGFHATCAPLDAFAGKIDGF
PAIVVVTASYEGQPPDNAISFLPYIEAMSEGALDGVKFSVLGCGNKQWARTYQAIPKRVD
AAFEKAGATRVHARGELDSGGDFFGEFDRWYAELWNRFAISFGKDTSVAQNNNAALNVSF
LNNVREKLLSVDDMTHATVIDNRELVDISAPGSRSKKHMELKLPETMTYRSGDYLAVLPR
NSKRNVDRVLRRFRLSWDTQVVIDGTSSNPRLPLGQAISCGELLSSYVELAVPATRSQVS
ILATATRCPPEKVDLERLSTGDFECEILEKRTTVMDLLERFGSVDLSFDKFLDMLPALKA
RQYSISSSPLWKADHVTLTVAVVDAPALSGNGRHEGVASSYLARLDAGDSVSVSVRPSNA
RFRPPAESNLPMILICAGSGIAPFRGFLQERAIQKQRGENVGPSLLFFGIDDPDVDFLYR
DELEEWANLGVVEVMPAYSNRPEEGARFVQDKVWLEREKIAALFAQGATVYVCGDGKNMA
PAVRATLGRIYQDSTGANDESAHAWIDTMEREHGRYVADVFA

>AK34_3593|KEGG|CYP229F8|[Burkholderia dolosa]bdl|BP

MNLTPADPIAAVTHRDPYPYYATLVDGPPLAFDRTLGLWVASRAATVTAVLGDPACRVRP
LDAPVPPALRGTTAGALFGELVRMNDGALRHDVPKHALRAALASFDANAFRDRATQVAAA
RLRAPCDSDALNAWCLTVPVCAVADLLGFDDAQLDDIAALVVDFVAALSPLSDESALARA
NDAARQLLDRMTERVARTKTRDGTLVAAVQQAARESGWHASGALIANLVGLLSQTCEATA
AWLGNTLIAWAGTAHDGVAIERDAPNDAALDAFIAEVARFDSPVQNTRRFVASRTTIDGV
TLEAGAAILVVLAAANRDPAVHREPDRLLPGRPSGPTFGFGTGPHGCPGERIARAVTAGA
FRAWLRAGGGVPRDALTWSYRASTNVRMPKFDAVR

>ABD05_24685|KEGG|CYP229F2|[Burkholderia pyrrocinia]bpyr|BP

MNSNPNDPIAAVTHRDPYPYYAALVDGPPLAFDATLGLWVASRAAAVTAVLGHPACRVRP
LDAPVPPALRGTTAGALFGELVRMNDGASRHDVPKHALRTALAPVDTGKLRDRAAQLAAR
RLPAPGDADALNAWCMTVPVCAVADLLGFDETQLDDIAARVVDFVAALSPLSDAAALARA
SDGARQLLDRMTERVAQTHAHDGTLVAAVQQAARASGWHASGALVANLVGLLSQTCEATA
AWLGNSLVAWNGEAEAARVAPDDAALDAFVAEVGRFDAPVQNTRRFVASRTTIEGVTVEA
GDAILVVLAAANRDPAVHRDPHRLMPGRAAGTNFGFGTGPHGCPGERIARAVTAGAFAAW
LRAGGWPPRGGLTWNYRASTNVRMPTFDAAR

>ABD05_28890|KEGG|CYP212A7|[Burkholderia pyrrocinia]bpyr|BP

MNTRSSSQCPFHAGAAAPAPVLHPPGVWPPGPRAGLTGWGLLRKMSRDLIGTLSGWQREY
GDLVHLRVWPEHAVVVTDPALVRELLVTHHDAFGRWERAIRIFAQVHGHSVLVAEGDAWR
DKRHALQPNFMPKPVQAFVPAIAATAGHALAQWPARDPDWPIESALTSLAMDVIMRTMFS
DTIGDDARAAEDAVRAVSAAADAAFYQPASTPDWMPWKRGKVRATAVLNGLIDRQLRARV
DMPERTWPDDLLSRLLRLHRADGRKWPLQAVHDECMTAFLAGHETTAGALTWWAWCMATN
PAVQAAARDEVSRVLGGRAPSAETRSSLRYLTQTLDETLRLYPSAPILISRRASRPVALG
AWQFPARTLFMLPLQLMQRDPRWFPEPDAFRPERFADDAPALPRGAYMPFGTGPRVCLGQ
HLAMTEMTVVAAMILQRHVLSVPPGMAAPRAVLNVTLRPERALHLAIEPTGLAADTVSA

>NL30_01690|KEGG|CYP229F2|[Burkholderia contaminans]bcon|BP

MNPTDPIAAVTHRDPYPYYAALVDGPPLAFDATLGLWVASRAAAVTAVLGHSACRVRPLD

APVPPALRGTTAGALFGELVRMNDGVLRHDVPKQALRTAFAPIDPAALRDRAAQVAARLL
PAPGDAGALNAWCMTVPVCAVADLLGFDETQLDGIAAQVVDFVAALSPLSDAAALARASD
AARALLDRMTERVARTHAHDGTLVAAVQQAARASGWQASGALVANLVGLLSQTCEATAAW
LGNALVAWHGEAGANAARVTPDDAALDAFVGEVGRFDSPVQNTRRFVASRTTIEGVTVEA
GDAILVVLAAANRDPAVHRDPQRFMPGRAAGPNFGFGTGPHGCPGERIARAVTAGAFGAW
LRAGGVPPHDGLAWDYRASTNVRMPKFDAAR

>NL30_05370|KEGG|CYP212A7|[Burkholderia contaminans]bcon|BP

MNTRSTSSCPFHAGAAAVAPVLHPPGVWPPGPRAGLTGWGLLRAMSRDLLGTLAGWQRAY
GDVVHVRVWPEHAVVVTDPALVRELLVTHHDALGRWERAVRVFAQVHGHSVLIAEGDAWR
DKRQALQPNFMPKAVQTFVPSIAATAGHALAQWPAHDPDWPIESALTSLAMDVIMRTMFS
NAIGDDARAAEEAVRAVSAAANADFYWPASTPDWVPWKRGKARAMAVLNGLIDRQLHARL
DLPARAWPDDLLSRLLRLHRDDARRWPLRAVHDECMTAFLAGHETTAGALTWWAWCMATN
PAAQAAARDEVSRVLGGCAPSAETRSSLRYLTHTLEETLRLYPSAPILISRRASRPIVLG
PWQFPARTLFMLPLQLMQRDARWFDAPDVFRPERFADDVPAAPRGTYMPFGTGPRVCLGQ
HLAMTEMTVVAAMILQRYALSAPPGGVPPRPVLNVTLRPDRPLHLAIAPAGERSA

>NL30_16140|KEGG|CYP229F2|[Burkholderia contaminans]bcon|BP

MNPTDPIAAVTHRDPYPYYAALVDGPPLAFDATLGLWVASRAAAVTAVLGHPACRVRPLD
APVPPALRGTTAGALFGELVRMNDGALRHDVPKQALRTAFAPIDTVALRDRVTQLAARRL
PTPGDAGALNAWCMTVPVCAVADLLGFDDTQLDDIAGLVVDFVAALSPLSDAVALARASD
AARQLLDRMTARVAQSHAHDGTLVAAVQQAARVSGWQASGGLVANLVGLLSQTCEATAAW
LGNTLVAWSGDASASAARVVPDDAALDAFVAEVGRFDSPVQNTRRFVASRTTIEGVTVEA
GDAILVVLAAANRDPAVHRDPHRFMPGRAAGPNFGFGTGPHGCPGERIARAVTAGAFGAW
LRAGGVAPHDGLAWDYRASTNVRMPKFDAAR

>NL30_31780|KEGG|CYP102J3|[Burkholderia contaminans]bcon|BP

MKSSSLVPQPPLKPVIGHLMEVLGPSPLAKMMDLARKYGPVYWFEVFGQGYYVVSGQTLV
NEVCDESRFQKCVHQSLLELRPAIGDGLFTAFGDEPNWAKAHRVLMQAFGPLSIWSMFDK
MVDIADQMFLHWERFGPETPVDVSDHMTRLTLDTIALCAFDCRFNSFYREDQHPFVDAMV

NTLSEAGKRELRPKLVSKLMVNRSRKFDADIEVMRSLATKMIEDRRKHPHDSEASMDLLD
RMLNGVDPVTGEKLDDENIVFQMITFLIAGHETTSGLLSFATYFLLKNPDILQKARDMVD
EVVGSETPRIEHLARLRYIEHILMETLRIWPTAPAFAVKPLADTTFGGKYAVSPDDIIMI
LTPMLHRDVSVWGEDVDAFRPERFAPENAEQLPPNAWKPFGNGARACIGRPFAMQEAHLV
LIMLLQRFDFSFADPDYELEVAETLTLKPTGLRINVKPRARSALKAPDTAVRARSSAQSP
SVAPVQSLPSSEDVSTMLVLFGGNTGSAESFARRIAGDASRHGFHATCAPLDDFAGKLDG
YPAIVIVTASYEGQPPDNARSFVPYVEALEEGALAGVHFSVLGCGNKQWARTYQAIPKRV
DEALEKAGATRVHVRGELDSGGDFFGEFDNWYTEMWDRFAISAGKEVSTVQHRDTALKVS
FAENTREKLLNFGDMANASIVDNRELVNISVAGSRSKKHIELKLPEPMTYQSGDYLAVLP
RNSKNNVDRVLRRFRVSWDTQVVIEGTSSNPRLPLGQPISCGDLFSSYVELAVPATRSQV
STLAAATRCPPEKVELERLSADDFECEILGKRTTVMDLLERFGSVDLSLEKFLDMLPALK
ARQYSISSSPLWKADHVTLTVAVVDAPALSGNGRHEGVASSYLARLNTGDSLSVAVRPSN
AHFRPPAETGLPMILICAGSGIAPFRGFLQERALQKQQGENVGKSLLFFGIDDPDVDFLY
REELDEWARCGVVEVMPAYSNRPEAGARFVQDKVWLEREKIAALFAQGATVFVCGDGKNM
APAVRATLGRIYQEATGANDESASAWIDTMEREHGRYVADVFA

>BW23_3354|KEGG|CYP229F4|[Burkholderia ubonensis]bub|BP

MNPTDPIAAVTHRDPYPYYAALVDGPPLAFDATLGLWVASRAATVSAVLAHPACRVRPLA
APVPPALRGTTAGELFGQLIRMNDGVLRHDAPKRAMQAALASVDALALRRRTATLAAQRL
PAHAAPDALNAWCMTVPVCAVAELLGFDDARLDDVAAWVADFVAALSPLSTAAQLAGASA
AAGRLLDRMTEIVAATRADDGSLVAAVQCAAGEAGWHASGALIANLVGLLSQTCEATAAW
LGNTVVAWLDPAKRPAGEPCAMDDATLDAFVAEVARFDAPVQNTRRFVAARTTLDGVTVD
AGDAILVVLAAANRDPAAQRDPHRFVPGRPPGPAFGFGAGPHGCPGERIARAVTVGAVGA
LLRGGGLPPGDAPGWRYRDSTNVRMPKFEPAR

>BW23_4825|KEGG|CYP169A2|[Burkholderia ubonensis]bub|BP

MSTDTSTITTCPVRKRLALRRKYWKLPDAGVNHWLDIQRDPLKWLADMHRTSPDLASLRM
GVKQLWCLFHPDAIRELMVSHRADLRRWEPSLCIMRQWNGSSFMMKEGEAARRQRQTVRP
HIQSPAVPEIQRIARHWSARIVPGQVVDLDLEMAAYSVTLAGHALFDIDLGPNAVTIAKA
VRILSRVALLESSTGLPLGHWFPSKMCPRKRWALRVMRAQIEQVAQESQRPLAMHRDDLA

TLMMASHQATGATLAWAQLLLARHPEVLAALRTELAAIDWSGVNQLQDLHRAPLLRAVIQ
ETLRLYPPAYALVPRQLSRAMTVSGTPLRRGDIVMISSWITHRDARWFPEPEAFRPERFL
EAPTWPQGAYFPFGVGDRACPGTGMAMMDLAVSLAYWIEHWDMTIVGPVTPQGWFSLRPK
NARVRLEPRMRADREPNETPRR

>BW23_5133|KEGG|CYP212A10|[Burkholderia ubonensis]bub|BP

MSRDLIGTLAGWRQTYGDVVHLRMWPEHAVVVTDPQLVRDLLVTHHDALVRWERGIRVFG
EVHGHSVLIAEGGAWRDKRHALQPGFMPKAVQGFVPTIAATAGHALAQWPAHDPRWPIES
ALTSLAMDVIVRTMFSDVIGDDARDAEAAVRTVSAAANAEFYQPWSAPDWMPWKRRKARA
SAVLNGLIDRQLHARLDVPDSAWPDDLLSRLLRLHRANARTWPLRAVHDECMTAFLAGHE
TTAATLTWWAWCMASNPAAQAAAREEVERVLGGRAPTARTRPLLRYLTQTLEETLRLYPA
APILISRRALRPIALGGWQLPARTLFMLPLQLMHDDPRWFPEPHAFRPERFDEAGPAVPR
GAYMPFGTGPRVCLGQHLATTEMTVIAAMLLQRFELKVPAGMAAPRSVLNVTLRPDRPLH
LGIAAISPDPVKVPADRSVTASPASPTPE

>WI26_20245|KEGG|CYP212A9|[Burkholderia diffusa]bdf|BP

MNTRSSSRCPFHADAAGAAPALHPPGVWPPGPRAGLTGWRLLRAMSRDLTGTLAGWQRAH
GDVVHLRMWPEHAVVVTDPALVRELLVTHHDALVRWERGIRVFSQVHGRSVLVAEGDAWR
DKRHALQPNFMPKPVQAFVPSIAATAGHALAQWPAHDAHWPIESALTSLAMDVIMRTMFS
DAIGANARVAEEAVRTISAAANAEFYQPVSAPDWMPWKRGKARALAVLNGLIDRQLHARL
DTPEHRWPDDLLSRLLRLHRADARTWPLQAVHDECMTAFLAGHETVAATLTWWAWNMAAN
PAAQAAARDEVVRVLGGRAPTADTRAALRYLTQTLEETMRVYPAAPILISRRALRPVALG
AWQFPARTLFMLPIQLMHRDARWFPAPTAFRPERFADDAPAIPRGAYMPFGTGPRVCLGQ
HLAMTEMAVVAAMILQRHELSVPPGMAPPRATLNVTLRPERPLHLAIAPTRPAAEAGPS

>WS51_04910|KEGG|CYP212A9|[Burkholderia territorii]btei|BP

MNTRSSSRCPFHADAAGAAPALHPPGAWPPGPRAGLTGWRLLRAMSRDLLGTLAGWQRAH
GDVVHLRMWPEHAVVVTDPALVRELLVAQHDALVRWERGIRVFSQVHGHSVLIAEGHAWR

DKRHAMQPNFMPKPVQAFVPSIAATAGHALAQWPAHDAHWPIESALTSLAMDVIMRTMFS
DAIGANARVAEAAVRTISAVANAEFYQPANAPDWMPWKRGKARALAVLNGLIDRQLHARL
DVPEHGWPDDLLSRLLRLHRADPRTWPLQAVHDECMTAFLAGHETVAATLTWWAWNMAAH
PAAQAAARDEVVRVLGGRAPTADTRAALRYLTQTLEETMRMYPAAPILISRRASRPVALG
AWQFPARTLFMLPIQLMHHDARWFPEPAAFRPQRFGDDAPAIPRGAYLPFGTGPRVCLGQ
HLAMTEMTVAAAMILQRHALSVPPGMAPPRATLNVTLRPERALHLAIAPTSPAANAAAS

>AOI65315.1|NCBI|CYP1013A3|[Burkholderia territorii]btei|BP

MSMKRLAPGPRGSVVMGNLAEYKRNPVTMLLKLQQQYGDIARNRLGPFLTHALAHPDGIQ
HVLQDNHRNYVRGRFYDNFKMFFGDGLLTTDGEFWRRHRRVVQPLFHRKQVESHTAALGD
AALALVERWLKRPAHEPFDAVEEMMRVSLRMLGLMLFNADISRHADDVGPAVRFGIAAMM
PQGNLNDFVPRWMPTPFNRRIAHARRGIDAIVDAIVAEHRAGRCETSDVISLLLAARDPE
TGAPLTEREVHDEVMTVFLAGHETTGSGMAWGLYALAQHPDVLRRLRDELDAVLGGRAPD
AADLERLPYLAQTVDEVLRLYPPIWGFTRDLVDDDEIGGYHVPAGSSVFMSPYVTHRHPA
LWANPDAFDPENFGSHAPARHKYAYFPFGGGMRKCIGYQTALLQIRVLIAVVVQHVDLSA
VPGHSLDTGATISLRPRDGIRLIAKRRSRERGGTGRAGADPGTPADRASVCPFSGAQAQA
QAQAQAPAPSPTPSTAPPANAASPTPVRSAGPALAPDMLAFLQRDPVTDEASPGADATPT
HRFTWRPVEIEPLPDLPAAELSGKRIAIVNGLGRTVERVAATLTRACAKPSVFAPADGVD
PATAASTFFVQSGPFDGIVDLGVETPFSLDVHDQWEAPLRRTVALLQACYGDWSQEASTS
RLFYLALTWQDGLMGHGDDTLVQPLGGLWAGLAKTLPQELPNCNVRVLDIAPDETGRVDQ
LIMRELYRWGRFEVGHRSGRRHTLVAARDALPADAAPDWGADDVVLFSGGARGIGFLAAC
ALAKRHRCTVIVTGRDALPAGDEPWLALDDAGFKRYGLEQLRQAAPGRSPKAIRDTLRQL
QRRRELKASLDAVAALGLPVHYRVCDVTDAHAVRTLCDAFGDRLRGVIHNAGIDQPVRLS
RKSADEFIGTVRVKVLGFVNLYAAVRNRPRVGCFVNVGSLTGRWGGMTGETDYAAANEAL
ARLGWWARRDAGARVVKTIAWPTWDGVGMITNLAVTRRYVTPMRIDEGVRHWLAELATPA
TPTSSGSGEPMYMGAVGRALTPIQLRGFDAVDGLPNLGELATRRHHAGTPRRFRPFSCFS
TRYRTSGAPYTQAFRFDGRAAVPASLLLEHARVVADWVAPPGGERACLHEAVDITIALDA
LADALDSHGDLALDTDANGRTAGDGWHVTVRCASLAGRVLLDATFVYRSHPPGGMTVALP
RETEDAPARDAARARWRDDLLPEARWSGGIAQGKAADPAALWGVQDDAARATCPQLWLPV
NHLENVLRVLLGARTADGEPPRAWRIGRIVFGAAQAGDADMLLRYPDDRFAIADRAGASI
AELYDARLAGATDAQAVAAPDL

>WJ12_27080|KEGG|CYP212A9|[Burkholderia seminalis]bsem|BP

MNPRTSSPCPFHAGAAAPAPVLHPPGVWPPGPRAGLTGWSLLRAMSRDLLGTLAGWQREH
GDVVHLRMWPEHVVAVTDPALVRELLVTHHDALVRWERGIRVFSQVHGHSVLVAEGDAWR
DKRHALQPNFMPKPVQAFVPAIAATAGHALAQWPAHDARWPIESALTSLAMDVIMRAMFS
DAIGEDARVAEAAVRTVSAVSNAEFYQPVSAPDWMPWKRGKARALAVLNGLIDRQLHARL
DRPEHGWPDDLLSRLLRLHRADARVWPLQAVRDECMTAFLAGHETAAATLTWWAWSMAAH
PDVQAAARDEVVRVLDGRAPSTDTRPALRYLTQTLEETMRLYPAAPILISRRASRPVALG
AWQFPARTLFMLPLQLMHLDERWFPEPHAFRPARFADDAPPLPRGAYMPFGTGPRVCLGQ
HLAMTEMTVVAAMLLQRYVLSVPPGTAPPRPVLNVTLRPRQALHLAIASPGLTAEAASAQ
GPA

>WS57_03680|KEGG|CYP102J5|[Burkholderia pseudomultivorans]bpsl|BP

MKSSALVPQPPLKPIIGHLMEVLGPSPLAKMMDLARTYGPVYWFEVFGQGYYVVSGQALV
DEVCDESRFQKSVHQSLLELRPAIGDGLFTAFSDEPNWAKAHRVLMQSFGPLSIWSMFDK
MVDIADQMFLYWERFGPETAVDVSDHMTRLTLDTIALCAFDCRFNSFYREDQHPFVDAMV
NTLSEAGKRELRPKLVSKLMLNRSRKFDADIELMRSLATKMIEDRRKNPHDNEEAMDLLD
RMLNGVDPVTGEKLDDENIVFQMITFLIAGHETTSGLLSFATYFLLKNPDVLQRARDMVD
EVVGNDTPRIEHLARLRYIEQILMETLRIWPTAPAFAVTPLADTTFGGKYPVSPNDIIMI
LTPMLHRDVSVWGEDVEAFRPERFEPENAEKLPPNSWKPFGNGARACIGRPFAMQEAHLV
LIMLLQRFDFSFADPNYELQVAETLTLKPTGLRINVRPRARGVRKVSNDNLRVRASIETP
NVDQAPAVSDKDASKMLVLFGGNTGSSESFARRIAGDAGRHGFHATCAPLDAFAGKIDGF
PAIVVVTASYEGQPPDNAIAFLPYIEAMSEGALDGVKFSVLGCGNKQWARTYQAIPKRVD
AAFEKAGATRVHARGELDSGGDFFGEFDRWYAGFWNRFAISFGKDTSVAQSSNSALNVSF
LNNVREKLLSVDDMTHATVIDNRELVDISVPGSRSKKHMELKLPETMTYRSGDYLAVLPR
NSKRNVDRVLRRFRLSWDTQVVIDGTSSNPRLPLGQAISCGELLSSYVELAVPATRSQVS
ILAAATRCPPEKVELERLSTGDFEYEILEKRTTVMDLLERFGSVDLSFDKFLDMLPALKA

RQYSISSSPLWKADHVTLTVAVVDAPALSGNGRHEGVASSYLARLDAGDSVSVSVRPSNA
RFRPPVESNLPMILICAGSGIAPFRGFLQERAIQKQRGENVGPSLLFFGIDDPDVDFLYR
DELEEWANLGVVEVMPAYSNRPEEGARFVQDKVWLEREKIAALFAQGATVYVCGDGKNMA
PAVRATLGRIYQDSTGANDESAHAWIDTMEREHGRYVADVFA

>WT74_26050|KEGG|CYP212A19|[Burkholderia stagnalis]bstg|BP

MNTRSSSQCPFHAGAPTPALHPPGVWPPGPRAGLTGWGLLRAMSRDLLGALGGWQQAFGD
VVHLRMWPEHAVAVTDPQLVRELLVTHHDAVARWERGIRVFSQVHGHSVLIAEGDAWRGK
RHALQPGFMPKPVQSFASTIASTAGHALAQWPAHDPHWPIESALTSLAMDVIVRTMFSDV
IGDDARAAEQAVRTVSAAANAAFYQPAIAPDWMPWKRAKARAQAVLNGLIDRQLHARLDK
PDSAWPDDLLTRLLRLHRADARAWPLRAVHDECMTAFLAGHETTAATLTWWTWCMASNPA
AQDAARDEVARVLGGRAPTTETRPALRYLTQTLEETMRLYPAAPILISRRASRPIALGGW
QLPARTLFMLPIQLMHRDARWFPEPHAFRPERFADGAPAVPRGAYMPFGTGPRVCLGQHL
AMTEMTVIAAMVLQRFVLSAPAGVAVPKPVLNVTLRPDRALHLGIAAVPA

>BBJ41_20335|KEGG|CYP212A7|[Burkholderia stabilis]bstl|BP

MNTRSSSQCPFHAGTAAPAPVLHPPGVWPPGPRAGLTGWGLLRAMSRDLIGTLAGWQRAY
GDVVHLRVWPEHAVVVTDPALVRELLVTHHDALGRWERAIRVFAQVHGHSVLVAEGDAWR
DKRQALQPNFMPKPVQSFVPAIAATAGHAIAQWPASDPDWPIESALTSLAMDVIMRTMFS
NAIGDDARAAEDAVRVVSAAANAAFYVPASTPDWMPWKRGKVRAMAVLNELIDRQLHARL
DMPERIWPDDLLSRLLRLHRADARRWPLRAVHDECMTAFLAGHETTAGALTWWAWCMATN
PAAQAAARDEVARTLGGRAPSAETRSSLRYLTQTLDETLRLYPSAPILISRRASRPIALG
AWQFPARTLFMLPLQLMQRDARWFPEPDAFRPERFAGDAPAAPRGAYMPFGTGPRVCLGQ
HLAMTEMTVVAAMVLQRYVLSVPPGMTPPREVLNVTLRPERALHLAIAPTGLTADTVSA

>JFN94_24295|KEGG|CYP212A9|[Burkholderia anthina]bann|BP

MNPRSSSRCPFAAGAAGASPALHPPGVWPPGPHAGLTGWRLLRAMSRDLTGALAGWQRAH
GDVVHLRMWPEHAVVVTDPALVRELLVTHHDALVRWERGIRVFSQVHGHSVLVAEGDAWR
DKRHALQPNFMPKPVQAFVPAIAATVGQALARWPAHDAHWPIESALTSLAMDVIMRTMFS
DALGADARVAEDAVRTVGAAANAEFYQPASVPDWAPWKRGKARALAVLNGLIDRQLRVRL

DLPAQDWPDDLLSRLLRLHRADTHAWPQQAVHDECMTAFLAGHETAAATLTWWAWNMAAH
PVAQAAARDEVVRALGDRPPTADTRPALRYLTQTLEETMRLYPAAPILISRRAVRPVALG
AWQFPARTLFMLPIRLMHHDARWFPEPHAFRPERFADDAPAIPRGAYMPFGTGPRVCLGQ
HLAMTEMTVVAAMILQRHVLAMPSGTPPPRPVLNVTLRPEQPLHLTIAPTGPTAVMVSA

>JFN94_28710|KEGG|CYP229F1|[Burkholderia anthina]bann|BP

MNPTDPIAAVTHRDPYPYYATLVDGPPLAFDATLGLWVASRAASVTAVLGHPACRVRPLD
APVPSALRGTTAGALFGELVRMNDGALRHDVPKQALRTAFAPIDAYALRERAAQLAGSRL
RALHDADALNAWCLTVPVCAVADLLGFDDTQLDEIAARVVDFVAALSPLSDDAALARASE
AARELLDRMTERVAHARAQDGSLVAAVQQAARDAGWQASGARVANLVGLLSQTCEATAAW
LGNVLVAWDATAAHRTERERPMPNAAALDEFVAEVGRFDSPVQNTRRFVASRTTIEGVSV
DAGAAILVVLAAANRDPAVHRDPHRLLPGRAPGPNFGFGTGPHGCPGERIARAVTAGAFD
AWLGACGGAPRDRLVWSYRASTNVRMPNFGTPR

>bglu_2g21150|KEGG|CYP107DN1|[Burkholderia glumae]bgl|BP

MSDPEIQQVSLYDLFQPRWRADPYPLYRRLRDESPVHADPNGNGWIVSRHADVVAVLDDA
RFSAARLGGIEDPGGAEQLIRRTLSRQMLFLDGADHTRLRKLFAKAFTPRRLDTFRPRIE
ALAQRMLAARKGHTIDFIGEFAVPLPVMVIALVLGVPMDAHAQLHDWSLAFGSVISGRML
SEAETAHAEAGIAAFGAYFAALIRERRREPCDDMLSDLLAAEEAGDRLDEPELIANLILL
LAAGHATTTHLLGNGLLALSREREQWRLLADDPAIAPAAVNELLRFDAPVQLTGRAARED
VELGGQRLPKGAYVYTLLGAANRDERHFAAPDTLDVRRPVSRVLSFGHGVHTCLGALLAR
IEAQIVFTSLAALYPGIEVDAQQAQRTESLSFRGLRSLPVRLV

>KS03_3812|KEGG|CYP107DN1|[Burkholderia glumae]bgu|BP

MSDPEIQQVSLYDLFQPRWRADPYPLYRRLRDESPVHADPNGNGWIVSRHADVVAVLDDA
RFSAARLGGIEDPGGAEQLIRRTLSRQMLFLDGADHTRLRKLFAKAFTPRRLDTFRPRIE
ALAQRMLAARKGHTIDFIGEFAVPLPVMVIALVLGVPMDAHAQLHDWSLAFGSVISGRML
SEAETAHAEAGIAAFGAYFAALIRERRREPCDDMLSDLLAAEEAGDRLDEPELIANLILL
LAAGHATTTHLLGNGLLALSREREQWRLLADDPAIAPAAVNELLRFDAPVQLTGRAARED

VELGGQRLPKGAYVYTLLGAANRDERHFAAPDTLDVRRPVSRVLSFGHGVHTCLGALLAR
IEAQIVFTSLAALYPGIEVDAQQAQRTESLSFRGLRSLPVRLV

>BC1001_1737|KEGG|CYP195A3|[Burkholderia sp.]bug|BP

MPTATAGDPAALARDFDLRRLSPDFYADPYPVYHALRAHEPVKRMPDGSLFLTRFRDVQA
VYRDPKTFSSDKTVEFRPKYGDSPLYTHHTTSLVFNDPPRHTRVRKLIAGALTARAIAAM
EPGLVRLVDGLLDAAAERGRIDLIGDFASAIPVEIIGNLLDVPHAERAPLRDWSLAILGA
LEPSLTDAQFERGNRAVSEFVDYLRDLVARRRRAPGDPQHDVLTRLIQGEAGGEQLSEEE
LLQNCIFILNAGHETTTNLIGNGLVTLTEWPDQRAALLAEPALIESAVEECLRFESSNQL
GNRMATVDTEIGGLPVARGTPVTLCIGAANRDPEQFPDPDRFDIRREPNRHLAFGFGIHQ
CAGLSLARLEARIAIDRFVQRFPSYRLDGEPTRGGRVRFRGFAEVPLLCR

>BC1001_3509|KEGG|CYP107DQ2|[Burkholderia sp.]bug|BP

MSMSLMQPAAFAPAFDPLVPHPAHVRDPYRVYAQLREHAPMYRSPHGVWIASRYAEVAFM
LKDARFGRGYFYFENLAHRLGAGIVEQPVYSSARNMMVMKDGRDHLRLREVVARSFLPRR
IEAMRPFMLSVMYELIEAARKKPTFDVMLDLAFPLPSAVICHMIGVPKEDWQKFCQRKAN
GSRALEPAPLSPLEMFEQNEAVNEARQYFEWLLELRRQHPGDDLTSQLAAAENAGLITRA
EAVDNLRMMFVGGQETTVNTIGNGLLALYRNPEQLHALRNDLTLLPRAVSEMVRYDSAVQ
MTPRQAREDVEVAGASIQSGETILCILASANRDAAAWPDADRFDVTRTSNHPLSFGGGPH
FCLGAHLGQVETEVALQAIFEHLPNLQPDIEEPRWLPGTVVFRGLQTLPATA

>BC1001_6037|KEGG|CYP1485A4|[Burkholderia sp.]bug|BP

MPEPESFSVLPSIIGTGSADPYPLYEWLRAHRPLCRDASGVWLASSHALVTRILDDARFS
ARPPPGAVPLDDPLGYTNMVVFQTGEAHERLRRFLAPLFSRKALLSLQAFIDDEVARLID
QCERLPRFDLVAHIASVLPVRTICRLLGFPDEHGASYLQASAGAWQLISGAPMSAEERAL
AALQTETFIAHVESFMLAIEPDVVPDHAAARFLRLEQEGGIGRRDMLVNVLFLFIAGYGT
TLLSIGNSIAALLRAPRAWQALKDDVSLVPQAVRELYRYDPAVQSVFRYAWDDVEIDGCR
IRRGERVMPLIGAANRDPAAFAAADEIRFEREAGRSLTFGAGPHSCMGLALARMQLESLL
SALMRRMPGLRLAPGEQPRQQHGSFHGYDRLMVEH

>BC1003_1566|KEGG|CYP195A3|[Burkholderia sp.]bgf|BP

MPTATADDPAVLARDFDLRDLSPGFYANPYPVYHALRTHEPVKRMPDGSLFLTRFRDVQA
VYRDPKTFSSDKTVEFRPKYGDSPLYTHHTTSLVFNDPPRHTRVRKLIAGALTARAIAAM
EPGLVRLVDGLLDAAAERKRIDLIGDFASAIPVEIIGNLLDVPHAERAPLRDWSLAILGA
LEPSLTPAQFERGNRAVSEFVDYLRDLVARRRRKPGDPQHDVLTRLIQGEAGGEQLSEEE
LLQNCIFILNAGHETTTNLIGNGLVTLTEWPDQRAALLAEPGLIESAVEECLRFESSNQL
GNRMTTVDTEIGGLPVARGTPITLCIGAANRDPDQFPEPDRFDIRREPNRHLAFGFGIHQ
CAGLSLARLEARTAIGRFVQRFPAYRLDGEPVRGGRVRFRGFAEVPLLCA

>BC1003_3437|KEGG|CYP107DQ2|[Burkholderia sp.]bgf|BP

MSMSLMQPAAFAPAFDPLVPHPAHVRDPYRVYAQLREHAPMYRSPHGVWIASRYAEVAFM
LKDPRFGRGYFYFENLAHRLGAGIVEQPVYSSARNMMVMKDGRDHLRLREVVARSFLPRR
IEAMRPFMLSVMYELIEAARKKTTFDVMLDLAFPLPSAVICHMIGVPKEDWQKFCQRKAN
GSRALEPAPLSPLEMFEQNEAVNEARQYFAWLLELRRQHPGDDLTSQLAAAETAGLITRA
EAVDNLRMMFVGGQETTVNTIGNGLLALYRNPEQLHALRNDLTLLPRAVSEMVRYDSAVQ
MTPRQAREDVEVAGASIRSGETILCILASANRDAAAWPDADRFDVTRTSNHPLSFGGGPH
FCLGAHLGQVEAEVALQAIFEHLPNLQPDIEEPRWLPGTVVFRGLQTLPATA

>BC1003_3622|KEGG|CYP1485A1|[Burkholderia sp.]bgf|BP

MTCAIADPGSHSVLPSIIGTGSGDPYPLYEWLRAQRPLWRDASGVWLASSHALVTRILDD
ARFSARPPPGAQPLDDPLGYTNMVVFETGEAHERLRRLLAPLFSRKALLSLQAFIDGEVA
RLLDQCEALPRFDLVAHIASVLPVRTICRLLGFPDAHGASYLQASTGAWQLISGAPMSVE
ERTHAAVQTETFIGNVDAFLREVDPDAAPDHAASRFLRLEQEGGIGRREMLVNVLFLFIA
GYGTTLLSIGNSIAALLRAPHAWQALVDDTSLVSQAVRELYRYDPAVQSVFRYAWDDVEI
DGCRIRRGESVMPLIGAANRDPTVFAAPDELRFEREAARSLTFGAGPHSCMGVALARMQL
ESLLRALLRRMPELRLAPGWQPRQQHGSFHGYDRLIVER

>BC1003_3801|KEGG|CYP133C4|[Burkholderia sp.]bgf|BP

MNFATFSSPAFFADPYPLYEKVREAGPLLSAGPSVLVTGRFEIIDALLRDRRMGKTYLQS
VRARYGDAATHYPVFQALSRTFLMMNPPSHTKLRTLLMKAFNARQIEKLKDIVEATSGAL
LDRIGSKAEFDLVREYALPLPVEIICRLLAIPSSHGTELGEAASRLVAAFDLAPLDEAAL
GAANEAALTLERYFLEVVEQRRADPGDDIISSLASVEDEGTKLTDDEIVSNVILLFIAGH
ETTSNMIGNAMVALFRHPPQLEMLKRDSTLVSKAVAECMRYDGAVQMVVRTAFEEVVMKD
VTLPAGSIVFMLIGAANRDPAVFSHPDRLDFARGANVPSLSFGAGIHHCLGARLALLELE
VAIGSLLARFPDMQPTQLDQLRWHARNNLRGVQELRIRT

>bgla_1g20160|KEGG|CYP133E1|[Burkholderia gladioli]bgd|BP

MQYSDLATPDFLKNPYPVFDAMRAEGQLVKLAPKLYASSHYGFGEKLLLDRRFGKGIVEA
VKARYGEGVIDQPPFRTFRSMLPGLNPPKHTNLRALLMKSFNARQVEKFREASYTISNQL
IDRLVKEPQGDLVTGFAFLLPMQTMCTILGVPLSDGAMFKRAADRAAGALNVTPLNAEQL
EESRKSAIELETYFAKVLAERRKEPGDDLISQMILAEEDGQRLTDDEIVANLCFLFVAGH
ETTENMIGNTLIALQRHPEQRERVKADLSIMPRVVTEALRYDSSVQIAQRVALEEVELEG
QTIQRGDLICVFLGGGNRDPEKFENPDRLDIDRENVRPLSFGGGLHYCLGARLALLEIAA
ALEVVYTRLPNLHLTNLDALPYRRNNALRGVDSLLGTW

>bgla_1g21680|KEGG|CYP267C1|[Burkholderia gladioli]bgd|BP

MDTAVTPVVPALYSAEFFRNRDPYPTYQKHLEGPVVQSSAMMPNTWLMFRYAACSTHLRD
PNLSAARPPKLLVPVPEADLPRFEDMASHLQRWLLMRDQPSHKPLRKLMNKGFSPLTIQQ
MRPRIELLVERLVDDLRAAPEPDIMRDFAYPLPVRVICELLGVPDALQDRCVVLTDDIAV
LLANPHVTTEAAECAQDAVRELAAYFRDIIRDHEDDGEENLLNLLLHIADDDEQQMSEEE
LLAQCVMLLFAGHETTRNLIGNGVYTLLRHPESLAAVRADPERVKSAVEEVLRFESPLQA
VRRVTTAEVEFDGTRIPAGHTVIFVTAAANRDPRQFEDPDRFDIDRRLNRHLAFGADHHV
CIGSTLARLEGQIAIDAIMRAFPNLQLLDQTPDWRGHAAMRGFRTLRVGI

>bgla_2g00750|KEGG|CYP133C4|[Burkholderia gladioli]bgd|BP

MDNTFLIRPATVLMNFATFSSPAFFANPYPLYAQLRAKGALLELAPNVRVTGDYALIDAL
LRERRAGKTYLESVRVRYGEERVGMPVFQVLSRMFLMMNPPVHTRLRKLLMKAFNARQIE

NLREIVEASTHELLDAIAAKPEFDFVADYALPQPLNVICRLLDIPFEDGARLGRAASCVA
SALDLAPLGEAMLAEANEGALALEAYFEGVIEQRRHQPGTDLVSALISVEDEGETFDEKE
VIANVMLLFIAGHETTSNTMGNAMIALFRNPESLAVMTADPALVPGGVAELVRYDSSVQV
VVRTVFEDLELEGHALPRGTLLFLLVGASNRDPAVFADPDRLDIRRANAKASLSFGGGIH
YCLGARLAQMELETAIGAMLARFPKLSPVDLDRLEWHHRNNLRGVKALPMRTGVN

>bgla_2g27800|KEGG|CYP107DN2|[Burkholderia gladioli]bgd|BP

MRDSEVSPFSMYQLMEPAQRADPYPWYRRLRDARPICRDPHVGSLLVARHAEVAAVLENP
AFSAARLGMSESDDPVEREIGRTLGEQMLFLDPPDHTRLRKLFAKAFTPRRLDAFRPRIE
ALTHQLLDACAGRRADFIGEFAVPLPVMAIALVLGVPTGERERLHAWSLAFGSIISGRPL
TVEEVGQAQRGIAEFRQYFAELIAARRAVPEDDMLSDLVAAEEAGDRLDEGELISNLILL
LAAGHATTTHLLGNGLLALAGEREQWRRFVADPSLAPTLVSELLRFDAPVQVTGREARED
VELDGMTIPKGQYVHLLLGAANRDERQYAEPDRLDIGRTGTRMMSFGHGIHTCLGAVLAR
IEAQIVFGALAERFPDFEVHEQDAERTATISFRGLRSLPVTLR

>BM43_3355|KEGG|CYP133E1|[Burkholderia gladioli]bgo|BP

MQYSDLATPDFLKNPYPVFDAMRAEGQLVKLAPKLYATSHYGFGEKLLLDRRFGKGIVEA
VKARYGEGVIDQPPFRTFRSMLPGLNPPKHTNLRALLMKSFNARQVEKFREASYTISNQL
IDRLVKEPQGDLVTGFAFLLPMQTMCTILGVPLSDGAMFKRAADRAAGALNVTPLNAEQL
EESRKSAIELETYFAKVLAERRKEPGDDLISQMILAEEDGQRLTDDEIVANLCFLFVAGH
ETTENMIGNTLIALQRHPEQRERVKADLSIMPRVVTEALRYDSSVQIAQRVALEEVELED
QTIQRGDLICVFLGGGNRDPEKFENPDRLDIDRENVRPLSFGGGLHYCLGARLALLEIAA
ALEVVYTRLPNLHLTNLDALPYRRNNALRGVDSLLGTW

>BM43_6819|KEGG|CYP133C4|[Burkholderia gladioli]bgo|BP

MDNTFLIRPATVPMNFATFSSPAFFADPYPLYAQLRAKGALLELAPNVRVTGDYALIDAL
LRERRAGKTYLESVRVRYGEERVGMPVFQVLSRMFLMMNPPVHTRLRKLLMKAFNARQIE
NLREIVEASTHELLDAIAAKPEFDFVADYALPQPLNVICRLLDIPFEDGARLGRAASCVA
SALDLAPLGEDMLAEANEGALALEAYFEGVIEQRRRQPGTDLVSALISVEDEGETFDEKE
VIANVMLLFIAGHETTSNTMGNAMIALFRNPESLAAMAADPALVPGGVAELVRYDSSVQV

VVRTVFEDLELEGHALPRGTLLFLLVGASNRDPAVFADPDRLDIRRANAKASLSFGGGIH
YCLGARLAQMELETAIGAMLARFPKLSPVDLDRLEWHHRNNLRGVKALPMRTGVN

>BM43_6846|KEGG|CYP183AG1|[Burkholderia gladioli]bgo|BP

MDIDLTDKPAHVDPAPLPEAPIPRAPGSLPIVGHAARLLFKPLSFLSGLAPLGGLVRVDL
GRLPMLLLTDPELTRQVLNDSRTFDKGGALFDKVRELTGNSLLTSRYDDHKLQRRQMQPA
FSRKRILTYIDVMNEAIGGVIGGWCDGDVVDLTAACYDITARTASRTMFAADVAGAAAAS
LVEDLSVYLGGLFVRMMLPSGLPGRVPTPGSLRYDRAVARLHAAIEQIIDAYRASGVDHG
DLLSMMLAARDEDGQPWSQQEVHDQVITLFLAGIETTAGVMSWTLYLLGRHPDIAERAAA
EVRTTLGDAAVPQAEHLMQLGETRRAMMEALRLYPPGWIFTRVVTRDTMLGPYRLRVGDG
LAYSPYVLHRNPALFAAPERFDPDRWLPERAAAIPEHGFIPFGGGVHKCIGDQFGTTENL
LALAAILSRWKLEPVTSQPVEPIPRRATLSPSRFMVRLRRR

>BYI23_B006960|KEGG|CYP1220A11|[Burkholderia sp.]byi|BP

MNATSVGAPLREVDDLPSPRGLPLVGNLHQLPPATHHLTLERWAAELGTPYVFRFGTTPV
TVWNDIELSHAVLRERPHRYRRYAPIENILKEFGCNGLFSIEGAAWEPQRRLVMQALSIP
HIKAFYPTLAAIAERLRRRWEAAARAGRVVDMTGDLKRFTVDVTSALAFGEDPNTLENER
VLIQEQLAMLLPMLMSRVNAPFAYWHYVKFPRDRKFDEAMSAVHRHIRELMARTRARMRE
QAAQTSESAAPQNLLEAMLAMRDAPGSGITDDEVAANVLTMLVAGEDTTATSIAWALLFL
GTDDALQARVAADSRRVLGGSAVCPDYTAMRELDLCEAVCTEASRLHPIAPYLSLEPLEE
VRLQGVRLPAGTKMFLLLNRPAMLDPHNFADPRRYDPDRWLHDQYREGAHESRAYLQFGAG
PRVCPGRHLAAVEMRLVVSMLTANFIAQLATNPADIHEICAFVSVPSEMPMRLVLR

>BYI23_D004940|KEGG|CYP1112A12|[Burkholderia sp.]byi|BP

MTSVPQSGIDLWADDVLVDPYPSYRALRELGEVVYLTHSKLYALTGYDVIKDALADPTRF
SSAQGIGFNDGVNQALQGTSLASDPPVHTRLRAALSENLTPRALRGLGEQIKANAQALVA
RLVKQPSFDAIDDIARRFPIGVVADLVGFQGDARANMLRWGQAAMEVVGPMNQRTAEHFP
IASELYGYCSQITQDALTHGSVGWGVFEAERKGIIPEGAARHIIHQYLGAGLDTTLAAIG
NTVALFGRFPEEFAKVREDLSLVPSAFNEVLRYYSPVHVWSRVATQDVEVGGVKIPGGSR
IAIILGAGNHDPRHYKDPEVFDVTRNPIDHLSFGYGPHGCAGQGLARMEGAAIIEALATQ

IKSFRIGDVARVPSNITLSFDTVPVMDLEAV

>BYI23_D011260|KEGG|CYP116B124|[Burkholderia sp.]byi|BP

MTAAPQCPFHDHAPNGCPVSPRAAEFDPFGDGYQQDPPEYVRWAREQEPVFWSPKLGYWI
VTRYDDIKAIFRDNITFSPSIALEKITPTGPEANAVLASYGFALNRTLVNEDEPAHMPRR
RALMDPFTPEALAQHEPMVRALAREYVDRFIDEGRADLVDQMLWEVPLTVALHFLGVPEE
DMDTLREYSIAHTVNTWGRPKPEEQVEVAHAVGKFWQFAGKVLDKMREDPSGPGWMQYGI
RKQKELPDVVTDSYLHSMMMAGIVAAHETTANATANALKLLLQHPDAWRDICDDPSLIPN
AVEECLRHNGSVAAWRRLATKDVTIGGIEIAAGSKLLIVTSSANHDQHQFADADLFDIRR
ENASDQLTFGYGAHQCMGKNLARMEMQVFLDELTRRLPHMRLAEQRFTYVPNTSFRGPEH
LHVEWDPAMNPERANPSVREARAVVRIGEPSSHAVSRAVIVDSVDASADRIVRVRLVSAD
GRDLPRWTAGSHIDVVCGETGLSRQYSLCGDPDDRRAFEIAVLRETDSRGGSAWVHDNVK
PGAHWRIRGPRNHFRLDETAKKLVLIAGGIGITPISAMARRARTLGIDYELHYSGRSRAS
MTLVDEMRALHGERLVLHMSEEGARNDFTTLAVDARTQVYACGPARMLDALADASASWPE
DALKIEHFESTLATLDAEKEHAFEVELKDSGLVLAVPADQTLLTALRRANVDVQSDCEEG
LCGSCEVRVLEGEIDHRDVVLTKGERQANNRMMSCCSRAKGKRLVLAL

>BYI23_D012450|KEGG|CYP105T2|[Burkholderia sp.]byi|BP

MMAHERAERAEPPFPFGRPDAPLSPPPEYACLRANQPLSKVRLWDGSTAWIATRWEDVRA
ILGSPFFSVDPFRPGYPSVSPARAVQARSRQAFINMDDPDHARFRRMLTKDFMQKRMSDL
RPMVRQFVDELIDRMIEHGPPLDFVEHFSQPLPSMVISALLGVPYEDHARLGEWSTTRND
HTAAPEVVQEAVRKMEAHLGRIVAQKESDPGDGRDMLSRLVIEQIHPGHVTREEVVRIAS
LLYSAGHGTTGSQIGLGTLSLLMNPAQRAELDADPSLLAGAVEEMLRFHTIAHFNSARVA
TADVTIGGQLIHAGEGVYPLIFAANRDPDVFPQPDTFDIHRNPQEHVAFAYGIHQCLGQP
LARLELQTVFSMLFKRLPELRLAVPVESLAFNSTSQVYRLEALPVTW

>BYI23_D012630|KEGG|CYP105CV1|[Burkholderia sp.]byi|BP

MWDGKDAWLFTRYDDVRAVLGDNRFSGDPHIDGFPSLSPARNAVLDLEPAFIRMDPPQHG
HFRRMLTKEFMIKRVSEMRPRIDSIFNRLVDELLEKGPPANLVEDLFLPLTSEVIAGLLD
VPASDHGFFQEQSRLKVLLDVDPSIPKAASDRILSYLDRLITERAKDADNRTDLLSRLIV
EQVRPGHLTHRELVIMAELLLMAGHETTANQMALGVFSFLTNPDQLRLLRETPSLLRNAV
EEMLRFHTIVHYNAFRVATEDVDVAGQTIRKGEGVIALISGANHDATAFEQPDRFDITRK
ADHHVAFSYGIHQCLGQPLARVELQVVFATLFERMPDLRFAVPLEEIKGKGDHFVQGLET
LPVTW

>BYI23_D012740|KEGG|CYP105T3|[Burkholderia sp.]byi|BP

MAPPSQYAMLREQAPVVQVSLWDGSLAWLVTDMEHFREVMTSPHFSASPLTPGFPFVSPS
RAAQSKSYQTFITMDPPEHGHYRRTLTKEFMAKRMQALRPLVQRSLDELLDDMERHGAPA
DFIEAVALPLPSLVISIMLGVPYEDHDKLQKWSGNRMDLSIAPEELTRSAQHMNQYIDAL
LLEKERDPGDGSDLLSRMAIEWINPGKVSHADAVQMATLLYLAGHETTANQIGLGLLSLF
QHPEQRAALMADSSLVKGAVEEMLRFHSITHMNSNRVATEDVVIGGQLIRKGEGVLALLH
GANHDPLAFPEPESFDIRRDTKDQSHVAFSFGIHQCLGQPLARLELQVVFETIFKRFPHL
ELAVPEDALRYKDKSFVYGLKALPVRW

>BYI23_D012760|KEGG|CYP105T4|[Burkholderia sp.]byi|BP

MHPPPRYAVVRESDPIKRVKAWDGSDVWVVTKMKDFRDVLTSPHFSASPETHGYPSVSPA
RAAQSKSYQTFITMDPPEHGQHRRTLTKEFMMKRIDELRPFVQETLEALLEQMIGKGAPA
DFIQDVALPLPSMVISIMLGVPYQDHARLQQLSSNRMNLDISPELLRQSAQEMEQYIDCL
LREKERDPGEGKDLLSRLAIEWINPGKLSHADAVQMGVLLYLAGHETTANQIGLGLLSLL
EDPGQVKALMADPSLVKGAVEEMLRFHSITHMNSARVATADVMIGGQLIRKGEGVFAPVQ
AANHDPEAFPEPDRFDIRRDGKGASHVAFSFGVHQCLGQPLARLELQVLFETLFKRLPTL
RLAVPFDALEFKRNGNVFGLVSLPVEW

>MYA_5060|KEGG|CYP229F5|[Burkholderia sp.]buk|BP

MNPTDPIAAVTHRDPYPYYATLVDGPPLAFDATLGLWVASRAASVAAVLGHPTCRVRPLD

APVPPALRGTTAGALFGDLIRMNDGALRHDVPKQALRAAFASIDAHALRERTASLAGRRL
RAPLDAEALNAWCLTVPVCAVADLLGFDEAQLDDIAALVVAFVAALSPLSDDATLARASD
AARRLLERMTERVARTRARDGSLVAAVQDAARAAGWQAGGALVTNLVGLLSQTCEASAAW
LGNALIAWEESARASADGGRAIPDAAALDAFVAEVGRFDSPVQNTRRFVASHTTIEGVSV
DAGAAILVVLAAANRDPAVHREPHRLLPGRAPAPTFGFGKGPHGCPGERIARAVTTGAFD
VWLHASSGELPSGLVWDYRASTNIRMPNFGIASQA

>BRPE67_BCDS09300|KEGG|CYP1220A10|[Burkholderia sp.]bue|BP

MNATSVGAPLREVDDLPSPRGLPLVGNLHQLPPATHHLTLERWAAEFGTPYVFRFGTTPV
TVWNDIELSHAVLRERPHRYRRYAPIENILKEFGCNGLFSIEGAAWEPQRRLVMQALSIP
HIKAFYPTLAAIAERLRRRWEAAARAGRVVDMTGDLKRFTVDVTSALAFGEDPNTLENER
VLIQDQLAMLLPMLMSRVNAPFAYWHYVKFPRDRKFDEAMSAVHRHIRELMARTRARMRE
QAVQTRESAAPQNLLEAMLAMRDTPGSGITDDEVAANVLTMLVAGEDTTATSIAWALLFL
GTDDALQARVAADSRRVLGDSAVCPDYTAMRELDLCEAVCTEASRLHPIAPYLSLEPLEE
MRLQGVRLPAGTKMFLLNRPAMLDPHNFADPRRYDPDRWLHDQYREGAHEPRAYLQFGAG
PRVCPGRHLAAVEMRLVVSMLTANFIAQLATDPADIHEVCAFVSVPSEMPMRLVLR

>BRPE67_DCDS01110|KEGG|CYP105T2|[Burkholderia sp.]bue|BP

MQNDADVMAHEPAFPFGRPDAPLSPPAEYACLRANQPLSKVRLWDGSTAWIATRWEDVRA
ILGSPFFSVDPFRPGYPSVSPARAVQARSRQAFINMDDPDHARFRRMLTKDFMQKRMSDL
RPMVRQFVDELIDRMIEHGPPLDFVEHFSQPLPSMVISALLGVPYEDHARLGEWSTTRND
HTAAPEIVQEAVRKMEAHLGRIVAQKEADPGDGRDMLSRLVIEQVQPGHVTREEVVRIAS
LLYSAGHGTTGSQIGLGTLSLLMNPAQRAELDADPSLLAGAVEEMLRFHTIAHFNSARVA
TADVTIGGRLIQAGEGVYPLIFAANRDPDVFPQPDTFDIHRNPQEHVAFAYGIHQCLGQP
LARLELHTVFSMLFKRLPELRLAVPVESLAFNSTSQVYRLEALPVTW

>BRPE67_DCDS01290|KEGG|CYP105CV|[Burkholderia sp.]bue|BP

MSGCPFSKPAFPMPRQCPFVPPAEYRDFQQQPGPVKVQMWDGKDAWLFTRYDDVRAVLGD
NRFSGDPHIDGFPSLSPARNAVLDLEPAFIRMDPPQHGHFRRMLTKEFMIKRVSEMRPRI

DSIFNRLIDELLEKGPPANLVEDLFLPLTSEVIAGLLDVPACDHGFFQEQSRLKVLLDVD
PSIPKAASDRILSYLDRLITERAKDADNRTDLLSRLIVEQVRPGHLTHRELVIMAELLLM
AGHETTANQMALGVFSFLTNPDQLRLLRETPSLLRNAVEEMLRFHTIVHYNAFRVATEDV
DVAGQTIRKGDGVIALISGANHDATAFEQPDRFDITRKADHHVAFSYGIHQCLGQPLARV
ELQVVFATLFERMPDLRFAVPLEEIKGKGDHFVQGLEALPVTW

>BRPE67_DCDS01410|KEGG|CYP105T3|[Burkholderia sp.]bue|BP

MLETTATCPFATERFPWPRDGAAPLAPPSQYATLREQAPVVQVSLWDGSLAWLVTDMEHF
REVMTSPHFSASPLTPGFPFVSPSRAAQSKSYQTFITMDPPEHGHYRRTLTKEFMAKRMQ
ALRPLVQRSLDELLDDMERHGAPADFIEAVALPLPSLVISIMLGVPYEDHDKLQKWSGNR
MDLSIAPEELTRSAQHMNQYIDALLLEKERDPGDGSDLLSRMAIEWINPGKVSHADAVQM
ATLLYLAGHETTANQIGLGLLSLFQHPEQRAALVADASLVKGAVEEMLRFHSITHMNSNR
VATEDVVIGGQLIRKGEGVLALLHGANHDPLAFPEPESFDIRRDTKDQSHVAFSFGIHQC
LGQPLARLELQVVFETIFKRFPHLELAVPEDALRYKDKSFVYGLKALPVRW

>BRPE67_DCDS01430|KEGG|CYP105T4|[Burkholderia sp.]bue|BP

MANDTSTQSCPFDNDTFPWPRAAECPLHPPPRYAVVRESDPIKRVKAWDGSDVWVVTKMK
DFRDVLTSPHFSASPETHGYPSVSPARAAQSKSYQTFITMDPPEHGQYRRTLTKEFMMKR
IDELRPFVQETLDSLLDQMIEKGAPADFIQDVALPLPSMVISIMLGVPYQDHARLQQLSS
NRMNLDISPELLRQSAQEMEQYIDCLLREKERDPGEGKDLLSRLAIEWINPGKLSHADAV
QMGVLLYLAGHETTANQIGLGLLSLLEDPGQVKALIADPALVKGAVEEMLRFHSITHMNS
ARVATADVMIGGQLIRKGEGVFAPVQAANHDPEAFPEPDRFDIRRDGKGASHVAFSFGVH
QCLGQPLARLELQVLFETLFKRLPTLRLAVPFDALEFKRNGNVFGLVSLPVEW

>BRPE67_DCDS06740|KEGG|CYP1112A12|[Burkholderia sp.]bue|BP

MTSVPQSGIDLWADDVLVDPYPSYRALRELGEVVYLTHSKLYALTGYDVIKDALADPTRF
SSAQGIGFNDGVNQALQGTSLASDPPVHTQLRAALSENLTPRALRGLGEQIKANAQALVA
RLVKQPSFDAIDDIARRFPIGVVADLVGFQGEARANMLRWGQAAMEVVGPMNQRTAEHFP
IAGELYGYCSQITQDALTHGSVGWGVFEAERKGIIPEGAARHIIHQYLGAGLDTTLAAIG
NTVALFGRFPEEFAKVREDLSLVPSAFNEVLRYYSPVHVWSRVATQDVEVGNVKIPGGSR

IAIILGAGNHDPRHYKDPEVFDVTRNPIDHLSFGYGPHGCAGQGLARMEGAAIIEALATQ
IKSFRIGDVARVPSNITLSFDTVPVMELEAV

>BRPE67_DCDS13390|KEGG|CYP116B124|[Burkholderia sp.]bue|BP

MTAAPQCPFHDHAPNGCPVSPRAAEFDPFGDGYQQDPPEYVRWAREQEPVFWSPKLGYWI
VTRYDDIKAIFRDNITFSPSIALEKITPTGPEANAVLVSYGFALNRTLVNEDEPAHMPRR
RALMDPFTPEALAQHEPMVRALAREYVDRFIDDGRADLVDQMLWEVPLTVALHFLGVPEE
DMDTLREYSIAHTVNTWGRPKPEEQVEVAHAVGKFWQFAGKVLDKMREDPSGPGWMQYGI
RKQKELPDVVTDSYLHSMMMAGIVAAHETTANATANALKLLLQHPQAWRDICEDPSLIPN
AVEECLRHNGSVAAWRRLATKDVTIGGIEIAAGSKLLIVTSSANHDQHQFADADLFDIRR
ENASDQLTFGYGAHQCMGKNLARMEMQVFLDELTRRLPHMRLAEQRFTYVPNTSFRGPEH
LYVEWDPAMNPERANPSVREARAVVRIGEPSSHAVSRAVIVESVDASADRIVRVRLVSAD
GRDLPRWTAGSHIDVVCGETGLSRQYSLCGDPDDRRAFEIAVLRETDSRGGSAWVHDNVK
PGAHWRIRGPRNHFRLDETAKKLVLIAGGIGITPISAMARRARTLGIDYEVHYSGRSRAS
MTLIDEMRALHGERLVLHVSEEGARNDFTTLAVDARTQVYACGPARMLDALANASASWPE
DALKIEHFESKLATLDAEKEHAFEVELKDSGLVLAVPADQTLLTTLRRANVDVQSDCEEG
LCGSCEVRVLEGEIDHRDVVLTKGERQANNRMMSCCSRAKGKRLVLAL

>BW21_3812|KEGG|CYP1486A1|[Burkholderia sp.]bul|BP

MRDMSKVEYDAAKIRPFNIDPQVAAIIKNPERPEAFPPLAKVPKSIPGTSGFIAGMKSLF
AYKREGLQFFANQANIHGKVFRNQLGPYSIVGVVDHEVISQILKNGDRCWSTALAVERVT
RGLSFDIDLKKKSRLMSLDFDAHRQVRTLVQPAFSDRALASYLAIIQQEFTRDIEAWPRD
GEVPFRVEARQIFSQLAARLFFGIEDSSEAARLEGATRDYWNAIPVVLKNRHLNERWRRG
LDGVATLSSIMYDLIPKRRDEGGTDLFSQLCKSREDTGGLDDDQLVDLMLNIVFAAFDTT
TMGITSMAYLLAKHPHWQEVLRAESMRLPEQAALADFNGMERTTQVWKETLRMYPVSAIT
PRVSLGECQVGPYVVPPRTLVFNFTGLLGQDSDLWDHPQRFDPERFSPERAEYQRHRGQY
LPFGHGVHTCVGAALANLEAKVFWSIVLTRYKFSLAKDYDAVHCMNPLGSVSGEVALKVE
RL

>BW21_4258|KEGG|CYP1488A2|[Burkholderia sp.]bul|BP

MSAAAKLALPKNLVNLNRGLHSMREPEHGRHKRLLTGTINRELFDAHRNGIRAALDRLGE
RLKEDGRIAVVSRMRELTSEMASHVFLGPRCHEEDAELAFLLSAYFTLRREASSLNARDP
HVYRAELIGVGQQLDRTLRERIRRYRNGPADDRAGLLRRLATAGLPGSPALSEDEIVGHA
NVMFVSSTEPVAISLTWLLLVLSQLPGLRRALRAEIAGRASSPTSPGGAPLLENVVNETL
RLLTPNALMVRATTRAVSLQGVALPAHCEIVVCPFLAHREAGPFPDPLAFSPSRWETARP
SPYEYFPFGAGGHFCAGRNLALLLIREVLSTLLLRFDFVLDGEQSIDWRIHIMLMPTGDP
TLIAHPADERRDTPSPKWRGPATDLFHFAPGLS

>BW21_4313|KEGG|CYP1013A3|[Burkholderia sp.]bul|BP

MSKYRQAPGPRGSLVMGNLAAYKRNPITMLLRLHQQYGDVARNRLGPFVTHAIAHPDHIQ
YVLQENHRNYVRGRFYDNFKMFFGDGLLTTDGEFWRRHRRAVQPLFHKKQVDAHTAAVGD
AALALAHRWSALPPGKAIDVVEEMMHLSLRMLGLMVFNTDVSSHAEAVGPAVRFGIEAMM
PQGDMNDFIPRWAPTRFNRRIAHARRAIDTIIAKIIADHREARCEPSDVISLLLNARDPD
TGAPMTQQEVHDEVMTVFLAGHETTGAGLAWALYALAQHPAVLRQLRDELDARLGGRAPT
VQDFEQLPYLSQVVDEVLRVYPPIWGFTRDLVEDDEIGGYRIPARSSVFMSPYVTHRHPA
FWRNPDAFDPENFASDAPARHRFVYFPFGGGMRKCIGFQTALLQMRVLVAVVAQHFDLNA
LPGHPIEFGATISLRPVHGIRLIVKPRERQQSHLARVREHESARALRPLGDTVAAAGAAC
PMAAALALDAAHGGGAAVAAAREAARQHEPGDVATLSLATSPATALPAAGTDTDASARSV
PAWRFTWRPVPVDPVPDEPSPALSGRRIALVNGRGGTVERVAAALARTCAKVSVFAPAAN
ADHAEAARVFVNEAGPLDGIVDLGLEAPFALDDASAWEAPMRRTVELLKACYGDWAAEDD
TSRLFYVALTWMDGQMGYGADAGAQPLGGLWAGLAKTLPQELPNCNVRVLDIAPDETGRV
DRLVVNELYRWGLFEVGYRNGVRYTLHAKRNELPPLAVDARSALGPGDAVLFSGGARGIG
LLCARALAGRHGCTVIVTGRETPPDGGEPWMTLDEEGFKRYGLEQLRAATPEHPPKAIRA
SLQRLKRRRELKTALDEMAALGLPVHYRVCDVTDRAAVRALCDELGDSLRIVIHNAGVDE
PARLIRKRADDFARTVRTKVLGFANLCDAVRARPRLAQFCNVGSLTGRWGGMTGETDYAA
ANEALARLGLWAAQHALSPACGVKTLVWPTWEGVGMITNFAVTQRYVSPMNVDEGVRHWL
RELASPGSGEVMFMGAVGRAPTPVQIKGFQPIRELPNIGELVTRRHHVGEPVRFRPFKRF
EGRYAIDPAVAPYAHAFRFNGRAVVPASLVLEHACAVGDWVMPPTGEPRELSEMANVVLR
LDALPAVPAGGAGVEIGSEAAGYMIGDVWCVDVRCSDARTRAELLSMTLVYRPQPEQIGA
AYARAPGATPEPAPLTSAPHAAWNGELLPAGDWSVPDDASAGIVRIGRVPPAAVLSLWTL
PLPLELRLPVNHVEHVLRVLLAEWGAAQTDAPAVWRIGGAKLARLPAASADGVVEYATGR

FAVLDARGYVLLRIDDTRVDGDAAQAMQTETGALAAPLTA

>AC233_07625|KEGG|CYP195A3|[Burkholderia sp.]buq|BP

MSTATADAATLARNFDLRHLSPEFYADPYPVYHALRAHEPVKRMPDGSLFLTRFRDVQAV
YRDPKTFSSDKTVEFRPKYGDSPLYTHHTTSLVFNDPPRHTRVRKLIAGALTARAIAAME
PGLVQLVDGLLDAAAERGRIDLIGDFASAIPVEIIGNLLDVPHAERAPLRDWSLAILGAL
EPSLTDAQFERGNRAVSEFVDYLRGLVARRRREPGDPQHDVLTRLIQGEAGGEQLSEEEL
LQNCIFILNAGHETTTNLIGNGLVTLTEWPDQRAALLAEPSLIESAIEECLRYESSNQLG
NRMTTVDTEIGGQPVARGTPVTLCIGAANRDPEQFAEPDRFDIRREPNRHLAFGFGIHQC
AGLSLARLEARIAIGRFVRRFPSYQLDGEPTRGGRVRFRGFAEVPLLCR

>AC233_33915|KEGG|CYP1485A3|[Burkholderia sp.]buq|BP

MTLTFDVPFTDTRTHADGEPPMTCEIPDPGSYSVLPSIIGTGSGDPYPLYGWLRAHRPLC
RDASGVWLASSHALVTRILDDARFSARPPPGTEPLDDPLGYASMVVFQTGEAHERLRRFL
GPLFSRKALVSLQAFIDDEVARLVDQCEHLPRFDLVANIASALPVRTICRLLGFPDAHGA
SYLEASAGAWQLISGAPMSAAEHALAAMHTEAFLAQVESFMRAIDPHAAPDHAASRFLRL
EQEGGIGRREMLVNILFLFIAGYGTTLLSIGNSVAALLREPRAWQALKDDVALVPQAVRE
LYRYDPAVQSVFRYAWDDVEIDGCRIRRGERVMPLIGAANRDPAVFAAPDEIRFEREAGR
SLTFGAGPHSCMGLALARMQLESLLRALTRRMPDLRLAPGWQARQQHGSFHGYDRLMVER

>BGL_2c05550|KEGG|CYP133E1|[Burkholderia plantarii]bgp|BP

MQYSDLATPEFLKNPYPVFEAMRAEGQLVKLAPKLYATSHYAFGEKLLLDRRFGKGIVEA
VKARYGEGVIDQPPFRTFRSMLPGLNPPKHTNLRALLMKSFNARQVEKFREASYTISNQL
IDKLVKEPQGDLVTGFAFLLPMQTMCTILGVPLSDGAMFKRAADRAAGALNVTPLNAEQL
EESRKSAIELETYFAKVLAERRKEPGDDLISQMILAEEDGQRLTDDEIVANLCFLFVAGH
ETTENMIGNTLIALQHHPEQRERVKADLSIMPKVVTEALRYDSSVQIAQRVALEEVELEG
QTIQRGDLICVFLGGGNRDPEKFENPNALDIDRENVRPLSFGGGLHYCLGARLALLEIAA
ALETVYTRLPNLHLTNLDALPYRRNNALRGVDSLLGTW

>BGL_2c27660|KEGG|CYP107DN1|[Burkholderia plantarii]bgp|BP

MSDPEIQQVSLYDLFQPRWRADPYPLYRRLRDESPVHADPNGNGWIVSRHADVVAVLDDA
RFSAARLGGIDDESRTEQAIRRTLSKQMLFLDGADHTRLRKLFAKAFTPRRLDTFRPRIE
ALAHRMLAAQAGRTIDFVSEFAVPLPVMVIALVLGVPMNAHAQLHDWSLAFGSVISGRVL
SDAETARAEAGIAAFGAYFAELIRERRREPGDDMLSDLLAAEEAGDRLDESELIANLILL
LAAGHATTTHLLGNGLLALSREREQWRLLADDPAIAPAAVNELLRFDAPVQLTGRAVLED
VELGGQRLPKGAYVYTLLGAANRDERHFAAPDTLDVRRPVSRVLSFGHGMHTCLGALLAR
IEAQIVFTSLAALYPGIEVDAQQARRTESLSFRGLRSLPVRLV

>bpln_1g20870|KEGG|CYP1481A2|[Burkholderia plantarii]bpla|BP

MTIPTLIPPSQHTRAVPFPGLIAHAPGPDTDTEHGAAFRAAVIEGGLDASDYFARSGVAE
LASRSGGLCTLWIDNDLAIYQNTNEPLVEDDDLMPSINTNAGLFGSFMGGLDANDPVRVA
KREFVERILGNARFVYGLSDEITAATRAYLDSPLSQDTSLDTFCLNLTAYVDSVIPGILD
LQIRPLTDYLAEPIFGRIATSFFEIASEAISKLNVAAIQDADLIVDLTMRILLDNYQSIE
AAPQGNLIRSQFGNFGIPFSRQGIATLTDAQLKELGTLIVATYDTTALSLLWTIAYLETT
PGARDRLLDSLGDHEVAQQTATMMVLEAIRLGGSNPTSLWRRSRRPVTILHRGSSVDIPA
GTMFWLDRRLANRDPQRFARPNVFDPGNIEGIANHGGIEAASLLARNRYEINSFSMINTV
RNPRKCPGRLFSVRAQALMLTELYRSNRVVVSGVSTGLMPGSAMPRPACPGSIRVEPIQ

>bpln_2g06110|KEGG|CYP133E1|[Burkholderia plantarii]bpla|BP

MQYSDLATPEFLKNPYPVFEAMRAEGQLVKLAPKLYATSHYAFGEKLLLDRRFGKGIVEA
VKARYGEGVIDQPPFRTFRSMLPGLNPPKHTNLRALLMKSFNARQVEKFREASYTISNQL
IDKLVKEPQGDLVTGFAFLLPMQTMCTILGVPLSDGAMFKRAADRAAGALNVTPLNAEQL
EESRKSAIELETYFAKVLAERRKEPGDDLISQMILAEEDGQRLTDDEIVANLCFLFVAGH
ETTENMIGNTLIALQHHPEQRERVKADLSIMPKVVTEALRYDSSVQIAQRVALEEVELEG
QTIQRGDLICVFLGGGNRDPEKFENPNALDIDRENVRPLSFGGGLHYCLGARLALLEIAA
ALETVYTRLPNLHLTNLDALPYRRNNALRGVDSLLGTW

>bpln_2g27700|KEGG|CYP107DN1|[Burkholderia plantarii]bpla|BP

MSDPEIQQVSLYDLFQPRWRADPYPLYRRLRDESPVHADPNGNGWIVSRHADVVAVLDDA
RFSAARLGGIDDESRTEQAIRRTLSKQMLFLDGADHTRLRKLFAKAFTPRRLDTFRPRIE
ALAHRMLAAQAGRTIDFVSEFAVPLPVMVIALVLGVPMDAHAQLHDWSLAFGSVISGRVL
SDAETARAEAGIAAFGAYFAELIRERRREPGDDMLSDLLAAEEAGDRLDESELIANLILL
LAAGHATTTHLLGNGLLALSREREQWRLLADDPAIAPAAVNELLRFDAPVQLTGRAVLED
VELGGQRLPKGAYVYTLLGAANRDERHFAAPDTLDVRRPVSRVLSFGHGMHTCLGALLAR
IEAQIVFTSLAALYPGIEVDAQQARRTESLSFRGLRSLPVRLV

>AQ610_21835|KEGG|CYP1486A1|[Burkholderia sp.]bud|BP

MRDMSKVEYDAAKIRPFNIDPQVAAIIKNPERPEAFPPLAKVPKSIPGTSGFIAGMKSLF
AYKREGLQFFANQANIHGKVFRNQLGPYSIVGVVDHEVISQILKNGDRCWSTALAVERVT
RGLSFDIDLKKKSRLMSLDFDAHRQVRTLVQPAFSDRALASYLAIIQQEFTRDIEAWPRD
GEVPFRVEARQIFSQLAARLFFGIEDSSEAARLEGATRDYWNAIPVVLKNRHLNERWRRG
LDGVATLSSIMYDLIPKRRDEGGTDLFSQLCKSREDTGGLDDDQLVDLMLNIVFAAFDTT
TMGITSMAYLLAKHPHWQEVLRAESMRLPEQAALADFNGMERTTQVWKETLRMYPVSAIT
PRVSLGECQVGPYVVPPRTLVFNFTGLLGQDSDLWDHPQRFDPERFSPERAEYQRHRGQY
LPFGHGVHTCVGAALANLEAKVFWSIVLTRYKFSLAKDYDAVHCMNPLGSVSGEVALKVE
RL

>AQ610_19620|KEGG|CYP1488A2|[Burkholderia sp.]bud|BP

MNGLPSSLPRVGSTESLFAEPLAFLARARSQHGDVVVMRERGPIF
SRASDCSGVIAVFGEHRLRQILTEIDDFALPMSAAAKLALPKNLVNLNRGLHSMREPEH
GRHKRLLTGTINRELFDAHRNGIRAALDRLGERLKEDGRIAVVSRMRELTSEMASHVFL
GPRCHEEDAELAFLLSAYFTLRREASSLNARDPHVYRAELIGVGQQLDRTLRERIRRYR

NGPADDRAGLLRRLATAGLPGSPALSEDEIVGHANVMFVSSTEPVAISLTWLLLVLSQL
PGLRRALRAEIAGRASSPTSPGGAPLLENVVNETLRLLTPNALMVRATTRAVSLQGVAL
PAHCEIVVCPFLAHREAGPFPDPLAFSPSRWETARPSPYEYFPFGAGGHFCAGRNLALL
LIREVLSTLLLRFDFVLDGEQSIDWRIHIMLMPTGDPTLIAHPADERRDTPSPKWRGPA
TDLFHFAPGLS

>ALX46860.1|NCBI|CYP1013A3|[Burkholderia sp.]bud|BP

MSKYRQAPGPRGSLVMGNLAAYKRNPITMLLRLHQQYGDVARNRLGPFVTHAIAHPDHIQ
YVLQENHRNYVRGRFYDNFKMFFGDGLLTTDGEFWRRHRRAVQPLFHKKQVDAHTAAVGD
AALALAHRWSALPPGKAIDVVEEMMHLSLRMLGLMVFNTDVSSHAEAVGPAVRFGIEAMM
PQGDMNDFIPRWAPTRFNRRIAHARRAIDTIIAKIIADHREARCEPSDVISLLLNARDPD
TGAPMTQQEVHDEVMTVFLAGHETTGAGLAWALYALAQHPAVLRQLRDELDARLGGRAPT
VQDFEQLPYLSQVVDEVLRVYPPIWGFTRDLVEDDEIGGYRIPARSSVFMSPYVTHRHPA
FWRNPDAFDPENFASDAPARHRFVYFPFGGGMRKCIGFQTALLQMRVLVAVVAQHFDLNA
LPGHPIEFGATISLRPVHGIRLIVKPRERQQSHLARVREHESARALRPLGDTVAAAGAAC
PMAAALALDAAHGGGAAVAAAREAARQHEPGDVATLSLATSPATALPAAGTDTDASARSV
PAWRFTWRPVPVDPVPDEPSPALSGRRIALVNGRGGTVERVAAALARTCAKVSVFAPAAN
ADHAEAARVFVNEAGPLDGIVDLGLEAPFALDDASAWEAPMRRTVELLKACYGDWAAEDD

TSRLFYVALTWMDGQMGYGADAGAQPLGGLWAGLAKTLPQELPNCNVRVLDIAPDETGRV
DRLVVNELYRWGLFEVGYRNGVRYTLHAKRNELPPLAVDARSALGPGDAVLFSGGARGIG
LLCARALAGRHGCTVIVTGRETPPDGGEPWMTLDEEGFKRYGLEQLRAATPEHPPKAIRA
SLQRLKRRRELKTALDEMAALGLPVHYRVCDVTDRAAVRALCDELGDSLRIVIHNAGVDE
PARLIRKRADDFARTVRTKVLGFANLCDAVRARPRLAQFCNVGSLTGRWGGMTGETDYAA
ANEALARLGLWAAQHALSPACGVKTLVWPTWEGVGMITNFAVTQRYVSPMNVDEGVRHWL
RELASPGSGEVMFMGAVGRAPTPVQIKGFQPIRELPNIGELVTRRHHVGEPVRFRPFKRF
EGRYAIDPAVAPYAHAFRFNGRAVVPASLVLEHACAVGDWVMPPTGEPRELSEMANVVLR
LDALPAVPAGGAGVEIGSEAAGYMIGDVWCVDVRCSDARTRAELLSMTLVYRPQPEQIGA
AYARAPGATPEPAPLTSAPHAAWNGELLPAGDWSVPDDASAGIVRIGRVPPAAVLSLWTL
PLPLELRLPVNHVEHVLRVLLAEWGAAQTDAPAVWRIGGAKLARLPAASADGVVEYATGR
FAVLDARGYVLLRIDDTRVDGDAAQAMQTETGALAAPLTA

>DM992_22260|KEGG|CYP229F2|[Burkholderia sp.]burk|BP

MNPNPTDPIAAVTHRDPYPYYAALVDGPPLAFDAALGLWVASRAATVTAVLGHPACRVRP
LDAPVPPALRGTTAGALFGELVRMNDGALRHDVPKQALRTAFAPVDTARLRARAAQLAAR
RLPAPGDADALNAWCMTVPVCAVADLLGFDETQLDDIAARVVDFVAALSPLSDAAALARA
SDGARQLLDRMSERVAQTHAHDGTLVAAVQQAARASGWQASGALVANLVGLLSQTCEATA
AWLGNTLAAWNGEAEAARNAPDDTVLDAFVAEVGRFDAPVQNTRRFVASRTTIEGVTVEA
GDAILVVLAAANRDPAVHRDPHRLMPGRAAGPNFGFGTGLHGCPGERIARAVTAGAFAAW
LRAGGWPPRGGLTWNYRASTNVRMPKFDAAR

>Bxe_A3593|KEGG|CYP153A10|[Paraburkholderia xenovorans]bxe|BP

MNTLVVDSSHVRLAPDALSQPVEDIDPSLPYRFQQQTHFAMFDRLRRESPVHYVKDSEYG
PFWSVHRYNDIIDVEIDHATFSSDVKYGGMLIKDLPENMRRTSFINADPPLHDHQRRVVS
PIVAPGNLNRLEHTIRREAADILDGLPRGETFDWVDNVSIELTGRVLCELMDFPRADRRL
LTYWSDIVNVDLEVGGEINTEEKRYVKLKECASYFGVLFKERMNSEPKDDLISMLAHSEY
TKNMPEQEFLGMIVLLMVGGNDTTRNSISGGLVALNQFPEQYAKLHNDPGLIPKLVPEIL
RWVTPVTHMRRTATRDIEFRGKQIRQGDKVVVWYASGNRDSDVIKDPYQFIIDRANPRLH
LSFGFGIHRCLGNRLAELQLRVLWEEILKRQMLIEMMGEPVRKYANNITGVMALPVRIAA

>Bxe_A2330|KEGG|CYP195A3|[Paraburkholderia xenovorans]bxe|BP

MTPATASDASTLARDFDLRHLNPAFHADPYPVYHALRAHEPVKRMPDGSLFLTRFRDVQA
VYRDPKTFSSDKTVEFKPKYGDSPLYAHHTTSLVFNDPPRHTRVRKLIAGALTARAIAAM
EPGLVRLVDGLLDAAAARGRIDLIDEFASAIPVEVIGNLLDVPHTERAPLRDWSLAILGA
LEPSLSEAQLERGNRAVSEFIDYLRDLVARRRREPGDPQHDVLTRLIQGEAGGEQLSEAE
LLQNCIFILNAGHETTTNLIGNGLVTLTQWPEQRAALLHEPSLIESAVEECLRFESSNQL
GNRMATVDTEIGGVAVARGTPVTLCIGAANRDPEQFADPDRFDIRRDPNRHLAFGFGIHQ
CAGLSLARLEARIAIGRFVQRFPAYRVNGEPTRGGRVRFRGFAAVPVELEPAGRRTA

>Bxe_C0290|KEGG|CYP220A1|[Paraburkholderia xenovorans]bxe|BP

MTFIYDPNDPDVRRDPHAVFRKLRETEPVHWSPKLSGWVVTSYELASEVLTTNGTYSAER
FTAVQQHLSEEKRVTAAEVMRWFQHWMVFRDPPDHTRLRRHMANTLNIPVFDARRETVIS
VVNELLDRIPVGDAFDFFQAFSLWMPGIVVADLLGVERDRLLEVKQWSDDMMTFIGSARG
VPDKYERARRGANGMGTYFLDLIAKRRAEPREDALSRLIASEVEGQRLSDDELVGCMMMV
LNGGHETTANLLNNSMLALAAHPQTVQHLRQHPDEMAAAVEEFLRYDSPVLSIGRIVTED
TELGDQEIAAGDRVFAMLVGANRDPEVFSDPDELRTSRNPNPHMAFGKGPHFCLGTPLAR
LEGQIALTAILERFSSIELCEPVESIPWLNSLVTHGPTRLPLRLK

>Bxe_C0599|KEGG|CYP226A1|[Paraburkholderia xenovorans]bxe|BP

MTSPAKSNALESAFESTASNYRGSDVDLNAIYRDMRRNSPVIAQDFMASLGVPNIAKLDP
NRPTFTLFKYKDVMSVLRDAANFTSGFIAEGLGSFFDGLILTGLDGEAHRRARALLQPVF
LPEVVNRWRESKMEPIVRNEFIGPMVPQRRADLMHFGLHFPIRLIYSLIGFADDRPEQVE
QYAAWALAILAGPQVDAQKAAIARKAAMEAAEALYAAIRSEVAVVRAKGAEGEDLISRLI
RAEYEGRRLDDHDIATFVRSLLPAAGETTTRTFGSLMTLLLERPALLERVKADRSLVSKA
IDEAVRFEPVATFKVRQAAVDTGIGGVSIPKGAMVQCIVSSANRDEEVFENSETFDIDRK
PKPSFGFGFGPHMCIGQFIAKVELQVAVNAILDLLPNLRLDPDRSPPKIVGAQLRGPDAV
HVVWD

>Bxe_C0631|KEGG|CYP226A2|[Paraburkholderia xenovorans]bxe|BP

MSTTLENPMLDLEAAYHAVSDTYRGPDIDLQALCREMRHKNPVMKGDFVATHLGIPTNAG
ASAAKCEVTLFRYQDVLAVMRDATTFTNGFIAEGLGGFFDGLIILAMDGDAHRRARGLLQ
PVFMPETVNRWRPELDRVIREDFLAPLVPNRHADLMDFGLYFPIREMYALMGFPTDDTAK
FNQYATWALAMVAGNQIDPGKIRIFGPIAAAAVKHLYDAVMEVVLQRRAAGADGNDLISR

LMRAEYEGHKLDDHEVTTFVRSLLPAAGETTTRTFSSVITLLLERPALVERVRNDRSLIP
RLIDEAVRYEPVATFKVRQAARDIEIGGVKVRSGGLVQCMVMSANRDEDVFENADTFDID
RKPKPSFGFGFGAHMCIGQFVAKIELQCAVNAILDLFPNVRLDPARPAPKIAGAQLRGAK
SVPVIWD

>Bxe_C0945|KEGG|CYP105T1|[Paraburkholderia xenovorans]bxe|BP

MTSAINDVRPQTTSTFPFARTGSPLHPPAEYARYRDGQPVTRVQMWDGRYAWIFTRMEDV
KAVLSSPHFSVVPSKPGYPFLTPARAATVKSYQTFITMDPPDHTRFRRMLTRDFTQKRME
ELRPQIAAYVNRLIDEMLARGSPGDLVSALALKLPVTVVSMLVGVPYEDHEDLVKWSGQR
LDLEQNPTVSESAADNMLAYFDGLLQRKERDPGDGADMLSRLVIEQIKPGHLSRLEAIHM
VNLLYFAGHETTANQIALGTLSFLLDPRQRALLENNPGLLKNAIEEMLRFHTISHYNSCR
VATADVEVGGTLIREGEGAYALIMAANRDPAAFPAPDRFDIERPNSQEHVAFSYGLHMCL
GQPLARLELQVCFEALFRRLPRLRLAVPLEELPFKREMYVYGLHALPVTW

>DR64_1290|KEGG|CYP153A10|[Paraburkholderia xenovorans]bxb|BP

MNTLVVDSSHVRLAPDALSQPVEDIDPSLPYRFQQQTHFAMFDRLRRESPVHYVKDSEYG
PFWSVHRYNDIIDVEIDHATFSSDVKYGGMLIKDLPENMRRTSFINADPPLHDHQRRVVS
PIVAPGNLNRLEHTIRREAADILDGLPRGETFDWVDNVSIELTGRVLCELMDFPRADRRL
LTYWSDIVNVDLEVGGEINTEEKRYVKLKECASYFGVLFKERMNSEPKDDLISMLAHSEY
TKNMPEQEFLGMIVLLMVGGNDTTRNSISGGLVALNQFPEQYAKLHNDPGLIPKLVPEIL
RWVTPVTHMRRTATRDIEFRGKQIRQGDKVVVWYASGNRDSDVIKDPYQFIIDRANPRLH
LSFGFGIHRCLGNRLAELQLRVLWEEILKRQMLIEMMGEPVRKYANNITGVMALPVRIAA

>DR64_7505|KEGG|CYP105T1|[Paraburkholderia xenovorans]bxb|BP

MTSAINDVRPQTTSTFPFARTGSPLHPPAEYARYRDGQPVTRVQMWDGRYAWIFTRMEDV
KAVLSSPHFSVVPSKPGYPFLTPARAATVKSYQTFITMDPPDHTRFRRMLTRDFTQKRME
ELRPQIAAYVNRLIDEMLARGSPGDLVSALALKLPVTVVSMLVGVPYEDHEDLVKWSGQR
LDLEQNPTVSESAADNMLAYFDGLLQRKERDPGDGADMLSRLVIEQIKPGHLSRLEAIHM
VNLLYFAGHETTANQIALGTLSFLLDPRQRALLENNPGLLKNAIEEMLRFHTISHYNSCR
VATADVEVGGTLIREGEGAYALIMAANRDPAAFPAPDRFDIERPNSQEHVAFSYGLHMCL
GQPLARLELQVCFEALFRRLPRLRLAVPLEELPFKREMYVYGLHALPVTW

>DR64_7815|KEGG|CYP226A2|[Paraburkholderia xenovorans]bxb|BP

MSTTLENPMLDLEAAYHAVSDTYRGPDIDLQALCREMRHKNPVMKGDFVATHLGIPTNAG
ASAAKCEVTLFRYQDVLAVMRDATTFTNGFIAEGLGGFFDGLIIILAMDGDAHRRARGLLQ
PVFMPETVNRWRPELDRVIREDFLAPLVPNRHADLMDFGLYFPIREMYALMGFPTDDTAK
FNQYATWALAMVAGNQIDPGKIRIFGPIAAAAVKHLYDAVMEVVLQRRAAGADGNDLISR
LMRAEYEGHKLDDHEVTTFVRSLLPAAGETTTRTFSSVITLLLERPALVERVRNDRSLIP
RLIDEAVRYEPVATFKVRQAARDIEIGGVKVRSGGLVQCMVMSANRDEDVFENADTFDID
RKPKPSFGFGFGAHMCIGQFVAKIELQCAVNAILDLFPNVRLDPARPAPKIAGAQLRGAK
SVPVIWD

>DR64_7847|KEGG|CYP226A1|[Paraburkholderia xenovorans]bxb|BP
MTSPAKSNALESAFESTASNYRGSDVDLNAIYRDMRRNSPVIAQDFMASLGVPNIAKLDP
NRPTFTLFKYKDVMSVLRDAANFTSGFIAEGLGSFFDGLILTGLDGEAHRRARALLQPVF
LPEVVNRWRESKMEPIVRNEFIGPMVPQRRADLMHFGLHFPIRLIYSLIGFADDRPEQVE
QYAAWALAILAGPQVDAQKAAIARKAAMEAAEALYAAIRSEVAVVRAKGAEGEDLISRLI
RAEYEGRRLDDHDIATFVRSLLPAAGETTTRTFGSLMTLLLERPALLERVKADRSLVSKA
IDEAVRFEPVATFKVRQAAVDTGIGGVSIPKGAMVQCIVSSANRDEEVFENSETFDIDRK
PKPSFGFGFGPHMCIGQFIAKVELQVAVNAILDLLPNLRLDPDRSPPKIVGAQLRGPDAV
HVVWD

>DR64_8143|KEGG|CYP220A1|[Paraburkholderia xenovorans]bxb|BP
MTFIYDPNDPDVRRDPHAVFRKLRETEPVHWSPKLSGWVVTSYELASEVLTTNGTYSAER
FTAVQQHLSEEKRVTAAEVMRWFQHWMVFRDPPDHTRLRRHMANTLNIPVFDARRETVIS
VVNELLDRIPVGDAFDFFQAFSLWMPGIVVADLLGVERDRLLEVKQWSDDMMTFIGSARG

VPDKYERARRGANGMGTYFLDLIAKRRAEPREDALSRLIASEVEGQRLSDDELVGCMMMV
LNGGHETTANLLNNSMLALAAHPQTVQHLRQHPDEMAAAVEEFLRYDSPVLSIGRIVTED
TELGDQEIAAGDRVFAMLVGANRDPEVFSDPDELRTSRNPNPHMAFGKGPHFCLGTPLAR
LEGQIALTAILERFSSIELCEPVESIPWLNSLVTHGPTRLPLRLK

>Bphy_0979|KEGG|CYP195A6|[Paraburkholderia phymatum]bph|BP

MNPSREAGIARAFDLQHLDASFHADPYPFYHALRTHEPVKRMPDGSLFLTRYRDVQAVYR
DPKTFSSDKKVEFAPKYGATPLFEHHTTSLVFNDPPLHTRVRKLIAGALTARAIAAMEDG
LVRLVDGLLDSAAARGEIDLIGDFAAAIPVEVIGNLLDVPHDERAPLRAWSLAILGALEP
ALTPAQHERGNRAVTEFVAYLEDLVARRRAAPGDPQHDVLTRLIVGEAGGEQLSEVELLQ
NCIFILNAGHETTTNLIGNGLVTLSSWTEERAALLREPELIESAVEECLRFESSNQLGNR
MTMRDTEIGGVPVTAGTPVTLCIGAANRDPEQFAEPDRFDIRRTPNRHLAFGFGIHQCAG
LSLARLEARIAIGRFVRRFPSYRLSGAPTRGGRVRFRGFAAVPCAVE

>Bphy_5447|KEGG|CYP133C2|[Paraburkholderia phymatum]bph|BP

MKFTDLSSPAYFDNPYPFYDGIRSAGAFVPLAPGIFLTGRHAVIDALLPDRRMGKTYMPS
VVVRYGETAREQPVFQALERTFLMMNPPAHTRLRALLMKAFNARQIETLRTIAEQTTDSL
IDAMQGKREVDLVSGFAMPLPLQIICRLLDVPVEEGERFGEAASLLVSAFDLAPLSPEAL
ARANQAALDLEQYFRAVIAERRAAPGHDIVSALIHAEEGGERLSEDEVVSNVILLFVAGH
ETTSNMLGNALIALHRHPSQLERLTSNLSLVPQAVAECARFDTAVQMVVRTAFDDIDVGG
QTLPRSSIVFMLLGSANRDPERFDAPDTLDIGREASGHLLTFGAGIHYCLGARLAMMELE
IALRKLMTRVPQLRLTNLDALQWRRRNNLRGVETLIAAL

>Bphy_6307|KEGG|CYP107DQ1|[Paraburkholderia phymatum]bph|BP

MYNVADSSRSASPTLPAAGFSPSYDPLIPHPEHVKDPYRVYAELRSNAPLYRSPHGVWIA
SGYEEVSTMLKSPDFGRGYFYFDNMAKRLGEAMVSQPVYASAREMMVMKDGPDHLRLRGL
VAHFFSHRNVEALRPFMRSVMEGLIDKALEKSSFDVMTDIAFPLPSAVICRLVGLPEADW
PRFRQRSTNGSRALEPAPLSPLELAEQNHAVEEARDYFSWLIELRKREPGDDLATLLVNA
EAAGQASREEVIENLRMMFVGGQETTVNTIGNGLLALHRHPDQLQALKSDRALIPDAVTE
IVRYDSAVQITPRQARCDMEMCGAAITAGDTILCVVASANRDERAWVQADRFDISRKRNY

PLSFGGGPHYCLGAQLGMVETEVALAALLDRMPNLALDIADPRWLPGTVVFRGLQSLPAH
A

>Bphy_7766|KEGG|CYP108S3|[Paraburkholderia phymatum]bph|BP

MNMSNYDLPKDMANHLVDPHYYTNPDRLHETYAWARANNPVGRAMADGHDPFWVVTKHAD
VSMISRDTVSFCNGEYSVVCRPKASIEHVISATGSPNIVRSLVHVDGDEHKNLRALTQSW
FMPNSIIKLDGRIRSLARAMTAKLLQQSGQVIDFANDVASHYPLNVIMDILGVPQEDSLR
LLTLTHEMFGSEDAEYKREMQRQGDSSSAMGKALLSVVADFKDYFKRLTEDRRANARNDI
ASVLANAVVNGQPIDERTLLGYYVIIAAAGHDTVSSSISVAMWALSRFPELLPRLQADRS
LIPQFIDETCRYASPVRHFMRTATVDTDVRGRTIRKGDWLMLCYGSANFDEEVFPEPFNF
NIDRKPNRHLAFGVGAHVCLGQHLANMEMRILFEELIPRLRLIEPAGEIEFIASSFVGGP
KHLPVRCVMQ

>BC1002_1423|KEGG|CYP195A3|[Paraburkholderia atlantica]bge|BP

MTPPTASDASSLARDFDLRDLSPAFYADPYPIYRALRTYEPVRRMPDGSLFLTRFRDVQA
VYRDPQIFSSDKTVEFRPKYGDSPLYAHHTTSLVFNDPPRHTRVRKLIAGALTARAIAAM
EPALIRLVDGLLDAAAQRGRIDLIGEFASAIPVEVIGNLLDVPHDERAPLRDWSLAILGA
LEPSLTDAQFERGNRAVSEFVDYLRGLVVTRRREPGDPQHDVLTRLIQGEGPDEQLSEAE
LLQNCIFILNAGHETTTNLIGNGLVTLSQWPDERAALLREPELIGTAVEECLRFESSNQL
GNRMTTVDTEIGGVAVARGTPVTLCIGAANRDPEQFAEPDRFDIRRDPNRHLAFGFGIHQ
CAGLSLARLEARIAIGRFVQRFPAYRVDGEPTRGGRVRFRGFASVPILLEP

>BC1002_6864|KEGG|CYP108S5|[Paraburkholderia atlantica]bge|BP

MSMTNYDLPKDIANRLVDPQSYTNLDGLHEIYAWARANNPVGRAMADAHDPFWVVTKHAD
VSMISRNNALFRNGDYSVVCRPQASIAHVIAATGSPNIVRSLVHVDGDEHKRLRALTQSW
FMPNSVVILEARIRSLAHLMIGELVQQSSSVIDFANDFALHYPLNVIMDILGVPQEESPR
MLRLTQEMFGSEDAEYKREISRQGGAGSAIGKALLSVVADFKGYFDRLTEDRRKNPRNDI
ATLLANAVVDGQPIDEATRLGYYVILAAAGHDTVSSSISVAMWALCQFPHLLPRLKADPS
LIPQFIDESLRYASPVRHFMRTAAAETNLRGRTIRKGDWLMLCYGSANRDEEVFPDPFVF

DIDRRSNRHIAFGAGAHVCLGQHLANMEMRILFEELIPKLQFVEPVGDVEFTTSNFVSGP
KHLPIRCVLQ

>BUPH_02254|KEGG|CYP195A3|[Paraburkholderia phenoliruptrix]bpx|BP

MPTATAGDPAALARDFDLRRLSPDFYADPYPVYHALRAHEPVKRMPDGSLFLTRFRDVQA
VYRDPKTFSSDKTVEFRPKYGDSPLYTHHTTSLVFNDPPRHTRVRKLIAGALTARAIAAM
EPGLVRLVDGLLDAAAERGRIDLIGDFASAIPVEIIGNLLDVPHAERAPLRDWSLAILGA
LEPSLTDAQFERGNRAVSEFVDYLRDLVARRRAPGDPQHDVLTRLIQGEAGGEQLSEEE
LLQNCIFILNAGHETTTNLIGNGLVTLTECPEQRAALLAEPALIESAVEECLRFESSNQL
GNRMATVDAEIGGLPVARGTPVTLCIGAANRDPEQFPDPDRFDIRREPNRHLAFGFGIHQ
CAGLSLARLEARIAIGRFVQRFPSYRLDGEPTRGGRVRFRGFAEVPLLCR

>BUPH_00139|KEGG|CYP1485A2|[Paraburkholderia phenoliruptrix]bpx|BP

MHTYSAMTCSMPEPESFSVLPSIIGTGSADPYPLYEWLRAHRPLCRDASGVWLASSHALV
TRILDDARFSARPPPGAVPLDDPLGYTNMVVFQTGEAHERLRRFLAPLFSRKALLSLQAF
IDDEVARLIDQCERLPRFDLVAHIASVLPVRTICRLLGFPDEHGASCLQASAGAWQLISG
APMSAEERALAAVQTETFIAQVESFMLAIEPDVVPDHAAARFLRLEQEGGIGRRDMLVNV
LFLFIAGYGTTLLSIGNSIAALLRAPRAWQALKDDVSLVAQAVRELYRYDPAVQSVFRYA
WDDVEIDGCRIRRGERVMPLIGAANRDPAAFAAADEIRFEREAGRSLTFGAGPHSCMGLA
LARMQLESLLSALMRRMPGLRLAPGEQPRQQHGSFHGYDRLMVEH

>BUPH_08319|KEGG|CYP108S4|[Paraburkholderia phenoliruptrix]bpx|BP

MNMSNYDLPKDMANHLVDPHYYTNPDRLHETYAWARANNPVGRAMADGHDPFWVVTKHAD
VSMISRDTVSFCNGEYSVVCRPKASIEHVISATGSPNIVRSLVHVDGDEHKNLRALTQSW
FMPNSIIKLDGRIRSLARAMTAKLLQQSGQVIDFANDVASHYPLNVIMDILGVPQEDSLR
LLTLTHEMFGSEDAEYKREMQRQGDSSSAMGKALLSVVADFKDYFKRLTEDRRANARNDI

ASVLANAVVNGQPIDERTLLGYYVIIAAAGHDTVSSSISVAMWALSRFPELLPRLQADRS
LIPQFIDETCRYASPVRHFMRTATVDTDVRGRTIRKGDWLMLCYGSANFDEEVFPEPFNF
NIDRKPNRHLAFGVGAHVCLGQYLANMEMRILFEELIPRLRLIEPAGEIEFIASSFVGGP
KHLPVRCVMQ

>Bphyt_1856|KEGG|CYP195A3|[Paraburkholderia phytofirmans]bpy|BP

MPPATASDASTLARDFDLRHLDPAFHADPYPVYHALRAYEPVKRMPDGSLFLTRFRDVQA
VYRDAKTFSSDKTVEFKPKYGDSPLYAHHTTSLVFNDPPRHTRVRKLIAGALTARAIAAM
EPGLVRLVDGLLDAAAERGRIDLIGEFASAIPVEIIGNLLDVPHAEREPLRDWSLAILGA
LEPSLTEAQFERGNRAVDEFVGYLRDLVARRRRAPGDPQHDVLTRLIQGEEGGEQLSEAE
LLQNCIFILNAGHETTTNLIGNGLVTLTQWPEQRAALLHEPSLIESAVEECLRFESSNQL
GNRMATVGTEIGGVAVARGTPVTLCIGAANRDPEQFAEPDRFDIRRDPNRHLAFGFGIHQ
CAGLSLARLEARIAIGRFVQRFPRYRLDGEPTRGGRVRFRGFAEVPLELEPVASRTA

>Bphyt_2741|KEGG|CYP1258B1|[Paraburkholderia phytofirmans]bpy|BP

MNASLQAVRSGSPDTVVIARTKELADDGIKAANIDGIDLIVLRHGNAISIFQGRCPHQGT
LLSEGTVKDGVLACRAHGWQFDCSSGCKQGPSSSNLKRFDAIVENGEVKVDRNEVRAWNA
QRPAPAVIMPCRESAVRSLAQLPGPKRVSFLGNTLQLRPEKLHLVLEEWCREFGPVYTFR
LIGRPFVAIADPILISQVLRDRPGAYRRWNVIETVARELGINGVFSMEGDAWRRQRQLVA
KALDPLHLRAFFPLLCEMTRRLLQRWEKAERERQIIDIQKDLMRYTVDVTTNLAFGYDMD
TIESEGDVIQRHLEVVFPMINRRINAPFPYWHYVKLAADKTLDRALIAVQALTKDLIAAN
RARLAQRPETDTMPTNLLEALLMAQHDGEAPLTDDEVLANVFTILLAGQDTTANTIAWVA
YFIASLPDVQRKMQEEVDAVIGSAAMLEDFDAGDALLYVNAVISESMRLKSVTPVLAVEP
NYDVQVGDIAIPKGAVLSLLTREAGLQQGGHSAPDVFDPARWLTGESAASHHRAGFMPFG
SGPRLCPGRSLALMEVRSAVAMLCRNFDLLLVDNASEVEEVFAFSMVPRNLRIELRRRG

>OI25_3427|KEGG|CYP195A3|[Paraburkholderia fungorum]bfn|BP
MPPATASDASSLARDFDLRHLSPSFHADPYPVYHALRTYEPVKRMPDGSLFLTRFRDVQA
VYRDPKTFSSDKTVEFKPKYGDSPLYAHHTTSLVFNDPPRHTRVRKLIAGALTARAIAAM
EPGLIRLVDGLLDAAAARGRIDLIGEFASAIPVEVIGNLLDVPHAEREPLRDWSLAILGA

LEPSLSEAHLERGNRAVSEFIDYLRDLVARRRREPGDPQHDVLTRLIQGEQGGEQLSEAE
LLQNCIFILNAGHETTTNLIGNGLVTLTEWPEQRAALQHEPSLVESAVEECLRFESSNQL
GNRMATVDTEIGGLAVPRGTPVTLCIGAANRDPEQFAEPDRFDIRRDPNRHLAFGFGIHQ
CAGLSLARLEARIAIGRFVQRFPAYRLDGEPTRGGRVRFRGFAAVPVELEPTSGG
>OI25_3857|KEGG|CYP226A18|[Paraburkholderia fungorum]bfn|BP

MSTPAATTSLESAFAGVADNYKGSDVDLHAIYRDMRRNSPVIAEDFMARLGVPNIAGLDA
NRPTFTLFKYKDVMSVLRDATHFTSGFIAEGLGAFFDGLILTGMDGEAHRRARSLLQPVF
MPDVVNRWRETKMAPIVRSEYIEPMVPKRRADLMDFGLHFPIRLIYSLIGFPDNRPEQIE
QYAAWALAILAGPQVDAEKAAQARKAAMEAAQALYDAVKLEVIEVRKNGAQGDDLISRLI
RAEYEGRSLDDHEVATFVRSLLPAAGETTTRTFGSLMVALLERPELLERVRADRSLVPKA
IDEAVRFEPVATFKVRQAAQDTEIGGFRIPKGAMVQCIVSSANRDEEVFENSESFDIDRK
LKPSFGFGFGPHMCIGQFIAKVELAVAVNTILDLLPNLRLDPEMPRPRIVGAQLRGPHAL
HVIWD

>OI25_3864|KEGG|CYP226A17|[Paraburkholderia fungorum]bfn|BP

MNAPTQEGLTLDLEAAYHAVSDTFRGPDLDLQALCREMRHTQPVMQGDFVATRLGVPTNA
GAKAAQCAVTLFKYQDVMAVMRDATTFTSGFIAEGLGAFFDGLIVLAMDGDAHRRARGLL
QPVFMPETVNRWRPELDRVIRQDFLEPLVPNKQAELMDFGLYFPIREMYALMGFPTDDTA
RFNQYATWALSMVAGNQIDPDKIRIYGPLAAAAVKHLYDAVKEVVVQRRAEGAHGDDLIS
RLMRAEYEGHMLDDHEVTTFVRSLLPAAGETTTRTFSSVMALLLERPELVERVRNDRTLI
PRLIDEAVRFEPVATFKVRQAARDVEIGGVKVPKGGLVQCMVISANRDEDAFDNPDIFDI
DRKPKPSFGFGFGTHMCIGQFVAKVELQCAVNAILDLFPNVRLDPGKPAPKIAGAQLRGA
KSVPVVWD

>OI25_5065|KEGG|CYP194C1|[Paraburkholderia fungorum]bfn|BP
MSDTTNAGNVKDGYDIFAGDYANDPDALWAAMRKGGCPVAHSDKWGGSWVVTRYDDVRDI
AHDGARFSSRAVEVAGPIPEIGHELFLPPLTSSPDDHKAHRDLLAPFFTPAKIAELEPFV
RDEARRLATAIAERGEGDAVGDFARPLALSVISHILKVPLDAQNRFVDWVVRLVRLGPLD
QKVRSAVVQEKLAYLEKLLEERASAPGDDLISYLAHATLNGEPLSRKHKLGSLFLLVLAG
ADTTWSAMGASLWHLASHADDRAQLLAQPDMMRTTAVEELLRAYAPVSIARLTSEPVELH
GRCIAAQERVILPLAAANRDPAVFDEPDTVKLDRKRNRHLTFSSGEHRCLGSNVARLELR

VALEEWLRVMPNFHLTAPDAVEWTAGQTRGPEQVLIAVDR

>OI25_5996|KEGG|CYP1155A1|[Paraburkholderia fungorum]bfn|BP

MPPLREFLRLAHATGTENEGRSAWQTGSDRYVPMLQILLGFLRRYWPIPRLFGWAAVTLS
DDVEEVLSLPNVFSTPFAEEMARLNDGARPGTQFLAMDDAHAHDAQVRQVMDAFRREDVK
ERVGDVANRKAAAIVAAANGRLEAIGGLITQVPLDICVDYYGIAIDDPKRFTEAFMVVSG
HLFGPPPVVQNDAVDEAAAYVRQVVSQAFEAERQQPRGTHTVIGRLLGTVKGGGITYLEA
HAFLSGMILGFVPTDTMAGGHILEMLLRRPGFLAASQAAARAGDDVLLGRCLFEALRFMP
INPGPFRVCAADYIVAAGTSRETRIRKGTKVLACTMSAMFDSRKVNNPFNFVPGRPASDL
MNFGFGMHWCVGAFIAQTQIVQTFKPLLLRSGLRRAPGPDGQLKLRHTFPASLVVEFEVA
S

>OI25_7436|KEGG|CYP289A14|[Paraburkholderia fungorum]bfn|BP

MNEKPINDWDPRSEDVTRDQAAAYDDMRHRCPVAHSDYLNLSLFRHDDIMRVLNDPETFS
SAVSSYLSVPNGMDPPEHTRYRSIIEPYFSPRRMMIFEPVCREIAGSLANGLSRGGEIEF
NARFAQDFAMNIQCAFLGWPKELRTPLLHWIRKNREATLTGDRIALDRVAVEFDTYIKGL
LVQRRKSGANAPDDITTSLLRERIGDRLLHDDEIVSVLRNWTVGELGTIAACVGILAHYL
AEHPDIQRRLRAQPSLLPEAIDEILRIRAPLIANRRISTRPAEIGGRAVPAGQRITLIWA
SANRDETVFGEPDEFRLDRDPANNLLYGSGIHDCPGAPLAQLELRIGIEELLASTHWIAL
VPGCPPRTPSIQPRGFLHCHCTSSESPIARPRTCKVPSCL

>K788_0007982|KEGG|CYP195A6|[Paraburkholderia caribensis]bcai|BP

MAGVTRIDVMLATTCKHHSAGMVHTEGSKRFNRGSTAVSAALAAKIKKETPMAPSNATSN
DADIARRFDLRHLDASFHADPYPVYHALRTHEPVKRMPDGSLFLTRYRDVQAVYRDPKTF
SSDKKVEFAPKYGATPLFEHHTTSLVFNDPPLHTRVRKLIAGALTARAIAAMEDGLVRLV

DGLLDRAAGRGEIDLIGDFAAAIPVEVIGNLLDVPHDEREPLRDWSLAILGALEPSLTPA
QHERGNRAVTEFVAYLKDLVARRRAAPGDPQHDVLTRLIQGEQGEGGGEQLSEVELLQNC
IFILNAGHETTTNLIGNGLVTLSVWTEERDALLREPALIESAVEECLRFESSNQLGNRMT
MIDTEIGGVPVAAGTPVTLCIGAANRDPEQFAQPDRFDIRRAPNRHLAFGFGIHQCAGLS
LARLEARIAIGRFVRRFPAYRISGEPTRGGRVRFRGYAAVPCAVE

>K788_0003175|KEGG|CYP133C2|[Paraburkholderia caribensis]bcai|BP

MKFTDLSSPAYFDNPYPFYEGIRSAGAFVPLAPSIMLTGRHAVIDALLPDRRMGKTYMPS
VVARYGESAREQPVFQALERTFLMMNPPTHTRLRALLMKAFNARQIEMLRTIAEETTDSL
IDAMQGKREVDLVSEFAMPLPLQIICRLLDVPVEDGVRFGDAASLLVSAFDLAPMSADAL
ARANQAALDLEQYFRGVIAQRRATPGHDIVSSLIQAEEGGERLSEDEIVSNVILLFVAGH
ETTSNMLGNALIALHRHPAQLERLRGDPSLVSKAVAECARYDTAVQMVVRTAFDDIEVEG
QTLPRGSIVFMLLGSANRDPERFDAPDTLDIGREPSGHLLTFGAGIHYCLGARLAMMELE
IALHKLLTRLPQLRLTNLDALQWRRRNNLRGVETLLAAL

>K788_0004290|KEGG|CYP1049A4|[Paraburkholderia caribensis]bcai|BP

MRNVHQGMMSTWLCDTAKAGDPIEFRGPMGSFYLRPIERPVLFLAGGTGLAPFLSMLDKI
VEDGGSPYPIHMILGVNSDADLVGIDRLEIYAQRLPNFTYACTVASPDSAYPNKGYVTHH
ISASRLNGGDADVYLCGPPPMVDAVRSFLSSEGLTPRNFYYEKFAGTGLVVQTGEERIAP
VDVDEAFDLRMVLELGAAQLTMGRLSSEQLIIFRQLAEATAQFVSGRRFTDVPRYIEANH
AFHKFTIEASGNAQLIALYRQLAVQDYIARALTGQVEIVGDIVQQHRDIVAAFELGDLNA
ARDVIALHALHSRATMSRALERMAMGKTAPMVAAPPSLPSAVPESAPQVCPFTAKTLPSY
SHELDWPEGLEPFKVVDDGSQGDPYAHYRWMREHAPVLRCQSATSDVWFLSRYDDVYQAI
RNPKLFSSEVVSPPPLTFLTLFDAPDHSRLRKVVQPSFLPLALDPFIGQIAQRAEDLLDA
MIANGGGDVVNDFAIPLSISTISTMIEVPNEDEEKMKFWSDETFSYFGRLARNAPGTGTD
EQSAQAFFAYLKQALERLALTDSQSIGGHIARMWKDGLLSEKEAKELCAFVFIAGHDTTT
ILVANAFRMFAEQPELLGRIRENPADADRFVEEVARYRGTVQRVSRITTEATTVAGVDLP
KGAVVRLLLSAANRDSRKFAEGETFDIDRDSSGHLGFGNGMHKCLGAPLAKLETLIATKV
LARKVGNIAFDPARPIQYVRGNNLTNSGPEHMFVKIGGLSA

>BJG93_05715|KEGG|CYP195A3|[Paraburkholderia sprentiae]pspw|BP

MTLASASDASSLARDFDLRDPDPAFYADPYPVYHALRTYEPIRRMPDGSIFLTRFRDVQA
VYRDPNTFSSDKTVEFRPKYGDSPLYAHHTTSLVFNDPPRHTRVRKLIAGALTARAIAAM
EPALIRLVDSLLDAAAQRGRIDLIGEFAAAIPLEVIGNLLDVPHGERAPLRNWSLAILGA
LEPSLTGAQFERGNRAVSEFVDYLRGLVARRRCEPGDPQHDVLTRLIQGEGPDEQLSEVE
LLQNCIFILNAGHETTTNLIGNGLVTLSEWAEERAALRREPALIASAVEECLRFESSNQL
GNRMTTVDTEIGGVALARGTPVTLCIGAANRDPEQFAQPDRFHIRRDPNRHLAFGFGIHQ
CAGLSLARLEARIAIGRFVQRFPAYRLDGEPTRGGRVRFRGFASVPVLLAP

>BJG93_16600|KEGG|CYP108S7|[Paraburkholderia sprentiae]pspw|BP

MSMPNYDLPKDIANHLVDPQSYTNLNRLHETYTWARANNPVGRAMADGHDRFWVVTKHAD
VSMISRENVLFHNGDYSVVCRPRASIEHVISTTGSPNIVRSLVHVDGDEHKSLRALTQSW
LMPNSILELDDRIRSLANMTIAKLLLHSGQVIDFANDFALHFPLSVMMDILGVPQQDSPR
MLTLTQEMFGSEDAEYKREVQRQGDASSAMGKALLSIVADFKDYFDRLTEDRRKNPRNDI
ASLLANAVINGQPIDEATRLGYYVIIAAAGHDTVSSSISVAMWALSRFPELLPRLQADCS
LIPQFIDETLRYASPVRHFMRTATADTNVRGRTIRKGDWLMLCYGSANRDEQVFPDPFAF
NIDRKPNRHLAFGVGAHVCLGQHLANMEMRIFFEELVPRLRLIEPAGEIEFVASSFVSGP
KHLPVRCVVHPH

>BJG93_29335|KEGG|CYP101F1|[Paraburkholderia sprentiae]pspw|BP
MSPTKDWTVPDHVPRELVVDFDYMNPPGVQDDVHIAWKTLHTGPDIVWSPYHGGHWIATR
GEDIEAMQKDHTRFSHRSVNIPPLNETRLVPLELDPPEHTAYRNLITPAFLPQAIASLEN
DVRNLAIELIDTLAARGECEFVSEFSKILPIVIFLRLADLPLSDREQLLEWAEWAVRGNV
EERNESQHRLVGYISTWVEKRQAEAGGDLVSLVANAQIEGKPISPERMFGMFIVVLFGGL
DTVASMMGFIARFLAENPAHRRQLIENPDLMISSVDELIRRFGVANTARLVTQDMEYKGI
QFRKGDQIQLPNSLFGLDDRKFNDPLAVDLTRKPVIHAAFGNGPHRCPGSFLARTEIKVF
LQEWLKRIPDFRIREDDKPRCGSGSVNGMLYLPLVWDVAR

>BJG93_29300|KEGG|CYP1111A3|[Paraburkholderia sprentiae]pspw|BP

MAGVLRLEDFDDPKFNPFFEDSLAYGADADPYPRIAELREKAAVQEGDYRVLMGGAADLT

MSDVAHYSVFGYDEVSKILGDPATFSNKSYERNLGISFGRSISTMDAPEHPRYRRIFQKA
FLPNTVAAWGDTLVDPVVSELMSTFMDRGHADLVSEFTLHYPFNIIYRQLALPPEDVKIF
HKLAIAQTLVSIDVEHGVEASTKLGAYFARLVDERRSHPGDDLVSSLATAEVDGERLPEE
VVISFLRQLVNAGGDTTYRATSVLLLALLRNPVQLEAVRQDRTLVAPAIEEALRWDGPVL
MQSRMATRDVTLGGVEIPAGAFLDVVAGSANRDPSRFADPDKFDIHRKAVHRHFAFAYGP
HVCIGQHLARVEMTRALNAILDRLPNLRLDPAAPAPQINGIMMRVPHSIPVIFG

>BJG93_29165|KEGG|CYP101N1|[Paraburkholderia sprentiae]pspw|BP

MSVAGHGVTPELAPVPAHVLPERVFDIDIYNPPGAEDDYHLSLKRLHAEGIPDILWTPRN
GGHWIATRGDDIYHILKDYENFSSKQLTVPLVEHQPLPPIFFDPPQHTAYRALIAPAFMP
QAIGKLEEKAREMAIELIDGFYPKGEVEFVSGFAQHLPIGIFMNMVALPAEDREYLLGLA
DKMVRPVDANEKLQALGAIFQYLGVKVAERRANPGDDLISKIVHSNIDGRSLTDDEVRGL
CGLVLIGGLDTVASAMGFIARFLAGSPAHRKQLIDNPDLTQKAVDELLRRFPVVNQGRRI
THDFEYNGVAMKAGDMIIMPTTLHGLDERKFDDPLNVDFTRPTPIHSTFGNGAHRCPGSF
LARVELKVFLQEWLKRIPDFQIKPGEKAGVHGGVNGTLYRLPLVWQVAR

>BTO02_24655|KEGG|CYP1220A18|[Paraburkholderia sp.]para|BP

MSTTASGNTNRTDGTRLRQLTDLPSPKGLPIIGNLHQVSLRTFHLNLERWGRELGTPYTF
RMPGQQIVVLGDTDHVQGISRERPHRYRRFKPVETVLAEFGANGLFSAEGAAWEPQRRLV
MQALSIPHIRAVYPTLADITGRLYTRWMRAAQEQRTVDMVEEMKRYSVDVTSALAFGEDP
RTLERDTNVIQEHLKLIMPALMSRTSAPFPYWHYVKLPRDRRLDRALIEIHRYVRRMMER
ARERMRDDPSEMPRNLLEAMLAMRDMPDSGITDDQVSANVLTMLLAGEDTTANSLAWSTM
FIATDEPLQARLAKDARAVLGSAPVCTDYAKLKELDVFEAVCTETGRFKPVAALSSFEPL
EDVTMGDVLVPAGTFMFFLNRPAMHNPANFVNPERFDPDRWLQARDPARGAHEQRAYLQF
GAGPRVCPGRHLAGVEMRAALSMLAANFEAKLAVDASEIEEINDFIMKPSKMPMNLTIRP
RAE

>BTO02_25280|KEGG|CYP1220A19|[Paraburkholderia sp.]para|BP

MADLQFDAPPTLRTVADLPCPRGVPLLGNLFQMAPAKLHLTLEQWARQFGSPYRVQLGTI
PVTVWTQADLFQTVMRERPHLYRRFAPMESVMAELGGNGVFSAEGAAWEPQRRLVMRALS
FPNVKSFFPTLAAITGRFLVRLQDAAKERRTLEMTKELKRYTVDVTSTLAFGEDPHTLER
ERGVIQEQLALVPPALMRRVTALFPYWHYFTLPEDRRLARAMVEVHGYIRQMIERTRERM
HESPDAPPRNLLEAMLAARNEPDSTVTDEQISANVLTLLVAGEDTTANAIAWALMYVAAD
DRLQQRLFEQSRAVFGEAGICPDYDALKHLDLCEAVCNEALRLRPVAAIQTFEPLSDVQI
GGVAIPTGTRMLFLTRPAMLDPLNFAHPERFDPDRWRHPRDPSEGAHELKAYLTFGAGPR
VCPGRYLAAVEMRLVLSMMLRHFEIELAIDPAKIEEVSAFTMVPNTMPVRLKPRS

>CJU94_29980|KEGG|CYP1220A16|[Paraburkholderia aromaticivorans]parb|BP

MDTVSSSHIRYRQVSDLPCPRGLPFFGNLHQLPPTRLHLVLEQWARELGSPFRFQICRMP
VTVWTDPELCQTIMRERPHRYRRYASIESVLEEIGSNGLFSAEGAAWAPQRKLIMHSLSI
PHIKAFYPSLKAITERLYLRWQRAAARGEVVEMTEDLKRYTVDVTSALAFGEDPRTLEQD
RGVIQEHLALIFPMLMNRINAPFPYWRYVRLPRDRALDRSLIEVHRYVRNMMSRARERMR
DEPSESPRHLLEAMLTMRDAPQSDLTDDQVAANVLTLLLAGEDTTANSIAWTLLYVAADS
ALQQRLFDSSRGVLGSAPVCPDFASLKQLDLFEAVCTETGRLRPVAAVNSFEPLEDVCLA
DILLPAGTKMFFLNRPATIDARNFSNPLQFDPDRWLHDGSRARTTHDSRAYLQFGAGPRV
CPGRYLAAVEMRLVLSMLMANFSIRMAVDPDTIREVTAFTMVPSAMPVRLSTRAT

>C2L64_08965|KEGG|CYP195A6|[Paraburkholderia hospita]phs|BP

MHPSNATSNDADIARQFDLRHLDASFHADPYPVYHALRTHEPVKRMPDGSLFLTRYRDVQ
AVYRDPKAFSSDKKVEFAPKYGATPLFEHHTTSLVFNDPPLHTRVRKLIAGALTARAIAA
MEDGLVRLVDGLLDRAAERGEIDLIGDFAAAIPVEVIGNLLDVPHDEREPLRDWSLAILG
ALEPSLTTEQHERGNRAVTEFVAYLKGLVARRRAAPGDPQHDVLTRLIEGEANGEQLSEV
ELLQNCIFILNAGHETTTNLIGNGLVTLSVWTDERDALLREPSLIESAVEECLRFESSNQ
LGNRMTMVDTEIGGVPVAAGTPVTLCIGAANRDPEQFAQPDRFDIRRAPNRHLAFGFGIH
QCAGLSLARLEARIAIGRFVRRFPSYRISGEPTRGGRVRFRGYAAVPCAVE

>C2L64_25710|KEGG|CYP133C2|[Paraburkholderia hospita]phs|BP

MKFTDLSSPAYFDNPYPFYEGIRSAGAFVPLAPSIMLTGRHAVIDALLPDRRMGKTYMPS
VVARYGESAREQPVFQALERTFLMMNPPTHTRLRALLMKAFNARQIETLRTIAEETTDSL
IDGMQGKPEVDLVSEFAMPLPLQIICRLLDVPVEDGVRFGDAASLLVSAFDLAPMSADAL
ARANQAALELEQYFRAVVAQRRATPGHDIVSSLIQAEEGGERLSEDEIISNVILLFVAGH
ETTSNMLGNALIALHQHPAQLERLRGDPSLVSKAVAECARYDTAVQMVVRTAFEDINVDG
QTLPRGSIVFMLLGSANRDPERFDAPDTLDIGRDPSGHLLTFGAGIHYCLGARLAMMELE
IALHKLLTRLPQLRLTNLDALQWRRRNNLRGVESLIAAL

>C2L64_30050|KEGG|CYP105T10|[Paraburkholderia hospita]phs|BP

MTDDTSQQRCPLESDTFPWPRAAECPLHPPPRYASVRESEPIRRVKTWDGSSVWVVTRMK
DFREVLTSPHFSASPETHGYPSVSPARAAQSRSYQTFITMDPPEHGQYRRTLTKEFMMKR
IDELRPFVQSTLDALLDQMIAKGAPSDFIQDVALPLPSVVISIMLGVPYEDHARLQMLSS
NRMNLDISPELLTQSAQEMEQYIDRLLREKEQHPGEGNDLLCRLAVEWINTGKLSHADAV
QMGALLYLAGHETTANQIGLGLLSLLQDSQQTAALIAEPSLVRGAVEEMLRFHSITHMNS
ARVATADVMIGGQLIRKGEGVFAPVQAANHDPEAFPEPDRFDIRRDTRSASHVAFSFGVH
QCLGQPLARLELQVVFETLFKRLPSLRLAVPFDELEFKRNGNVFGLIALPVEW

>C2L64_30060|KEGG|CYP105T9|[Paraburkholderia hospita]phs|BP

MNMSETTLKCPFATESFPWPRDSAAPLIPPPQYARLREKATVVQVELWDGSLAWLVTDME
HFREVMTSPHFSASPLTPGFPFISPSRAAQSKSYQTFITMDPPEHGQYRRTLTKEFMAKR
MQELRPLVQQSLDRLLDEMESKGAPADFISGVALPLPSLVISIMLGVPYEDHDKLQKWSG
DRMDLSIAPEALTQSAKSMSQYIDALLLEKERNPGDGSDLLSRMAIEWINPGKVSHADAV
QMATLLYLAGHETTANQIGLGLLSLFQHPEQRDALMADGSLVKGAVEEMLRFHSITHMNS
NRVATEDVMIGGQLIRKGEGVLALLHGANHDPAAFPEPERFDIRRDTKDQSHVAFSFGIH
QCLGQPLARLELQVVFEMIFKRFPNLRLAVPADTLHYKDKSFVYGLKALPVSW

>C2L64_30120|KEGG|CYP105CV2|[Paraburkholderia hospita]phs|BP

MSGCPFSKPAFPMQRQCPFLPPGEYRDFQQQPGPVKVQMWDGKDAWLFTRYDDVRAVLGD

NRFSGDPHVSGFPSLSAARNAVLDLEPAFIRMDPPQHGQFRRMLTKEFMIKRVSDMRPRI
ESIFSRLVDELLEKGPPANLVEDLFLPLTSEVIAGLLDVPACDHGFFQEQSRLKVLLDVD
PSIPKAASDRILSYLDRLITERAKDAEHRTDLLSRLIVEQVRPGHLTHRELVIMAELLLM
AGHETTANQMALGVFSFLTNPDQLQLLRENPTLLRSAVEEMLRFHTIVHYNAFRVATEDV
EVAGQMIRKGEGVIALISGANHDATAFDQPERFDITRKADHHVAFSYGIHQCLGQPLARV
ELQVVFASLFERMPGLQFAVPLEDIKGKGDHFVQGLEALPVTW

>C2L64_30215|KEGG|CYP105T2|[Paraburkholderia hospita]phs|BP

MQKDLDVLVQDDTEREFPFQRRGNPLNPPLEYAHLRAEQPLAKVTLWDGSTAWIATRWDD
VRAILGSPHFSVDPFRPGYPSISPARAVQARSRQAFINMDDPDHARFRRMLTKDFMQKRM
SDLRPMVRKLVDELIDGMVEHGPPVDFVKHFSQPLPSMVISALLGVPYEDHATLGTWSNT
RNDHTASPEVVQQAVRRMEAHLGQIIAQKEADPGDGSDMLSRLVIEQILPGNVTREEVVR
IASLLYSAGHGTTSSQIGLGTLSLLMNPDQRAELEADPSLLSSAVEEMLRFHTIAHFNSA
RVATGNVMIGGQQILAGEGVYPLIFAANRDPAVFPNPDSFDIHRNPQEHVAFAYGIHQCL
GQPLARLELHTVFGMLFKRLPELKLAVPVEALTFNSTSQVYRLETLPVTW

>C2L64_49995|KEGG|CYP1049A4|[Paraburkholderia hospita]phs|BP

MAHQMTLRFEDGVTRFIECEEGERVTDAAIRARINIPLDCRDGVCGTCKAICESGEYVLG
DCVEDALSPEEATARKVLTCQMSPRSDCVIQIATNSDVSGTGPCSHRGRIVACHRASDTT
IAFSVELENRADLSFLPGQYVNIRVPGTDQTRSYSFSSGPSEPHLSFLVRNVRQGMMSTW
LCQTAKAGDPIEFRGPMGSFYLRPIERPVLFLAGGTGLAPFLSMLDKIVEDGGSPYPIHM
ILGVNSDADLVGIDRLEIDAQRLPNFTYACTVASPDSAHPSKGYVTHHISASQLNGGDAD
VYLCGPPPMVDAVRSFLSSEGLTPRNFYYEKFAGTGLVVQTGEERIAPVDVDEAFDLRMV
LELGAAQLTMGRLSTEQLIIFRQLAEATAQFVSGRRFTDVPRYIEANHAFHMFTIEASGN
AQLIALYRQLTVQDYIARALTDQIEIVGDIVQQHRDIVAAFELGDLNAARDVIALHALHS
RATMSRALEHMAMVKTTPMVAAPPSLLSAVPESAPQVCPFTSRTLPPYSHELDWPEGLEP
FKVVDDGSQGDPYEHYRWMREHAPVLRCQSATSDVWFLSRYDDVYQAIRNPKLFSSEVVS
PPPLTFLTLFDAPDHSRLRKVVQPSFLPLALDPFIRQIAQRAEDLLDAMIAKGGGDVVND
FAIPLSISTISTMIDVPNEDEEKMKFWSDETFSYFGRLARNAPGTGTDEQSAQAFFAYLK
RALERLVLTDSQSIGGHIARMWKDGLLSEKEAKELCAFVFIAGHDTTTILVANAFRMFAE

QPDLLGRIRENPADADRFVEEVARYRGTVQRVSRITTEATTVAGVDLPKGAVVRLLLSAA
NRDSRKFAEGETFDIDRDSSGHLGFGNGMHKCLGAPLAKLETLIATKALARKVGDIAFDP
ARPIQYVRGNNLTNSGPEHMFVKLGGLSA

>C2L65_08430|KEGG|CYP195A6|[Paraburkholderia terrae]pter|BP

MHPSNATSNDADIARQFDLRHLDASFHADPYPVYHALRTHEPVKRMPDGSLFLTRYRDVQ
AVYRDPKTFSSDKKVEFAPKYGATPLFEHHTTSLVFNDPPLHTRVRKLIAGALTARAIAA
MEDGLVRLVDGLLNHAAERGEIDLIGDFAAAIPVEVIGNLLDVPHDEREPLRDWSLAILG
ALEPSLTPAQHDRGNRAVTEFVAYLKDLVARRRAAPGDPQHDVLTRLIEGEANGEQLSEV
ELLQNCIFILNAGHETTTNLIGNGLVTLSEWTDERDALLREPALIESAVEECLRFESSNQ
LGNRMTMVDTEIGGVPVAAGTPVTLCIGAANRDPEQFAQPDRFDIRRTPNRHLAFGFGIH
QCAGLSLARLEARIAIGRFVRRFPSYRISGEPTRGGRVRFRGYAAVPCTVE

>C2L65_22700|KEGG|CYP133C2|[Paraburkholderia terrae]pter|BP

MKFTDLSSPAYFDNPYPFYEGIRSAGAFVPLAPSIMLTGRHAVIDALLPDRRMGKTYMPS
VVARYGESAREQPVFQALERTFLMMNPPTHTRLRALLMKAFNARQIETLRTIAEETTDSL
IDAMQGKREVDLVSEFAMPLPLQIICRLLDVPVEDGVRFGDAASLLVSAFDLAPMSADAL
ARANQAALELEQYFRAVVAQRRATPGHDIVSSLIQAEEGGERLSEDEIISNVILLFVAGH
ETTSNMLGNALIALHRHPAQLERLRGDPSLVSKAVAECARYDTAVQMVVRTAFDDIDVDG
QTLPRGSIVFMLLGSANRDPERFDAPDTLDIGREPSGHLLTFGAGIHYCLGARLAMMELE
IALHKLLTRLPQLRLTNLDALQWRRRNNLRGVESLIAAL

>C2L65_33045|KEGG|CYP105T7|[Paraburkholderia terrae]pter|BP

MQHESNELIEARAERDFPFQRTGCPLRPPSEYAQLREEQPLAKVRLWDGSEAWIATRWDD
VRAILGNPQFSVDPFREGYPSVSAARAVQARSRQAFINMDDPDHARFRRMLTKDFMQKRM
SDLRPMVRKLIDELIDKMIEHGPPLDFVTHFSQPLPSMVISALLGVPYEDHTKLGTWSST
RNDHTASPEVVQEAVRNMEVHLGEIIARKEENPGDGNDMLSRLVVEQIRPGHASREEVVR
IASLLYSAGHGTTSSQIGLGTLSMLMNPSQRAELEADPSLLSGAVEEMLRFHTIAHLNSA
RVATADVMIGDQLIRAGEGVYPLILAANRDPEVFPEPDTFNIHRKPQEHMAFAYGIHQCL

GQPLARLELHAIFSTLFKRLPDLRLAVPVESLTYNGTSQVYRLESLPVTW

>C2L65_33140|KEGG|CYP105CV1|[Paraburkholderia terrae]pter|BP

MSGCPFSKPAFPMPRQCPFVPPAEYSAFQQQPGPVKVQMWDGKDAWLFTRYDDVRAVLGD
NRFSGDPHVGGFPSLSPARNAVLDLEPAFIRMDPPQHGHFRRMLTKEFMIKRVSDMRPRI
ESIFNRLVDELLEKGPPANLVEDLFLPLTSEVIAGLLDVPACDHGFFQEQSRLKVLLDVD
PSIPKAASDRILTYLDRLITERTKDAENRTDLLSRLIVEQVRPGHLTHRELVIMAELLLM
AGHETTANQMALGVFSFLTNPDQLQLLRETPSLLRNAVEEMLRFHTIVHYNAFRVATEDV
EVAGQMIRKGEGVIALISGANHDATAFEKPDRFDITRKADHHVAFSYGIHQCLGQPLARV
ELQVVFASLFERMPGLRFAVPLEDIKGKGDHFVQGLEALPVTW

>C2L65_33200|KEGG|CYP105T8|[Paraburkholderia terrae]pter|BP

MNKPETTLQCPFATERFPWPRDNAAPLAPPHQYATLREQAPVVQVSLWDGSLAWLVTDME
HFREVMTSPHFSASPLTPGFPFVSPSRAAQSKSYQTFITMDPPEHGQYRRTLTKEFMAKR
MQELRPLVQRSLDQLLDDMANQGGPADFIAAVALPLPSLVISLMLGVPYADHDKLQKWSG
DRMDLSIAPEALTQSAKSMNEYIDALLREKERDPGDGGDLLSRMAIEWINPGKLSHADAV
QMATLLYLAGHETTANQIGLGLLSLFQHPEQRKALTEDSTLVRSAVEEMLRFHSITHMNS
NRVATQDVVIGGQLIRKGEGVLALLHGANHDPVAFPEPERFDIRRDTKDQSHVAFSFGIH
QCLGQPLARLELQVVFETLFKRFPDLRLAVPEDALQYKDKSFVYGLKALPVIW

>C2L65_33210|KEGG|CYP105T11|[Paraburkholderia terrae]pter|BP

MTDDTAGGRCPFEGDRFPWPRAMQEVLQPPPRYASVRESEPIRRATTWDGNTVWVLTKMK
DIREVLTSPHFSASPQTHGYPSVSPARAAQSRSYRTFITMDPPEHGQYRRTLTKEFMMKR
IDELRPFVQTTFDTLLDQMIENGAPADFIRDVALPLPSIVISIMLGIPYEDHARLQLLSS
NRMRLDISPELLAQSAQEMEQYIDRLLREKEQEPGDGEDLLSRLALEWINPGKLSHEDAV
QMSVLLYLAGHETTANQIGLGLLSLLQDPQQKAALIAEPSLARGAVEEMLRFHSITHMNS
ARVATADVMIAGQLIRKGEGVFAPVQAANHDPEAFPDPERFDIRRDTKHASHVAFSFGVH
QCLGQPLARLELQVVFETLFKRLPSLRLAVPFEQLHFKCNGNVFGVETLPVAW

>C2L65_38220|KEGG|CYP1049A4|[Paraburkholderia terrae]pter|BP

MAHQITLRFEDGVTRFIECEESERVTDAAIRARINIPLDCRDGVCGTCKAICESGEYVLG
DCVEDALSPEEASARKVLTCQMSPRSDCVIQLATNSDVSGTGPSSHSGRIVACQRASDTT
IAFSVELENRADLSFLPGQYVNIRVPGTDQTRSYSFSSGPSEPHLSFLVRNVRQGMMSTW
LCETAEAGDPIEFRGPMGSFYLRPIERPVLFLAGGTGLAPFLSMLDKIVEDGGSPYPIHM
ILGVNSDADLVGIDRLDIYAQRLPNFTYACTVASPDSAYPNKGYVTHHISASQLNGGDAD
VYLCGPPPMVDAVRSFLSSEGLTPRNFYYEKFAGTGLVVQTGEERIAPVDVDEAFDLRMV
LELGAAQLTMGRLSSEQLIIFRQLAEATAQFVSGRRFTDVPRYIEANHAFHMFTIEASGN
AQLIALYRQLTVQDYIARALTDQIEIVGDIVQQHRDIVAAFELGDLNAARDVIALHALHS
RATMSRALEHMAMGKTAPVVAAPPSLPSAVPESAPQVCPFTSRTLPSYSHELDWPDGLEP
FKVVDDGSQGDPYEHYRWMREHAPVLRCQSATSDVWFLSRYDDVYQAIRNPKLFSSEVVS
PPPLTFLTLFDAPDHSRLRKVVQPSFLPLALDPFIGQIAQRAEDLLDAMIAKGGGDVVND
FAIPLSISTISTMIEVPNEDEEKMKFWSDETFSYFGRLARNAPGTGTDEQSAQAFFAYLK
QALERLALTDSPSIGGHIARMWKDGLLSEKEAKELCAFVFIAGHDTTTILVANAFRMFAE
QPDLLGRIRENPADADRFVEEVARYRGTVQRVSRITTEPTTVAGVDLPKGAVVRLLLSAA
NRDSRKFAGGETFDIDRDSSGHLGFGNGMHKCLGAPLAKLETLIATKVLARKVGDIAFDP
ARPIQYVRGNNLTNSGPEHMFVKIGGLSA

>C2L65_44715|KEGG|CYP1176B1|[Paraburkholderia terrae]pter|BP
MGCPVHERLNDVPADRVASFNYRTGEAMVVNPWTVLHDLGAGPGFFYSPELGGHWCAAKR
ETVIDILRNWELFSCRNIRVPRVDRTVPSIPHHMDPPEHGKYRISVVELFGHRILDELTP
AIRATARDLANGLAARNEAEFVRDFAMRMPVEIFMRMCGLPLERRDEMIGWVQSYFHGAS
QQESNDAHGKSIAFLSGWLDEQLADPEKSGGHIMPSLLKTRVDGRPLSREEMLSILITLF
NGGLDTVAAQMTHIMRYLAEHADQRQKLLDNPEKIPDAVEELLRRFSIISIGREVTRDAT
YEGVALRKGDMVLCLISAAGLDEEIFPRALEVDFDRSNRRQHCAFGSGPHMCVGAPLARL
ELRIMLEELLPRLPGLRVRPGASLSYHTGVTIGLVDLPLQWGESTHVS

>C2L65_44875|KEGG|CYP1487A2|[Paraburkholderia terrae]pter|BP

MEISMNSSVQTDGGAARCPFHSAFDPLEPAQIADPFPILAKMREESPVFYMEKYDLWVVT
RREDVLAVYKDMVSFSNGNAHKPLSPKSQAVIDRVGEDWPLPVDGSLNAMDPPEHLPLKQ
LFMSVFHKALPGTDEWLDEKLNKLVDRFANDGEADLVPSFFWPTTVGAVAHLIGAADSET

ERFNEWAENWFELTGSTLLSPERAEACWMAFIDFEQWAYALLEARRRDPQHDLISKLIEA
QRSGAAVTDRQIVTNMIGMVAAGTDTTANTIAQMIYTLLKNPDQLQRVREDPKLRPQMIE
EVIRLRLPIRGVVRTMTKDVEMGGVTLPKGAKVYIHLGSANHDPAMFANPDAFDIDRNNA
HKHVSFGAFNRACVGAPLARLEIAKALDIILDRLPGLRLADENEPLRYTESIIVPSLKSL
RVRWDVPAKAV

>CUJ91_08245|KEGG|CYP195A3|[Paraburkholderia graminis]pgp|BP

MSTATADAATLARNFDLRHLSPEFYADPYPVYHALRAHEPVKRMPDGSLFLTRFRDVQAV
YRDPKTFSSDKTVEFRPKYGDSPLYTHHTTSLVFNDPPRHTRVRKLIAGALTARAIAAME
PGLVQLVDGLLDAAAERGRIDLIGDFASAIPVEIIGNLLDVPHAERAPLRDWSLAILGAL
EPSLTDAQFERGNRAVSEFVDYLRGLVARRRREPGDPQHDVLTRLIQGEAGGEQLSEEEL
LQNCIFILNAGHETTTNLIGNGLVTLTEWPDQRAALLAEPSLIESAIEECLRFESSNQLG
NRMTTVDTEIGGLPVARGTPVTLCIGAANRDPEQFAEPDRFDIRREPNRHLAFGFGIHQC
AGLSLARLEARIAIGRFVRRFPSYQLDGEPTRGGRVRFRGFAEVPLLCR

>CUJ91_23590|KEGG|CYP1485A3|[Paraburkholderia graminis]pgp|BP

MTCEIPDPGSYSVLPSIIGTGSGDPYPLYGWLRAHRPLCRDASGVWLASSHALVTRILDD
ARFSARPPPGTEPLDDPLGYASMVVFQTGEAHERLRRFLGPLFSRKALVSLQDFIDDEVA
RLVDQCEHLPRFDLVANIASVLPVRTICRLLGFPDAHGASYLEASAGAWQLISGAPMSAA
EHALAAMQTEAFLAQVESFMRAIDPHAAPDHAASRFLRLEQEGGIGRREMLVNILFLFIA
GYGTTLLSIGNSVAALLREPRAWQALKDDISLVPQAVRELYRYDPAVQSVFRYAWDDVEI
DGCRIRRGERVMPLIGSANRDPAVFAAPDEICFEREAGRSLTFGAGPHSCMGLALARMQL
ESLLRALTRRMPDLRLAPGQQARQHGSFHGYDRLMVER

>CUJ87_03085|KEGG|CYP1220A2|[Paraburkholderia caledonica]pcj|BP

MAIASTRTLKVREISDLPAPRGLPLIGNAHQLAPSRTHLILERWAHELGTPYRFQIGSRP
VTVWDDTELCHSIMRERPNGYRRYSPIEENLDEMGSNGVFSAEGTAWEPQRKLVMQALSV
AHVKAFYPTLSAITERLRVRWERAARSGRVVEMTEDLKRYTVDVTSALAFGDDPGTLEQD
GNVIQEHLALIFPMLMNRINAPFPYWRYVKLPRDHRLDHALAEVHRYVRLTMNRARERMR
DRPHDEPGNLLEAMLRFRDTPGSRITDDEVAANVLTMLLAGEDTTAHSIAWALLYLSSDD

ALQSRVSSRARTVLGAAGVCPTYETLKTLDLCEAVCTEASRFRPVVPVHAFEPLQDVEHR
GIHLPAGSRMFFLTRPSMLDASNFEHPEKFDPERWMHELTIQRGAHETRAHLQFGGGPRV
CPGRHLSTVEMRLVISMLSAGFVSRLAVDTSSIREVAAFTMVPSAMPVRLCLRQ

>CUJ87_07550|KEGG|CYP195A3|[Paraburkholderia caledonica]pcj|BP

MPTATAGDTAILARDFDLRHLSPEFYANPYPVYSALRTHEPVKRMPDGSLFLTRFRDVQA
VYRDPKTFSSDKTVEFKPKYGDSPLYTHHTTSLVFNDPPRHTRVRKLIAGALTARAIAAM
EPGLVRLVDGLLDAAVERGRIDLIGEFASAIPVEIIGNLLDVPHAEREPLRDWSLAILGA
LEPSLTSEQFERGNRAVTEFVDYLRDLVARRRREPGDPQHDVLTRLIQGERGEGGDGEQL
SEEELLQNCIFILNAGHETTTNLIGNGLVTLTEWPDQRAALLEDPSLIESAVEECLRFES
SNQLGNRMTTSDTEIGGVPVARGTPITLCIGAANRDPEQFAEPDRFDIRRAPNRHLAFGF
GIHQCAGLSLARLEARIAIGRFVQRFPAYRLDGEPTRGGRVRFRGFASVPLLCR

>CUJ87_11010|KEGG|CYP133C8|[Paraburkholderia caledonica]pcj|BP

MNFATFSSPAFFTDPYPMYEKLREAGPLLPVGPNALVTGRFEIIDALLRDRRMGKTYLQS
VRARYGDAATEQPVFQALSRTFLMMNPPAHTKLRTLLMKAFNARQVEKQREIVEATSREL
LDRIGSAAQFDLVSGYALPLPVEIICRLLDIPSSDGAQLGVAASRLVAAFDLAPLDEAAL
ADANEAALTLERYFRTVIEQRRARPGDDIVSSLVHVEDEGATLSDDEIVSNIILLFIAGH
ETTSNMIGNAMVALFRHPARLEALRREPAAIAKAVAECMRFDSSVQMVVRTALEEVVIHD
VTLKPGTIVFMLTGAANRDPAVFPHPDQLDFDRGSKVPSLAFGGGIHYCLGARLALLEME
VALRDLLARFPQMQPEDLDQLNWHRRNNLRGVQSLRIHT

>CUJ87_32005|KEGG|CYP1097A18|[Paraburkholderia caledonica]pcj|BP

MPPIDAHQPSATHTGSTLGLRRLADLPGPRALPLLGNLHQFDFRRLHQTLEKWTRQHGPV
YRVTVPRSAVLVLADPDLVGKVLRERPDTFRRPGVLAAVAAELGGMTGLFDSEGATWRNQ
RRMVMQAFAPQPVKAYFPSLVEVARRLQRRWEDRAEGGDVIDLGADLRLYSVDIVAGLAF
GTDVNTIEMGDHPVQRNLDMILQGVARRTMMPFPYWRYVRLPADRRLERSVIALRDAVFD

LVAQAKERMRLDPLRAANPPNMLEAMLAAAAKGGSGVTEADVAGNVSTMLLAGEDTTSNS
LVWLIWLLQRHPDALQRAREEVLRCVPSLDELTIEQIRRLDFLDACTHESMRLKPAAPFI
SVEALEDTTVGDVAIDKGTIVLCLMRDTSPDNTHFENAAQFDPRRWLLDSVNRKAAMPFG
AGPRICPGRYLALLEIKVAMVMLLARFDIASLATAHGGEPDERLAFAMSPETLHIRLRLR
V

>CUJ90_07885|KEGG|CYP195A3|[Paraburkholderia terricola]pts|BP
MPPATTSDAAALARDFDLRRLSPEFYANPYPVYHALRTHEPVKRMPDGSLFLTRFRDVQA
VYRDPKTFSSDKTVEFRPKYGDSPLYAHHTTSLVFNDPPRHTRVRKLIAGALTARAIAAM
EPGLVRLVDGLLDAAAERGRIDLIGEFASAIPVEIIGNLLDVPHAERAPLRDWSLAILGA
LEPSLSDAQLERGNRAVSEFVDYLRGLVAQRRREPGDPLHDVLTRLIQGEEGGEQLSEAE
LLQNCIFILNAGHETTTNLIGNGLVTLAEWPEQRAALLREPSLIESAVEECLRFESSNQL
GNRIATLDAEIGGVPVARGTPVTLCIGAANRDPEQFAEPDRFDIRRAPNRHLAFGFGIHQ
CAGLSLARLEARIAIGRFVQRFPAYRLHGEPTRGGRVRFRGFAEVPVELDPAARRTP

>CUJ90_19250|KEGG|CYP1220A16|[Paraburkholderia terricola]pts|BP

MDTVSSSRVRCRQVSDLPCPKGLPFVGNLHQLSPTRLHLVLEQWAQELGSPFRFQICRMP
VTVWTDPELCQAIMRERPHRYRRYASIEPVLEEIGTNGLFSAEGAAWAPQRKLIMQSLSI
PNIKAFYPGLKATTERLYRRWQSAAAHGKVVEMTDDLKRYTVDVTSALAFGEDPRTLEQD
RGVIQEHLALIFPMLMNRINAPFPYWRYVKLPRDRQLDRSLVEVHRYVRSMMSRARERMR
DEPSESPRHLLEAMLAMRDTPGSGVTDDQVAANVLTLLLAGEDTTANSIAWALLYIAADP
ALQRRLFDSARGVLGSESVCPDFTSLKQLDLFEAVCTETGRLRPVAAVNSFEPLEDVCLA
DIMLPAGTKMFFLNRPATLDEKNFSNPLQFDPDRWLHDGSRPRTAHDPRAYLQFGAGPRV
CPGRYLAAVEMRLVLSMLMANFSIQMAVDPGTIREVTAFTMVPSAMPVRLTARTA

>CUJ90_31030|KEGG|CYP1485A6|[Paraburkholderia terricola]pts|BP

MTVTLADPDFYSALPSIIGTGSADPYPVYAWLRANRPLCRDASGVWLASSHALVTRILED
ARFSARMPPSATPFDDALGYTDMIVFQNGAEHDRLRRFLAPLFSRKALAALQAFIDDEIA
RLVERLRDTPRFDLVADIASVLPVRTICRLLGFPDAHGARYLEASTGAWQLISGAPLAPA
ERMSAITQTEAFLAQVESFIADIDPDAMPDHAASRFLHLEREGGIGRREMLVNILFLFIA

GYGTTLLSIGNGLAALLRAPRAWQALRADPALVPQAVRELYRHDAAVQAVFRYAWEDVEI
DGQRIRRGELVVPLLGAANRDPAAFDAPDEIRFEREAGRSLTFGAGPHSCMGLALARMQL
ESLLRSLLAHMPDLRLAPGPQVRQQHGSFHGYDRLVLERAGG

>DSC91_002542|KEGG|CYP105B78|[Paraburkholderia caffeinilytica]pcaf|BP

MTDEGGMLSSHRQGENMNTIVDGATEGILDYPTPRDPAAPFLPSPELRALRDAGVEITRV
RIWDGSTPWILLSHAAQRKIMGDPRVSANDHLPNFPFMNRAIAETIHDRPKTIFDTDGED
HARLRRMLTNSFTRHRMEKIRPQIQQATDDLIDRMLAGPKPTDLVEALSLPLPSLMICDL
LGVPYDDHDFFEKHSKIANARDKSPDENRSSTQALGEYIGKLIQQKMDHPEEDVVSDLGQ
RVKTGDLDMAGAVQLGVILLIAGHETSANMITLGTALLLQNPDQMKLLRETDDPKIVAGA
VEELLRYLTIPHLLQRRIAIEDIEFNGTIIRAGEGICSPLPSANFDPVAFPDPAKVDITR
DARHHHAFGWGPHQCIGQQLARVELQVVYPTLLRRVPTLQLAVPFAELRFKHDSLAYGIE
ELPVMW

>DSC91_007111|KEGG|CYP195A3|[Paraburkholderia caffeinilytica]pcaf|BP
MTPATASDASILARDFDLRQLSPAFHADPYPVYHALRAHEPVKRMPDGSLFLTRFRDVQA
VYRDPKTFSSDKTVEFKPKYGDSPLYSHHTTSLVFNDPPRHTRVRKLIAGALTARAIAAM
EPGLIRLVDGLLDAAAARGRIDLIDAFASAIPVEIIGNLLDVPHAERAPLRDWSLAILGA
LEPSLSAAQLERGNRAVSEFADYLRDLVARRRRDPGDPQHDVLTRLIQGELGELSGEQLS
ETELLQNCIFILNAGHETTTNLIGNGLVTLAAWPEQRAALLREPSLIESAVEECLRFESS
NQLGNRMATVDTQIGGVAMARGTPVTLCIGAANRDPEQFAEPDRFDIRRDPNRHLAFGFG
IHQCAGLSLARLEARIAIGRFVQRFPAYRLDGEPTRGGRVRFRGFAEVPVEVEPAHSGSA
SQ

>FNZ07_03695|KEGG|CYP133C9|[Paraburkholderia megapolitana]pmeg|BP

MNFASFSSPAFFTDPYPIYEKVREAGPLLPVAPNVLVTGRFEIIDALLRDRRMGKTYLQS
VSVRYGEAATQYPVFQALSRTFLMMNPPSHTRLRTLLMKAFNAKQIEKLREIVEMTSDDL
LARIGSKAEFDLVAEYALPLPVEIICRLLGISPADGLKLGAAASRLIAAFDLAPLDEARL
AEANEAALTLEWYFRQVVEQRRASPGDDIISSLVSVEDDGATLSDEEIISNVILLFIAGH

ETTSNMIGNAFIALFRQPVQLLALKRDPALIPKAITECMRYDSSVQMVVRTALEDVAIGD
ATLARGTIVFMLVGAANRDPAVFAHPDQLDFSREGNVPSLAFGGGIHYCLGARLAQLEIE
VAIGALLSRFPDMQPTNLDQLIWHPRNNLRGVESLRVVTAAA

>FNZ07_08320|KEGG|CYP116B207|[Paraburkholderia megapolitana]pmeg|BP

MSDAINPNRTASCPFHDNTGSPPSTSDGCPINHRAADFDPFEDAYQQDPPEYVRWAREQQ
PVFYSPKLGYWVVTRFDDIKAIFRDNMTFSPSIALEKITPTGPEANEVLASYGYAMSRTL
VNEDEPAHMPRRRALMEPFTPEALERHKPMVRRLVRQYVDRFINDGKADLVDQILWEVPL
TVALHFLGVPEEDMDTLRKYSIAHTVNTWGRPKPEEQVEVAQAVGKFWRFAGNVLEKMRS
NPNGHGWMQYGIRKQKELPEVVTDSYLHSMMMAGIVAAHETTANATANALKLLLQHPQAW
RDICDDPRLIPNAVEECLRHNGSIAAWRRLATRDVTISGVEIPAGSKLLIVTSSANRDER
QFAGADLFDIRRENASDHLTFGYGSHQCMGKNLARMEMQIFLEAFTRRLPHMRLAEQRFS
YVPNTSFRGPEHLLIEWDPALNPERNDLSVLRPRTAVRIGEPSAHAISRPVVVTSVIYAG
ADIVRVKLASRDGRPLPRWTPGSHIDVECGDTGISRQYSLCGNPAEAETFEIAVLKEHDS
RGGSVWLHDAVKPGALLHIRGPRNHFRLDETAQRVVLIAGGIGITPISAMARRAKELGID
YEVHFSGRTRASMVLLDELSALHGDRLRVYVSDEDTRNDFSALRVDDATQVYACGPANML
DALEQIATNWPDNTLKIEHFVSSIGTRDPDREHAFDVELKDSGLVVTVAADQTLLAALRQ
ANVDVQSDCEEGLCGSCEVRVLAGEVDHRDVVLTKTERQSNTRMMACCSRAQGTRLTLEL

>FNZ07_10045|KEGG|CYP133C13|[Paraburkholderia megapolitana]pmeg|BP

MNYTDLASPDFYRDPYPVYQLLRQAGNLVPIAPRTLITGHADIARTVLADRKMGKSYLEA
VRTRYGEAGVSQPAFQALSKMLLMMNPPAHTRLRALLMKAFNSRQIDLLRDISQSTADEL
IDALEANRPIDLVSEFAAPMPIRIICKIMDVPLDHAPTLGANVGRLAQTLEAAPLSPEQI
AAANTALENLEHYFKDIIATRRSHPGTDLISTMLSINDNGETFSENEIIWNVIFIFAAGH
ETTSNMIGNALIALHKHPDQLEKLRARPELLTQAINECMRYDSSVQIVQRVALEDTDIHG
VAVPKGTIVLLSLGATNRDPMQFDQPDTLLIERPESANAPLVFSGGIHYCLGARLAMFEL
ETALGTILRRLPDLRLTNLDALQWHQRNTLRGVTSLLGHIDQRPLAA

>FNZ07_25550|KEGG|CYP1043A12|[Paraburkholderia megapolitana]pmeg|BP

MAAALGIVLGFAVFASIPWWLPQRVLALRARIFVRINGEEGIAVPGPTVSAAQFMQVYSH
PAANGRSKGAGLSDLFWYWLSPGPEIHQEHLEAGARYDEVARATRQFLALPKVNAEELAA
RCAERAYADLSGKNMVLVRLRNLMMSVWADFYYQLVFGEPCTPTARKLIVDNADDVVTAL
KCCGLRHMKKRERLTAFLIEKIKAGKLTNPLPDGLSIDEQALYLQGVFFNTAIVQMSEAM
THLILAIAQHQEVQQALVDNDHDNHYLDQVIAETLRAFPLFGIAHRIATADIAVDDHTTI
SQGSVLCFNYSDYQRTGFDDPERFDPTRWNGLSKRDATYIPFGVTANRPCPAQAVAMVTM
RAAGRELLKRFACYSSASHTRSIPHRGPCLLVARGGAEHPRVRSGVLAFMALRDRWEDVW
RSLLQLVLGTWMVWHARRLRLCQRHFEAARCPVHADTR

>FNZ07_26685|KEGG|CYP1686A1|[Paraburkholderia megapolitana]pmeg|BP

MPQRRRASPAATATDCMHGDPAHLARDVNGNSCRGGPMSAKPIQTVKGWPVLGSIMHYKR
DALSLFLNAARDLGDVSAIMMGPTRVVLVNSPQHVAEILQDRTDAFGRSPSYRELELLLG
AGMLTTEGAQWKAQRAQAQPAFQKKQLTSMIPTIREKIDQVVGEWMPAGQTGLRRRDLFA
DMLDMSMRITLRTLFGQVHELAHRKMITALEDGSRYVIQRLEGIVNLPPHWPTPSAKEFW
KNRAVLDASVLDIIRQYQRGTQPPNLLGMVLEGDGPQDEENIKNQLLTLFITSYETVATG
MTWVFHFLSQHPRWYQAVVEELERYDELGYDDIFSLTTTNAVIHESLRLMPPIWSFSRTA
KRDVTIDGYPIAAGTMVVVSPYCLQRHPSHWDDPDTFNPARWTAGSSTSKRVSAFLPFGT
GPRICVGMNYAMMLLPLILKGACRRGTFSLDSAHPVRIKAGVTIRPVGGLMGHWTSHERL
LEQKGIAT

>G5S35_09305|KEGG|CYP116B212|[Paraburkholderia tropica]ptro|BP
MSETPSQTSGRCPFPHAAQAVPNTQDAARCPMHANHAVSTHAAAFDPFSDGYQQDPPEYV
RWAREQEPVFYSPQLGYWVVTRYDDIKAIFRDNLTFSPSIALEKITPTGPEANAVLASYG
YAMNRTLVNEDEPAHMPRRRALMEPFTPEHLKHHEPMVRRLTREYVDRFIDSGRADLVDQ
MLWEVPLTVALHFLGVPEEDMDLLRQYSIAHTVNTWGRPRPEEQVAVAHAVGNFWQFAGK
VLDKMRQQPDGPGWMQYGIRKQQEQPDVVTDSYLHSMMMAGIVAAHETTANATANAMKLL
LQHPRAWREICEDPSLIPNAVEECLRHNGSVAAWRRLVTKDVRIGGIEIAAGAKLLIVTS
SANHDERHFADADLFDIRRDNASDHVTFGYGSHQCMGKNLARMEMQIFLEEFTRRLPHMR
LAEQQFSYVPNTSFRGPEHVWVEWDPKQNPERANRALLDAEVPVRIGEPSAHALSRPVVV
ERVERVAQDIVRLRLVAPDGGVLPRWAPGAHIDVECGESGVSRQYSLCGDPADTHAFEIA
VLRETESRGGSVWVHDTLREGMRLKIRGPRNHFRLDEEGRRAIFIAGGIGITPVSAMARR
ARELGIDYELHYSGRARANMALIDELTQLHGERLCVHVSEEGTRNDFGALRVDALTRVYA

CGPARMLEALEAASAAWPEDALRVEHFAASVGKLDPADEHAFEVELKDSGLVLPVAADQT
VLDALRRANIDVQSDCEEGLCGSCEVRVLAGEIDHRDVVLTRAERDTQTRMMACCSRAKG
QRIVLEL

>G5S35_20475|KEGG|CYP1220A14|[Paraburkholderia tropica]ptro|BP

MSTASTGSATALRQVADLPCPRGLPLIGNLLQLEPTRLHQILERWAEELGTPYRFQVSSI
PITIWTDTELIQSIMRERPHRYRRYSPIESVFDEMGANGLFSSEGVQWESHRRLVMQALS
IPNIKAFYPTLAEITQRLRTRWLRAAERGETVEMTDDLKRYTVDVTSALAFGEDPRTLDQ
DHGVIQEHLERILPAVMGRINALFPYWRVFKLPADRKLDHSLAEVHRYIDSMMARARTRM
RDEPSELPRNLLEAMLVQQQHDGEPISDAQIRANVLTLLLAGEDTTADSIAWTLPYLAAD
AALQTHLGEEAHRVLGDAPVCPDYATLKDLDIFEAIAIEATRLRPVAAVHSFEPLEDVVV
DNVALPKGTRMFFVARPAMIDEKNFVDPQRYDPWRWMRKGEHDAPREGAHGAHDVRAYLQ
FGAGPRVCPGRHLATVEMRLALSMLMNEFEIELAIDPEQIKEVSAFTMVPDTMPVKLRKR
VR

>G5S35_27570|KEGG|CYP133C11|[Paraburkholderia tropica]ptro|BP
MQFSDFTSPAFFANPYPAYEKIRAAGALLPIAPQVFVTGRFDVIDALLRDPRMGKTYLQS
VAARYGEAATARPVFQSLNRTLLMMNPPTHTRLRALLMKTFNARQMEKLRDTVEATTRAL
LDAIGTNAGFDLMSDFALPLPVRIICQLLDVPLEDAHALAGAAGRLVAAFDLAPLDAQAL
SAADEAALTLERYFQNVIEQRRSQHADDLISALLHVEVDGAKLTDEEVVSNAVLLFIAGH
ETTSNMIGNTMLALFRHRDRYQALQDDRTLLPRAIAECLRYDGAVQMVVRTAFDDIDVGD
SKLARGSVVFMLIGAANRDPQAFTHPDQLDFARDDTAKSLSFGAGIHYCLGARLAMLEME
VAIGHLLDRFPALQAENLDALVWQHRNNLRGVTALPVRTGI

>G5S35_28475|KEGG|CYP1112A12|[Paraburkholderia tropica]ptro|BP

MRSWPVTPRSSGIDLWAADVLVDPYPTYRALRELGDVVYLTHSNLYALTGYDVIKDALAD
PARFSSAQGVGFNDGVNQALQGTSLASDPPTHTQLRAALSENLTPRALRGLGEQIKANAQ
ALVARLVKQPSFDAIDEIARRFPIGVVADLVGFQGEARANMLRWGQAAMEVIGPMNQRTV
ENFPIAGELYGYCSQITQDALTPGSVGWGVFEAENNGMIPAGAARHIIHQYLGAGLDTTL
AAIGNTVALFGRFPEAFAKVRADLSLVPSAFNEVLRYYSPVHVWSRVTAQDVEVGGATIP
GGSRIAVMLGAGNRDPRHYANPDIFDVTRNPIDHLSFGYGPHGCAGQGLARMEGAAIIEA

LATQIKSFRIGEVARAPSNVTVSFDTVPVIGLEVA

>I6I06_04640|KEGG|CYP195A3|[Paraburkholderia ginsengisoli]pgis|BP
MTPATASDASILARDFDLRHLSPAFYADPYPVYHALRAHEPIRRMPDGSLFLTRFRDVQA
VYRDPKTFSSDKTVEFKPKYGDSPLYAHHTTSLVFNDPPRHTRVRKLIAGALTARAIAAM
EPGLVRLVDGLLDAAAERGRIDLIGEFASAIPVEIIGNLLDVPHDEREPLRDWSLAILGA
LEPSLSDAQLARGNRAVSEFVDYLRDLVARRRREPGDPQHDVLTRLIQGEAGGEQLSEPE
LLQNCIFILNAGHETTTNLIGNGLVTLTEWPEQRAALLREPSLIEPAIEECLRYESSNQL
GNRMATVDTEIGGVAVARGTPVTLCIGAANRDPEQFAEPDRFDIRREPNRHLAFGFGIHQ
CAGLSLARLEARIAIGRFVQRFPTYRLAGEPTRGGRVRFRGFASVPVEVAV

>I6I06_29345|KEGG|CYP1013A6|[Paraburkholderia ginsengisoli]pgis|BP

MNTKQRAPGPRGSLVMGNLEEYKRDPITMLLKLQQQYGDIARNRLGPFLTHALAHPDHVQ
YVLQENHRNYVRGRFYESFKLFFGDGLLTTDGDFWRRHRRAVQPLFHKKQVEEHIAAVGD
AALALVRRWQALPSGEPVEVVEEMMHLSLSMLGLMVFNTDISQHAQQVGPSVRFGIAAMM
PQGNMNDFIPRWMPTPFNRRIAQARRNLDVIIGKIVEDHRAGHCEASDVISLLLNSRHPE
TGTPMSEQEVHDEVMTVFLAGHETTGSGMAWALYALAQYPAVLRRLRDELDARLGGRAPT
LEDLEALPYLTQVVDEVLRVYPPIWGFTRDLAEDDEIGGFHIPAGSSVFMSPYVTHRHPA
FWNNPEAFDPENFASNAPNRHRFGYFPFGGGMRKCIGFQTALLQMRVLTAVVAQHFDLNA
LPGHPIERGALISLRPLQGIRLIVKPRQRQSPTQRPDPGMGAASPTSDAACGAKPQREVT
SGCPFAEAPALPSSGPLSDEVVVVKPANPEPRSAPVLPPAATGMVPALRFTWFPSEIEAL
PSTPSPALGGKRVVIVNGRAGTAERVAVALLRACAKATVFAPGVDADPVEAAKALVKQSG
PFDGIVDLGLEESFSLAAANQWEMPMRHSIAMLQACYGDWEVEESASRLFYMAVTWMDGS
MGYGDAVAQPLGGLWSGLAKTLPQEVPNCNVRVLDVAPNEAGRVDQLVVRELYRWGLFEV
GYRAGRRYTLKARRSELPALPAGSAPDLGPGDVVLFSGGARGIGLLCAKAMAGRYGCTVI
ITGREPLPDGSEPWMTLDDAGFSRYGQEQLRLATRERTPKRIREELARLQRRRELKASLD
EIASCGLPVQYRVCDVTDAAAVRALCGELRDVLRVVVHNAGVDRPVRLPQKSADSFIDTV
RTKVGGFANLCAAVADCPRLVQFCNVGSLTGRWGGMTGETDYAAANEALARLGLWAQRHA
LPCSVKTLVWPTWDSVGMITNFAVTKRYVTPMAADEGVRHWLREFASRDSGEVMFMGAVG
PALTPVQLKGFSPILELPNIADLVTRHHHAGEPLRFRPFRSFATRYRIDPIEVPFAQAFR

FGGRHALPASMLIEHACSVGAWVMPATVEPQRLDALINVAVHLHALPFEGEPEAGIDVLS
EAKGYLLGDLWHVDVHCAHAATQVGLLSLTLVYRAGAAPSVRRITWPAEGLMAKEALPSS
TAAAWNAHVLPAADWQVPLDRSKGIGRIGCAVPADASDLWAIPFPPDLRLPANHLENILR
AVIADNTDSGEALPHEWRIDRMALSGLPPSSADAVVQYTDGRFDVVDALGQVLLELQGVT
LETVSSVVPAAPPATEALAVSPLG

>FAZ97_08880|KEGG|CYP116B210|[Paraburkholderia acidiphila]pacp|BP

MSETPSQSTGRCPFPHAAGEARDAAPRCPVSANAAAFDPFSDGYQQDPPEYVRWAREQEP
VFYSPQLGYWVVTRYADIKAIFRDNLTFSPSIALEKITPTGPEANAVLASYGYAMNRTLV
NEDEPAHMPRRRVLMEPFTPEHLKHHEPMVRRLTREYVDRFIDAGRADLVDEMLWEVPLT
VALHFLGVPEEDMDLLRKYSIAHTVNTWGRPRPEEQVEVAHAVGNFWQFAGKVLDKMRQN
PDGPGWMQYGIRKQQEHPEVVTDSYLHSMMMAGIVAAHETTANATANAMKLLLQHPNAWR
EICEDPSLIPNAVEECLRHNGSVAAWRRLATKDVQIGGIDIPAGSKLLIVTSSANHDERH
FADADLFDIRRDNASDHVTFGYGSHQCMGKNLARMEMQIFLEEFTRRLPHMRLAEQTFTY
VPNTSFRGPEHVFVEWDPAQNPERANAALLGAHVPVRIGEPSAHALSRPVVVERVDAVAE
GVVRLRLVAPDGGALPRWAPGAHIDVECGDTGISRQYSLCSDPADTRAFEIAVLRETQSR
GGSAWMHDTLRTGMRLKIRGPRNHFRLDEEGRRAIFIAGGIGITPVSAMARRARELGIDY
ELHYSGRSRKTMALADELAQLHGERLHLHVSEEGTRNDFAALRVDDHTRVYACGPARMLE
ALEACSEAWPEDALRVEHFAATGGKLDPSQEQAFEVELKDSGLVLNVGADQTVLDALRRA
NIDVQSDCEEGLCGSCEVRVLAGEIDHRDVVLTRAERDTQTRMMTCCSRAKSGRLVLEL

>FAZ97_18265|KEGG|CYP2742A1|[Paraburkholderia acidiphila]pacp|BP

MTINRTANQPDPDRFRSWPPPEETMRCPFAYFHEARVTSPVIRLAEPAAPGVPVYLVTGH
RACVQVLTQPLLFASDLAGVLPGFDKSNVPPPFPDRPTFHDRQVVFFARGEDHKIKRGWA
MELVRRSRLERLRPLIVEEADRLIDAFTDAGHCDFQEQFSHPLPLRVLLHALDLPETALP
VIRRMSIAIATTDVDPNLGEEERVAKANAFGELFTMIRGLLEERHIHPGSDYISELVKAQ
VARDGELDVNTLSIHLQGVMFGGDHAVGAHLCHLAAALAGDPELQARLRAEPGLIPRFVL
ETFRLEAPIPWLFRVCTADTELEGVSIEAGAVVIAATSAANRDPERFPDPGHLSLERDNV
GREHLSLGRGAHRCIGEPLAYLVAEATVTRLLERLDAIRIDPARSDLRARASLQFRCPER

VHLLFEKR

>FAZ97_23870|KEGG|CYP1220A5|[Paraburkholderia acidiphila]pacp|BP

MSTASTGQPLRQIADLPCPRGLPLLGNLLQLSPPRLHLILEQWAAELGAPYRFQVGGIPI
TIWTDTELIQQIMRERPHRFRRYQPIEAVFEEMGFNGLFSAEGVAWEPQRKLVMQALSIP
NIKAFYPTLAEITQRLHTRWQRAAERGETVEMTDDLKRFTVDVTSALAFGEDPRTLERDH
GVIQEHLERILPGVMRRTNALFPYWRYVKLPSDRKLERSLAEVHRYVNAMMARARERMRD
EPSETPRNLLEAMLAQQQAPGGAASISDAQIRANVLTLLIAGEDTTADSIAWTLPYLATD
TTLQQHLGDEASRVLGHSTVCPDYATLKDLDIFEAICIEATRLRPVASVHSFEPLEDVVV
DNVALPKGTRMFFVARPAMLDAKNFADPQRYDPWRWMKRAEREADDGKAHDVRAYLQFGA
GPRVCPGRHLATVEMRLALSMLMKSFTIELAIDPAKIQEVQAFTMVPDTMPVRLKPREGA
VLA

>FAZ97_31575|KEGG|CYP133C10|[Paraburkholderia acidiphila]pacp|BP

MQFSDFASPAFFANPYPLYEKIRAAGPILPVGPKAFVTGRFAIVDALLRDPRMGKTYLQS
VAARYGDEAPARPVFQSLSRTLLMMNPPAHTRLRALLMKTFNARSIEKLTSIVEATTHEL
LERAASRAEFDLVSDYALPLPVQIICRLLDVSVADATTLGDAASQLVAAFDLAPLDEDGL
RAADEAALSLECYFHGVVEQRRAQPGDDLISAMLSVEDEGSRLTDEEVVSNAVLLFIAGH
ETTSNMIGNMMIALFRHPQQMEALKHEPKLLPRAIAECMRYDGAVQMLVRTALEDITLGD
ATLARGSVVFMLIGSANRDPAAFPAPDTLDFARSTGTPAVAFGAGIHYCLGARLAALELD
VAMRALLARFPEIRPVDLDALVWHRRNNLRGVTSLRVRMR

>FAZ97_32485|KEGG|CYP1220A17|[Paraburkholderia acidiphila]pacp|BP

MDAAASSAQRLRQVRDLPCPRGLPLIGNLHQLNAPRLHRVLEHWAQELGTPYRFQICGMP
VTVWSDAELSQSVMRERPHRYRRYASLEPVLAEIGCNGVFSAEGAAWQPQRRLVMQALSI
PHIRGFYPTLQAITARLHDRWQRAAERGDVVEMTDDLKRYTVDVTSALAFGEDPRTLEQE
RGVIQEHLAQIFPTLMHRVNAPFPYWRYVRLPRDRRTDRALAEVHRTIRGMMARSRERMR

DAPSSIVRAPRHLLEAMLALRDAPGSAPGSEVTDDIIAANVLTLLLAGEDTTANALAWSL
FYLAADEPLQQRLANEARAVLGEHDVCPDYGLVKDLDLFEAVCSETLRFRPVAAIMAFEP
VENVEVGGVALPAGSKMFFLTRPPMLDSRHYAQPERFDPDRWLRGHADADARVHDPRAWV
QFGAGPRVCPGRHLAAMEMRLVLSTLMNRFKVRLAVDPATIEEITAFAMVPNRMPVRLEC
RPTVQ

>FAZ98_08660|KEGG|CYP116B299|[Paraburkholderia acidisoli]pacs|BP

MSQTPSQSGQNAAGLGAAGSGAAGPGALSRCPFPHGAATAAVTPAAAGCPVSANAAAFDP
FSDGYQQDPPEYVRWAREQEPVFYSPQLGYWVVTRYDDIKAIFRDNLTFSPSIALEKITP
TGPEANAVLASYGFALNRTLVNEDEPAHMPRRRALMEPFTPEHLKHHEPMVRKLTREYVD
RFIDDGRADLVDQMLWEVPLTVALHFLGVPEEDMDLLRKYSIAHTVNTWGRPRPEEQVAV
AHAVGNFWQFAGKVLDKMRQNPDGPGWMQYGIRKQKDHPDVVTDSYLHSMMMAGIVAAHE
TTANATANAMKLLLQHPHAWREICEDPSLIPNAVEECLRHNGSVAAWRRLATKDVQIGGI
DIPAGSKLLIVTSSANHDERHFADADLFDIRRDNASDHVTFGYGSHQCMGKNLARMEMQI
FLEEFTRRLPHMRLAEQTFSYVPNTSFRGPEHVLVEWDPAQNPERVNAAVRDAQVPVRIG
EPSAHALSRPVVVERVEVPADGIVHLRLVAPDGATLPRWAPGAHIDVECGDTGLSRQYSL
CGDPADTRAFEIAVLREAQSRGGSAWVHDTLRVGARLKIRGPRNHFRLDEEGRRAIFIAG
GIGITPVSAMARRARELGIDYELHYSGRSRATMAFVDELTQLHGERFHLHVSDEGSRNDF
AALNVDDATRVYACGPACMLEALESCSANWPDDALRVEHFAVTRGTLDPAHERAFEVELK
DSGLVLNVAADQTVLDALRRANIDVQSDCEEGLCGSCEVRVLAGEIDHRDVVLTRAERDT
QTRMMTCCSRAKCGRLVLEL

>FAZ98_19255|KEGG|CYP1220A15|[Paraburkholderia acidisoli]pacs|BP

MSTASPARATRQVADLPCPRGLPVIGNLLQLAPPRLHQILEQWAAELGTPYRFQIGNIPI
TIWTDTELIQQIMRERPHRYRRYQPVEAVFDEMGFNGLFSAEGAAWEPQRKLVMQALSIP
NIKAFYPTLAEITQRLHARWLRAAQRGEIVEMTEDLKRFTVDVTSALAFGEDPRTLERDH
GVIQEHLERILPGVMGRINALFPYWRYVKLPRDRALDRSLEEVHRYVRAMLERARERLRD
DPPETPPDAPAMPRNLLEAMLMQQAAGAPVTDAQICANALTLLLAGEDTTADSIAWTLPY
LAADAGLQRHLGEQAQRVLGAATVCPDYATLKDLDMFEAICIEATRLRPVASVHSFEPLE
DVVLDGVALPKGTRMFFVARPAMLDAKHFADPLRYDPWRWMKREAGTGAEEKAHDVRAYL
QFGAGARVCPGRHLATVEMRLALSMLMKSFSVELAIDEAQIREVQAFTMVPERMPVRLRL

RDNTPAAPL

>KZJ38_02460|KEGG|CYP201E1|[Paraburkholderia edwinii]pew|BP

MQAPSPNTLPSPADIDARRAPLFTPPMPELPARRVSATGFVHAMRNDGLRLWTRDAYEAD
YVEERLFHQKRVLVNKPDMIHRVLVENAANYPRTDYAVRLIRPIGRYSVLLATGEQWKHQ
RRTIAPTLAPKMLPVLARHVAACTEDEVHALAALDGAPVDLLPVMRLLALKIAARSMFSL
EIREFGPEVRAAVQEFMQRHTRASLAELVLPYWVPTPRDIPRNGFRRRWHALVERIIDAR
ERQPAADGPRDLFDALMAARDPETGAAFSRVELADQVGTMIVAGHEPTGLTLLWALYLLA
NTPAAQAALADEVRDVDLSPERAGDALKALPYTRAVIDETLRLYPPAWVILRHCVEPDAL
EGLAVTRGTQMMISPWVLHHHRGFWQNPDAFDPTRFLRGAPAPERFTYLPFGTGPRVCIG
AQFTLAETTLALARLVQAFDAQLASDEPVRPIGRATTIPDRPLWFRLKPRHARR

>KZJ38_17180|KEGG|CYP133B8|[Paraburkholderia edwinii]pew|BP

MNLADFATPSFLANPYPVYDALREEGPLVPVGPNQFVSGHYDIVDKLLHDRRMIHNFESS
VRKRYGEEGAQQPVLLGYSRMFLLMNPPVHTRLRGLMMKAFNARQVESMRDIARTSADRL
IDSFESDGSVDIMAAYAMPFPIEIICRMLDVPVEHAQKLADAIQHVAKMLDMTPVDDAQL
AVSNDAYRTLDTYFSEVIEARRRQPGTDLISMLLKVEEQGETLTHDEIVANVVLLYLGGH
ETTSHMIGNALISLQRHPQQFDALRKDRAKLPNAIMECLRYEGSAQLSGRTASEDMDIAG
VPVARNSIVYLSLGAANRDPAKFDHPDQLDIGRDLPRVLTFGGGVHHCLGYRLALLELET
ALGALLTRLPKLTVDGLDNLHWNGRSSLRGVETLRVHW

>KZJ38_22295|KEGG|CYP133B9|[Paraburkholderia edwinii]pew|BP

MKLADFATPEFFADPYPLYERLRNEGPLLTVGPKKMITGRYGVIEELFLNRQIGRNYLPS
VRARYGEEGLRQPALLGFSRMLSQLNPPAHTHLRGLTIKAFNARQIGPLRDTAQAMAHRL
IDSFAPRSHGTVDLIAAYAQPLPIAIISGMMDVPFEHSSHFSAALKQMFRLLEPMPLDSE

ELAKTNDAYLLLARYFGEVVDARRKRPGTDLVSLLISAEENGETLTHDEIVANVIMLYLG
GHETTANMIGNTLIALQRHPQQFDALKSDPAKLPNAILECLRYDGSVQLTARMARADTEI
NGIRIPRDTHVFMALGAANRDPDKFYKPDQLDIERESPRPLTFGAGIHHCLGYRLALLEL
EVALGVLLSRLPSLALTGLEDLRWLPTSAIRGVEQLIVRW

>KZJ38_26430|KEGG|CYP1220A18|[Paraburkholderia edwinii]pew|BP

MSTTASGNTNRTDGTRLRQLTDLPSPKGLPIIGNMHQVSLRTFHLNLERWGRELGTPYMF
RMPGQQIVVLGDTDHVQGISRERPHRYRRFKPVETVLAEFGANGLFSAEGVAWEPQRRLV
MQALSIPHIRAVYPTLAEITGRLYTRWMRAAQEQRTVDMVEEMKRYSVDVTSALAFGEDP
RTLERDTNVIQEHLKLVMPALMSRTSAPFPYWHYIKLPRDRRLDRALIEIHRYVRRMMER
ARERMHDEPSDMPRNLLEAMLAMRDTPDSGITDDQVSANVLTMLLAGEDTTANSLAWSTM
FIATDAPLQARLAKDARAVLGTEPVCTDYARLKELDVFEAVCTETGRFKPVAALSSFEPL
EDVTMGDVLVPAGTFMFFLNRPAMHNPANFVNPERFDPDRWLHARDPARGAHEQRAYLQF
GAGPRVCPGRHLAGVEMRAALSMLAANFEAKLAVDASEIEEINDFIMKPSKMPINLTIRP
QAA

>KZJ38_27455|KEGG|CYP1220A19|[Paraburkholderia edwinii]pew|BP

MPILGNLFQMAPAKLHLTLEQWAKQFGSPYRVQLGTIPVTVWTQADLFQTVMRERPHLYR
RFAPMESVMAELGGNGLFSAEGAAWEPQRRLVMRALSFSNIKGFFPALEAITGRFLVRLQ
QAEKEQRTLEMTKELKRYTVDVTSTLAFGEDPHTLEREQGIIQEQLALVTPALMRRITAI
FPYWHYVTLAEDRRLARAMVEVHSYIRKMIERARERMREAPDAPPRNLLEAMLAARNEPD
SKVTDEQISANVLTLLVAGEDTTANAIAWALMYVAADTRLQNRLFEQARMAFGESAICPD
YDALKHLDLCEAVCNEALRLRPVAAIQTFEPLTDVYIGGVAIPARTRMLFLTRPATRDPL
NFAHPETFDPDRWKHPRDPSEGAHELKAYLTFGAGPRVCPGRYLAAVEMRLVISMLTRHF
EIELAIDPAKIEEVSAFTMVPNTMPVRLRPRG

>KZJ38_30935|KEGG|CYP133B6|[Paraburkholderia edwinii]pew|BP

MKLADLTTPEFLDNPYPFYEKLRAEGPLVRIGPKAVITGRYDVIEAILHDRRFGKNYPES
IRMRYGDDAMHMRLFQGFSRMFLVMNPPSHTRQRALIVKAFDARHVDAMKDVSHRIAHEL
IGAFEKAGTANLMAQFAFPLPLRMIGQMLDIPAEDTTQLGEAVSAIAKVIDVAPMNADDL
GRACAGYEDLEHYFHCVVEARRRRPGNDLISMLLAVEEEGETLSQDDIVSNTILLLLAGH
ETTSNMIGNALIALHRHPEQLALLKREPSLMPAAVLECMRYDGSAQTTIRSALEDVEIAG
VDVPCGTSVLLMFGSANRDPAQYADPDLLDIERNEARLQSFGSGIHHCLGYRMALVQLET
ALGALLERLPGLELTGLDNLNWNRRGNLRGVTSLVARW

>KZJ38_31880|KEGG|CYP133B7|[Paraburkholderia edwinii]pew|BP

MEFADFKTASFFDNPYPVYASLRAEGPIVSIAPNMMMTGHYDIVDALLHDRRIGKAYLES
VRARYGEAAAEQPVFQNVSRTMFSINPPAHTSLRSLMMKAFNARQIESIREIARATANEL
IDAIAKTDGADLMASYASPLPIRIICRMLDVPVEDAGALANATSEMVRVLDAVPIPLDEL
AGPNEACATLERYFADVIEARRSKPGDDLISLLLRIEDSGVKMTEEDIIANVILLFNAGH
ETTSNMLGNSLVALHRHPEQLHALRQHPDRTAQAVFECARYDTSVQSTVRTALEDVEIGG
TTIPRGTIIMTMLGSANRDPAKFSDPDRLDISRDDARLISFGAGIHHCLGYRLALLQLET
AMKVLLERLPRLHLTNLDSLQWRRHGNLRGVKALAAHW

>KZJ38_32695|KEGG|CYP133C14|[Paraburkholderia edwinii]pew|BP

MNAPAFQSPAFFDDPYQYYEVLREQGPLVALTPDGKRFATARYGIVTALLDDRRMGRRYM
PGVVRRYGEERASQPTFQALSRMFITMDPPEHTPLRALLMQAFNARQVERLREVTRATAE
RLIGALAHDKPFDLVSGYALPLPVQVIAGVLGLPFEEAAAIGLKMERFARAFESIAMDDA
TLAAANAATLELEHYFYGVLEARRTQPGNDLISMLISVEAEGRRLTNDEIVSNVLLLFFA
GHETTSNMIGNAVVSLHRHPEQLKKLREQPQLLPKAIIECMRYESSVQTALRTTLEDGVE
IEGIALPRGSIVTLMLGGANRDPAQFHDPNQLILDRTENDRRILSFGGGLHYCLGARLAQ
LELHIALETLLTRLPDLRVLNLHELHWRHHNTLRGVEALWCEHPAKAATAAARAVV

>KZJ38_34845|KEGG|CYP133J1|[Paraburkholderia edwinii]pew|BP

MQFSDFSNAAFFTNPYPLYDEMRAAGPLVKLSPGLYVTGQYSVVHDLLFDRRFGKGIEAS

VKARYGENAVEQPIFRMARHMLAGLNPPVHTRVRALLMKSFTARQLERFRQASHDISHRL
ADALIASDEPDVIRDFAFPLPMQIICTIMNIPLEDAPMIKQLTDNASSVLDVNLMSPEQL
QKANDSVLELHRYFTVLFEARRRNPGDDLISQMVAAEENGDVMTNEELFANVLLLFVAGH
ETTTNIIGNSLVSLHRHPEQLILARSNPAMLPNVIMECMRHETSVQSTMRVALEDAEVEG
VQIARGDVVFVWLGSAHRDPGMFVEPDRLKIDRSFKDVKILSFGGGLHFCLGARLASMEI
EAALGTLFSRLPDMRITNLDDLHWHKRSQLRGVESLQIAPS

>RBRH_02574|KEGG|CYP1464A3|[Mycetohabitans rhizoxinica]brh|BP

MNHKQDTIALAQADAGMEAQPSQVAVNDLKIERNDQPANVARPVAKNLKMKDGFPVMPGK
IPLLGHVHKISQNALSELRQAEAVCGPMFWTYFGTKLPVLQIMDEDGLAILQNKYTDNSY
LREQMPVITGEAMNAFDGQRHRNARRASMDAFTPKGLTRARVGQFVVETIEQRLKRWTSE
RKLAIFPETKDIALEVVFRIMGIETHELEQWRHQYEEFFLGMIPLKINLPGFPAWRCRKA
RGWLEQRVSQIVATVRANNDRDSLVGAMIFGRDDNGNGMSEVELVHNILGLGFAGSETTA
AVMAWSALMLSQHPHVWQQLCQQVAGLDSVPYTHEELVKQVPLAEAIFRETLRLYPPAPF
EMRKVHTEFEMMGHTVPAGAMVGVSLLHVSRHPERYPDPDSWRPERWLGMDRAPNQVESC
QFGGGPHTCLGRHVAALEITLFIAMLAREMGPKGISPRLVGKLPPPTYLPFLRPSNKAFL
DLTGR

>U875_08260|KEGG|CYP116B197|[Pandoraea pnomenusa]ppk|BP

MSQTSSPTADTGACPFHQATAAKAPNGCPISARAAEFDPFEDGYQQDPPEYVRWAREQEP
VFYSPKLGYWVVTRYDDIKAIFRDNITFSPSIALEKITPTGPEANAVLASYGFALNRTLV
NEDEPAHMPRRRVLMEPFTPEHLKHHEPLVRQLAREYVDRFVNDGRADLVDQMLWEVPLT
VALHFLGVPEEDMDKLREYSIAHTVNTWGRPKPEEQVAVAHAVGNFWQLAGKILDKMRQN
PDGPGWMQYGIRKQKEHPEVVTDSYLHSMMMAGIVAAHETTANATANAMKLLLQHPDVWR
EICEDPSLIPNAVEECLRHNGSVAAWRRLATKDVNIGGVDIAAGAKLLIVTSSANHDEAH
FADADLFDIRRENASDQLTFGYGSHQCMGKNLARMEMQIFLEEFTRRLPHMKLAEQKFTY
VPNTSFRGPEHLWVEWDPAQNPELRDPSILARHSPVRIGEPSGHSVTRPVVVESVSPVAD
GIVKLRLVAPDGRAMPRWAPGSHIDVECGDTGLSRQYSLCGDPADVGAFEIAVLHEPQGR
GGSNWMHTQVKVGDRLRVRGPRNHFRLDETTPRAIFVAGGIGVTPIAAMARRAKALGMDY
EIHYSGRSRRTMALVDELRALHGERLHLWVKDEGQRADYVALLATPQAGTQVYACGPVRL
LDALAECCAYWPDDTLRVEHFQSQLGTLDPEKEHAFEVTLKDSGITLTVPADQTLLTALR

AANIDVQSDCEEGLCGSCEVQVLEGDVDHRDVVLTRAERDANTKMMSCCSRACGKKLVLA
L

>RO07_12270|KEGG|CYP212A13|[Pandoraea pulmonicola]ppul|BP

MSDDLLGALAAWQREYGDVVHLRIWPEHTVVVTDPSLVRELLITHHNALIRWERGVRVMS
QLHGHSVLIAEGGAWHQKRQALQPDFLPKAVQAFAPNIVGATAQALAAWPEHDARWPIER
ALTSLTMDVIIRQVFSEETDDDIRVAEDAMRIASEAANAEFYWPVSWPDWAPWKRKKRRA
LRILKGLVDRHVQMRLATPDDAWPDDLLTRLLRLHRGNAQAWPLEAARDECMTTFLAGHE
TTAATLTWWAWCMASNPTMQAAAREEVTRVLSGGLPISASRQSLPYLAQTLDETLRLYPT
APTLISRRAIAPVTLGDWTFPARTLFMLPVQLMQHDARWFEAPGEFRPERFAAGAAQIPR
GAHMPFGAGPRVCLGQHLALTKALLIAAMILQRFELSVPAGAQPPQPRLNVTLRPAQPLR
LEIAQRRR

>SG18_23715|KEGG|CYP116B192|[Pandoraea apista]papi|BP

MSQTISPTPDTAAASAGGCPFHQTAPVTAPNGCPVSARAADFDPFEDGYQQDPPEYVRWA
REQEPVFYSPKLGYWVVTRYDDIKAIFRDNITFSPSIALEKITPTGPEANAVLASYGFAL
NRTLVNEDEPAHMPRRRVLMEPFTPEHLKHHEPLVRQLAREYVDRFVNDGRADLVDQMLW
EVPLTVALHFLGVPEEDMDKLREYSIAHTVNTWGRPKPEEQVAVAHAVGNFWQLAGKILD
KMRQNPDGPGWMQYGIRKQQEHPEVVTDSYLHSMMMAGIVAAHETTANATANAMKLLLQH
PDVWREICEDPSLIPNAVEECLRHNGSVAAWRRLATKDVTIGGIDIPAGSKLLIVTSSAN
HDEAHFADADLFDIRRENASDQLTFGYGSHQCMGKNLARMEMQIFLEEFTRRLPHMKLAE
QSFTYVPNTSFRGPEHVWVEWDPAQNPERRDPAVLTRHSPVRIGEPSGHSVTRPVVVESV
TPAAEGIVKLRLVAPDGRTMPRWAPGSHIDIECGDTGLSRQYSLCGDPSDAHAFEIAVLH
EPEGRGGSNWVHTQVKIGDRLRIRGPRNHFRLDESISRAIFVAGGIGVTPIAAMARRAKT
LGIDYEVHYSGRSRRTMALLDELQTLHGDRLHLWVKDEGQRADYASLLATPQNGTQVYAC
GPVRMLDGLAQCCAHWPEDTLRVEHFQSQLGTLDPEKEHAFEVTLKDSGITVTVPADQTL
LTALRAANIDVQSDCEEGLCGSCEVQVLEGDVDHRDVVLTRAERDANTKMMSCCSRACGK

KLVLAL

>AT302_08215|KEGG|CYP212A15|[Pandoraea norimbergensis]pnr|BP

MNTRTPPQCPFHGDAAAASEATAPTLHPPGVWPPGPSAGITGWGLLRQMSGDLLGTLAQW
KRTYGDVVYVRFWPEHAVVVTDPQLVRELLVTHHHSLIRWERGVAVMSQLHGHSVLIAEG
EAWHRKRQTLQPYFLPKSVQTFVPHITAATARALDAWPQRDAHWSIESALTSLTMDVIMR
QVFSGEIGDEARMAERALRVASEAADAEFYWPASWPDWMPWKREKRHAVRGLKGLIDRHV
NARLAMPPDAWPDDLLSRLLRLHQEDPQTWSLQAVRDECMTTFLAGHETTAGTLTWWAWC
MASNPAMQEAAREEVIRVLGSAGEIVPTAETRPALRYLSQTIDETLRLYPAAPTLLSRRA
TRPITLGPWQFPARTLFMLPVQLLQHDARSFAEPTAFRPERFASDAPAPPRGTHMPFGAG
PRVCLGQHLATTEIIVIAAMILQRFTLSVPEGMEAPKPRMHVTLRPETALHLGLTAIPSI
DV

>DRB87_05310|KEGG|CYP229G2|[Pandoraea sp.]pand|BP
MSAATADRLTVPPPANALAAVVHPNPYPYYQSLAARRPFDFDASLGMWVAASPQAMAAVL
RHPACGVRPAGATVPAALANGAAGDWFGTLVRMNDGAAHAAMKPWLKARLATLHADGATL
DRLRNASQAACADAYLAQGQTPSARLDDFVFRQPIYALAAWLGVPPSQWVDVYDDVRTLV
AAMAESTQSSAHAETLARGHSAATTLRHRAMRWLDNDAAPGTWLRDTARSAARDEAVDYA
ALTANVVGLFTQTHDACAGLVANSLRRLAQHASPGAVPALNSAQSLPANLTVAITAIGEV
IRHDPPVQNTRRFLHAPAIIHERTLARGDAILVVLAAAPARAAPDDVAWTFGHGAHTCPG
RSPASTIAAIGVQTILDSDVDLAAIATGSTYHPLGNIRIARFHSGAAAA

>PI93_012205|KEGG|CYP116B196|[Pandoraea fibrosis]pfib|BP

MSQTISPTPDTTAASAGGCPFHQTTPAKAPNGCPVSARAAEFDPFEDGYQQDPPEYVRWA
RDQEPVFYSPKLGYWVVTRYDDIKAIFRDNITFSPSIALEKITPTGPEANAVLASYGFAL
NRTLVNEDEPAHMPRRRVLMEPFTPEHLKHHEPLVRQLAREYVDRFINDGRADLVDQMLW
EVPLTVALHFLGVPEEDMDKLREYSIAHTVNTWGRPKPEEQVAVAHAVGNFWQLAGKILD
KMRQNPDGPGWMQYGIRKQKDYPEVVTDSYLHSMMMAGIVAAHETTANATANAMKLLLQH
PDVWREICEDPSLIPNAVEECLRHNGSVAAWRRLATKDVNIGGIDIAAGSKLLIVTSSAN
HDEAHFADADLFDIRRENASDQLTFGYGSHQCMGKNLARMEMQIFLEEFTRRLPHMKLAE
QSFTYVPNTSFRGPEHVWVEWDPAQNPERHDATVLTRQSPVRIGEPSGHSVTRPVVVESV

TPVAEGIVKLRLVAPDGRAMPRWAPGSHIDIECGETGLSRQYSLCGDPLDASAFEIAVLH
EPEGRGGSNWVHTQVNIGDRLRIRGPRNHFRLDETTSRAIFVAGGIGITPIAAMARRAKA
LGIDYDIHYSGRSRRTMALIDELQTLHGERLHLWVKDEGQRADYARLLATPQEGTQVYAC
GPVRMLDSLAQCCTHWPEDTLRVEHFQSQLGTLDPEKEHAFEVTLKDSGITVTVPADQTL
LTALRAANIDVQSDCEEGLCGSCEVQVLEGDVDHRDVVLTRTERDANTKMMSCCSRACGK
KLVLAL

>PI93_021335|KEGG|CYP125U1|[Pandoraea fibrosis]pfib|BP

MKIGDVNLADPDAFFTAVPYDYFAMMRREAPVSWVPRVGDDGFWSITRYADIADVEKRPE
VFSSRANILPLPIPEYTLARDIDHNIILSDPPRHGFLRRVIMSAFTQKAILGINERVRVL
TTRIIDSVIESGECNLVDVAAYVPIEIVAEMLGVPESDRARLFAWADATVGSADPDATRH
ISSKQAAEEMFEYASRLGAERRRNPQGDIFSTIAAAKENGERLSDVDLGCFFLLLATAGT
ETTRTQILQGSLALIEHPDQRRKLIEDRSKLPNAIEEMLRWSTPAMCFGRKALVDTQVGG
QAIKAGQQVVLWYCSGSRDESVFANPDVFDVDRHNAREHQAFGVRGSIHQCLGSMLARSE
LVAVFDALLERMPDMSLAGPVVRLRSNFANGIKQMPVRFTPGIVLGDGPSARWYAAENEG
CPAHSSPSATRCPVTSHGE

>SBC2_20620|KEGG|CYP195A25|[Caballeronia sp.]caba|BP

MTPTERALDFDLRKLDAAFYADPYPVYDALRATDPIKTLPDGSLFLTRYRDVQAVYRDPK
TFSSDKTVEFGPKYGVHTPLFAHHTTSLVFNDPPRHTRVRKLIAGALTARAIAAMEPGLV
RLVDGLLDQAAARGEIDLIDDFAAAIPVEVIGNLLDMPHDERAPLRDWSLAILGALEPAL
NPEQEARGNRAVTDFIDYLVTLVARRRAHSGDPQHDVLTRLIQSEADGERLDETELLHNC
IFILNAGHETTTNLIGNGIVLLAQWPEQRAQLLADPTLIESAIEECLRYESSNQLGNRIA
TADTHIAGVAVARGTPVTLCIGAANRDPEQFEAADCFDIAREPNRHLAFGFGIHQCAGLS
LARLEARIAIGRFIARFPNYGLAGEPLRASRARFRGFVKAPVRVG

>SBC2_72680|KEGG|CYP289A14|[Caballeronia sp.]caba|BP

MSMNEKPTNDWDPRSEAVTHDQVAAYDEMRHRCPVAHSDYLNLSLFRHDDVMRVLNDPET
FSSAVSSYLSVPNGMDPPEHTQYRTIVEPYFSPQRMAVFEPVCRDIAATLVNGLSRGREL

EFNAQFAQDFAMNIQCAFLGWPQELRAPLLHWIRKNREATLTGDRIALDRVAVEFDTYIK
GLLAQRRQSGAEAQDDITTSLLRERIGDRLLHDDEIVSVLRNWTVGELGTIAACIGILAH
YLAEHPDVQQRLREQPSVLPKAIDEILRIRAPLIANRRISTKSVKIGGHEVPVGQRITLI
WASSNRDETVFGDPDEFRLDRDPASNLLYGSGIHVCPGAPLARLELRVGMEELLERTNWI
ALVPGSPLTKAVYPAAGFSALPLRIE

>BRPE64_DCDS12190|KEGG|CYP116B12|[Caballeronia insecticola]buo|BP

MTDAAGENRAPQCPFHDVAGAQALAPNGCPVSARAAEFDPFGDGYSQDPPEYVRWAREQE
PVFWSPQLGYWVVTRYDDIKAIFRDNLTFSPSIALEKITPTGPEANAVLASYGFALNRTL
VNEDEPAHMPRRRALMDPFTPEALAHHEPMVRALAREYVDRFIDDGRADLVDQMLWEVPL
TVALHFLGVPEEDMDTLRLYSIAHTVNTWGRPKPEEQVEVAHAVGKFWQFAGKVLDKMRE
DPSGAGWMQYGIRKQKDMPEVVTDSYLHSMMMAGIVAAHETTANATANALKLLLQHPEAW
RDICEDPSLIPNAVEECLRHNGSVAAWRRLATKDVTIGGIRVAAGSKLLIVTSSANHDQH
QFADADLFDIRRENASDQLTFGYGAHQCMGKNLARMEMQIFLDELTRRLPHMRLAEQRFT
YVPNTSFRGPEHLYVEWDPAANPERADPSVREPRAVVRIGEPSSHAASRAVTVASVEACA
DRIVRVRLVPADGRALPRWTAGSHVDVVCGDSGISRQYSLCGDPADAGALEIAVLRETAS
RGGSAWVHENVKPGAQWHIRGPRNHFRLDETAKRLVLIAGGIGITPISAMARRARALGID
YAVHYSGRARTSMALLDELRALHGERLFTYISDEGARNDFAALDVNEDTQVYACGPARML
DALTQASARWPEDALKIEHFESKLGTLDPSKEHAFDVELKDSGLVLTVPADQTLLTTLRR
ANIDVQSDCEEGLCGSCEVRVLDGEIDHRDVVLTKGERQSNGRMMACCSRAKGKRLVLAL

>BRPE64_ECDS01410|KEGG|CYP1112A12|[Caballeronia insecticola]buo|BP

MLVDPYPAYRALRELGEVVYLTHSKLYALTGYDVIKDALADPTRFSSAQGVGFNDGVNHA
LQGTSLASDPPEHTQLRAALSENLTPRALRGLGEQIKANAQALVAHLVEQQSFDAIDDIA
RRFPIGVVADLVGFQGDARANMLRWGQAAMEVIGPMNRRTAENFPIAGELYGYCSQITQD
ALTPGSVGWGVFEAERKGIVPEGAARHIIHQYLGAGLDTTLAAIGNTVALFGRFPEEFAK
VRDDLSLVPSAFNEVLRYYSPVHVWSRVATQEVKVGNVKIPDGSRIAIILGAGNHDARHY
RDPEVFDVTRNPIDHLSFGYGPHGCAGQGLARMEGAAIIEALATQIKSFRIGEVARAPSN
VTVSFDTVPVLELEAV

>HKT17_08605|KEGG|CYP153A108|[Limnobacter sp.]limn|BP

MTTKSSMSGTIQTKVINATAKIVPMNLQIRALKVLMKAKRKTIGPKTPMLHFVESPLPDV
TTLSIEEIDASNPFLYRQGKCSAYFKRLRDEAPVHYQKNSPFGPYWSITRFDDILYVDKN
HQLFSSEPQIVLGDPPDGLSVEMFIAMDPPRHDKQRRAVQGVVAPQNLREIEGLIRSRTG
EVLDSLPLNEPFNWVPKVSKELTARMLATLLDFPYEERHKLVGWSDRLSGASSATGGEST
NEDVMFDDAADMAWSFSTLWRNKEADRAAGEKVGFDLISMMQSNEDTKDLINRPMEFIGN
LALLIVGGNDTTRNSMSGGVLALNRFPEEFVKLKANPGLIPNMVSEIIRWQTPLAHMRRV
ATQDVELHGQTIKKGDRVVMWYASGNRDERKFENPDQFIIDRHDARNHMSFGYGVHRCMG
NRLAELQLRILWEELLKRFDDIEVVGEPELVQSNFVRGYSKLMVKLTEKSR

>FNU76_17870|KEGG|CYP1202B1|[Chitinimonas arctica]cari|BP

MPTSATLPGSPPPLRAPKPAGQLLLGHLKPYRADMLGFLSRSRAELGDIFRIRLGLRHLH
VLGHPDMAEQVLIKDKARFGKLAELAELGSKREIGLGLVLGKGLVTNYGESWRRQRQMIQ
PMFHKAQIAAMADAMTAAGDRLLAHWRTSYQPGQTVDVAQEMMRVTVDVVSQTMFGADLV
DKACFVGDRFPLLLRYAFSSISNPFSPPPSWPTARNRAFHKAMQELDELLYGLIDQRLQR
GEPRGDLLDMLLVARDHESETGMSVRQLRDEVATIFGAGHETTANALTWTWYLLSRHPEA
KRKLKEELASVLGGRTPTFADLPRLVYTRAVFEESMRLFSPAPALPRAVLQDTTVAGYGL
RRGELVMVVSDLLHHHPAFWREPARFDPERFLPGAVPLQAHRCAYLPFGAGPRVCIGNHF
ALMEGQLLLAQIAQRYDLQLVSGHPVEREVAVTMRPRYGMPMKLVEIT

>BP2405|KEGG|CYP240A1|[Bordetella pertussis]bpe|BP

MIADTARQHRGDSMQPADPLEAVAHPDPYPYYAALARERPFYHDDRLGLWVAAGPQAIRA
VLTCPAARVRPPGEPVPAALGAGPAAQMFGRFIRMNDGAVHERLKPMLTAYLTQRTAADL
AEPAWPAIGNDPAQVDRYLYQAPVHAQACLMGLPDEVAASCAREIEAFMAACRPGADAAA
VARADQAAQALQARMLAHLRAARGDAALGVLRRLALAGGVEADALAANLAGLLLQSCEAG
AGLLDNALVHAGRLSPAAAPDLLHTCVEIVTHVARHDPPLHNTRRFLAAPATLLGQSAPA
GAGILVVLAAAHALAEGAWPWTFGAERHACPGRTPALLHAAQALAHALRHGVDAPALARC
VRYRPLPNARVPRFHFPPGDTP

>BPTD_2362|KEGG|CYP240A1|[Bordetella pertussis]bpc|BP

MIADTARQHRGDSMQPADPLEAVAHPDPYPYYAALARERPFYHDDRLGLWVAAGPQAIRA
VLTCPAARVRPPGEPVPAALGAGPAAQMFGRFIRMNDGAVHERLKPMLTAYLTQRTAADL
AEPAWPAIGNDPAQVDRYLYQAPVHAQACLMGLPDEVAASCAREIEAFMAACRPGADAAA
VARADQAAQALQARMLAHLRAARGDAALGVLRRLALAGGVEADALAANLAGLLLQSCEAG
AGLLDNALVHAGRLSPAAAPDLLHTCVEIVTHVARHDPPLHNTRRFLAAPATLLGQSAPA
GAGILVVLAAAHALAEGAWPWTFGAERHACPGRTPALLHAAQALAHALRHGVDAPALARC
VRYRPLPNARVPRFHFPPGDTP

>BN118_2610|KEGG|CYP240A1|[Bordetella pertussis]bper|BP

MIADTARQHRGDSMQPADPLEAVAHPDPYPYYAALARERPFYHDDRLGLWVAAGPQAIRA
VLTCPAARVRPPGEPVPAALGAGPAAQMFGRFIRMNDGAVHERLKPMLTAYLTQRTAADL
AEPAWPAIGNDPAQVDRYLYQAPVHAQACLMGLPDEVAASCAREIEAFMAACRPGADAAA
VARADQAAQALQARMLAHLRAARGDAALGVLRRLALAGGVEADALAANLAGLLLQSCEAG
AGLLDNALVHAGRLSPAAAPDLLHTCVEIVTHVARHDPPLHNTRRFLAAPATLLGQSAPA
GAGILVVLAAAHALAEGAWPWTFGAERHACPGRTPALLHAAQALAHALRHGVDAPALARR
VRYRPLPNARVPRFHFPPGDTP

>B1917_2316|KEGG|CYP240A1|[Bordetella pertussis]bpet|BP

MIADTARQHRGDSMQPADPLEAVAHPDPYPYYAALARERPFYHDDRLGLWVAAGPQAIRA
VLTCPAARVRPPGEPVPAALGAGPAAQMFGRFIRMNDGAVHERLKPMLTAYLTQRTAADL
AEPAWPAIGNDPAQVDRYLYQAPVHAQACLMGLPDEVAASCAREIEAFMAACRPGADAAA
VARADQAAQALQARMLAHLRAARGDAALGVLRRLALAGGVEADALAANLAGLLLQSCEAG
AGLLDNALVHAGRLSPAAAPDLLHTCVEIVTHVARHDPPLHNTRRFLAAPATLLGQSAPA
GAGILVVLAAAHALAEGAWPWTFGAERHACPGRTPALLHAAQALAHALRHGVDAPALARC
VRYRPLPNARVPRFHFPPGDTP

>Q425_22250|KEGG|CYP240A1|[Bordetella pertussis]bpeu|BP

MIADTARQHRGDSMQPADPLEAVAHPDPYPYYAALARERPFYHDDRLGLWVAAGPQAIRA
VLTCPAARVRPPGEPVPAALGAGPAAQMFGRFIRMNDGAVHERLKPMLTAYLTQRTAADL
AEPAWPAIGNDPAQVDRYLYQAPVHAQACLMGLPDEVAASCAREIEAFMAACRPGADAAA

VARADQAAQALQARMLAHLRAARGDAALGVLRRLALAGGVEADALAANLAGLLLQSCEAG
AGLLDNALVHAGRLSPAAAPDLLHTCVEIVTHVARHDPPLHNTRRFLAAPATLLGQSAPA
GAGILVVLAAAHALAEGAWPWTFGAERHACPGRTPALLHAAQALAHALRHGVDAPALARC
VRYRPLPNARVPRFHFPPGDTP

>BN117_3234|KEGG|CYP240A1|[Bordetella parapertussis]bpar|BP

MIADTARQHRGDIMQPADPLEAVAHPDPYPYYAALARERPFYHDDRLGLWVAAGPQAIRA
VLTCPAARVRPPGEPVPAALGAGPAAQMFGRFIRMNDGAVHERLKPMLTAYLTQRTAADL
AEPAWPAIGNDPAQVDRYLYQAPVHAQACLMGLPDEVAASCAREIEAFMAACRPGADAAA
VARADQAAQALQARMLAHLRAARGDATLGVLRRLALAGGVEADALAANLAGLLLQSCEAG
AGLLGNALVHAGRLSPAAAAAAPDLLHTCVEIVTHVARHDPPLHNTRRFLAAPATLLGQS
VPAGAGILVVLAAAHALAEGAWPWTFGAERHACPGRTPALLHAAQALAHALRHGVDAPAL
ARRVRYRPLPNARVLRFHFPPGDTP

>BPP3270|KEGG|CYP240A1|[Bordetella parapertussis]bpa|BP

MIADTARQHRGDIMQPADPLEAVAHPDPYPYYAALARERPFYHDDRLGLWVAAGPQAIRA
VLTCPAARVRPPGEPVPAALGAGPAAQMFGRFIRMNDGAVHERLKPMLTAYLTQRTAADL
AEPAWPAIGNDPAQVDRYLYQAPVHAQACLMGLPDEVAASCAREIEAFMAACRPGADAAA
VARADQAAQALQARMLAHLRAARGDAALGVLRRLALAGGVEADALAANLAGLLLQSCEAG
AGLLGNALVHAGRLSPAAAAAAPDLLHTCVEIVTHVARHDPPLHNTRRFLAAPATLLGQS
VPAGAGILVVLAAAHALAEGAWPWTFGAERHACPGRTPALLHAAQALAHALRHGVDAPAL
ARRVRYRPLPNARVPRFHFPPGDTP

>BN112_4694|KEGG|CYP240A1|[Bordetella bronchiseptica]bbh|BP

MIADTARQHRGDSMQPADPLEAVAHPDPYPYYAALAREQPFYHDERLGLWVAAGPHAIRA
VLTCPAARVRPPGEPVPAALGAGPAAQMFGRFIRMNDGAVHERLKPMLTAYLTQRTAADL
AEPAWPAIGNDPAQVDRYLYQAPVHAQACLMGLPDDVAASCAQEIEAFMAACRPGADAAA
ARRADQAAQALQARMLAHLRAARDDAALGVLRRLALAGGIEADVLAANLAGLLLQSCEAG
AGLLGNALVHAGRLSPAAAAPDLLHTCVEIVTHVARHDPPLHNTRRFLAAPATLLGQSLP

EGASILVVLAAAHALAEGAWPWTFGAERHACPGRTPALLHAAQALAHALRHGVDAPALAR
RVRYRPLPNARVPQFHFPSGDAP

>BB3721|KEGG|CYP240A1|[Bordetella bronchiseptica]bbr|BP

MIADTARQHRGDIMQPADPLEAVAHPDPYPYYAALARERPFYHDDRLGLWVAAGPQAIRA
VLTCPAARVRPPGEPVPAALGAGPAAQMFGRFIRMNDGAVHERLKPMLTAYLTQRTAADL
AEPAWPAIGNDPAQVDRYLYQAPVHAQACLMGLPDEVAASCAREIEAFMAACRPGADAAA
VARADQAAQALQARMLAHLRAARGDAALGVLRRLALAGGVEADALAANLAGLLLQSCEAG
AGLLGNALVHAGRLSPAAAAAAPDLLHTCVEIVTHVARHDPPLHNTRRFLAAPATLLGQS
VPAGAGILVVLAAAHALAEGAWPWTFGAERHACPGRTPALLHAAQALAHALRHGVDAPAL
ARRVRYRPLPNARVPRFHFPPGDTP

>BN115_3393|KEGG|CYP240A1|[Bordetella bronchiseptica]bbm|BP

MIADTARQHRGDSMQPADPLEAVAHPDPYPYYAALARERPFYHDDRLGLWVAAGPQAIRA
VLTCPAARVRPPGEPVPAALGAGPAAQMFGRFIRMNDGAVHERLKPMLTAYLTQRTAADL
AEPAWPAIGNEPAQVDRYLYQAPVHAQACLMGLPDEVAASCARDIEAFMAACRPGADAAA
VARADQAAQTLQARMLAHLRAARDDAALGVLRRLALAGGVEADVLAANLAGLLLQSCEAG
AGLLGNALVHAGRLSPAAAAAAPDLLHTCVEIVTHVARHDPPLHNTRRFLAAPATLLGQS
VPAGAGILVVLAAAHALAEGAWPWTFGAERHACPGRTPALLHAAQALAHALRHGVDAPAL
ARRVRYRPLPNARVPRFHFPPGDTP

>BBS798_3501|KEGG|CYP240A1|[Bordetella bronchiseptica]bbx|BP

MIADTARQHRGDIMQPADPLEAVAHPDPYPYYAALARERPFYHDDRLGLWVAAGPQAIRA
VLTCPAARVRPPGEPVPAALGAGPAAQMFGRFIRMNDGAVHERLKPMLTAYLTQRTAADL
AEPAWPAIGNDPAQVDRYLYQAPVHAQACLMGLPDEVAASCAREIEAFMAACRPGADAAA
VARADQAAQALQARMLAHLRAARGDAALGVLRRLALAGGVEADALAANLTGLLLQSCEAG
AGLLGNALVHAGRLSPAAAAAAPDLLHTCVEIVTHVARHDPPLHNTRRFLAAPATLLGQS
VPAGAGILVVLAAAHALAEGAWPWTFGAERHACPGRTPALLHAAQALAHALRHGVDAPAL
ARRVRYRPLPNARVPRFHFPPGDTP

>Bpet1785|KEGG|CYP240A4|[Bordetella petrii]bpt|BP

MARRARRLRGAAGLTPAIFRPASKRHGRPAPHTGAMHPADPIQAVTHPDPYPYYRTLARE
RPLYYDPALGLWVASSPPLIHAVLAHRAARVRPAAEPVPRALGDDAAGLLFSRFVRMRDD
AAHAGLKPLLVDCLQALPCGGAPCWPAAPHTLPGLDEFLFAAPVYAMASRLGLAQPAIAA
CARDVRLFRAALGPAATADQLAAGHAAAARLQTAMMEHLHAQADDAPLALLRSRAAQAEF
EPAEVAANLAGLLFQSCEAGAGLVGNTLAALGRDPGLCAAARRDAALCLHAAAHTARHDP
PVHNTRRYLDASMSLDGQVLPAGATVLLVLAAAGAAAPGDAPWTFGAGRHACPGRTPALQ
AAGQAVAFLLDAGLEPAALTQKLGYWPLPNARVPRFARHGVQAK

>ACR54_04430|KEGG|CYP199H1|[Bordetella hinzii]bhz|BP

MSQSQLAAPVVDWDPYDDAVLESPFEMHARLRAAGPLVFLERYGVYAMGRHRDIQPALKD
WETYSSTSGSGIADIRKPGAWRPGSPIVEVDPPQHTQVRTAVQRILSPVVIRQWRGDFER
EADRLVAELVERGRFDGVTDLAETYVATTFPDALGLAPSPQRRENLFLLGELNFDGQGPR
NARYLATQARADLIQDWYEASMRREAMLPGGFGEKIFQAADAGAIDPAIAPLLVRSFLRG
GLDTTSSSISATLAYLARDPAQFALLREDPGRARAALEEAMRLETPIQTVCRLTMRDVAI
DGVAVPRDNKIVMMLASANRDPDFWERPDDYDMTRSTLGHVALGNGIHMCIGQMVARLEG
ESVLKAVAKHIGELRLEGEPVRKLNNNLRSLKSIPLSVKPA

>SAMEA390648700219|KEGG|CYP212A17|[Bordetella trematum]btrm|BP

MARDLPGALAAWQQRHGDVVHLRLWPEHVVVLCDPQLARELLLEHHDALIRWERGVRAFA
QIHGQSVLTTEGDAWRSRRQALNPGFSPKAVQAFAPLIISTAAQSLARWPQEDARWPIES
RLAEMTMEVILQMVFSGSHEEDAGAIEEAVRITSATTHAAFYAAASLPDWLPWKRRQRRA
RLLLQTMIERQVQARLAIARDAWPPDLLSRLLTCQPDLSASSLRDVRAECMTLFLAGHET
TAATLTWWAWCMAANPQAQSRAREDVRLALGPDTPSVDALQNAGYLSQTLKETMRLYPAA
PMLMSRRTTRPLRLGSWTFAPRTLFMIPVQAIQADARRFPDPQRFQPERFAKQAPEIPRG
AYFPFGAGPRVCLGQHLAMTEMGLLAALMLQRFEFSVCEDAPPPRPVLNVTLRPAQALRL
AIKRIDGG

>CBF45_10850|KEGG|CYP1232F2|[Bordetella sp.]boj|BP

MDVLTTDVPVADWVDLAELMADPYPTYARLRREMPVAWVPALNRFLVTGFDACFQAELDQ
ETFSSHEDAERSTQVRTMGRSMLRKDDPEHKRDRAASAGALRPVTIRKVWMDIFERNASR
YLQHVQALGPGVDIFREFAVPFAADNLCAILGLRGVDASTMMDWSHTLIAGIGNVTDDQD
VWRETQRVRYEMDAAIDESVAWLSREPDASMLSAMLHAAEPLSDEAMRANVRLTVSGGMN
EPSHVIGSAIWALLTHPDQLAEVRAGVWNWADVFEETARWQSPVGMYPRLVRKETWLAGV
RIPAGATVGLVIASANRDERRFSHAERFDLRRERLTNLAFGNGTHICAGNWAARAMIGNV
ALPRLFEALPGLRLEDPQAVAFRGWVFRGVRSLPVCWEAT

>AXYL_01591|KEGG|CYP240A3|[Achromobacter xylosoxidans]axy|BP

MHPAHPLQAVTHPDPYPYYAALARERPLYREAALDLWVASHPDTIAAIMRHPAARVRPVA
EPVPKGLGTGPAADLFGRFVRMNDSRIHQQVKTLLSAFIREQGAQTCPPAWPDPGPLNAM
AVDRYLSAAPVHAQAAFLGLPPDLYADCAADIAAFLSALPATATADRIEAGHAAAMRLQT
RLESHLQAPDAAPALRRLREDGARAGIVPALLAANLAGLLFQSCEAGAGLLGNAIILAGR
RGLPADPTEDAALALMDEVERSDPPLHNTRRFLAADIDIGGQRIAAGQTVLLVLAAAATV
QPEAHWTFGAHGHACPGRLPARQHAAGALSHLLRAGIDAAALAARFRYRPLPNARVPQFD
FSEEPAP

>AXYL_03286|KEGG|CYP116B81|[Achromobacter xylosoxidans]axy|BP

MGHPSTPAPTPPANTGGCPIDHAALARAQACPVSPRAAEFDPFGDGYQQDPPEYVRWARE
QEPVFYSPKLGYWVVTRYDDIKAVFRDNHTYSPSIALEKITPTGPEANAVLATYGYAMNR
TLVNEDEPAHMPRRRVLMDPFTPDELKHHEPMVRRLTREYVDRFIDDGRADLVDQMLWEV
PLTVALHFLGVPEEDMDLLRKYSIAHTVNTWGRPKPEEQVEVAHAVGNFWQLAGKILDKM
RQDPDAPGWMQYGIRKQKDHPEVVTDSYLHSMMMAGIVAAHETTANASANAIKLLLQHPD
AWRELCEDPGLLPNAVEECLRHNGSVAAWRRLATRDTEIAGVAIPAGAKLLIVSSSANHD
ERHFADADFFDIRRDNASDQLTFGYGAHQCMGKNLARMEMQIFLEELTRRLPHMRLAQQQ
FTYVPNTSFRGPEHLWVEWNPADNPERRDPALLVQAQPVRIGEPSTHAISRKLVVTQVTA
AADGIARIRLESPDGKPLPRWTPGSHIDVECGDTGLSRQYSLCGDPAAANALEIAVLKET
AGRGGSAWIHQHLRAGSIVRARGPRNHFRMDESAKRLILIAGGIGITPISAMARRARELG

MAYELHYSGRSRACMAMLEELQQLHGERLRLHISGEGSRNDFAALLSLPEAGTQIYACGP
ERMLDALQAACSAWSEDSLRVEHFHSTLATLDPSKEHAFEAELKDSGIVVQVPAGQTLLT
ALRSANIDVQSDCEEGLCGSCEVRVLGGAVDHRDVVLTRAEREAGTRMMACCSRAQGGRI
VLEL

>AXYL_03542|KEGG|CYP1110A2|[Achromobacter xylosoxidans]axy|BP

MRPTDIPDLKSAAFLADRYPTYRRLQSDFPHFEMDINGEECIVLTRYSDVDEVLRNPLAT
VQQAPGVFPERIGQGAGARFYRESLPNIDAPDHTRIRRIVTPAFNPKTVANMRGWVEKVI
VEHLDRLEGQDEIDFVSSFADPVPAEIACQLLHVPVSDAPELFARQHGLNAVLSVSDITP
ERLAEADASAAYYYEYMDDVLDTLKGKLPEDDFVGALMAAEGGDNGLTRSELVTTLIGFL
VASYHTTKVAMTNTVLTLLNHDGERARLVAQPDLARNAWEESLRYDSPVHFVHRYASEPL
TIGGQPVAQGKRLLLGLHAASRDENRFAQADHYLIDRPDNRHLAFAGGGHFCLGSQLSRL
EGDVLLRTIFQRFPAMRLTETRFERVPDLTFPMLLRMTVSLRAGQG

>AXYL_04967|KEGG|CYP108S2|[Achromobacter xylosoxidans]axy|BP

MNHTDFDLPSDVAKALVNAQVYANIDELHSTYSWARANNPLGRAVNEGFDPFWVVTKHAD
VAAISRDSQIFRNGDYATISAPKAMIDHLKAVTGSYNGGIKTLVHVDGDEHKGLRTLTQA
WFMPNSILKLDERIRDLACRTVDKLAAAEGEVIDFATQIALHYPLHVIMDILGVPAEDEA
RMLLLTQELFGTEDPEFKREGKDAGGNDDALAQNVLAIVADFRAYFEKVTADRRVNPRND
VATLLANAVIDGVPLSEDSRLGYYVIIATAGHDTTSSSTAVAMWALSQYPELLPRLQANP
GLIPQFIDEAVRLASPVRHFMRTAAADTEIRGRRIHKGDWLMLCYGSANRDEDVFENPFE
FNIDRKPNRHLSFGLGAHICLGQHLAKMEMRILFEEMIPRLQRVELAGDMAMSASCFVGG
PKHLPVRCFMN

>NH44784_004871|KEGG|CYP240A2|[Achromobacter xylosoxidans]axo|BP

MSTPFPTDPLQAVTHADPYPYYAGLARDRPLYRDARLGLWVASAPGTIHDIMRHPAARVR
PMAEPVPRGIAAGPAGLLFGRFLRMNDGPRQARLKALFRAFLARQAPLAPAPDWPRLEMD
TGSAGAIDRYLHAAPVFAQACALGLPGAVAAECAREVGAFLAALPPAAPEDRVAAGHTAA
ERLLARLGAHLDDRAASPALRELRGRGADAGLEPALLAANLAGLLFQSCEAGAGLLGNAL
LRAGRQRAGAALTLAQAWELVDATLRHDPPIHNTRRFLAGPIELEGQLMQAGETVLLALA

AAAMAQPEAHWTFGAQSHACPGRDAARRDAAGALVHLLRAGVDGAALAARFRYRPLPNAR
IPQFDFTEEPTP

>AX27061_1499|KEGG|CYP240A2|[Achromobacter xylosoxidans]axn|BP

MSTPFPTDPLQAVTHADPYPYYAALARDRPLYRDARLGLWVASAPGTIRDIMRHPAARVR
PMAEPVPRGIAAGPAGLLFGRFLRMNDGPRQARLKALFRAFLARQAPLAPAPDWPRLEMD
TGSAGAIDRYLHAAPVFAQACALGLPGAVAADCAREVGAFLAALPPAAPEDRVAAGHTAA
ERLLARLGAHLDDRAASPALRELRGQGADAGLEPALLPANLAGLLFQSCEAGAGLLGNAL
LRAGRQRAGAALTLAQAWELVDATLRHDPPIHNTRRFLAGPIELEGQLMQAGETVLLVLA
AAAMAQPEAHWTFGAQGHACPGRDAARRDAAGALVHLLRAGVDDAALVARFRYRPLPNAR
IPQFDFTEEPTP

>C2U31_19705|KEGG|CYP1199C1|[Achromobacter sp.]achr|BP

MYEMDNRTIHYHKEIRRLFDDPSQRGVFRIDEQTVGVTDGPLSKAILAARPMYDFERIVF
KPILGQPIAKATFSAVMRAVGQDARRVAESPPEPGPRLAGRWPDAGYLYLQQRLYASDPW
PIYALTHRVANRTGWLKPLVDGLSSRLMTRLLHRCAPIHSSALAQLVNEAPDAHERRRAV
TLYRRTARSMCITVACLVTNALWLASARDARERPRDCITETLRLMPPAWMYHRNACQEFT
AVDARIRETDDVLVIPLITQRDPRHWPDADAYEPDRWLGVSNPEASDHYLPFGHSHDRCW
ARELVLMLAEHTIRQIAMQDHWTDSRQTSVQVPLKSLLCPTRVNVVAV

>C2U31_30205|KEGG|CYP1110A3|[Achromobacter sp.]achr|BP

MRPTDIPDLKSAAFLADRYPTYRRLQSDFPHFEIDINGEDCIVLTRYSDVDEVLRNPLAT
VQQAPGVFPERIGNGAGARFYRESLPNIDAPDHTRIRRIVTPAFNPRTVANMRGWVEKVI
VEHLDRLEGQDEIDFVSAFADPVPAEIACRLLHVPLSDARELFARQHGLNAVLSVSDITP
ERLAEADASAAFYYEYMDDVLDTLRGKLPEDDFVGALMAAEGGDNGLTRSELVTTLIGFL
VASYHTTKVAMTNAVLALLSHDGERARLMAQPDLARNAWEESLRYDSPVHFIHRYASETL
TVGGRDIARGKRLLLGLHAASRDEARFPQADRYQIDRPDNRHLAFAGGGHFCLGSQLSRL
EGDVLLRTIFQRFPGMRLTETRFERVPDLTFPMLLRMTVALRAPQG

>C2U31_20070|KEGG|CYP2308A1|[Achromobacter sp.]achr|BP

MNDMRFNPFAKDFSAAPYAHYRDLREQDPVHRSAMGIWVLTRYAE
VSQVLRDPSYSSEVSRWPGFAQRYRSRPGVAWLLTHSVLNRDEPDHKPLRRAIIQAFSP
ASEGRLARMIDDILDQRLNALDGDQDFDAVGDFALPVPVSTVCRVFDVASADQARVKAW
SNQVASLIEPLPAAPVLGDAAAGIAAFKEYLREKMECPEDAGLAGDLMRSMQEHPVGLE
DALANLVLMFPAGHETTVNLIGNGLLLLLRHPEQRRLLREQPDLWPKAIEEILRVESPQ
QIAWRVATQDHELAGKRIAAGDQLMLVLGAANRDPEVFEDPERFDITRTNNRHVAFGLG
RHACLGGWFARMQGQRALQAIVARFPRLSLAGAPLWHPTVSFHGLRHLPVTQTPAFPNG
GQQ

>DVB37_24515|KEGG|CYP116B73|[Achromobacter sp.]achb|BP

MSQSPSSSRPSGCPIDHAALAAMQSPTGCPVSSRAAEFDPFGDGYQQDPPEYVRWAREQE
PVFYSPKLGYWVVTRYEDIKAIFRDNITFSPSIALEKITPTGPEANAVLESYGYAMNRTL
VNEDEPAHMPRRRVLMDPFTPEELKHHEPMVRRLTREYVDRFINDGRADLVDQMLWEVPL
TVALHFLGVPEEDMELLRQYSIAHTVNTWGRPKPEEQVAVAHAVGNFWQLAGKILDKMRQ
DPSGPGWMQYGLRKQQTHPEVVTDSYLHSMMMAGIVAAHETTANASANAIKLLLQHPDAW
REICEDPDLIPNAVEECLRHNGSVAAWRRLATRDAEIAGVPIPAGSKLLIVTSSANHDEG
QFNDADLFDIRRENASDQLTFGYGSHQCMGKNLARMEMQIFLEELTRRLPHMKLSDQAFT
YVPNTSFRGPEHLWVEWDPKQNPELASPELLNDQHPVRIGEPSSHAITRPVVVQSAVEAA
DGILRLRLVSPDGKPLPRWTPGSHIDVECGDTGLSRQYSLCGDPSEAGVFEIAVLKETEG
RGGSAWMHANAVVGARLRIRGPRNHFRMNEAADKLILIAGGIGITPISAMARRAKELGLD
YQLHYSGRARTCMAMLDELVALHGDRLHVHVKDEGTRADYAALLSQPDAGTQIYACGPQG
LLDGLAACCAAWPEDALRVEHFHSTLGTLDPSKEQPFEVVLKDSGIVINVPADQTLLTAL
RGANIDVQSDCEEGLCGSCEVRVLEGQIDHRDVVLTRAERDANNKMMACCSRSCGGGKVV
LEL

>HLG70_25295|KEGG|CYP116B298|[Achromobacter deleyi]ady|BP

MSQAPTSPRPSGCPIDHAALSAAQGPTGCPVSARAAEFDPFGDGYQQDPPEYVRWARDQE
PVFYSPKLGYWVVTRYEDIKAIFRDNITFSPSIALEKITPTGPEANAVLESYGYAMNRTL

VNEDEPAHMPRRRVLMDPFTPEELKHHEPMVRRLTREYVDRFVNDGRADLVDQMLWEVPL
TVALHFLGVPEEDMDLLRQYSIAHTVNTWGRPKPEEQVAVAHAVGNFWQLAGKILDKMRQ
DPSGPGWMQYGLRKQQTHPEVVTDSYLHSMMMAGIVAAHETTANASANAIKLLLQHPDAW
REICEDPDLIPNAVEECLRHNGSVAAWRRLATRDVEIAGIAIPAGSKLLIVTSSANHDEG
QFADADLFDIRRENASDQLTFGYGSHQCMGKNLARMEMQIFLEELTRRLPHLKLSEQSFT
YVPNTSFRGPEHLWVQWDPQRNPERADPALLDGQHPVRIGEPSSHAITRPVVVQSAVLAA
DGILRLRLASPDGKPLPRWTPGSHIDVECGDTGLSRQYSLCGDPAEAGVFEIAVLKETDG
RGGSAWMHEHALPGARLRIRGPRNHFRLNEAAGKLILIAGGIGITPISAMARRARELGLD
YQLHYSGRSRRSMALLDELAQLHGARLHLHIKDEGRRADYTALLAPEAGTQIYACGPQN
LLDALAGCCAAWPEDTLRVEHFHSTLGTLDPTKEQAFEAVLKDSGIVITVPAGQTLLTAL
RGANIDVQSDCEEGLCGSCEVRVLEGDIDHRDVVLTRAEREANTKMMACCSRACGGKKIV
LEL
>PT7_0379|KEGG|CYP289A16|[Pusillimonas sp.]put|BP

MHTACPHIHDWNPRDERVQQNQIAAYDTMRKQCPVARSEYLHWSVFKHAEVVRILNDHDT
FSNQVSPRASVPNGMDPPEHTPFRQLIEPYFDPSAMADFKPACESIARNLAENLHQGQID
IMSQLAHPYALEVQCAFMGWPATLHQPLRDWMRKNHKATLAKDRAAMDQIAQEFDGYMLA
LINERRQAGANAPDDTTTRLTRGHVDGRPLNDEELISILRNWTVGELGTIAASVGILAHY
LAAHPALQEQLRKQPELLPPAIDEILRIHAPLIANRRITTRPVELGGRHLDAGERVSILW
ASANRDEAVFGDPDDFRLDRDPLLNLLYGAGIHVCPGAPLARMELRLLMEALLACTQRLD
LTSGQPPIRARYPGGGFTSLILQVL

>PT7_2686|KEGG|CYP1183A1|[Pusillimonas sp.]put|BP

MNTTTRSVDDYSFMDPDVQDNPFEFYELLQQERPVYQMPETGFFVVTRYEDVRKVVADFK
TFSNDLVREKEFQKEKYAIHSGVLAERGWPLMQTLQRTDPPVHGRYRRLLDKVFTPKQVQ
QLKPRIDDIVNHLIDDFIDKGECEFVHDFALMLPGLLIAEQIGLDDDVLTFKKWVDAMFA
TGMNILSEDEIRKAAEIELEAQLYFAKIFEDRRKNPRDDIVSNLVHASVGEDEAPLSTQE
LQSLMRHIVGAGFESTASALASGMLRLINHPDQLKKLKSDRSLMPGFVEEVLRIDSPVQG
HFRKAMKDVTIGDVHIPAGSIVIPRYGAANHDPEQFQCPHAFDIERKNANRHLAFGMGTH
FCLGANLARQEISSAFSTILDRMDEIKLARPLVRPIHQVNFILYLLNELPITFVKRNTQP
NQG

>CKA81_16930|KEGG|CYP1157D3|[Pusillimonas thiosulfatoxidans]pus|BP

MTDHMPDDLSAIDRDLRAYTDELRPRHGVVRNIQGEWVLLRHDLVVQAAQDHDRFSNAVS
SHLQVPNGIDAAEHTAYREALDDFLSPAALAPFVKAFRQVASDLVASLPRNVPVDAVSEF
GVRFAVRAQSAWLSWPTELEDRLVDWVAQNHAATRSGDRSRMRQVAQAFDDLVRSVMLPR
RGVESKHDLNDVTARLMRTEVNGRPLSDAEIVSILRNWTGGDLGSIAQCIGVLLHCLATR
PFLQDHLRNGVPAAEFDAIIDEILRVDDPFISNRRVTTCPVTIGGVDLPQGARVKLNWTS
ANRDEAVFGDPDAMEPDRNAEHNLVYGIGKHVCPGRHLATLEMRIALQELLAATLSVALG
PQHLAQRAVSPVGGWAKLAIVLT

>G9Q38_04420|KEGG|CYP199V2|[Pusillimonas sp.]pud|BP
MVSLATFTKDAVPVTDFDPYAEEVLIDPYPYYEQLREAGPVVLLERYGAYALGRHDEVHA
VLSDAHTFCSGAGVGLTNFNTEKPWRKPSIILEADPPHHARTRKVVARVLSPASIGRLRE
TFEAEADALVDKIIQMGQFDAAKDFSEAYPLKVFADAVGLPAEGRENLLPYGDMVFNGFG
PMNERFTSRLAKAEESVKWINNVCQRENLTPNGLGADLYKAADAGEITHEEAGMLVRTLL
SAGLDTTVFTLCNAMLCFAKYPDQYEVLRENPQLARQAIEEVIRFEPTFHSFYRTTTKPV
EVAGVEMDANQKICVFVGSANRDPRRWSDPDKFDVSRKATGHIGFGTGIHGCAGQMMARL
EGDILLRAFAKKVRSIELMGEPVRHYNNTVRGFKQIPARVVAL

>G9Q38_04600|KEGG|CYP199A38|[Pusillimonas sp.]pud|BP

MTTSTSTTTPPDSVPRLDIDPFGDDFLTMPYPYHEQMREAGEAVWLDSYGLWAFTRYEQV
KAGLSDWENFISSAGVGLANFRVEKPFRPPSLILEADPPDHTKARAILSRILSPKVVQQI
RGDFQEKAEVLIDRLLEQGEIDAVNDLAKVYPLQVFPDALGLAEDEREILLLYGDMVFNS
FGPRNHIFQRAVEKYEPVREWIMNKCRREELRPGGFGDQIYQAADAGEITLEEAPMLVRS
FLSAGVDTTVNGIGNAIYCFAQFPEQYDKLAAEPKRARPAFEETLRFESSVQTFFRTVNR
DVEFAGAPMKENDKVLFFLAAANRDPRKWENPDVFDIDRKATGHMAFGTGIHGCVGQAIA

RLEGELILTALANKVKRIEQIGEPTRRLNNTLRALDSLPVRLVPR

>TKWG_13525|KEGG|CYP116B8|[Advenella kashmirensis]aka|BP

MNTQSTSQPTQAQCPYSGAHTNAGTGCPVSARAAQFDPFEDAYQQDPPEYVKWSREQEPV
FYSPKLGYWVVTRYEDIKQIFRDNITFSPSIALEKITPTGPEANAVLESYNYGMSRTLVN
EDEPAHMPRRRLLMEPFTVDALKHHEPMVRALTRQYVDRFINDGKADLVEQMLWEVPLTV
ALHFLGVPEEDMDTLRKYSIAHTVNTWGRPKAEEQVAVAHAVGNFWQLAGKILEKMRKNP
EQPGWMQYGIRMQQLHPDVVPDSYLHSMMMAGIVAAHETTANASANAIKLLLSHPHVWQE
ICEDPNLIPNAVEECLRHNGSVAAWRRLATRDVQIGGVSIAAGSKLLIVSSSANHDERHF
ADADLLDIRRENASDQLTFGYGSHQCMGKNLARMEMQIFLEELTLRLPHLILSEQTFTYV
SNTSFRGPEHLWVHWDPSANPERQDPSILNRQHPIRLGEPSTHSLSRQVVVQETEMLTDD
IMRIRLAAADGQTLPRWAPGSHIDLVCGDLGISRQYSLCGDPANPQVMDIAVLREDAGRG
GSAWVHQHVRSGLPLHIRGPRNHFHFDEQASKAIFIAGGIGITPIIAMARVAKNRGIDYQ
IHYCGRSRKSMALLASLQEHGERLHVYAADEGLRADFASLLATPDAETQIYACGPQRLTD
CLQELCECWPDDALHIEHFHSTLSKLDPSREHAFEAELKQSGITVTVAANQTLLEALRAA
NIDIQSDCEEGLCGSCEVSVLGGQPDHRDVVLTRSERAAGNKMMSCCSRAKDKHIILDL

>BN940_07996|KEGG|CYP289A15|[Castellaniella defragrans]cdn|BP

MNTPQDWDPRAPEVLADPIAAYDRMRRRCPVAHSDYLHHSVFRHADVMRILLDHETFSSQ
ASRHVSVPNSMDPPEHTAYRRLIEPYFAPERMAAFEPACRRLCAELVAALPRNERVEIMF
GLAHPFALRMQCAFLGWPDSLHEPLRQWIHKKNVATLSGDAHAIAAVATEFDETIRALLK
VRRDAGDQAPDDATTRLLRETVNGRPLAEDEIVSILRNWTVGELGTMASSVGIIAHYLAS
HPDLQDRLRSQPELVRPANDEILRIHAPLIANRRATTCPVRLGGTQIPAGERVTLIWASA
NRDEAVFGDPDEFRLDRDPSLNLLYGAGIHACPGAPLARLEIEAMIQALLAGTRALEPVA
EQPPMPAIYPASGYRQAMLQLRHD

>AFA_04380|KEGG|CYP1319A1|[Alcaligenes faecalis]afq|BP

MFGDFNLASPYPAYARLRQGPPLVWDADFCGGAWLVHRHGDVQAALRHPDLSVRRVGGWV
SQAGAHPSPRLQTFKGLMARTLVFLDRPRHTRVRRVVNAAFSAKALDTLHGTIEKRVAQI

HLGLENKLQHGSADLVDEVCRPLPALVMMDVLGLQQLPLSVFQAWSEQLARFIGQATPDQ
PLLMDTQDALLDMADFLGNSANLQEGGLAWRLLHDEQLSLQGRRERLAQSCMLLFAGYET
SRHLLSSLFQTLLDDCTKQQDLLSNPQKIPAAIKEVLRHDSPIQYTARRLMQDQHWHGQH
LRKGQLLLLLLGAANRDPEIFAHPDELDFNRNNQAELSMGHGIHHCLGAGLVQLEARLTL
EQFLPLLPRLQLGQGERIQLPAYRGWNRLSVQYRALLS

>D3M96_05490|KEGG|CYP1319A1|[Alcaligenes aquatilis]aaqu|BP

MFGDFNLVSPYPAYARLRQGPPLVWDADFCGGAWLVHRHSDVQAALRHPDLSVRRVGGWV
SQAGANPSPRLQTFKGLMARTLVFLDRPRHTRVRRVVNAAFSAKALDTLHGTIEKHVAQI
HLSLENKLQHGSADLVDEVCRPLPALVMMDVLGLAQLPLSVFQAWSEQLARFIGQATPDL
PLLMDTQDALLELADFLGNSANLQEGGLAWRLLHDEQLSLQGRRERLAQSCMLLFAGYET
SRHLLSSLFQTLLDDCIKQQDLLSNPQKIPAAIKEVLRHDSPIQYTARRLMQDQHWHGQY
LRKGQLLLLLLGAANRDPEVFAHPDELDFNRNNQAELSMGHGIHHCLGAGLVQLEARLTL
EQFLPLLPRLQLGQGERIQLPAYRGWNRLSVQYRALPS

>FE795_04400|KEGG|CYP1319A1|CYP1319A1|[Alcaligenes ammonioxydans]aamm|BP

MFGDFNLASPYAAYARLRQGPPLVWDANFCGGAWLVHRHQDVQAALRHPDLSVRRVGGWV
SQAGAPPSPRLQAFKGLMARTLVFLDRPRHTRVRRVVNAAFSPQALRDIDRSIENRVTHL
HSAFTNQLRHGPADLVEQVCRPLPALVMIDLLGLQQLPLAVFQSWSEQLARFIGQATPDL
PLLMDTQDALLDMADFLGNSAKLQEGGLAWRLLHDEQLSLQGRRERLAQSCMLLFAGYET
SRHLLSSLFQTLLDDASKLQDLLLNPQKIPAAIKELLRYDSPIQYTARRLMQDQHWHGQH
LRKGQLLLLLLGAANRDPEVFSHPDDLDFSRNNQAELSMGYGIHHCLGAGLVQLEAKLVL
EQFLPLLPRLQLAQRERIQLPAYRGWNRLLVHYRAMPS

>ODI_R0617|KEGG|CYP1962A1|[Orrella dioscoreae]odi|BP

MSAGLLLAEHEAAPAGPAPGQAVPRRDDAAILTPLFQDDLIARERDGVLRLYKALTRDRP
LFRDANGSWVVSSFEQGCAVLSDPAYVAQPPLPAEEAAVYAPILATMLLRDGDAHAALRR

HFAPLFKTQRMDRLHAFMDQDIATCVAALPASAFDAGASLAHHLPARMACVLMGLAPEHA
PRLVALTAPVLRLISGTPLPPTSMLALLQASRVAIQALATLMARQAPATLHYLDIDPHAL
ATHALSDGTEHASCASNLCLLFVAAYTTSMLSLGNTLALATQRPGLWTQMAQEPSRIPRT
VAELGRLAPAAQALQRYAAHDLTLGDVPLRAGEPVVVMVAAANRDPRAFEHPDEVDLGRH
ARTLTFGAGPHGCVGVGLARRQLGGLLAALTSRYPRLAPLARPSRGLQIGTFLGYDRLWL
GVPAESAKAPAPVRQPNA

>DBV39_09585|KEGG|CYP152E6|[Orrella marina]boz|BP

MLKTPSDKTLDSTLPFARDGYLFVTKRCDRYRTDLFQTRLLLRKSICMRGEDAARLFYDE
GRFERSGVAPVRVQETLFGRGGVQGSDGAAHRWRKEMFLSLMSAQAISRLGELSAMQWRA
YAAKWEEQEQIVLLDEACEILCRAVCEWAGVPLAELEVTKRARDFAAMIDAGAAIGLRHW
RGRWARNRAEKWIGGIVEDVRNHRLAPDEGSALHMIAFHRDRQGQLLNERIAAVELINVL
RPTVAVAWYVTFAALALHQYPECRQRIAAAQEGYLECFVQEVRRFYPFFPVVAARTRHEF
EWKGYRLPGRTRVLLDLYGTDHDTRLWDRPDAFVPERFRHWDGSAFNFIPQGGGSHRNGH
RCPGEWITIELMKVAAEFLSARIQYEVPQQDLRISLSRIPALPNSRFLIRNVRQI

>I6G29_02045|KEGG|CYP289A28|[Oligella ureolytica]oue|BP

MSNNEKLSDDVYQASISSEPIAAYDKIRRACPVKHDSHLHWALFKHADVLSVINDHDTFS
SVVSKHVSVPNGMDPPEHTVYRQLIDPYFNEERMAAFEHSCQKIADTLVQNAPKNTEFDL
IEHFARVYAVQVQCAFLGWPESLQEPLTQWVRKNHRATLAQDRPAMSEIALEFDGFIREL
LNTRRADGQDAPDDLTTALMQETVDGQVLADEIIVSILRNWTVGELGTIAACVGIVVKYL
ADHPELQQKLREQPALLDDSIDEILRIHPPLISNRRVTTKEVSLGGETIGANQRLTILWA
SANRDEAVFGDPDEFCPERNGEHNLLYGAGIHYCPGAPLARLVLRLVIGALLRDTQGFLL
SPNHEAVHAKYPAGGFDLLPITLVAGSCCGGCSG

>EGT29_07245|KEGG|CYP199A34|[Pigmentiphaga sp.]pig|BP

MTDIAANIPVVDFDPFSDAFLTDPYDYHRRMRDAGPVVHIPKYGILAVARHAEVHGVLND
WQTFISSAGVGLANFRTEKPFRPPSLILEADPPQHTRSRTVLGRILSPKTVQQIRAQFQE
VAEALIDRLLEQGTIDGVKELGEAYPLKVFPDAVGLTEDGRENLLAYGDMVFNAFGPRNQ
LLINAAKRVEPLTDWIMTNCQRQNLRPGGFGDQIYQAVETGELTETEAPMLVRSFLSAGV
DTTVNGIGNALWCLANHPDQWDKLHANPALARQAFEESLRFEAAVQTFFRTAGRDCELAG
VPIRSDTKMLTFLASANRDPRQWQNPDVFDIERRATGHMTFGSGLHGCVGQVVARLEGEL
ILSTLARRVRRIEPLGPPTRRLNNTLRALASLPLKLHAA

>EGT29_08175|KEGG|CYP1108A5|[Pigmentiphaga sp.]pig|BP

MPTPPPDWDPLAPARHGDPAEVYGGLREKCPVAWSDRFDGFYAISRFDDVVKAALDVETY
SSAKATIPDNAGPDRPPRAPVEVDPPVHGDFRDVLNRFFTPDRIRLLEPVIRRTARSLLG
RCIALGETDVVANFTFYMPIQVQCTFLGISPDDGERIKNTINRIIHAGAVGDASEHKKSN
DEIYAYIESIIDARRRTPYDPNDVISALIHEKVGGRTLTDADIAGVIRLFLQAGHGTTTH
ALGSIIRYVGTVPADQKRLREKPGLIPQAIEEILRVWSPVRAVGRKTTCDVEVAGRLIPK
GSKVGLMIASANRDPEAFEDADRVDFDRKPNRHIALGYGIHRCVGAALARAQLRVALEEL
LSMTDDVVVTGEPEFSSWSHNGPSKLMVRLKPATQAAGAPVIRAGHKELSMAVGGIRPVA
TDVIELELRPSSGMELPEWTPGAHIDLVLPGDLRRSYSLVGAPADRSCWRIAVLREATGR
GGSDAIHRMKTGDTMRVRWPTNNFVLRAAPNYHFFASGIGITPILSMAQAARAQGTPFRL
DYIGRSRERLAYLDRVSGLGDVHCHFTSESGRPELGELLGRTDPSAPVYACGSQDFLLAL
EAAATALGRSFHTEWFAPRPGARQAGDGALEAFTVRLERSNVEVGVLPGQSIIDACAEAG
VSIPASCFEGTCGSCLSTVLEGVPDHRDSFLSPNERKSNRLMAACVSKSMTAKLVLDW

>EGT29_25055|KEGG|CYP116B100|[Pigmentiphaga sp.]pig|BP

MSAPPPRAGGCPIHHDAASAGVGPTGCPVSARAAGFDPFEDGYQQDPPEYVRWAREQEPI
FYSPRLGYWVVTRYEAIKAIFRDNITFSPSIALEKITPTGPEANAVLEQYGYAMNRTLVN
EDEPAHMPRRRVLMEPFTPEHLKHHEPMVRRLAREYVDRFVDDGRADLVDQMLWEVPLTV
ALHFLGVPEEDMDTLRRYSIAHTVNTWGRPEPEEQVAVAHAVGNFWQYAGKVLEKMRQAP
DQPGWMRYGIRKQQEHPEVVTDSYLHSMMMAGIVAAHETTANATANAIKLLLQHPDAWRE
ICEDPGLIPSAVEECLRHNGSVAAWRRLVTRDTRIEGVDIPAGSKLLIVTSSANHDDRHF

ADADFFDIRRENASDHLTFGYGSHQCMGKNLARMEMQIFLQELTRRLPHMRLADQRFSYV
PNTSFRGPEHLWVEWDPARNPERQDATLLQIQPPVRIGEPSRHAIARSVVVESATPVADG
IVKLRLVSPDGRALPRWAPGSHIDVECGEPERSRQYSLCGDPADAGALEIAVLHEPEGRG
GSSWIHGRVRVGDKLRIRGPRNHFRLDETAEKLILVAGGIGITPIAAMARRARELGMDYE
LHYSGRSRAGMAFLDELATLHGERLHVYVSEEGQRNDLAALLGQPREGWKICACGPKRML
QALEAHCAAWPDDALRIEHFESNLAQLDPAREHAFEVELKDSGITVTVPADRTVLASLRA
ANIDVPSDCEEGLCGSCEVRVLAGQVDHRDMVLTKAEREANDRMMTCCSRACAGKLVLEL

>LSG25_13615|KEGG|CYP116B301|[Paralcaligenes sp.]park|BP

MTNAIHHKLPSACPIAHVAGAVPKPSTGCPISQHAADFDPFQDAYMENPSEFIRWAREKE
PVFYSPKLDYWVVTRYQAIKNIFRDPITFSPSNVLEPIEPLSNEAAAILKHYGYAMNRTL
VNEDEPAHMARRRVLMAPFTPEHLKQHEPMVRRLVTEAIDQFMDEGHVDLFQKLLWDVPF
SVALHFLGIDDDSDREKMHRFSIAHTVNAFGRPTPEERESVAHTVGQFWQLAGEVLEKMK
RTPDGPGWMRYSIRQQKDYPDVVTDSYLHSMMMAIIVAAHETTSFASANAIKMLLEHPEA
WQDLCADPGLISPAVEECLRHSGSIASWRRKVMSDVEIEGVRIPAGSKLLLVVASANHDN
KQFSDPDFFDIRRDNTVEHLTFGFGAHQCLGKNIGRMEMQIIIGELTRRLPHMRLAQQSY
KYVHNLSFRGPRNLLVEWDPNLNPERLDSVALQQVQPVRLGAPLAKNMVRKLVVESAEKV
APDTVLLRLASPTGSPLPNWTPGAHLDVECGDTGLSRQYSLCGDLENRETWQIAVQRDPN
SRGGSAWIHDYAKPGATLRIRGPRNHFRMSEASTDRIVLIAGGIGITPIMAMAQHAQSLG
IDYEIHFSGRSRAALPLIENLQRRHGVRLRLYISEEGHRNNFRELLAQPNATTQIYACGP
SRMLDDLQNVLVQQGWPDRALHVEHFINTANRLDPDREMAFEVELRNSGLTLQVPANKTL
LEVLRASNIDVQSDCEEGLCGACEVGVVSGEIDHRDSVLSLTEKQKSTRLMACCSRGLSK
PLVLDL

>LSG25_18200|KEGG|CYP107DG11|[Paralcaligenes sp.]park|BP

MINREFLNDPYPAYRALHAAGPLHWNREFCGGAWLLVRHADVASVLRDPRFSVRRAGGWV
NSSGLEARNELRAFKRIFARSILFLDAPRHARLRQIMSAGFKPAALQALIPQIQAIADGL
LDQIIAGVQSGETETQGSFETTQFDFMHDFARPLPALVIAAMLGIDAADRNDFVAWSDDI
AGFIGSPTPSIETARRAQAGLMAMSDYFRGLLAQRRLQPRNDLISQLIVAEADGGIATSK
ELLAQCCTLLFAGHETTRNLLGNGMLALLAHPRQWQLLQQTPSLLPSALKELLRFDSPVQ
YTGRRLKVDVEMHGRLMKKGELVIPLIGAANRDPAKFSAPDRLDITRNEGSHLSFGYGPH

VCLGATLTYLEAEIALRSVMRRLPKLRLAGDVQSWGLNAVYRSLNQLPLRFECRSKTQAS
QEQVSHA

>Rfer_0298|KEGG|CYP203A5|[Rhodoferax ferrireducens]rfr|BP

MFSFDPYAPAVDANPFPFYKTLRDEHPCFWSPEADMWILSRYADIVSAGQDAQTYSSASG
NLMTELPGRAGATLGTTDAPRHDRLRGLIQHAFMKRNLESLSEPIRDIAREVAESLRGKK
QFDFISEFSSKFTVRVLFAALGLPTGAEVTVPESTVRDKAVMMVQSDPVTRTKGPEHIAA
YNWMQDYAAGVIAERRASPRNDLISHFSMAEIDGDKLDEREVLLTTTTLIMAGIESLGGF
MAMFALNMADFADARRQCVANPALLADAVEESLRFNTSAQRFRRRLMKDVTLHGQTMKAG
DFVCLAYGSGNRDERQYPNPDAYDITRKPRGHLGFGGGVHACLGTAIARLAVKIAFDEFH
KVVPEYHRVQEQLPWMPSSTFRSPLVLELAVV

>Rfer_4141|KEGG|CYP289A18|[Rhodoferax ferrireducens]rfr|BP

MSNMRSPDWDPKSAAVLRDQRAACDEMRERCPVAYSDLLGWSLFRHEDVVRVLNDPDTFS
NVVSRNLSVPNGMDPPEHGQYRRIIERYFAPQTMQAFEPQCSSIAADLVRSLRGRVALEL
MGDFAHPFAVRVQCAFLGWPASMHEPLRLWAQKNHVATLAQDRPAMADIAHEFSGYVLGL
LQPRRDAGIQGHQDVISSLTSQQIKGRPLNDEEIISILRNWTVGEISTIAAAVGVVAQYL
AEHTELQQQLRTQPTLLPDAIEEILRMYGPLLANRRVTTRTVEIGGRTINAGERISLNWV
AANRDGLVFTDPQSFRLDRDASANLLYGAGIHVCPGAPLARMELRVAVRALLDGTTSIDP
IAQRPPTSAVYPASGFATLPLHLTFPSQPSTHQR

>EUB48_06975|KEGG|CYP289A27|[Rhodoferax sediminis]rhf|BP

MNDERKCDWDPRSEAVMRDQRAAYDEMRERCPVAYSEFMQWSLFRHEDVSRVLHDHKTFS
NAVSRHLSVPNGMDPPEHTKYRRIVESYFTVQRMDAFEPVCRQLVAELVRDVLARGEVEL
MADFALPFAVRVQCAFLGWPPMLRERLVRWTRKNYEATLAQDLQAMSEIAREFEGFIDGM

LETRMQAGVRPEDDVIGALTHEKVWGRPLTNEEIASILRNWTVGEIGTISASVGILIHYL
SQHVELQQRLRTQPLLLPAAIDEILRIHGPLVASRRVATRKVEIGGRKIDAGERISLNWV
SANRDGRVFEDPQAFRLDRDPAANLLYGAGIHVCPGAPLARMEMRVCLEQLLERTTRIQD
APGKAPSLAVYPASGFATLPLQVR

>EUB48_07025|KEGG|CYP195A28|[Rhodoferax sediminis]rhf|BP

MSSGIPSMIITPAMTPERARKIADTFDLRALPPDFYANPYPVYAALREHEPARRMPDGSY
FLTRYADLVAVYRDAQTFSSDKKAEFGPKYGQASPLFEHHTTSLVFNDPPLHTRVRQLIM
GALTRRAIDSMEAGLVTLVDGLLDRIEAQGGGDLVGDFAAAIPVEIIGNLLGVPHADRGP
LRGWSLAILGALEPALTPEQEALGNRSVSEFGAYLKNLVAERRRHPGDPEHDVLTRLIHG
DGSDTHEARAGSEPLSETELLQNCIFILNAGHETTTNLIGNALVALQEWPDAKHRLLSKM
KLSAQNPQGLEADLTLAVDEFLRFESSNQLGNRRALKACSVGGIQLEAGTLITLGIGAAN
RDPAQFADPDVLNLARGNGRHGAPGSKHLAFGVGIHQCAGLSLARLEGRIAISRFLQRFP
DYRLTQMPTRGGRARFRGFLHAPFASA

>EUB48_13810|KEGG|CYP1097A17|[Rhodoferax sediminis]rhf|BP

MEPDTRRTPASGAPPPALRRLDELPGPRGLPVLGNALQIRPARMHQQFEAWSKTYGPLFR
VQLGRRKFLVMADHELMGAALRERPERFRRPLLGGRITREMGFDEGLFFANDDVWRRQRR
MVMAGLDPGHIKAYFPSLLKVTRRLQARWQTAARSGATIDLQADLMRFTVDAIAGLAFGA
DVNTLSSDEDVIQQHLNKIFPALFKRTLSMLPYWRYLRLPADRALDRSVAEVKAAIRGFI
AQARDRIRADPSLREHPGNLLEAMINAADAGGSSMNDHDVAGNVLVMLLAGEDTTANTLA
WMIHLLHRNPPALQRAGEEVRRIAGEPSQFTPERMAQLDYLEACAHETMRLKPVAPLLAV
QAVRATTLADVEIPTDTVVLGLMRHDSLLDRHFPHPTAFLPERWLGDGGPAQVASSAKRV
SMPFGAGPRVCPGRYLALLEIKMAAAMLLSSFDIDSVDSSSGTDVPEKLAFSMGPVGLRM
RLRERR

>EXZ61_03070|KEGG|CYP212B5|[Rhodoferax sediminis]rhg|BP

MNPASTSSPSISAQTSALTRPPGPPSRWWGLPLLAAMKRDYLGFTTQLQRDHGDISYMRL
GHERAYDLMSPDLVREALVSHADALIRWERGIEVFAQVFGQSVLVTEGPVWQRQRRMLMA
AFMPKQVAGYATLMTSAAQDAFAQLIPPGQTSARIPMDALWSSVAMDVILRTLFSRSAQA
DSQAAIAATQALSAAAFQEMFLPFSLPDWLPLPGKAAKRRAIRSLRQLIQRHINERHAEL
AAHPDAPAATDLLGRLLALRDETTGEALSAQEVFDQCMVSFQAGHETSATALLWWSRLMA
EHPDLAQRAHDEVATVLAGQTPGPEHMAQLPWLTATLKEAMRLYPPVAAIMSRRTTAPIT
VGGWQIPQGAMLRITPWLLHHDERWFANPEQFQPARFLDGAPAIPKGAWMPFGAGPRVCL
GQHFAMMEMTLLAAMLLQRYRLRLPADAAPSTPELHVTLRPRAPMELMLSHL

>Bpro_1581|KEGG|CYP107DG2|[Polaromonas sp.]pol|BP

MNADLRPPAPAYPIIDAAFLANPYPTYDALREAGAIHWSEEFFGGAWLVTRHADVELVLR
DPRFSAQRTGAWVKDREETSGELSGFQQLFARALLFLDAPDHPRIRKVLHAGFRMDVLQR
LVPHIEQAVTELLDQGEGADCFDFIETVARPLPVRVITRLLGIENLQYDDFMAWSEDLAS
FIGAPQPTRGQARRAQVSLLAMSRYFETLLEQKRQTPGDDLTSRLAQAEARGEIQGGAEL
LAQCAMLLFAGFETTRHLLGSGLQALLAHPDQWQRLQQAPELLPGAVRELLRFDSPVQYT
GRRVSTDLVLHGQQLRRGDLVVPLIGAANRDPRRYAQPDVLDITRRDGSSLSFGSGPHVC
IGAALTLMEAEIVFQQLLRRWPELSLVDATPRRITNPLYRGLVTLPLRCRAPA

>Bpro_2807|KEGG|CYP1180A1|[Polaromonas sp.]pol|BP

MSDGVLPQPGNPIEAVTHVDPYPYYRQLREQRPLYFDEGLKLWVASSHSVIDEAFHNVAL
RVRPPAEPVPQALLGTAAGEVFAQLVRMTDGEFHAVHKPAVEQAARRWSLADVTRASEAA
ARDLFAAGPPQGKHGPLGGQRSVGAKFRCLTVNDLMGVLPVQAMARLLGLPDGLLDDSCR
WVRQFVQGIAPGASTQAVALAAGAAEALMDQGRALGLSTVQAANRIAFMQQSLDATAGLM
GHTVLMLAQYPDLALLADQSPQAMRGFVAEVERHQAPIQNTRRFAADPVVLAGQSIEAGQ
GVVLVLASANRDGLLNPRPDQFDPQRGERRSMGFGAGAHACPGAAIAIEMVAACIQWMRA
TSQFDGCFGALAGFWPLANARIPLFASRKEA

>Bpro_4920|KEGG|CYP1098B1|[Polaromonas sp.]pol|BP

MSHFCPGYPKPREKRSSVWALFRSARNSWIDALYARSYSMQMGEVHLPGVDLYMVNDPAL

VRRMLSGQPDQFPKSPLLADALRPLLGDSILTTNGDQWQRQREMMNPAFAQAKLDVAFPV
MRAAADDLVERLTEAAQRGSELNIEVELTHVTADIIYRTIFSEPLSGDDAHKVFDAFARF
QALAPKLMLPSMYGVRWLVWPWNVWQSRRAASDIRRLLEKLIRSRHEAWLRGDDLGKNDI
LSALMTSRDRKTGEPFSFDELVDQVAVLFLAGHETSASALTWAVYLLSAVPEVQERVHQE
TCRVFGQRTPEQRDMKALVLTRNVFRETLRLFPPVGFMARESAQTCPMRDKMVPKGASVM
ISPWLIHRHRERWSDPDAFNPDRFDDDASRESIRQSYLPFGMGPRVCLGAAFALQEATLI
LASLIRAFRLETVTGHTPMPVGRLTIRSDNGVRLRIFKREDQ

>Bpro_5301|KEGG|CYP153A76|[Polaromonas sp.]pol|BP
MSEAIVVNNQNDQSRAYAIPLEDIDVSNPELFRDNTMWGYFERLRREDPVHYCKDSLFGP
YWSVTKFKDIMQVETHPEIFSSEGNITIMESNAAVTLPMFIAMDPPKHDVQRMAVSPIVA
PENLAKLEGLIRERTGRALDGLPINETFDWVKLVSINLTTQMLATLFDFPWEDRAKLTRW
SDVATALVGTGIIDSEEQRMEELKGCVQYMTRLWNERVNVPPGNDLISMMAHTESMRNMT
PEEFLGNLILLIVGGNDTTRNSMTGGVLALNENPDEYRKLCANPALIASMVPEIVRWQTP
LAHMRRTALQDTELGGKSIRKGDKVIMWYVSGNRDPEAIENPDAFIIDRAKPRHHLSFGF
GIHRCVGNRLAELQLRIVWEELLKRWPNPGQIEVVGAPERVLSPFVKGYESLPVRINA

>Pnap_4029|KEGG|CYP107DG3|[Polaromonas naphthalenivorans]pna|BP

MNAVLTPPAGALGRPIVDAAFLADPYPTYRALSEAGPIHWSDEFFGGAWLLTRHADVEKV
LKDPRFSAQRTGAWVKDREDAPGELSSFQKLFSRALLFLDAPDHTRIRKVLNAGFRPEVI
RGLAPYIEQAVGELLDRAEAASARNTGNTPFDFMQAVARPLPVRVIARLMGIEASAQDDF
MVWSDDLATFIGAAQPGREQARRAQASLLAMCRYFEALLPRRRQAPGDDLVSRLVLAEAA
GEIEGGPELLAQCAMLLFAGHETTRNLLGNGLQALLSHPAQWQKLQQSPELLPGAVRELL
RYDSPVQYTGRRVTTELVLHGQLLRRGDLVLPLIGAANRDPARYAQPDRLDIARRDGGSM
SFGSGAHVCIGAALTLMEAEIVFGQVLRRWPGLCLAGAAPQWGSNPVYRGLLALPVRNVA
AC

>DT070_17790|KEGG|CYP229M1|[Polaromonas sp.]pos|BP

MAFMTPHAKPETAPEHILAASAHRNPYPYYASLVAGPALVFDAPRKLWIAGSAASVKAVL
GNPACHVRPAAEPVPAAIVGSAAGEVFGHLVRMNEGLRHDAPKLALQRFLAGVDLGRVKA

RTEVLAATQAGQGPLHGSALSRWAFEVPVMAVADMLGFAEDELPQVARWMGDFVACLSPV
STAAQLAASADAASALLARFAELVRDANAHPGSALARLQYEASQVGWRDARALLANAVGL
LSQTYEATAGLIGNSIVALATQPALLDAMRREPGKLLQMVNEVSRFDPSIHNTRRFVAEA
TQVAGVSLQPGDVVLLVLAAAGRDPLCNARPDEFLLDRPDRDVPGFGRGAHQCPGQALAC
QIAAAAVKTLLVSGRLQAASSFSWQYRPSMNARIPVFVD

>DT070_20855|KEGG|CYP195A26|[Polaromonas sp.]pos|BP

MTDALPFAITPAMPAARAMEIAAGFDLRALPVDFYANPYPVYAALRENEPIRRMPDGSFF
LTRHADAVAVYRDAQTFSSDKKVEFEPKYGAGSALFEHHTTSLVFNDPPLHTRVRKLIMG
ALTRRAIADMEPGLVMLVDSLLDAVEAKGGGDLIEDFASAIPVEIIGNLLGVPHADRGPL
RAWSLAILGALEPKLTPEQEALGNQSVRDFTGYLKDLVADRRKHPGDPEHDVLTRLIQGE
TAGEKLNETELLQNCVFILNAGHETTTNLIGNALVLLQEWPDAKRHLLSKVEQQPNAVIG
PEADLTVEIDEFLRFEPSNQLSNRRALKACQVGGVDLPEGALLTLCIGAANRDPAQFANP
ETLDLTRADNRHLAFGFGIHQCAGLSLARLEGRIAVGRFVQRFPDYRLTASPTRGGRARF
RGFLKAPFAV

>F7R28_09720|KEGG|CYP195A26|[Polaromonas sp.]poo|BP

MTDALPFAITPAMPAARAMEIAAGFDLRALPVDFYANPYPVYAALRENEPIRRMPDGSFF
LTRHADAVAVYRDAQTFSSDKKVEFEPKYGAGSALFEHHTTSLVFNDPPLHTRVRKLIMG
ALTRRAIADMEPGLVMLVDSLLDAVEAKGGGDLIEDFASAIPVEIIGNLLGVPHADRGPL
RAWSLAILGALEPKLTPEQEALGNQSVRDFTGYLKDLVADRRKHPGDPEHDVLTRLIQGE
TAGEKLNETELLQNCVFILNAGHETTTNLIGNALVLLQEWPDAKRHLLSKVEQQPNAVIG
PEADLTVEIDEFLRFEPSNQLSNRRALKACQVGGVDLPEGALLTLCIGAANRDPAQFANP
ETLDLTRADNRHLAFGFGIHQCAGLSLARLEGRIAVGRFVQRFPDYRLTASPTRGGRARF
RGFLKAPFAV

>F7R28_12795|KEGG|CYP229M1|[Polaromonas sp.]poo|BP

MAFMTPHAKPETAPEHILAASAHRNPYPYYASLVAGPALVFDAPRKLWIAGSAASVKAVL
GNPACHVRPAAEPVPAAIVGSAAGEVFGHLVRMNEGLRHDAPKLALQRFLAGVDLGRVKA
RTEVLAATQAGQGPLHGSALSRWAFEVPVMAVADMLGFAEDELPQVARWMGDFVACLSPV

STAAQLAASADAASALLARFAELVRDANAHPGSALARLQYEASQVGWRDARALLANAVGL
LSQTYEATAGLIGNSIVALATQPALLDAMRREPGKLLQMVNEVSRFDPSIHNTRRFVAEA
TQVAGVSLQPGDVVLLVLAAAGRDPLCNARPDEFLLDRPDRDVPGFGRGAHQCPGQALAC
QIAAAAVKTLLVSGRLQAASSFSWQYRPSMNARIPVFVD

>Ajs_3072|KEGG|CYP1318A1|[Acidovorax sp.]ajs|BP

MAQGHLAPGPDQGYDLDANDETLPVLQQLRSRYGPICRVPSLTRPGDGLVIHDPDDIRRV
LLTNRGNYVKGAGLERVRVLLGNGLIVSDGDLWVRQRRMIQPAFQGQATRGFAPVVRQVN
ADLLTRWSAHADSGEPIDLTHELSSVALEIVLRALFSADFDRLVETEGGSPFDLLTEESR
RDLQFAARFRGLARFVRAIIETRRLEARVESDWLSMLMQARDKASGEPMPDRALLDEVMT
LIVAGHETTASTLNWTWYLLARHPEAEAALHAAIANAAPTEAAPDQALATPFASADYVEQ
VLQEALRLYPPVWLFSRRAVQDDTLGGYHVPAGTDIFICPYLLHRDAAQWERPEEFLPAR
FMPDAAAGRHRFAYLPFSAGPRFCVGAGFAMAEMATHLTMVAERFRWVPVDAEPAEAEFQ
INLRTRHPLRMRLVSRR

>Ajs_3226|KEGG|CYP289A19|[Acidovorax sp.]ajs|BP

MTTTPPRWDPQAPEVLRDQRAAYDQLRERCPLAHSDAQHWSVLRHAEVMQVLGDHATFSS
AVSAHPAVPNGYDQPLHTQYRRALDPFFAPERMAHFEPVCRALSAATVQEALALGGELEI
THEFALPFAVRVQSAFLGWPTLLQDELLGWLRRSQAATRSGDRTQTSAVAQEFEDLVGRV
RQARLEAPPTQDITSELMHTRVEGRLLDLRELASVLRNWTVGEVGTIAASIGILLHWLAS
HPDWQQRLRAQPGLLPTAIDEVLRIDGPLVANRRITTRDVTLGGCPVPMGERLSIHWIAA
NRDPRVFGDPDEFRMDRDPALNLLYGAGIHVCPGAPLARLELRVLMEELLARTSAIALPA
GHAPTRATAPDAGYAALRVRLHQTPA

>Dtpsy_2579|KEGG|CYP289A19|[Acidovorax ebreus]dia|BP

MTTTPPRWDPQAPEVLRDQRAAYDQLRERCPLAHSDAQHWSVLRHAEVMQVLQDHATFSS
AVSAHPAVPNGYDQPLHTQYRRALDPFFAPERMAHFEPVCRALSAATVQEALALGGELEI
THEFALPFAVRVQSAFLGWPTLLQDELLGWLRRSQAATRSGDRTQTSAVAQEFEDLVGRV
RQARLEAPPTQDITSELMHTRVEGRLLDLRELASVLRNWTVGEVGTIAASIGILLHWLAS
HPDWQQRLRAQPGLLPAAIDEVLRIDGPLVANRRITTRDVTLGGCPVPMGERLSIHWIAA

NRDPRVFGDPDEFRMDRDPTLNLLYGAGIHVCPGAPLARLELRVLMEELLARTSAIALPA
GHAPTRATAPDAGYAALRVRLHQTPA

>C380_19955|KEGG|CYP212B1|[Acidovorax sp.]ack|BP

MRADYLGFVTQLQREHGDLTRMRLGYEDAWDLMHPDLVREALVTHADQLIRWERGMEVFE
EVFGQSVLVTEGDTWQRQRRMLMPAFTPKRVAGYAQLMTDAARSALDTAVPPGQPGAQVA
VDALWTDVAMDVILRTLFSTSAQADAREAAWATQTLSEAAFHEMFMPFTLPDWLPLPGKA
AKRRAIRSLKGLVWRHIHARQAEVAAPGAPERSDLLHMLLALRDEQTGEALSAQEVFDQC
IVSFQAGHETSATTLLWWTLLMAQHPEAAQRAQAEVDAVLQGGTPGPEHLAQLPWLGATL
KEALRLYPPIAALMSRRTTAPITLGGVPVPQGAMLRITPWVLHRDARWFAEPDRFVPERF
LDGAPPIPRGAWIPFGLGPRVCIGQHFAMLEMTLLAAMLLQRYTVRLPEGGAVGAPRFQV
TLRPEGTSALWLQRRDAPQGR

>BSY15_2248|KEGG|CYP212B2|[Acidovorax sp.]acra|BP

MSNSFNTGLLLRCTMHSMHHVTTTVPSKPATPSPSAPLAPNRPLQPGGPPSRWWGLPLLW
AMRADYLGFVSRLQREHGDLTRMRLGNEDAWDLLSPDLVREALVTHADQLIRWERGIDVF
EQVFGQSVLVTEGATWQRQRRMLMPAFTPKRVAGYAQLMTDAARSALDAAVPAGQAEALV
PMDTLWTDVAMDVILRTLFSESAKADARDAAWATQTLSETAFREMFMPFTLPDWLPLPGK
AAKRRALKALKGLVQRHIGARSREAADSAGAPPRTDLLHMLLALRDESTGEPLSPQEVFD
QCMVSFQAGHETSATTLLWWSRLMAEHPEAAQRAQAEVDSVLPPGQTPGPEHLAQLPFLS
ATLKEALRLYPPIAALMTRRTTAPITLGGVEVPQGAMLRITPWVLHRDERWFTQADRFVP
ERFLDDAPAIPKGAWIPFGVGPRVCIGQHFAMLEMTLLAAMLLQRYRLRLPEGGRASVPK
LHVTLRPEGPMHLWLVRR

>EAG14_04345|KEGG|CYP212B7|[Acidovorax sp.]acio|BP

MAAVADPADVPRPLQLSGPRSPWWGLPLLRDMRADYLGFVTRLHQQHGDLTRMRLGYEDA

WDLMHPDLVREALITHADQLIRWERGIEVFEQVFGQSVLVTEGATWQRQRRMLMPAFTPK
RVAGYAQLMTDAARRALDAAVPAGQGRALVPVDALWTDVAMDVILRTLFSESAQADARDA
AWATQTLSEAAFREMFMPFTLPDWLPLPGKADKRRALRALKGLVQRHIDARQSEAPVADG
ASGKPERSDLLHMLLALRDESTGETLSPQEVLDQCLVTFQAGHETSATTLLWWTTLMAQH
PEAAERARAEVDAVLQGGVPGPEHLAQLPWLGATLKEALRLYPPIAALMNRRTTAPITLG
GVAVPQGALLRITPWVLHRDARWFAQPEQFQPERFLDGAPPVPKGAWIPFGLGPRVCIGQ
HFAMLEMTLLAAMLLQRYTVRWPDAAPACAPRLNVTLRPEERVFVWLERRG

>HUK68_16805|KEGG|CYP110J3|[Acidovorax antarcticus]aant|BP

MKRGPLPPGPQWLRPLSFFQAGLTPPAPVLARLAARHGDPFRVSYLSRPVTFTGHPDALR
AIYTASPDCFEVWDGSFIEPVFGPASVVVTSGDRHRQDRNLLAPAFSGPAMRADGAVMAA
VAEEKMAQIAVGQPFRMLDVTQRITLDIMIRVVFGMQDETRIARTRAAVLELIESLHVIL
FLFPSARRVLSGWGPWARNQRAVEALGALLREQMRERRLGAASGADLLGRMLAARYDDGS
GMDDAQILDQLRGLLFAGHEATATVLAWIFHWLGREPAVLARVAQEIKDLGPAPAPDALA
ALPYLEMVCKECLRLYPPVVDSARVVRKPFALLGYTIPAGEVLRASSALLHARPALYPEP
ERFRPERFAERKYSSFEYIPFGGGARRCLGAAFAMYELKVVVASIVRGHRLRLLDKQPPV
HERRALSLGPRGGVPMVVQERRSLPHR

>Daci_3643|KEGG|CYP212A16|[Delftia acidovorans]dac|BP

MGWGLLRRMSRDLLGTLAQWRGEHGDAIHLRIWPEHQVVVTDPELVRELLVGQHDALVRW
EHGISVFAQLHGHSVLVSEGQPWRSKRQALLPAFSPKAVQAQIPALAAAAQAALQAWPQQ
SEDFPMEQALTALAMDVIMRLMFSGAIGEDARSAEQAIREVSHAANSEFYWPLRWPDAMP
WKRRKRRALAWLRGFIARQVRLRLAQADADWPEDLLSRLLRLHRGDAQAWTLQAVRDECM
TAFLAGHETVASSLTWWSWCMATHPEAQQQVADEIQEQLQGRTPTAQDLPALRQLGWSLQ
EAMRLYPAAPVLISRRCTRPVQLGPWRLPARTLFMIAPGLMQRDARWFPQPDAFQPQRFA
DADKDGAPAAPRGSWMPFGTGPRVCLGQHLASTEMTVVAAMVLQRLRLSVPEGASAPRPV
LQVTLRPSTPLRLRLQSRSGAQAG

>BO996_16530|KEGG|CYP212A16|[Delftia sp.]dhk|BP

MSRDLLGTLAQWRGEHGDAIHLRIWPEHQVVVTDPELVRELLVGQHDALVRWEHGISVFA
QLHGHSVLVSEGQPWRSKRQALLPAFSPKAVQAQIPALAAAAQAALQAWPQQSEDFPMEQ
ALTALAMDVIMRLMFSGAIGEDARSAEQAIREVSHAANSEFYWPLRWPDAMPWKRRKRRA
LAWLRSFIDRQVRLRLAQADEDWPEDLLSRLLRLHRGDAQAWTLQAVRDECMTAFLAGHE
TVASSLTWWSWCMATHPEAQQQVADEIQEQLQGRPPTAQDLPALRQLGWSLQEAMRLYPA
APVLISRRCTRPVQLGPWRLPARTLFMIAPGLMQRDARWFPQPDAFQPQRFADADKDGAP
AAPRGSWMPFGTGPRVCLGQHLASTEMTVVAAMVLQRLRLSVPEGAGAPRPVLQVTLRPS
TPLRLRLQSRSGAQAG

>I6G47_21230|KEGG|CYP116B17|[Delftia lacustris]dla|BP

MTASAASNAAPSASAPAFAPASARCPFDHRQPSGQTGPTGCPVSAGAAAFDPFSDGYQQD
PPEYVRWAREQEPVFYSPQLGYWVVTRFDDIKAVFRDNIAFSPSIALEKITPTGEEANAV
LASYGFALNRTLVNEDEPAHMPRRRALMDPFTPEELRHHEPMVRELARAYVDRFIDDGRA
DLVDQMLWEVPLTVALHFLGVPEEDMDRLREYSIAHTVNTWGRPRPEQQVAVAHAVGNFW
QLAGKVLAKMRENPDAPGWMQYGIRKQKELPEVVTDSYLHSMMMAGIVAAHETTANATAN
AVKLLLQHPGAWKQICENPALIPNAVEECLRHNGSVAAWRRLATRDVVIGGVAVPAGSKL
LIVTSSANHDERHFADADFFDIHRDNAGDHLTFGYGSHQCMGKNLARMEMQIFLEEFTRR
LPHMRLAEQRFTYVPNTSFRGPEHLWVEWDPALNPERARPALLQARQPVRIGEALQGGAS
RPMVVAEAEAAAEGVLRLRLKAADGHALPRWTPGSHIDLQCGDTGLSRQYSLCGDPQDTG
SYEIAVLREANGRGGSAWVHAHLHRGATVQVRGPRNHFPLDEGASHLILVAGGIGITPIS
AMARRARALGLSYEIHYSGRRRAGMALLAPLQAEHGARLHLYVAEEGRRADYAALLATPR
AGARIHACGPARLLHGLEAASRHWPEDSLRVEHFVSTVGALDPAREQGFEVELRSSGLVL
QVRNDQTLLDALRSAKIEVQSDCREGLCGSCEVPVLDGEVDHRDMVLTRGERAACTKMMS
CCSRARSGRLVLGL

>Vapar_4796|KEGG|CYP102A24|[Variovorax paradoxus]vap|BP

MAGKNSLHPIPHPAKKPFVGNLLSIGSDSPVLDMWKIAQDLGGIYWLDMPGMPVIVVSSP
ALVDELCEEARFDKSTRGALRRLRAASHGLFTSDTHEETWSKPHNILLANFSQRAMQAYH
PMMLDIAGQLVTKWERLNFDEEVDVVRDMTALTLDTIGLCGFGYRFNSFYREGFHPFVDA

MVRTLETVQNRRGLPLEEFMLKKELAQQRKDIRFMHKMVEDIIEERRAGGADIATKPDLL
SYMIAGVDKKSGEKLTDKMIRDECIEFLIAGHETTSGLLSFAIYFLLKNPEALAKAQAEV
DNVFGPDTSQKPTYAQVNRLQYVMQVLKEALRLYPTAPAIAMRAKEDTTIGGQYTIKKKN
MVIMHALALHRDKGIWGEDADEFNPDHFSREAERERPVNAFKPFGNGQRACIGRQFALQE
AVLTLGMILQRFDLVDHTGYKLRIKEALTIKPEGLKIKALLRDPATRPRGNTADAAAAPV
QPRQAAARKPQAARHGTSLLVLQGSNLGTAEDLARQLAEAGELRGFSTQVASLDDYAERL
PANGAVAIVCASYNGVAPDNAAEFHRWLDKADDSLNGVRFSVFGCGNTDWAATYQAVPRR
IDERLGALGATRVHPRGEGDAREDMDGAFQDWSDALWPELVKAFGIQSGADTPAEAEPLY
TLEELPPPQKNALVDALGAVALRVIENRELQKDPGGAGEAGRSTRHVELMLPEGVDYRAG
DHLSVVPRNSPAQVERAMARFGFDRAAHVRLHADSGRKTALPVDQVIAVDRLLGDYVELQ
DVATRKQIATLTAYTECPATRPKLAALSGSDDASQAAYKAQVLHRRRSLLDLLEEHRACQ
LPFAVFLEMLSPLSPRYYSISSSPTMTPGRCSVTVGVVSAPALSGKGTFEGVCSNYLARA
EAGDTVHGVIRETTAEGFRLPEDAQRPLVMIGPGTGLAPFRGFLQERAAQAERGQALGEA
MLFFGCRHPEQDFIYAEELEAWSHRGLMKLHTAFSRAGERKVYVQDLIREQGAAVWKLLE
AGGVIYVCGDGSRMEPDVRRTLADLARGHGHDGAAWMDQMIADQRYVLDVWAGA

>Vapar_4960|KEGG|CYP229B3|[Variovorax paradoxus]vap|BP

MPLDPVAAVTHADPYPYYAELRAGPPLAWNDALHLWVASRAEVIESLLLAHGALRVRLAA
EPVPRAIAGSAAGEVFGHLVRMNDGTAHQAHRPALQRALAGLDLGAVRMAALRVARDVPV
QPVSDALFSMPVRTVAHLLGIADEALPQVDRWMRDFVACLSPLSTAQQLQGASAAAGELM
TRFEELAAGAAPRDGTLLAAVRAESQASAPLSRALLANLVGLLSQTCDATAGLLGNSLIA
VMREPSLQQAAQTPGGLQAIVEETARHDPSVHNTRRFVAEPVTVAGTPLAVGDAVLLVLA
AANRDPALNPEPERFILVRARRRMLGFGHGVHACPGQALACTLAAAGLEALLHRTPDLAA
LRARGWHYRPSVAARIPVFH

>Varpa_5460|KEGG|CYP102A24|[Variovorax paradoxus]vpe|BP

MAGKNSLYPIPHPAKKPFVGNLLSIGSDSPVLDMWKIAQDLGGIYWLDMPGMPVIVVSSP
ALVDELCEEARFDKSTRGALRRLRAASHGLFTSDTHEETWSKPHNILLANFSQRAMQAYH
PMMLDIAGQLVTKWERLNFDEEVDVVRDMTALTLDTIGLCGFGYRFNSFYREGFHPFVDA
MVRTLETVQNRRGLPLEELMLKKELAQQRKDIRYMHKMVEDIIEERRASGADIATKPDLL
SYMIAGVDKKSGEQLTDKMIRDECIEFLIAGHETTSGLLSFAIYFLLNNPEAMAKAQAEV

DSVFGGDTSQKPTYAQVNRLQYVMQVLKESLRMFPTAPAISMRAKEDTTIGGQYTIKKNN
MVIMHALALHRDKAIWGENADQFNPDNFSREAERERPVNAFKPFGNGQRACIGRQFALQE
AVLTLGMILQRFNLVDHTGYKLKIKEALTIKPENFKIKALLRDPASRPRGNGETTAAPDA
PAKPAARKPQAARHGTSLLVLQGSNLGTAEDLARQLAEAGELRGFSTQVASLDDYAERLP
ANGAVAIVCASYNGVAPDNAAEFHRWLDKADDALNGVRFSVFGCGNTDWAATYQAVPRRI
DERLEALGATRVHPRGEGDAREDMDGAFQDWSDALWPELVKAFGIKAGADTPAEAEPLYT
LEELPPPQKNALVDALGAVALRVVENRELQSPGGDAGTSRSTRHVELMLPEGVNYVPGDH
LSVVPRNSPAQVERAMARFGFDRSAHVRLSAAPGRKTALPVDQVIAVDRLLGDYVELQDV
ATRKQIATLAAYTECPFTKPKLVALSGSDEASQAAYKAEVLHKRKSLLELLEEHRACQVP
FAVFLEMLSPLSPRYYSISSSPSMTPGRCSVTVGVVSEPALSGNGIFEGVCSNYLARAEA
GDTVHGVIRETTAEGFRLPEDAMRPLIMVGPGTGLAPFRGFLQERAAQVERGEALGEAML
FFGCRHPEQDFIYAEELQGWSHRGLMKLHTAFSRSGERKVYVQDLIREQAAAVWKLLEAG
GVIYVCGDGSRMEPDVRRTLTDIAREHGHDSNAWMDKMIADQRYVLDVWAGT

>Varpa_5641|KEGG|CYP229B2|[Variovorax paradoxus]vpe|BP

MPVDPVAAVMHADPYPFYAQLRAGPPLMWNEKMRVWVASRADVIESVLLAHGALRVRPAA
EPVPRAIAGSPAGEVFGHLVRMNDGAAHQGHRPALQRALAGLDLNAVHTAALRVARDVPM
APLSDALFSMPMQAVAQLLGFADEALPQVDRWMRDFVACLSPLSTADQLQAASAAAGELM
TRFELLTASAPPRNGTLLAAVLAESGAGTPLSRSLLANLVGLLSQTCDATAGLLGNSLIA
LMREPSLRQAACTRDGLQAIVDETARHDPSVHNTRRFAIEPITIAGTPLAAGDAVLLVLA
AANRDPAFNPEPDRFQLVRERRRMLGFGHGMHACPGQALACTLAAAGLDALLNRQPDLDA
IRARGWSYRPSANARIPVFH

>VAPA_1c37190|KEGG|CYP116B257|[Variovorax paradoxus]vpd|BP

MSDTAIPPHASGCPVAHGPLSSERTPTGCPVSERAAGFDPFEDGYQQDPPEYVRWAREK
PIFFSPKLGYWVITRYDDIKAVFRDNITFSPSNALEKITATGDEANAVLASYGFALNRTL
VNEDEPAHMPRRRVLMDPFTPEALKHHEPMVRELARSYVDRFIDDGKADLVDQMLWEIPL
TVALHFLGVPEEDMDTLRKYSIAHTVNTWGRPKPEEQVAVAHAVGNFWQYAGKVLEKMRQ
DPDAPGWMQYGIRKQKLHPEVVTDSYLHSMMMAGIVAAHETTANATANAMKLLLQHPQVW
RELCEDPGLIPNAVEECLRHNGSVAAWRRLVTRDTQVGGVDLPAGSRLLIVTSSANHDEA

HFSDADLFDIRRDNASDHLTFGYGSHQCMGKNLARMEMQIFLEEFTRRLPHMRLSQQRFS
YVPNTSFRGPEHLWVEWDPAANPERSSPKLRDVQVPVRIGEPSRHAIARPVVVERVTPVA
EGIVKLRLVSPDGKPLPRWTAGSHIDVECGTPELSRQYSLCGDPDEAGVLEIAVLHEPEG
RGGSAWVHAQVKAGDRLRIRGPRNHFRLDESLPKAIFVAGGIGITPVSAMARRAKALGMD
YELHYSGRSRRRMAFLDELAALHGERLHVYARDEGRRCDLPALLAEPVAGAQVYACGPLR
MLEALEGCCAAWPEGALRVEHFESTLATLDPAREHAFEVELKDSGLVVIVPPEQTLLSAL
RAANVDVQSDCEEGLCGSCEVRVLSGRVDHRDVVLTRSEREANNKMMACCSRACESRLVL
EL

>VAPA_1c49620|KEGG|CYP102A24|[Variovorax paradoxus]vpd|BP

MAGKNSLHPIPHPAKKPFVGNLLSIGSDSPVLDMWKIAQDLGGIYWLDMPGMPVIVVSSP
ALVDELCEEARFDKSTRGALRRLRAASHGLFTSDTHEETWSKPHNILLANFSQRAMQAYH
PMMLDIAGQLVTKWERLNFDEEVDVVRDMTALTLDTIGLCGFGYRFNSFYREGFHPFVDA
MVRTLETVQNRRGLPLEEFMLKKELAQQRKDIRFMHKMVEDIIEERRAGGADIATKPDLL
SYMIAGVDKKSGEKLTDKMIRDECIEFLIAGHETTSGLLSFAIYFLLKNPEALARAQAEV
DNVFGPDTSQKPTYAQVNRLQYVMQVLKEALRLYPTAPAIAMRAKEDTTIGGQYTIKKKN
MVIMHALALHRDKGIWGEDADAFNPDHFSREAERERPVNAFKPFGNGQRACIGRQFALQE
AVLTLGMILQRFDLVDHTGYKLRIKEALTIKPEGLKIKALLRDPATRARGNGAHVEAAVA
PSASTKQAARKPQAARHGTSLLVLQGSNLGTAEDLARQLAEAGELRGFSTQVASLDDYAE
RLPANGAIAIVCASYNGVAPDNAAEFHRWLDKADDSLNGVRFSVFGCGNTDWAATYQAVP
RRIDERLEALGAMRVHPRGEGDAREDMDGAFQDWSDALWPELVKAFGIQSGADTPAEAEP
LYTLEELPPPQKNALVDALGAVALRVIENRELQKNPGGAGEAGRSTRHVELMLPEGVNYR
AGDHLSVVPRNSPAQVERAMARFGFDRSAHVRLHADPGRKTALPVDQVMAVDRLLGDYVE
LQDVATRKQIATLAAYTECPVTRPKLAALSGSDEASQAAYRAEVLHKRKSLLDLLEEHRA
CQLPFAVFLEMLSPLSPRYYSISSSPAMTPGRCSVTVGVVSAPALSGNGTFEGVCSNYLA
RAEAGDTVHGVIRETTAEGFRLPEDAQRPLVMIGPGTGLAPFRGFLQERAAQVERGEALG
DAMLFFGCRHPEQDFIYAEELEAWSHRGLMTLHTAFSRASERKVYVQDLIREQGAAVWKL
LEAGAVIYVCGDGSRMEPDVRRTLADLAREHGHDGAAWMDRMIADQRYVLDVWAGA

>VAPA_1c51480|KEGG|CYP229B3|[Variovorax paradoxus]vpd|BP

MPLDPVAAVTHADPYPFYAALRAGPPLAWNEALRLWVASRAEVIESLLLAHGALRVRPAA
EPVPRAIAGSAAGEVFGHLVRMNDGTAHQAHRPALQRALAGLDLGAVRTAALRVAHGVPV
QPLSDALFSMPVRAVAHLLGFDDEALPQVDRWMRDFVACLSPLSTAEQLQDASAAAGELM
ARFEALAAGTPPRQGTLLAAVLAEGEASAPLSRALLANLVGLLSQTCDATAGLLGNSLIA
VMREPSLQQAAPTRGGLQAIVEETARHDPSVHNTRRFVAESVTLAGTPLAAGDAVLLVLA
AANRDPALNPEPERFSLVRARRRMLGFGHGIHACPGQALACTLAAAGLEAVLHRTPDLAA
MHERGWHYRPSVAARIPVFH

>AX767_08380|KEGG|CYP102A27|[Variovorax sp.]vaa|BP

MAGNNALYPIPHPSKKPFVGNLLSIDSESPVMDMWRIAQELGGIYWLDMPGMPVIVVSSP
ALVDELCEEARFDKSTRGALRRLRAASHGLFTSDTHEPTWSKPHNILLANFSQRAMQAYH
PMMLDIAEQLVTKWERLNFDDEVDVTRDMTALTLDTIGLCGFGYRFNSFYREGFHPFVDA
MVRTLETVQDRRGLPLEEFMMKKQLTQQRKDIRYMHTMVEDIIKERRESGADIASKPDLL
SYMIAGVDKKSGEKLDDKMIRDECIEFLIAGHETTSGLLSFATYFLLKNPDVMARAQAEV
DSVFGTDLSVRPTYAQVNRLQYVLQILKEALRLYPTAPAISMRAKENTTIGGKYTIHKKN
MVIMHALALHRDKGIWGENADQFNPDNFTREAERTRPANAFKPFGNGQRACIGRQFALQE
AVLTLGMVIQRFKLIDHTDYKLKVKEALTIKPEGFRIKVVPREAAALRARPSTAIADTAA
SQVAGALAVPLAASRASRHGTSLLVLHGSNLGSAEDLARQLAEAGEMRGFSVTLASLDDY
AERLPAAGAVAIVSASYNGTAPDNAVEFWRWLERADGSLNGVAFSVFGCGNTDWASTYQS
VPRRIDERLAELGAERVHARGEGDAREDMDGAFQDWADALWPVLSKRFDLEAEAPIQAEP
LYAVEALPPPQTSGLVESLGAVPMRVVANDELQTTGGETPSGRSTRHVELALPEGVNYRA
GDHLSVVPRNGAAQVERAMARFGFERDAHVRLHAAQGRKAFLPVDEVIAVERLLGDYVEL
QDVATRKQIATMATHTRCPVTQKAFAALTGSDEATAAHYKREVLVPRKSVLDLMEEFPVC
ELPFAAYLEMLSPLSPRYYSISSSPRVDAGRCSVTVGVVREAARSGRGTFEGVCSNFLAR
VEPGDTVHGFVRATTAEGFGLPDDPSKPIIMVGPGTGLAPFRGFLQDRAAQKTAGATLGD
AMLFFGCRHPQQDFIYAGQLQAWAKEGLVDLHTAFSRAGGRKAYVQDLIREQADAVWKLL
EAGATVYVCGDGSRMEPDVRRTLADIAREHGEDSDAWMDKMLSTQRYVLDVWAAS

>CKY39_29690|KEGG|CYP102A23|[Variovorax boronicumulans]vbo|BP

MAGKNSLHPIPHPAKKPFVGNLLSIGSDSPVLDMWKIAQDLGGIYWLDMPGMPVIVVSSP
ALVDELCEEQRFDKSTRGALRRLRAASHGLFTSDTHEETWSKPHNILLANFSQRAMQAYH
PMMLDIAGQLVTKWERLNFDEEVDVVRDMTALTLDTIGLCGFGYRFNSFYREGFHPFVDA
MVRTLETVQNRRGIPLEELMLKKELAQQRKDIRYMHKMVEDIIEERRTSGADIATKPDLL
SYMIAGVDKKSGEKLTDKMIRDECIEFLIAGHETTSGLLSFAIYFLLNNPEAMAKAQAEV
DSVFGGDTTQKPTYAQVNRLQYVMQVLKESLRLYPTAPAISMRAKEDTKIGGQYTIKKNN
MVIMHALALHRDKGIWGEDADQFNPDHFSREAERERPVNAFKPFGNGQRACIGRQFALQE
AVLTLGMILQRFNLVDHTGYKLKIKEALTIKPENFKIKALLRDPASRPRGTVEAAATPDA
PAKPAARKPQAARHGTSLLVLQGSNLGTAEDLARQLAEAGELRGFSTQLASLDDYAERLP
ASGAVAIVCASYNGVAPDNAAEFHRWLDKADDSLNGVRFSVFGCGNTDWAATYQAVPRRI
DERLEALGADRVHERGEGDAREDMDGAFQDWSDALWPALVKTFDIKNGADTPAEAEPLYT
LEELPPPQKNALVDALGAVPLRVIENRELQSAGANGEAGRSTRHVELMLPEGVNYVPGDH
LSVVPRNSPAQVERAMARFGFDRSAHVRLSAVPGRKAALPVDQVIAVDRLLGDYVELQDV
ATRKQIATLAAHTECPFTKPKLQALSGSDEASQAAYKAEVLHKRKSLLELLEEHRACQVP
FAVFLEMLSPLSPRYYSISSSPTMTPGRCSVTVGVVSAPALSGNGTFEGVCSNFLARAEA
GDTVHGVIRETTAEGFRLPEDANRPLIMIGPGTGLAPFRGFLQARAAQVERGEALGEAML
FFGCRHPEQDFIYAEELQAWSHRGLMKLHTAFSRAGDRKVYVQDLIREQGAAVWKLLEAG
AVVYVCGDGSRMEPDVRRTLSDLAREHGQDSAAWMDQMIAEQRYVLDVWAGA

>C4F17_21040|KEGG|CYP229B4|[Variovorax sp.]vam|BP

MDRNNSPTPPDATLDPVAAATHADPYPFYASLRNGPPLAWNEKLRLWVASRAEVIEEVLQ
AHGALRVRPAAEPVPSAIAGSPAGEVFGHLVRMNDGTAHEVQRPALQRALAGLDLNAVRE
AALQVARQVPQQQPLSDTLFSMPVRSVAHLLGFADEALPQVDLWTRDFVACLSPLSTASQ
LQGASTAAGELMARFEDLVAGTPPRRGTLLAAVMAESAAPSRSLLANLVGLLSQTCDATA
GLLGNSLIALLREPSLRTAARTRDGLQAIVEETARHDPSVHNTRRFVATPVTIAGTPLAA
GDTVLVLLASANRDPAFNPDPDSFVLVRAKRRMLGFGHGTHACPGQTLACTLAAAGLEAL
LQRAPDVDAQLLRGWHYRPSVAARVPVFH

>C4F17_22035|KEGG|CYP102A20|[Variovorax sp.]vam|BP

MAGKNSLHPIPHPAKKPFVGNLLSIGSDSPVLDMWKIAQDLGGIYWLDMPGMPVIVVSSP

ALVDELCEEARFDKSTRGALRRLRAASHGLFTSDTHEETWSKPHNILLANFSQRAMQAYH
PMMLDIAGQLVTKWERLNFDEEVDVVRDMTALTLDTIGLCGFGYRFNSFYREGFHPFVDA
MVRTLETVQNRRGLPLEELMLKKELAQQRKDIRYMHKMVEDIIEERRASGADIATKPDLL
SYMIAGVDKKSGEQLTDKMIRDECIEFLIAGHETTSGLLSFAIYFLLNNPEAMAKAQAEV
DNVFGPDTTQKPTYAQVNRLQYVMQVLKESLRLFPTAPAISMRAKEDTKIGGQYTIKKNN
MVIMHALALHRDKGIWGENADRFDPDNFSREAERERPVNAFKPFGNGQRACIGRQFALQE
AVLTLGMILQRFNLVDHTGYKLKIKEALTIKPEGFRIKALLRDPATRPRGNMTDAAAAPV
QPVQAAAARKPHAARHGTSLLVLQGSNLGTAEDLARQLAEAGEMRGFSTQLASLDDYAER
LPANGAVAIVCASYNGVAPDNAAEFHRWLDKADDALNGVRFSVFGCGNTDWAATYQAVPR
RIDERLEALGATRVHPRGEGDAREDMDGAFQDWSDALWPELVKAFDIKTGADTPAQSEPL
YTLEELPPPQKNAIVDALGAVALRVIENRELQKGGNGEGGRSTRHVELMLPEGLVYRAGD
HLSVVPRNSPAQVERAMARFGFDRTAHVRLHAVAGRKAALPVEQVIAVDRLLGDYVELQD
VATRKQIATLAAYTECPFTKPKLLALSGSDEASQAAYKADVLHKRKSLLDLLEEHRACQV
PFAVFLEMLSPLSPRYYSISSSPTMTPGRCSVTVGVVSGPAKSGIGTFEGVCSNFLARAE
AGDTVHGVVRETTAEGFRLPENASRPLIMVGPGTGLAPFRGFLQERAAQIEAGNALGEAL
LFFGCRNPEQDFIYADELQAWAHRGVMKLHTAFSRAGERKVYVQDLIREQSAHVWKLLEA
GAVVYVCGDGSRMEPDVRRTLSDLAREHGHDSAAWMDKMIADQRYVLDVWAGS

>C4F17_30875|KEGG|CYP108E2|[Variovorax sp.]vam|BP

MSISDIDGEIASHQLYADPLRMHAVFDRLRREDPLHWTQAPGHPPFWAVSKHADVVEVGR
HPDVFVAAPKTFLLDDEDLRVRIAETAATGGKLVRTMIHMDDPDHRKYRGLTQSFFMPAN
IKRLETIIQDRARALVGRMLERADGCEFCNEIAVWFPLQIIMTLLAVPEKVHAYLLKLTQ
QFLAPKDATLKRDGPEAQGKGAVAREYFTYFGKMLAERRAAPLADDLGSLIAHAQVDGKP
LETMEAVSYYVILATAGHDTTSSSMGSGLYNLLRQPGELERLRARPELMASAVEEMFRHG
SPVKHFVRTATRDYELRGKTIRAGDEVALMYHCANFDEEVFDDPRAFRVDRAPNRQIAFG
FSVHACLGQNLARSSMRAFFTELLARTAHIELMGEPAFIASNQVGGLKALQVRTTPLAAS
ATDAQPAGAAA

>CtCNB1_2750|KEGG|CYP1098B6|[Comamonas thiooxydans]ctt|BP

MSRFCPVYPKPHPEQTSKLRMFWHARRSWMDALFERSYSMQMGEVHLPGVDLYMLNDIKE
VKRVMQSEAQQFPKSHLLHESLEPLLGDSIFTTNGEQWQRQRTMMNPAFAQARVNVAFPR
MLGAAQSMLERLDAVEEGQPYDIEIEMTHVTADIIFRTIFSKPMQGPDAHRIFEAFARYQ
SLAPKLMLPALFGARWLTMPWYRWQSNQAAREIRGLLHAMIKPRYEAHLKGEAEEQEDIL
AAFMQAKDPNTGEPFNEEELVNQVAMLFLAGHETSASALTWASYLLAQSPDIQQRAYEQV
VEVVGDRLPQQADMKSFSQVWNIFRETLRLFPPVGFFARENVQSCPIRGKQLKERSTVVV
APWLLQRHRKWWKNPDAFDPDRFDREEDKEAIKHAYMPFSMGPRVCLGAAFALQEAVLIL
ACLLQRYEILPAAGHVPQPVGRLTIRSENGVRINLRKREAA

>O987_16625|KEGG|CYP1098B6|[Comamonas testosterone]ctes|BP

MSRFCPAYPKPHPEQTSKLRMFWHARRSWMDALFERSYSMQMGEVHLPGVDLYMLNDIAE
VKRVMQSEAQQFPKSHLLHESLEPLLGDSIFTTNGEQWQRQRTMMNPAFAQARVNVAFPR
MLGAAQAMLERLDAVDQAQPYDIEIEMTHVTADIIFRTIFSTPLQGPDAHRIFEAFARYQ
ALAPKLMLPALFGVRWLTMPWYRWQSNQAAREIRGLLHAMIKPRYEAHLRGEAEEQEDIL
AAFMQAKDPNTGEPFNEEELVNQVAMLFLAGHETSASALTWASYLLSQSSDVQQRAYEQV
LEVVGDRLPQQADMKSFSQVWNIFRETLRLFPPVGFFARENVQSCPIRGKQLKERSTVVV
APWLLQRHRKWWQNPDAFDPDRFDREEDKEAIKHAYMPFSMGPRVCLGAAFALQEAVLIL
ACLLQRYEILPAPGHVPQPVGRLTIRSENGVRIKLRKRKDA

>O987_22120|KEGG|CYP226A26|[Comamonas testosterone]ctes|BP

MTSTSTSTSASSLERAFAGVADNYRGSDVDLHAVYAAMRRDSPVVAENFMAGLGVPNIAG
QDANRPTFTVFKYRDVMAVIRDATRFTSGFIAEGLGAFFDGLILTGMDGEEHRRARALLQ
PVFMPDVVNRWKPKMDAIIRNEYLLPLAASKRADLMDFALHFPIRLIYSLIGFPENDPQR
INQVAAWALAILAGPQVDPEKAVRAREAAMEAARMLYDAVMEAVVQVRAEGATGEDLISR
LIRAEYEGRQLNDHEITTFVRSLLPAAGETTTRTFGSLLVLLLERPELLDRVRNDRALVG
KAIDETVRYEPVATFKVRQAAKDLEIDGVHIPKGAMVQAIVMSANRDEETFEHADRFDID
RKAKPSFGFGFGPHMCIGQFIAKTEMQVALNAVLDLLPNLRLDPAKPAPRISGAQLRGPH
EVHVVWD

>O987_26720|KEGG|CYP116B146|[Comamonas testosterone]ctes|BP

MTISTPSRCPIDHSQFGKPTGQSGPTGCPISHGAASFDPFADGYQQDPPEYVRWAREQEP
VFYSPQLGYWVVTRFDDIKAIFRDNLTFSPSIALEKITPTGDEANAVLASYGFALNRTLV
NEDEPAHMPRRRALMQPFTPEELKHHEPMVRRLAREYVDRFIDDGRADLVDQMLWEVPLT
VAMHFLGVPADDMDTLRQYSIAHTVNTWGRPRAEEQLAVAHAVGNFWQHAGRILDKMRAD
PDAEGWMQYGIRKQKELPEVVTDSYLHSMMMAGLVAAHETTANAAANAVKLLLQHPKVWQ
EICADPSLIPNAVEECLRHNGSVAAWRRLATKDVVIGGQAIPAGSKLLIVTSSANHDEQH
FVDADLFDIHRDNASDHLTFGYGSHQCMGKNLARMEMQIFLEEFTRRLPHMQLAEQRFTY
VPNTSFRGPEHLWVQWDVSRNPERGNPAVLAEQQPVRIGESSQGGVSRSMLVQSTELIAQ
DVLRVRLKAPDGRDLPRWTPGSHIDLHCGDTGISRQYSLCGDPQQADTYEIAVLREEHSR
GGSAWIHGALQAGMKVQMRGPRNHFKLDESAQQLILVAGGIGITPVAAMAARAQALGIPY
EIHYSCSQRERMALLTALQARHGERLQIYVGAEGQRADYQALLAQVRPDAQIYACGPQRM
LDGLAQASQHWPEDSLRVEHFSSTLGALDPDQEHGFEVELRESGITLQVRNDETLLDALR
SANIDIECDCREGLCGSCEVPVLEGDIDHRDMVLTRAERTACTKMMSCCSRARTPESRIV
LAL

>CCO03_00415|KEGG|CYP108S8|[Comamonas serinivorans]cser|BP

MTTAVATPDVDLPAHIASFLVDAQGYADVERLHDTYTWMRAHNPLGRVTHPDYAPFWAVT
KHADVTAISRNNTLFSNGASSVICRPQAFIDHLIAKNGIPHSVKALVHVDGEEHKALRGL
TQSWFMPNSILKLNERISALARQNVDALARHNGQTVDFVTDLALHYPLHVIMDILGVPPE
DEPRMLALTQEFFGNQDPDLKRGLDSGGSDNGLGLGQNMLAVVADFRAYFDRLTDERRRN
PRNDVASLLASGTINGEPLGDLQRLGYYIIIATAGHDTTSSSASCAMWALAQHPELLPRL
QADPALIPQFIDEAIRIATPVRHFMRTAMADTDIRGRTIRQGDWLMLCYGSANRDEEVFE
QPFAFNIDRKPNRHLAFGFGAHVCLGQHLAKLELRLLFEALIPRLQRVEQVGEMRMMSSL
FVGGPKTLPLRCVVQ

>CCO03_02720|KEGG|CYP153A109|[Comamonas serinivorans]cser|BP

MTINQAVQAAARERAYAMPLADVDPCNVDYFEDDTVGHYFERLRREDPVHMAVHPLHGPY
WSITRYQDIMSVDTNHQVFSSDWSLGGITITDEKMEEARLQMFIAMDPPKHDVQRKAVTG
IVAPSNLANMEALIRERTIKVLESLPRGEAFDWVDKVSIELTTLMLATLFDFPLEDRRLL
THWSNVATSSKLVDPDAPTPEERLAELHKMLAYFTRLWNERVNAEPGHDLVSMLAHNPAT
RNMGPAEYMGNLVLLIVGGNDTTRNSMTGGLLAMSQNPGEWQKFRDKPELIPNMVSEIIR

WQTPLAHMRRTATQDTELNGKTIKKGDKVVMWYLSGNRDDSAIANPEQFLIDRPQARQHL
SFGFGIHRCVGNRLAEMQLRILWEEIHKRFPVIEVLAPPKRVRSTFVRGYSEMQVRIPV

>CCO03_08185|KEGG|CYP108S9|[Comamonas serinivorans]cser|BP

MNTALAQAPVDLPEHIAAAIVDHKHYANVDGLCETYAWMRANNPLGRLNHPAFHPFWVVT
KHADVMAISRDNALFTNGAYSPTCAPQAYLDHVLATQGTRHPIKSLIHVDGAEHKELRGL
TQAWFMPNSILKLDERIKQLAQTSVDKLSSLSAQGQVVDFVNDVALYYPLHVIMDILGVP
PEDELRMLKLTQELFGAEDPDLKRQSAAVSPEERAKVMMEVVGDFRNYFNALTEARRQDP
RNDIATLLANGTVAGQPLDPLQLLGYYMIVATAGHDTTSSTATMAMHALATQPGLLQRLK
DDPKLIPAFIDEAIRVATPVRHFMRTAMADTEIRGRTIREGDWLMLCYLSANRDEDVFAN
PNVFDIDRKPNRHLAFGFGGHVCLGQHLAKMELRILFEALIPRLQSVSLAGEMRMTATLF
VGGPKTLPLHCVVS

>FOZ74_03160|KEGG|CYP289F1|[Comamonas sp.]cof|BP

MTDRPSSDWDVRSDEVQRNPLAAHDRLRESHGVARSEVLHTSVFRHADVLAVVQDHARFS
SVVSAHVAVPNGMDPPEHTSYRRLIEPYFAPDRVAAMRPLYQRLAHELLAHTRQVHARQP
FDFIAGCALPYAARTQCAFLGWPLALEGVLCDWIARHHAAVFAQDRARLAQLGLAFEALI
GRMLDERDASASGPPRDATEALLRERVEGQPLARAAIASILRNWTVGEIGTIAASLGILA
DWLARHGELQQQLRAQPALQAEAIEEILRLHGPLAVNRRVARCPVQLGGHEFGAGERVTV
HWGAANRDPRVFEAPEEFRFGRDHARSLLYGAGIHVCPGAPLARAQLGLWLQALLGATTH
FRLAQPAQPPQYARYPAMGFERLPLVLDWA

>LAD35_12920|KEGG|CYP1982B1|[Comamonas odontotermitis]codo|BP

MSQPFSDDFWARMYELAREEPGRNAEVIHDGLRVVVVQDLDDADIVLRKRLANYPKNLQW
FAQVGGQSRLTEDGDQWRFRSELSQPSFSRYDHDRGYAISAKQTRRMAGLLAANTKVPLL
DEDVIHHCILSIFTEMFLDVELDKIPIAHDGPSRLIELASKYTCIHRGQGQDAHTKDHIR
QILESRRQVFGSMQCLRDGNAYDSAMMRRMLAAEAVPGYDFRFEKEMTTLFVAGADTASN
STGWALHLLASYPELQERLHTGQRAIHTSLPDGTERCRAILADQDLKAFISELLRLHSPL

PFVTRVAKADDRLSNIDVHARDVVIVSLIGVNRKAMARPDPWVPDIDAAAREGVGMGTGI
NSSFTWGARVCGGRSFALLELTTILSFLIAELHFGLSENLPIECEWVGQMRRKGGHRITS
SVRK

>Alide_3136|KEGG|CYP289A19|[Alicycliphilus denitrificans]adn|BP

MTTTPPQWDPQAPEVQRDQRAAYDQLRERCPLAHSESQHWSVLRHAEVMRVLDDHATFSS
AVSAHPAVPNGYDQPEHTAYRRALDPFFAPERMARFEPVCRALSGATVQEALALGGDLEI
TQEFALPFAVRVQSAFLGWPTLLQDELLGWLRRSHAATRSGDRVQTSAVAREFEDLVERV
RQARRDAPPTQDVSSELMHTRVDGRLLDLRELASVLRNWTVGEVGTIAASIGILMHWLAS
HPDWQQRLREQPALLPTAIDEVLRIDGPLVSNRRITTRATALGGCPLPAGSRLSVNWIAA
NRDPRVFGDPDAFRMDRDPGLNLLYGAGIHVCPGAPLARLELRVLIEELLARTTRIVLAA
GHAPARAVYPDAGYATLRMRLG

>Alide2_1311|KEGG|CYP289A19|[Alicycliphilus denitrificans]adk|BP

MTTTPPQWDPQAPEVQRDQRAAYDQLRERCPLAHSESQHWSVLRHAEVMRVLDDHATFSS
AVSAHPAVPNGYDQPEHTAYRRALDPFFAPERMARFEPVCRALSGATVQEALALGGDLEI
TQEFALPFAVRVQSAFLGWPTLLQDELLGWLRRSHAATRSGDRVQTSAVAREFEDLVERV
RQARRDAPPTQDVSSELMHTRVDGRLLDLRELASVLRNWTVGEVGTIAASIGILMHWLAS
HPDWQQRLREQPALLPTAIDEVLRIDGPLVSNRRITTRATALGGCPLPAGSRLSVNWIAA
NRDPRVFGDPDAFRMDRDPGLNLLYGAGIHVCPGAPLARLELRVLIEELLARTTRIVLAA
GHAPARAVYPDAGYATLRMRLG

>Rta_14540|KEGG|CYP195A32|[Ramlibacter tataouinensis]rta|BP

MQECRMSAAEPNPPALPPVTPAMSPARAAEIAAGFDLRALPADFYANPYPVYARLRETEP
VRRMPDGSYFLTRYADLVAVYRDAATFSSDKKVEFTPKYGEGSPLLEHHTTSLVFNDPPL
HTRVRKLIMGALTRRAIAEMEPGLVALVDGLLDRIAQRGGGDLIEDFASAIPVEIIGNLL
GVPHADRGPLRDWSLAILGALEPRLTPEQEALGHRSVQDFTAYLRTLVAQRRRHPGDPEH
DVLTRLIQGEAAGEQLSEAELLQNCVFILNAGHETTTNLIGNALVALQEWPGEKARLLHR
LKHSAHHPSGLEADLTLAVDEFLRFESSNQLGNRRALRACEVGGVALPEGALVTLCIGAA
NRDPAQFGQPDVLRLDRESNRHLAFGFGIHQCAGLSLARLEGRIAIGRFLQRFPRYRLPE

APTRGGRARFRGFLKAPFSVA

>Rta_37620|KEGG|CYP152AR2|[Ramlibacter tataouinensis]rta|BP

MNRFFTAPKPARPIPVLAGPDHTLAFAADPYRFIGRECAARHTDVAQGRLLLAPTLFLTG
APAAQLFYDKERFVRAGAAPEPLRATLFGKGAVQTLDGSAHLRRKAFFLEATAPARVAAL
VRQAEVTWLTMASHWRSQPQLVLYTSAQEWLTRSVCAWAGVPLPEHEVQLRTGQLAALFD
SAASGLGAHLRARRARRQAETWLAGLVQEIRDGRRRVAGRTLLQAAAELTDERGEPLPAR
IAAVELLNVLRPTVAVSVFVVFAAHALHHHPGWAPTLAAGGDLGADAFVQEVRRFYPFFP
AIAARVRRDFVWEGYHFPQGRRVLLDLYGTNHDGRLWPHPGEFRPERFMGRIPGLFELVP
QGGGRADDQHRCPGEGVTLALMKLALRLLVQRSRYRLPPQNLAIDMRRLPALPRDRFVIA
DFQPV

>L103DPR2_02277|KEGG|CYP195A34|[Limnohabitans sp.]lim|BP
MSITWQTPALSAQRVQDICSQFDLRNLPADFLANPYPVYAALRETTPIKQMPDGSFFLTR
HADLVTVYKDAAKFSSDKRIEFAPKYNHEPFNQAPFAKPGQDAPLFEHHTNSLVFNDAPR
HTRVRKLIMGALTRRSIDAMETGLIQLVDSLLDQLEARQHGDLIEDFASAIPVEVIGNLL
NVPHADRGPLRGWSLAILGALEPRLTAEQETLGHRSVQAFSNYLRDLVADRRRHPGNPEQ
DVLTRLIQGESSEHSADVLSETELLQNCIFLLNAGHETTTNLIGNALLSLLEHPAQKASL
MADLQTTRSGSHEDAATPILNTAVDEFLRFEASNQLSNRRATVATRIGNVDLPEGALVTL
CIGAANRDPAQFPNPEVLDLKRADNRHLSFGFGVHQCAGLSLARLEGRIALGRFLQRFPN
YHLTATPTRGGRARFRGFLHAPFATGL

>L63ED372_01421|KEGG|CYP195A31|[Limnohabitans sp.]lih|BP

MSADRAQKIAQTLDLRALPRDFLDNPYPVYAALRESEPFKRMPDGSYFLTRHADLVAVYR
DAKVFSSDKQVEFGPKYNHAPFNQPPFAATGGVAPLFEHHTHSLVFNDAPRHTRVRRLIM
GALTRRAIEAMEPGLVQLVDSLLDKIEAQGGGDLIEDFASAIPVEIIGNLLDVPHADREP
LRGWSLAILGALEPSLTPEQEALGNRSVSEFLAYLRELVAQRRQHPGDPEHDVLTRLIQG
EENGDALSEVELLQNCVFLLNAGHETTTNLIGNALISLQEWPAQREQLQADLKQAATEAE

GEQIMGLAVDEFLRFESSNQLGNRRALQATQVNGMDLPAGALVTLCIGAANRDPAVYENP
VQLNLRRTGNKHLAFGFGVHQCAGLSLARLEGRIAIGRFLQRFPDYQLTQSPVRGGRARF
RGFLHAPFSIE

>L63ED372_01617|KEGG|CYP225A10|[Limnohabitans sp.]lih|BP

MTVWHPTDDGHADLADHDSYVNGTPHNTFQRLRDEDPMAWCDWSEGKGFWSVTRHADILE
LNRNHQRLSSAQGIRMEDQTHEEYMARRTFQETDPPEHTRTRMLVAKAFSKPMMALYEDQ
IRELCVGILDQALGQGEFDATHQIARQLPMRMLGRIAGLPEEDLDWLVDKGDALMANTDS
DFTSHVVDKMDTEAYRFMPFRSPAGADLFDYAARMMARKRAAGDTSGVLHLITQPDAQGN
VISDTEFRNFFCLLVAAGNDTTRYSIAAALHALANQPGLMAQLKAVQGEAAWEGVADEFI
RWASPATHFRRTATEDFEYHGKQVKAGDKVLLWFISGNRDERAFEQPFTVDLNRGVNRHL
SFGQGGAHACLGMWLARLELRVLLQELIQRVDRIEQTGEHAFLRSNFVCGIKRLPVRMHL
I

>BSY239_1146|KEGG|CYP195A27|[Hydrogenophaga sp.]hyr|BP

MSFVLRPEHITPPMAPDRARDIAARFDLRALPPDFLANPYPVYAALREHEPVRRMPDGSV
FLTRCADLMAVYRDAQAFSSDKHVEFAPKYGAGSALFEHHTTSLVFNDPPLHTRVRRLIM
GALTRRAIADMEPGLIALVDSLLDDLAERGGGDLIEDFASAIPVEIIGNLLDVPHADRGP
LRDWSLAILGALEPTLTPQQQALGNQAVTDMLAYLRTLVVQRRQHPGDPERDVLTRLIQG
EDGGEQLSEVELLQNCIFLLNAGHETTTNLIGNGLIALQEHPQARQFLHTALKQTEDDAA
AQEAVLTRAVDEFLRFESSNQLGNRRAVKDTQVGGVALPAGTLVTLCIGAANRDPAQFPD
PEVLKLARENNKHLAFGFGIHQCAGLSLARLEGRVAIGRFLQRFPDFKLTEAPVRGGRAR
FRGFLRAPFSVS

>BSY239_2681|KEGG|CYP199V1|[Hydrogenophaga sp.]hyr|BP

MTHPNPYDNVPTSDFDPYATEVLANPYPHYEQLRELGPWAWLERYGVLAVARHDEVQRIL

SQPDIFCSSAGVGLANFHTETPWRKPSIILEADPPHHSRTRKVVARVLSPASIGRLRATF
EDVAARMVDELVERGSFDAAKELCEAFPIKAFGDAVGLPAEGREHLLPYGDMVFNGFGPI
NERFQDRMNSSGEAVNWIAKVCQRDALTPDGLGAQLFAAADAGEIGHDEAGMLVRTLLSA
GLDTTVFTLCNAFTSFARNPGQWALLRDDPSLARQALEEVLRYDSTFHSFYRTSTQDVEL
GGITVKQGQKIVVFIASANRDPRRWPEADRFDITRRATGHMAFGTGIHGCAGQMMARLEG
EIVLTALARRVASIRLDGEPVQHFNNTVRGLKSMPVTVEPLLAAVH

>LPB072_02045|KEGG|CYP212B6|[Hydrogenophaga sp.]hyl|BP

MKTDYLGFCTRLRRDHGDISHMKLLSENVWDLMSPDLVREALVTHADHLLRWERGIEVFS
EVFGQSVLVTEGATWQRQRRMLMTAFTPQRVNGYAQLMTTAAKEAFDAVSPKGQERALVN
MDTLWSGAAMDVILRTLFSQTAEPESLAAAKATQVLSTTGYREMFHLFTLPDWLPLPGKA
AKRQALHTLRSLIRGHIDARRSSHVEGGDHADLLGRLLALRDEETGEILSPQEVFDQCMV
SFQAGHETSATALLWWSRLMAEHPEAAKRAREEVASVVGAGTPGPEHLPQLPWLTATLKE
AMRLYPPVAAIMARRTTAPIELGGWHTPKGAMLRIAPYVLHRDERNFANAEAFLPERFLE
GASPIPRGAFMPFGAGPRVCLGQHFAMLEMTLLSAMLLQRFELHLPDNAPRAEPEMHVTL
RPKQSIQLWLHRVPNARAGQGAAP

>E5678_02035|KEGG|CYP195A29|[Hydrogenophaga sp.]hyc|BP

MTTPLAPQTITPAMTRERAQAVASGFDLRALPPDFLANPYPVYTELRETEPIKRMPDGSV
FLTRHADLMAVYRDAQHFSSDKHVEFAPKYGAGSPLFEHHTTSLVFNDPPLHTRVRKLIM
GALTRRAIADMEPGLIQLVDKLLDRIEAQGGGDLIEDFASAIPVDIIGNLLGIHEGERGP
LRQWSLSILGALEPVITPAQQVEGDRSVTAMLTYLKGLVAERRLRPGDPERDVLTRLVQG
EANGEQLSEVELLQNCIFLLNAGHETTTNLIGNGLILLQEFPETKDKLLRNMAAVDHDAA
AHEAVLSAAVDEFLRYESSNQLGNRRVVHATQLSGVDLPVGSLVTLCIGAANRDPAAFAL
PETLDLQRSPNKHLAFGFGIHQCAGLSLARLEGRIAIGRFLQRFPGYHLTAPPVRGGRAR
FRGFLHAPFATV

>HPF_14080|KEGG|CYP2730A1|[Hydrogenophaga pseudoflava]hpse|BP

MAGLPDWAVEILKHHPQKVSHGLNLVLKLKHPELMPLVLWDIVVARPLIQRALDGLSFIH
FARFVPSWDGKALMVITEFDGPLEPYVMDFAIAIGDVFDRLLSYTEGAPPLPVREHPDEF
WTFVQEWNRVPFFPWDGERPDPKDPNSPFFPSEFDYPLYSAYPKLSAIDIAGPHEKRLPP
ALDNPAADVDARDVQGNILRPYAAATARYLFLRVTDAARARLWLAQQAAPAAADRTDAWP
PVTDASPWPRGADERVKKPAQMLNVAFTFAGMQALLPKRLEDLRLFPQAFRQGALERAKA
NGDISASKPDHWLIGKPGQVLHVVLTLHTFASESIGDADWQLFTAAFDALKANAVAHGFA
VEATHEAQAFLGQDRQPTGEIYFGYHDGIAQPRIAGQCPAHGGLKSDFQPAASPGEFLLS
EAYSDIFRGPSLGNLPPELARNGTYAALRLLEQDVEGFDGMLASESASAGVPAAELQARL
MGRWANGDPLTLSQTPPPLGTTSSRNDFDYAPSWEQPDLEQDHAGHLCPVGAHIRRTNPR
SARVAGQRHSRRLIRRGMPTQWEESGTTRRGLLGLFICGSLERQFEFIQREWIQGDLAAS
GIAGMQDPIAGLRDKPTDFHFFAGGQEGQARLVKVPPLVTTRGSLYLFIPGMGTLRHLEQ
VNDGKWRPPAHTSIATSAPASSVQVDLQTVSLAADALEKQRAAAVQIAKDVTENQFGVTQ
VQANSPTQVPGSVPLKVKGRAPVAALSSLATLEEMARRPIETLRQTGPILSVTVEASFSG
DAPLVDLPDAIRKMLAELVLEGVRSEWFKRLIEAFAPPPPDNPASGQPALGLPITELDPA
DAKFIIDPFGYWKQLRDAGMPVVWSKAHRAYWVLDYNLGQLVLSEPIDFVQQPSGAKLRG
IITLDPPRHTAVRKAVEEALMVAAAKNRVDGLVDDAVRAALESIGNLQQFDAVPLFTNAV
PAQVYWNVFGLPEENRIECAALARTVMLHFGQPEHRGVGDQLVSADASVRLVTRLALLLA
EALLAGLLEYLTGRNRFKGTLIGELADRVELPYMPSVKRVIGFEEALLTLLQLVLAGFMS
MQFLMGTALRNLLSPDPRANRGGEQPWAALGALLKGKPPDFPAALALALEEARRFDPPVT
IVQRFVGPNGAKLHGVQVAKDSPVFVVVGSANRDAAVAFDEPDQFHWDRPAGAGHLSLGH
GIHLCVGQTLQQLIVPSALTALLKAMPSLQLVDSAATPAWLDNVYFRALQSLPVHRC

>HPF_17070|KEGG|CYP195A33|[Hydrogenophaga pseudoflava]hpse|BP

MIQPQQITPPMSAERAREIAATFDLRALPADFLANPFPVYALLREHEPVRRMPDGSLFLT
RHADLMAVYRDAAVFSSDKHVEFAPKYGVGSPLYEHHTTSLVFNDPPLHTRVRKLIMGAL
TRRAIADMEPGLVTLVDQLLDRIEAQGGGDLVEDFASAIPVEIIGNLLGVPHADRGPLRD
WSLAILGALEPVPTPEQHAAGNRSVTEMLAYLRTLVAARRQAPGDPERDVLTRLIQGEAS
GEKLSEVELLQNCIFLLNAGHETTTNLIGNGLILMQEFPAQRKQLLADLRTAGTDEAARE
QVLTLAVDECLRFESSNQLGNRRALTAATVGGVALDAGALVTLCIGAANRDPAVYDQPET

LNLRREGNKHLAFGFGIHQCAGLSLARLEGRIAIGRFLARFPRFELSAPPVRGGRARFRG
FLSAPFRTA

>F9K07_04220|KEGG|CYP105T5|[Hydrogenophaga sp.]hyn|BP

MSDTRVPASTPRCPFHHDSHAEGDVAALDAFPFPRTDKLGPPTQYAAYREARPVARVCLW
DGSAAWLVTRYEDVKAVLGDRSFSVDPAKGLKSTSPARAAQIASRKTFINMDDPEHLRLR
RMLAPEFMQPSLQRLRPQIEQCVQARMDAMERKGTRADLVADFAVPIPSEVISILLGIPS
EDIEQLNHLSHTRNDHSAGVQVVEAAAQAMEDYLDQLIQKKKRDTRHSDPGEGSDLLSRL
VNQQVLPGHLTHDGAVKMAALLYSAGHGTTSNQIALGALGFLLWPDQIELLKADPSRLAG
AVEEMLRFHTVAHMNSARLATQDVTVGGQLIRAGETVYAMDASANRDPTVFEEPDKFDIC
RAGNKHLAFAYGIHQCLGQPLARLELTVVYERLFQRFPRMRLAQAPETLEFSESSQVYGV
KCLPVEWS

>F9K07_04270|KEGG|CYP105T6|[Hydrogenophaga sp.]hyn|BP

MPLPRDDGPLNPPGGYARLRSECPVSRATLWDGSEAWVVTRWQDVRAVLSSPSFSSDPNT
PGFQSLSQARQQQVLARNTFINMDDPEHARFRRMLTRDFMQKRMGEMKPQVDSLLEGLYA
GLEQSPQPSDFMEKVANPLPVMVVSLLIGVPFEDFEMLGRWSNLRIDHRAAPQEIRQATL
DMEAYFENAIRERLKKPGDASDLLTRLVQEQLQPGHIELEELVRLSALLYSAGHGTTSTQ
IGMGTLTLLRHPEQKDAMAADPALVPRAIEEMLRYNAIAHLNSSRVAIADVEIGGQSIKA
GESVHAMLVAANRDPATFENPDRFDIHRQSQPHVAFSYGVHQCLGQPLARLELNAVFSTL
FQRFPRLQLAVPFEELRFNAMSQVYGVEALPVTW

>F9K07_19630|KEGG|CYP195A30|[Hydrogenophaga sp.]hyn|BP

MSLLRDAITPPMSAARAQAIAQSFDLRALPADFLANPYPVYAALREAQPVRCMPDGSVFL
TRSSDLVAVYRDAQSFSSDKHVEFAPKYGEGSPLFEHHTTSLVFNDPPLHTRVRGLIMGA
LTRRAIAAMEPGLTMLVDGLLDEMDARGGGDLIEDFASAIPVEIIGNLLNVPHADRGPLR
AWSLAILGALEPTLTAQQQADGNQAVSEMTAYLRTLVAERRRRPGDPEHDVLTRLIQGEA
GGEQLREVELLQNCIFLLNAGHETTTNLIGNGLILLQEFPEARAGLLHALRETAGDAAAQ

EAVLGRAVDEFLRFESSNQLGNRRAVKDTQVGGVDLPAGTLVTLCIGAANRDPAQFAEPD
VLNLAREGNKHLAFGFGIHQCAGLSLARLEGRIAIGRFLQRFPHFRLSDAPTRGGRARFR
GFLRAPFLLV

>H9K76_13975|KEGG|CYP1982B1|[Diaphorobacter ruginosibacter]drg|BP

MSQQFSNDFWNRMYELAREAPGRNAEVMHDGGRVVVVQDLDDADAVLRKRLANYPKNLQW
FAQVGGQSRLTEDGDQWRFRAGLSQPSFSRYDHDRGYAISARQARRMAGLLAANTKAPLL
DEDAIHHSILSIFTEMFLDVELDRIPMAHDGPSRLIELASRYTCIHRGQGQDAHTKEHIR
QILESRRQVFGGMQCLRDAGEYDSAMMRRMLAAEAVPGYDFRFEKEMTTLFVAGADTASN
STGWALHLLATYPELQERLHAGQRAIHASLPDGPERCKAILADQDLKAFVSELLRLHSPL
PFVTRVARADDRLSDIDVHAGDVVIVSLIGVNRKAMARPDFWIPDIDAAAREGVGMGTGI
HSSFTWGARVCGGRNFALLELTTVLSFLIAELRLELSEDLPVENEWVGQMRRKGGHRVRV
TPRVLFSPIFDQ

>H9K76_15125|KEGG|CYP1982B1|[Diaphorobacter ruginosibacter]drg|BP

MSQQFSNDFWNRMYELAHEAPGRNAEVMHDGGRVVVVQDLDDADAVLRKRLANYPKNLQW
FAQVGGQSRLTEDGDQWRFRSGLSQPSFSRYDHDRGYAISARQARRMAGLLVANTKAPLL
DEDAIHHSILSIFTEMFLDVELDRIPMAHDGPSRLIELASRYTCIHRGQGQDAHTKEHIR
QILESRRQVFGGMQCLRDAGDYDSAMMRRMLAAEAVPGYDFRFEKEMTTLFVAGADTASN
STGWALHLLATYPELQERLHAGQRAIHASLPDGPERCKAILADQDLKAFVSELLRLHSPL
PFVTRVARADDRLSDIDVHAGDVVIVSLIGVNRKAMARPDFWIPDIDAAAREGVGMGTGI
HSSFTWGARVCGGRNFALLELTTVLSFLIAELRLELSEDLPVENEWVGQMRRKGGHRVRV
TARS

>C6571_02485|KEGG|CYP289E1|[Simplicispira suum]simp|BP
MPSHPKPDWDPLSPQAQHDPQATADGMREHCPVAWSQALHWSLFRHADVLRAVKDHTTFS
SVVSRHVAVPNGMDPPQHTVYRRVIEPYFSKARVDAFEPQCRAMVRSLVATLPRSAPIEW
IDALALPFAVWAQCDFLGWQDEDHAALAQWMQANYAATLAQDRAAHTELAARFQAVVTAQ
LERRRGIGAAAAPGDLTWALMQEKVHGVPLTDAEIASILRNWTVGEIGTMAASAGILCDW
LAHHAPVQAQLREHPSAIPAAVEEILRLRGPLATNRRITTCPVDIGGRHLPAGSRVTLHW

PAANRDARAFDAALEYRPERDQTANLLWGAGVHVCPGAPLAQMELRVLLEEVLVSGASME
TVPGESPEPAMHPASGWARVPLRLVPLQA

>SRAA_1070|KEGG|CYP203A12|[Serpentinomonas raichei]cbaa|BP

MTPFQFDPYAPAVDADPFPYHHRLREEFPCFWSEQAQMWVLSRYADIVSALNDWQTYSSA
SGNLMTELPGRAGATLGSSDPPRHDRLRALVQHAFMKRNLLALEEPIRAVAQQVFGQLQG
VDQFDFKDVSSQFTVKVLMTALGLPMGDEALVAEHEVRDHAVLMVQSDARTRAKGPEHIA
AYQWMQNYAAQVIALRRAEPRNDLISHFAMAEIDGDRLDEREVLLTTTTLIMAGVESLGG
FMMMLAYNLASFDAARRAVVADPALLPDAIEESLRYNTSAQRFRRRLMCDVTLHGQTMRQ
GDFVCLAYGSGNRDPRQFPQPDRYDLARKPRGHLGFGGGVHACLGTAIARLALKIAFEEF
HRVVPEYHRVADQLPWMPSSTFRSPLVLELAVG

>SMCB_0828|KEGG|CYP203A12|[Serpentinomonas mccroryi]cbab|BP

MFSFQFDPYAPAIDADPFPYYRRLRDEHPCFWSEQAQMWVLSRYVDIVSALNDWQTYSSA
SGNLMTELPGRAGATLGSSDPPRHDRLRALVQHAFMKRNLQALEEPIRAVAQQVFGQLRG
VSQFDFKDVSSQFTVKVLMSALGLPMGEEALVAEHEVRDHAVLMVQSDARTRAKGPEHIA
AYQWMQNYAAQVIALRRAEPRNDLISHFAMAEIDGDRLDEREVLLTTTTLIMAGVESLGG
FMMMLAYNLASFDAARRSVVADPSLLPDAIEESLRYNTSAQRFRRRLMKDVTLHGQTMRT
GDFVCLAYGSGNRDERQFPDPDRYDIGRKPRGHLGFGGGVHACLGTAIARLALKIAFEEF
HRVVPEYHRVAEQLPWMPSSTFRSPLVLELAAG

>IS481_09000|KEGG|CYP152B10|[Schlegelella thermodepolymerans]sthm|BP

MPRDPRLDSSLALLADPYRYIGKQCRAFGTDLFEARLLLQPAICMTGPAAAALFYDPQRF
VRAGAAPGPLRATLFGNGGVQGLDGADHHHRKALFMALMQPATVAELGQRYQEQLERSAA
GWIGQRRVRLYEALHLPLARAVCAWADVPWPEAEEAGRCADLVALFDQAAAPGRGHFFHA
RAARKRCEAWAGRLVEAVRQGRQPVRAGSALAAVAMHRDAGGQRLPVPVAAVELLNLLRP
TVAVSVYVVFVAHALHRHPECALALQQRSNGDYAECFVHEVRRCYPFFPAVVARVREDFE
WNGYAFPAGRRVLLDLYGTNLDPRTWHDPLSFQPERFRERTPGRFDFVPQGGGDPHLGHR
CPGEAIALELMQRSARFFATRLRYAVPEQDLTLDMTRAPALPRDRMVIAQAMPALP

>IS481_11920|KEGG|CYP116B144|[Schlegelella thermodepolymerans]sthm|BP

MELKSHPAGRCPVAHEAVTGAPTGCPVSARAAAFDPFQDAYMEDPAEFVRWAREQEPVFY
APRLGYWVVTRYQTIKDIFRDPVTFSPSIVLERMSPTSDEALQVLKQYGYAMNRTLVNED
EPIHMARRRVLMAPFTPEHLAGHVPMVRELVRKAVDRFIDDGRADLVDQLLWEVPFTVAL
HFLGIDAEDREKMRRFSIAHTVNAFGRPSPEQQVSIAHTVGQFWQFAGEVLEKMRRTPDG
PGWMRYSIRQQKLYPDIVTDSYLHSMMMAIIVAAHETTAFASANAIKLLLQHPTAWRDLC
EDPALISPAVEECLRYSGSVASWRRRTTRDVVLDGVPVPAESKLLLVVSSANHDGAHFVD
PDLFDIRRENSAEHLTFGFGAHQCLGKNIGRMEMQVMIEELSRRLPHMKLAPQTFEYVHN
VSFRGPQHLWVEWDPARNPERRDPSILQRRHPVRIGAPHARDKVRPLVVRAAEPVAEGIV
RLRLAATDGRPLPQWAPGAHIDIECGETGLSRQYSLCGPLSDTSEWTVAVQLDPASRGGS
AWIHRHATPGTVLRARGPRNHFRLDEQARRLIFVAGGIGITPIMAMAERARALGIPYEIH
YSVRCRACLAFERELRELHGERLHLYVSEEGRRNDLAGLLATPDPQARIYACGPQRMLEA
LERLAAGWPEDALRIEHFSGSAPRLDPSCERPFEVELRNSGLRLEVPADRTLLEVLRASN
VDVQSDCEEGLCGSCEVGVLEGEVDHRDSVLGRAERQGHRRMMACCSRARSQRLVLDL

>IS481_12540|KEGG|CYP229P1|[Schlegelella thermodepolymerans]sthm|BP

MDEEILDPVAAVTAPDPYAAYAAWRARGPLVWHERLQLWLATDAAAVEAVLSHPALHVRP
AGEPVPRGLAGSAAGAVFGQLARMNDGPAHQDARQALQARLAHIELPRWSALAQRHARRL
PVPGAGALDEWVVRVPLLAVAEVLGIAQSLQDEVVTQAHRFAAGISPLASMSQREAAAEA
AQRLAGWLDPVAGPTPEGLSRACWVANLLGLLSQTCDATAGLIGNTLVALAREPGLWPRL
RDDAGALREAIEEVARHDAPVQNTRRYAHAEGALVLGQQIAPGQAVLVLLAAAGRDPARH
PEPDRFMLRRAPRVPYGFGHGPHRCLGERIACALAQGALSVVPPGAVEVARAFLQRPRYR
PSVNARIPWFAPEEAA

>G8A07_01530|KEGG|CYP1097A20|[Kinneretia sp.]kia|BP
MELSSPSAAAGRSYRDLPGPRGIPLLGNALQIESARFHQQLEHWAAIHGPLYRLQLGDRR
AVVVGEHQAVAAILRDRPEGYRRTTKLEAVWREMGLPVGVFGAHGEAWARQRRMVMAGFD
PGHVREYFPAMQRVAARLAARWRLAARVERSIDLQADLMRYTVDTIAGLAFGTEVNTLAP
EAGEEVIQRHLDKIFPALLARVLAPLPLWRWWPSRADRELAGSIEAVNTAVASFIARSRA

RLMAEPSRRARPTHLLEAMLVAADQPDSGVDDDQVAGNVLTMLLAGEDTTANTLAWLIYL
LWRHPVALARVQAELLDGAPQAEQTGQEQLAGLAYLDACIQETMRLKPVAPILALQALQE
RRIGDLHIEAGTVVLCLLRHDSVDEAQVPRPQAFEPERWLDGTSAAALRHLAMPFGAGPR
ICPGRYLAILEIKLAMAMLLGRFEILAVDTPEGGEAAERLVFTMAPTSLGMRLRER

>G8A07_02905|KEGG|CYP212E1|[Kinneretia sp.]kia|BP

MQQAHHSLSPPQPALPPGPRQRWLGLPELGAMRRDYLGFMSALRRRHGDIVHMRLVGYRD
YSFFHPEQVRELLLAAHERLIRWERGTEVFASAHGRSVLVAEGAAWQRQRRLLQPAFAPR
QVEAFAPRMAEAAEAALARWPARDAQFPFEAAMTQLTMEVILRSLFSRADEAPSRAAAQA
VHRLGQIGMSEMYWPVSAPLWAPWKAGKRRALATLDALIHGELARRRREPPPADAPADLL
QMLLALRDGEADERPALSERALRDECMTIFLAGHETSASALTWWGWCMTAHPQAQARAAA
EVLAQLGPTRAPTMADLPRLPWLTACIKETLRLYPPAPALLTRRSIAPLTIGGHELPPGA
TIRLTPGVIQRDPRWFEEPEAFRPERFDPASGHAEIPRGAWLPFGAGPRVCLGSHFALTE
ITLIAALILQRHELHPQPGAPAPRPVLNITLRPEQALRLALRRRD

>C1M51_06735|KEGG|CYP1097C1|[Methylibium sp.]metp|BP

MREPNEADDAAVARARAADAAPLRSVAGLPGPRGWPLIGNLLQFDLPRLHRQFEDWADRH
GPTYRLRLGRREVLVISDAETIAALLRDRPDTWRRMRPIEAVIREAGGHGVFSAEGDDWR
RQRRLVMSAFDPGHLKRFYPSLQRVTERLRVSWCEAARRGEAIDLQASLMRYTVDVTTGL
AFGVDLNTIEAPHPLQEHLDKLFPMLFRRVNLPFPYWHYLRLPIDREYDRHLRRVHEAVR
GFVQAARQRLQDEPARRERPTDLLEAMLAARDADGSALTEEEVAGNVLTVLLAGEDTTAN
TLGWAIWLLHEHPDDWRALVAEVDAALGDARLPPSFDAARGLASIEASVGEAMRLKPVAP
LLFMEAIRETALAGFRLPPGAVVFCLMRRAALDARLAEDVADFRPGRWQDGAERAVQKAS
MPFGAGPRLCPGRYLALLEMKMVLGMLARNFELLEVGPEGGGVPGERMAFTMFPVGLKMR
LAERVRG

>GJA_4741|KEGG|CYP1097A11|[Janthinobacterium agaricidamnosum]jag|BP

MHTDTPDTTADQPLRRIKDLPGPRPLPLIGNGHQIKPQRIHQHVERWSLQYGPLMRMYFG
ATPILVVADHEMVGAVLRDRPDGFRRPSISATISNEMGGIPGLFLAEGADWRNQRRMVMA
GFAPTAIKAYFPALVAVALRLRRRWQAAASARKAIDLESDLKRYTVDIIAGLAFGSDVNT
LESGEDVIQRHLDDILPAVARRSLALVPYWRYVKLPADRRLDRSVAVLRTAVQDLIGQAR
QRMLDNPARRERPPNLLEAMIAAADQSGSGVTDLNVAGNVTNMLLAGEDTTANTISWMIY
LLQRHPHTLQKARDEVRRNAPDAARFTIEQLDSLDYLGACANEAMRLKPVAPYLPLEALR
DTVIGDVAVPAGTMIWCVLRHDSVAEKHFPDPLLFDPQRWLQADGKPNSDKRVTMPFGAG
LRTCPGRYLALLEIKIAMAMLLGSFDIAGVDTPDGKEAQELMGFVMSPVGLSLRLE

>YQ44_18725|KEGG|CYP1097A5|[Janthinobacterium sp.]jaz|BP

MPISREIADLPAPPTLPFLGNAHQLNPRNIHQTMEKWSGSYGPIFRARIGSRQVVVLSDS
AAISQVLRARPDTFRRPAVTADISAELGGAPGLLLAEGDEWLTQRRMVMHSFSPSMVKAY
FVRLRQVGLRLAQRWSTAAQAQQEMDLSGDLKRYTVDVIAGLAFGEDVNTLLNGDDKLQE
DVSEVMAGVARRSLMPFPYWRWLRLPADRRLERSVRALQAATDGFIAQARKALAADSALR
AQPVNMLQAMLLAAEKPGSGVNHAVISGNVSTMLLGGQETTASALAWLIWLLWRNPTALK
QAQQEVLNKVSDTRTMTPEQMDSLDYVEACALEAMRLKPPAPFLPLQALCETTVLDVRLP
ADTLLWCVLRHDSVAEAFMPAAQRFNPERWLGDDGAARRLSLPFGAGPRMCPGRYLALME
IKVAMVTLLANFDVSQVRPLTGAAPNEVMGFTMMPTPLAITLRQRESAALD

>YQ44_23945|KEGG|CYP1097A1|[Janthinobacterium sp.]jaz|BP
MPPLDHAAAPAIARPLRQIRRLPGPFALPIVGNSLQIKLTRIHRDVEQWVQRYGPYLRAW
FGRTLVLVVADSEAVAAVLRDRPDGFRRPLSSFIVSEEMGGIPGLFLAEGAQWRNQRRMV
MAGMAPGAIKAYFPALVSVAQRLRRRWHLAATQGQAIDLDGDLKRYTVDIIAGLAFGTEV
NTLESGEDVIQRHMDDILPAVARRSLSMFPYWRYVKLPIDRRLDRDVAALSAAVADLVQQ
ARTRMAHDPARRERPPNLLEAMIAAADEKDSGVDDRAVAGNVLTMLLAGEDTTANTISWM
IYLLQRHPRSLARAREEVRRLAPDAAHFTIEQMDSLDYLGACASEAMRLKPVAPFLPVEA
LRDTVIADIAVPAGTLVWCVMRNDTVADAHFPDAQAFQPERWLAQSGEANNKRASMPFGA
GLRTCPGRYLALLEIKIAMAMLLGSFDIAGVDTPDGGEAQELMGFVMSPIGLSLRLARPQ

>BZG29_04195|KEGG|CYP1097A1|[Janthinobacterium sp.]jal|BP

MPPLDHAAAPATTALRQIRRLPGPFALPIVGNSLQIKLTRIHRDVEEWVRRYGPYLRAWF
GRTLVLVVADSEAVGAVLRDRPDGFRRPLSSSLVSEEMGGIPGLFLAEGTEWRNQRRMVM
AGMAPGAVKAYFPALATVAQRLRRRWHLAATQGQAIDLDGDLKRYTVDIIAGLAFGTEVN
TLEAGEDVIQRHMDDILPAVARRSLSMFPYWRYVKLPVDRRLDRDVAALSAAVADLVQQA
RTRMARDPARRERPPNLLEAMIAAADLEGSGVTDRNVAGNVLTMLLAGEDTTANTLSWMI
YLLQRHPHTLARAREEVRRLAPDAARFTIEQMDSLDYLGACASEAMRLKPVAPFLPVEAL
RDTVIADIAVPAGTLVWCVMRHDTVSDAHFPDAQAFQPERWLAEGGEANNKRAAMPFGAG
LRTCPGRYLALLEIKIAMAMLLGSFDIAGVDTPDGGEAQELMGFVMSPIGLTMRLARPQ

>CNX70_04245|KEGG|CYP1097A1|[Janthinobacterium svalbardensis]jsv|BP

MSMPPLDHAATPDIARPLRQIRRLPGPFALPIVGNSLQIKLTRIHRDVEQWVQRYGPFMR
AWFGRTLVLVVADSEAVGAVLRDRPDGFRRPLSSSVVSDEMGGVPGLFLAEGTEWRNQRR
MVMAGMAPGAIKAYFPALVTVAQRLQRRWHLAATQGQAIDLDGDLKRYTVDIIAGLAFGT
QVNTLESGEDVIQRHMDDILPAVARRSLSMFPYWRYVKLPVDRRLDRDVAALSAAVADLV
QQARTRMAHDPARRERPPNLLEAMIAAADLEGSGVTDRNVAGNVLTMLLAGEDTTANTVS
WMIYLLQRHPHTLARAREEVRRLAPDVARFTIEQMECLDYVGACASEAMRLKPVAPFLPV
EALRDTVIGDIAVPAGTLVWCVMRNDTVADAHFPDAQVFQPERWLAEGGEANNKRASMPF
GAGLRTCPGRYLALLEIKIAMAMLLGSFDIAGVDTPDGGEAQELMGFVMSPIGLTLRLAR
PQ

>EKL02_04155|KEGG|CYP1295B1|[Janthinobacterium sp.]jaj|BP

MKTSDHDNKIPPGPSAPWLGLPLLGEMKNDLLGTAQRMREQYGDVVHFRMASQPYYYFFS
PELIRELLVEHADDLIRHERAIEVFSLVYGDNVLTTEGDTWKRQRRILMPGFLPKKITGY
VDLMTGAVADAMVTAFPEKGPAGNVLDVDSFTTVLTMDVILRVLFSHKVSEAESQRAFEA
SRALEHQGMRELFWPKTPPDWFPYPGRKQKLKSKADLENLIGSQVQVRRAAGEDQATKTD
YLAMLLSAHDDEAQGASSSPTLSTEEIRDNCMVIFAAGHDTTATVMTWWIGLMAHHPEVA
EKVRQEVAEVAGDRNPTADELSRMKWMNATIKEAMRLYPPTPNLFFRCPVRDIEIGGWHV
PKGASINVPVWHVQHDARWFPEPESFRPERFMPNAAEIPRGAYMPFGAGPRVCIGQHLAT
IEMALIATFLIRQFDLSPGDAAGLPSPKIDMVLKPETTLRVKFTRR

>EKL02_04905|KEGG|CYP1098B4|[Janthinobacterium sp.]jaj|BP
MSRFCPVYPKPHKARSGALRLFSSLRRSWLDGLVERNYQMQMAEVKFPGLDLYIVNDPKL
VREAMVDEYANFPKTDLLGKLLDPLIGPSIFTTNGRQWQIQREMVGTGFSHMRLKTAFPL
MMAAVDAMEARVAALPDGIVTDIDQEMTHVTADIIFRTILSLSMESEDARKIFHNFNIFQ
KMAPRQITPIMFGLPRWLSPWLDIYRSNRAAREIRSLLEKFIRPRFDAHRAGNPGEHQDI
LSALLGAKNPDTGDGFAFSELVDQVGMLFLAGHETSASALAWSLYLISMSPEIQERMHAE
LSAVVGDGDIEYGHVKDLELTWNVFRESLRLYPPVGFFVRQAKVGRTMRGKNVAKDAIIV
IAPWLIQRHRDLWERPDEFDPDRFASDSAKESLKCAYLPFGMGPRVCIGASFALQEAALI
LATLVRRFRFAAPPDGQAPQPVGRLTIRSENGVRVTIHKRTAT

>EKL02_15905|KEGG|CYP1098B2|[Janthinobacterium sp.]jaj|BP

MSRFCPVYPKPRKNKASALLMLFGKRRSWLDGLYERSYGMKMGEVKLPGAQLYMVNEPPL
VRRILIEQAAKFPKHTMLGKLLAPLLGESIFTTNGAQWQRQRTMMDGSFAQTRLKLVFPL
MKAAAAAMKERLDKLEDGAEYDIDLEMTHVTADIIFRTILSQTLEGETSRKIFEEFTRFQ
EMAPRILNPMLFKLPRIFSPWLAIRRSNRAAREIRAQLEVFIRPRYDAHRAGTPGEYQDI
LASLLDAVDPVAGTAFSFDELVDQVAMLFLAGHETSASALSWAMYLISHRAEIQERMHAE
AMAVMGEREPDYSDIKSMELIWNVFRETLRLYPPVGFFAREATETQGMRDKTVHEGASVI
IAPWLIHRHRELWERPDEFDPDRYGSASARESLKCAYLPFSLGPRVCMGATFALQEAGLI
LATLVKHYRFEAPAGYTPEPVGRLTVRSENGVRVIIRKRKQGNGEAA

>FJQ89_21240|KEGG|CYP1097A1|[Janthinobacterium tructae]jas|BP

MSMPPLDHAAAPDIARPLRQIRRLPGPFALPIVGNSLQIKLTRIHRDVEQWVQRYGPFMR
AWFGRTLVLVVADSEAVGAVLRDRPDGFRRPLSSSVVSDEMGGVPGLFLAEGTEWRNQRR
MVMAGMAPGAIKAYFPALVTVAQRLQRRWHLAATQGQAIDLDGDLKRYTVDIIAGLAFGT
EVNTLESGEDVIQRHMDDILPAVARRSLSMFPYWRYVKLPVDRRLDRDVAALSAAVADLV
QQARTRMAHDPARRERPPNLLEAMIAAADLEGSGVTDRNVAGNVLTMLLAGEDTTANTVS
WMIYLLQRHPHTLARAREEVRRLAPDVARFTIEQMDSLDYLGACASEAMRLKPVAPFLPV

EALRDTVIADIAVPAGTLVWCVMRNDSVADAHFPDAQVFQPERWLAEGGEANNKRASMPF
GAGLRTCPGRYLALLEIKIAMAMLLGSFDIAGVDTPDGGEAQELMGFVMSPIGLTLRLAR
PQ

>G3257_04380|KEGG|CYP1097A1|[Janthinobacterium lividum]jlv|BP

MSYSFKTRARKRATVMPSFPLKQELAMPPLDHAAAPDIARPLRQIRRLPGPFALPIVGNS
LQLKLTRIHRDVEAWVRRYGPYMRAWFGRTLVLVVADSEAVGTVLRDRPDGFRRPLSSSI
VSEEMGGIPGLFLAEGTEWRNQRRMVMAGMAPGAIKAYFPALVTVAQRLQRRWHLAATQG
QAIDLDGDLKRYTVDIIAGLAFGTEVNTLESGEDVIQRHMDDILPAVARRSLSMFPYWRY
VKLPVDRRLDRDVAALSAAVADLVQQARTRMAHDPARREHPPNLLEAMIAAADLEGSGVT
DRNVAGNVLTMLLAGEDTTANTISWIIYMLQCNPHTLARAREEVRRLAPDVAHFTIEQMD
SLDYLGACASEAMRLKPVAPFLPVEALRDTVIADIAVPAGTLVWCVMRNDTVSDAHFPDA
GTFQPERWLAESGEANNKRASMPFGAGLRTCPGRYLALLEIKIAMAMLLGSFDIAGVDTP
DGGEAQELMGFVMSPIGLTMRLTQPQ

>Hsero_2448|KEGG|CYP107NP3|[Herbaspirillum seropedicae]hse|BP

MAVQWSTAHCGGAWVISGHEQVAAALRDPRFSVRRAARWINSGLGGKQADSAAERRFKRL
FSRSLLFLDGRAHRRLRGVLNPGFKPAQLALQAPAIEALAQQLIDDILRDPQSPHGFDFI
SRFARPLPALVIARLMGLPGAVPPAFIDWAADLAAFIGSPTPDAAQTLAAQHAMQALCDF
FSSTLEQEQGQALPPHSLLAQLLQARQQGQINQVELLAQCSTLLFAGYETTRNLLGNGLL
ALLRHPEQWRLLKSEPHWLRSAIREMLRHDSPVQYTGRRLLAPVQLDGQTLARGDLAILH
IGLANHDAKRYHQPQDFDIRRDQGMHLSFGQGPHVCLGAALTQMEAEIAFTALMRRLPTL
ALDTAAPVWQDNPAYRALARLPVVFTRNHHA

>ACP92_12235|KEGG|CYP107NP3|[Herbaspirillum seropedicae]hsz|BP

MAVQWSTAHCGGAWVISGHEQVAAALRDPRFSVRRAARWINSGLGGKQADSAAERRFKRL
FSRSLLFLDGRAHRRLRGVLNPGFKPAQLALQAPAIEALAQQLIDDILRDPQSPHGFDFI

SRFARPLPALVIARLMGLPGAVPPAFIDWAADLAAFIGSPTPDAAQTLAAQHAMQALCDF
FSSTLEQEQGQALPPHSLLAQLLQARQQGQINQVELLAQCSTLLFAGYETTRNLLGNGLL
ALLRHPEQWRLLKSEPHWLRSAIREMLRHDSPVQYTGRRLLAPVQLDGQTLARGDLAILH
IGLANHDAKRYHQPQDFDIRRDQGMHLSFGQGPHVCLGAALTQMEAEIAFTALMRRLPTL
ALDTAAPVWQDNPAYRALARLPVVFTRNHHA

>Hrubri_2195|KEGG|CYP107NP2|[Herbaspirillum rubrisubalbicans]hrb|BP

MSVQWSAAHCGGAWVISGYEEVATALRDPRFSVRRAARWINSGLGQPATGAVQDAAQLRF
KRLFSRSLLFLDGRAHRRLRGVLNPGFKPADLEAQRPAITRIAQQLIDAIVADPKAHQGF
DFIDRFARPLPALVIASLMGLPEQVPPQFIDWASDLATFIGSPTPEAQQTRAAQAAMAAL
CDFFAQALEHPQTLPADSLMQRLLQARQAQQLNQNELLAQCCTLLFAGYETTRNLLGNGL
LALLRHPEQWTALKAELHRGKPDLRPALREMLRYDSPVQYTGRRLLADVEMGGHSLRRGE
LAILHIGQANHDHRRFAQPQCFDIRRDQGMHLSFGQGPHVCLGAALTQMEAEIAFTLLMQ
RLPTLALDHHHAPPRWHSHAAYRALESLSLVF

>E2K99_12915|KEGG|CYP107NP1|[Herbaspirillum huttiense]hhf|BP

MPLHWSTAHCGGAWVITGYEEVAAALRDPRFSVRRAARWINSGLGDTGNGQQDEAQRQFK
RLFSRSLLFLDGRAHRRLRGVLNPGFKPADLQARRGDITALAQRLADDIVTDPQAARGFD
FIARFARPLPALVIAQLLGLPDRVPAEFIDWAADLAAFIGSPTPDATQTLAAQQAMRSLC
DFFSQALARADELPEACLLRGLLQAHASGQLNRNELLAQCCTLLFAGYETTRNLLGNGVL
ALLQDPVQWAALKARQADPAALRMAVREMLRHDSPVQYTGRRLLQDVTLAGQRLQRGQLA
ILHIGQANHDARRFRDPQRFDIRRDEGMHLSFGQGPHVCLGAALTQLEAEIAFSVLMQRL
PTLSLADETPVWQSNAAYRALDRLSVFF

>G5S34_13020|KEGG|CYP107NP4|[Herbaspirillum frisingense]hfr|BP

MSVQWSAAHCGGAWLISDYEAVAAGLRDPRLSVRRGARWINSGLAGLDALDSAPAELQAQ
RRFKRLFSRSVLLLDGRAHRRLRGVLNPGFKPTDLEAQRPAITRIAGQLIDAIVADPLAP
QGFDFVSRFARPLPALAIAGLMGLPDRVPAAFIDWAADLAAFIGSPTPDAGQTLAAQAAM
DALCDFFGEVVKDADTLPPDSLVARLLQAQREQRINRNEMLAQCCTLLFAGYETTRNLLG
NGLLALLQHPAQWTALKADPTLLRTALREMLRYDSPVQYTGRRVLTDLQVAGRTLRQGEL

VILHIGQANHDGRRFTGPQHFDIRRDEGMHLSFGQGPHVCLGAALTLLEAEIAFTALMQR
LPTLALSATPPRRQQHAAYSALDRLPLVF

>CFU_4256|KEGG|CYP107DG6|[Collimonas fungivorans]cfu|BP

MINEDFLNNPYPAYHALRAAGALHWSPEFCGGAWLLTGHADVASVLRDPRFSVRRAGGWA
NSSGPQALTELREFKRIFSRSLLFVDAPQHTRLRQVMNAGFKPSALQELGPQIQEIVDRL
LDQVLAKAAEAAPGQFTEFDFMRDFARPLPALVIAGMLGIAAADRSEFVAWSDDIAAFIG
SPTPTLEIARAAQVSLVAMNEYFRQLLPQRRAALGDDLMSQLIRAEASGGIITTKELLAQ
CCTLLFAGHETTRNLLGNGMLALLQHPEQWQALQDTPALLPSALKELLRFDSPVQYTGRR
LKVDVEMHGQRMKKGDLVIPLIGAANRDPAKFTDPDRLDIQRNQGAHLSFGYGPHVCIGA
TLTYLEAEIAMRSVMQRLPQLRLAGAAQSWGGNAVYRSLNRLPLQFARPAAAAHLEPAEV
LYA

>LT85_4854|KEGG|CYP107DG6|[Collimonas arenae]care|BP

MINEAFLNDPYPAYHALRAEGALHWSPEFCGGAWLLTGHADVASVLRDPRFSVRRAGGWA
NSSGPGALNELREFKRIFSRSLLFVDAPQHTRLRQVMNAGFKPAALQELAPQIQIIVDRL
LDDILSKAGTTANGQFAEFDFMHDFARPLPALVIASMLGIAAEDRSEFVAWSDDIAAFIG
SPTPTLDIARAAQVSLIAMNDYFRQLLPQRRAALGDDLMSQLIRAEANGGIITTKELLAQ
CCTLLFAGHETTRNLLGNGMLALLQHPQQWQALQAAPSLLPSALKELLRFDSPVQYTGRR
LKVDVEMHGQLMKKGDLVIPLIGAANRDPAKYTDPDRLDIQRNEGAHLSFGYGPHVCIGA
TLTYLEAEIAMRSVMQRLPQLRLASATQSWGVNAVYRSLNALSLQFSPPAPAAESPVVEA
DYA

>CPter91_0565|KEGG|CYP1199A10|[Collimonas pratensis]cpra|BP

MKLMQAIGSDVRAALKRPVSKTVDFSGEWPRVGHVYLRDLILGEDPYRLRFLMDRILELT
PKLTWAVIAAGAASSVRLRPEASAIAKLTASASTYNDRRYAMGLYRRTAAPVCFTISTLV
ANALWLGSPFDKDTSNRNILYEALRLLPPSWNILRSASPEYCALDSRIGPNDDVLILPLL
SHRDPAIWDDADEFRPERWDSLNPSEQPGYLPFGHTSERCWGQHMVMPLAERLLDIIRNN
GLAVSPRQITADVPLAGLLGVFQVSVVRQ

>CPter91_5329|KEGG|CYP107DG6|[Collimonas pratensis]cpra|BP

MINEAFLQDPYPAYHALRAEGALHWSPEFCGGAWLLTGHADVASVLRDPRFSVRRAGGWA
NSSGPEALTELREFKRIFSRSLLFVDAPQHTRLRQVMNAGFKPAALQELAPQIQNIVDRL
LDKVLADAAAPPSGQFAEFAQFAQFDFMRDFARPLPALVIASMLGIAAEDRSEFVAWSDD
IAAFIGSPTPTLEIARAAQVSLIAMNEYFRQLLPQRRAALGDDLMSQLIRAEATGGIITT
KELLAQCCTLLFAGHETTRNLLGNGMLALLQHPQQWQTLQADPALLPSALKELLRFDSPV
QYTGRRLKVDVEMHGQLMKKGDLVIPLIGAANRDPAKFSEPDRLDIRRNEGAHLSFGYGP
HVCIGATLTYLEAEIAMRSVMQRLPQLRLAGSMQSWGGNAVYRSLNALPLQFAHPAAAAG
APVVETAAKTVAEAVHA

>C9I28_12805|KEGG|CYP1097A16|[Massilia armeniaca]masz|BP

MLGSALQLRPRTIHRTMEAWSRQYGPMFRVRLGTREAVVLADAQAIGAVLRDRPDGFRRP
GVTAEVTRELGGLPGLTLAEDDDWRRQRRMVMQAFAPGFVKAYLPRLAQVGVRLVARWRQ
AAMAGQPIPLSADLKRYAVDVVAGLAFGADVDTVNAGTSDLQDHIDTVLAGVARRSLAPL
PYWRWLPLPAERRLAASVTALQGAIDGFVASGRMALAADPALRARPANLLQALIAAADEP
GSGLGDAEVAGNVATMLIAGEDTTASALAWTAWLLARHPAALCRARDEVRATVPDLARIT
PAQADALDYVEACALEAMRLKPPAPFIPLQALRERTVLGVTLPPGTLLWCVLRHASVDAA
LLACAAEFRPERWLPGGDATTPAARQAILPFGAGARACPGRYLALLEMKVALAALLGHFE
LDSVTAAGGAEPAEVLGFVMAPAPLKMTLRPRVPGAGDLSAA

>C9I28_19965|KEGG|CYP1355B1|[Massilia armeniaca]masz|BP

MPLPVTLNPAVRPAAEGPPAARRVHELPKPKGEPVIGNMRQLDGLRFHATLENWARELGP
LYRFTVLNRTMVVASSRPALTALMRERPDLIRRGSRTSVMMEEAITPGVFSAEGEDWRQQ
RKLVMRALTPEVVHNFFPTLAALTERLRLRWVAAAEAGRKIDVLRDLKAWTLDVTIGLAM
GHDIDTLQNEHNPLQRDIETVFERIARRLTTPVPYWRLFRLAVDRESDAAAQRISDAVAG
FIANARARIAANPALRAKPSNMLEALVVARDEPGSGFTDEAVIGNAITMVFAGEDTTSNT
IGWLLDFLVRHPDCNAAVAREADAVLGADKVLQDYRLLDSLPYLDAALREGMRLKPVAPF
MILEPVADIVIDDTLIPAGTPIFTLLRHSFERDNTLVEPELFRPERWLAGSEPGADDPAR
KLFPFGGGPRFCPGRYLAMAEMKIAVSMLAHQFTPVLDPGAPAVEERFTFTMTPSVLPVL

LQPRR

>DPH57_02135|KEGG|CYP1355B1|[Massilia sp.]masy|BP

MHPAVRPAAEGPPATRRLGDLPKPKGEPVIGNMRQLDSLRFHRTLENWARELGPMYRFKV
LNRTMVVTSSRPALTALMRERPDLIRRESRASGIMEETITPGVFSAEGENWRQQRKLVMR
ALTPEVVHNFFPTLVMLTERLRLRWVAAAEAGRRIDVLRDLKAWTLDVTIGLAMGHDIDT
LQNEHNPLQRDIETVFERIARRVTTPLPYWRLFRLAVDRESDAAAQRISDAVAGFIANAR
ARIAADPALRARPSNMLEALVVARDEPGSGFTDEAVIGNAITMVLAGEDTTSNTIGWLLD
FLVRHPDCGAAVAREADAVLGADKVLQDYRLLDSLPYLDAALREGMRLKPVAPFMILEPV
ADIVIDDTLIPAGTPVFTLLRHSFERDNSLVQPELFRPERWLASGESGVDDPGRKMFPFG
GGARFCPGRYLAMAEMKIAVSMLARQFTPVLDPGAGPVEERFTFTMTPSVLPVLLQPRR

>DPH57_08810|KEGG|CYP1097A16|[Massilia sp.]masy|BP

MPDRLIATLPGPRGLPLLGSALQLHPRTIHRTMEAWSRQYGPMFRVRLGARTAVVLADPD
AIGSVLRDRPDGFRRPAVTADVTRELGGLPGLTLAEGDDWRRQRRMVMQAFAPGFVKAYL
PRLAQVGVRLAARWQQAAVAGGPIPLSADLKRYAVDVVAGLAFGADVDTVNAGTSDLQDH
IDTVLAGVARRSLAPLPYWRWLPLPAERRLRASVTALQAAIDGFVASGRAAMAADPALRA
RPTNLLQALIAAADAAGSGLGDAEVAGNVATMLMAGEDTTASALAWLAWLLARHPAALRR
AQDEVRAKVPDLASITPAQADALDYVEACALEAMRLKPPAPFIPLQALRATEVLGVTLPP
GTLLWCVLRHASVDDALLTCAAGFQPERWLPGGDATTAPARQAILPFGAGARACPGRYLA
LLEMKVALVALLGQFDLDSVAAADGAEPAEVLGFVMAPAPLTMVLRPRAAKRLNAA

>DPH57_15060|KEGG|CYP229L4|[Massilia sp.]masy|BP

MTVSAPPADALAAVSHPDPYPYYAALARQEAPFHDERLGLWVAAHPGTVRAVLAQADCRV
RPPHEPVPPALAGPAGSVFGALARMNDGARHELPKAVLLEALAALPAEAVAAHVRRTADA
LLVQGVALTTFAFELPVRAVASLLGCTDPAVAGHVRSFVAGLAARADAAAIAAAHDAVPH

LLALADDIESDAPLLRAVQEAARRHGWTDAAALRANVLGLLSQTYEATAGLIGNGIVARL
RGDARPAAALVDHVLRTDPPVQNTRRFTAADVDIAGVRIAAGQTILLVLAAAGEPFGHGR
HACPGQALARAIALHALAALQEHGPLPDVAWRYRASPNGRLPEFIEGEGK

>EYF70_23845|KEGG|CYP229L2|[Massilia albidiflava]mali|BP

MEPDHPPADAVAAVTHPDPYPYYAMLAADPAPRYDERLRLWVAAHPATVRQLLADPACRV
RPVQEPVPAALAGPAGDLFGALVRMNDGPRHATPKAVLLHALAALPQSLAADHAASVATA
MAAEVCDAATLNAFVQGVPVRAVASLLGFADGELPRVAALVARYVACLSPLACPGEIAAG
HEAAQALLDALRQLVRRAPGTALLAGVTREPWPDEHALLANLAGLMTQTHDATTGLLGNC
IVARLRGDASGPAALVPAVMEHDPAIHNTRRFTAAGLDVAGVGVPAGQALLLVLAGTAGF
GDGHHACPGQTLARSIVTQALRALRAAGPLPRAAWRYRPSVNARMPVFVGEGGA

>EYF70_29330|KEGG|CYP1097A15|[Massilia albidiflava]mali|BP

MPQPTPHPRAIADLPGPRGLPLLGNALQLKPATIHLAMERWSREYGAMFRARIGASEVVV
LSDSAAIMTLLRDRPDGFRRPAITAEVSAELGGSVGLFLAEGDVWRDQRRMVMHAFSPTA
IRAYFDRMVQVAQRLEARWLAAGDRDIDLNADLKRYAVDVIAGLAFGADVNTLESGGDSI
QEHVETVLAGVARRSLLPFAYWRWCRLPADRRLERSVRAVREATDGFIAAARAAMATEPA
LREHPANLLQAMLAAAEQPGSGVDDDAVAGNVSTMLLAGEDTTSSALAWLIWLLWQNPPA
LERAQAEVRQHARELTVENIAALDYLDACALEAMRLKPPAPFLPLQAMRDTTVLGVHLPK
DTLLWCVMRHDSVGDKAFPDPLRFDPERWLAGTVDKGVALPFGAGPRMCPGRYLSLMELK
VAMAVLLGRFDIAGVRARDRSEPAEVMGFVMGPTPMAMRLRPRAE

>FCL38_05710|KEGG|CYP226A25|[Massilia umbonata]mum|BP

MTVIKSVSLESAYSGVADNYRGEDVDLHAIYRDMRRNSPIIAENFMGRLGVPNIAGLDAN
RPTFTVFKYKEVMAVLRDAANYTSGFIAEGLGSFFDGLILTGMDGDAHKKARALLQPIFM
PEVVNSWRDTKMAPIVREEFLQPLVAHKKADLMDFGLLFPIRVIYALIGFPDNDPEKVKQ

YAAWALAILAGPQVDAEKAAQARQAAMEAAKALYDATHAAVVEVRRNGAAGDDLISRLIR
AESDGRRFDDHEVTTFVRSLLPAAAETTTRTFGTTMTLLLERPELLARVRADRSLVPKAI
DEAIRLEPVATFKVRQAAQDLELAGVAIPKGAMVQCIVSSANRDEEAFERSEEFDIDRKP
KPSFGFGFGPHMCIGQFIAKTELQVAINGVLDLMPNVRLDPEQAPPRITGAQLRGPKHVH
VIWD

>FCL38_05735|KEGG|CYP226A20|[Massilia umbonata]mum|BP

MSTDSDTIDLESAYTAVSDTYKGKGDIHAACRAARKASPIFVGDFIRQFGVPTNAGMQQG
TRPTFALFRHQDVMAVLRDASSYTSGFIAEGLGAFFDGLIILAMDGEQHRQVRALLQPAF
MPETVNKWRDQIDAVIRKDFLEPLAGNKGADLMKFGLEFPIREMYALMGFPTDNPDAYRR
YAGWALAMVGGNQVDPAKADEARRNAGIAVRLLYEAILEVVAQRRVDGSQGDDLIGRLLR
AEHEGRRLDDHEVTTFVRSLLPAAGETTTRTFSSVMALLLTTPGLQERVRADRALVPKLI
DEAVRFEPVATFKVRETAREVQFHGVTIPKGAFVQCMVASANRDEEVFERPDEFDIDRKP
KPSFGFGFGPHMCIGQFVAKMELNCAVNAILDLFPGLRLDPAMPAPVIEGAQLRGAAHLH
VLWD

>FCL38_05785|KEGG|CYP107DG7|[Massilia umbonata]mum|BP

MVRQAWPLPCQPAPPANTPLPLFDGLFLQDPYPTYRALREQGPVHWRDDVFQGAWILPHY
ADVAFALRDPRFSSQRTGGWIKRVPDAATPAADGRGQAGRDAFQRMFARAMVFLDAPDHR
RLRRAMAAGFHPSRIRALAPRIEQLADELFDPLQHCPQFEFIGAVARPLPSRVMALLLGV
DRSDEARFMAWSDSLATFIGALQPSADELRGARRGLMQMAGYFDGLLRERRQAPGTDLIG
CLLQAEAAGEIHADGELVAQCAMLLFAGHETTRNLLGNAVHTLLSHPAQWRALRACPEAM
PAAIREILRYDSPVQYTGRRVAATLTLHGHTLRRGDLVLALIGAANRDPARFAAPDTFDI
ARRESSHLSFGSGPHVCIGATLALLEAEVTLRQMARRWPDLRLAESTTSWNGNPGLRGLT
KLQLRTVG

>FCL38_09965|KEGG|CYP152B8|[Massilia umbonata]mum|BP

MKTNAHSTAAPASRALPRDGRLDATLALKRDPYGYLRSQCERLGSDAFQTRILLQRTICI
TGPQAAQLFYDPQRFMRKGAAPMRLQATLFGRGGVQAMDDAAHGVRKAMFMNLMTPSRIG
ELGDLFDETWRAAMHRWAAAPRVELYAAVREVLTRAVCRWAGVPLAEAEVPLRTKQLSAL
FDLAGAVGPWHWFSRYARFRAQQWCGGVIERIRAGQLAVPPDSAAYAVARHVDADGRRLD
ARTAAVELLNVLRPTVAVAVYIVHEALALHAFPACRAPLRDDTDGRYAGWFAQEVRRFYP
FFPAAVARVRQDFDWQGWHFPKGRRVMLDLHGTNHDPRAWQEPLRFQPERFATWNDNPFS
FVPQGGGNAATGHRCPGEWITLELMQRAARWLAGGMGYEVPPQDLALDMTRLPAIPKSGF
MIAVHGD

>FCL38_20285|KEGG|CYP1104B1|[Massilia umbonata]mum|BP

MSTAALKAPAPRTIADLPAPPGLPLLGNALQMKPSTVHLAMERWCRRYGPIFRARLGRYE
TVVLADSDAIAAVLRDRPDGFRRPVITAEVSAELGGQVGVFIAEGDAWRNQRRMVMSAFS
PTAIRAYFERMAQVAQRLEHRWRAAGEREIDLSGDLKRYTVDVVAGLAFGADINTLDSGG
DAIQEHVETVMAGVARRSLLPFAYWRWFKLPADRRLERSVRAVRDATDGFIAEARAAMAA
DPTLRERPANMLQAMLAAADQPGSGVDESAVAGNVTTMLLAGEDTTASALAWLIWLLWQN
PEALARAQREVRLHAPALTVESIGALDYLEACALEAMRLKPPAPFLPLQALRDTTVMNVH
LPKGTLLWCALRHDSVDAGVFPEPQRFDPERWLPGGGGNRQVALPFGAGPRTCPGRYLSL
MELKVAMAVLLGRFDIVEVRARDRIEPAEVLGFVMGPTPLAMRLRAA

>FCL38_25475|KEGG|CYP229L3|[Massilia umbonata]mum|BP

MDTDHWPADAVAAVTHPDPYPYYTALAVDPAPRYDERLRLWVAAHPGTVRELLANPDCRV
RPVHEPVPAALAGPAGDLFGALVRMNDGARHAAPKAVLQRALAALPEEAAADVAAAVAAN
MVADQAADKAADTAAALNAFVHGVPVQTVAALLGFTDGELPRVAALVARYVACLSPLASA
SDIVAAHEAEAALVDALRRLVRDGRQTALLASVAAAPWPDEHAMLANLAGLMTQTFEATA
GLLGNCIVARLRGDASDPAVLAPAVMARDPAIHNTRRFAAGDLRIDGAHVPAGQALLLVL
AGAAPFGDGRHACPGQALARIIATQALHALPASGPQAAGPVPGVTWRYRPSVNARMPVFI
GESKR

>GO485_20845|KEGG|CYP229L1|[Massilia flava]mfla|BP

MERNTEPGAWPADPIAAVTHPDPYPWYAHIAAEPKLVYDERLKLWVAAAPTLMREVLAHP

DCRVRPVHEPVPAAIAGPAGALFGALARMNEGPRHASPKAALLHALATLDEQAVRACADR
VAAPLAAGVADAQTLNAYLFAAPVRTVAALLGWPDERLAQVEGWTREFVACLSPLSSAAA
IAASHGAADALLAALRELVRAECTNPGKRHLLAGVLAAPMPDEHTLLANLAGLLSQTYEA
TAGLLGNCVTALLRDSAADRSTLVERVSRADPAIHNTRRYAACDVDVGGVRVTAGEGILL
VLATADGFGHGRHACPGQALATTIVTRALLALPAGLPALGWHYRPSVNARIPVFMEAV

>GO485_27915|KEGG|CYP1097A12|[Massilia flava]mfla|BP

MSQTFKTAAARGGTLGASQHQHEDQKMSETMSTTLPGHPAARDIADLPAPPGLPLIGNAL
QLHPRTIHRTMEDWSRRYGTMFRARIGSRQVVVLADGAAINAVLRDRPDGFRRPAVTAEV
SDELGGNPGVFLAEGDAWQRQRRMVMHAFSPAAVKAYFGRLAHVARRLEAGWLQAAAQGH
EIDLTGDLKRYAVDVIAGLAFGADVNTIEAGDDAIGRHVDTVLAGVARRSLMPFPYWRWV
RLPADRRLERSVLAVRSATDGFIAGARAALAADPGLRERPANLLQAMLVAADHPDSGVDD
AAVAGNVTTMLLAGEDTTSSALAWLIWLLWRNPAALARATDEVRRHVPDVAALTYEQTEP
LDYVQACALEAMRLKPPAPFLPLEALRETTVLDVRLPPGTLLWCVMRHDSVDARCFPSPD
TFDPERWLPDAQGAHRADRQAALPFGAGPRICPGRYLSLLEIKVAMATLLSRFDITAVHA
TGRPEPAEVMGFVMGPSPLTMTLRPRA

>E1742_07130|KEGG|CYP1355B1|[Massilia plicata]mpli|BP

MPPALLQPAVRLAADGPPPTRRVRDLPAPQGSPIIGNMRQLDGAYFHRTLENWARELGPL
FRFTVLNRSMVVSSDRTALTALMRERPDLIRRGARTSAMMEEAITPGVFSAEGDDWRQQR
KLVMRALTPEVVHNFFPTLAALTERLRLRWARAAAEGRRIDVLRDLKAWTLDVTIGLAMG
HDSDTLQQEDNPLQRDIELVFARIASRLTTPVPYWRVFRLAVDRESDAAAQRISDAVAGF
IAIARRKMEANPALRARPSNMLEALVAARDEPGSGFTDQAVIGNAITMVFAGEDTTSNTI
GWLLDFLVRHPEQLAGVTREADGILGTGNVVQDHRLLDNMPYLDAAIREAMRLKPVAPFM
ILEPVADIVMDDTLIPAGMPIFTLLRHSFERENTLTNPELFQPERWLAQGTAGADDPARK
LFPFGGGPRFCPGRYLAMAEMKMAMSMLAHGFTPLLDPAAPPVEERFTFTMTPSVLPVLL
RPR

>HH213_01020|KEGG|CYP1355B2|[Duganella sp.]dug|BP

MELTPGLAPLRHIADLPGPKARPLLGNVREVRAQPFHQVLEGWGREYGPLYRFRIMRKQM

LATADREMIVALLRDRPDMMRRSSRTAEMLNEIGTRGLFTAEGEDWRRQRKLVMRALTPE
VIHHFFPMLAAMTDRLRLRWESALAAGQPVDLLRDLKAYTLDVTIGLAMGQDINTLELEE
HPLQRDIEFLFKLVARRLTTPFAYWRVPVLKRAQDHEAIAASARIQQAIFGFIADARKRL
AENPARRLKPGNLLEALIVARDEPDSGFTDEAVVGNAITMVFAGEDTTSNTAAWLIDFLA
DHPAAAATMAQEAQQVLGERAVLADYHLLDQLKYIDAATREAMRLKPVAPFMAAEPNQDV
VLGDLVVPAGTVIFLLLRHASERECELPQPERFLPERWLDGAAAHGDDPGRKLLPFGGGP
RFCPGRYLAMAEIKMVMSMLVRHFALERVVGAPAARECFTFTMTPDSLPVRLARKT

>HH213_03910|KEGG|CYP1097A19|[Duganella sp.]dug|BP

MYNTIAAELRHIADLPGPRPWPLAGNLPQITPTRLHQDLEGWAKQYGPLFKISFGRTQML
VVSSHELVNAVLRDRPDGFRRPSITATVSDEMGGRPGLFLAEGTAWRDQRRMVMTALAPH
AVKAYFPSLVAVGLRLQQHWQRAAREGRVIDLTEDLKRFSVDVIAGLAFGSDVNTIDGGE
DVIQRHMDIVLPAVARRSMALFPYWRYVRLPQDRQLDASLLALNQSIDGLITIARARLAA
EPERRVRPANLLEAMICAADEGGSGVDDVAVAGNVATMLLAGEDTTSNTLAWMLYLLQQN
PAAMQKAREEVLRVAPDPRAFTIEQMDGLDYLDACAQEAMRLKPVAPFIPMEALRDSVVG
DVQVPKGSLVWCVMRNDSVSNDFVADAADFHPERWLAGAAQPMNKQVSMPFGAGVRTCPG
RYLALLEIKLASAMLLSSFDIASIGTASGAEPQELDGFTMSPIGLQMRL

>EJN92_09775|KEGG|CYP229M2|[Undibacterium parvum]upv|BP

MFPSNPVAAVCHSDPYPYYRSLRVGPALQFDTELKLWVASSAAVVRQVLEHPACVVRPAG
EPVPLAIAATSAGQVFGRLVRMNEGRAHSTPKLVLAQALASLTPEQIAARTSYFSNLLAQ
QHGLPTAAGINAWMFELPVYVVADLLGFAETELSQLAIWMGDFVRCLSPLSRAEQLASAG
LAAQSLLARFGELLAATSTSENSLVSRIQQAARQVGWQNQDAILANLIGLLSQTYEATAG
LIANSLVALLSQNGLQQQMRDTPEQIAAMLQEVCRYDPPVQNTRRFVVQTCSIAGVELQP
GDAILLVLAAASRDESDFPNADEFSLDRPAHTLLGFSHGRHLCPGQTLALGIGTAALQSL
LALTIGLDPTQLQWTYRPSLNGRLPLFQQISPTLTESH

>EJN92_11585|KEGG|CYP1097A13|[Undibacterium parvum]upv|BP

MHSAANAAPSATTAPAAPVTLRSYEDLPGPRAVALFGNALQIDMPQAHLQFEEWCREYGA

LFKVRMGSRKLLVVGEHELIAKILRDRPDGFGRSDRLQEMWHEMGLPGGVFSTNGEVWKR
QRRMVLSSFDPAHVKNYFPALQGVAQRLCGRWQKAAAAGQAIDLQADLMRFTVDTIAGLA
FGAEVNTLESDQDVIQQHLDKIFPALFSRIFAVIPVWRIRRSASDKQLEKSVREVMLAVD
GFVAQARARMQADAGLHQHPTNLLEAMIAAADQPDSGIDDSQVAGNVLTMLLAGEDTTAN
TLAWMIHLLWLNPATLALATAEVRRVCVGSSVPTLEQLSQLDYVEACAHETMRLKPVAPQ
IPQQAHKDTVVGDVMVPAGTMVICLMRTDSVSDAHIAQADRFAPERWLGQGELAHSSSSA
KRIAMPFGAGPRICPGRYLALLEMKMAMAILLLNFEIQNVSTPDGQPARELLKFTMTPVG
LKMQLAARQL

>EJG51_012640|KEGG|CYP229M3|[Undibacterium piscinae]upi|BP

MFPSNPVTAVCHPDPYPYYRSLLAGPGLQFDEGLKLWVASSAAVVRQVLEHADCLVRPLA
EPVPRAIVGSSAGQVFAYLVRMNEGSAHAVPKLALQQTLAALDPARIGARASYFSALLAP
RHGVPTAAGLNAWMFDLPVYVVGDLLGFAEAGLPQLALWMGDFVRCLSPLSDESQLAAAS
VAAQALLARFAELLAAAVPTVDSLLSRLQQAAQQAGWQHALLANLIGLLSQTYEATAGLI
GNSLVALLSQPDLQQQMRLAPEQTAALLEEVCRYDPPVQNTRRFVANACCIAGVALQPGD
VILLVLAAAGRDDQAHPNANQFVLDRPARQLFGFGHGRHLCPGQQLAASIVNAALQSLLA
LPLSLDPAQLQWSYRRSLNGRLPLFQPVCH

>FAY22_07660|KEGG|CYP289A26|[Noviherbaspirillum sp.]nok|BP

MNKEMPQDDWDPRSEAVLDDQIASYDEMRRGCPVAHSEYLHWSLFRHEDVVRALHDHRTF
SNAASSHPSVPNGMDPPQHTLYRRVIEPYFSAQRMHAFEPVCRNIAVSLVDDLPAEGEIE
LMASFAEEFALQAQSAFLGWPAQLNEPLRRWTRKNHAATLARDRKAMEEVAFEFDGYIRA
LLAQRRADRDGTPTDITACLLRERVADRLLNDEEIISILRNWTVGELSTMSASVGILAHY
LAARPALQEQLRGQPSLLPAAIDEILRIHAPLIANRRITTSEVDVGGRRLAAGERVTLMW
ASANRDEAVFGDPDEFRLDRDPSKNLLYGAGIHVCPGAPLARMELRVILEELLRGGRRLE
LVPGKAPVNAVYPASGFAALPLRITS

>JQN73_09080|KEGG|CYP107DG8|[Glaciimonas sp.]glc|BP

MINNGFLNNPYPTYEALRQAGPLHWNEEFCGGAWLVTEYADVASVLRDPRFSVRRAGGWA

NTSGPEALAELREFKRIFSRSLLFVDPPQHTRLRQVMNVGFKPAALQALAPRIQTLVDGL
LEGVMTTTAASFDFMRDFARPLPALVIAAMLGIDGDDRAEFVAWSDDIADFIGSPTPTME
IARRAQISLIAMNDYFRAILPQRRSHPGEDLISQLTLAEASGGIITTKELLAQCCTLLFA
GHETTRNLLGNGVLALLQHPEQWQMLQQNTALLPSALKELLRFDSPVQYTGRRLKVDVEL
HGQRMKKGDLVIPLIGSANRDPAKFTTPDALDITRNQGTHLSFGFGPHVCIGATLTYMEA
EIALRSVMQRLPGLQLAGDTPSWGHNAVYRSLNALPLSYAAALTTSRLEDAVHV

>Lcho_3645|KEGG|CYP107DG10|[Leptothrix cholodnii]lch|BP

MSAVLRPVGQPLGRPIVDAAFLADPYPTYAALREAGPMHWSDEFFGGAWVLTRHADVDAV
LRDDARFSAQRTGGWVMGSDDTTSPDALNSRAELQDFQRLFARAMLFIDAPDHQRLRGVM
NAGFRGDQLERLRPGIERWIDQRFDAIDAKGQAGSGFDFMAQIARPMPAQVIATLMGITE
ADRGDFIAWSDDLADFIGAPMPTRELTRRARISLLAMARYFEQLTAQRRRQPGDDLVSRL
VQGEARGQIQPGAEMLAQCAMLLFAGHETTRNLLGNGLHALLAEPGHWQRLQDEPALLPG
AVRELLRHESPVQYTGRRVATDLVLHGQLLRRGDLVVGLIGSANRDPARYEQPDRLDLAR
RAAPSLSFGAGPHVCIGASLTLMEAQILFSRALQRWPGLQLVDATPRWNGNPVYRGLQSL
RVIAPATPD

>THI_2350|KEGG|CYP107DG9|[Thiomonas arsenitoxydans]thi|BP

MNEVSIRPPCVLGRSIVDRAFLADPYPTYRALREAGPLHWSEEFFGGAWLLTRHQDVETV
LRDARFSAKRTGGWVNDLEHERGELSRFQRLFGRALLFLDSPDHSRLRNLMQSAFRPDAL
TRLRPEIERIADELLDSLNGVGPFDFIAQVARPLPVRVISHILGIVGADTEEVTIWSDDL
ADFIGAPTPTREQACRAQSSLLAMTRFLQDQLETKRTARGDDLLSALLLQRERGTLHDDA
ELLAQCAMLLFAGHETTRNLLGNGMQALLSHTEQWQLLVKDPNLVPDAVREILRYDSPVQ
YTGRRVISDLLFHGQLLRRGELVLPLIGAANRDPARHADPEELDVRRTDRGTLSFGSGPH
VCLGAALTRIEADVVFRQMLARWPDLRLASAEPQWFANPAYRGLKTLSLRPAM

>RGE_22720|KEGG|CYP152AR3|[Rubrivivax gelatinosus]rge|BP

MPHVLPPLATRATPAPLPRLPGFDHTLAWLADPYRFIARGCATLGADVFQIRLLMRPTIC
MSGPLAAELFYDESRFVRAGAAPEPMQATLFGHGGVQTLDGDAHRVRKALLVGLTGADSV
QTLVSIVGEEWDRAAAGWAATPQPVTLYREVQPLLARAACRWAGVPLPEADVARRTRQLV

ALFDEAAAGPIAHLQARWARSRAEAWLTALVEHARAGRTALPPGCAAQAIVAYRDLDGRP
LPAHTAAVELLNLLRPVVAVSVWISFVAHALQRFPAWRATLASPDGENLAPAFIDEIRRH
YPFFPAIVARVRTDFDWCGLHFPAGRRVLLDVFGTHHDARIWRTPQSFLPERWLRRQPGP
YEFLAQGGGDVAGGHRCPGEDLAAQLMRLALGRLLHMRVIVPPQVMALKMSRLPAVPVDG
IRLRVLDPG

>A4W93_27770|KEGG|CYP212B4|[Rhizobacter gummiphilus]rgu|BP

MAELQRDYLGFTSQLRAQYGDLSYARVAFEHAYDVFSPELVRELLVANADGLKRWERGLE
VFEQLMGQSVLVTEGEVWQRQRRMLQPGFSPRRVAGYAALMSDAAAKGLDAALPAASPAG
QVDMDRLFTGLAMDVILRTLFSSEASEESARAVGAVQVASEFAFREMFHPMTLPDWLPLP
GKAAKRRALRTLRDLVDGHIARRRVAGAAEHDDLLGMLLSAREETTGEGLSAQEVRDQCM
VTFQAGHETTATALLWWSRLIAGDAEALEGARREVDEVLQGETPSAADMARLPWLTATLK
EAMRLYPPVPALMSRRTTREIQLGEWRIPKGALVRVTPWVIQRDGRSFEHPDRFLPQRFA
APDDKAPRGAWLPFGTGPRVCIGQHFAMLEMTVIAAMLLQRYQLGLPQGAPACEPVMHVT
LRPRNGVTLNLRRRGPARTERP

>DEH84_02665|KEGG|CYP152AR1|[Aquabacterium olei]aon|BP

MRPASNPPTLPAGTGIPKLTAWDSTLAWRADPYRFLGRQAALLGSDVFEARLLLQPTLCL
TGPQAARLFYDDSLFQRQGAAPPALVSTLFGQGGVQGLDHARHRHRKALFMAITAPERLQ
RLIELVRREWLASLHDWILSPPFSLYRALQPVLARAVCQWAGVPLADDDLPRRTAQLVAL
FEDAAAGPVAHLHARWARRQAEAWLTPLIAAARTGHTRLPTDTAAHAISTHRDLEGHLLP
PRVAAVELLNVLRPTVAVSVYMTLLAHALHTHPVWKSRLAGAASGPDAMGFVQEVRRHYP
FFPAVAARVRADFDWQGLHFEAGRRALFDLYGTNHDPRSWPGPDTFSPERWARQAASAAF
VPHGGGDPHLQHRCPGEDLTARLMLLALDILLHVMRFDVPVQRLALNMRRLPALPRAGFV
IERVRRIA

>EWH46_11610|KEGG|CYP107NN1|[Sphaerotilus natans subsp. Sulfidivorans]snn|BP

MSAVVLQPGAADPHPAYAALRQAGPIHWSEDFFGGAWVLTRHADAEQVLRDPRFSAARTG
GWVRRGETQRREAGAFQRLFARALLFLDGQDHAGLRRLMMAGFDGQAMAALRPWIEQRCT
ALMDTLQAEQAAAPERPVDWIAGVARRLPAGVISRLLGLPAEDEPMLVGWSDEIAPFLGA

ADPSPEVTRRAERALVAMAGYFRSHFARSPGAAAQEPAGLLERLLLAHRQGQAGLDDSVG
LLAQATMLLFAGHETTRHLLGNAVQLLLTHPVQWQALRADPGLAPAAVREALRHQCPVQY
SGRRVVTPLELHGQRLERGDGVIVLIGAANRDPERFERPDDFDIALPRRASLAFGSGPHV
CIGAALTRLEGEIMLGQLARRWPHLALARPETPDWVPDQPLYRGLRSLPLWLQGPPT

>EWH46_13000|KEGG|CYP1098B5|[Sphaerotilus natans subsp. Sulfidivorans]snn|BP

MARFCPAYPTPRRNKASGLLMFLSARHSWLDALYERSYRMQMGEVRLPGLDLYMVNEPGL
VRRVMVEQAEDFPKSELLGQALEPLLGESIFTTSGEQWRRQRAMMDPAFAQARLQVAFPV
MAAATEAMLARLAQLAEGTEHDIEVEMTHVTADIIFRTIFSVPMEGADARRVFDAFARYQ
ALVPRLILPSIFGLRWLVWPWDAWRARRAAREIRGLLETLIRPRFEARRTGAEHPQADIL
AAFLDARDEQGRAFGFEELVDQVAMLFLAGHETSASALTWACHLLARAPDIQQRVHDEVA
TQLGARAPGTADMKGLTLTRNVFRETLRLFPPVGFMARQARTGCPMRGKQVKAGATVVIS
PWLIQRQREGWHDPDAFDPDRHARDEEGSTGHGRAGYLPFGMGPRVCLGAAFALQEATLI
LAQLVRRWRLEAVEGHEPRPVGRLTIRSANGVRLRLHRRAEAAAG

>LRM40_02785|KEGG|CYP1098B7|[Ideonella dechloratans]idc|BP

MSRFCPHYPRPAARRPSVWRMFFSKRRSWLDGLYERSYRMKMGEVHLPGVDLYMVNEPAL
VHQVMDHADAFPKHEMLGEALRPLLGDSIFTTNGEVWQRQRTLMNPSFEGARVRKVLGRM
RAAGDAMVQRLHAVPPGGVHDVEMEMTHVAADIILRTILSQSLDSEGAQRIFAAFARYQA
LAPRLMMPAFFGLRWLRPWWQIRRSRSAAREIRSLLEAYIRPRYEAARAGGPADETAEDD
ILASLLRVRDEATGEGFGFDELVDQVAMLFLAGHETSASALSWALHLLAHDPQVQERLHE
EACRVLPGLDTEGANAIKDLTLARDVFRETLRLFPPVGFLARQSAQACPMRDKTVPAGGS
VVVSPWLIQRHRELWARPDEFDPDRYSRDQPSMAEPLDEPAARESLRQAYLPFGMGPRIC
IGAAFALQEAALILALVARHVRVEPVPGETPEPVGRLTIRAENGIRLKIFPREAG

>E1O_09790|KEGG|CYP107NQ1|[Burkholderiales bacterium]bbag|BP

MFTRYDEVAAVLRDPNCSAARAGAWVNVVGPDARREFRALKAVLARTMVFADGRRHRRLR
QVVAHGFRRAALEAMLPSIRAAVEDGIAALLAAEARDGEADFMAQFARPLPVRVIAGMLG
VPFEDALGLVASSDAIARFIGEPAPSAAAARAAEDGAQELVGYFERVLRQADRDGTPPDP

AGGGLFAILVDAHAAGRLSTRELIVQGAMLLFAGHETTRNLLGNGIQALLRHPDEWRRLA
ESPARARAAVFEVLRYDSPVQYTGRRVIRDCTVAGRRLRRGDLVIAHLAAANRDPEKFPD
PDVFRVDRHGEATPLSFGWGPHVCIGAELSVLEAWTAFAQLRQRLPGLARIEEAPTRIDN
AVYRGLARLRVRCGASADGTAVIPGNACPV

>AAW51_1491|KEGG|CYP229N1|[Polyangium brachysporum]pbh|BP

MDEIRDPVQAVTAPDPYPYYAALRERHGLYHEPRLDAWVATAAAVVDAALQHPGLRVRPP
GQPVPPALQGTPAGAVYARLVRMSEGPAHAGTRRAVTQTLDPMASGQVAGRARDHAAQAL
AMTAPGDRERLRALYRVPLLTMAEGIGVPIGQRERVALLTERFVACLSPLSGAPALSEAQ
DAVHALHGLFDEGLSSWAPAPPSGGPERETWIANLIGLLSQTCDATAGLIGNTLVALGRE
PALVSRLRQGIVQVAEVVDEVARHDPPIQNTRRYAAEPLTLAGHALEAGQCVLLLLASAN
RDPAPCGTVAIDPAPDVFAPGRPPCRSFGYGAGHHRCPGDRLASAIAGGVVQALLAAQAS
LLDGLAQRLLASGAYRASVNARIPCFP

>AAW51_3718|KEGG|CYP152B9|[Polyangium brachysporum]pbh|BP

MPSESQLDSTIALLANPYSFITERCNRHGSELFTARIMLQPTICMSGPRAAQLFYDARRF
TRVSAAPEPLRATLFGDGGVQTLDGEPHKIRKGLFMAVLGPDRVAALADRCAQEWHRHAE
AWARQGGQVVLYPALHTLLTAAVCDWAGVPVPPDELPTRAEQLTALFDRAAAKGAGHLQS
RRARKHAEDWISGLIEDIRGGRLETPPDSAAARVAQHRDADGSLMPPRVAAVELLNVLRP
TVAVSVFIVQAAHALHVYPECRLPLQKAEPGYADAFVDEVRRYYPFFPAVVARVRDDFEW
EGYHFPKGRRVMLDLHGTNRDPRAWRDPDVFRPERFRDTPPGLYNFIPQGGDVAATHHRC
PGEGVAKALTKVALDFLVQRLRYRVPEQDLRLNLKRLPCIPHDGFVIEAVELLA

>NAL212_2776|KEGG|CYP1138A1|[Nitrosomonas sp.]nit|BP

MSTNYLERYDATADVEKFPLVRRWMDTEPLPFFKELRAKRPILVTPDCTLVTRFDDVREI
LTMYKVFTVKPYVPKMDNYLMAHDDDALHTREKSLMQFMLNRDDLPRVRNMIADIAKGIL
SNANGQIEVVNSFCRMVPATLVQKYFGLTGAKREDLIEWSYWNQYDTFHNQPFDLVPPEL
SQRIIDRHNETSKKLSDYITMLIAKRLLAVKIEKLTFSTIVRLQDDIVTRMLRTSFAKEL
DFDIKRLGINAGGLLIGAIETTAQAVAQVLQYLFQHSEWMEKAKAVAQKDDPTEFDGIVW

EALRFVPITPYLFRTTVSDYTAAKGTDHETILRAGTYVLPVTLSATFDERAFELPEEFIP
QRNWYNYFHFGFGDHECLGRYVGMVMIPEMVRQVFLQKNIEPKGNIDYKSGPFPESYMLS
WNTQ

>Nit79A3_0628|KEGG|CYP1138A1|[Nitrosomonas sp.]nii|BP

MNTSTSYLEQYDATADAEKFPLVRRWMDTEPLPFFKELRAKRPILVTPDCTLVTRFDDVR
EILTMYKVFTVKPYVPKMDNYLMAHDDDALHTREKSLMQFMLNRDDLPRVRNMVADIAGG
ILGQANGKIEIVNNFCRMVPATLVQKYFGLTGAKSEDLIEWSYWNQYDTFHNQPFDLLPP
ELSQRIIDRHNETSKKLGSFITMLIAKRLLAVKAEKLTFSTIIKLQDDIVTRMLRTSFAK
ELDFDIKRLGVNAGGLLIGAIETTSQAVAQVLQYLFQHAEWLVKAKAAAQKEDPSELDGI
VWEALRFVPITPYLFRTTVSDYTAAKGTDYETVLRAGTHVLPVTLSATFDERAFDSPEDF
IPQRNWYNYFHFGFGSHECLGRYVGMVMIPEMVRQVMLRKDVEPKGNIDYKSGPFPESYD
LSWTTQ

>AAW31_11620|KEGG|CYP1225A8|[Nitrosomonas communis]nco|BP

MIANPLQAFYGWQQNYGDLVSFRLAARQFYLFSHPKLVEQALIRQSDVFVKTYNPEKPTG
LALILGQGLVTSQGDLWQRQRRLMQPVFQRSNVTTLLPQMATAGNNMLARWRQLGEGAQV
NLSGEMMRLTLEVITQTMFSTSVLDKIEQIAPSLEILLRYAAKTIANPLTLPLYVPTPAN
RKFKQALEIIDNVIYGMIDQRRTAPSGQNDLLDMLLKARDDNNGEKMTDRQVRDEVITIF
SAGHETTANLLSWTLYLLVRHPGALARLREELDRLLQGKIPNAADLQQLVYTRAVLSESM
RLRPPASFLLRKVSKDTEVDDYFLKAGRLAIFSIFNLHHHADFWPQPEQFDPERFLVSQN
RRYSFIPFGTGERICIGSHFALMESQLLLCMIIQHCDLQLLDPDEVEIEMAITLRPKGGI
PACINWR

>Nmul_A2167|KEGG|CYP1225A8|[Nitrosospira multiformis]nmu|BP

MAHNKTVPQVKGEFLLGNLRQMIASPLQAFYDWQQNYGDLVSFRLAARQFYLFSHPKLVE
QALIRQSDVFVKIYNPEKPTGLALILGQGLVTSQGDLWQRQRRLMQPVFQRSNVTTLLPQ
MVTAGNNMLGRWRQMGEGAQINLSGEMMRLTLEVITQTMFSTSVLDKIERIAPSLEILLR
YAARTIANPLTLPVYVPTPANRKFKQALGIIDDVIYGIIDQRRAAPSDQNDLLDMLLKVR
DDDSGEKMTDRQVRDEVITIFSAGHETTANLLSWTLYLLVRHPGVLTRLREELDRVLQGK

IPNAEDLQQLVYTRAVLSESMRLRPPASFLLRKVSKDTEVDGYFLKAGKLAIFSIFNLHH
HADFWPQPEQFDPERFLLSQNRRYSFIPFGTGERICIGSHFALMESQLLLCMIIQHCDLQ
LLDSKEVEMEMAITLRPKGGIPVRINWR

>EBAPG3_004825|KEGG|CYP1138A4|[Nitrosospira lacus]nlc|BP

MSASHYLEQYDAAADADKSPLARRWIDTEPLPFFKELREKRPILITPKFTLVTRFDDVRE
VLSMPKVFTVKPYVPKMADYLMMHDDDALHTREKSLMQCMLNRDDLPAVREMVANVSKEI
LDTANGNVELVNNYCRMVPATLVREYFGLTGAKRSDLIEWSYWNQWDTFHNQPFDLLPPE
KSQQIIDRHDETSKALGMFIFELIARRTLQVKAEQITLSTLVRLDDDIVTRMLRTDYPKQ
LDFDIKRLGLNAGGLLIGAIETTSQAVAQIIQYLMLHPEWLEKAKAAAQKDTPAEFDGIV
WEALRFVPITPYLFRTTASDYTAAKGTGYATVLRAGTYVLPVTLSAMFDERAFESPDEFI
PQRNWYNYFHFGFGSHECLGRYVGMVMIPEMVRQVLRRQDIKANAPIDYKSGPFPEQYDL
SWT

>BSY238_2437|KEGG|CYP1246A|[Methyloversatilis sp.]metr|BP

MTAFPAGIDARPTYIPPRPQALSPIISLFRLIAQGDGDLLSLLPADAYRVPVGHLGYSRR
QILIVNDPQLYRNILTDPTDIYPKNDLMVGALEPLVGNSIFVSSGDTWRKQRRMIDPAFS
HMRLGQAFPSMAAGVDAYEDKLREHALTGEAFSLDLAMSHLTADIICRTVFSTSFDTRAS
REVFDAFTLFERSVAQVEWKRLIFDPAWQRVDQRPDVEAACHIIRERLGELVDTHLTGPI
TRWNDIAAEMVNAEDPETGYRFDRKELIDQLGVMFLAGHETTASVLTWVFFILSSQHDTV
ARMRAEVDAVVGEGDIGFEHIRRLTFTRNVFRETMRLYPPITFIPRVAAEATQIGKYRVK
RGAMIMISPWTIHRNENYWRNAHHFDPDRFSTEREHELVPGAYLPFGLGPRVCVGAAFAT
TEATLILARLVRRFDFSALDADAVRPVARLTTRPKHQIMCRVRLRERAGT

>DENOEST_2817|KEGG|CYP2733B1|[Denitratisoma oestradiolicum]doe|BP

MKDAPQNTDYLDRPDNTRLSHLPGDYGLPFLGCMIPLVNNPSALIGSYYERYGPVCRTQT

TGQKMVWALGPDYNRDITVDSQQLFSAHMGYDGMLRDFFAGGLLMKDFGEHHFHRRIMQT
AFKTESLKQHLVQINERVSRDLPTWSSRPDFHFYPTIKTLLLDIGAKVFIGLEMGNEAHR
LNQAFLDMMEGVLSIIRRDWPGLLYRRAMNGRRSLERYFLELTPQKRTSTANDMMSHFAR
ERDEDGQPYPDTVVRDHILFLLLAAHDTTTSALTMAAYYLARNPEWQERLRQEAVGTGGE
APEYNTIKGGLPLIEQTFHEILRLHPPVPQLMRRTVQDTELGGYEIPADTLVAISPSFTH
RMKEYWREPDNFDPDRFGPERLEHKAHPYLWVPFGGGAHKCIGMHFADILFKCVMHDMLR
RYRWTLPDGYPTNPAIMHFPFSKLKDNLPLRLEPLH

>DENOEST_3328|KEGG|CYP108AB1|[Denitratisoma oestradiolicum]doe|BP

MAVPILPSEIDISSPEAYLNERRLQADFSRLRNEMPIAWVDREPYRPFWSVVRNADIKAI
EKNHQIFINEPRLTLQTRKAEAASAAMFGGQRYGVRTLTDMDEPDHRKYRDISSRWFVGP
GLRKVLERVDRIADRFVDRMVKQGGQCDFATEVAMWYPLYVILSLFDLPEEDAPRLLQLT
QHLVSASDPDLQRSDLGGTDAVPEFFEYLGRLLVERRAHPGDDLASAIATAEIDGKPIDT
LEALSYYLIMVTAGHDTTSGAITGGMHALVENPAEREKLRQPTPALLRNASGEMCRWVTP
VKHFMRTATEDFSFHDATIKAGDAVALFYASGNRDDACFEDPDRFRVDRKVDGHVAFGFG
IHSCVGRQLALAEMDAFFGRLIPRLRHVEFAGDPKLIQSNLIGGYKSLPLHYEITH

>Mfla_1442|KEGG|CYP152S1|[Methylobacillus flagellates]mfa|BP

MARPPLESTYALWKKGYVFIDSTCRALGVKAFRTRLMMRPAICMQGEAAARLFYDNEKFM
RAGAMPARAKKTLVGEGGVQGMDGHAHRHRKQMFTRELMSPERIEHLVCLHRGHWKEAIQ
AWKPGSEIVLFREAQKLLCKSVCAWAGVPLTGHELDRRAREFGHMIDSGAKLGPHHWRGR
WARKSCERWLGQLIEDIRHYGEQGQAGSILQSIAWHRDEHGQRLPLQVAAVELINILRPT
VAIAQLAVFCALALHSFPACRQKLQQGDEDYLGWFVLEVRRFYPFFPFLAAVVREDFRWQ
NLEFKRGQRVLLDIYGTHRDPALWQDPAQFIPERFKDWDGNSFGLIANGGATYEYHHRCP
GEWITIALMKAITRMLVHEIEYTVPAQDLRLDLSRIPALPESGFIIQPVITPRYLEKKHA
NS

>BN1209_1156|KEGG|CYP2159A1|[Candidatus Methylopumilus turicensis]mbac|BP

MVSLTQELLYSFDELNKKLIADPYPFYDELLCHDGPIYAIDEKNPDQKTLLLTRYDDVLK

VLSLAKEISNDKNYLTPEKPRNYGDSFILNTDGVEHATLRKIFVSFFCKSRLENVRIFLN
KYLPSLIQSLEIHDEIDLNKHVAESIPLAVMGHLMGIDNMADLRNLRVWTLELMSLADEF
TNQSDFAIQAEKNQAAKSMSDFCKSLILQKSNNTSDDLVSFIVDECSKQKIDEKKILENV
IFMMIASHDTSVNMIANGLFLLLSHPKELHKLALNPELADDATEEILRFESPLQRATYRV
TKSEINLDYFKIPSDTKVMLFLGAANRDPRHFESPDEFIISKKKTPNLTFGHGIHNCIGK
ALARLEGALAFPALTPYLINVKLKSAKPTWRNNTLFRIPETLIVTKR

>ACJ67_12780|KEGG|CYP152S2|[Methylophilus sp.]meu|BP

MALAALPQDTAFDSTLSLIRKGYRFISDTCRALDTDVFETRLLFRSVICMRGAEAARIFY
DPDKFLRHGAMPTRAQKTLVGVGGVQSLDGAAHRHRKHLFISHLMNHERINALAELHRMH
WHSTLALWPIHEEMVLFEHAQNTLCRSVCAWAGVPLPPSECSRRAKEFGRMIDSGARLGW
HHWRGRMARSSSEKWLMAEIEKIRSSKQEAATSILQAFAWHRDEHGDLLPCHTAAVELIN
VLRPTVAIAQLVALSALAIHQYPHVREKLLNQHSEYLDWFILEVRRFYPFFPFLAAYARE
DFNWKGWHFKKGTLTLLDIYGNNHDGRHWEQPDEFKPERFKNWSQDEYSFIANGGATYAE
HHRCPGEWITNRLMKVALKLLVQNLRYTVPKQDLSVSLARIPTLPKSGMIIAPLSYSAS

>ZMTM_05240|KEGG|CYP2743A1|[Methyloradius palustris]mpau|BP
MNIQPHQLTPHFHSQAFQENLYTNYRQYLEFTPVFRSAENVIYLTRYEDCIKLLSSENFK
RIAPSGGSNPLNQLSNEQTPLEMMITSWMLFMDPPRHDLVRKAFSPPFLVGALKQLEPFI
REQLQSLFASMPRSGEVEILDALAFPLPVIVITEILGVPKSDIEMFRLWSLELTKALDSG
NDLEIRNGTAAALQLKAYFTDLLKRPKFLLGHGLINTILNSNEYSLTVDELLYGAIFILW
AGHETTKNLISSGFHTLSQYPEVVRDLQAHPVLIEAAIEEMLRYETPVQKISRWAHSHEQ
FGEYVVPKSTLVTALLGAANRDGAIFEQPDVFDLRRKKNRHIAFGTGIHHCLGALLARME
GRIVFGELIPQLVKLEPVSHEWRIYSAFRSLDSLRLDVKFK

>ebA473|KEGG|CYP1157D2|[Aromatoleum aromaticum]eba|BP

MPVVFDWTGGDLGSIAQCIGVLVHYLATHPAVQDYLRSGVPDAEIDAIIDEILRIDDPFV
SNRRVTTCPVTIGGAELPQGARVKLNWTSANRDEAVFGDPDAMDPERNADRNLVYGIGRH
ACPGRLLATLEMRIAMQELLAATSSIALDSQRPAERAVSPVGGWAMVPIVLA

>ebA475|KEGG|CYP1157D1|[Aromatoleum aromaticum]eba|BP

MTDHMPDDLSATGRDLRAYTDELRPRHGVVRNAQGEWVLLRHELVVQAALSDDRFSSAVS
SHLHVPNGMDSAEHTAYREAVDGFLTPEALAPFRERFRRVATGLAASLPRGVAVDAVSQV
GVRFAVRAQSAWLGWPADLEDRLVDWVAENHAATRSGDRSWTQRVAESFDDLIRSVLEPR
RGAAHEDGADDVTARLMRTEVNGRPLTDAEIVSVLRNSVMR

>ToN1_06480|KEGG|CYP226A23|[Aromatoleum petrolei]apet|BP

MSSVKDVTLESAFAGVADNYRGEDVDLHAIYREMRKNSPIIAENFMGKLGVPNIAGLDPN
RPTYTVFKYKDVMTVLRDAANYTSGFIAEGLGAFFDGLILTGMDGEAHKKARGLLQPIFM
PDVVNTWRDSKMDPIVRNEYLLPLVPKKKADLMDFGLHFPIRLIYALIGFPDDEPEKVEQ
YAAWALAILAGPQVDAEKAAIARKAAMEAAQALYDAVKEAVVAVRQRGAEGNDLISRLIR
AEYEGRKLDDHEITTFVRSLLPAAGETTTRTFGSLMVLLLERPDVLDRVRKDRSLVGKAI
DEAIRFEPVATFKVRQAAQDLELGGVRIPKGAMVQCIVSSANRDEEAFESSEEFDIDRKL
KPSFGFGFGPHMCIGQFIAKTELQVALNAILDLFPNIRLDPDEPKPRITGAQLRGPHSVP
VIWD

>ToN1_06580|KEGG|CYP226A21|[Aromatoleum petrolei]apet|BP

MSTDILNLDLETAYTAVSETYKGKGDIHAACREMRGKSPIYVGDFIKQFGVPTNAGLKQG
TRPTFALFGYKDVMNVLRDADTYTNGFIAEGLGAFFDGLIILAMDGEQHRRVRALLQPAF
MPDTVNKWRGQIDHVIRKDFLEPMVSQRKADLMDFGLYFPIREMYALMGFPTDDTEKYKK
YAAWTLAMVGGNQVDPEKIDEARRNAGVAVKLLYDAIQEVVVKRRAEGSHGDDIIGRLLR
AEYEGERLDDHQVITFVRSLLPAAGETTTRTFSSVMTLLLTTPGLVDRVRADRSLVPKLI
DEAVRYEPVATFKVRETAKEVELHGVTIPKGSFVQCMVVSANRDEEVFENPDQFDIDRKL
KPSFGFGFGPHMCIGQFVAKVELNCAINAILDLFPNLRLDPDKPAPVIEGAQLRGASHVH
VIWD

>ToN1_49970|KEGG|CYP1157D1|[Aromatoleum petrolei]apet|BP

MTDHMPDDLSAVGRDMRAYTDELRPRHGVVRNVQGEWVLLRHELVVEAALSDDRFSNAVS
SHLQIPNGMDSDEHTAYRDALDRFLTPDALAPFHERFRRVAADLVASLPRDVAVDAVSQV
GVRFAVRAQSAWLGWPADLEDRLVAWVAENHAATRSGDKAWTQKVAESFDDLILSVLEPR
RAARGDDTDDLTARLMQTEVSGRRLTDAEIVSVLRNWTGGDLGSIAQCVGVLIHYLATHP
AVQDHLRAGVPDAEIDAIIDEILRIDDPFLSNRRVTTCPVAIGGVQLPQGARVKLNWTSA
NRDEATFGNPDSMDPERNADRNLVYGIGKHACPGRWLATLEMRIALQELLAATSSIALDP
QQSTERAASPVGGWAKVAVVLA

>C0099_03585|KEGG|CYP289A29|[Aromatoleum pumilum]atw|BP

MNTARHRAAPDWDPRAPSVQRDQIAAYDGMRIRCPVAHSDYLHWSIFRHADVLRVIGDHE
TFSNRVSQHLSVPNGMDPPQHAAFRAIVERHFDATAMATFEPACRRIAHKLAAALPVPGE
VELMRDFAHPFALEVQCAFMGWPGWMRDALADWMRENQRATHSGDRAAMRSVAERFDATL
RRLFDERRAAGAAAPDDATTRLLRERVDGRALTDEEIVSIVRNWTAGELGTIAAAIGIVA
RRLAVEPTLQTTLRADRSLLAPAIDEVLRIDPPLIANRRVTTCPVRLGERDIAAGERISV
MWAGANRDEAVFDEPDRVRLDRDPADNLLYGAGIHVCPGAPLARLELHAAIEALLERCAV
LAPLADRPPHRAGYPAGGWDKVPLQLG

>azo2527|KEGG|CYP203A3|[Azoarcus olearius]azo|BP

MFSFDPYSPAVDADPFPFYKTLRDEHPCFWSKEANMWVLSRYADIVTALNDWQTFSSAKG
NLMTELPNRAGATLGTTDPPRHDRLRGLVQHAFMKRNLESLAEPIRQIARETAAPLADLK
QFDFIEDFSSKFTVRVLFAALGLPLGDEALVREKAVTMVQSDAATRQKGPEHIAAYNWMQ
DYASKVIAERRANPKDDLISHFSMAEIDGDRLDEREVLLTVTTLIMAGIESLGGFMGMFA
LNMADHADARRQCVANPDLLVDAIEESLRFNTSAQRFRRCLTRDHTLHGKTMREGDFVCL
AYGSGNRDERQFPNPDVYDIGRKPKGHLGFGGAVHACLGTTIARISVKAAMDAFHRVVPD
YVRVQEHLPWMPSSTFRSPLRLELARA

>dqs_2684|KEGG|CYP203A3|[Azoarcus olearius]aoa|BP

MFSFDPYSPAVDADPFPFYKTLRDEHPCFWSKEANMWVLSRYADIVTALNDWQTFSSAKG

NLMTELPNRAGATLGTTDPPRHDRLRGLVQHAFMKRNLESLAEPIRQIARETAAPLADLK
QFDFIEDFSSKFTVRVLFAALGLPLGDEALVREKAVTMVQSDAATRQKGPEHIAAYNWMQ
DYASKVIAERRANPKDDLISHFSMAEIDGDRLDEREVLLTVTTLIMAGIESLGGFMGMFA
LNMADHADARRQCVANPDLLVDAIEESLRFNTSAQRFRRCLTRDHTLHGQTMREGDFVCL
AYGSGNRDERQFPNPDVYDIGRKPKGHLGFGGAVHACLGTTIARISVKAAMNAFHRVVPD
YVRVQEHLPWMPSSTFRSPLRLELARA

>AzCIB_2536|KEGG|CYP226A23|[Azoarcus sp.]azi|BP

MSSVKDVTLESAFAGVADNYRGEDVDLHAIYREMRKNSPIIAENFMGKLGVPNIAGLDPN
RPTYTVFKYKDVMTVLRDAANYTSGFIAEGLGAFFDGLILTGMDGEAHKKARGLLQPIFM
PDVVNTWRDSKMDPIVRNEYLLPLVPKKKADLMDFGLHFPIRLIYALIGFPDDEPEKVEQ
YAAWALAILAGPQVDAEKAAIARKAAMEAAQALYDAVKEAVVAVRQRGAEGNDLISRLIR
AEYEGRKLDDHEITTFVRSLLPAAGETTTRTFGSLMVLLLERPDVLDRVRKDRSLVGKAI
DEAIRFEPVATFKVRQAAQDLELGGVRIPKGAMVQCIVSSANRDEEAFENSEEFDIDRKL
KPSFGFGFGPHMCIGQFIAKTELQVALNAILDLFPNIRLDPDQPKPRITGAQLRGPHSVP
VIWD

>CEW83_02400|KEGG|CYP226A22|[Azoarcus communis]acom|BP
MIIFYGPVPCEHNKETELSSDLNDLDLKSAYTAVSDTYKGTGDIHAACREMRDKSPIFVG
DFIKQFGVPTNAGLQQGTRPTFALFRYHDVMNVLRDATTYTNSFILEGLGAAFSDGLFIL
GMDGEAHRRVRALLQPAFMPDTVNKWRDTMETLIRKDFLEPLVPQKKADLMEFGLYFPIR
IMYALMGFPTQDTDKYKKYAAWTLAMVGGNQIDPTKVEEARRNAGTAAKMLYDSIMEVVV
ARRAAGSQGDDLIGRLLRAEYEGEGLDDHQVVTFVRSLLPAAGETTTRTLGSVLTLLFNT
PGTLERIKADRSLVPKLIDETIRYEPLATFKVREVAKDVEFYGVKIPKGSFVQCLVVSAN
HDGEIFENPDVFDIDRKQKPSFGFGFGPHMCIGQFVAKVEIACAINSILDLFPNIRLDPD
MPPPVIEGAQLRGASHVHVIWD

>CEW83_02430|KEGG|CYP226A24|[Azoarcus communis]acom|BP

MNNAKNVTLEDAFAGVADNYRGEDVDLNAIYREMRKNSPIIAENFMGKLGVPNIAGLDAN
RPTYTVFKYKDVMNVLRDATHYTSGFIAEGLGAFFDGLILTGMDGEAHKKARGLLQPIFM
PDVVNTWRDSKMDPIVRNEYLLPMVSEKKADLMEFALYFPIRLIYALIGFPDDKPEKVKQ

YAAWALAILAGPQVDAERAALARKAAMEAAKALYDAVMEAVVEVRRNGAEGNDLISRLIR
AEYEGRKLDDHEITTFVRSLLPAAGETTTRTFGSLMVLLLERPELLERIRNDRSLVGKAI
DEAIRFEPVATFKVRQAAEDIELAGMPIPKGAMVQCIVSSANRDEEVFENSETFDIDRKL
KPSFGFGFGPHMCIGQFIAKTELQVALNAVLDLLPNIRLDPDFPAPRITGAQLRGPHSVR
VIWD

>CDA09_09135|KEGG|CYP108Z1|[Azoarcus sp.]azd|BP

MQAKIDDLICTPATYADSALYHRIFNSLRRDDPVHWVEPQDYRPFWLITKLEDIREVERQ
NDIFLNAGRPTLRSRTEEEKIRAVTGGKPDVFETILHMDGMDHRAHRAVAQSWFMPANLK
KLEGAIAQVAREFVDRLERQGGTADFCEVVAARYPLRIIMTIFGVPPEDEDQMLYYSKMI
TAPTDDDVETTEPRNELRIRAAQGLFDYFRKLLADRRANPRDDLSSVIANAEVDGKPISE
LAALSYCVTIATAGHDTTSMTSSGGLLALVQHPEQMARLRADRSLMPGAIDEILRFVSPV
KHMMREAKVDAIVRGKQIRAGDSLMLCFPSGNRDEDVFDNPHEFRIDRSPNRQIAFGSGV
HSCLGLHLAKMEIRALYAELLDRVDRFELDGTPGFYKGFVVQGLKHLPIRYSMSKVTA

>CDA09_13845|KEGG|CYP226A23|[Azoarcus sp.]azd|BP

MSSVKDVTLESAFAGVADNYRGEDVDLHAIYREMRKNSPIIAENFMGKLGVPNIAGLDPN
RPTYTVFKYKDVMTVLRDAANYTSGFIAEGLGAFFDGLILTGMDGEAHKKARGLLQPIFM
PDVVNTWRDSKMDPIVRNEYLLPLVPKKKADLMDFGLHFPIRLIYALIGFPDDEPEKVEQ
YAAWALAILAGPQVDAEKAAIARKAAMEAAQALYDAVKEAVVAVRQRGAEGNDLISRLIR
AEYEGRKLDDHEITTFVRSLLPAAGETTTRTFGSLMVLLLERPDVLDRVRKDRSLVGKAI
DEAIRFEPVATFKVRQAAQDLELGGVRIPRGAMVQCIVSSANRDEEAFENSEEFDIDRKL
KPSFGFGFGPHMCIGQFIAKTELQVALNAILDLFPNIRLDPDQPKPRITGAQLRGPHSVP
VIWD

>CDA09_13895|KEGG|CYP226A21|[Azoarcus sp.]azd|BP

MSTDILNLDLETAYTAVSETYKGKGDIHAACREMRGKSPIYVGDFIKQFGVPTNAGLKQG
TRPTFALFGYKDVMNVLRDADTYTNGFIAEGLGAFFDGLIIILAMDGEQHRRVRALLQPAF
MPDTVNKWRGQIDHVIRKDFLEPMVPQKKADLMDFGLYFPIREMYALMGFPTDDTERYKK
YAAWTLAMVGGNQVDPEKIDEARRNAGIAVKLLYDAIQEVVVKRRAEGSHGDDIIGRLLR

AEYEGERLDDHQVITFVRSLLPAAGETTTRTFSSVMTLLLTTPGLVDRVRADRSLVPKLI
DETVRYEPVATFKVRETAKEVELHGVTIPKGSFVQCMVVSANRDEEVFENPDQFDIDRKL
KPSFGFGFGPHMCIGQFVAKVELNCAINAILDLFPNLRLDPDKPAPVIEGAQLRGASHVH
VIWD

>Tmz1t_0929|KEGG|CYP1157D1|[Thauera sp.]tmz|BP

MTDHMPDDVSAAGRDLRAYTDELRPRHGVVRNVHGEWVLLRHELVVQAALSDDRFSSAVS
SQLHVPNGMDSTEHTAYREALDRFLTPEALTPFRERFRRVAADLVASLPRGVAIDAVSQV
GVRFAVRAQSAWLGWPADLEDRLVDWVAENHAATRSGDRSWTRQVAESFDDLIRSVLEPR
RGAREDDTDDVTVRLMRTEVNGRPLTDAEIVSVLRNWTGGDLGSIAQCIGVLVHYLATHP
AVQDQLRSGVPDAEIDAMIDEILRIDDPFVSNRRVTTCPVTIGGVELPQGARVKLNWTSA
NRDETVFGDPDALDVERNADRNLVYGIGRHACPGRSLATLEMRIAVQELLAATSSIALEP
QQPAERAVSPVGGWAKVAVVLA

>Tchl_0232|KEGG|CYP116B253|[Thauera chlorobenzoica]tcl|BP

MSGLAAAFDPFEGPYQVDPAEALRWSRDQLPVFYSPKLGYWIVSRYDDIKSVFRDNVLYS
PRNALEKITPATPEAMKVLASYGYAMNRTLVNEDEPAHMERRRVLLDHFMPENLEAKQEM
VRRLTREKIDGFIDRGRVDLVEEMLYEVPLTIALHFLGVPEDEIERMRSFAVAHAVNTWG
RPTPEQQIEVAHAVGRFWQYAGEVIEKMRADPDGEGWMHYTIRKNAEIPEIVTDSYVHSM
MMAIIVAAHETTSLASTNMFKTLLTHRDAWEDVCADPSLIPNAVEECLRYAGSMVAWRRE
TTAPAQLGGVELPAGAKLLIVQASGNQDERHFEDGDKFDIYRDNAVEHLTFGYGSHQCLG
KNIGRLEMRIFLEEMSRRLPHLRLAEQEFTYLPNTSFRGPNNLWVEWNPEQNPERANPAL
RRGGVHFPVGAPLRKEIARKVRVTAVRREADGVLGLTLEDVRGRPLPRWSAGAHGELCLG
EYERKYSLCGRPDAPGYDVAILLEEQGRGGSRYLHEVVRPGTELKLRGPNNLFRLDESAR
GYVLVAGGIGITPILAMADRLKMLGRDYVIHYAGRSRASMAFLARLESDHGGHVRIYSKQ
EGERVDLPAIVGSLAEGAQIYACGPARMIRALEDLSAELPGNTLHFEHFSAQGSGLDPEQ
ESAFEVELMDSGLTVAVAADTTLLEAIRAVGIDLASDCCEGLCGSCEVTVLEGEIDHRDL
VLTRSERAENRRMMACCSRAKSGGRIKLAL

>CCZ27_08860|KEGG|CYP226A23|[Thauera sp.]thk|BP

MSSVKDVTLESAFAGVADNYRGEDVDLHAIYREMRKNSPIIAENFMGKLGVPNIAGLDPN
RPTYTVFKYKDVMTVLRDAANYTSGFIAEGLGAFFDGLILTGMDGEAHKKARGLLQPIFM
PDVVNTWRDSKMDPIVRNEYLLPLVPKKKADLMDFGLHFPIRLIYALIGFPDDEPEKVEQ
YAAWALAILAGPQVDAEKAAIARKAAMEAAQALYDAVKEAVVAVRQRGAEGNDLISRLIR
AEYEGRKLDDHEITTFVRSLLPAAGETTTRTFGSLMVLLLERPDVLDRVRKDRSLVGKAI
DEAIRFEPVATFKVRQAAQDLELGGVRIPKGAMVQCIVSSANRDEEAFESSEEFDIDRKL
KPSFGFGFGPHMCIGQFIAKTELQVALNAILDLFPNIRLDPDEPKPRITGAQLRGPHSVP
VIWD

>CCZ27_08910|KEGG|CYP226A21|[Thauera sp.]thk|BP

MSTDILNLDLETAYTAVSETYKGKGDIHAACRAMRGKSPIYVGDFIKQFGVPTNAGLKQG
TRPTFALFGYKDVMNVLRDADTYTNGFIAEGLGAFFDGLIILAMDGEQHRRVRALLQPAF
MPDTVNKWRGQIDHVIRKDFLEPMVSQRKADLMDFGLYFPIREMYALMGFPTDDTEKYKK
YAAWTLAMVGGNQVDPEKIDEARRNAGVAVKLLYDAIQEVVVKRRAEGSHGDDIIGRLLR
AEYEGERLDDHQVITFVRSLLPAAGETTTRTFSSVMTLLLTTPGLVDRVRADRSLVPKLI
DEAVRYEPVATFKVRETAKEVELHGVTIPKGSFVQCMVVSANRDEEVFENPDQFDIDRKL
KPSFGFGFGPHMCIGQFVAKVELNCAINAILDLFPNLRLDPDKPAPVIEGAQLRGASHVH
VIWD

>Tharo_0243|KEGG|CYP116B253|[Thauera aromatica]tak|BP

MTIENSTQAQPTRGGCPVRHSEGACPASALAAAFDPFEGPYQVDPAEALRWSRDQLPVFY
SPKLGYWIVSRYDDIKSVFRDNVLYSPRNALEKITPATPEAMKVLASYGYAMNRTLVNED
EPAHMERRRVLLDHFLPENLEAKQEMVRRLTREKIDGFIDRGRVDLVEEMLYEVPLTIAL
HFLGVPEDEIERMRSFAVAHAVNTWGRPTPEQQVEVAHAVGRFWQYAGEVIEKMRADPDG
EGWMHYTIRKNAEIPEIVTDSYVHSMMMAIIVAAHETTSLASTNMFKTLLTHRDAWEDVC
ADPSLIPNAVEECLRYAGSMVAWRRETTAAAQLGGVELPAGAKLLIVQASGNQDERHFED
GDRFDIYRDNAVEHLTFGYGSHQCLGKNIGRLEMRIFLEEMSRRLPHLRLAEQEFTYLPN
TSFRGPNNLWVEWDPEHNPERANPALRRGSVHFPVGAPLRKEIARKVRVSAVRQEAGGVL
GLTLEDVRGRPLPRWSAGAHGELCLGEYERKYSLCGRPDAPGYDVAILLEEQGRGGSRHI

HEVVRPGTELKLRGPNNLFRLDESARSYVLIAGGIGITPILAMADRLKMLGRDYVIHYAG
RSRASMAFLARLESDHGGRVRIYSKQEGERADLPAIVDSLAEGAQIYACGPARMIRALED
LCAELPGNTLHFEHFSAQGSGLDPEKESAFEVELMDSGLTVAVAADTTLLEAIRAVGIDL
ASDCCEGLCGSCEVSVLEGEIDHRDLVLTRAERAENRRMMACCSRAKSGGRIKLAL

>AIO55061.1|NCBI|CYP1013A3|[Burkholderia mallei 23344]bmal|BP
MSKHRQAPGPRGGLVMGNLAAYKRNPITMLLRLHQQYGDVARNRLGPFVTHALAHPDHIQ
YVLQENHRNYVRGRFYDNFKMFFGDGLLTTDGEFWRRHRRVVQPLFHKKQVDAHTAAVGD
AALALAYRWSALPPGKALDVVEEMMHLSLRMLGLMVFNTDVSSHAEAAGPAVRFGIEAMM
PQGNLNDFIPRWAPTRFNRRIAHARRAIDTIVAKIIADHREARCEPSDVISLLLNARDPD
TGAPMTQQEVHDEVMTVFLAGHETTGAGLAWALYALAQHPAVLRQLRDELDARLGGRAPT
VQDFEQLPYLSQVVDEVLRVYPPIWGFTRDLVEDDEIGGYRIPARSSVFMSPYVTHRHPA
FWRNPDAFDPENFASDAPARHRFVYFPFGGGMRKCIGFQTALLQMRVLVAVVAQHFDLNA
LPGHPIELGATISLRPVHGIRLIVKPRERQQSHLARVREHESARALRPLGDTATTAGAAC
PMTAAREPGASAAALAAVEAGALDAAHRGGAAAAAAGEPCPPRDPAEAAPLPLAAAPAPA
SPAVDADASARGVPAWRFTWRPVPVDPVPDEPSPALSGRRIALVNGRGGTAERVAAALAR
TCAKVSVFAPAASADHAEAARVFVNEAGPLDGIVDLGLEAPFALDDAGAWEAPMRRTVEL
LKACYGDWAAEDDTSRLFYVALTWMDGQMGYGETAGAQPLGGLWAGLAKTLPQELPNCNV
RVLDIAPDETGRVDRLVVNELYRWGLFEVGYRGGVRYTLHAKRNELPPHAVDARTTLGAG
DTVLFSGGARGIGLLCARSLAERHGCTVIVTGRETPPDGGEPWMALDEPGFKHYGLEQLR
AATPEHPPKAIRAALQRLKRRRELRTALDEMAALGLPVHYRVCDVTDRAAVRALCDELGD
SLRMVIHNAGVDRPVRLIQKRAEDFADTVRTKVLGFANLCDAVRARPRLVQFCNVGSLTG
RWGGMTGETDYAAANEALARLGLWAARHAFSPACGVKTLVWPTWEGVGMITNFAVTQRYV
SPMNVDEGVRHWLRELAAPGSGEVMFMGAVGRAPTPMQIKGFVPIRELPNIGELVTRRHH
VGEPVRFRPFKRFESRYAIDPAVAPYAHAFRFNGRAVLPASLVLEHACAVGDWVMPPTGE
PRELSEMTNVVLRLDALPDVPAGGAPVELASEAAGYMIGDVWCVDVRCSDARTQAELLSM
TLVYRPQPEQIGAAFARAPDTTPEPAPLTSAPHAAWNGELLPAGDWSVPDDASAGIVRIG
RVPSAAVLSLWALPFPLELRLPVNHVEHVLRVLLAEWGAARADAPAVWRIGGAKLARLPA
ASADGVVEYAAGRFAVLDARGYVLLRIDDARVDGEPAQAALPETGALADPLTA

Deltaproteobacteria

>Desaf_1336|KEGG|CYP152E2|[Desulfocurvibacter africanus subsp. Africanus]|daf|DP

MSTIPRDKLPESTLALFRDGYEFIGKRCKRLGSDVFQTRLMLQKTVCMRGPEAAQVFYDQ
RYFQRKGGAPLRLRKTLFGQGGVQGMDGEAHRHRKAMFMSLMSSESIARLVRLMSDEWRA
ALPGWAEKGRVVLLDAARDILCRAVCAWTGVPLKESEVRKRTQDFAAMIEDSGAIGPRHW
KGRLSRKRAEGWIGGVIEAVRAGRLNPPEGSALRTVAFHRELDGELLKTRIAAVELINVL
RPTVAVARFITFAALALYEYPDWRRRLARQKDRELELFVQEVRRFYPFFPFVAARVSKPF
DWQGYHFPEGARALLDLYGTDHDPRAWDDPETFRPERFLTWDGSPFNFISQGGGDHYENH
RCPGEWITIELMKEAVRLLVMSMDYIVPQQDLTVSMTRMPTMPQSGFVIADVRPREVIV

>MXAN_0683|KEGG|CYP197F1|[Myxococcus xanthus]|mxa|DP

MVRSTCASGSSPLQDPFMSQVRLPPGPQGHFIAGNLVEFSEDPLGFLTRCAREYGDVVRL
GKRNFLLNHPDLIERVLVNGDGNFVKLAGVGQGKRHKGGFPEAMMNSEGEDWLRKRRLVQ
PAFHRKHVAACGDTVVALTETMLQTWRPGDARDVHADVSALALDIVSRFLFHTPIDDEAR
HVADAVDAVMRHTDSPLRPPIWVPTPTNLRLRRALGRLNTLLATLVRRYREQPESRTDLL
ALLLSAPVPLSENQLRDELATMIMSGHETTADALVWAWYLLAQHPEAEARLVAELETVLG
GRLPGAEDLPRLRYTEAVVKEAMRLYSPAWITSREALRDCELGGFHVPAGTMLAVSQWVT
HRDARYFDAPESFRPDRWLSEDAQRMHRYVYFPFGGGPRFCIGSALAMMETVLITACVAR
RFRLELAPGCVVRPRPALALQPLGVWLIPRHRSHTTQEGEVRHAAGA

>MXAN_1743|KEGG|CYP1506A3|[Myxococcus xanthus]|mxa|DP

MPNLLSRGLFSLFFGGRRKPLASLPGPAPGILGNIGDFLGAQPWDVCARYARTYGPVAVA
WLGPSPALVLNDPTLIHEVLETRRLEFEKGNIGDQIRHSVTDDTPFIAQQGEDWARKHAM

DPLAQPWTPAWQAAQVEPLRAAILESVEALLQQPSIDLTPTLRKLTFDAFCVATVGEKLP
TSVYDDFMLLAAAADARIQAKLPLKFVSPPKGFADAKERFYGLFLDKVRAARKAPAPHRV
DLMSWTLREMPQLDDVVHANLLGGFFFGGVFSSSTTLVGAFHQLNKYPATEERLALESAA
LGQGPLTLERLKDAPWGEAVAYEALRILPAVRIFLRRTPPTPTRLAEVTLPPGTTLMISN
QHLHRDPSHWTAPETFAPERWLDGGAARNPHGSDHFFPFGRGPRACVAGDFAIVYLRTAL
ATVSARARIHVDSTEPFEEGFFFGVVRPQGVTGKLVPRQVQTLHPPTGAPPPPAVESA

>MXAN_2304|KEGG|CYP253H2|[Myxococcus xanthus]|mxa|DP

MSIHPAGASTAAPLPPLSPGAPLIGHLRALRKDPLHFLQAQAREYGDVVRLPMGPADLVL
VAHPDGVRHVLQDHARNYSKQSRGFRVLQELLGHGLLTSDGDHWLRQRRLAQPAFHRQRV
AGFTRTMVDAAADLAATMEARADTGAAFNVAEDFTRLTLRIASSTLFGADVSSATHDIAT
VMSRLQVFVYKRLTQPVPLSLRLPLPAHRQFERDVGSLNRVVHGIIAKRRRESGEHHDLL
QMMMEAHDDDTGERMSDSQLRDEVLTLLLAGHETTASALAWTIMLLSQHPGVRRDMESEL
ARELGGRNPTHEDLPRLELTHRVVDESLRLYPPAWALSRIATKEDLVGGFRIPKGAHLLI
APWVTHRHPSIWDNPEGFDPDRFLPEREQARPRFAWFPFGGGPRQCIGNQFALMELVLVL
ATLLQRVRLNLTPGQVIHPTPAITLRPRPGVWVTASRP

>MXAN_3943|KEGG|CYP209A1|[Myxococcus xanthus]|mxa|DP

MCGRPPGQGLEFMGTSEPVEPDHALSKPPPVAPVGAQALPRGPAMPGIAQLMMLFLRPTE
FLDRCAARYGDTFTLKIPGTPPFIQTSDPALIEVIFKGDPDLFLGGKANNGLKPVVGENS
LLVLDGKRHRRDRKLIMPTFLGERMHAYGSVIRDIVNAALDRWPVGKPFAVHEETQQIML
EVILRVIFGLEDARTIAQFRHHVHQVLKLALFLFPNGEGKPAAEGFARAVGKAFPSLDVF
ASLKAIDDIIYQEIQDRRSQDISGRQDVLSLMMQSHYDDGSVMTPQELRDELMTLLMAGH
ETSATIAAWCVYHLCRHPDAMGKLREEIAAHTVDGVLPLAKINELKFLDAVVKETMRITP
VFSLVARVLKEPQTIGGTTYPANVVLSPNIYGTHHRADLWGDPKVFRPERFLEERVNPFH
YFPFGGGIRKCIGTSFAYYEMKIFVSETVRRMRFDTRPGYHAKVVRRSNTLAPSQGVPII
VESRLPS

>MXAN_4127|KEGG|CYP147A2|[Myxococcus xanthus]|mxa|DP

MAQASIFEQILDPANRANPYPLYAELRKTPVAREADGTYIVSTYDEIVALLHDPRVSSDI
RNLVRQAGATPSPQEGPPGLPEPFIRRDPPDHDRLRKLAMRPFGPPHTPGRIDALRPWLV
ETTTGLLDALAGKNQVDIVGDVAYPFPVTVICKLLGVPREDEARFHELADAGVETLDPTT
GTIEQRKAKRDRTKAELGQYLAALADAHLRQPGGDLLSGFLTDNGPDGRMSREEVLSTAA
LLLVAGHETTVNLIANGMLTLLRHPGVFERLRREPELSIPLVEELLRYEPPVQFLPDRVT
LADIDIAGTTIPQGSPLVLMLAAGSRDPERFDDPDRFMPDRPNNSHLGFGSGIHYCFGAP

LARLEAQIALTELACRLEHPRLVADPPPYRPNPALRGPLHLLVEVDGIRAAGVR

>MXAN_4919|KEGG|CYP1069C1|[Myxococcus xanthus]|mxa|DP

MSKTNAPSGLGSEYHPLDSPQLENPHPFLARARSEEPVFFSPALGAWVVTRHADISAVVA
DTTRFSSAESITVGAATTPPEVVTALMDGYPLVPSLVDNDPPAHGRFRSLVSKAFTGRRL
AEKEPFIRTLVDALIHSFRHERQADLFLRFAHPLSCSIIAEILGIPSSDIHRFRRWSDEL
STVLAAHGPIEHQVACAQGVVEFQRYLASTLKEREVSPREDLLSDIVTGAQGMTPPMSMA
ELVSLLMQVHFAGHETTAGLIVGAVELLLEHPEQLRALRDDPGLIAGAVEEAVRMISPVH
AMFRTALEAVEIGGVPIPKGAHIRIVYASANRDEARFHEPERFDIRRPEVKKHLAFGQGL
HFCIGAPLARLEARLAVEALLRSLPGLRLVPGQKPGFLRSVTVRRHESLEVAWDVQANQA
L

>MXAN_7298|KEGG|CYP1069A3|[Myxococcus xanthus]|mxa|DP

MPVSVQKQEETGVPAHASAGRCPHLGAQYNPFTGPHAEDPHAFFEKLRKDEPVSFSPMLN
MWLVSRYEDISQVLKSPAQYSNRDMLASGTHLTDEAKAILDQGFPTAHVLLGMDPPEHTR
LRRLMNRGFTAQRIAGMGPFIREMATTLVDRFAQDGEADLVKQLAWPLPVHVILGVMGVP
QEDVWKIKRWSSDWQQLVFEYVAPELQVEMAKGVIEFQQYCIRLIEDRKKNPQEDLTSYL
VAVESDGEALTMHELVMAVGASMLSAGHESTTALMANAWKLALQHGLWQRLRDNRDLVPK
FLEESSRYDSVSHAMIRTAKEDVELGGVKIPQGSRLLLLFAAGSRDESLCPHSSKLDVDR
EKVPQHLTYGRGTHFCLGAPLARLQFEITTNILLDRLPDPKLVPGQDFGTWQSLVLRQMK
HLKVEWTPT

>MYSTI_02578|KEGG|CYP1506A2|[Myxococcus stipitatus]|msd|DP

MPNLLSRGLFNLFFGSRRKPLASLPGPPPGILGNLGDFLGAPPWDVCAHYARTHGPVAVA
WLGPSPALVLNDPAVIHEVMETRRLEFEKGNIGTQIRHSVTDDTPFIADQGADWARKHAM
DPLAQPWTPAWQAAQVEPMRAALEESVEALLREPSLDLALALRKLTFDVFCVATVGEKLP
TSVYDDFILLAAAADARIQAKLPLRFVSPPKGFAAAKERFYGLFADKIRAARQAPSAHRV
DLMSWTLREMPDFDDRVHAHLLGGFFFGGVFSSSTTLTGAVHQLQKYPAVEERLAKESAA
LMRVPLDFERLKGGSWGEAVAFEALRILPAVRVFLRRSPLAPTQLVGVTLPPGMTLMISN
QHLHRDPSHWVQPEVFDPERWLNGGAARNPLGSGHFFPFGRGPRACVAGDFAMVYLRTAL
ATLFARVRIQVDSTEPFEEGFFFGVVLPKGVTGKFVARQRATDSTPMGAPPSSAVESA

>MYSTI_03599|KEGG|CYP253L1|[Myxococcus stipitatus]|msd|DP

MDSTQVPSHPPSPWGLPLWGHLLRHDRDPLGFAVESFRKHGDVVRLKLGGETTYLVSHPD
HVRYFLTENANNYSKPPLVNDPFLGNGLFVSEGTYWRRQRRLVQPSFHRERLNGLLSGMA

ALIDGMLKSWEERTRAAQPLDIAEEMGILSLHMTTRALYSEVPDAPTTAAVRRMMHTFNA
RESLLARVLRLERLPLYRRKWADFFDTQRLMRRKAKEVVERRREGGPEDDIMAMLVAARD
RDTGEAMTEKELRDEFMNLFSGNEGPGAALAWGWHLLSLHPEIADRLASESAAVLGGRAP
TLEDLPRLRYATQVFEESLRLYPTAWKLVRVAGAADTLGTYPVAPGTVFMAISYIIHRHP
EFWSSPEVFDPDRFAPGGPARHKFAYLPFGGGQHICIANNLSLMFGALVLAMVSQRFRLR
GIPGRRVDIHPGISLLPRGGLPMTLEPRP

>MYSTI_04048|KEGG|CYP1501A1|[Myxococcus stipitatus]|msd|DP

MDTSVSSSSGRRLAGPRGLPVLGSFFPMLSEPLALANKLHARYGDVSLIRVYKTPIAFLR
NPVDVKRVLIDEPQTFPKPLIDNPFNGNGLTVSRGDHWKRQRHMVQPLFHKEKVRLWSGI
FSEELAKSVERWKEMGARGESFDMYTEGARLMFGVMWRTCFEEQPEIAYFDRIMDAIDVF
GRRVSPLSVLLYNLMPRMDPRHERVYTSVKLINGWIYERIGKRRGRGTEDGDITLLSMLV
EAKARDSGESMNDKEVRDEIVNLFGASFEMIATSLTWAIHACTQHPEVTQAIRDEVQGVT
GGAPPRYEDVAKMPYTNRVVQEINRLCPPAWTILRETASEVEVGGVMLPKGTQLLLCPYA
IHRHPDHWKDAETFDPDRFLPERLTGMHKGAYVPFGAGSRICVGQHMSLVDTVQTLAAVL
QQLDVEYLGKTPVEWQTRLTFVPKHGMPVRMRARQG

>MYSTI_04343|KEGG|CYP1491A1|[Myxococcus stipitatus]|msd|DP

MQGTHAETPLPPGSRLHWLLGNGPRQVKDALGQLVRGHKRHGDIVYFRFFEGPTFLLCHP
EHAQRVMVDNARNYRFPGPENGFDGPLMGRGLFASRGDYWLRQRRMVQPAFHRPRLGGMV
SGLVEGTLAMVERWEPHLHSGQPLELLEEMRHLSISLLGRTIFSRDVYAESAQLREALDF
FTRDSHGPRDSVVKVVRQLLRLNPGHHERFMGAITKMNTVLYALIAERRAAPSLGDDVLG
MLMSARDAQGEAMTDRQVRDELVSLFVGGQEATAVALTWTWYALARNPEVEQRIRKELAD
VLGGELPTSATLPELHYTRATVEETLRLYPPSWQFARRAREADEVGGFKVPPGMLMLISP
YILHRHPSAWEEPERFLPERFMGEQRERRSQRLAYMPFGAGQRLCIGNHFTPVLMTAVLA
VTLRRFQHRLVPDFPVVTMSGSTFRPRNGLMATLHPASPLP

>MYSTI_04792|KEGG|CYP1497A1|[Myxococcus stipitatus]|msd|DP

MTRHELPPGLSASAAAQGFRFFQNPLSFLDQCVEQFGDLFTIQFPGSRKQVCLSNPEDLK
EMYTGSPDLLHAGEAAGSVFSPLTGWNCTLTLDGEAHARRRELVQAPFRGGQVERHAGAM
YAIATAAIERWPRTGRFPLQPQMQDIALRIIFRTVLGLDDTAPGHARFLELISQVARLGM
GSKMLLIPALRWDLGPLSPWGRIVRIVRDTDALLFAEIARRRTDPKLATSEDVLSLLIRG
EGQDGLRLTDQEIRDETVALLIAGLETTAVALTWVAERLMALPEVRERLRAELEPLHTNG
ELDVTRLSALPYLDAVLKESLRNRSLSPICGGRVLKAPMRLREYELPADTVLINSPYLLH
RRRELYSEPDAFRPERFLGSPPSAHKWTTFGGGNRRCLGQKFALLELKVVTSAMVLQTGL

ELATPRVVPKADGIHLVPAAGLPVRRGTCPFAKKAAAAS

>MYSTI_04794|KEGG|CYP120F1|[Myxococcus stipitatus]|msd|DP

MHQPPLPPHASGVPWVGDSVEFGRNPQRFVLKRQARFGPVFMGHILGASTAVMVGPQALR
FVLSSHRHHFVSGPGWPRGLGMLMSGALMMKDGDIQTRTRRLLAPALAGSALASYTPVME
ATARRHLERWVQQGRLSLYDGLKGLTFDTASQLLFGTPEGADTGRLGKLFATYTAGMQGL
HPVVPLRVPFTPFGRAFAARSEMLKEVTRIIQEREKSTGSDALARLLESRKEQGEGPSAD
ALAEQAFFLLFAGHESTSSLTTWACLELSRHPDLLDSAREEAQSLGEGPLTMERVERLPF
LEQVLLETERLHPPFSGSFRQVVKSFEFNGFHVPEGCRVFYSINGTHGDPATFPEPERFT
PGRFTPEAARCARHELGLVGFGAGPRSCLGMGFARLQAKVVLALLLRDYEWSLQPKQSLD
PVYLPSLFPKDHLRVSFRRRSAQETRHRETA

>MYSTI_04805|KEGG|CYP1503A1|[Myxococcus stipitatus]|msd|DP

MSQTPEISIFSPSFLANPFPIYQQLREQAPVFWSRELGAWLISRYADVKMLLTDERATPD
QKAWAGYPELKTSAQPTEHVQWALDATLLLCHGAQHARMRRFANTPFQSAGCGRIARVTE
QVVHEVLDGFSGRKEIDLLADFSAPIRIKVMGRLFGAGFTPEEEEFLVTGTNLALGFLEP
SLAPEAITRNEGALRGFRALVENVIRRGKEAGAEDSILGDLLQPNREGDRLSDEEVLSLV
FSFLLAGTEANGSLITMGVLLLLEHSGQLAHLQREPDRYPDAIREILRYRSFTKFLPRYL
KEDISLHGNVMKQGQIALLLFQSAFRDPETFENPDVFDVMRKRSHELLAFGAGPHRCVGE
RMAEIEAVIALREFFKRFPKATLRGSEADWVKHHMLVAVPRSVPVVPVLLS

>MYSTI_04813|KEGG|CYP1495A1|[Myxococcus stipitatus]|msd|DP

MEPDFNPAHPAFRADPHRWLGWLRERDPVHFSPRLNAWVLTRYDDVRRAATDVTHFSNDT
LSVLVGKHAGAMGPRRTEHRIGTNLGMADGAVHTRLRTAIAPYFTPGAIHRLETGLQHYV
DGLLRRAASGGELDLVAGLGRPLAVEAVASGLFGIPEGDREPLSGWAAATTRISDPLLTR
EERRENFLELTRFADYLDGLVVQRRQQPGDDLLSRIADLDRSGLTHPEVLATCMSIVGGG
IDTVSMGISRGILALLDHPEQMELLRENPDLLPGATEEILRFCAPAVFVARVVRVDLELR
EKTLAAGDVVLYSPAAACRDPAVFSEPDRLDLRREVERGHNLAFGHGAHYCIGAALARLE
LRVAFRGVLSRLPRLELKVPRSMLTYGRNLMSMGIESLPVVFPPQNIPLP

>MYSTI_04828|KEGG|CYP125N1|[Myxococcus stipitatus]|msd|DP

MHKTELADLASPITYGRAIPHETFEHLRREAPVYFHQEPDGGTGFWAITRHEDIITISRD
PATYSSYRGGTSIEDYSQEDLSLIRFMMLNMDPPQHVKYRRLVSSGFTPVAITYLEPRIR

AVTKEILDKVVHEREFDFVTSIAAELPLQVIAELVGIPREERHQLFAWSNRLIDYDDSGR
ARSFDDAKMAAMEMWQYANQLAARNQGREGKDLVSVLMNAEVDGEKLNEAEFDAFFLLLI
VAGNETTRNLISGGMLALMEHPEELARLRANPALLPTAVEEMLRWVSPVVCFRRTVTRDT
VLRGQQLREGDKVVLFYPSANRDESVFENPGRFDISRFPNEHIAFGIGQHYCLGTSLARL
EIRVMFEELLKRLDGLELAGPVERRRSKLVNAIRAMPVRRPPSSRPREGARENQEGTACE
PSMRG

>MYSTI_06075|KEGG|CYP1352B1|[Myxococcus stipitatus]|msd|DP

MSKSAPPTFISRFEPHNVENPYPLYARMREEAPVHFSPEMHLWVVSRHDDVKAVVLNPQD
FLSANSFRNPVPPAPEVVAVLEQGYPQVPALVDDDPPNHTRMRVIVTKALAPHRLSAMEA
RVRSITTELLDAFAADGHADLVDKLGYPLPARVIGAIMGLPDSDLERMKRWTEDLALLSA
GNVPVARQVECAHGLVSIQKYLAGYIAERRKTPGDDLISALIEARYEDSPPLSDVELISL
LSLLHFAGHETTTNLLGNLLVWVLQEPERLKELREDPTRIPRAIEETLRLDSPIQGMMRT
TARAVTLNGVEIPANARVLVLFASANRDETIFHAPDHGDPRRPDVGKHLGFGMGIHYCIG
APLARLEVKLAMEMLLKRLPNLRLAPGNAFSYVPNFLHRGPHRLLVEWDPA

>MYSTI_07239|KEGG|CYP229H1|[Myxococcus stipitatus]|msd|DP

MSPSNPVVAATHPDPYPFYADLVARRPFYRDDTLGLWVASSARAVGAVLSSPACRVRPLP
EPVPRVLQGLASAEVFGRLARMNEGPFHLVVRRALMTVLGTLGAEHGAEEGRRQARRLIA
EEVPLRELVFRLPVQAVGALLGLEPGGLPEVASWTGALVRGMAPGADAEQVREGALAAER
LMAVFHARLAHSEDGPLTLLSRELGASSTAPGALSREQADAESSAVIVSNAIGLLVQTHD
ATAGLLGNALRASLADPAVYAQVSSRPEWLPRLLDEVLRFDPPVQNTRRFLSEDAVIDGR
ALRAGDTVLVVLAAANRDPAVHHAPHRFDLHREPSELATFGEGRHACPGAALARAVSLGA
LEVLFATDLVSHRAAFSATGFQPSVNGRVPLLPRLHLHSREHTT

>A176_002600|KEGG|CYP109S1|[Myxococcus hansupus]|mym|DP

MSNALWGVRPLVEFEPFSSVHWEDPYPVYRALREHSPVHWAPKAKLFCVSRHADVDFVLR
RPEQFSSRAMTAVMFENKRPELGDVPSLLRLVVRGRVDVQKFFQGPPDAIINSDPPRHGA
LRGMVSRCFTPRTIASWEPRIRELVNECLAPLRAGQPFDVVSGLAVPLPVVIVTEMLGVE
PEWRADFKRWAAGFITYATGAGRDTRPMREFFDVLTEFRAYMRTLVEKRRQAPTEDLISV
LVDPAQKEVLDEEELFSFIHTLLLGGNETTTNLIGNAVAALLDHPEQLALVQGAPEKIPG
LVEETLRWESPVQFTLRSAVEDVDVGGVRLPAGSNMALLLGAANRDERAFKSPDTFDVTR
TPSPHRAFGFGIHHCLGAGLARLEARVALEELVPLLPGVRRADARREYVDSLMMRGLARL
PLVPR

>A176_004746|**CYP253H1**|[Myxococcus hansupus]|mym|DP

MSTHSAGESTAAPLPPSPVGTPLLGHMRVLRSDPLTFLQAQVRQYGDVVRIHVGPASLVV
VAHPDGVRHVLQDQAKRYGKRTRGLAALRELLGHGLLTSEGSFWLRQRRLAQPAFHRQRL
AGFARTMVDAASDLASELEARADAGTAFDVAEDCMRLTLRIASSTLFGKDVSGAWHDIAD
AMGRVQVFTYKRLTQALPIPRRLPLPTHRRFERDTHMLDRVVRGIIETRRRDTGAHHDLL
QMMLEQQDADTGERMSDTQLRDEVLTMLLAGHETTANALSWTLMLLSQHPSVRRDLEAEL
AQVLGGRKPTDEDLPRLALTRRVVDEVLRLYPPAWSLSRVAIEDDVIGGFRIPKGTYLLL
SPWVTHRHPRVWDNPEGFDPDRFLPEHEQERPRFAWFPFGGGPRQCIGNQFALMELVLVL
ATLLQRVRLNLTPGQIIRPAPAITLRPRSGVWVTAARAP

>A176_006320|KEGG|**CYP197F1**|[Myxococcus hansupus]|mym|DP

MPQVRLPPGPKGHFIAGNLVEFSEDPLGFLTRCAREYGDVVRLGKRNFLLNDPDLIEKVL
VNGDGNFVKLAGVGQGQRHRGGFPEAMMNSEGEDWLRKRRLVQPAFHRKHVAACGDTVVS
LTEQMLQTWRPGDARDVHAEVSALALGIVSRFLFHTPIDDEARHVADAVDAVMRHTDSPL
RPPMWVPTPTNLRLRRALDRLDTLLTTLLRRYREHPESRTDLLALLLSSPVPLSEAQLRD
ELATMILSGHETTADALVWAWYLLAQHPEAEARLREELDTVLGGRLPGAEDLPRLSFTEA
VVKEAMRLYSPAWITSREALRDCELGGFHVPAGTTLAVSQWVTHRDPRYFDAPDAFRPER
WLSEDATRRHKYVYFPFGGGPRFCIGAALAMMETVLITACVARRYRLELAPGCVVRPRPA
LALQPLGVRLIPHPRAARGLESEARHAARA

>A176_007236|KEGG|**CYP1069A2**|[Myxococcus hansupus]|mym|DP

MSVPKQEETGAPAPASSGRCPHLGAQYNPFAGPHVENPHPFFDQLRKEEPVAFSPMLNMW
LISRYDDIAQVLRDPARFSNRDMLVSGTHAHLTEEARAILAQGYPTAHVLLGMDPPEHTR
LRRLMNQCFTPQRVASLAPLVQRMATELVERFDKTGQADLVTQLAYPMPVHVILGAVGVP
QEDFWRIKGWSTDWQRILFEYVPPEKQVDMAKGFLEFQQYCIRLIEDRRRNPRDDLASHM
LAVEDNGEALSLHELIMAIGGSMLAAGHESTTALLANAWKLALQHGLWQRLRDHRELVPR
FLEESSRYDSVSHAMIRTATEDVVVGGVKIPGGSRLLLLYAAASRDEALCPHANKLDLDR
EKVPQHLTYGRGTHFCLGAPLARLQFHVATNLLLDRLPDPRLVAGQDFGTTQNLVLRQMK
HLKVEWTPH

>MFUL124B02_14085|KEGG|CYP1506A6|[Myxococcus fulvus]|mfb|DP

MPNLLSRGLFNLFFGGRRAPLASLPGPTPGLLGNLGDFLGTQPWDVCARYAREYGPVTVA
WLGPSPALILNDADVIHEVMQTRRLEFEKGNIGEQIRHSTTDDTPFIAEQGDDWARKHAM
DPLAQPWTPAWHAAQVEPLRRALLESVEACFAHPSVDLTPTLRKMTFDAFCVATVGEKLP
ASSYDDFMLLAAGADARIQAKLPLRFVSAPKGFEAAKERFYGAFARRIHEARQARSSTAV
DLMSWTLREMPRLDDQVHAHLLGGFFFGGVFSSSTTLVGAFHQLNKYPATLERLALESDA
LAQGPLSLSRLVAEAPWGEAVAYEALRLLPAVRVFMRRSPLTPSRLAGVTLPPSTTLMVS
NQHLHRDPSHWASPETFDPERWLNGGAARDPLGSGHFFPFGRGPRACVAGDFAMVYLRTA
LATLSARARILVDSTEPFEEGFFFGVVRPQGVTGKITPRERSTLHPASPEAPLPPAVESA

>MFUL124B02_42165|KEGG|CYP1069A4|[Myxococcus fulvus]|mfb|DP

MPVTVQKQEETGVPAHAPTGRCPHLGAQYNPFAGPHVDNPHPFFERLRQEEPVSFNPMLG
MWLVSRHDDILQVLKETTRFSNRDMLVSGTQLTEEAKNILSQGFPTAHVLLGMDPPDHTR
LRRLMNRGFTAQRIAGMEPFIEKMARELIGRFAKDGHADLVEQLAYPLPAHVILGVMGVP
QEDVWKIKKWSADWQSLTFEHVPAEKQAEMARGVLEFQRYCVNLIEERRKAPREDLTSYL
VEVEADGEALSVHELVMAIGASFLSAGHESTTALMANTWKLALEHGLWGRLREDPELIPK
FIEEGSRFDSVQHAMIRTALEDVEVGGIQLPKGARLLLLYAAGSRDESLCPHANKLDVDR
EKPPQHLTYGRGIHFCLGAPLARLQTQITTRLLVEAVGNPRLVPNQDYGTWQSIVLRQMR
HLKVEWDVTP

>COCOR_00795|KEGG|CYP197J1|[Corallococcus coralloides]|ccx|DP

MHAAPSDSAVLPPRLPAGLPWVGQGLEYRKDPLGFFLRYADRGAVVRTRFVGTDIYLLNT
SEAIEHVLVKNFRNYPKDAFQKRALEAVVGHGLFTSHGDFWMRRRRMMQPAFHKNLLTAH
GSVAVKAASTWMASRREGASFDAYPEMMALTLDVVAETLFGANLSAQARELGRAMEAVML
HAQHLFDTPISLPAWVPTLGQRRFRAGLRALHAVVDDVVERRRRQGGPGEDLLGLMLEAQ
AEDGEHLTDAQLRDECLTLMIAGHETTATALALSLYLLARHPDAQAALRRELATVLGGRE
PTVADLPSLPYCEQVVKESLRLYPPAWGMSRVAEQDDRMDGVLVPAGTVVAWSQWALHRD
ASHFPEPEAFRPERWADGLERRIPRFAYCPFGGGPRLCIGAGSALMVIRLVLATVLQRIH
FEAEPGPAPEVLPAITLRPKNGIPLVARRVSRQE

>COCOR_02122|KEGG|CYP264K|[Corallococcus coralloides]|ccx|DP

MQTYDLFAPTTETERHALYARMRAESGLCRIAPFGAYAAARYEDVRTIQKDAQRFSSEAL
ATTAEPPWLGPNPVAQSLVTRDPPRHTQLRALINRAFGPTGMARLEAQVRQEAQTLAEAA

VSQREVDLVDAFSFVLPRNIIGRMLGLEPSTFTEFKRWSVNMGLITSAVPAQHAGIRDTV
KEMEDYLGGVITARRRQPGEDMVSDLLRAEVEGQRLTDAEVLSFLFLLLPAGMETTAQLI
GNAAILLARHPEQLEQARADRAHIPRFIEEVLRCEPPAQFAFRVATSDVELSGTPIPAGS
LVMGLVASANRDERIFEQPDVFAPGREKASQHLSFGHGIHFCLGAQLARMEARLALEALV
PRIRALRLRAPDIEWLPGLTIHGPRTLPVELLPA

>COCOR_02428|KEGG|CYP253D2|[Corallococcus coralloides]|ccx|DP

MHAGHVGVLPLRPMTTATAAVTRRAPGHRGHLLMGILPDVRRDLLGCMSTLHRQYGDVVG
YRLGPQRAHLIAHPDGVKHVLQDHVKNYTKDHLTYRMVRWLTGNGLLVSTGDFWLRQRRL
AQPAFHRQRIAGMAAGMVRRTEAMLQRWEPAAASGTPLSISEEMKRLTLTIVGEALFGTS
VEDQTERVGVAFTELGKQIAERFRTFRMLPPVLPTPYDRAFRAARASLQETVRGIIATRR
ERGDDSGDLLSMLMLARDEDTGEGMTDEQLGAEVMTMLLAGHETTATALSWTWGLLSKYP
EAEARLHAELDAVLGGRAPTVEDMPRLTYTKQVLEETMRLYPSVPIFSRTVDEDDVIGGF
HIPKGTSVNLCPYVTQRHPDFWEEPDAFRPERFAPEAAAKRHRFAYFPFSGGPRMCIGSG
FTMMEAQLIVATVAQRYRLREAPGFTLEPNANLTLWPKGELPMYLERRS

>COCOR_02429|KEGG|CYP253D1|[Corallococcus coralloides]|ccx|DP

MTTAAATATVTRQPPGPRGHLLMGILPDVRRDILGCLGTLHREYGDVVRYRLGPMRSHLV
AHPDAVKHVLQDHVKNYTKDHLTYRMGRWITGNGLLTSTGDFWLRQRRLAQPAFHRQRIA
GMAAGMVRQTQGLLQRWETAAANGTPVGINEEMMRLTLAIVGEALFGTSVEAQAGQVGAA
FTELSQQIAERFRTFRMLPPVLPTRYDRAFRDARATLLRTVRGIITTRRERGDDTGDLLS
MLMLARDEDTGEGMTDEQLGAEVMTMLLAGHETTATSLSWVWGLLSKHPEVEARLHAELD
AVLGGHAPTVEDVPRLTYTKQVVEEAMRLYPAAVIFSRSVQEDDVIGGFRIPKGTSVDVS
PYVTQRHPDFWEEPEAFRPERFAPEAAAKRHRFAYFPFSGGPRQCIGNSFAMMEAQLVLA
TVAQRYRLREAPGFTLDPDSHLTLRPKGALPMYLERRS

>COCOR_02734|KEGG|CYP1506A1|[Corallococcus coralloides]|ccx|DP

MPNALSRGLFNLFFGAKRKPFASLPGPQPGILGTAGDFLGASPWDVCARYGREYGGVTLI
WMGPNPGLVLNDPALIAEVYESPRRMEFEKGNISEQIRPSTTDDTAFTAELRGDWVQKRA
LEPSAQPWAPEALADLVGPMQAAISEFVDALLKQERIDFAPALRRLTFDAFSVAQVGEKL
PDQVFEDFMLLARAADARIQAKLPLKFVKPPKDFEAVKARFYGHFVDRIREARKRQDPGA
VDLMSRYLRETPGIDDQVLAHMLGGTYFGGAFSSSVTLVGAFHQLNKYPGADARLAAEAA
SLVADGPLTFQKVESAKWAEAVAYEALRILPAVRVMTRTPPKDAQLAGVTLPAGSMIMIS
NQHLHRDPAHWPDPDTFKPERWLDGGTTRDPLGSGHFFPFGRGPRACVGADFAMVFLKTA
LTTIASRVKVQLDSTEPFEEGFFFGVVLPKGVTGKLVARTAQASLKAKVG

>COCOR_03933|KEGG|CYP264D1|[Corallococcus coralloides]|ccx|DP

MSERFNLLSPEVKANPYPTYARMRREAPVCQVEPGGMWAVSRHEDVLRVLKDPQRFSSQG
FRVATNPPWLGGNPFSESMLTMDPPQHGRLRVLVQKAFGAGAMARLEPRVRDLCRQAVAE
LPRGVPVDLMPPYALRIPAAVISELLGLDPARATRLKQWADLITGGVTTVRPEEEDRKQR
ARDAVAELRQYFGEVLDARAHAPGTDLVSELQQARVDGEALSKDELIAFMALLLVGGIET
VVHLLGASLVVLREHPEIWAQLRSDRSRIPAFIDEVLRYEPPAQAAPRLTTEAVELGGVS
LPKGAPVLVLLGSAAHDEAHFPDGDRFNLSRPGPQNLPFGHGVHFCLGAQLARMEGRLAL
EAMLDAFRHLQAGPEPMTWHRTLVVRGPATLPLVLHPH

>COCOR_04510|KEGG|CYP1069A1|[Corallococcus coralloides]|ccx|DP

MSTAVEAVKVDGQSEAIHIDAHASKAAGAMKCPHLGAQYNPFAGPHVEDPHPFYAQLRQD
APVSYNPMLGMWLVSRYEDICHVLKDPTRYSSADLGNMGSVLAPATLAVLAEGYPLADSL
INSDPPAHTRLRKLLGRGFSAQRIAAQEAPIRAVSEELIQAFARKGHADLVTEFAYPLPV
RVILGMAGVPQEDMADIKRWCDDFFRMIFTRVPAEEQPPLARSWVTFQHYVARLIRDRTD
EPRDDLISYLVTTDADGEALTLPELIIAIAGSMLAAGHETTTALLAQCWKQALLQPGLWQ
KLREDRSLVPHLIEETLRFDSVAHGMIRTTMEDVELAGVALPKGSRLLLLYASGSRDAAL
LADGDHFDISRHHPTHLGFGRGIHFCIGAPLARLEALIATNLLLDQLPDLKLEENPDFGH
TQSLTIRTIQHLRVNWTPTGT

>COCOR_04899|KEGG|CYP1335A1|[Corallococcus coralloides]|ccx|DP

MTPPTDPYQAVTHPDPYPYYAELRARGGLHRVPGFGPWIAADAATVRAVLASEHCRVRPR
SEPVPPHLVGTAAGALFGRLVRMNDGGPYPAVKQTLSSALSLALREVEAESRRQAAWLFA
TPSLSRLPDAALQLPVFVLGSLLGFPEDTLEASVRDVDAYVRSVAGPPAGSEGAARLNER
VAACFQSAARTAFTARLMEGHGAEDLRLPNTVGLLTQAYEATAGLLGATLRAFARMPELR
EQLSRGGCTMDDVVQEAARHDSPVQNTRRFTHAAALVAGQALEAGETVVVVLAAANRDPL
ENPQPDAFLPRRTGRQTFTFGLGLHACPGPSLATTMATAAAERVLASGLDLSPLSGPVTW
RASTNTRVLGGLE

>COCOR_05111|KEGG|CYP264D6|[Corallococcus coralloides]|ccx|DP

MSTSALEKPLAPGPAVASRPQTPGMFFNPRDPAFNKDPYPHYKRLREEDPVHRSPWGVWF
LGRYEHVRGVLRDKRFSVQDVPGQLRRRSELLKTRKLIPNQPANLDGLIANSENWFAFLE
APDHTRLRNLVSSAFQKRSVERMREQIRQCAVELLEPVRKQGHMDLMKDFACILPQNVIA
HLLGLPKEDFPQCVAWAEVIGRIFDPLVSLEEYARLNEDSISFMAYLKELVAKRRVEPKD
DLISALIEARDGQDRLSEAELISIIILIFGAGEETVVSLIGNGSLALIRHPEHLEYLRTH

PEVLPSAVEEMLRYDAPLQMTSRTALEDVELGGKIIRKGDQVYVALGSANRDPDQFTEPD
SINLLRERNRHMSFADGHHYCVGAPLARIEAQEAYRVLFDTLGDFEVAIDELSYRDHTVL
RSMVSLPVRFKERR

>COCOR_06417|KEGG|CYP264D6|[Corallococcus coralloides]|ccx|DP

MTGRVNLMAPEVRANPYPVYEELRRTAPVCQVEPGGLWAITRFEDVAAAFKNPQVFSSAG
VRTTTAPPWLGHNPFSESMIVMDPPHHMRLRTLVSRAWTPAAVNRLEPRIRSFARILAER
LSPEREVDFVDAFAMPLPASVIGELFALDPTMTARYKRWSVDLSSVSGTTEKDTHRHESI
KATVREMEDYLSNVVAERRRHPQDDMVSDLVKTRVDGEALSDAEVMSFLFLLVVAGLETT
VQLVSHSVRMLMEQPHLMARLRENPSQVGRFVEEVLRYEPSVHGLVRVTTKETQVAGVVI
PEGARVALMVGSACRDGERFKDPDTFNMDREGVNNMPFGHGIHFCLGAPLARLEARVGLE
VLLSRFTRFTSTGPVKWNTSLTVRGPLSLPLIPHA

>LILAB_05370|KEGG|CYP197F1|[Corallococcus macrosporus HW-1]|mfu|DP

MPQVRLPPGPKGHFIAGNLVAFSEDPLGFLTRCAREYGDVARLGKRNFLLNHPDLIEQVL
VNGDGNFVKLAGVGQGQRHKGGFPEAMMNSEGEDWLRKRRFVQPAFHRKHVAACGDTVVA
LTETMLQTWQAGEARDVHADVSTLALDIVSRFLFHTPIGDEARHVAAAVDAVMRHTDSPL
RPPIWVPTPTNLRLRRALGRLNTLLATLVRRYREQPESRTDLLALLLSSPEPLSEHQLRD
ELATMIMSGHETTADALVWAWYLLARHPEAEGKLVAELETELGGRLPGAEDLPRLRYTEA
VVKEAMRLYSPAWMTSREALRDCELGGFHVPAGTLLAVSQWVTHRDARYFDAPESFRPER
WLSEDAQRMHRYAYFPFGGGPRFCIGATLAMLETVLITACVARRFRLELAPGCVVRPRPA
LALQPLGVRLIPRHRAHGARESEARHAASP

>LILAB_09935|KEGG|CYP1069A3|[Corallococcus macrosporus HW-1]|mfu|DP

MPVSAQKQEETGVPAHAQAGRCPHLGAQYNPFAGPHVEDPHGFFEQLRKDEPVTFSPMLN
MWLVSRFEDISQVLKSPAQYSNRDMLVSGTHLTDEAKAVLDQGFPTAHVLLGMDPPEHTR
LRRLMNRGFTAQRINGMAPFIREMASTLVDRFAKEGQADLVTQLAWPLPVHVILGVMGVP
QEDVWRIKRWSSDWQQLVFEYVEPERQVEMAKGVIEFQQYCIRLIEDRKKNPREDLTSYL
VAVENDGEALSMHELVMAVGASMLSAGHESTTALMANAWKVALQHGLWQRLRDHRELVPK
FLEESSRYDSVSHAMIRTAKEDVELSGVKIPAGARLLLLFAAGSRDEALCPHANTLDLDR
EKVPQHLTYGRGTHFCLGAPLARLQFEITTNILLDRLPDPKLVPGQDFGTWQSLVLRQMK
HLKVEWTPT

>MYMAC_007011|KEGG|CYP1069A3|[Corallococcus macrosporus DSM 14697]|mmas|DP

MPVSAQKQEETGVPAHAQAGRCPHLGAQYNPFAGPHVEDPHGFFEKLRKDEPVTFSPMLN
MWLVSRFEDISQVLKSPAQYSNRDMLVSGTHLTDEAKAVLDQGFPTAHVLLGMDPPEHTR
LRRLMNRGFTAQRINGMAPFIREMASTLVDRFAKEGQADLVTQLAWPLPVHVILGVMGVP
QEDVWRIKRWSSDWQQLVFEYVEPERQVEMAKGVIEFQQYCIRLIEDRKKNPREDLTSYL
VAVENDGEALSMHELVMAVGASMLSAGHESTTALMANAWKVALQHGLWQRLRDHRELVPK
FLEESSRYDSVSHAMIRTAKEDVELSGVKIPAGARLLLLFAAGSRDESLCPHANTLDLDR
EKVPQHLTYGRGTHFCLGAPLARLQFEITTNVLLDRLPDPKLVPGQDFGTWQSLVLRQMK
HLKVEWTPT

>STAUR_0027|KEGG|CYP1494A1|[Stigmatella aurantiaca]|sur|DP

MHLLPGPGLPPALQIARYRFQPYEFLDGCASRYGDLFTVRFPILGPLVCASRPESIRRIF
AASSEELRLGEANDIFRPLFGERSISVLDGPSHLKLRRLSVPLFQGEQSYAWTGMILEVA
SRRTRRWRPGQHLRLREEMEALTLEVILRALLGLEDPAQLRLASRHARTMVQWSASPLSA
LLMVPALRRDLGPLTPWKGYHRDLSTLAALVMDQAARRRRARDASGRRDLLSRLMQEGAG
LSDEELKDLVLMLLFAGYETTATSLCWAFEALLSHPGERTWVERELAEVTGGGPLEAGHL
EHLVRLDSAIKEVLRLYPVVPILGMARRAVRPFELQGVTFPAGTKLVPTSYLAQRRADVY
PDPTHFRAARFLDSKPDPASWLPFGGGLRRCVGLPFALHELKAVLAHVLSQTRLRLTRTS
PAHASLEGITVGPRGGTPVAVESVRA

>STAUR_0257|KEGG|CYP107CJ10|[Stigmatella aurantiaca]|sur|DP

MALHEPLPDPVPLTGCPYKANPYFLYKRMREAGPVHRVLFPSGVRAWLVTGYEAARSALN
DDRLGKNHGRGNDRWRARASIMPEPQHSQLQVHLLHQDPPRHTRMRRFVTDAFTPRRIEQ
LRPRFQALADALVDGLPEHGPADLVTGFAARFPFQVLAEFIGLPPELAGRFDRDWGKVVQ
PVGPTDPGRPLYEARLHGLQSYIAGVVAHKREHREDDLLSRLVVAHERSELSQEELGSMI
FQLLVAGQEPVTNQITTALIALFRHPSQLARLRGNPDLLPRAVEELLRYDSAFELTTWRF
FDRDSDLHGTRVPAGDSVIVSLCAANRDPRRFPDPDTLDFDRSPNPHLAFGHGIHFCPGA
ALARTELQIALGTLLSRLPGLHLSIRDEAIAWIPAVLGRGTNHLPVGYGRRL

>STAUR_0258|KEGG|CYP107EN2|[Stigmatella aurantiaca]|sur|DP

MGREVKWVCEGSGGMAPTGLGTRYSCKARPEHARHHKREGFLTLARDKRYRCVTRVRSPL
CRRMSTRNASRGDAQAIPGSPPHYPFGPVRALDLHPRYAELCREEPVSRVRMPFGGEAWL
LTGYAEIKQFLADPRFSSLKATAPDTARVTPLPLRPGNLLTMDPPDHTRIRRVVAKAFTM
RRVEQLRDRIRDVVDKQLDLLVAQGPPADLVASLAVPLPVVMISELFGIPYADREQFRRY

ADVFVATTAYDAVEIDRSRTALEQYFQELLEQRRSCPTDDLVSTLLEAMDTERLTPLETA
RTGIGILMAGHETSLSMISNSCFLLLSHRALYAQLVAEPLLLPTAVEELLRYIPLRSTGS
FPRRATEDVELGGVLIRKGETVIFQRASADRDERVFTCPEKIDLTRRPNPHLGFGHGAHH
CLGASLARCELSLAIEGLIRRFPNLRLAVPDGEVPWKPGLIARCPAHLEVTW

>STAUR_0406|KEGG|CYP1069E1|[Stigmatella aurantiaca]|sur|DP

MREPERDERGPLFIQCAVREGGAGGCVGKPPLFQNTLESPYHLGMPKTQPPVRVSPALST
PVPSSRCPHLGDEYNPFKGPHLQAPYPFFAQLRKEEPVTFSPMLGMWLISRLEDIHEVLT
HSASYSSASMLTSASQLTPEARAILGPGPILHDSPLNTDPPAHTRLRRLLQRGFLPARIA
RQEASTRQLANALIDTFIHKGRVDLVEAFTYPFPMHVILNMVGVPHEDMNRVKRWCDDLF
GLIFSQTPPEAQPSMARGVVEYRAYCADLIEKRRREPREDLTSYLVHTETGSDSLNTEEL
VSLLGGSLIGAGHEATTAQLGLILLNLLRKPERWQALCENPALIPRAVEECIRLEAASHG
MVRTAVEDVRIGGVMLPKGSRLLLLYSSANHDEAQHPDPERYTLDRTNLHHLGFGHGVHY
CLGSHLARLELCVAVELFTQRMPGMRLVPEQDVGYHQELLILRNISRLEVEWPG

>STAUR_0533|KEGG|CYP1489A1|[Stigmatella aurantiaca]|sur|DP

MQTTATKIPAPKGWPLLGVAPEMLRNPLPLISRLTRDYGDVVYMPGVDTYLISEPELVKT
ILLDAEEYFTKSPKVMKKLSPAIGAGLSTLSGDPWKRQRRMANPAFSRASIATFEGIMQK
AIAEAIVEWEARAGETIDVTGEMKRLTLRIVLLCLFSTDVSSRADEIIENLDVLQRYSVH
LLWSMMPLPEFIPTRKNREYQKARGTLDSIIYGIIAERRKSGNHDRKDLLAMYMSAVDEE
TGEGMSDEQLRHELMNLFLAGHDTTANGLAFTFYLLSKHPEALKRVDEERDAVLGNGRMV
TNEDLPQLNYARWSFEEALRIYPPTFAMSRTSVRELKHNDYVIPAGSNIFVCQWALHRNP
RLWPNPDHFEPERFSPERSEGRHRFAYLPFGAGPRICIGAHLARTEAQMIIAMLLQRFRL
NSEPGYEPRLVARLFVTAEPGISMRLSRR

>STAUR_0821|KEGG|CYP1011E2|[Stigmatella aurantiaca]|sur|DP

MNPVKLNLFDPAFHADPYPVYTRLREEEPVHRTVMGAWVITRYADVKVLLRDNTFGVFDI
PTRIERKSQFLERNKTGELRTLAQAMRRWLMLCDPPDHTRLRGLVNRSFTPSAVEALRPR
VQEMVDQLIARVRDTGKMEIVGELACPLAVTMIASMLGVPEEAHASVTRWADGLSRVLDP
LRSLEQYAEMNRVAQEFLEYFRQLFAERRRNPQNDLISSLIAVTDQGDKLTEDEMLSVCM
LLFIAGEKTTVNLISNGVLALLRHRDEFERLKQDPALIRGAIDEILRYDSPVQLNTRVPK
KDFTLHGKTIPAGDLIYFSLGSANRDPSQFPDPDKFDITRKENRHLAFSGGIHYCLGAAL
ALIEGQLAISTLAREFPKMELVPGTIDWQKEIIFRGPQTLPVLLNP

>STAUR_1562|KEGG|CYP107DP2|[Stigmatella aurantiaca]|sur|DP

MSTPDLFHELWSPEVIENPFPLYARMRQAAPVARSHHPSVAGPIWVVCRYHAAVEFLKDN
RFAKSKQKLGRESQRRYFRVSALKHLDQHMLSADPPMHTRLRSLVAQAFTARRVEALRPR
ITAIAEQLLDSVQQQDRVDLLDAFAFPLPITVIAELLGVPVEDQDRFREWTTTFLTPPKD
GDVAPLRRMAQEFQAYLQEFLARRRAEPRDDLASAMIAAEEQGDRLSPVELMSMVFLLLV
AGHETTVNLIGNGIWALLKHPEQLERLRAAPALLDSAVEEMLRYCGPVKHSTSRFTLEDT
EFHGQHIPAGEMVVAGLVSANHDAEVFPEPERFDIARQPNRHIAFGSGIHFCLGAPLALL
EAKLSFRLLLERLPQLRFAVEPSTLRWRDSLLIHGLERLPVTF

>STAUR_1628|KEGG|CYP264A2|[Stigmatella aurantiaca]|sur|DP

MTPQRVNILSPEFRADPHSGYAQLRRDTPVVQVEPAGFWAISRYDDVAFVIKNPQLFSSE
GFKAAWQPAWVGYNPLANSMIATDGAGHARMRTLVSRAFNASAIQRLEARIRKLAHRLAD
ELAQKGEADFVSQFAMPLPTFVIGELLGLDISLHHRFKGWSDDIASVTPEPLSPEYAQRT
LTAISDATRYITEVIEARRHSPAEDLVSDLVRAEVDGQSLTTREIVDFLILLLIAGLETT
VHLLANSLLFLAERPEESERLRADPTLIPRFIEEMLRYDSPVQSLVRIVTSDVTVAGVTI
PKGEVVLAIIASANRDERQYPEPDRFDLHREQSSISFGYGAHYCIGAQLARMEARCGLEA
LLSRFSRFQRTSAELTWSQAITVRGPHALPLRFTPA

>STAUR_1874|KEGG|CYP264A3|[Stigmatella aurantiaca]|sur|DP

MTRRVNILSDDFRANPYPGYAELRRNSPVTQVEPSGLWAVSRYEDVAFVLDNPLLFSAEG
IQAVWEPAWLGYNPLAHAMASLDGPRDAPLKTWVSQSFSPSALQRLEPRVRQLADILSDE
LLSRKSTDFIASFALPLPALVLSDLLGLEASDYRLFKNWSDDFACILPKVPSEDALRLRN
TVTRLTGHLTCLIEQRRLTPADDLISEMLRDDLPGPVPTDRERIELLTLLLVAGLETTVP
LLANGLILLAHQPELLAQLRADRTLIPSFVEELLRYDPPTQGVLRTTLEEVEISGVSLPK
GAGVLALTGSAGRDERQYPEPDRFLLHREQSSIAFGKDSLTCLGAPLARMQARIGLEALL
SRFEGFCLVPADLTWNKALTVRSLHALPLELTPVRAGALATSSAWVGSP

>STAUR_2030|KEGG|CYP120H1|[Stigmatella aurantiaca]|sur|DP

MTTLARESELPLPPKSPSLPVLGDTLAFLRDGPGLVETRGRQLGPVFQLNLFGQPTAVLL
GEEANRFVLHSGTKHFNWRYGWPPHFRDLWVDTIFLEDGDPHRLRRKLLMPAFQTEAIEG
YLPQMEKSHLAAIARWEKAGRMVCIEDFGRVTLRVANQLFLGDDTEDGAEDLPQWMKHWL
EGFYSVPIRFPLGTFSRALAARHKLISHVERAIHSRREKKTTDALGLLTGFKDSDGVALT
TDQLASEMLVLVAAGFHTTTSMLTYVMFALAEHPDVLKKARQEQRDLGIQGSVTLEQIHR

MSYLNAVMAEIERLYAPVPFGFRRVVEPFEFHGYRIPAGWQVAWSVEGAQKDPRNYADPL
RFDPERFTRAGSDADHRGDFKLVGFGGGPRMCIGTNFAKVQAKLLMSHLLRGYEWEVEPD
QDLRPRFTPERTPKSGLRVRFKKRAGPPDSPA

>STAUR_3582|KEGG|CYP1498A1|[Stigmatella aurantiaca]|sur|DP

MSYAVANTAFYPRYDLLDPDFPKSPYPLLHRMRTEEPVFWFEPLNTWVVTRYEEVSSILK
DPMRFSSRRADRMLQAQLPSDTPSGIRQTIAHVMSLAAMFSDPPVHTHLRSEANKGFSPR
AMAPMAQRIQKVTDELISRMEGRGEMDGFLDFFEPFALTVIGDLMGVPREDQHLFIPWTQ
KTTKLLGGSKLTLEEAQASLRAFEEMNAYLAQALAERQRNPRDDMMSFLLAGYDPGRFPL
EALAGMCSEIIGGANAPTGDTLGNALLACLSHPEELEKARHTPALWKTAVPELLRYDGPI
LFWSRLATEDVVLGGKRIRAGQMVYASLAGANRDPDVFPEPDQLDFSRPNAHKHVSFSFG
PHHCIGAGLARMEMTIVFETLFRRLPGLRLAGPVQWREGSLTTRGPTRIPLVF

>STAUR_5213|KEGG|CYP1486B1|[Stigmatella aurantiaca]|sur|DP

MSTAAAIEHLPGEAGIVANVRNTLGWMRRGNEHLLEQHRRFGPVYRTVFAGYTVVCVADP
EMVMSIARDDHSWSTALAWLTFFQGIDAKINASQIDSPLFLDFKPHRDARKILQPAFGHT
ATAGYFDAATPIFEKAIDRWVEQGHVSFKDAIRSLLVEVSTRIFLGVDSGAREFERALID
YWEGPLSLSRSALLSSKWRRSIRGHRVLCEMLRSRIAERRATGGDDLFSRLCAKTQESEG
ILDDDGLVRLVIGVMAAAFATTSSGLASMAYLLAIHPEWQEKMREEALAVSKGRVSYEDS
KQLEVTSRVWKETMRLYPIAPYCARRALHDVNLGQFRIPAGTFVMGLISVVMQDSALWSN
PQRFDPDRFTEERAEDKKSKASFLPFGTGAHTCTGMHLANAEAKSFWHAMLTRCRFTLER
NYRGRHTYMPAGIVSGDVKLRVERL

>STAUR_5214|KEGG|CYP126B2|[Stigmatella aurantiaca]|sur|DP

MSVEPPNRSAPLGGLHGPPSTPQPLPAVLEGFDLTDQSRFSNGFPHQVFARLRRDAPVLF
HPPGQSIDGDGFWVLSKHADIREAAANPAFSSRGGGGRPHGGTHIDDARPELPGVLFNMM
DDPRHQDFKDVMTPAMGRQALATLEGKLRPYAAELVSSLLARGNCDFATEVGATVGAHAI
SQLLGIPSQDWPLFAEWTSLLMGFDDREAGRPTERSQKIHMDIFRYGCQLLAAKRAGPSD
DLTSLLAGGKLASTSGEGPISDLERQTCFCLMVLAGTESTRNMIAGGVLALAENPEQWRA
LRADRSLVPSAVEEILRWTSPTPYNRRTATRDIRIRDTQIRAGDKVTLWWTSANRDEDVF
SNPFSFDIRRKPNPHLAFGYGSHTCFGDHLGRLEMRVVLEALLDRVEQIELTGQVAWAGS
NKHTVLLRMPVALTGA

>STAUR_5220|KEGG|CYP110U2|[Stigmatella aurantiaca]|sur|DP

MKSAKSSREGFPEGPSSAFWTSVRYARDPVGLYRALTKYGDGHTVTMPLLLGPIVGAISP
QSAKDILTADTASLDIFGPEALAMVFRPRSLVMLSGEQHARERKLLMPAFNRADAVRAYA
VTMQETALQYASEIAVGKPFVMQKLAQRILLQVVIRDVFGVTEPDEQAELKQRVRELFEA
SSPALIFFPGLRHRFGGIGPYANWQRADARLTRLIHDLIARRRASPPGSKVDVLSLLLTA
RYEDGSVPSDEVVHDELMALFFAGHAATATTISWVFHWLHRYPETLAKLRAELATLPQDV
EPTSYMKLPYLEAVCNETLRIYPPVADLYRKLRVPLRIGSTMVPAGTGVAVFTTIIHARE
DLFPEPMRFRPERFAERSYTPFEFLPYGVGARRCIGAAFAHQALKIVVASILRRWELGLE
TPDEPAVRQGVGVGPKHGVRMRILRNATEEQAPGATGRSAALS

>STAUR_5324|KEGG|CYP167B5|[Stigmatella aurantiaca]|sur|DP

MTREAPPAVKFDPSTAEFDVNPYPMLERLRAQPALPYWEEGRGWLLCRYEDAIAVLRDNK
RFTANRSVWEFDAELGLTHQIPELAELSQYGLFALADADHARVRKLVSPALTPRAIERLR
PEIQAIVDEILDGVEAKGTVNVVAEIADLIPARVIGSMLKIPKGREVQFQRFTDAMIKNF
VPGLVCAREVEAMRPDIAGGLELIRETVEDRRRHPQEEDILTTLIRTEERGDSLNAQELL
SLVAALIVGGFETTVHLISFTVYNLLQRPEVLAQVKAEPELSRNVIEEVLRFDNFGKMGL
ARYALEDVELGGVTIKKGQLVLVMLNSALRDEGTFSSADVFDVRRHTNASIAFGHGVHYC
IGANLARLEVQLAVSTLVRRFPRMQLLGPPSFGPHPVLRKMESLEVQLRPQ

>STAUR_6876|KEGG|CYP1387B1|[Stigmatella aurantiaca]|sur|DP

MPLSQTFHPFAPPLRDNPHPIFEEARKEEPVFFSEILKCWVVTRYEDVYAVLQDSRRFSS
AGNLAVHKESLCPAAKSLLIENQYTDPPLVNEDAPVHTRRRPVYKKLFSQEAIRSLEPRI
RDITKELVDGFAHQGHADIVSQLAYPLPMRVVLSWMDIPQEMMETIKRWGDAWVLLLFAE
LTPEHQMECAHMVVKLQRYIADLLAERLDQKRTDAMTQLATQLLTLEKLSIEELAGMVSG
TVVAGNATLTSMVGLTTRVFLQQPNLWQRLREQPERISQAIDEALRLESPFWGLRRVTTE
PVEVGGVKLPAGARVLVAFISANRDVERFPQSEVFDLDRPNASQHMSFGKGVHVCVGSGL
AKQEVQAAMEVLLQRLPDARLVPGQELTFVPGLLRQHERLLVEWSPQRV

>STAUR_7270|KEGG|CYP264J1|[Stigmatella aurantiaca]|sur|DP

MHSTADLMTPENCRNPYPLYEELRRDRPVCHVEPGGLVAVSRYKDVEHVLKHPEVFSSHG
FRVAWEPEWVVDNPLAHGIVAQDGAEHLRLRSAVSRAFTPVAINRLGAQVRETAAQLADA
LLEQGEADFMDGFATPLPARAMSGILGLDPSLERHYKRWTEAVVGITPVPLSEKHIHYTR
ATIVEMKAHMQRAIDERRQQPTDDLLGLIVRGKSDSEPLSNSQMMALCFNLLTGGLETTS
FFFSNAVRVLAERPDVYAQLRADRTLLPQFIEEVLRYDGPVRGLPRIVTSDVELSGVRIE
RGACVLLLVASANRDEAQFQAPNHFDLQREQKGISFGYGTHYCVGAFLARLEAQAGLEAL

LDRFSGFSLIPERLVWNRGIITSGPEKMPVRFLSA

>AA314_00027|KEGG|CYP109R1|[Archangium gephyra]|age|DP

MSLLDHFDFFHPEVHNNPYPYFAELREKAPLFWTKSLQAHVLSRYEDVAYVLKNPALFSS
VDIRVNGMRPQDRNELGELSSVTNLVNSDPPIHTRLRGLVSRAFLPKRISEMEPRVRELS
RGLVAEMTAQGEFDFMDGLASPLPVTIIAEMLGIEISRRRDFKRWSDSLMSATAESLRTG
KMSEEATRSAGEMFAYMKEIAEKRRLEPKGDLISLLVQSSEGVEALSPAEVNSFAVLLLI
AGNETTTNLLGNSLLALIRHPEQYEWLRNNPSREACAAVAEETLRYDSPVVGLMRRATQD
VELGGGKVPAESTVMVLVASANHDPRKFPNPERFDPQRDTNGTLSFGHGIHFCLGAPLTR
LEAPVALQELMERAPRLGFASRQPERIDYGSSFFLRGPRSLWLRKS

>AA314_00028|KEGG|CYP107HX1|[Archangium gephyra]|age|DP

MTPEINLKTRELRANPYPTYARLRKESPVVLYKHPIFGKTYYLTRYNDIVSSFTDPRLAN
DRRNVLEGKKDPMDRWWVPKLFRILQNNMLAKDVPDHRRLRDLVHKGFTPRRVEEMKQAV
ERIVSELLDAAEKKPAVDLLADFALPLPLTVISEMMGVPEEDRLTFHRQMGGLLDNLTTP
AGFLLQTPNAFNMMRFFRKLIRLRQTEPRDDLLTALVQAEEQGDRLNEDELISMIFLLLL
AGHETTVNLIGNGTLALLQHPEQLQKLREHPELIGSAVEELLRYGNPVDQPSPRYAREEI
QLEGHVIPKGATVMCLLASANRDESVFENADTLDITRKPNRHVAFGMGVHYCLGAPLARL
EGTIALQHLVRRFPDMKLAVPAEQLRWRGSIGLRGLKALPLLLSPSGAPAAMREAA

>AA314_00098|KEGG|CYP197G1|[Archangium gephyra]|age|DP

MLGNILAAAKGELDFFKELGSYGDVSSFHLGRRRFYFVNHPELIRKVLTSQNFVRTAISR
DLMESFLGRGLFSQEGENHLQQRRLMQPAFHHKRIEGYGAVMEGFATRMLEGWRDGETRD
ISHDMMRLTFEIVSKTLFDADTGEQASQVGKAFTSIMQAINKEYPLYSVLPAWVPVKRWG
ASRRAVEALNRITSTIIQERRGTGTDRGDLLSMLLLARDEDGTRMTDEQVGAQTLSLLFA
GHETTANLLSWTWLLLSRNPDIRLKLQQEVDTVLEGRAPTVGDLPRLKYTESVVKETLRI
YPPAWYAERAPLTDTELGGYTIPANAPIVISVYVTHRDGRFFEQPERFWPERFLGVEDRQ
LPAYLPFGGGTHLCIGNRFALMEAQLLVAAIAQRYELELEPGLAVKPRALITLGVEGLRM
KVKRRQPGSASPRASPASSASDSSA

>AA314_00303|KEGG|CYP107DL4|[Archangium gephyra]|age|DP

MATNPPNEQQQETAPVTAPHEAPRADATHILDRTNPEFFERAHGLYSDLRALGPVVRAPM
RGDLTAGAHAEGSPERQARDAGQERYFVTRYDESVSALLDDRLSSDFRTGLPPQQREFLN
KAMPEEFKPIAFSLIMMDPPDHTRLRKLVQPNFTARAMEALEPRVQRLVDELLDKAEQAA
AARGEPASERCMEFVEAFAYPLPVRVISDMLGIPEEDRAQVHAWAERLLHAETPAARADP
QVRAGLKAFASYLEGLFERKRREPGEDMISQMVHAQEDGDRLSHQELLSMVFILFFAGHL
TTVNLLGNGVVALLNHPEQHARFLADPAGMSKGMVEETLRYWGPVDYLSTPRIATESFAL
GDTQVPQGAKVSVGLASANRDPERFANPDAYDISRPDAHRNIAFGKGIHVCIGAPLARLE
ARIAFETLFRRFPKLRLAVPESELRWGAGAAMRGFNRIPLFF

>AA314_00471|KEGG|CYP253G2|[Archangium gephyra]|age|DP

MPEARTLGEAPGPRGHWFWGNIQERRHDPLQLFLRAHQEYGEFVRLRMGPTISLISVTSP
ELIKHVLVDHADRYGKPSSLVRDAWPVLGNGLFTSSGDFWKRQRRLMQPSFHKERLATLA
GSMVDATRQMLARWRARPSPSEPLDVAAEMMHLTLGIVGRALFSTDVSGNASRVGQALTV
ALAETNRRILSLGLYAPDFLPTARNRAFRQALGTLDSVIFDIIARRRAGETQGDDLLATL
MAAQDADTGERMTDAQLRDEATTLFLAGHETTANALAWLWHLLAQNPEVETRARAEVQRV
LGGRTPTAQDIPQLRYLTQVFEEAMRLYPPAWIMARQPVKEDVLAGVQIPASPRVIVVTP
PYAIHRNPRLWPEPERFDPERFSPEQTAQRPRMAYLPFGGGQRLCIGNNFALMEATLIIA
EVLQHFRLRAVPGHTVEPEPLVTLRPKGGLPMFVEPLHASA

>AA314_00680|KEGG|CYP1347C1|[Archangium gephyra]|age|DP

MASAARKLQLNPVDPENLLNPVPFYKELRENDPVHWSDVVHGWFLTRHEDVMNGLRDSRL
SADRGKVLEHQFQMMGLSPESIREVTDTFKRQMACKDGAAHLKPRRQTAAAFAPQVLDSW
RPLIHRTLVTHLDKLRDRREADLVPELFYQMPPLVIAELLGITPEDRERLQQWGEAVTQL
TSTGAAKNLMEVARQANEGQARINQFLTNLVEERRRAPGEDLISHMLRAQEQGGMTTEEV
VANTSLLLAAGHLTTTDQLSNGLYELLTRPEQLALLRREPSLLKPAIEEMMRFAPPVPFV
HRIAVDNIELRGRTIRKGDIVFLGLAAANHDPAAFKDAEYFDISRDPHQQKSMSFGFGPH
HCLGAGLARRELEIAFSELVHRMPEMRLDAAKLPRIKCGGLLFRGFESLPVRW

>AA314_01068|KEGG|CYP1069C1|[Archangium gephyra]|age|DP

MSKTNAPSGLGSEYHPLDSPQLEEPHPFLARARREEPVFFSPALSSWVVTRHADISAVVA
DTARFSSAESITVGAATMPPEVVTVLMGGYPLVPSLVDNDPPAHTRFRSLVSKAFTGRRL
TELEPFIRTRVDELLHSFRHEGRADLFLRFAHPLSSSIIAEILGIPRSDIHRFRRWSDDL

STVLAAHGPVEHQVACARGVVDFQRYLASALEERKTAPREDLLSDIVTGAQGMTPPMSMA
ELVSLLMQVHFAGHETTAGLIVGAVELLLHHPEQLRALRDDPGLIAGAVEEALRMTSPVH
AMFRTALEAVELGGVSIPKGAHIRIVYASANRDEARFHEPERFDPRRPDVKKHLAFGQGL
HFCIGAPLARLEARLALEALLRSLPGLRLVPGQKPGFLKNVTVRRHESLEVAWDV

>AA314_01308|KEGG|CYP253G1|[Archangium gephyra]|age|DP

MTPSAPLPPMPPGHWLWGHLPERASNPLGLYLDSQRRLGDVVRYRMGYIHVEQLTHPDHV
KHVLADASARYTKGTVFDKTRPLVGNGLLTAEGDFWKRQRRLAQPAFHRERLAALGTRMT
DTVAELLPAWERAADGGTPLPVFQELMRLTLTVVVRALFGVDVAPHTTTMGKAFTTALEV
TNERIISPLPYLPWLYRLPTRSNRAFRQAVDTLDGMVRGLIAQRRAAGTQAEDLLGMLMA
ASDADTGDTFNDAQLRDEVMTLLLAGHETTATSLAWTFHLLARHPEVEATLHAEVDAALG
GRMPTVADLPRLRYVGCVFEEAMRLYPPIWAIPRVPQEDDVVDGYRIPKGDIVILVPYVT
HRHPDFWPEPERFEPTRFLPENSKDRPRWAYLPFGGGQRQCIGNNFAMMEAQLILTLVAQ
RFRLREVPGVPVVPEPHVSLRPRGPMPMRVERREEVPRVRVA

>AA314_01700|KEGG|CYP197K1|[Archangium gephyra]|age|DP

MIAIHNEPPGPEGAWGIANVADFHADPLRFVTRCAREFGDVVRLGQDNYLLCHPRDIEHV
FVNSQRSFAKETGQKPTWAKTGSYHHALMHTDGKDWLYKRRLLQPLFKRDRVDTGAEPIV
AAAEEMIASWRPGEVRALEGDVAPLSAQVVGRFLLGSDLSANEVQRVTSFLAWSFRPLPI
RIPRWLPTPRNLRFRWSVLQFDRAIYGLIERLRERSRGSSNLLELVQQARDGEEGCLAPP
WYPRDELAIMLLAAHQPTAIALAWTLYLMALHPEVEARVAAEVKQTLGDRRATAEDAERL
VYTSAVIKETLRLYPPVWMTARDSLQEVELGGYRIAAGVSLALSPWVAHRDPRWFADPDT
FRPERWLDGSLEGLPKYAYFPFGGGPRLCMGAWLAKLALVLVIATVVRRYRLTLAAGAKV
TPFAAASFLIPQGVRLVPTART

>AA314_01792|KEGG|CYP264D2|[Archangium gephyra]|age|DP

MTGRPNLMTPELKANPFPLYAELRRNAPVSQVDPGGMWAVTRYDDAMAVLKNPRTFSSAG
FGLATNPPWLGGNPVSQSMGALDPPQHGRLRTLVNRAFTPTAVARMEPRVRDFVARVATA
LPLGRPVDMVPAFALPVPAYVLGELLGLDPSLHSQLKRWADQLTSVTAIRAEETERQEPV
RQAVAEVRRYFSEVVELRRQRPGEDLVSDLLRARVDGEALTQDELLAFMFLLLLGGLETT
VHLMGLCVLALMEHPEVMERLRADRSLVPNFVEEVLRTGAPGHGLLRLTTEEVELGGVRL
PKGARVVVLLGSVCRDESQFKDGERFDLDRPGSQLPFGHGPHYCIGAALARLEAKLATEA
LLERCGGISPGEGPVVWHRSMVVRGPSSIPAVLHPA

>AA314_02215|KEGG|CYP109D3|[Archangium gephyra]|age|DP

MSAPLSPDVLNTPEGISNPYPAYRAFQGPNPVRYLRLPAGPLTGRTEPLYAWALLRHADV
LAAIRDPATFSSQTPAVFKTMPKFALLHDDPPHHTRMRRLVSKAFSPQRIASLSEWIGRL
ANELLDTTGRGPVELMAAYAVPLPMRVIAAMLGVPDQDYPSFKLWSEAVVAYVGMSKEER
MRKMQEMAAYLDQAIAARRAQPAEDLLSALVEAKVEGEALTDLEIRGFAAVLLVAGNETT
TNLIGNMLALLADRPELWRRAREDRSLVEPIIEEVLRYESPVQRFPRLTTRPVKIGDVEI
AEGEIVDMVYGAANRDPSIFEDPDSFNPERPTNSQHLGFGLGTHFCLGAPLARLEARVTL
NAFLDRFPALGRGEGAAVRQGFAPVSLGYKSLPLVLG

>AA314_02385|KEGG|CYP110R1|[Archangium gephyra]|age|DP

MSTPQLPSGPRITPVQTFRFIRDATGFFVDCRARYGDPFTAPLPVGNVVITGDPEGIRDI
FSADPATFEPLGQLPLAPAVGDNSLLLMGGQRHKRERKLLMPPFHGERMRAYGQLMRDIT
LRAVETLRPGGPFRAQELTQSISLEVIIRAVFGIEEPERVRRYREVLVGYMEAYTPLLMM
AVPLRRSFGGRGPWARFQRHVAELDQLLTEELATRRGHEAGHTDILSLLLAARDENGQPM
TDAELKDELRTLLIAGHETTAIGMAWALYHLHRVPETKQRLLEELAPLGPTPDPEALTKL
PYLTAVCDESLRLHPVVAVVGRRTVAPFTLRGRELPPGTALMAAIALAHVDPVIYPEPER
FRPERFLERKYSPFEYLPFGGGARRCIGAAFAQYEMRIVLGTLLAMHRFSLASDTPERPV
RRNVTIGPGGGVEMVYQGPRQGAVA

>AA314_02449|KEGG|CYP1284B1|[Archangium gephyra]|age|DP

MSPFSGQKLDKLPAHVPPELVYEYDNARDPRMLEDPHARMRSLILEAPPIFFSPCNGGMW
FVTRKKAIVDITMNPEVYSNSFFEAAAAHAGEHAGERQDAQPGFRMLPISEDPPRHTAYR
APLNQPLAAKTVSGLETAIRTMTNELIDKVLAAGRCDFFSEIAEPLPVTLFMKLAGMPTD
RLAEFRDLATQATSATVDAATRAVTFKRIAGILAEVIKARQEKREDDLISKLLDANIHGR
NPTFEEMLGYSVTLFLGGLETVVNALSFGVRHVARDQELQAKLRADPSLIPGAIEELLRL
YGIASTPRRVMRDEVCHGVQFKKDDMVLLLLPAANYDDAAFPNPEQFILGRKEQHQTFNT
GPHRCVGLHLARLEMKVFYEEWLKRVPPFRLDPQAPPRFMGGFNLAVTSLPLVWG

>AA314_02454|KEGG|CYP107DP4|[Archangium gephyra]|age|DP

MTASPLFAEGETLWSPHVRANPFPLYQRMREEFPAARITDPGTGMHFWLLTRYADVVSIV
RDPRFTQDPSRLPDEVRQRYFSHKNRALTQHLVTADPPDHTRRRSLVSKAFTPRRIEELR
PRIKELCSELLEKLMAQGSGADFLKVLAFPLPIMVIAEMLGIPPEDGDQFREWTQTFFTP
PSKDAQEKTRNVHQLFINYLEGIFEQRRQQPRDDLISALLAVQEQGDRLSAEELTSMVFV

LLVGGYETTGNLLGNGLLALLQHPEQLQRLREDRSLIPSAVEEMLRYCGPFELSILRFAK
EDMELHGQRIQAHEAVRVNYLAADRDAGQFSEPDRFDVGRTPNKHVAFGHGIHFCLGAPL
ARLEAAIAFEVLLERLPELRLATAPEKLVWRPSAQSRGLASLPLAF

>AA314_02467|KEGG|CYP1011E|[Archangium gephyra]|age|DP

MELLDQPAVNGGAGSGVPRAPVKFNLFDPAFHADPYEVYARLREEDPVHRTVMGAWVITR
YADVKTLLRDNTFGVFDIPTRIERKSQFLEKKNMGELRTLAQAMRRWLMLCDPPDHTRLR
SLVNRSFTPGAVESLRPRVQEMVDRLLARVRHTGRMDIVNDLACPLAVTMIASMLGVPEE
AHASVTRWADGLSRVLDPLRSLEQYAEMNNVAQEFLAYFRELFAERRRHPREDLISSLIA
VTDQGDKLTEDEMLSVCMLLFIAGEKTTVNLIGNGTLALLKNRGEMERLRQDPSLIRGAI
DEMLRYDSPVQLNTRVPKKDVTLHGKTIPAGDLIYFSLGSANRDPTQFPDPDRFDITRRE
KGHLAFSGGIHYCLGAALALIEGQVAFSTLVREFPDLELVPGEIDWQKEIIFRGPQTLPV
TFSP

>AA314_02616|KEGG|CYP109C5|[Archangium gephyra]|age|DP

MMNLFSDEVRRNPYPAYEQLRSRSPVLHEPHSDLWMVFDYESVKRALQDHEAFSSIVAPP
SAMTSQWLVFADPPRHSKLRALLLRAFTPRAVASLEPRILALSHELLDRTMARGEMDLAE
DFSVPLPLRVIAEMLGVPTTDLPHFKRWSDVMMALGYAVAGSEEAARVQREFSEVTEEMR
VYVRDLAGQRRETPREDLLTRLVEAEVEGERLTEDELLGFVQLLLSAGHETTTNLLNNAL
LCFLEHPEQLARLRAEPALLPSAIEEVLRYRSPVQAMFRVTRSEVSLHGQVIPPGKMVLA
MIGSANRDPRKFQDPDRFDISREPNPHIAFGHGIHFCIGAPLSRLEARVGLSVLLERMRD
VALASDAPWEPRRAIHVHGPTRLPIRFTPGT

>AA314_02730|KEGG|CYP267A3|[Archangium gephyra]|age|DP

MTAIPMEVADPFGLLHPERRKDPYSGYARMLREAPVHFCEPWGAWLVSRYADVNAGFRDL
RLLSARTGQFSAGMSEEVRKMMEPLARNLSSWTLFLDAPSHTRLRGLISKAFTPRMMEAL
RPRIQALVGELLDAARGKERMEIISELANPLPVIVIGELLGLPREDRYRLKRWSDQLAGL
IGTGRPTQEQVTAGLNAILEFEAYFRTLIAQRRTAPTNDLLSALVHAEEKGNLLSEQELL
STCTMLLFGGHETTTNLIGNGLLALLRHPEQWELLCASPELLPDAVEEFLRYDPPVQFTN
REAAVDMEIGGQAIRKGERVMLMMAAANRDPAHFKDPDRLDVRREELRHMTLGMGPHYCL
GAALGRIEAQETFAALMRRFPRLKLEPGATLDWVDSAGFRGLLSLPVVLGAEAR

>AA314_02743|KEGG|CYP110V1|[Archangium gephyra]|age|DP

MNRLPPGPRNNLLVSLKYLREPYATLLETGRRYGDTFAWPGFAGKMVVTGHPEGVRTLFT
ADPDTFLALGAEMLAPILGESNLILLSGERHRAMRKLQLPPFHGARMRAYGQLMLDAARE
HAARWTRGRPIPVHRTMQEISLQVILEAVLGLREPAERESFKTTVLEVIAALKPSFMFIK
ALRRPLLGLSAWARFQRKREEITALFADVLRARRASPEPHEDILSLLLSARYDDGHALTD
DELLTQMLNLIVAGHETTASSLAWAFHFIHREPAVKARLVEELRGLPEPLEPEAVSRLPY
LEAVCSEALRLNPVAPIIGRTLREGLTLQGYELPPGMDVGISILQVHRRPELYPEPERFR
PERFLERGYSPFEYLPFGGGARRCIGAAFALYEMKLVLAAILRTYDLLPASDAPVGVSVR
NTTVGPRGEVGLLLA

>AA314_03310|KEGG|**CYP1329A1|**[Archangium gephyra]**|**age|DP

MDGLPRPRRSPSGALPRSRPHPLPTARDIPRFPGGFLLGNLSDFRHHRLKLFRDIKERCG
EIGTIRIGPVPVQVVTSGSISHTLLHTHANDVDGGPLVKTFTPLVGERSLVLLHGPQHRK
QRKLLAPSFQREQLAHFTQTMVQYTLDAIEGWRDGQTFDVHDEMATLTMRIIGKTLFDVD
YRHETNELLGAMGQALEHIEYLTSSLVQIPLSWPTPRNRRVREALRTIDTTFRGMIRQRR
VDHAQRKDMLSSLVQVRDEEGQPIDEDQLHDDLMTIYSGHESTALSLGWTWYLLARHPEV
LARVRQEVDTVLGGRPPAFEHLPQLRYTLQVIKESLRLYPVIFMLARAPTKDLEIEGYRL
PKGQMVLMPPYIIHRDEKFFPDPERFDPDRFLPEREKQLPRYAWFPFGAGPHTCIGNHFA
LMEKQLIVATMVQRLHIELVDGQEISPEVLTVTLRPSHLKMRVEHRKDAPVSRVA

>AA314_03966|KEGG|**CYP1248B1|**[Archangium gephyra]**|**age|DP

MDDISTRFNPMAPDQLDNPYPLYARMRRERPVFYSPMFDLWVVSRYADISEVERDTASFS
SVGALDARAEPHPEVRAVLAQAYPRFLSLVQSDIPDHTRVRAVFGKALSTHRIAAMEPTI
RATADALIDGFIRDGKADLIQQFAYALPGFIICDLLGVPRSDMEQLKRWSDDKTLLMSAT
APIEQQVQSAHGFIAMERYFQEQIHERRRHPREDLLTMLVPQSLGGTAPLSEQEAVCNAM
DLFAAGHETTTGLIGNGMWLLFNAREQLEAVREDLSLLPNALEEVLRVESPIRGFFRTVM
ADTPLSGVTLPKGSRAFILYASGNRDETQFTEPDRFDIRRAEAKKHLAFGKGIHFCVGAP
LAKLEGRIAFEQLLRRLPGLRPRTDEAPVFRPYFMLRGFEHLPIAWDVPA

>AA314_04153|KEGG|**CYP167B4|**[Archangium gephyra]**|**age|DP

MSANPMNPEARPAVPFNPYAPGYDVNPHPTLAKLRAEAPLFYWEQGRGWIISRYDDAIAV
LRDNKRFSSSRADWEFAGMVGSAVSIPELLELNKNSLLSLSGQDHARVRKFVSPALTPRA

VERLRPEIQAIVDELLDAAAAKGTIDVVADLAEHVPSRVIGTMLKMPKGQEALFMRFTYA
VVKSIIPTLLRPEEVPELRTDLREGTRLLHEVIDERRRNPLENDILTTLIQTEEQGDRLN
TQELLALVSSLIVGGFETTMHLIAFTVFHILQRPEVLAQVKADPELLKNTIEEVLRFDHF
GKVGVSRYPLEDVELGGVKIRKGQMMLILLGSTLRDETAFDKAEVFDVRRNTNASIAFGH
GAHYCIGASLARLEVQLTVGSLLQRFPELRMVKPPSFAPHPVIRQMKDLEVKLSP

>AA314_04160|KEGG|CYP1298A1|[Archangium gephyra]|age|DP

MTHSFKSVPRIKESALLGSWSEFKSDRLGLVLRVMRECGELGEFHVGPVSMYFPTGPKLL
HQILVEQADKFTTEPFMANFYPVLGRTTLPGIDGAHHRRIRRIMAPAFQPKRMMAYAEHM
VGFADEVQRGMVDGSEVEIVGVMQHLALKILAKSVFHLEFNEDKYFFDCIHVASEYVGVR
VSNPVQLPLWVPTPRNRRDQAAIAHLRGRARSLLEEGRQRGDKGDVLSLLLTAKDEDGTG
LSEAELLDQILTLYIAGHETTANALSFAFMLLGQHPESQAKLQAEVDSVLGGRAPTYEDA
PKLTYTLQVIKESLRMFPPAVFFGRAPIEDLEIEGYRFRKGQFCLLSPYAIHRNPEFFPE
PERFAPERFTPENEKKLPRNTYLPFGTGPHVCIGQYFALLEAQLVLAHICQNVNVSLVPN
QQIRLMPLATLGPSPFRVKVTRRKTATALAS

>AA314_04162|KEGG|CYP1347B3|[Archangium gephyra]|age|DP

MTGSGTPETTPTQSLTESAARKARLNPLSPENLLNPVPFYKELRETSPVHWSEELQSWLI
TRYDDVTNGFRDPRLSSDRGALFEYQMKALGAKSPEAFVQRNRLMMINRDGVSHLNQRRQ
TSPGFSPQSLDVYLPAIRRIMRSLVEQVQDRGEMDLVQEVSYQMPLLVLVEFLGLPTEDR
QKFKDWSKPLADFVNPMPGTDPVAAANAAITASGELSNYLDRLINERRQEPGTDMISRMI
QGGKMSQEELVANTHVMITAGHTTTTDQLGNGLHDLLSHPDQLQLLRENPSLVKSAVEEM
LRFNPASPFFTRIAAEDFQLHGQDIKKGQLVFLGMAAANRDPKYFPDADRFDITRDNLMH
KPMTFGFGPHHCLGAGLARRQLEVAIETLLERFPRLRLDESRKANPRAEGLVFRGFHSLP
VRW

>AA314_04396|KEGG|CYP1007C1|[Archangium gephyra]|age|DP

MPLLGEFGSQFRLFSDFIGGLSELQRHGPLVSLARGSGRCILVFGPEYVQQVLSNPSLFL
SLEPGKNAPPGSSLRRLWTGLGTTNGEVHKKGRRLMLPAFHKKRVEGYRDDMVSLFQRML
DGWRPGETRDLSADMRRVTLLVVTKALFGLEDEQECIAHGELLSEWFRLFGSASVQLLQH
DWPLLPYRRVMRISEKGEAAIRDIIARKRRDGGGGHDVLSLLLEARDEDGGSLSDEELVG
HITILFLAGHETTANALTWTLFLLDQHPDVHAALVDELQGTLRGEAPALEQLEQLPLLDR
VIKESMRILTPVPLSQRMAAAPFEMGGYSFDAGTEILYSPYVTHRMPELYPEPARFLPER
WKTLDPPVYAYIPFANGPRRCLGATLAMMELKLALAMMLQRYRLALVPGSRIDREVLITL

NPKHGMPMTVHAQDRRFTRAPWRGNVREMLALD

>AA314_04497|KEGG|CYP264K2|[Archangium gephyra]|age|DP

MQTYDLFAPAITPADRSALFAKMRSEPGLCRVEPFGAYAAARYEDAAAILKDPKRFSSEA
LAVTAEPAWLGPNPVAQSMLSMDPPRHTPLRALVTRAFGPAGMARLEAQVRRASETLAEA
AVRQGEVDLVDAFTFVLPRDIIGVMLGLDPSTFSEFRRWSTNMGLISSARTPEQYEEIRS
VVREMKDYLTDVIHARRRQPGEDMVSDLLQAEVEGQKLTDEQLLSFLFLLLPAGMETTSQ
LLGNAVLTLAQHPEQLELMRADKTHIPRFVEEVLRYEPPTQLSFRMAMQDVELSGTKVPA
GSLVLGLIGSANRDERVFDQPERFLPGREKATQHLSFGYGVHFCLGAQLARMEARLGLEA
LVSRIRGLRLRSPEVQWLPGFTIHGPAVLPVELVAA

>AA314_04571|KEGG|CYP264E1|[Archangium gephyra]|age|DP

MSQPLNLMTPEVRANPYPLYAELRRRPVCQVEPGGMWAVSRYDDVVTVLKDPRRFSSEGL
GRSFRPPWLERNPTADSLVMKDPPEHTRLRSLVTRAFNGATLSRLEPSIRAIAEELADSA
VRRQEVDFIAEFALPLPARVLNLFFGLEPAMALRLKHWADDLVSIPASQPSPQRQEEIRH
SLQEMERCFNALIEQRRARPGDELVSELLRGELTHEEVLSFLFGLVPAGVETTVYLLANT
MVVLSEHPRELERVRENPALIPRLIEEVLRFEPPGQSSLRLTTEDVELSGVRIPRGSIVV
ALVGAAMRDESRFPQADQFLLERESQSSLAFGHGPHYCLGAMLARLEARLGLEALFSRIG
GFSLQRKELDWSQSIIARGPRALPLHLEPMR

>AA314_04912|KEGG|CYP262B2|[Archangium gephyra]|age|DP

MPLVGALPGLLSKRLDFLEAARQRHGDIYTLDLGFTDAIILSHPRHVQHVLVDHARKYFK
GGALWESMRTFLGNGLPVSEGDFWKRQRRMIQPAFHQQRLIALTEKMVAAIDESLAGWEP
AARTGEPFDIAEAFSQMTMNVMVRAMFGSGLEADEAKQVAQALAYIIDYMMQGMVTKSLP
EWLPVPGRARYREAIRYIDEVVLRVIERGRQRSEEDNLLSLLLHAVDGESGERMTNAQLR
DEAVSLFVAGYETTAVGLAWAFHLMTRHPESFQRLRTEVREVLGQRVPGFADLRQLGYAR
NVLQEALRLYSPSYWIPRTSSEEDELDGFQIPAGKMVVVFTHLIHHHPGVWEEPQRFDPD
RFTPERSEGRHKQAWMPFGAGQRLCIGKEFSLMEGQHILARIAQRYDVSAVPGREAQVHI
GTSIRAKNGVWVHLKPHAP

>AA314_05283|KEGG|CYP1347B1|[Archangium gephyra]|age|DP

MTGSTVTDRNLFSPENLRNPIPLYKDLRENDPVHFVEPMQAWFITRHDDVMEGFRDPRLS
ASRGQLFEYQLQGVDPNVAREFLGTIQHQMFMKDGSEHVRLRRQTLTGFSNQKLDDMRPT
VHRIMRELLDEVYPQGHMDLLKDIAFPMPALALAELINVPLADRARFKRWSEYLADFSAP

AVGANPVELATNANQAMVEMKQLLLPVIEERRRNPGTDSLSLMIQAEAEGRMTPEELVAN
ICLIMFAGHTTTTDQLCNGLYDLLTHPDQLQKLRADMGLMKSAVEEMLRYTSAVPSITRV
AGEDFEWHGKQLRKGQLVFLMMASANRDPSAFPEPDRFDITRDSTQQKHASFGFGAHHCM
GAGLSRRELEIGLTLLLERLPGLRLDETRQPRLKCHSLTFRGFDSLPLRW

>AA314_05303|KEGG|CYP109N1|[Archangium gephyra]|age|DP

MSLPVPQFTPEVTLDPYPFYAALREAGPVPFVPSLMGGCYALTRQEDILSVLKNPVLFSS
KLMAPSAMMPKELGEDVLRLFQVENNLLTSAPPLHTRLRTLVSRAFTPRRVAELEPRIRK
LCGELLDTMLAREEFDLIPALAAPLPVIVITEMLGVEPERRPDFKRWSDDVMRAISLRSA
SEDLGRLRESILEFHAYLETIVERRRREPRNDLISAIIEASEAEGGFLSTLDIMAFTRLL
LINGNETSTHLISNGMVALLRHPSEWERLTADPSLLPNAIEEMLRYDSPGQAVMRLVTED
TELAGHRLPKGSRLLLLLGAANRDPHRFPEPDRFDVTREVQGLVSFGHGIHFCLGAPLAR
LEAKVVFEELFRRVRRVSFAPGQEGTIPWNPALFIRGPKSLRLRAERHPSA

>AA314_05305|KEGG|CYP109N2|[Archangium gephyra]|age|DP

MQTPSLPPMAELLANPHPFFAALREAAPVLKVEAFMGAYVVTRHEHIGAVLKNTTVFSSR
GMGFEQVLPPELGEEVLRYFRVEHNLISSDPPEHTRLRTLVSRAFTPRRVAELEPHLREI
TRGLLDTLLARDEFELMAGLAAPLPVTVIAELLGIEPEHRQDFKRWSDAILQAASFTIRD
KSQLGQSIRQLQDYLEAAIERRRREPRNDLMGALVEASEAEGGFLSTLELIDFVRLLLIA
GNETTTNLIGNGMVALLQHPSEWERLVADPSLLPNAIEEMLRYEPPAQSVLRVTTQDTEV
AGVPIPKGSRIMLLLSAANRDPRRFPEPDRFDITRDAHGHLAFGHGVHFCLGAPLARLEA
RVVFEELLRRVRRVSFAPGQEDHIAWGETFIVRGPLSLRLRAGRR

>AA314_05406|KEGG|CYP107CJ11|[Archangium gephyra]|age|DP

MSGIPDDTLPDPVPLMGCPYKANPYPLYERMREASPVHRVLFPSGVQAWLVTGYEAARSA
LNDERLGKNHGRGNDHWRARASIMPEPQHSQLQVHLLHQDPPHHTRMRRFVTDAFTSRRI
DQLRPRFQELADALVDGLPEHGPADLVTGFAARFPFQVLAEVIGLPPELAGRFDRDWGKV
VQPVGPTDPGRPRYEARLHGLQGYIADVIAHKREHGEDDLLSRLVVARDRGELSQDELGS
MIFQLLVAGQEPVTNQLTTALIALFHHPGQLARLRGDPDLLPRAVEELLRYDSAFELTTW
RFFDRDSELHGTRVPAGDSVIVSLCAANRDPRRFPEPDTLDLDRGSNPHLAFGHGIHFCP
GATLARTELQIALGTLLARLPGLHLSIKEEDLEWIPAVLGRGTHHLPVGYDQRL

>AA314_05534|KEGG|CYP1347B2|[Archangium gephyra]|age|DP

MYRQLQEDDPVHWSEELQAWLVTRHEDVTGCFRDPRLSANRTQLFAEHQLGGLGPELVKD
YLRVAEGMMLMKDGTEHTRLRRQANTGFTSQLLDGWRPTIQRIIDSLLDRVQERRRMDVA
TELSEPLPALVMTELFDIPAEDHERFQRWSNDTANFFGAAVGDVRSAALRANEGSAQLSR
YLTAAIEARRERPGEDLLSLLLRYEEQGRMSTEELVSNAVLILTAGHVTTIDQLSNGVYE
LLTHPEQLEQLKRQPELCKAAVEELLRYTPAVPFMHRVVAEDLELRGRTLRKGQLVFLGI
AAANRDPNVFADPGRFDITRQNNKYVSFAFGPHLCLGAGLARRELELALRTLVCRMPDLR
LDEQRPPRLKCNSLLFRGFDSLPVRW

>AA314_05592|KEGG|CYP120G1|[Archangium gephyra]|age|DP

MDVSHLPLPPGSSGLPLLGETLAFVRDSGKFIEERRRLHGDVFRSHVLGDPIVFMIGPEA
NRWIFAGENKYLRNRWHRSARKLLGQQSVAMINGSAHLERRRLLAPHFTYAAMRHFVPRI
EALVHRHCEQWLSAAQPFKAVDAVRALVFEVAIVLLMGESRVDAAYMSRLFKQWAAGLFA
AVPLDIPFTTFGRGAAAEKEMREYLTPLVAEREKLAEQPPDTLGSLLSARDEHGRPLPRE
TVLDELQLLLFAGHDTTVTATSNLMMLLARYPEVLERCRQEQQQVAEKGPLTLESLRAMP
YLHQVIQEVMRFIPPVAGAFRVTTQDVAYGGYRIPKGWQVSLGIRGTHQRAPWTSPERFD
PDRMGPERNEQKPQGVYIPFGGGPRVCVGQHFAMVEMAVVSSLLVRGYQWELVPGQDLSP
IPFPIPLPRDGLLVRFSRR

>AA314_06132|KEGG|CYP1007D1|[Archangium gephyra]|age|DP

MTQPAALTGVALRGPRPLPLLGAKGNFLRFFSDPIGQSLRLHRDYGEVCALTDGDPSVLC
LFGPAYNQQVLSDRNLFHHNDKAFFPVPETARQLLSGLTSMNGELHRRQRKLMMPPFQKG
RVEGYRDDMVALTEHALSRWTPGRTLDVSGEMTELTLQIALRCLFGIEDERQAHDLGHLG
MDFLEGLTSPAVMMLPRALPGTPYARFVRTSNALHARLLELIRHKRTLPEGRDVLSMLLR
AHDEDGSALTDEELLGQASVIYIAGHETTSHTLSWTLFLLSQHPRVLADLEDELSGTLRG
AAPSVEQLSRLPLLDAVVKESMRLLPATPLLFVRFATDGFQLGPLALPKGTGVMLSPFIT
HRQPDLYPEPDRFLPERWQRLQPSLYEYLPFGAGPRMCLGATFASQEVRLVLATLLQRFR
LSLAPEARVSCQVRGITLGPRHGLPMRVGGRDTPLPPPVRVRGDIPRIVRLES

>AA314_06415|KEGG|CYP167B3|[Archangium gephyra]|age|DP

MSLQTPSQSPSVSFAPDAPGYVLDPYPALAELRTKAPLYYWPEGRAWLLSRYEDATAVLR
DARRFSPNRDEWEFAAIAGSAALIPELAELNKGGLFALSGSDHARVRKLVSPALTPRAIE
RLRPELQAVVDDILNDLAGRDTLNVVKDISDRIPPRVIGSMLKIPKGRETLFQRFTEAAI
KSFLPGLLRPEEVPSLREDVREGIALVRETIEDRRRNPLPDDILTTLIQTEEQGDRLNTS

ELLSLVAALIVGGFETTVHLIGFTVYNVLKRPELLTQVKAEPELLKGVIEEVLRFDNFGK
LGIARYALEDVELGGVTIKKGQMVLVLLNSALRDESVFPKADVFDVRRNTNASIAFGHGM
HFCLGASLARLEVQIAVETLLKRFPALRLVKEPSFGPHPVIRKMESLEVQLNQQ

>AA314_06425|KEGG|CYP1502A1|[Archangium gephyra]|age|DP

MEPNAPEAAPHDVRDWRDHAAFFLNLSRLCPDEVVPYMAQGRPAFLINRPEHVTHVLSTA
QANYRNPYHPYAELAGLYQPEGSFLLRLGHRAEHRSAYTHQLTRMVESARARFRVLGESS
SQGPVEVERELKELLLCVFAQALFGVDVAAESESFVRAVGLLEEVQANHVFARGADTGPL
RLRYQQAVEDQQRLVERIVQGSTGMREAVEEAPERHSLVHTAIIRTLLNGYNAMATALGW
TLHLLGQHPETQRQVREEALGLEEELLTIRDLPRLRVTRCVVQESLRLYPPAWMLGRHVR
EEERLGGITLPTGAIISVSPYTMQRHPRVWEEPDAFRPERFLGAESRQPGAYFPFGGGMR
SCPAEHSVVGPLQLLVATLVREHQIEPVPGVTVRPRGLVSLRPSPTLRLRFLSLA

>AA314_06601|KEGG|CYP167E1|[Archangium gephyra]|age|DP

MQTTSTREFGPDFVLNLDDPTFVTNPYPTYKWLREQAPVYHWRPRGDAIVFTRHKDVRAL
LLDRRFSNDYRVWEFAQNTSAWPPEHAEYKKMMDNGLFGLADADHTRVRRLVSGAFTPRA
AERMRGEIQKAVDDIIAENVKGDRANLTSVTEPLPMRVISDMLKIPDDKRAEFRAFGLAV
IRASITFNKPEELFALIAPMPRWLQMIRELIADRRQHLLEDDLLSTLITAQDSGSKLTED
EMISLIQALITAGSDTTVHAANWALHSLLRHPDQLALLRADPSLIRNTIEESLRYDLFGK
GGIPKFAKEELEFAGQKVRKGQMVIPFVTAALHDPEVFPEPERFDIRRDVSQTIAFGAGQ
HFCLGAALARQELDLVVGTLVQRFPKMKLLGEPEFQPHPIMRAMSKLEVSLG

>AA314_06640|KEGG|CYP107HX2|[Archangium gephyra]|age|DP

MVETARVDLQREFKAPEARANPIPVYAKLRKLGPVLRSADLFGEGFVLPRYEEVVRVLKD
PRFANDRRNVPGGSSLDRWWMPAIMRMLISSMVLKDPPDHRRLRNLVQKAFTPVMVENLN
GRVEQITEDLLDAAARKPVVDLMEDFALPLPLTVIAEMLGVPQSDRIPFRALIAKVLDSS
AVTPISFIRNYPNMLRLNSFLRKLVKLRREQPGDDLVTALVQAEEGGDRLSEDELISMVF
LLLFAGHETTVNLIGNGVLELVRHPEQLQKLREQPDLIDSAIEEMLRFTNPVGIVAPRFA
KEDVEIAGVPIPKGSALTLLIASANLDETAFPHADELDITRSPNRHVSFGYGIHYCLGAP
LARLEARVAIPALLRRFPRLDLAVPAEKLRWRPHIGLRGLEALPLSVSLAGSAAERSAA

>AA314_06855|KEGG|CYP242A3|[Archangium gephyra]|age|DP

MSHPPLVFDLLSLKAAQDSHSLYHRLRAQEPIHWSPQLQGWLLTRHADISNALRDPRMVA
GPMTGQFERLPEAARKQLLPLRDAVNMWMGHTTHEGHLRFQSLLKRYFTPRTVDNLRPRI
QSITDALLDAAAAKGGSIDLVQELAIPLPSSVIAEMLGVPLTDEYMLQRWSRDLTAIFSA
FDVPQLLRSQKSVMEMMNYMRDALAQHRRAPSENILEVFVRAQAEGLVSEEEILSNCVLL
LFAGHETTARLISKGMWLLFQHPEQLAKLREDASLIPQAVEEVLRYGDVVGFITRLTVAP
VELGGQAMQQYQMVYLALAAANRDPEVFAEPDRFDINRKPSKHVAFGFGSFYCLGAALAR
LEAQVFFETLLRRFPHVRPAPGTQPTWEPRLPLNRELRALPVEL

>AA314_06918|KEGG|CYP117E1|[Archangium gephyra]|age|DP

MQALTNLFSRSPRKAELREGIPVLPGAVPVLGHAPYTLGNALTFLRDAESKVGPLFWTKN
IGRYTLVCMGEPGFELLKNKVTSSEFIREGSPDFIGDSLMSRDGASHRNLRSAMSAAFTP
RGLTASGAASIAAEVVESYTSRLPQEPFSLLDLTQGLTLDVIFRIIGIDNEELAAWNRNY
REFVLGALPIKLDFPYSPARRARQGKQWLDSRLSNLISKARGKPEMPGTLSALLKARDEE
GKELTEDELIQNLRLLALAGHETSASVMAWMGIVLAQQPALWEQLRDEAMAAPAMPRSPE
ELKRHPFAEALFREVLRLYPPVSWTGREAIEDLELYGKRVPKGTTILVPVGTYGHDPKLF
PDPERCDPTRWMGRRLPPSAIETAAFGGGPHFCLGYHLAWVELVQFATGFAREVARRGMK
PQLAPGTAEPKLTYFPFGAPPKKTRIQLVPA

>AA314_07253|KEGG|CYP1286A1|[Archangium gephyra]|age|DP

MQPPRIQLPPFLQLHQWVAHPLRFMEESVQRHGDSFVMQFPNTPPFFFTRDVEMVRQLFT
GKPEDLYVGPVNRVLESVVGKNSLLLLDDQEHIRERRMMTPPFHGERMLSYGRSMREAAS
RSIDGWPQGKAFTLHEVFQDITLDIILETVFGLAEGETFTHFHELFARMLDMGSHNAVLY
MSALLPAERMLKLLAVGRDPVQLGPWKVNVSRALPWTQLSRLMQEVDAYLFAEIARRRAE
GVEKREDVLSLLMQAHDEQGQPMSGQELRDEAITLLAAGHETTATTLAWAFHFVLQHPEV
EEKLRAELRQVAGTGPLSPEQVNRLEYLDATLKETLRLVPVAPAVGRVLQRPMKVGGWDL
PAGMGVMACAYLTHRRPDLWPEPERFNPERFLGKRPSPYEYFPFGGGARRCLGMAFATYE
MRIVFAEVLRRVKLRPATPYSFQVSRRGVTLAPRGGVPVIVESRN

>AA314_07540|KEGG|CYP264D5|[Archangium gephyra]|age|DP

MNLLAPKVRMNPYPLYAELRRSAPVSQVDPGGLWAVARYADVLTVMKNPRLFSSEGIRRT

YRPPWISNYPMADSMLVMDPPHHTRLRSLVNRAFGASVMTRLEPRIRELAQQLATTLPAG
QTMDFVDAFSIRVPIAVLGDLVGIPPSLHTRLKQWAEVMTQFTSVGPEDTGLQERIRATV
AEAREHFEQVLEERRRAPREDLVSDLLRARVDGEALTATDLMGFMFLLLIGGLETTVHLL
SASALKLQEDPALMARLRAEPELIPRFIDELLRHSGPVHGVVRLVTDEVELGGARIPRGE
RLLLLMASANRDEAHFPEPDRFDLDRPGPQNLPFGHGVHFCLGAQLARMEARLALEALLA
RFSRLTLGEQPARWNISLVMRGPTLLPLAAHRD

>AA314_07541|KEGG|CYP264D4|[Archangium gephyra]|age|DP

MSSRVNLLAPEVRANPYPVFAELRRNAPVSQVDPGGLWAVTRYADAAAVLKNPQLFASEG
FRQAYRPAWFSDYPMADSMLVMDPPHHTRLRALVNRAFGPPVLTRLEPRIRQFVEQLAAG
LPTGRSVDFAEEWSVRIPLIVMADLLGLSPEVHPRLKRWADTFGSFTGIGPNDTERQEHM
RATVAEARQHFQQVLEERRREPRDDLMSDLLRARVDGEALTDAELMGFMFLLVVAGMETT
VHLMNHSAIRFRDEPELMGRLRADHSLIPRFVEEILRYEPPVHGIFRVTTQETELGGVRL
PKGARMLVVLCSANRDEAQFPGADRFNLDRPGPQNLPFGHGIHFCLGAQLARLEARLGTE
ALVTRFSRLSPGEGPVRWNTSLVVRGPLSLPLVAHPA

>AA314_07542|KEGG|CYP264D3|[Archangium gephyra]|age|DP

MSGTERVNLLAPEIRANPYPTYAELRRHAPVSQVEPGGLWAVSRHDDVLFVLKNPQLFAS
EGFRQAYRPPWISNYPLADSALVMDPPRHTRLRALINRAFGASVITRIEPFVRQLAARLV
AAIPEGRPVDFVEHFATPMPMGVIGELLGLSPEVHPRLMHWAEHLARFTSIGPNDVEKQE
AMRTTVNEARGHFEQVLEERRRNPGDNLVSDLVRARVDGESLTDTELLGFMFLLLIGGLE
TTLHLLSHAAHRLHLQPEVMTLLREQPILVPRFIEEVLRHEPPVHGMIRLATQEVELGGT
RVPQGARVLILMGSANRDEAHFPNPDHFNLDRPGPQNLPFGHGIHFCLGSQLARLEARLA
VEALLARFSRLTPGDGPVQWNASLIVRGPAKLPLVAHPG

>AA314_07761|KEGG|CYP102M4|[Archangium gephyra]|age|DP

MSKPALSTTIPQPRPRPLLGNAPDIGSETPLQNMMKLARELGPIFRLSFPGASIQVIGSY
ELVADACDETRFEKMVTQALLHLRALGGEGLFTVETEDPKWSKAHRLLVPAFSPAAMRDY
HDGMLDVADQMLTRWARFGPETDIHVSDDMTRLTLDTIALCGFSFRFNSFYQTQLHPFVD
SMVRALEEAGNRTRRLPVQTQLMVRTRRQFEADTGYLRELTQQLIEKRRATRPGEAPRDI
LSLMLEAVDPLTGERLDDTNIRDQLVTFLIAGHETTSGLLSFALYYLLHNPEVLQKAQAE
VDRVFGSEPPRFEQVSQLQYIDQILRETLRLWPTAPAFSLHAKAHDTLLGGRYPIGPDDP
LLVLIPMLHRDPAVWKDPERFDPERFAPEVRDSIPPHAWKPFGNGMRACIGRAFAMQEAT
LVLGMMLQRFHLSEPEPYKLRIRETLTLKPDGLKLRVRERKPASRPVAPRPTPRPAVPVS
QPSEKAAAHGTPLLLLYGSNSGASEAFARRIASDGLARGYSARVAPLDEYTGKLPREGAV
FIVTASYNGKPPDNARAFHTWVSQLPEGSLEGVRYAVFGCGNKDWGDTYQAVPRHLDERL

SAAGARRLFARGEADARADFFGEFEHWYAPFWEGVGTALGVSSREVASGPLFTVEVVPSA
SVELVRQNKLELATVVENRELVDMTSPFGRSKRHLVFKLPEGVTYAAGDYLAVLPENHPE
LVERAARRFGVRTDAAVVLRSTRGDQASSLPTGRPLSVQELLGRHVELSAPATRKDLERL
AEKNPCPPHAMHLAALAQDAERYKKEILDKRVSVLDLLEQYDSCILSFGDFLELLPAMRV
RQYSVSSSPLAETSRCTLTVAVVDAEAWSGRGRFHGTCSSYLARLRPGEQAAVAVRTPNV
PFHPPASNATPIILVGAGTGLAPFRGFLQERALRHARGEAAGPALLFFGCDHPDVDFLYR
DELAAWEQQGVVKVLPAFFRQPDGDVTFVQHRLWKERERVKALLGQGALFFICGDGQHMA
PAVRETLARIHQEAVGCSAEVATQWLSELEKQGRVVSDVFT

>AA314_08132|KEGG|CYP2624B1|[Archangium gephyra]|age|DP

MSAAPPEAPTAHPTAAIGQDYNPFDPAFQSNPYPFYARARNEAPVAYCPQFNVWAVTSHE
LITHVLKSPTLFSSLHNLEYTVVLPAEIEALLATGHYPHAPGLFNNDPPGHTRVRALFSK
AFTAQRVAEMEAGIRTLTEGLVEQFAHQGHVDLVAGLAHPLPMTVIADLLGVPREDMHRV
KQWHDSWMTLFSPQTPLEEQRVAARSCVDYQNYYAGLLADRAAHPTGDLTTALVQARVDG
ENPFTAAEMISQMMILLSAGHETTTSLISSLLYNLLRNPEQWQAVRDDPKLLEAAIEETV
RFTSPVQMEPRVAKKDTELGGVAIPQGARLNIIYGSGNRDERAFPDPETFNVHRPQPARH
MGFGWGIHFCIGAPLARLEARVALEVLRERLPHLRLAPGFMPEYEPSFFFRALKKLELVW
DSQA

>AA314_08208|KEGG|CYP253G3|[Archangium gephyra]|age|DP

MMTGAREAPGPRGHWFWGSLRERRSESLGFLVRMHREYGDVVQWRMGPVNRVVSLVNPEH
VRHVLVENVGRYTKGSGAQRLSTMLGNGLLTSEGPFWMRQRRLAQPAFHRERLMRMVGMM
GEEVEHMVERWRARPDTSAPVEMADEMARTTLSIASRALFSTQVLGEADRVLPSLTVGQQ
VVNERVLSLFPLPLSLPTPGNRAFLRARQTLDSVVFEIIARRRRGETEGQDLLAMLMEAQ
DAETGERMTDAQLRDELMTLFIAGYETTANALTWAWYLLSQHPEVEQRARDEVAQVLGER
APGPEDIPRLRYLAQVLEESMRLYPPAWVMVRQAREDDVLDGIRIPADPRLIVAVSPYVI
HRQPRFWPEPERFDPERFSPDRAAERPKWVYLPFGAGQRHCIGSHFAMMEMVLVLAGVLR
RFRPRLVPGRTVELEPLVALRPKGGLPMLLEPVAASG

>AA314_08399|KEGG|CYP107EN3|[Archangium gephyra]|age|DP

MRMPFGGDAWLLTGYAEIKQFLADPRFSSLEATQPDTARVTPLPLRPGNLLTMDPPDHTR
IRRVVVKAFSMRRVEQLRDRIREVVDEQLDQLVARGPPADLVASLAVPMPVVMISELLGI
PSADRERFRRHSDVLVSTTAYDAAEVNRTRTALEEYFQELLEQRRSRPTDDLVSTLLEAM
DTERLTPLETARTGVGILMAGHETSLSMISNSCFLLLSQRELYARLVAEPSLLPTAIEEL
LRYIPLRSTGSFPRRATEDVELGGVLIRKGETVIFQRAAADRDERVFTCPEKIDLARRPN
PHLGFGHGAHHCLGAALARVELSLALEGLIRRFPDLRLAVPGDQVPWKPGLIARCPARLE

VTW

>AA314_08581|KEGG|CYP1492A1|[Archangium gephyra]|age|DP

MNTALSVDTPRTLKEYAPSTLELLTAIRREGFLGWLMSTWRKHGDLIRIRMGSKSLLLVT
HPDHVRHVNVTGRERYDKGESYDSLRELLLGHGIVTATGEDWRRQRRLMAPFFTPRGVEK
FFPIFLSDTQKLIEHWRAQHQGSGRPVEMLDEMMQVTAAVILHSVFSTDSGETLLRVKNS
IETLVSHISNSQMSPLQVPMWVPTPGNLRFNRAKKLVTAYIQELIARRRAIPTEQWPDDL
LTKMMTTRDEESGTFMAEQLLVDNGLTMFAAGHETTARTLSFLWYALSQNPEVERRLHAE
LDSVLGEAPPTINDLKKLPYTLQVVKEVLRLYPAAPMYARDAVADDELDGVRIPAGTRTI
VFSYATHRHPAFWDEPERFEPDRWLPEREAAQHAHAYHPFAIGPRICLGNNFSLLETHVM
TAMLARRFKLRLKPGHVPRIDMFGTLGSANGMPMLIEAR

>AA314_08585|KEGG|CYP107HV1|[Archangium gephyra]|age|DP

MQTLTQPDFKSQRFKANPFPFYARLREEAPVFRIKVPANEAGWLVTRYDDVNQVLKHGGI
SKDRIGSMTPEQQAKMPWFFKFFTPLVQNMLSRDPPDHTRLRALVHKAFTPRLIERLRSR
VQTLSDSLLDTAARKGSMDLVAEYAFLLPVTIIAEMLGVPPGDYKKFQHWSNRLVSNTDM
SDVLLSIPSVVMFTRYLRKLIAQRRTSLGDDLLSALIQAEEAGDKLTGDELVSMAFLLLV
AGHETTVNLISGGTLALLQHPEQLERLRKEPGLIEPAVEELLRYASPVEVATERIARDGV
TVSGVTIPRGDLVFAVLASANRDERHFKDPNTLDLGRDPNKHVSFGMGIHYCLGAPLARL
EGQIAIQTLVNRFPNLRLSKPAESLKWRTGVLMRGPKQLPVKLT

>AA314_08757|KEGG|CYP264L1|[Archangium gephyra]|age|DP

MTRPNIYSPEIWNDPHPFYAALRREAPVSQVEPGGMWAVTRYEDVLYVLKNPQLFSSEGF
RPAVQPAWLQQRNPVADSILMMDPPTHTRLRALITRTFSASGVARLEPYIRQSAGEIVDG
MLARRKVDFMAEMALVLPSRAMSALLGLDPSLAPRFKTWADAVTGVGIIPADDLKRRAEL
TGYLDELTHHLTGVLELRQREPGNDVVTDLLQARAQGGSMSDDELLGYCVLLLVAGLETT
YDLLGMSALTLADNPDLWARLKKDRALVPKFVEEVLRFHAPSQSTIRTTTQETELGGVRL
PKGATLLVQFVSANRDESVFPNAHRFELERTGSPHLTFGQGIHFCVGAPLARMEARLALE
ALLEKCERLVREPGPVTWNVAITTRGPAVLPLELLPA

>AA314_09024|KEGG|CYP1007C2|[Archangium gephyra]|age|DP

MIATTPGLHAVELAGPRTTPLLGVTGNYIRFVADQLTWLEKLSSYGPLVPLARGNTQHLF
IFHPDYNMQVLSNPALFRALELWRTGPEDSSLRRLWTGLVSINGEVHRQHRRMMMPSFHK
KKVESYRDAMVACIGRMLSGWKPGEVRDIFLEMRQVTLLIVTQVLFGLEDDRETLRMGML

MAEWLERYASPAVHLLPWDKPGLPFRRLMNVSERGEAAIRALIARKRAQGADGDDVLSML
LHARDEEGGSMSDAELVGQTNILFVAGHETTTNSLTWTLFLLDQHPEVHAALVEELEGEL
KGAPPTIEQLERLPLLDRVVKESMRLMPPVTTAFRTSTAPFEMGGHRFEAETQLYYSPYI
THRLPELYPEPKRFLPERWLKAEPPVYGYIPFSNGPRRCIGSTLALLEMKLLLSMLLPRF
RLSLPPGTRVEPMVRATMRPKGGLPMRIHTQDKHFERPEVRGRIRDLVALS

>AA314_09111|KEGG|CYP107DP|[Archangium gephyra]|age|DP

MSTSTNRHDLWAPEARANPLPVYARMRQEAPLVRLFDPYYKVPIWMATRYKDAVELIRDP
RFTKDMRKLPDSSPSKQLRSGESDVFNQHMLSVDPPDHTRLRTLVSKAFTPRRVEELRPR
ITAISHRLLDAVQTSGSMDLLDAYAFPLPVTVIAEMLGVPQEDQDQFREWTNIVINPPLD
GDLQNVRKAGMQFAQYFQGMMAKRRAEPRDDLLSALLAVEEQGDRLSPMELTSMLFLLLV
AGHETTVNLIGNGVWALLNHPEQLERLRANPALIESAVEEMLRFRGPVETSTQRWATQEL
EFRGQVIPAGETVVASLLAADHDPEHFAEPERFDIAREPNRHIAFGFGIHFCLGAPLARL
EGTIAINLLLERMPRLRFAEDPARLRWRDGILVNGLQRLPVAF

>AA314_09271|KEGG|CYP264F1|[Archangium gephyra]|age|DP

MSGRLNLVLPEVLSNPYPHFARLRRDSPVCQVEPGGLWAVTRYADVMHVLKNPQVFSSAG
LQRSMLPSWLEHNPIAHSLLVMDAPQHGRARALVSRAFSTSMLTRLEPALRTTAEQLVSA
LVAQRTVDFLPALALPLPAAAVGLLLGLDPSLFPRFKRWSDDLGAVSATSESDHEGQKRI
RHTIQEMEHYLREVVAERSRAPREDLVSALLQARVDGEALNDAELMAFLFLLLVAGFETT
VNLLGNGAVLLADQPGLLERLRAEPALVPAFIEEVLRYESPAQSTFRLTLEEVELGGVRL
PQHSVLILLLGSASRDEAYVPEPERFILERPQRGNLAFGHGAHFCLGAALTRMEARVALE
ALLPRIQRLSRGPEPLQWTPSIQLRGLSRLPVEVTPA

>AA314_09346|KEGG|CYP264C1|[Archangium gephyra]|age|DP

MSQRLNLLSPEFRENPYPFYARLRGESPVCQVDPHGFWAVSRNEDIVTVFKDPELFSSSE
MKIATQPPWLGRRNPVSEALNLAAPPQHARLRGLVSRAFTQSMVSRMEPFAREVTSRLVD
GLPRGEPVDFLSGFALGLPANIMALMMGVDPSLQKEFKRWSDDISGVVVVAPDDVVRQAE
VRKTLDDMEAYILALIDRRRANPGTEDLVTDLLRVQENGERLTDAEVVSFLVLLLVAGLE
TTTYLLSHMALILARNPEWFERLRGNEALIKAFIEEVLRYEPPNHAQIRVTTRETTLGGV
KLPAGAMVLLLLASGLRDEKSFPDAERFNPERGVQANLAFGHGIHFCLGAPLARMEARVA
LGALLASFSRFELATDRVRWIQSLFFRAPESLPLRFIPL

>AA314_09827|KEGG|CYP242B1|[Archangium gephyra]|age|DP

MSFAETYDFFSPEAVWNSRAFLHRMRTEDPVYWSAQFRGWVLTRYTDVLAAARDRRLVSP
PATGWLDRLPAELKPRFQPARDALRFWAGLSGEQDHLDFQRALKKYFTPAQMDRLRPRVQ
RLTSTLLAETRDSEVLEVVEELARPLSASVIGELLGLPVEDRALLLRWSTDINSFFQHAD
LESLHRGQRSLLEMQDYMRPLIEERRRAPREDLVSVLVSQQEGFFAREPEAVVANCVMLL
FSGHETTSRLISSGLLLLLLEHSGQQALLRERPELMPSAIEEMLRWEGPTSGMTRVSREPL
ELGGRRFGAGETFVLVYRAASHDPEAFPEPDTFDITRQPNRHLSFGMGAFACLGSALTRM
EAEVCFQALLEHLPGLRAAFETPDWVPLPPLNRRLRSLRVTGRRKA

>AA314_10088|KEGG|CYP1315B1|[Archangium gephyra]|age|DP

MVRWLTGAGMQRAVFGFMRRHHPNLMWGKRALVTRAEDVNEVLKTDAVFGVTEIYAERME
RTTGPFFLGMENTPQYQREATLSRRAVRPEDLEHIRSMVREHAEQLIALARPAGRLDVVN
GLARPVAVGLVDRFFGVPGPDPETMKRWMRIIFWDIFLNLGNDADVTARAKAASAELKTY
MEGLIAQRRAGLATESRDDFLTRLLRMQQDEATRLDDDAVRRNVGGIIVGAVDTSSKALA
QLVNELLEHPEALGEAQGAARAGDTARVAAHAFEALRFDPHNPLLMRVCHEDYVLARGTD
REMRIPKGTLVIASTISAMFDPEVMQHPDEFRVDRPPPGYMHFGRGLHTCYGERINHIVL
PEVLGCLLRLRNLRRASGSEGRMRFEGPFPDRLVVEFDAG

>JQX13_05850|KEGG|CYP107DP|[Archangium violaceum]|avm|DP

MRQEAPLVRLFDPNLNIPVWVATRYKDVVDLVRDPRFTKDMRKLPDGSPSKLRRIDSMEV
INRHMLSSDPPDHTRLRTIVSKAFTPRRVEELRPRVTAITHELLDAARARGEVDLLDAFA
FPLPITVIAELLGVPVEDRDQFREWTTTIITPPPNGDFEPLQKAGMQFIQYFQGLLARRR
ADPRDDLLTALMSAEEQGDRLSPQELISMLFLLLVAGHETTVNLIGNGVWALLRNPEQLE
RLRANPALIESAVEEMLRYRSPVETTTQRWATQDIEFRGQVIPAGESVMGSLLAANHDPE
QFPEPEKFDITREPNRHVAFGFGIHFCLGAPLARLEGAIAINLLLERMPRLRFAVEPSAL
RWRDGILVHGLQRLPVAF

>JQX13_06265|KEGG|CYP197AA2|[Archangium violaceum]|avm|DP

MSTSRAQLPPGPRPLPVLGSVLDYAKEPLAFIERCARGYGDVVYVNFLSGPTYVLQHPEH
IEHVLVTRHRHYVKDKLQQRILGGRLLGKGLLTNEGDSWLRQRRLMQPAFHRQRLAAYGQ
VMVGHARRQLASWRDGEVHDVYADMMRLTLGIVIKSLFDLEAEGEAGAVGPALARVMEHF
ARLQSVVFPDWLPTPENLGYREAVERLNVFVSHLIQRRREAGGDAGDLLSLLLHVQDEEG
GRMSDQQIRDEVLTLALAGHETTSITLAFCFHLLARNPEAEAALHRELDSVLGGREPTVE
DLPALPFTECVVKESLRLYPPAWSLSREALEEDEVGGWRIPAGAVVVMNPWTVHRDARFF
EEPEAFRPQRWAGGLEQRLPRFAWFPFGGGPRLCIGSGFAMMEARLVLATLAQRFRFERV
PGDAVELLPSITLRPKHGVKVRLRAR

>JQX13_10145|KEGG|**CYP253D4**|[Archangium violaceum]|avm|DP

MTSPAPSPTTPRIATGPRGLPLIGVVRDVRKDVLGWLTRTAAEHGPVAQYRFGPGRAYLV
THPDGLKHVLQDHVKNYTKDHYSYAMFRRIVGDGLLTSQGETWLKQRRLAQPAFHRARIS
AMAGQMVRATVELSEQWATSERTGEPRHAVRDMMALTLRIVGEALLGTDVRAETEAVSAA
FNVISEQTVERFRSLRLIPPVLPTAYDRAFRRANHALREVVARVIAKRRERMEDQGDLLS
MFMLARDEETGEQMDDEHLQSEVLTMMLAGHETTATALSWAWALLHQNPDAEKKLHEELD
AVLGGRPPTAEDIPRLVYTRRVLDETLRLYPPVYILSRKVMEDDTVCGYQILAGSSLDIS
PYVTQRLPELWPDPERFDPDRFTPEKIAARPRYAYLPFLGGPRQCIGNNFALMEGTLILA
TLAQRHRPRMVDGYTPTPEPLITLRPSGELPVIITAR

>JQX13_10185|KEGG|**CYP120G5**|[Archangium violaceum]|avm|DP

MISPHGLFPQRPGDIDMTLQVDASSPSPENLPLPPGKSGLPLLGETLEFLRSSRAFIQRR
QSQYGPVFRSHVLGSPTVFLLGPEAIQWIFAGEGKYLKNRWTPGVRQLLGAHCLSMIEGE
EHLERRRLLAPHFSYATMRGFVPTIEAIATRHFERWAALPGDFTLWPAMRELAFEIALAL
IFGQDTVDVPFLMRHFKSWTAGLFVPVTVNLPWTTFGRALAAKKAMVGYLEQVVSERQSR
TEQPPDLLGSLIQSRDEAGNPLTREVIVDELQLLLFAGHDTTVTATSNLLLMLAQYPDVL
QRGREAVAGLQGPLTLDGLRAMPYLVQLLNEGMRLIPPIGGAFRVTTRDVAYNGYRIPKG
WMIPVSIKSAHGGSTWSAPERFDPDRFGSERNEQKKPGTFIPFGGGPRICLGQHFAMVEM
SVMLALLLKHYTWELVPGQDLEYVQVPFPHPKSGIQLRVRRLG

>JQX13_10655|KEGG|**CYP264D11**|[Archangium violaceum]|avm|DP

MSGRINLLAPEVRAHPYAVYAELRRNSPVCQVDPGGYWVVTRHDDVVTAFKNPQIFSNVG
MRVATKPEWLGHNPFSDSMIGQDPPTHTRLRNLVNRTFGPAALARLEVRVRQYAESIVAR
IPEEQLVDFVDAFTLPLPASVIGELIGLEPSLHARCKRWADDLTSISANPQDEKRREEIR
ATVREVETEMAAVLDRRRREPREDLVTDLLQARVDGESPGDAELMSFMFLLLVAGLETTI
HLLGHCMRVLMERPDVLARLRADRSLLPRFIEEVLRYEPPVQAMGRLTTAETELSGVRIP
AGARVMLLIGSANHDDTRFPDADSFNMDREGVNNLPFGHGIHFCLGAPLARMEARIGLDV
LLSRFAGFTSGGPVEWNHSFTVRGPRVLPVVAHTQP

>JQX13_12475|KEGG|**CYP110R5**|[Archangium violaceum]|avm|DP

MPPPQLPPGPRQLKPLQTFRFLKNATAFLSECRSRYGDPFTAALPVGDVVVTGDPEGVRD

IFSADPALFEPLGHLILAPAVGDNSLLLMGGQRHKRERKLLMPPFHGERMRAYGLLMRDI
TLRAAEGLRPGSTLRAQDVTQAISLDVIIRAVFGIEEPERMRRFREALSVYMESYTPLLM
AAKPLRRSFGGLGPWARFQRHVAELDRMLSEELATRRGHEEGHEDILSLLLGARDEEGQP
MTDAELKDELRTLLLAGHETTAIGLAWALHHLHHHPDTLERLLKELAPLGAVPEPEALAK
LPYLGAVCDESLRLHPIIPVVGRRTVAPFTLRGRELPAGTGIMAAICVVHRDPVLYPEPE
RFRPERFLERKFSPFEYLPFGGGARRCIGAAFALYEMRIALGTLLAAHRFALASGEQERP
VRRNVTHAPENGAELVYQGPRHGALA

>JQX13_12730|KEGG|CYP1347E2|[Archangium violaceum]|avm|DP

MASAARTLQLNPVAPENLLDPVPLYRELRSNDPVHWSEAVQSWLITRHDDVVNCFRDPRL
SANRVRFYEDQLRGLGPDLIKDFLKGLNDQMSQRDGREHIQMRRQAAAGFSPQALDRWRP
AIRRTAEQLVEQVRGQGHMNAVESLASQLPPRVIAEVLGIPVQDREQFLEWARPIADFNA
PAAGTDVVDLARRTNTATRELFAYLRRIIEERSHSPGEDLLSQMLQSQQAGQMTLDQLVS
NASLILFAGHTTTTDQISNGLHDLLSHRDQFHKLQEDRTLVRSAVEEMLRYNPAVPFMHR
IAAETFQLRGKTIRKGDVVFMGLAAANRDPEAFPDPDRFDITRDSNHQKHMSFTFGPHHC
LGAGLARRMLEITFEVLLERLPELHLDPKQLPQLKCHSLMFRGYDSLTVRW

>JQX13_13845|KEGG|CYP110AV1|[Archangium violaceum]|avm|DP

MSLPPGPRAPAVVQTLEFLKDPLGFLRRCYARYGDVFTLREIGFGTEVMVSSPALLKELF
TGPPEVLHAGEANERIFGSITGTQSSFILEEQAHLRRRRLLLPSFHGDRMHEYGDTMRRQ
TEKAVASWPLREEFALHPEMQSITLHVILHAVFGLGQAANDSTLEHLLTRTANEAVASML
LILPPLQRDLGRWSPWGRLLHLIHQMDEALLAEIARRRREGAKPEQRDILSLLLQARYEN
GEGLSDGELHDELVTLLMAGHETTGTSLTWAMECILSHPEVEQRLRAELDDVLGDEQLGH
EHLTRLEYLDAVIKESIRYRPIMPTGGARLVKRPFSLGGYELPPGVWVINALSVVHHRSD
LYPEPDVFRPERFLGRRVDPYEWTPFGGGVRRCLGMAFALYEMKVVLATVLRSARLELAR
SPVKARRRGFFIAPENGLPVRVLSRLRRAGDVPGTQEVATGGAPARSEDTMGWGA

>JQX13_14855|KEGG|CYP1069J1|[Archangium violaceum]|avm|DP

MTPSQLGLEFTPLEPHFLNDPFPFYARLRREAPVTFAPAFHFWVVSRYADVMTALKDARH
FSSRDTLRPPVALPREVMAILEPAGYRADYPLLGDDPPAHTRLRALVGKAFNQARVNALE
PRLRQLVGAHLDTLRDGEPRAELISQLISPLAMAVMTELLGLPTSSGKRIKQWCDDEKLF
FVPIPLDQHLRAARGVADFRRYLRELVEDRRKSPRDDLISSLLQARTEGDRPLDTEELVA
LTCVLVFAGHETSTNLLATALHHLLRHPGVWGELRKDTYLVRNAIEESLRYDSPVVGMMR

TTTEPLQLGGTSLPEGARLFLLFASANRDESVFEQGERFNIHRASAVRHLGFGHGLHYCV
GASLGRLEAQLTLEALLERLPGLRLAPGEPVTYLPNLVHRVPRQLLVEWDA

>JQX13_15660|KEGG|CYP197F4|[Archangium violaceum]|avm|DP

MTTLHGEPPGPEGAWGIANVADFHADPLRFVTRCAREFGDVVRLGQDNYLLCHPRDIEHV
FVNSQRSFAKETGQKPTWAKTGSYHYALMHTDGKDWLYKRRLLQSLFKRDRVDTRAEPIV
EAAEEMIASWRPGKVRALEGDVAPLAAQVVGRFLLGSDLSANEVRRVTSFLAWSFRPLPI
RIPRWLPTPQNLRFRWSVWQFDRAVYGLIERLRERSRGSSNLLELVHQARDGEDGCLAPP
WYPRDEVAIMLLAAHQPTAIALAWTLYLMALHPEVDARVAAEVEQTLGGRRATVEDSDRL
VYTAAVIKESLRLYPPVWMTARDSLQEGELGGYRIAAGVSLALSPWVTHRDPRWFVDPEA
FRPERWLDGSLERLPKFAYFPFGGGPRLCMGASLAKLALVLVIATVTRRYRLTLAAGAKV
SPFAAASFLIPQGIRLVPTART

>JQX13_15775|KEGG|CYP107DL9|[Archangium violaceum]|avm|DP

MTTNRVEAQKEPVTAAPEPGGSCPMKALREAPAVDSVLLDRESPEFMARAHAIYAELRDT
APVVRCPFGRGLADGAHPVGSQARASGKVPGRDRLFVTRYDETVAALLDDRLSSDFRSAM
TPEQRARVESSMPEEFRPVAYSILMLDPPDHTRLRKLVQPNFTARAMEMLRPRIQKLVND
LLDTAERKAAERGEAVSERRMDFVESFAYPLPVTVISDMLGIPMEDREQVHGWAEMLLKT
DRQEAMADQKVRGSLRAFSQYLEGLFERKRREPAEDMISQMVHVQEDGDTLSHQEMVSMV
FILFFAGHLTTVNLIGNGVVALLTHPEQHALFMANPAGLAKGLVEETLRYWGPVDYISTP
RIAQADLELGGTHIPKGENLSLGLASANRDPARFANPDVFDITRPDAHRNIAFGKGIHVC
IGAPLARLEAQIAFELLFQRFPKLRLAVPAEEMKWGSVASMRGFNRIPVLF

>JQX13_16055|KEGG|CYP2528A2|[Archangium violaceum]|avm|DP

MPLPPLMGQGLPLLGHFPYIHGLGVDSFAFFMEGYRQHGRVFAVRDMKDTFAVLLGPEAN
RFVLERETPHLSWGAAYGDEGSRVMGPTAMPLLDGEEQRRRRAPMMSAFRHQNITAFGDA
MVELARAQTAGWKEGRPVDLIGEMSELTFRVACGFLLGVEIGEDYPRFRELYKKMFTPVG
LALQAAGLPYMQRYRDGLRRLSARWIADRRAHPRDDIISRLLEAGLDDEDVISQVLVLMF
AGHDTTKLSLTWTLALLLRHPEYLARVLAEQDEVLGDGPIDAEATRKLPVLERAIREAHR
LYPPASLLRRGVLEGFELGGYTIPKGWKVMYLPRVSHHLPDVFKDPERFDPDRFAPPREE
HKQPYAMVEFGGGYRTCIGKELAMFEAKVVLSYLLRTFRFELAPGQRLDVANSGAMTSPR
HGVKVFAKPTGRRVPLARAA

>JQX13_18665|KEGG|CYP1284B6|[Archangium violaceum]|avm|DP

MSSFSGKKLDKIPAHVPPELVYEYDNARDPRMLEDPHARMRSLILEAPPIFFTPYNGGNW
VVTRKKAIVDITMNPEVYSNAFLVSGHAEEHSGEHKDSQPGLMMLPIAADPPQHTMYRAP
LNQPLSAKSVSGLETAIRDMTNELIDKVLAAGRCDFLPDIAEPLPVTLFMKLAGMPTNRL
AEFRHLATQATSSTVDPATRAGVFKSIAGILAETIKARQEKREDDLISHLLDANLNGRNP
NFHEMLGYSIALFLGGLETVVNALSFGVRHLARDQELQAKLRADHSLIPGAIEELLRLYG
IASTVRQVMRDEVCHGVQFKKGDTVSLLLPAANYDDEAFPNPEQFIIGRKEQHQTFNTGP
HRCVGLNLARLEMKVFYQEWLKRVPPFRLDPEKKPQFVGGFNLGITSLPLVW

>JQX13_18675|KEGG|CYP1284B7|[Archangium violaceum]|avm|DP

MHADSGSQKLDKIPAHVPPELVYEYDISRDPRMLEDPHARMRSLIHEAPPIFFSPCNGGQ
WMVTRKKAIMDMTLNTEVFSSRSTGTGENPGEHKDPTGGLTLLPISVDPPQHTMYRTPLN
QPLSAKSVARLEPAIRDMTNELIDKVLTAGRCDFLPDIAEPLPVTLFMKLAGMPTNRLAE
FRHLAAQAASPTVDPATRAATFKHIAGILAETIKARQEKREDDLISHLLDANLNGRNPDF
HEMLGYSILLFLGGLDTVVNALCFGVRHLARDQELQAKLRADPSLIPGAIEELLRLYGIV
CMPRHVARDEVYQGVQFKKGDTVLLLLPAANYDEAAFANPEQFILGRKEQHQTFNTGPHR
CVGLNLARLEMKVFYQEWLKRVPPFRLDPEKKPRFIGGYTLALTSLPLVW

>JQX13_18835|KEGG|CYP253G6|[Archangium violaceum]|avm|DP

MTTPAPLPPMPPGHWLWGHLPERARDPLGLYLKSRERLGDVVRYRMGYIFVEQLTHPDHV
KHVLADANARYTKGTVFDKTRPLVGNGLLTADGDFWKRQRRLAQPAFHRERLAALGARMT
DTITETLQTWEPAVASGRPLPVFQEMMKLTLTVAVRSLFGVDVGEHTRAVGDAFTTALSV
TNERIISPLPYLPWLYRLPSRDNRAFREAVDTLDSIVRGIISQRRVKGPGAGADEDLLGM
LMAASDADTGDTFDDAQLRDEVMTLLLAGHETTATALAWTFHLLEQNPEVEATLHAEVDA
ALGGRVPTLADLPKLRYVGCVFEEAMRLYPPVWAIPRVPLEDDVVDGYRIPKGDIVILVP
YVTHRHPDFWPEPERFDPTRFLPENSKDRPRWAYLPFGGGQRQCIGNNFAMMEAQFVLAL
VAQRFRLRGVPGVPVEPEPHVSLRPRGPMPMHVERREDRSQVRARTA

>JQX13_19770|KEGG|CYP1263C1|[Archangium violaceum]|avm|DP

MTLPQSPVAAVTHPDPYPYYARLVADRPLHHDRELGLWVASSAEAVTAVLSHDACRVRPV
SEPVPRALLGSPAADIFRLLVRMNDGASHCPLKQAVSATLGTVDEARALALGRKWARRLF
EELAPASLPGGLTDFAFRLSVHGVGELLGMPEAHLPRVADWMSDFVRCLAPASSPEQVER
GRVAAGQLLGLFREWLFARHALPAESLLSTLARELGRRSGGLDVGEAVAANGIGLMSQAY
EAGAGLFGNTLRVLAAHPEWRERVVANSGLLGDVLREVLRYDPPIQNTRRFVVRDDVVAG
GRMRAGDVILVVLAAANRDPRVHPSPERFDPFRPERETRSFGAGVHACPGEALALALAKA

GVERVLMSRMPFESLAGAVTYRASANARIPLFEPGSARL

>JQX13_21720|KEGG|CYP264D12|[Archangium violaceum]|avm|DP

MSGRPNLMTPELKANPFHLYAELRRNAPVSQVDPGGMWAVARYEEGMFVLKNPRLFSSAG
FGLATNPPWLGGNPASRSISAMDPPQHGRLRTLINPAFTSAAVNRMEPRVRAFAREVVAR
LPLGSPVDMVPAFALPVPAFVLGNLLGLDASLHSQLKHWADQLTSVTAIRPDETERQEPV
RRAVADVKRYFGEVVEHRRREPGEDLVSDLLRARVEGEALTDDEIMAFLFLMLLGGLETT
VQLLGLCVLALMEHPDVMARVRADRSLIPRFVEEVLRTGAPGHGLLRVTTEEVELGGVRL
PKGAPVVVLLGSLCRDEALFPNGDRFDIDRPASQLPFGHGPHFCIGALLARMEARLAVEA
LLERCGGVSPGSEPVVWHRSMVVRGPSSIPAVLHP

>JQX13_22375|KEGG|CYP1496B2|[Archangium violaceum]|avm|DP

MSASADIDLMSESFFANPFPTFERLRTQAPVYFFEPFQCFILTRGADIEAVTKNPSFSSR
RANEMLGSLGVLREDAASEEMLATWSRLVFFQDPPRHTLLRQLVMKGFTPAALERFRPRL
ASLVERTLEKVRRQGELDVVADFAEPIAINAIAELFALPEADRPRFMRWSKDLLKPTSSA
VRTDEVRNSVRRTSHDMVTYLRDLVEKRRAAPGDDLISQFIAGEEGNLQLAGEAVIQSFQ
MIGAGFVTSTNQLTNTVLALLKHPEQLRELRGDPGLIRGAIEESLRHEPAILSINRLCVQ
DTEVGGTRIPQGRFVHAMTAAANRDPEVFPDPDRFDITRAPNRHVTFGVGAHYCPGASLV
RLEVEEALRALLTFPRWELADRPYNYQGSNFQDRGPGSLHVRFPSN

>JQX13_22385|KEGG|CYP264A9|[Archangium violaceum]|avm|DP

MTTQRVNILAPEFRADPHPRYAEMRRNTPVIQVEPAGFWAVSRYEDVAFVIKNPQLFSSQ
GFKAAWQPEWVGYNPLANSMLAMDGAGHTRLRTLVSRAFNASAITRLEARIRKLANRLVD
ELAEKGEADFVSRFAMPLPAFVIGELLGLDVSLHHRFKDWSDDIASVTPEPRDPEHARRT
LIAISDATRYISEVIEARRRSPADDLVSELIRAEVEGQSLTDREIIDFLVLLLIAGLETT
VHLLANSLLFLAERPDVRARLRAEPALVPGFIEEMLRYDTPVQALVRIVTSDVTLSGVKI
PQGDVVLALIASANRDERHYTEPDRFDLHRGQPGLSFGYGVHYCIGAQLARMEARCGLEA
LLSRFSGFERIPAELNWGQAITVRGPQRLPLRFIPA

>JQX13_24835|KEGG|CYP107DP9|[Archangium violaceum]|avm|DP

MTASPLFAEGETLWSPHVRANPFPLYKRMREDFPVARITDPQLGQHFWLLTRYEDVVSMV
RDPRFTQDPTRLPEEVRQRFFGHKMRSLSRHLLTVDPPDHTRMRSLVSKAFTPRRIEELR
PRIREICSELLEGLMAHGSGGDFLKLFAFPLPVIVIAELLGIPPEDRDRFREWTQTFFAP
PSKDSQEKAREAYQRFFKYLGELFEQRRQQPRDDLISALLAVQEQGDRLSSEELTSMVFL
LLVGGYETTGHLLGNGLLALLQHPDQLQRLREDRSLIPSAVEEMLRYCGPFELSILRFAK
EDLELHGQRIQAHEAIRVNYLAADRDAEQFSEPDRFDVGRTPNKHVGFGHGIHFCLGAPL
ARLEASIAFELLLERLPELSSATAPEALAWRPSAQARGLVSLPLAF

>JQX13_25725|KEGG|CYP102M17|[Archangium violaceum]|avm|DP

MRAVSASAPIPQPRTRPLVGNIPDVESETPIQSLMQLSREYGPIFRLTFPGRSILVLSSH
ALVDEVCDERRFDKSVGGALKNIRDFAGDGLFTAYTNEPNWERAHRILMPAFGPQAMRGY
FDFMLDIAEQMLTKWERLGPDADLDVTDNMTRLTLDTIALCGFGYRFNSFYQREMHPFVD
AMVRSLAEAGERARRLPLKTRLMLMTQQQYQRDIAYMHQVVDEVIRERRHNGDAATRKDL
LSLMLQGVDPVSGQQLDDQNIRNQVVTFLIAGHETTSGLLSFTLYQLLQHPDVLARATAE
VDRVLGRDLSKRPTVEVLSKLTYLDQVLRESLRLWPTAPAFGLYAREDTVLGGAWPVRKG
EPIMVLTPMLHRDPEVWTEPERFDPERFSPEAVAARPPNAWKPFGNGQRACIGRPFAMQE
ALLVLGMLLQRFQLIDHTRYQLHIKETLTLKPEGFRMRVRARGDADRPVVELRPAAAAVA
PQKPKWTSGPVAQHGTPLLVLYGSNSGSSEAFAQRIASDAVVQGYVPTLGTMDAYAGRLP
HEGAVVIVTASYNGNPPDNARAFCRWLEELQPGALSGVRYAVFGCGNRDWAATFQAVPRH
VDERLAAAGAQRLLARGEADARADFFGDFDGWYQGVWPRLGESFGVETAEANQAPRYEVE
RLEQAADPLELAHGVVPVEVVANRELVDMTSPFGRSKRHLEVRLPEGVSYRTGDHLAVLP
ENAPEQVERVARRFKLSPDETVLIRRTREEPGTLPFDRPVTVRALLGRYVELSAPATRRA
LQLLAKYTACPPEKKRLLALAGEGGAEPEVYRTQVLEKRLSVIDLLEEFQACELPFGVFL
ELMPPMKPRRYSISSSPLEAPDRCTLTVAVVDAPAWSGRGRFQGACSSYLTRVEPGTRIH
AVLREPHAPFRPPEDPSVPVIMVGAGTGLAPFRGFIQERARLAERGTRLGRALLFFGCDH
PDVDFLYREELEAWQRQGVVEVYPAFFRQERDGVTFVQHRLWEERERVGVLLDAGARLYV
CGDGRYMAPAVRETVARIHQERTGCTAEQATAWVRELETQGRYLADVFGG

>JQX13_28490|KEGG|CYP264D14|[Archangium violaceum]|avm|DP

MTVRLNLFAPEFRANPYPFFAELRRQPTLSNVDPLGFWAVSRYADVLHVLKNPQLFSSTG
NRAPAEPEWLGRSNPFADSMVMVDPPRHTRLRALVNRSFGPTALSRMEPRIRAYAEQAAA
ELTLGRPVDFVESFALRVPAAVIGELLGLDASLHPRFKRWADDINNTSSTPPDAHEWHAQ
IRGTYAEMEQYLKEVIAERRRAPREDMISDLLAARIEGEALTDAELLGFLFLLLIAGLET
TVHLLGHAALVLAERPDVFARVRADRSLIPRFIEEVLRYEPVAQTLLRLTTAETELGGVR
LPAGSHVMLLLGSACRDEAQFPNSESFDLDREGSQSMPFGHGIHFCLGAPLARMEARLAL
NALLDRCGGLVRDAAPVQWHASIVVRGPTVLPLTVLPG

>JQX13_28950|KEGG|CYP267A4|[Archangium violaceum]|avm|DP

MPMEVADPYGLLSPERRQNPYPFYARLLREAPVHFSEPWEAWVVTRHADVNAGFRDMRLS
SVRTGMFSQGMPEEVLRKLEPMGRNMASWLLFHDAPSHTRLRSLINKAFTPRMVEALRPR
IQALVEELLDAARGKERMEVISELANPLPVIVIGEMLGLPREDRYRLKKWSDKLASLIGT
GRPTLAEVEGALGAILEFEDYFRPLLARRRTQPGNDLLSALVQAEEQGNLMSEQEVLSTC
TLVLFAGHETTTNLIGNGLLALLRHPDQWELLRGAPELLPDAVEELLRYDSPVQFTNRVA
AVDLEFGGHTFRKGDRVILMIAAANRDPAHFPEPDRLDVRREQLRHQGFGMGPHYCVGAA
LARVEAQETFAALLRRFPRMQVAPGVLPEFVDNVGFRGLQSLPVVLEPAA

>JQX13_30100|KEGG|CYP264D13|[Archangium violaceum]|avm|DP

MSNRVNLLAPEVRANPYPVYAELRRNAPVSQVDPDGLWVVARQADVLTVFKNPQHFSSDG
IRQAYRPAWLPDYPLADSMLVMDPPHHTRLRAIINRAFGTSVVARLEPRIREVAHQVVAS
LPVGRSVNFVDAFSIWVPIAVLGEMLSLPQSLHPRIKHWAEVYGEFTSIGENDTERQKTI
LETVAETRFHFNQVLEDRRRTPRDDLVSELLRTHVNGEELSYTDLMGFLFLLLIGALETA
VHLLSSCALMLQEQPELMARLRAAPTLIPRFIDEMLRHSGPAHGLFRLNTDEVELGGVRI
PRGARMLLLIASANRDEAAIPDPDRFDMDRPGPQNLPFGHGIHFCLGAQLARMEVRVALE
ALLARFARLTPGDGPIQWNASLVMRGLTQLPLVAHLP

>JQX13_31565|KEGG|CYP1248B6|[Archangium violaceum]|avm|DP

MLDHNAPAPDDVSADFNPLAPDQIDNPYPLYARLRRERPVFHCPMFDLWVVTRYADISEV
ELDTARFSSVGALDARAEPHPEVRKVLEQGYSQFLSVVQSDPPDHTRVRAVFGKALSAHR
IAAMEPAIRATADSLIDGFIRDGQADLIQQFAYALPGFIICDLLGVPRSDMEQLKRWSDD
KTALMSATAPIEQLVQCAHGFIAMERYFKEQLHERRRHPREDLLTLLLPQSLGGTAPMSE
QEAVCNAMDLFAAGHETTTGLIGNGMWLLFNAPDQLEALREDPSLLPNALEEMLRMEAPI
RGFFRTVMADSTLGGVMLPKGARAFILYASGNRDETQFTEPDRFDIRRADAKKHLAFGKG
IHHCIGAPLAKLEGRIAFEQLLRRLPGLRLRTDVAPVLRPYFMLRGFEHLPITWDVPA

>JQX13_32710|KEGG|CYP1043A11|[Archangium violaceum]|avm|DP

MPDVLNLRPVANTLASRLPVALIGLRNRIFTRINGEEGIPIPGERVGVSHFKELYSNPAA
DGRSEGAVLSDLFWYWLSPGAEIHQEHLEPGERYEEVAQATRRILSLPKKTAEELAARCT

VRVLEERAMHRATLVRLRDLMMPIWAEFYYELVFNERCPREARDLIVDNADDVVTALKGL
SLRHMDRRLRLTRYLKARLEAGAVAHELPSCLSLEQKAFYLQGVYFNTAVVQMSEAMAHL
VMFLAQHQEVQTRLADNLDDDRYLDRIITESLRLYPLFGIAHRITSSDIRVDDKTTLPKG
SVLCFNYQAYHHEGFEDPLRFDPDRWLKHSTKESNYMPFGAAANRPCPAQAIALVTMRAV
ARETIRRFRAFSSASHTRPLPNRGPCVLEPRKENPDPRAREELLASMSAREPWEEVGRSV
LQLVLGTYMVLDAKRLRLCQRYFEAERQASTTGAKASRCPFSHS

>JQX13_34720|KEGG|CYP242A7|[Archangium violaceum]|avm|DP

MKPAPEIAMLNANVVTSPRYEFFSPEAIANSQSLLSRIRSEDPVHFSDQLGGWLVTRYAD
VMAALKDPRFLQATGTGRLDAMPEAARQQLRPLRDSIRLWMGNDSTEDHLRFQRILKKYF
TPGTLEALRPRIQELTTELVDAARARGRAEVVSELAYPLPANVIAEMLGVPTKDRELLQR
WSRDLLPIFTASSMEQLLQSQKSVLEMTDYMRPIVEEHRREPRQDLISVFLAEEAAGNIR
DVEEIAANCVLLLFAGHETTANLICNGLSALFDFPDQLELLRAKPELMQSAVEEMLRFSG
IGGTITRVAGTDLELGGKKIAPRQLVFVSLASANHDPEVFPEPSRFDITRKTNKHVTFGY
GSYYCLGAALARLEAQVFFSTFLPRFPNLRLVDRAWEPSPPLGRRLTALNVSF

>JQX13_34870|KEGG|CYP1329C1|[Archangium violaceum]|avm|DP

MPKALPRLQEPPLIGSLRSFRNERLELFNRVRRECGELGLFRVGPLSIMLVNSSRALQEV
TSLHADAFEGGLAVNALLPIFGRYSLVLLPNKEHRTYRKIMAPTFQPRRVSMFAEPMVKA
TLEAQARWTDGQEIDVGQEMLRLTMRIVGKTLLGLEVLDEADDFGAALTTCLEYSNHLVA
NIVPVPLWLPTKRNRELKKALAFIRGMLMEKLSSRRHAGCPATQDFASMLMGVRGEDGQG
LSDEQLRDNLIFIFSAGYETSANTLAWTWYLLSKHPEVYERLRKEVDEVLQGRAPTYEDL
ASLPYVHQVVKEVMRIYPVAYFFGREARRDVEIDGYQVPKGTFAAVCPYTLHRNPEYFPD
PERFDPDRFAPEQESKLPKYAYLPFGAGVHACLGVHFFMIEAPLLLATLVQRVHIDVLPG
QTVKPVVEVTLRPSRILARVHHRS

>JQX13_36270|KEGG|CYP264K4|[Archangium violaceum]|avm|DP

MRTYNLFDPAVLADPHAVFAQMRSEPGLCRVEPFGAFAAARYEDAAAILKDPQRFSSEAL
AITAEPPWLGPNPVAQALISKDPPNHARLRALVNRAFGPAGMARLEAQVWKVSEELAEAA
VRRREVELVDAFTFVLPRDIIGYLLGLDPSTFSNFKRWSLAMGLITSATTPEQHEEIRFV
VREMKGYLTEVIEARRRQPGEDMVSDLLRAEVDGRKLTDEEILSFLFLLLPAGMETTSQL

LGNAIIYLARHPEHLARAREDKTHIPRFVEEVLRYESPVQLSFRMATQDVELSGTKIPAG
SFVLGLVGSANRDERVFEQPDRFLPGRDKGTQHLTFGYGIHFCLGAQLARMEARLGLEAL
VSRVGAIRLRSPEVQWLPGYTIHGPATLPVELIPA

>JQX13_36820|KEGG|CYP264E1|[Archangium violaceum]|avm|DP

MDQPLHLMTPEARANPYPLYAEFRRRAVCQVEPGGMWAVSRYDDVVTVLKDPRRFSSAGL
GQSFLPPWLERNPTADSLVMKDPPDHTRLRAMVSRAFSGMALSRWESRIRDIAEELAEAA
VRHQEVDFIADFALPLPTRVLNLFFGLEPAMALRLKGWADDLVSIPASQPSPQRMEQIRH
SLAEMERCFNALIELRRADPGEDLVSELIRTEALTHEELLSFLFGLVPAGVETTVYLLAN
TMLVLSEHPRELERVRENPTLIPRLIEEVLRYEPPGQSSLRLTTEDVVLSGVTIPRGSVV
VALIGAAMRDEGRFPQADRFIIDRENQGQLAFGHGPHFCLGAMLARMEARLGMEALFSRI
RGFTLARRDVTWSQSIIARGPLALPLRLEGPLR

>JQX13_38215|KEGG|CYP152E5|[Archangium violaceum]|avm|DP

MGGMPRDSRLDSTLALLSEGYRFIGNGCARHRSDVFETRLLLRPTLCMSGAEAARMFYDS
ERFQRRGAAPLRLRKTLFGMGGVQGLDGEAHRSRKAMFMSLMSPDGIRRLSEQAEKHWRA
SIREWERKGRLVLLDEVQQLLCRVVCEWTGVPLPEAEVIQRTRELTALIDGSGGVGPRHW
RARLMRMRSEAWLGRLVSRVRAGRHAAPEGSALRTVAEYRGPDGKRLGPRLAAVELLNVL
RPTVAVARFVVFEALALHEHPESRAWLLEEEDDDAYEHFVQEVRRYYPFFPFAAARVRRD
FTWKGYHFPRGRRVLLDLYGTNHDVRLWERPHEFRPERFRTWDGSPFSFIPQGGGDHHTG
HRCAGEWSTIALMKVAARMLTRGMRYDVPSQDLRVDLSRIPAEPASRFVIANVRDAREPP
HERAAAASTAGRKAVALPGPESPRGV

>JQX13_38560|KEGG|CYP1224J3|[Archangium violaceum]|avm|DP

MLPPGPALPSPLQMSLYMADPLSFLRKQRKRHGDTFTLRMSGMGEFVFVSAPEDIKRIFN
APPDALHAGEGNAVILPLVGSSSLVMQDGAEHIRLRRLLLPPFQGERLHTYAGPMRETTH
ASLEQWPVGKPFPLLPRMMSLTIEILLRSVFGIEGKEELALFARRFPRLVDSIASPLYLI
PALSGVDVFQKVPWLRASKMKREVDEAIYALIAHRRAQPADPRRQDMLSLLLESRDEAGQ
PLTDRELRDALFTVIVAGYETTAIGMCFTVERLLSTPEALARVHEELERVVGDTELQAEH
LGQLEYLDAVIKETLRLRPVVPLVSRRTKVPFELSTYTLPPETMLVPGIALTHLREDIYP
EPDSFQPERFLGKKPDPYAWLPFGGGSRRCLGMALSLFEMKVVLATMLRRAKLRLASKRP
IHMVRRSIMFAPSRGMPVVLDEVRPRFGQPQKQVA

>JQX13_39850|KEGG|CYP1224J2|[Archangium violaceum]|avm|DP

MPTPLPPGPRLPGPVQTALYLSQPLHYLRQWHQQYGEAFTLRMAGAGNMVFISSPQDIKR
VFNAPMEVVHAGESNSLLRPFLGPHSVVILDEWDHVRQRRLLLPPFQGERMQAYAEVMRE
VTHASLERWPMARPFPLLDVMTELTLELMLRNVFGLETPEEIAAFSRDFSSLVDSTNSPL
RMLPSLIGMDLFTLLPWARASRLKRRVDAAIYALVARRRAEPRRPERTDVLGLLLESTHE
DGKPMSDQELRDALLTLIVAGYETTAIGLSFTVERLLAEPRTLTRMHEELERVVGDDELG
PEHIGQLEYLDATIKEALRVRPLVPLISRQLKGPLELTHYTLPAGVTVLPAIVLTHFRED
LYPEPEHFKPERFLEQKPDPYAWLPFGGGARRCLGMAFAMYEMKVVLATLLWRAKLRLAS
SEPVPMINRHIMLAPGGGVPVVLDEVRARREGPRRTG

>JQX13_41630|KEGG|CYP109N4|[Archangium violaceum]|avm|DP

MQPQALKLTPEMVANPYPVFAALRESAPVHYVEAFRGSYVLSRHEDMAPVLKNTTLFSSK
MASVSALMPKELGEDVLRYFRSENSLLSSDPPQHTRLRTLVSRAFTPRRVADLEPRIREL
TRELLDAMLTRQEFDLMADLAVPLPIIVIAEMLGIEPERRLDFKRWSNAISQSVTLTVGG
LDLEWIATGIREIHAYLEAAIERRRQEPGNDLISALVESNEQQGGFLSSMDVIGFVRLLL
FAGNETTTNLIGNGTLALLNHPAEMERLAEDASLIPNAVEEMLRYDTPAQVIFRITTADT
EIAGTPIPKDSRIMLLLGAANRDPRKFPDPDTFDITRDTQGHVAFGHGVHFCIGGPLARL
EAKVVFEELFRRVRRVSFAPGQEGHLDYGTTFSLRGPKSLRLRAERR

>JQX13_41680|KEGG|CYP1347B6|[Archangium violaceum]|avm|DP

MSSAAPTLDRNLVSPQNMLNPFPLYQYLRENEPVHWSDAVHAWFLTRYDDVMAAYRDPRM
SANRAKLFEYQVQGLSPDITREFMQTVRNQMFMRDGPEHVRLRRQTGMGFTPQRLDELRP
TVHRIMRELLEQLASRHEMDLVKDLAYPLPALAIAELLGVPPEDRDRFWVWSEHLALYSS
PPVGADSQLLASNANKTMVEMKEYLLPIIEHRRQSPGTDGLSQMLLAQEQGKMTPEELVA
NACLILFAGHTTTTDQLSNGVYDLLTHPDQFQMLREDMGLLRSAVEEMLRYTTSVPAITR
IASEDIQMHGKTIRKGDMVFLVMAAANRDPSVFPDPERFDITRDSYHQKHISFGFGAHHC
MGAGLARRELEIGISMLLERLPNLRLDETKPPQVKCHGLSFRGFDSLPVRW

>JQX13_41805|KEGG|CYP109R10|[Archangium violaceum]|avm|DP

MSLLERFDTLDPEVIRNPYPYFAEMREKAPLFWIERLQAHAVSRYEDAAYILKNPALFSS
ADIRIAGKLPSERSSLELGSVSNLLTSDPPIHTRLRGLVSRAFVPKRISEMEPRVRELTK
GLVAEMTAHDEFDLMDGLGSPLPVTIIAEMLGVEPSRRRDFKRWSDSLVESGAEIIRDGK
ASDKNIRLGQEMLAYMTEIAEKRRREPRGDLISMLVQGGEGAEPLTPKEVNSFAILLLAA
GNETTTNLIGNALVALIRNPEAYEWLRRDPTIAACAAVCEETLRYDSPVLGLTRRTTQEV

ELSGGKLPADSTLVVMVASANHDPRKFPNPDRFDPQRDTTGLFSFGHGIHFCLGAPLSRL
EAPVALQELMARAPRLGFAQRQPEHIDYGMSFFLRGPRSLWLRKN

>JQX13_45100|KEGG|CYP167B11|[Archangium violaceum]|avm|DP

MSANPTNPSETRPSVQFNPYAPGFDANPYPALEKLRTEAPLFYWEQGRGWLVSRYEDAVA
VLRDNKRFSPDRAEWEFASVLGSAAMVPELAELNKNGLFALNAQDHARVRKLVSPALTPR
AIERLRPEIQAIVDEILDGVAAKGRLDVVNEFAERIPARVIGSMLKIPKGREELFQDFTN
AVIKNVLPGLVAPEEQELLRRHIREGIALVGETIEDRRRNPLESDILTTLIQTEEQGDKL
NKQELLSLVAALIVGGFETTVHLIAFCVYNLLQRPEVLAQVKTEPELLKNFVEEVLRFDN
FGKLGISRYALEDVELGGVRIKKGQMLLIMLNSALRDESTFDKADVFDIRRNTNASIAFG
HGAHYCIGANLARLEVQIAVGTLVRRFPELRLVKPPSFGPHPVIRRMESLEVDLRSPSA

>JQX13_47220|KEGG|CYP1833B1|[Archangium violaceum]|avm|DP

MPTAVEFEPLHPETLRDPYPVYARMRALAPVMWHERLNSWLLTRHAECLQVLKDSRRFAA
DWRRVGAAVPEQSLSIQSMDPPEHGELRGLLVSAYRAQDLAAVERRAYLHANHLLAGLRA
EGGGELMTRFAAPLALHAVCDFLGVPAPDPATFGEMSDAIIRGMDAGLAPERAAPGAAAR
AGLSALIASWFEPPPTGGMFGFLARSESAAGMPRAVLMNSMRVVFHAGYTSVYSAVGSAI
ISLLRHGMELSRLRDPALLETAVEELFRYDGPVQATGRVCTEDVELGGVRIARGQALVLL
LGSANRDPEAFSQPEALVLDRQPNPHLAFGWGIHACVGGLLAKAVVRLALCSLIEHAPGL
RLSGDVVHKPQATQRCPDRIPIAFGA

>JQX13_47720|KEGG|CYP109N5|[Archangium violaceum]|avm|DP

MQSPPTHPLLNRDVLANPYPVYAQLRESAPVHYIEPPLGMYVICRHEDIGPILKSPAMFS
SKGMELSRRTTSKLSPEVLALVTPENSLIASDPPIHTSLRNMVGRAFTPKRVAELEPRIR
EITVELIDDMLRKDELDLMEDLAAPLPVTVIAEMLGVEPERRREFKRWSDDAISSSNLSL
ADTDPSRLEVGVRALHEYMRQAIEERRRSPRGDLISALVEAGGKEDFVTSNDLVAFCRLL
LIAGNETTTNLIGNGMVALLRNPAEWEKLVADPSLVPNAVEEILRYDSPVQGIFRETTQE
LQVGGQTLPEGSRVMTLFGSANRDPRKFQEPERFDVTREVQGHYSFGYGIHFCLGAPLAR
LEAKVAFEELLRRVRRFEFASSPVESLEWNNNLFLRGPKSLRLKARLR

> JQX13_49065|KEGG|CYP117E4|[Archangium violaceum]|avm|DP

MQALTNLLFQLPQKETHRDGIPILPGGLPLVGHAPFNIGSPLTFLRDAASKVGPLFWMKN
MGGWSLVCMGEPGFELLKNRVTSSEFIREQAPDFIGDSLLSRDGVPHRNLRSAMSGAFTP
RGLTSSGAAAISAEVIEASVAGLPQQGPFSLLAETQKLALDIIFRIMGIDRSELEAWNRN
YRDFVLGALPVRLDLPFSPARRASRGRRWLDARLADLISAARGKPDMKGTLAALLTARDE

EGNALGDSDLIQNLRLLALAGHETSASVMAWLGIVLAQRPDLWEKLRDESMAAPGLPRSP
DELKRHPFAEALFREVLRMYPPVAFTAREATEDLVLHGRRVPRGTMITVPIGTYGYDPAL
FSEPEYFDPSRWMGRRVPPSAIETAAFGGGPHFCLGYHLAWMEVVQFAAAFARELTRRGV
RPQLAPGAAPPRLRHMPFGQPPKKTRIELARAA

>JQX13_51560|KEGG|CYP1286A2|[Archangium violaceum]|avm|DP

MLPPRIPRPALFQLFQWTAHPIGFMKKATERHGDCFLARFPKSPPVVFTSDPEMIRQLFT
GKPEDLHAGEANRLAEFVVGKNSLLLLDGKEHIHERRMMTPSFHGERMLSYGLSMRESAD
RVIDRWPVGRPFSLHAQFQDITLDVILKTVLGLSEVESLERFRKLLVRMLKLSTHPSLLF
LSALLPAERMHKLLSLGSKPLRLGPLKADMSQAMPWTRLSRLTQEVNTSLFAEMAWRRAV
GVEGREDILSMLMQARDEDGRPMSDQTRSCATR

>JQX13_51845|KEGG|CYP242A6|[Archangium violaceum]|avm|DP

MQQATNPQPLTFDLLSLETARNPHPMYHALRTQQPILWSPQLHGWVLTRHADISQVLKDP
RLVAGPMTGQFERLPEDIRKQLTPLRDAVNMWMGHTTNEGHLRFQALLKRYFTPRTVENF
RPRIQALTDTLLDAAAARGSSFDMVKDLAIPLPSNVIAEMLGVPLADEYLLQRWSRDLGG
IFTNFNPEQLFQSQKSVLEMMDYMREVLSQYRRGASGENILEVFLRAQDEGLVTEEEILA
NCVLLLFAGHETTARLISRGLALLFDHPEQLAMLRQQPSLIPQAVEEMLRYGDVAGMTTR
LTVAPVELGGQTMQPYQMVYVMLAAANRDPSLFPDPDRFDITRKPGKHVAFGYGSFYCLG
AALARLEAQVFFETLLRRFPNVRPAKGASPVWEQQGPLNMHLVTLPVEL

>JQX13_52240|KEGG|CYP1329A2|[Archangium violaceum]|avm|DP

MEASQPSKSIPRVSGAPLIGNLSEFRASRLDLLLRVSRECGDIGSFRIGPVEVVVISSAT
LANSLLVGHAEDFNGGPIGRIFMPLVGDKSLIFQDGAPHREQRKLLAPSFHPKRLAEFCQ
SMTRYTQEAASGWQDGQMLDMVHEMTILTMRIIGKTMFDVDFQNEASDLREAMMVTLEHL
DHMSSSAPIPLSWPTARNKRARAAIATIDNSIRAMIDERRRDATDRKDMMSSLLQARNDA
GQPIDESQIRAHIVTFFSAGHETTALALSWTWQLLARHPEVYARVRQEVDALGGKTPGFE
DLPRLSYLQQVIKESLRLHPTIFMIARAAVRDVEIEGYPIRKGQMVLIPPYCIHRNPEYF
PDPERFQPERFEPEREKSRPKQSYMPFGSGPHTCIGSHFAMMETQLIMATMLQQLDLELP
AGEQVKPQVLTVSLRPSAYKMRVKRRGSDAPQTKVA

>MEBOL_000247|KEGG|CYP253D|[Melittangium boletus]|mbd|DP

MNAPSASPRIPTGPRGLPLIGVIRDVRKDSLGFLMRTATEHGPVSGYRLGPGRSYLVSHP
DGLKHVLQDNVQNYTKDHVSYSLLRRVVGDGLVTSQGETWLKQRRLAQPAFHRARISAMA

AQMVRAAAELSEQWTDAQRSGEPRRAARDMLGLTLRIVGEALLGTDVRAQTDAVSAAFNV
ISEQTVERFRDLRVLPPILPTAYDRAFRQANQSLREVVATLVAQRRRHLEDRGDLLSMFM
LAQDEETGERMDDAHVQDEVLTMLLAGHETTATALTWTWALLEQNPDAERAFHAELDSVL
GGRLPTAEDVPRLVYTRRVLDETMRLYPPVYILSRKVVSDDTICGYRVLGGSMLDVSPYV
THRLPEFWPDPERFDPDRFSPERSATRPRYAYFPFLGGPRQCIGNNFALMEGVLILATLG
QRHRPRMVSGYTPKPEPLITLRPSEELPVVITSR

>MEBOL_000316|KEGG|CYP107DL5|[Melittangium boletus]|mbd|DP

MTAKTPERNQDGTCPASALREAPTWDSAVLDRENSEFIAKAHAIYDELRAVGPVVRCPFG
RGISERANPEASQAREPGKGTGGDRLFVTRYDEAVSVLLDERYSSDLRSVMTPEQRARLE
ANMPEEIRPLAHSILLLDPPDHTRLRKLVQPNFTARAMELLRPRIQRLVNDLLDTAERKA
VERGEAVSERRMDLIEALAYPMPVTVISDLLGIPVEDRERVHGWAETLLRAEGQDAMRDE
RIRTSMRAFGQYLDGLFERKRREPTEDMISQMLHVQEDGDTLSHQEMVSMVFILFFAGHV
TTVNLIGNGVVALLSHPEQHARFLADPAGLAKGLVEETLRYCGPVDYISTPRIAREEMEL
GGTHIPKGENLSLGLASANRDPARFAHPDVFDITRPDAHRNIAFGKGIHVCIGAPLARLE
GQLAFETLFQRFPKLRLAVPAEEMRWGSVASLRGFNRIPVLF

>MEBOL_000406|KEGG|CYP117E2|[Melittangium boletus]|mbd|DP

MQLNTPNALRERLSRPATYKDGIPILPGALPLVGHVPFNLGDTLTFLREAAHQVGPLFWM
RNLGSETQLVCMGEPGFELLKNRVTSSEFIREQAPDFIGDALLSRDGAPHRSLRTAMSGP
FTPRGLTSSGASALSAEVIEASLAKLPSGPFSLLAETQRLALDIIFRIMGIDRSELDAFN
HHYREFVLGAFPVKITVPFGPYWRSVRGRRWLDERFSRLIVAARGRTDMKGTLAALLAAR
DEQGQELKEVDLVQNLRLVALAGHETSASVMAWLGIVLAQRPDLWRQLREEAEAAPALPQ
SPEDLKRHPFAEAMFREVLRLYPPLSATARQATEDLTLHGKHVPKGTMVTVPVGVYGFDP
AIFPDPERFDPSRWMGRRLPPSAIETAPFGGGPHFCLGYHLAWMEVVQFATGFARELARR
GLRPRIAPGSAPAKLRYLPFGQPPKKTRIELVPA

>MEBOL_000745|KEGG|CYP264J2|[Melittangium boletus]|mbd|DP

MQSIPDLMPLAHRMNPYPLYAELRRDHPVCRVEPGGLVAISRYEDVEYVLKHPEIFSSHG
FRVAWQPEWVGDNPLAQSVAASDGQEHTRLRSLIGRAFTPAAINRLGERIRATASRLADD
LLARGEADFMESFAAPLPARAISALLGIDPSLERHYKRWTEVLVSITPIPENTEHVRRTR
DTIAEMKHHVQRLIDERRAQPSDDVLGLIVRGGPDGQRLSDPEIVGLVFTLLTAGLETTS
FLFTHAVRLLAERPDVLEQLRAHRELLPKFIEEVLRYNGPVQALPRIVTTEVELAGVRIE
RGACVLPLIASANRDEARFPHADRFDLEREESGIPFGYGAHYCLGAFLARLEARTGFDAL
LDRFSGFSLIPEGLVWNRGLVTSGPVKMPVRFLKN

>MEBOL_000961|KEGG|CYP262F1|[Melittangium boletus]|mbd|DP

MDSSSPSHGASAPRARLPPLASGLPLVGSTPQVLANPIRFLMRARERYGDIYTLDLGVQR
MVVLNHPRQHEHVFLTKSKNYLKQGALWDAMRTLLGNGLAVSQGEVWKRQRRMVHPQFHR
QRLARITSLMVAAIEDQFERWDEQTRAGQSLDAVPMFAELTMKLLAKSVFGTAVSHESVE
TLRPEMLYVIKYMVPSMLTQSLPSWVPRPGERRYQQALERIDRLLFSVIEQRRGEPGGSD
DLLSMLLEALDEETGLPMQARQLRDETLTLFLAGYETTSVALAWAVHYILEDAAVWRRLR
EEVEQVLGDREPTFGDMGELKYTRMVIQESLRICPPIWWNPRLAMEDDEIDGYAIPKGSV
IAPLTYVVHHHPDVWKDPERFDPLRFASEEGETRHRCAWGPFGIGPRKCVGVEFAMMEAQ
LVLAMFVRRYDVSRMAGPPVEKAMLGTLRPKAGIPIGLHRRRS

>MEBOL_001720|KEGG|CYP167B10|[Melittangium boletus]|mbd|DP

MSAEQTNPSENRPASQFNPYAPGYEANPHPMLEKLRAEAPLFYWELGRSWIVSRYEEGLA
VLRDDKRFSPNREDWEFASVMGSDAIIPEMEELNKNGLFALGEQGHARVRRLVSPAFTPR
ATERLRPEIQAIVDELLDTMAAKGRLNMVHDFSERIPARVIGSMLKIPSGHEEQFHEFTN
AVAKSLFPSALAPEELAPLRRQIREGISLVTETIEDRRRNPRENDILTTLIQTEEQGDTL
NKHELLALVSSLIVGGFETTVHLMSFCVYSLLQQPGVFAEVKARPELIKNLVEEVLRFDN
FVKMGPARYALEDLEMGGVRIKKGQMLIILLTSVLRDERAFDKAHVFDVQRNGNSNIAFG
HGAHYCLGANMARLLVQIAMGTLVRRFPEMRLVNPPAFGPHSLMRKMEVLEVELGTPSA

>MEBOL_001721|KEGG|CYP251V1|[Melittangium boletus]|mbd|DP

MTAPPANLQRDVSPTTPPERPPLTVAVMEAPGGLPLLGHLPRMVRAPHEYIQSLRAHGDI
VRIRVGTSPVYVVTSPELIHQVLVARSDGFERGRVFDKATAAIGKGVIVTNGDFHLRQRR
LLQPGFQRARIHGYMETIHRQVDTQAARWTPGGRISLREEMHRFTLNAVTRTLFGANTEE
GIATEIGDYIDAANAWVSHHTLLSSEFFERLPTPVNRAFVRKKARFDELIGAFIDARRAD
ERDHGDLLSALLAAQDAETGKGMSDEQLRIEIAGLFVAGSETTATALSWLFHEVGKDPQV
EARIQAEVDSVLGGRPVTADDLPRLQYIQCVVSETLRLYPIAWLLMRRTLRDLELGGYHL
PAGVEVLVSPIMSHRNPRIYPEPMRFDPDRWLPERARELPRYALLPFGDGKHKCIGDMFA
RTEMVIAVAAIAARWRLVPVPGHRVWEVPRSVLRPNQVLMTTVPRTPHTPQETQS

>MEBOL_001915|KEGG|CYP1284B2|[Melittangium boletus]|mbd|DP

MDAFNGQKLDKIPAHVPPELVYEYDNARDPRLLQDPHARMRSLILEAPPIFFTPYNGGSW
VVTRKKAIVDITMNPEVFSNAFISGAKDSQQSLSLLPISVDPPKHTAYRAPLNQPLSAKS
VAGLESAIRAMTNELIDKVLAAGRCDFLSDIAEPLPVTLFMKLAGMPTDRLAEFRHLATQ

ATSSTVDHATREGVFKRIAGILAEVIKARQEKREEDLISHLLDVNINGQNPTFQEMLGYS
ITLFLGGLETVVNALSFGVLRLARDQELQAKLRADPSLLPGAIEELLRLHGIASTVRQVT
RDEVCHGVQFKKGDTVSLLLPAANYDDAAFPNPEQFILGRTEQHQTFNTGPHRCVGLNLA
RLEMKVFYQEWLKRVPSFRLDSQSPPRFMGGFNLAVTSLPLVW

>MEBOL_002117|KEGG|CYP1492A3|[Melittangium boletus]|mbd|DP

MNTALTLDAPRPLHEYAPSSLELIKGIRREGYLGWMMKVWRQHGDLVRIRMGSRSFLLVT
HPDHVRHINVTQRESYDKAESYDSLRELLLGNGIVTATGVDWRRQRKLMAPFFTPRGVEK
FYPIFLSDTQQLIEHWRSQHQGSGRPVEMLDEMMQVTAAVILHSVFSTDTGETLQRIKHS
IETMISHISNMQMRPVQVPLWVPTPGNLRFHRAQKLVTAYIHELIARRRAIPTEQWPDDL
LTKLMTTRDEETGTVMAEQLLVDNGITLFAAGHETTARTLSFLWYALSQNPEVERRLHAE
LDSVLGDAPPTINDLKKLPYTLQVVKEVLRLYPAAPMYARDAVADDDLDGVRIPAGTRTI
VFAYATHRHPAFWDEPERFDPDRWLPEREAERHAHAYHPFAIGPRICLGNNFSLLETHVM
AAMLARHFKLRMKPGHVPQFDLLGTLGSRNGLPMLIEARTGTKTL

>MEBOL_002190|KEGG|CYP107DP7|[Melittangium boletus]|mbd|DP

MSTTLSPQEIWAPHTRFNPLPLYARMRPEGPLFRQMDANRGVPIWLSTRYKASVELLRDP
RFTKDERLVAPQARQARVAEMEAINRNMLMSDPPDHTRLRTLVSKAFLPRRVEELRSRVE
AIAHRLLDALPAQGSVDLIDAFAFRIPVTVIAELLGVPPEDQDQFRVWTTAITQLPSADR
LESIKQASLAFMAYVQNLMEKRRAEPREDLVSDLLQAEEQGDRLSSEELIGMVVLLLNAG
HETTVNLIGNGVWALLRHPEQLERLRQNPALIDSAVEEMLRYRGPLECATHRWAREDIEF
HGQVIPAGETVLVSLLAANHDPEQFPEPERFDITREPNRHIAFGFGIHFCVGAPLARLEA
SIALPLLLERLPRLRLAVDESQLQWRQGLVLHGLKQLPVAF

>MEBOL_002509|KEGG|CYP102M15|[Melittangium boletus]|mbd|DP

MGKMLAERLNLVTGQLGMERVSIPQPRVRPVVGNAPDLGSETPMQNMMKLARELGPLFRL
SFPGGRSLLVVSSHELVADLCDESRFDKKVGAALEEIRDFAGDGLFTAYTHEPNWGKAHR
LLMPAFGPAAMRGYHDEMLDVADQMLTRWERFGPEEAIDVPDNMTRLTLDTIALCGFGYR
FNSFYQREMHPFVESMVRALAEAGNRARRVPLKTQLMLRTRRQFQVDASYMHEVTRELIA
QRRKLAPEDAPRDLLGLMLSEKDPLTGEGLDDDNIRNQLVTFLIAGHETTSGLLSFALYF
LLRHPDVLQKAYAEVDRVLGSETPRFEQVNQLHYIDQILRETLRLWPTAPGFSVQALVDT
RLVGTHPLSAGDTLFVLTPMLHRDPTVWRDPERFDPDRFAPEVRDGIPQHAWKPFGNGQR
SCIGRAFALQEATLVLGMLLQRFHISEPAPYSLHVRETLTLKPEGLRIRARVRQNVSRSL
TTRPSPAPVAATSQGQGGVAAHGTPLLLLYGSNSGASEAFARRIASDGLARGYSAKVAPL
DEYTGKLPKQGAVVLVTASYNGQPPDNARGFHAWISHVPEGSLLGVRYAVFGCGNRDWAA
TYQAIPKHFDERLHAAGAQALVARGEADARGDFFGDFEHWYAPFWSTVGEALGVSTGEVN

SGPLYKVEVVPSASVDLVAQNKLGMSTLVENRELVDMTSPLGRSKRHLTFRLPEGVTYAA
GDYLAVLPENHPDLVERAARRFGVRTDAAVVLRSTRGAMAASLPTDRPVSVQELLGRHVE
LSAPATRKDLERLAAMNPCPPHAQHLLALAQDAERYKAEVLDKRVSVLDFLEKYSSCVLS
FADFLELLPTMRVRQYSVSSSPRADPTQCSLTVAVVDADAWSGQGRFHGTCSSYLARLHP
GDPVAVAVRTPNAPFHPPASNATPLVMIGAGTGLAPFRGFIQERALRHVHGEAAGPALFF
FGCDHPDVDFLYREELAAWEREGVVKVLPAFFRQPEGDVMFVQHRLWKEREQVKALLAQG
ATVFLCGDGRRMAPAVRETLARIHQETVGCSEEEASRWLAEMEKQGRLVADVFA

>MEBOL_003675|KEGG|CYP109R9|[Melittangium boletus]|mbd|DP

MSLVDRFSFFDPEVMQDPYPYYAELRERAPLLWNAPLQAYIVTRHEDVAHVLKNPALFSS
ASLRLAGQPIDVVGTILGRPSVPALVNTDPPLHTRMRSIVSRTFTPKRISALEPRVRELS
QKLVAEMTAQEEFDFMEGLATPLPVIIIAEMLGIEPARRRDFKRWSDTLISLASAAGPGP
EFRRDGQEMHAYMTRIAEERRLAPQEDLISLLVQGGEGQTLSAEEVNAFALLLMLAGNET
TTNLLGNAMHALLSHPEQFDWLRKNPSGCGAAIEEALRYESPVVSVMRRTLREVELSGGK
VPADSMLMLVLSSANRDPRKFPDPDRFDIQRDAQGVMSFGHGIHFCLGAPLARLEAPTAL
GELIARAPRLRFAPRQPLRPEYPPSFGVRALKTLWLRRE

>MEBOL_003803|KEGG|CYP110R3|[Melittangium boletus]|mbd|DP

MTHPLPPGPRFTPVQALRFLRDPTRFCLESKARYGDPFTAPLPVGDVVLTGQPDGIREIF
SADPAVFEPLGNLPLAPIVGELSMLLLSGPRHKRERKLLMPPFHGERMRAYGQTLRALTL
HAAESLRPGEGLRAQHLTQHISLEAIIQIVFGVQEPERVRRYREVMVPAMDSYTPLLMMA
VPLRRSFGGLGPWARFQHHLAQVDALLTEELANRRGHEAGHTDILSLLLAARDEDGQAMT
NEELRDELRTLLLAGHETTAIGMAWALHYVHRLPEVQARLRDELAPLGPSPEPEALTRLL
YLGAVCDEALRLHPVVPVVGRRTLAPFTLLGRELPVGTGVMAAICLAHASPELYPEPERF
RPERFLERRYSPFEYLPFGGGARRCIGAAFAQYEMRVVLGTLLAAHRFSSPPHAAPERPA
RRNITLGPRHGVALVYEGPARA

>MEBOL_003931|KEGG|CYP253G4|[Melittangium boletus]|mbd|DP

MTTSTLPLAPMPPGHWLLGHLRERETNPLGLFLRGREQLGDVVRFRMGPQYVEQLAHPDH
VKHVLADASARYTKGSIFAKTRPLVGNGLLTAEGEFWKRQRRLSQPAFHKDRLATLAGVI
TQTATEAIAGWEAPVAAGTPVPVFTEMMRLTLNVVVRALFGLDVAGQTQLIGDAFTQALE
VTNRRIISPLPYVPWFYRLPTRDNRLFQRAMDTLHHTVEGLIAQRRAQARDGQSPPGEDL
LGMLMTACDADTGDAFDDLQLRDEVMTLMLAGHETTATSLAWTFHLLEQNPEQEALMHEE
VDQVLGGRTPTLEDLPKLRYTGCVFEEAMRLYPPIWALPRTPHEDDEVGGFRIPKGDIVL
LVPYVTHRHPEFWPDPERFDPTRFLPEASRQRPRWAYLPFGGGQRQCIGNNFAMMEAQFI
LAMVAQRFRLRGVPGVPVEPEPLVTLRPKGPLPMHVHRRQGKSRTHLASSA

>MEBOL_003979|KEGG|CYP253S1|[Melittangium boletus]|mbd|DP

MTHDAARRIPPGPKGHWLLGNLPERRSDPLSLFLRGRERYGDVVRYPMGPFLMHQLSHPD
DVKRVLVDNAQNYRKTALMQRLRPVLGEGLLLSEGDFWKRQRRLAQPAFHKERLAGMATV
ITGLIEDALPRWDALAERGEPFDLSAELMRLVLAMTGRVLFGADLSDSARDVARAVTTVL
EELNHQVLSVLPLPASLPLPGHRRLRRAIQVLDSIVFGIIDARHRGAHASEDLLAMLMQA
RDADTGEGMSDRQLRDEVMTLVLAGHETTANALTWTFHLLEQHPEAEARLAEEVTRLLGE
RTPTLQDLPRLGYTARVFDESMRLYPPAWLISRVALNDDILGGYPVARGTIVVILPYVIH
RHPAFWEHPERFDPDRFLPARSGARPRFAWLPFGGGQRMCVGSGLALLQGHLVLAMLARR
YHFQRVPGHPVEPQALVTLRPRHGLRVTARRRSAPR

>MEBOL_004399|KEGG|CYP109AK1|[Melittangium boletus]|mbd|DP

MQSSDYNPLSSTVQADPYPYYATLRENTPVYFNEQFGWYIVSRYEDVVAITKNPAVFSSA
RAIVGPEKLDEAAKVAPHALRTFRRGVLISEDPPTHTKTRGLVTKAFTPKRISEMEPRIR
QLARELISQLPRSGEFDLIKDLAEPLPVIVIAEMLGVEPERRHDFKRWSDDALATSFALA
KGSELSGIERSNRELSDYMAQALEARRQQPREDLVQALLDNGVREGVLSVSDAVDFCRLL
LVAGNETTTNLLGNGVRALLSHPDQRDRLIREPALIPNAVEEMLRYDSAAQALFRKTTQE
VEVAGTRIPAGASVLLLFGSANRDPRKFQEPDRFDVTRNVVGQVAFGHGIHFCLGAPLAR
LEAKVVLEELLTPDRRLSLVPGQPWEVLEHFSIRGLKSLRVRVEPAAGARASA

>MEBOL_004662|KEGG|CYP107DL4|[Melittangium boletus]|mbd|DP

MAMNPMDEKRTEESGTSPVGVCPMKASREVPHAGSVRLDREDPAFLAVAHEAYERMRKQG
PVVRASFGRGFSDDLLPDGDKPRTAKGEPGSERYFVTRYDEAVEALLDDRLSSDFRSTMT
PEQRAQLPFMPEELKPIAYSLLMLDPPDHTRLRKLVQPNFTARAMETLRPRIQRIIDRLL
DQAESEAAARGESAPERRMDLLPAFAYPMPIAVISDMLGIPEEDRAQVHTWAENLLSAEG
PSIGDEQRRGVLREFIRYLEGLFERKRRAPGEDMISQMAHFQEDGDKLSPQEMMSMVFIL
FFAGHVTTVNLIGSGVVALLTHPEQLERLLADPSLVKGAVEETLRYWGPVDYMGTPRIVL
KDMELAGCPIAQGEKLTVGLASANRDPERFPNPDVFDITRPEAHRNIAFGKGIHVCIGAP
LARMEAQLAFETLFRRYPKMRLAVPAGELRLAATSLRGFDRVPIFF

>MEBOL_004948|KEGG|CYP1506A5|[Melittangium boletus]|mbd|DP

MPNVLSRGVFNLFFGSKRQAFASLPGPQPGILGTAGDFLGAQPWDVCARYGREYGGVTLI
WMGPNPGLVLNDPVLIEQLLDSRRTEFEKGSLRQELTPTTTDQSLFIAPQSGDWAEKRRI
DPFVQPWSPDWLAAQVAPMQAAISDFVDGLIASGKTVDLAPVLRRLTCDAFAVAAIGEKL
PDSVYEDFMLLAKGADARIQAKLPLKFVSLPKGFEEARERFYRFFDERVSAARRNPKPEA

NDLMSRTMREVPDFGDKALAVNIALIFYGGAFSSSTALAGAFHQLQKHPDAEARLASEAA
AFAGGPPTLERLNEARWAEAVVLESMRVLPPVRVFTRSPVTDVSFAGVTMPAGATIMVSN
QYLHRDPQHWPNPDTFEPARWLDGGTARDPIGSGHYFPFGRGPRMCVATDFAMVFLRTAM
VTIAARAKAEISSTEPFEEGFFFGVVLPTNVHTKFITRPR

>MEBOL_005233|KEGG|CYP264D7|[Melittangium boletus]|mbd|DP

MDSPRFTRRAPMSERINLLAPEVRANPYPLYARLRQHAPVSQVDPGGIWAVSRYEDVMYV
LKHPQLFSSEGFRQAYRPAWISNYPLADSALVMDPPRHTRLRGLINRAFGTSVLTRIEPR
VREIAATIAANLPEGQEVDFVEAFSVPMPMGVLGELLGLAPEVHPLLVRWAQHLAQFTSI
GPADTARQEAMRATVNEARGHFERVLAERRRHPGDDLMSDLVQARMDGESLSDTELLGFM
FLLLIGGLETTLHLMSHVALRLHLQPELMTLLREQPALVPRFIEEALRHEPPAQGVMRLT
SEETELGGVRLPRGVRMLLLMGSATRDEAYCADPDRFDPHRTAPQNLPFGHGIHFCLGSQ
LARLEARLSLEALLARFTRLSAGTQPIQWNSSLIVRGPTRLPLVAHVD

>MEBOL_005971|KEGG|CYP242C1|[Melittangium boletus]|mbd|DP

MSAAPAVSTSNYDLFAPSVLEDPTELLTRLRAENPVYYSPQLQGYVLTRYDDIVMVLKDP
DMGSAMLTGWIDQMPQEAQEQLQPLRASLDLWMGHKNNDDHQRIQRVLRRYLTSATIESL
RPRVESLVKSLFDAVEGKTEVDIIQEIANPLPCNIIGELLGVPQEDRDQLQQWSQQALNL
FRIADLPTLLETQKAILAIQDYMKPIVEARRHAPLDDLPSVLATAQAEGQMKSDAEILGN
CVALMFGGHETTVYLIANALLALMQNPEQFQMIKDRPELIPAAVEETLRYDGPVDLITRV
TPKPLELVGGHVPANSMMILMLRAGSRDPALYPNPDRFDITRPTTVRSLAFGLGSFYCLG
TALARMETQICLREIIHRMPNIRPLFDVNKPDYRPLPPIRRRLETLRVAL

>MEBOL_006681|KEGG|CYP107DP8|[Melittangium boletus]|mbd|DP

MTTSPLFAEGETLWSPHVRANPFPLYKRMREEFPVARITDPQLGMHFWLLTRYDDVVSMV
RDPRFTQDPTRLPETVRQRFFGHKLRSLSRHLLTVDPPDHTRMRSLVSKVFTPRRIEELR
PRITAICSELLENLKAQGSGADFLKVLAFPLPVIVIAELLGIPSEDRDQFREWTQTFFAP
PSKDSQEKAREAYQLFFKYLEELFERRRQQPQDDLISALIAVQEQGDRLSAEELMSMVFL
LLVGGYETTAHLLGNGLLELLRHPEQLQRLREDRALIPSAVEEMVRYCGTFELSILRFAK
EDLELHGQHIPAHEAVRVNYLAADRDPGAFSEPDRFDVGRTPNKHVGFGHASTSVWERPS
PGWRPSSPSSCCWSDSRSCVWPRNPRSSCGARVPKRVGW

>MEBOL_007050|KEGG|CYP1347B4|[Melittangium boletus]|mbd|DP

MSSVMPLMEEDRNLLSPENLANPLPLYEELRENEPVHWSPRVNAWFLTRHEDVLACFRDP

RMSADRLKFFEHQIQGLDPDTIREFMETSRNQMVMRVGAEHIRLRRQTGAGFTPPRLDAM
CPSIHRTMKQLLDRVYEQGRMNLAQDISYPLPTRVIADLLGVPAEDRERFQRWADMLADF
AAPAAGACMLDAARRANQAMVEMKDYFLPLIEQRREHPTPDTLSLMVQAQEQGQMTPGEL
VANAILLLFAGHTTTTDQLSNCVHDLLAHPEQLQLLRRRPELLRTAVDESLRFHPAVPFV
FRVAVEDVALRGRLIRRGDVVFLGMAAANRDPRAFRDPDRFDITRDNSHPRHLSFGFGAH
HCMGAGLARREMETGVSMLLERMPRLRLDESRPTRLKCHSLNFRGFEVLPVRW

>MEBOL_007148|KEGG|CYP120G3|[Melittangium boletus]|mbd|DP

MTSPADASPPSPPETLPLPPGSSGLPLIGETLEYLRSSRAFAERRQRQYGAVFRSHVLGS
PAAFLLGPEAVQWIFAGEGKYLKNRWSPGVRRLLGAHCLAMLEGNEHLERRRLLAPHFSY
ASMRGFVPIIESLSRRHFERWTARPGDFTLWPAMRELAFEIALALIFGQDTVDLPFLMHH
FKAWTAGLFSPFPIDLPWTTFGRALASKKAMIGYLEQVVAERQARTEQPPDLLGSLLQRR
EGEEPLSRAAIVDELQLLLFAGHDTTVTAMSNLMLMLAQHPDVLQRGREAVAGLEGPLTL
EGMRATPYLVQLLHEGMRTIPPIGGAFRVTTRDVAFNGYRIPKGWTVPVSIKMAHAGANW
PTPDRFDPERFGGENPEQKKPGTFIPFGGGPRICLGQHFAMVEMSVMLILLLKHHAWELV
PGQDLEYTQVPFPKPRSGIQLEFRRIDAGERPLAEP

>MEBOL_007173|KEGG|CYP107DP5|[Melittangium boletus]|mbd|DP

MMRRMNSSPASFQELWAPTTRANPLPLYARMRQEAPVVRVVDPYFKLPVWVVSRYKDAVE
LLRDSRFTKDQSKLPEDSLPRQMRNDTMAALNRHMLMADPPDHTRLRTLVSKAFTPRRVE
ALRPRVTAITERLLDGVKKQGSVDLLSAFAFPLPVTVIAEMLGVPAEDQARFHEWTTLIV
NPPLDGDLGPLRAAGMQFLLYFRDLMEKRRSDPRDDLLTALVSAEEQGDRLTTEELVGML
FLLLVAGHETTVNLIGNGVWALLRHPEQLERLRKNPALIESAVEEMLRYRGPVETTTQRW
APQDLEFHGQVIPQGETIIASLLAADHDPEQFPEPERFDITREPNRHIAFGFGIHFCLGA
PLARLEASIAIPLLLERLPQLRLAVDEDALQWREGILVHGMKQLPVAF

>MEBOL_007319|KEGG|CYP197AA1|[Melittangium boletus]|mbd|DP

MSTARAALPPGPRSLPVLGTLIDYAKDPLGFIEACVRDHGDVVYVELLGQRTYVLQRPEH
IEHVLVTRHRHYIKDAFQRELMGGRLLGNGLLTNEGEPWLRQRRLMQPAFHRQRLAAYGR
VMAEHARRQLATWRDGEVRDVNADMMRLTLGIVIKSLFDLELDGKAEAVGPSLARVMEHF
AGVQALIFPDWLPTPENLGYRAALGQLDALVSSLIRRRREAGGGETEDLLSTLLHVQDDE
GQHMSDPQIRDEVMTLMLAGHETTSINLAFCFHLLARHPEAEASLHRELDSVLGGREPTL
EDLPALPFTDSVVKEALRLYPPAWTLGREALEDDEIGGWSIAKGSVMMMNPWTVHRDARL
YEDPLAFRPQRWVDGLEKRLPRFAWFPFGGGPRLCIGMGFALMEARLVLATLAQRFRFER
APEDDVELLPSITLRPKHGVKVRVRTR

>MEBOL_007543|KEGG|**CYP167B6|**[Melittangium boletus**]|**mbd|DP

MSPDSTQSHPPTVSFSPDAPGFAANPFPVLEELRKAAPLSYWKEGRAWLVTRYEEALAML
RDSKRFSTNRAHWEFASVSGIAAIIPELAELNNAGLFALSGADHARLRKLVSPALTPRAI
ERLRPEVQAIVDEILDEVSGQDTVNVVSDIADRIPPRVIGSMLKIPKGRETLFNRFTEAA
VKSFLPGLLRPEDVPALRADVQEGIALVRETIEERRRNPLPDDILTTLIQTEEQGDRLST
AELLSLVSSLIVGGFETTIHLIGFTTYNLLKRPELLAQVLVEPDLIKGVIEEVLRFDNFG
KMGLARYALEDVEVGGVTIKKGQMILLMLNSALRDEAAFSQADVFDARRNTNSSIAFGYG
VHFCLGANLARLELQVVMNRILERFPTMSLVKEPSFGPHPVIRKMESLEVQLRSR

>MEBOL_007929|KEGG|**CYP1284B4|**[Melittangium boletus**]|**mbd|DP

MHAFSGQKLDKIPAHVPPELVYEYDNARDPRMLEDPHGRMRSLILEAPPIFFTPYNGGHW
FVTRKQAIVDITMNPEVYSNKISGEHSGEHKDSQPGLNLLPISEDPPRHTAYRTPLNQPL
SAKSLAGLESAIRVMANELIDKVLAAGRCDFLSDIAEPLPVTLFLKLAGMPTNRLAEFRH
LAVQAASATVDPATRAETFKQIAGIMAESIQSRQEKREEDLLSRLLDAKIDGRNPTFQEM
LGYSMLLFLGGLDTVVNAMSFGVRHLARDQELQAKLRADPSLLPGAIEELLRLYGIASVP
RRVTRDTVCHGVQFKQGDTMLLLLPAANYDDAAFPNPEQFMLGRTEQHMSFNSGPHRCVG
LNLARLELKVFYQEWLKRVPPFRLDPQAPPRFMGGFTLAVTSLPLVWE

>MEBOL_007972|KEGG|**CYP1O9R3|**[Melittangium boletus**]|**mbd|DP

MDLLSRFDFFSPDVMRNPYPYFAELRDKAPLHYDAKLKAHVLSRYEDVAFVLKNHALFSS
TKIRIAGKLQEERASVKELGSVSNLVSMDPPVHTRLRGLVSRSFTPKQINAMEPRVRELS
RELVAEMTSRSEFDFMDALASPLPVTIIAEMLGIDPVRRRDFKRWSDNLIQSNNATLQSG
ETPEEVIRSAKEMLAYMTEVAEARRREPRGDLISLLVQEIDGVAALTPPEVNSFTTLLLI
AGNETTTNLLGNALTALIRNPGEYEAFRRDPSPAACSAICEETLRYDSPVIGLFRRTTQE
VEMSGGKLPADTSVMVLLASANHDPRKFPEPERFDPTRDTHGLMSFGHGIHFCLGAPLSR
LEAPVALRELMERAPRLGFSPRQPQDIDYGGSFFLRGPKSLWLQKN

>CYFUS_000165|KEGG|**CYP11OR3|**[Cystobacter fuscus**]|**cfus|DP

MTTPQLPSGPRITPVQTFRFLRDTTRFFLDCRARYGDPFTAPLPVGNVVVTGDPEGIRDI
FSADPALFEPLGQLPLAPAVGDNSLLLMAGQRHKRERKLLMPPFHGERMRAYGQLMREIT
LRSVETLRPGGPFRAQELTQSISLEVIIRAVFGIEEPARMRRYREVLVGYMESYTPLLMM
AVPLRRSFGGVGPWARFQRYVTELDQLLTEELSTRRGHEVGHTDILSLLLAARDEEGQPM
TDAELKDELRTLLIAGHETTAIGMAWALHHLHRAPGTMQRLLEELAPLGPEPEPEALVKL
PYLTAVCDEALRIHPIVPVVGRRTVAPFTLRGRELPPGTGVMAAICLAHVDPVLYPEPDS
FRPERFLERKYSPFEYLPFGGGARRCIGAAFAQYEMRTVLGSLLAAHRFSLANDTPERPV

RRNITIGPARGVEMVYEGPRRGALA

>CYFUS_000318|KEGG|CYP264D9|[Cystobacter fuscus]|cfus|DP

MSGRINLLAPEVRANPYAVYAELRRHAPVCQVDPGGYWVITRHDDVVAAFKNPQLFSSTG
VRMATKPEWLGHNPFADSMIGQDPPRHTRLRNLVNRAFGPPALARLEGRVRHYAEAIVAR
IPEGREVDFVDAFTLPLPASVIGELIGLEPSLHSHCKRWADDLTSISANPQDEKRREEIR
AAVRETEAEMARVLALRRSEPREDLVSELLQARVDGQSLSDAELMSFMFLLLVAGLETTI
HLLGHCVRVLMERPDVLARLRADRSLLPRFIEEVLRYEPPVQAFGRLTTAEAELSGVRIP
AGAKVMLLIGSANHDDARFPDGDRFDMDREGVNNLPFGHGIHFCLGAPLARLEARIGLEV
LLSRFERFTPAGPLEWNHSFTVRGPRVLPVIAHGSA

>CYFUS_000663|KEGG|CYP167E3|[Cystobacter fuscus]|cfus|DP

MLQKTPTPDHGPDFVLDMEDPAFVHNPYPTYAWLREKAPAYRWKARGDAIVFSRHKDVRA
LVLDRRFSNDYRMWEFARKEEWPAEHAEYKSIMDNGLFGLADADHLRVRKLVSPAFTPRA
AERMRDEIQKAVDDIIAEQVKGERVDLTTITEPLPMRVVSDMLKIPEDLRGEFRAFGLAS
IRSSVLFTKSEELFALIAPMPRWIRMLREVIAERRANLLENDLLSTLITASDEGQKLTEE
EMISLVHALIVAGSDTTVHAANWALYSLLRHPDQFALLRDEPSLIRNTIEETLRYDLFGK
GGLPKFAREEMEFAGTKLRKGQMVMPFIPAALHDPEVFPEPERFDIRRDVSQTIAFSAGQ
HFCLGAALARQELDLVVGTLVRRFPHMRLSQEPEFVPHPIMRALTRLEVTLS

>CYFUS_000749|KEGG|CYP253G4|[Cystobacter fuscus]|cfus|DP

MNNSAPLPPMPPGHWLWGHLPERESDPLGLYLRGRALLGDVVRFRMGPIYVEQLTHPDHV
KYVLADAPARYTKGPIFHKTRPLVGNGLVTAEGDFWKRQRRLAQPSFHRERLAGLAGVMT
ETAAEVLAQWEPRVRAGEPVPVFPEMMRLTLLVVVRALFGVDVAEHTRELGESFTTALEV
TNERIISPLPYKPWLYRIPTAKNLAFQRAMVPLNRIVEGIIAQRRARGPADESQDLLGML
MAARDADTGDTFDDVQLRDEVMTLLLAGHETTATSLAWAFHLLEKNPEQEALLHEEVDRV
LDGRIPTLEDVPKLRYTSCVFEEALRLYPPIWAIPRVAEEEDVVSGYRIPKGDLVLLVPY
VTHRHPDFWPDPERFEPTRFLPENSKQRPRWAYLPFGGGQRQCIGNNFAMMEAQFILAMV
AQRFRLRGTGAPVTAEAHVTLRPHGTMPMHATRREKQPQLQSA

>CYFUS_001301|KEGG|CYP253D3|[Cystobacter fuscus]|cfus|DP

MTDTSAAPRAPAPRIPTGPRGLPLIGVVREARKDILGWFLRTAAEHGPVAQYRFGLGRSY
LVSHPDGIKHVLQDNVKNYTKDHFSYAMVRRVVGDGLLTSQGDTWMKQRRLAQPAFHRAR
ISAMADQMVRATVELSEQWAEAQRTGEPRLGAVDMMSLTLRIVGEALLGADVRADTEAVG
RAFTVISEQTVERFRSLRFIPPVLPTAYDRAFRDANRSLRQVVTRVIAERRAHTEDRGDL

LSMFMLARDEETGERMDDTHLQDEVLTMLLAGHETTANALSWTWALLAQNPDAERTLHAE
LDAVLGGRPPTVEDIPRLVYTRRVLDETLRLYPPAYALSRKVVEDDVICGYQVRSGSSVD
MSAYMTHRLPEFWPDPERFDPDRFTPEKVAARPRYAYFPFLGGPRQCIGNNFALMEGTLI
LATLAQHHRPRLVEGYTPRPEPVITLRPSGHLPVRITPR

>CYFUS_001363|KEGG|CYP264A6|[Cystobacter fuscus]|cfus|DP

MTTQRVNILAPEFRADPHPRYAELRRNAPVTQVEPAGFWAISRYEDVAFVIKNPQLFSSQ
GFKAAWQPEWVGYNPLANSMLVLDGVGHARMRTLVSRAFNASAISRLEVRIRQLANRLVD
ELAEKGEADAVSSFAMPLPAFVIGELLGLDVSLHHRFKDWSDDITSVTPVPHNPEHAQRT
LTAITDATRYISEIIEARRRSPADDLVSDLIHAEVEGQSLTDREIVDFLVLLLIAGLETT
VHLLAHSLLFLAERPEEQARLRAQPALVPGFVEEMLRYEAPVQALLRVATSDVTLSGVKI
PQGDVVLALLASANRDERHYTEPDRFDVHRGQPGLSFGYGIHYCIGAQLARMEARCGLEA
LLSRFSGFTRTSTELSWGQAITVRGPQHLPLRFIPA

>CYFUS_001619|KEGG|CYP107DL6|[Cystobacter fuscus]|cfus|DP

MATNPVNEQTTKAAASGPTPQATCPMKALREAPRCPALLDKESPEFLAHAYATYADLRTQ
APVVRTSFAHNFADAVLPGGTKRPESGSHPAKERLFVTRYDEVVEALLDERLSSDFRTSL
TPEQRANLQYMPEEVRPIAYSLLMLDPPDHTRLRKLVQPNFTARTMEMLRPRIQRIADTL
LDQVERAAAERGEREGERRMDLLHAFAYPMPITVISDMLGIPEEDRSRVHGWAESLLGAD
RRDAAMDELRRSSLREFARYLDGLFERKRREPAEDMISQMVHAQDEGDKLNHQEMVSMVF
ILFFAGHVTTVNLIGSGVVALLTHPEQLERFLADPTNLSKGAVEETLRYWGPVDYMSTPR
IIKEEMELAGTHLPQGEKLSVGLASANRDPQHFPNPDVFDITRPNAHRNLAFGKGIHICL
GAPLARLEGQIAFETLFRRYPRMRLEVPFESLRWNTGSGLRGFDRVPVRF

>CYFUS_001993|KEGG|CYP1492A2|[Cystobacter fuscus]|cfus|DP

MNPALAIDTPRPLKEYAPSTLELLKAIRREGFLGWMTNTWRQHGDLLRIRMGSQSLVLVT
HPDHVRHVNVTRRESYDKGESYDVLRELLLGNGIVTATGEDWRWQRRLMAPFFTPRGVEK
FYPIFLSDTQQLIERWRSQLQGSGRPVEMLDEMMRVTASVILHSVFSTESDEALLRIKNS
IETMVSHISETGMRPVQVPQWVPTPGNLRFRRAHKLVTAYIRELIARRRAIPTEQWPDDL
LTKMMTIRDEETGRLMAEQLLIDNGLTMFAAGHETTARTLSFLWYALSQNPEVERRLHVE
LDSVLGDAPPTLNDLKKLPYTLQVVKEVLRLYPAAPMYARDAVADDELDGVRIPAGTRMI
VFSYGTHRHPAFWDEPERFDPDRWLPEREAARHAHAYHPFAAGPRICLGNNFSLLETHVM
AAMLARRFKLRLKPGHVPRIDMFGTLGSSNGLPMLIEAR

>CYFUS_001994|KEGG|CYP1284B4|[Cystobacter fuscus]|cfus|DP

MNAFNGQKLDKIPAHVPPELVYEYDIAGDPRVLEDPHARMRSLILEAPPIFFSPFNGGHW
FVTRKKAIVDITMNPEVYSSKNPGEHGKEATGGLSLLPISVDPPQHTLYRTPLNQPLSAK
SVAGLETAIRAMTNELIDKVLAAGRCDFLPDIAEPLPVTLFLKLAGMPTNRLAEFRELAL
QAASATVEPATRAETFKRIAGILAETIEARQEKREDDLISRLLDANLDGRNPTFQEMMGY
SILLFLGGLDTVVNALCFGVRHLARDQELQAKLRADPSLLPGAIEELLRLYGIASIPRHV
TRDTVCHGVQFKQGDALLLLLPAANYDDTAFPNPEQFILGRTEQHMTFNTGPHRCVGLNL
ARLEMKVFYQEWLKRVPPFRLDPQAPPRFSGGFALALTSLPLSWA

>CYFUS_002491|KEGG|CYP264D7|[Cystobacter fuscus]|cfus|DP

MSGRINLLAPEIRANPYPAYAELRRNAPVSQVDPGGLWAVSRQEDVMHVFKNPQLFSSQG
FRQAYRPPWISNYPLADSALVMDPPRHTQLRALINRAFGTQVVTRIEPRVREFAQRIVQA
LPDGQEVNFVEHFSVLMPMYVIGELLGLSPEVQPRLLSWVEWLGQFTGVGPADTARQEAV
RATVNEARGHFEQVLTERRRNPGDDLISDLLRARVEGESLSDTDLLGFMFLLLVGGMETT
IHLLSHSALRLQLQPELMRQLREQPSLLPRFIDEVLRHETPAHGVMRLTSEETELGGVRL
PKGTRLLMLMGSANRDEAQHPDPDRFDMNRTQSNLPFGHGIHFCIGSQLARLEARLSLEA
LLSRFTHLTAGTQSIQWNSSLVVRGPTRLSLIPHEN

>CYFUS_002676|KEGG|CYP105EK1|[Cystobacter fuscus]|cfus|DP

MTDSYPLPKDWQHPLDPPPEYKRLRKEAPVCPVRTWDGNTPWLITRYEDVLAALGDPRLS
LDSSLPGFPHTSPASAARQSRPLPFPFLPEAEYRAQRAMLVPEFAPRRMEALRPRIQQTV
DEVLDAMLAGPRPADLISAFALPVSMRVICDLLGVSREDADRLHVISRTIGSRASSREVA
EKALDELDTYFRRLVEENLREPTDTLVGRVVAEHVQPGRLSVPDATAMFHALFYAGHGPS
GYMVGMGVLALLLHPDQLDRFRALETPDALSAAVQELLRYVTVSHMGRQRAATEDVTIGG
QLIRAGEGVLAQPDSANRDETVFPEPDRLDVHREPYRNFAFGHGIHLCTGRTLAIIELEV
TFHTLFRRIPNLRLAAPLEQIRFKKDENLIGAHELPVTW

>CYFUS_003078|KEGG|CYP120G3|[Cystobacter fuscus]|cfus|DP

MTAQADPHAPSPENLPLPPGRTGLPLLGETLDFLRSSRAFTEQRRNQYGPVFRSHVLGSP
AAFLTGPDAIQWIFAGEGKYLKNRWTPGVRRLLGANSLSLLEGEEHLERRRLLAPHFSYA
TMRGFVPAIESLATRHFERWAALPGDFTLWPAMRELAFEIALSLIFGQDTVDVPFLMRHF
QAWTAGLFVPLAVNLPWTKFGRALAAKKAMITYLDQLVAERQKRTEQPPDLLGSLLRHRD
GEEPLPRETIVEELQLLLFAGHDTTVTATSNLMLLLAQHPDVLQKGREAVADMEGPLTLD
GLRAMPYLVQLIHEGMRLIPPIGGAFRVTTRDVAFKGFRIPKGWMVPVSIRTAHAGDHWP
LPERFDPERFGSENNEQRKPGTFIPFGGGPRICLGQHFAMVEMSVMLALLLKHYTWELVP

GQDLSYLLVPFPQPRSGIQLRLRRRS

>CYFUS_003193|KEGG|CYP251V1|[Cystobacter fuscus]|cfus|DP

MDTVTPLPTATAPEVPGGLPLLGHALRLLRLPIAYLQSLREFGDIVQLRIGSSPVYVVTS
PEFIHQVLVTHADSFERGRVFDKATAAIGKGLIVSNGTFHRQQRKLLQPGFHRPRIHGYM
EIIHRQAEAQIAGWSPGARISLREEMHRLTLNAVTRTLFGANTEERIAAEIGDYIDAANS
WVGLHTVLPGRFFERLPMPVNRAFERKKARFDEIINTFIESRRADGRDQGDLLSALLASQ
DDETGKGMSDEQLRMEIAGLFVAGSETTATALTWLFHEVGRHPEVEARIHAEVDAVLGGR
PVTTEDLPRLQYIQSVISETLRRYPIAWLLMRRATRDVELAGTLLPAGAEVIVSPMMVHR
NPQLYPEPLRFDPDRWLPERARELPRHAFLPFGDGKHKCIGDYFARTEMVIAVATIAARW
RLVPVPGHRVWEVPRSVLRPNQVLMTAVPRSAAPVPEENH

> CYFUS_003263|KEGG|CYP117E2|[Cystobacter fuscus]|cfus|DP

MQMQTLSNMLERLPMKETHRLGIPILPGGLPLVGHAPFNLGDTLTFLRAAEAKVGPIFWM
RSFGSEMQLVCMGEPGFELLKNRVTSSEFIREQAPEFIGDALLSQDGARHRNLRTPMSGP
FTPRGLSSSGAATLSAEVIEASVASLPASRPFSLITETQKFALDIIFRVMGIDRSEIQEF
NTNYREFTLGAFPLKLDLPYSPRWRARRGRAWLDARFSSLIAAARGRPEMPGTLSAMLAA
RDTEGQPLKEDDLISNLRLLALAGHETTASTMAWLGLVLAQRPDLWEKLREEATAAPGLP
RSPEELKRHPFAEALFREVIRLYPPVSSTAREATEDLTLHGKLVPKGTMITVPLGTYGYD
PANFPEPEKFDPSRWMGRRIPPSSIETAPFGGGPHFCLGYHLAWVEVVQFATAFARELSR
RGVRPQLAPGVAPPKLRYLPFGQPPKKALIEFVPVV

>CYFUS_003343|KEGG|CYP1284B2|[Cystobacter fuscus]|cfus|DP

MSAFSGKKLDKIPAHVPPELVYEYDNARDPRLLEDPHARMRSLILEAPPIFFTPYNGGNW
VVTRKKAIVDITMNPEVYSNAFLVSGHQSEEHAGEHKEAQQGLMLLPVAADPPRHTAYRA
PLNQPLSAKSVAGLETAIRDMTNELIDKVLAAGRCDFLSDIAEPLPVTLFMKLAGMPTDR
LAEFRHLATQATSATVTPADREVTFKRIAGILAETIKARQEKREEDLISHLLDANINGRN
PTFQEMLGYSIALFLGGLETVVNALSFGVRHLARDQELQAKLRADPSLLPGAIEELLRLY
GIASTVRQVMRDEVCHGVQFKKGDTVSLLLPAANYDDAAFPDPEQFILGRKEQHQTFNTG
PHRCVGLNLARLEMKVFYQEWLKRVPPFRLDPEKKPRFVGGFNLAIASLPLVW

>CYFUS_003436|KEGG|CYP102J22|[Cystobacter fuscus]|cfus|DP

MTPSLLPIPQPRPWPLVGNLTDLDAEQSILSIMELARLHGPIFRLVFFGQSLIVVGSQEL
VNELCDETRFGKLVHSSLRELRAIGGDGLFTAHTEEPNWGKAHRILMPAFGPLGVRDMFA

PMLDVAEQMLLRWERFGAGAVLDVPDQMTRLTLDTLALCAFDYRFNSFYQDALHPFVGAM
VDGLHEAGVRADRPEVLNRLLLPSARRYAEDIRLMHAVADQIITERRRDPDAGQKNDLLG
RMLSARDPVTGETLSDENIRYQMVTFLIAGHETTSGLLSFALYLLLKNPRVLHAARARVD
EVLGGDTPRLEHLPSLRYVEQILMETLRLWPTAPAFAVRAYEDTVIGGRYAITPQDAVMI
FPPILHRDPKVWGPDVEAFRPERFDREAEARLPPNAWKPFGNGQRACIGRPFALQEALLV
LSMILRRFDLIEDDPSYTLKIKETLTLKPEGFRIRARRRDLAALKSRSPVPGAPRGTPAP
EPTPALARTEGPRTPLLVLYGSNTGSAQAFAQRIGSDAPSHGYSVRVAPMDEHAGRLPTQ
GAVIVLTASYEGQPPDNARQFVAALEAAPAGAFAGVKYTVFGCGNRQWARTYQAVPLQVD
ALLEQAGATRLKPRGEADASEDFFGAFDAWYATLWPALAATFGQEAAGTSEAPRLQVEVV
RDNRSTLLRQEDLGAGQLVENRELVNLASPHARSKRHLEILLPEGMTYRAGDYLAVLPRN
PGVSVERALRRFGFAQDSQIIVHKEGARLTALPTGYPIAVSELLTSYVELGQPATRAQVE
TLAKATRCPPEKQPLEALAQEALYRREVLDKRVSVLDLLERFPACELSFAAFLEMLPPLK
ARQYSIASSPLWNEHRCALTVAVVDAPAYSGQGRYLGVTSNYLAALAPPTRVSVAVRPSH
PRFHPPTDPQEPLVMICAGTGLAPFRGFLQERALQAREGRRVGPALLFFGCDHPDADFLY
REELEAWQRAGIVDVRPAFTYAPEGEVTFVQHRVWKDRADVAGFFQRGATVYVCGDGRRM
APAVRETLVRIYEEATQVPREEAEQWALRMEREQGRFVADVFA

>CYFUS_003477|KEGG|CYP262B4|[Cystobacter fuscus]|cfus|DP

MSETTSQTSSDRSLPPRVSSLPLVGCLPRLIANPLSFMEQAQREHGDIFSMDLGFTQVIG
LCHPRYVHHVLVEHAHKYSKGGPMWDSMRTFMGNALPMSEGAFWKRQRRMIQPSFHHQRV
SLMTDTMVEAIDECLLEWDLAALEGKPFDVSVALSRVTMTVLVRTLFGSGLDKDDAEKVA
QAFSFILEYFIAGMVTHSLPEWMPVPGRQRYRESIKMIDEIMRRLIERGREQASGEDNLL
SLLLQAVDGESGERMTNEQLRDEALGFFIAGYDTTAAGMTWVLHALTQHPEVTDKVRVEL
DAVVGTRRPGFADLMRMPYTRNVLQEALRIHSPSVWLPRLSVVEDEIDGYRIPPGVMMVI
FTRLIHRHPGIWDDPLTFDPDRFTPERSEGRHKLAWLPFGSGQRQCIAKEFSLLEGMLIM
ARIVSRYELSSVPGRLPQERVSTNLRTKDGMWLNLRPRLPQAQVPRIQLSGTA

>CYFUS_003785|KEGG|CYP109AK1|[Cystobacter fuscus]|cfus|DP

MQASDYNPLSSTVQADPYPYYAALREHSPIYFNEQLGWYIVSRYEDVVAITKNPAVFSSA
RAIVQPEKLDAAEKVAPISVRSFRRGILIGEDPPTHTKTRNLVTRAFTPKRIAEMEPRIR
QIARELISQLPRSGEFDLIKDLAEPLPMIVIAEMLGVEPERRHDFKRWSDDAIAISFALV
KGAELSGLEHSSREMADYMARALEARRQQPREDLIQALLDNGVREGLLSVDDASAFCRLL
LVAGNETTTNLLGNGMRALLGHPDQLERLTREPSLIPNAVEEMLRFDSAAQALFRKTTQE
VEVSGVRIPAGASVLLLFGSANRDPRKFQDPDHFDVTRNVAGQVAFGHGIHFCLGAPLAR
LEAKVALEELLTPDRQLSLVPGQRLENVAHFTLRGLKSLRLRTEPARSARASA

>CYFUS_003950|KEGG|CYP1335A2|[Cystobacter fuscus]|cfus|DP

MTPPTDPYQAVTHPDPYPYYASLCARGGLQRIAGFEPWIAADAATVRAVLTSEACRVRPQ
AEPVPSHLLGSPAGALFGRLVRMNDGAPSAAVKQALASALPTVMRAVDAESRRWSARLFS
EPRLSLLPEKALQLPVFVLGCLLGFPEEFLAESVREVDAYVRSVTQPGARTEGALGAVRL
VERVTACLQAKPRAPELLGAFSSRMREEGGALEALVPNAVGLLTQAYEATAGLLGAALLA
FARMPALREQLARGECSIDDVVREAARHESPIQNTRRFIHACATVANQELEAGATVVVVL
AAANRDPRVNPQPDVFLPRRPERQTFTFGLGAHACPGRELAVTMVSAAVERVLASGLELT
PLSKSITWRASTNARILGGLQ

>CYFUS_004102|KEGG|CYP167B9|[Cystobacter fuscus]|cfus|DP

MSAQQTNPSENRPEVQFNPYAPGYDVNPHPALEKLRAVAPIFYWEQGRCWVVSRYEDGLT
VLRDDKRFSPNRDDWEFASVLGSDALIPEMEELNKNGLFALGERNHARVRRLVSPAFTPR
ATERLRPEIQAIVDEILDSMAAKGRLNMVHEFSERIPARVIGSMLKIPKGNEELFLDFTN
AVAKSVFAGALAPEELAPLRRQIREGIVLVTETIEDRRRNPQENDILTTLIQTEEQGDKL
NKQELLALVSSLIVGGFETTVHLFSFCVYNLLQHPEVFAELRTRPELTKNLVEEVLRFDN
FVKMGPARYALEDMELGGMPIKKGQMLVVLVGSALRDERAFDKADVFDVQRNASAGIAFG
HGAHYCLGANLARLMGQIAMGTLVRRFPELRLVKQPSFGPHSLMRKMEVLEVDLGSPSA

>CYFUS_004514|KEGG|CYP102M6|[Cystobacter fuscus]|cfus|DP

MSKPSLSTTIPQPRSRPLVGNVPDVGFETPLQNMMKLAREFGPIFRLSFPGENFLRVISS
YELVADACDETRFEKMLGQVLLQLRDLGGDGLFTADAREPNWGKAHRLLMPAFSPAAMRN
YHDGMYDVADQMLTRWARFGPDTDIDVSDNMTRLTLDTIALCGFDFRFNSFYQREMHPFV
ESMVRALAEAGDRTRRVPLQTQLMLRTQRQYQSDLQYMYEVVRELIVRRRALPPEEAPKD
LLGLMLEAKDPLTGEKLDDDNVRSQLMTFLIAGHETTSGLLSFAVYFLLHYPEVLQKAYE
EVDRVLGSEAPRFEQISQLQYIDQILRETLRLWPTAPAFTLHAKADNTLLAGRYPVGVKD
TLMVLIPMLHRDPTVWKNPERFDPERFAPEVRDSIPTHAWKPFGNGMRACIGRAFALQEA
TLVLGTMLQRFHISTPEPYTLRIRETLTLKPDGLKLRVRARKPVSRAPAARRAPTPAPVA
SSSQPTEKTSSHGTPLLLLYGSNSGASEAFARRIASDGLARGYTAKVAPLDEYAGKLPKE
GAVVIVTASYNGQPPDNARAFHTWLSNVPAGALQGVRYAVFGCGNRDWGETYQAVPKFID
ERLSAAGARSLLSRGEADARADFFGDFDYWYAPFWTRVGEGLGVASSEVDAGPRYTVEVV
PPVSAELVKQNKLELATLVDNRELVDMTSPFGRSKRHLVFKLPQGVTYAAGDYLAVLPEN
HPELVERAARRFGVSPDATVILGSARGDQGSSLPTGRPVLVRELLGRHVELSAPATRKDL
ERLAEKNPCPPHAMHLVALARDAERYKKEILDKRVSVLELLEQYTSCILSLGDFLELLPA
MRVRQYSVSSSPLADPTECTLTVAVVDAEAWSGQGRFRGTCSSYLARLRPGEQVAVAVRT
PNVPFHPPASNATPIVLVAAGTGLAPFRGFLQERALRHARGEAAGPALFFFGCDHPEVDF
LYREELAQWEREGVVKVLPAFFRQPDGDVTFVQHRLWKEREQVKALLDQGALLFICGDGR
LMAPAARETLARIQQEKVGCSSEEALAWLAAMEKQGRLACDVFA

>CYFUS_004527|KEGG|CYP167B8|[Cystobacter fuscus]|cfus|DP

MSHEQTKSETKSCPFNPNAPGFDVNPYPMFKELRTQTPISYWDQGHGWIVTRYEDVIAAL
RDNKRFSTNSADWEFASTMGTAALIPERDELNRANLFSLPDADHTRVRRLVSPAFTPRAV
EWLRPDIQAIVDELLDAAEAKGTINVVSDIADPIPARVMSSMLKIPKGREVLFQRFTEAS
IKSLLPSLIPPEELVTVREAIREGIVLIRETIEERRRNPRENDILTTLIQTEEQGDKLST
PELLSLVASLIVGGFETTVHLIGFTLSNILRRPELFAQLKAEPELVKNVLEEVLRHDNFG
KLGLTRYAREDLELGGVKIKKGQRLFLMTNSAMHDEAAFPHPETFDVRRNNTTTIAFGNG
MHFCLGVHLARLEGRIAVDTLLKRFPDMKLVKPPTFGSHPVLRKMETLEVQLRARS

>CYFUS_005195|KEGG|CYP107HV5|[Cystobacter fuscus]|cfus|DP

MPTITPPDFSSQQFKANPFPFYARLRQEAPVYHFTSLTKEPTWLVTRYEDVSQVLKHASL
SKDVFGAAGAERRAQMPWLLKIFEPVSQNMLSKDPPDHTRLRSLVHKAFTPRLIEQLRSR
VQALSDRLLDEAARKGSMDLVSEYALIVPVTIIAEMLGVPPSDYRKFQHWSNRLISNTSL
WDVILSVPSVVMFTRYLRQLIARRRSSLGDDLLSALIQAEEAGDKLNADELVSMVFLLLV
AGHETTVNLISGGTLALLQHPEQLERLRKEPGLIAPAVEELLRYASPVEVSTERFTKEDI
TVGGVTIPRGHLVFAAIGSANRDERQFKDPDTLDLGREPNRHLSFGMGIHYCLGAPLARL
EGQIALQTLVNRFPKLRLATSAQSLKWRTGVLMRGPQRLPVSLS

>CYFUS_005203|KEGG|CYP1347B4|[Cystobacter fuscus]|cfus|DP

MQSTVALVGMDRNPVSPQNLNNPIPLYHELREHEPVHWSEPLQAWVVTRHEDVMACFRDP
RLSAERWKFFEYQIQGLEPETIREFMETTRDQMAMRVGPEHTRVRRQASAGFTPMKLEEM
RACIHHTMAELLEGVRERHGMNLAQEISYPLPTRVIAELLGVPAEDRDRFRRWSDTLAEF
AAPAAGASMLGAARRANQAMMEMKAYFLPLIEQRRAHPTPDALGMMVQAEEVGHMTAGEL
VANAILLMFAGHTTTTDQLSNCVHDLLTHPEQLQVLREEPERVRAAVEESVRFHPAVPFI
FRVAVADVELRGRLIHAGDVVFLGMAAANRDPRAFPEPDRFDITRDHTQHRHLSFGFGTH
HCMGAGLARRELETGITLLLEHLPGLRLDETRPARLKCHSLNFRGFDVLPVRW

>CYFUS_005279|KEGG|CYP107HX5|[Cystobacter fuscus]|cfus|DP

MVEAVRVNLQREFESPEARANPIPVYAKLRKLGPVLHSTALYGEGFVLPRYEEVVSVLKD
PRFANDRRNVPGGTSMDRWWMPAILRLLVSSMVLKDPPDHRRLRNLVQKAFTPIMVENLN
GRVEQITEELLDAAVRKPVVDLMEEFALPLPLTVISEMLGVPEPDRINFRKLIAKVLDPS
SVSPIAFIRNYPHMLRLNRFLRRLVNLRREQPGNDLVTALVQAEEGGDRLSEDELISMIF

LLLFAGHETTVNLIGNGVLALVRHPDQLQKLREQPDLIDSAIEEMLRFTNPVGQVAPRFA
KEDVEIAGVRIPKGSAVTLLIASANLDETAFPNADKLDITRSPNRHVSFGYGIHYCLGAP
LARLEARVAIPALLQRFPRLELAVPAEKLRWRPNIGLRGLEALPLSVSPASSAAERSAA

>CYFUS_005395|KEGG|CYP264A5|[Cystobacter fuscus]|cfus|DP

MTQINLMSPEVLTNPYPYYAELRRLSPVCQVEPHGMWAVSRYEDVLFVLKNPSLFSSSGF
TVAWQPAWVGYNPMANSMITRDPPQHTRLRALLLRAFGPATIARLEPRVRATAERLASGL
VDGADFIQTLALPVPAYVISEILGLDHQLLPYFKQWSDDIVAITPSPASPEAAERIRGTI
AKLTEYVGKVIEERRRAPADDTVSDLLRAEVEGQRLTDVEIKDFLILLLIAGLESTTNLL
GNSLLFLADHPEMMAHLRAEPSLIPSFIEEMLRYDGVAPGVPRLAASDVTLAGVTIPQGS
MVFPLMSSANRDERKFPDPDRFDLHRGSQGGLAFGQGAHFCLGAMLARMEVRIVLETVLA
RFQRVERLLGNIQYQCALTTRGPVALPLRYIPVGG

>CYFUS_005459|KEGG|CYP1011E3|[Cystobacter fuscus]|cfus|DP

MESVARPSVDVKELSTPRRSSIKFNMFDPAFHADPYPVYARLREEEPVHRTIMGAWIITR
YADVKVLLRDNNFGVFDIPTRIERKSPFLERKKIGELRTLASAMRRWLMLCDPPDHTRLR
SLVNRSFTPTAVESLRPRIQETVDQLIARVRSSGRMDIVNDLACPLAVTMIASMLGVPEE
AHSSVTRWADGLSRVLDPLRSLEQYAEMNNVAQEFLAYFRELFAERRRNPKNDLISSLIA
VTDQGDRLTEDEMLSVCMLMFIAGEKTTVNLLGNGMRALLLHRSEFERLRQDPTLIRGAI
DEILRYDSPVQLNTRVPKKDVVLHGQTIPAGDLVYFSLGAANRDPAQFEQPDTFDITRRE
NRHLAFSGGIHYCLGAALALIEGQIAIGTLVREFPDMALVPGETEWMKEIIFRGPQTLPV
TFTP

>CYFUS_006003|KEGG|CYP109R5|[Cystobacter fuscus]|cfus|DP

MDLLSRFDFLNPEVMRNPYPYFAEMREKAPLHYDAKLQAHVLTRYEDVSYILKNPALFSS
ADVRVAGKHQKERSQESGLEGVDSLLTTDPPVHTRLRGKVSRSFTPKQISAMEPRVRELS
RELVAEMTASNEFEFMSGLASPLPVTIIAEMLGVDTSRRRDFKRWSDAIANSGNASFTGK
TPEDVVRNAKEMLAYMTEVAEARRREPRGDLISLLVQENEGQEALTPPEVNSFAILLLLA
GNETTTNLLGNALTALIRHPEAYEWLRRDPTQAACAAVAEETLRYDSPVITVVRRTTQEV
ELSGGKVPANTTLFAVVSAANRDPRKFPDPDRFDPRRDTTGLMSFGHGIHFCLGAPLARL
EAPVALQELMARAPRLGFSPRQPEHIDYGATFALRGPRVLWLQKN

>CYFUS_006004|KEGG|CYP109R3|[Cystobacter fuscus]|cfus|DP

MDLLSRFDFFNPEVMRNPYPYFAEMREKAPIYYDAKLQAHVLSRYEDVSFVLKNHALFSS
TKIRVAGKRQEERASVEELGSVSNLVTTDPPVHTRMRGLVSRAFTPKQISALEPRVRELS
RTLIAEMTASPEFDFMEGLASPLPVTIIAEMLGVDTSRRHDFKRWSDILIHSSRAALQSG
KVSEQVERSAREMLTYMKEVAEARRREPRGDLISLLVQETDGVAALTPPEVNSFTVLLLI
AGNETTTNLLGNALNALIRHPEAYEWLRRDPSPAACAAVAEETLRYDSPVIGLVRRTTQE
VEVSGGKLPEDSTVMVLLASANHDPHKFPDPERFDPRRDTNGLMSFGHGIHFCLGAPLTR
LEAPVALRELMERAPRLGFAPRQPEALDYGGSLFLRGPRSLWLQKN

>CYFUS_006005|KEGG|CYP109R5|[Cystobacter fuscus]|cfus|DP

MDLLSRFDFFNPEVMRNPYPYFAEMREKAPLFYDAKLQSTLVSRYEDVSYILKNPALFSS
AQIRIAGKLQTERNQDAGLEGVDSLLTTDPPVHTRLRGRVNRAFTPKQISALEPRVRELS
QECISEMTAHNEFEFMSGLASPLPVTIIAEMLGVDTSRRRDFKRWSDALVNSSNASITGK
TPEEVIRSAKEMLAYMTEVAEARRREPRGDLISLLVQESEGQEALTPAEVNSFAILLLLA
GNETTTNLLGNALTTLIRHPEAYEWLRRDPSPAACAAVIEETLRYDSPVLTLSRRTTQEV
ELSGGKVPADTTLMVLVSSANHDPRKFPDPDRFDPRRDTAGLMSFGHGIHFCLGAPLARV
EAPVALQELMARTPRLGFSPRQPENIDYGASFFLHGPRSLWLQKN

>CYFUS_006176|KEGG|CYP242A4|[Cystobacter fuscus]|cfus|DP

MTTTTAASLPSYDLFTPEMMANPMPTLHRIRAENPLVWSPQLNAYVLTRYADILAALKDR
RLASSNMANWMDLLTPEQQQELLPVRQSIQLWMGHTNEQDHLRFQLLLKRYFTPGTVDAL
RPRVRQLTEELLDAAEPRGGMDVVEELAYPLPANVIAEMLGMPTRDREKLQAWSRDITAL
FQRSDIHQLRRSQRSVLEMQDYLRPLLEERRRAPSEDLISVLLAASKEGTLSEEEIVSNC
VLLLFAGHETTANLIANGLVLLFEHPDQFEALKARPELMATAVEEMMRCDGPASVISRVS
IEPVEVCGRSFPAGQTFYLALGAGNRDPEVFPDPDRFDITRKPNRHLGFGMGAFYCLGAA
LARMEADECFRILLRRFPNVRPAYQQPDWQVSPPLGHNLKSLQVKF

>CYFUS_006207|KEGG|CYP1387A1|[Cystobacter fuscus]|cfus|DP

MSSPSSPRPEAANASGCPFSARSFNPFAPPQHENMQALFAQARREQPVFFSEAMNAWVVT
RHEDICAAVEDTQRFSSKLDSQIFEALLPEPRAYLADAGYRKMPMIYDDPPEHSRSRQIV
ARLFSKENLAALEPLVRSITEELVDGFAQDRQVELVSRLAYPLPIRVIFAWMGLPLELMD
DFKKWSRHLTLMLSLQARTLELQNECVRGVTDMQRCVAELISERVAHPREDGLTVLAQSL
REGTTLDAADLAAMVMLLISAGHETTSSLMGMAVRVLLEQPERWRQLREEPHTLPRVVEE
VLRYETPMAILARHTATQAELGGVTIPAGARVLLVVLSGNRDERRFPEPEGFDPKRPQLG

QHLAFGRGIHVCVGAGLARLEARVMLEVLARRLPGLRIVEPPRLVPGPVRHQEQLLLAWD

>CYFUS_006425|KEGG|CYP107DL4|[Cystobacter fuscus]|cfus|DP

MKPMDESQKQTVEGVPSPAGTCPFKHLMEAPAPSGGSVVLDRENPEFLTTAYATYARLRE
QAPVVRTLFARGVMDTRKVDSRAGSTADLASSEERLLVTRYDEAVAALLDGSLSSDFRTG
MTPRQIEGLAHMPEEARPLADSLISRDPPDHTRLRKLVQPSFTARAMETLRPRVQRITDA
LLDKAEREAAGRGQAAPDRQMDLLEAFAYPMPITVISDLLGIPEEDRERINHLAELLMNT
DRFDASLAATRRDQTRRFNAYLEELFERKRREPAEDMISQMVHTQEEDGDRLGHEEMISM
VHVLFFAGHLTTVNLIGNGVVALFSHPDQHARMVADPSLAKGMVEETLRYWGPVDYIGTA
RTAIADTQVGGVSIPQETELSIGLGSANRDPSRFPDPDVYDITRPEAHRNIAFGKGIHVC
LGAPLARLEGQIAFETLFRRYPKLRLAVPAEQLKWGAGVGSGVGLRGFARIPVLF

>CYFUS_007441|KEGG|CYP183BE1|[Cystobacter fuscus]|cfus|DP

MRVPTAPARWPLLGHSWPLLSRPRDFLLSLEKVGSLVRVYIGQMPMVVITDPELTRQVLH
DSATFDKGGSLYEKLSELTGNGLLTSQSEPHKRQRRLVQPAFKKSSIKDYSSVMAGVVEV
ALRDWEDDAVVDMKAAAYHIVSRIAARSMFSAEMADASIARIAEALSIYLNGLFVRTVGP
GVLFERLPTPGNLRYKQALEVLHQEISRIISEYIASGVEREDVLSILLREKDEGDGAGLS
PQEVHDQVTTLLLAGIETSAATLCWCLYLLSHHPDVTQRLQAELDGVLGGRAAQWDDIPE
LKQTRRIVMEALRLYPPGWLFTRVTMRDTVLGAYPLPRGTGIAYSPYLLHRSPSSFEQPD
RFDPDRWLPERSNRLSMNSFVPFGGGVRRCIGEMLGVTEVMLTLATIASHWEARCVREGP
IEPAPAHVSLIPKQLRMRVTRRKR

>CYFUS_007462|KEGG|CYP109AK2|[Cystobacter fuscus]|cfus|DP

MQASDYNPMLPAVQADPYPYYATLRENAPVYFNEQLGWYIVSRYEDVIAITKNPAVFSSA
RAVVRPEQLDAAEKVAPTAVRAFRRGILLSEDPPMHTKTRSVVTRAFTPKRVAEMEPRIR
QLARELISQLPRSGEFDLIKDLAEPLPIIVIAEMMGAEPKLRHEFKRWSDNAVATFYALA
RGAELSGIERTSQEMHDYMSQALEARRQQPREDLMQALLDNGVREGLLSVDEAIAFCRLL
LVAGNETTTNLLGNGMHALLSHPDQLERLTREPALIPNAVEEMLRYDSAAQVFFRKVTQD
VEVSGTRIPSGANVLLLFGSANRDPRKFQDPDRFDVTRNVAGHVALGHGIHFCLGAPLAR
LEARVVLEELLTPDRQLSLVPGQHLENVPNFNVRGLKSLRVRTEPAPGTRASA

>CYFUS_008532|KEGG|CYP167B6|[Cystobacter fuscus]|cfus|DP

MSLEATKQSSPAVLFNPQAPGYDANPYPMLDELRTKTPLVYWEQGRGWLLSRYEDAIEVL
RDARRFSPNRDEWEFASVLGSAAMIPELVELSKTGLFALSGADHARVRKLVSPALTPRAI
ERLRPEVQALIDEVLDEAAVKGTINVVSDISDRIPARVIGSMLKIPKGRETLFQRFTEAS
IKNFLPGLLRPEEVEALRADIREGIALVRETIEDRRKNPLPDDILTTLIQTEEQGDRLST
SELLSLVAALIVGGFETTVHLIGFTTYNVLQRPELRAQLKTEPDLLKNVIEEVLRFDNFG
KIGVARYALEDVELSGQTIKKGQMVLIMLNSALRDEAAFPKADTFDVRRNTNASIAFGHG
VHYCLGANLARLEVQLAVGTLLRRYPDMQLVRPPTFAPHPVIRRMETLEVQLRAQ

>CYFUS_008546|KEGG|CYP107DL5|[Cystobacter fuscus]|cfus|DP

MAPNPLDEQQQQTPAQPPREAPFGGPVVLDKESLEFMTRAHAVYAELRDKGPIVRVPSGR
GIVDRAQAERASPGASSPEQYFVTRYDEAVSILMEEKLSSDVLKAMPPEQRARMDAAMPE
ELRPIVRSILVLDPPDHTRLRKLVQPNFTARAMEALKPRIQRIVEDLLDKAEREAATRGE
VSPDRRLDLVESFAYPLSITVISDMLGIPVEERETVYPWAERLLRAKGPEGMMDGETRAG
LTAFANYLESLFARKRQAPAEDMISQMVQVQEDGDILSPQELLSMVFILFFAGHLTTVNL
IGNGVVALLSHPEQHARFLEDPAACVKGMVEETLRYWGPVDFIGGPRIALEDLDVGGTRV
PRGAKVAVGLASANRDPRRFTNPDAFDISRPDAHRHIAFGKGIHVCIGAPLARLEAELAF
EALFRRWPELRLAQPAGQLELSMGAALRGFKRIPVVF

>CYFUS_008576|KEGG|CYP264J2|[Cystobacter fuscus]|cfus|DP

MQPTPDVMALAHRLNPYPLYAELRRDHPVCRVEPGGLVAVSRYKDVEYVLKHPELFSSHG
FRFAWQPAWVGDNPLAQSLAASDGKEHARLRSLVSRAFTPVAINRLEEKVRANAARLADG
LLARGEVDFMEEFATPLPARTISDLLGIDPTLERHYKRWTEVLVSIAPVPENDEHVTRTR
DTIAEMKRYVQQIIDARRSQPGDDVMGLIVQGGPDGQRLGDQEIIGLAFTLLAAGLETTS
FFFAHALPLLAERPDVFERLRADRELLPKFIEEMLRYDGPVRGLPRIVTTDVELSGVRIE
RGTCVLPLIASANRDEARFPHADRFDLDREQPGIPFGAGSHFCLGAFLARLEARAGLDAL
LDRFSGFSLIPEGVVWNRSIVTSGPLKMPVRFLPA

>CYFUS_008681|KEGG|CYP1069J1|[Cystobacter fuscus]|cfus|DP

MCAMISSQLGLEFTPLEPHFLENPFPFYARLRREAPITFAPAFQFWLVSRYADVMTVFKD
PRHYSSRDTLRPPVALPREVMAILEPAGYTSDYPLLGDDPPAHTRLRALVGKAFNQARVN

ALEPRIRQLVGEHLDTLRDGEPRAELISQLISPLAMSVMTELLGLPASDRERIKQWCDDE
KLFFVPIPLEQHLRAARGVADFRRYLRELVEDRRESPRDDLISSLLLARTEGDRPLDTDE
LVALACVLVFAGHETSTNLLATALHHLLRHPGAWDELRKNTYLVRNAVEETLRYDSPVVG
MMRTTTRPVQLGGTALPEGARLFLLFASANRDESVFEQGERFDIHRASAVRHLGFGHGIH
YCVGASLGRLEAQLTLELLLQRLPGLRLAPGEAVTYLPNLVHRVPRQLLVEWDT

>CYFUS_008687|KEGG|CYP107HU2|[Cystobacter fuscus]|cfus|DP

MAERDVNAAQGQPAPQPLPTEVVTLTLDPHISPETYARLRQQGRVARVSFTLGANAAPAG
EKSAELIEFLGHEHLFVTRYEDVIAALLDDRISSDPRTALTEEQKKKLPNVPDELRPLAR
SLLSVDPPDHSRLRKLIQPSFTPRAMESLRSRIQRIAEDLVDGAERAAAERGESRPERRM
DLIAEFAYPLPVKVISDMLGIPEEDREMVKGWTENLVKNNAGRGFRDAESLAKVREFTDY
LRRLFVAKRKNPADDMISQLLRIEEDGDKLNEDETLSTVFILYLAGHVTTVNLIGNGIFA
LLSHPEQLARLKANPSLVKGLVEETLRYWGPIDFISRRIAKEELDLAGTHFPKGEPMMLG
LASANRDPARFAHPEVFDITRPDADKHIAFGKGIHICIGAPLARMEGQIAFETLIRRLPE
LRLAVASEELHWSNSLLRGFAQLPVLF

>CYFUS_008947|KEGG|CYP107DP5|[Cystobacter fuscus]|cfus|DP

MSTSTLTSELWAPQTRTNPLPFYARIRQEAPVVRMIDPYFQTPVWIVTRYKEAVELLRDN
RFTKDQDKLPENSPSRRMRIDSLAAINQHMLSADPPDHTRLRTIVSKAFTPRRVEELRPR
VTAIAQRLLDEAWPQGSMDLLDSFAFPLPVTVIAEMLGVPAEDQDQFREWTNIIINPPVN
GDVGPLQKAGMQFLQYFQQLMARRRAEPRDDLLTALMTAEEQGDRLSPTELVSMLFLLLV
AGHETTVNLMGNGVWALLKHPEQLERLRANPALIDSAVEEMLRYRGPVETTTYRWALQDT
ELYGQVIPAGEAVLASLMAADHDPAQFPEPERFDITREPNRHIAFGFGIHFCLGAPLARL
EATVALNLLLERMPRLRLAVDERELRWREGILVNGLQRLPVAF

>CYFUS_009336|KEGG|CYP102M7|[Cystobacter fuscus]|cfus|DP

MNKPSHSSTIPQPHVRPLVGNVPDVGFETPMQNLMKLARELGPIFRLSFPGGRTSLILSS
HELVADVCDESRFDKQVSGALKQIRDFAGDGLFTAMTEEPNWGKAHRLLMPAFGPAAMRN
YHDDMLDVADQMFTRWERFGPDAVLDVPDNMTRLTLDTIALCGFGYRFNSFYQKEMHPFV
ESMVRALAEAGNRARRVPLQTQLMLRTQRQFQADIGYMHEVTRELIASRRALPPEEAPRD
LLSLMLDARDPLTGEGLDEENIRNQMVTFLIAGHETTSGLLSFATYFLLRHPEVLEKAQA
EVDRVLGDEMPRFEQVSQLHYIDQILRESLRLWPTAPAFSVHPKAEETLIAGTYPIRRDN
TAVVLTTMLHRDPTVWKDPERFDPDRFAPEVRDSIPPHAWKPFGNGQRSCIGRAFALQEA
TLVLAMMLQRFHLVEPAPYELHVRETLTLKPEGLKLRVRVRKPVSRPLASRPTPGAAAPA
PKQEGVASHGTPLLLLYGSNSGASEAFARRIASDGLARGYSAKVAPLDDYTGKLPKEGAV
VLVTASYNGQPPDNARAFHTWISNVPEGSLLGVRYAVFGCGNRDWAATYQAIPKHFDERL

SAAGAEALVARGEADARGDFFGDFEHWYAPFWEKVGAALGVSSRAVSSEPLYTVEVVPSS
STELVKQNKLELATLVDNRELVDMTSPLGRSKRHLAFKLPEGVTYAAGDYLAVLPENHPD
LVERAARRFGVRTDAAVVLHSTRGAMASSLPTDKPVSVQELLGRHVELSAPATRKDLERL
AEKNPCPPHAMHLAALAQDAERYKKEILDKRVSVLDLLEQYTSCLLTFGDFLELLPAMRV
RQYSVSSSPRADPTVCTLTVAVVDAEAWSGQGRFHGTCSSYLARLRPGEQVAVAVRTPNA
PFHPPASNATPLIMVGAGTGLAPFRGFIQERALRHARGEAAGPALFFFGCDHPEVDFLYR
EELAAWEREGVVKVLPAFFRQPEGDVMFVQHRLWKEREQVKALLDQGALFFICGDGRRMA
PAVRETLAKIHQETVGCSEAEATAWLEGMEKQGRLVADVFA

>sce0122|KEGG|CYP109C1|[Sorangium cellulosum So ce56]|scl|DP

MNLFPDEMRRDPYPLYDQLRTGSPVFHVAPLDLWMVFDYDGVKRALTDHEAFSSAVAPPT
GKAPDWIVFSDPPRHTKLRSIVLRAFTPRSIAGLEPRIRELSRDLLDPWIERGEMDLAAD
YAGPLPTMVIAEMLGVPPEDRARLLRFSEVIVNLSHAISGGEEAARAVSEHAVVKEEMKV
YLAGLIEARRRAPAEDLLTRLVEAEVDGERLTEGDILNFFQFLLAAGTETATNLIDNAIL
CFLESPAELFRLRAAPELLPSAIEEVLRHRSPLQMVFRETRRAVEVHGQVIPAGKLVLPV
IGSANRDPHQFHDPGRFDIGRDPNPHVAFGHGIHFCIGAALARLEARIALPDLLCRLKGL
RRASDEPWEPRKAIIVHGPARLPIRFEPGRRAAVHAAGAHTPCPG

>sce0200|KEGG|CYP261A1|[Sorangium cellulosum So ce56]|scl|DP

METARAEVAPSAGMRDLSTLPGPRRLPLLGNLLEIRPSQFHLTLEGWNRKYGDLYTFWLG
RTPVVVVGSAELVQRILRERPKEFRRWSPLQELIDEMGFGGVFSAEGDQWTRQRRLVMSA
FTAGQLRESHGTLSTITRRLRERWRASAARGAPVDARRDLARYTVDVTTAVAFGLDMNLI
ERGTDPLSDQLETVFASLNRRVFAPFPYWRHVKLPADRALDQALSEVRTRMLDILRTTRA
ELDRDPARAAAPRSLLEALLVARDADDPKARLSEQEVLGNVLTLLLAGEDTTADTMAWML
HFMALRPDVQARMRAEVDAELGDADLPESPEHAQRLRYLGAVAQETLRLKSAAPILFMEA
CADTVVGSLRLAAGTRMILLTRQIALKEEHFHDAASFMPERWLTPRPAGAGKHDPRAALA
FGSGPRVCPGRALSMVESAMVGAMVARDFDVSLVDPARPVKELLGFTMKPEGLFVRFPPR
HR

>sce0675|KEGG|CYP267A1|[Sorangium cellulosum So ce56]|scl|DP

MNSPDAPKPDAPPAANPAADADLDPFRLQSPETLANPYPVYARLRQEAPVYFSAAYNGWL
ITRYDQVAAGFRDPRLSAKRSSAFVTKLPDEVRQRLEPLRRNLASWALLLDPPEHTRIRS
LINKAFVPRLVEGLRSRVETLVNELLDAVAPAGRMDVLRDLGDLLPLLVIGEVLGVPAED
RHRLKGWSNALSGFLGAGRPTLEIAGGALSAVAELEDYFRGVIAARRQSPGNDLLSQLIL
AEEQGMILGEQELLSTCCMLLFGGHETTKNLIGNGLLALLLHRSEREALRATPSLIGPAV
EELLRYDSPVQWMSRVALDDIELDGVRIPKGDRAFLVLGAANRDPAQFPDPDKLDFRRTD

IRHISLGLGVHYCAGSALARVEAQAAISTFLRRFPDAELSPGPLTWRMNPGMRGVTALPI
ELGPQSSAS

>sce1588|KEGG|CYP260A1|[Sorangium cellulosum So ce56]|scl|DP

MDFPLANLFFVPSEDATAFGRRLRAAAQQAPIVFDTAFGMPILLRKSHITTAYRDTATFS
TRMFQAGILNGGLAAMQGDEHARMRRVYNMFFLPRAVSQYEERFVRPISEQVVDRLAGKP
RVDLLEDFAMELPRRVIGELFGFPAEKLHETDERVRAMLRGLVRMHDPAAVAESQRAYGE
TLGLITEVVERESRDTSDTLLGEILRTLKAEHMDTIEASRQIVLSLILGGYETTSWLVAN
TIHALLAHPDTLARVRQDPSLLPAAIEEGMRWCPSSFGVLRMVERDVRLDDQALSAGTVV
CLAGIAGNYDETAYPSPEVYDIDRKPLPAANVFGGGAHFCVGAPLARMEARVGLQALLAR
FPGLRAVPEERPSFMYGAKDSVAHGPDKLPVLLH

>sce2191|KEGG|CYP262A1|[Sorangium cellulosum So ce56]|scl|DP

MVGLLPALARDAPSLLRQLARQYPGELIELDLGLTRAYLATHPEQVQYVLHDNWRNFGKE
GGMWKPIGRLLGNGLVTAGGDEWLRNRRRMQPLFSSRQLAGLVDRMFDVVEGDLPRLEER
ARAGAVVDMDKEMMQLTQRVILATMFGVSITPREADSLGEVLLVAIQALNARMFLYFMPD
RLLPGERALRDAIARIDEAILRLVRERRRSKEERDDLLSLLLRARDESGSGMDDRQLRDE
LVTMFIAGNETTAITMTWLFYLLDRNPGIERKLRAEIEEVVGDRRPTAADLSRMEYTKMV
IQEAMRMYPPSWLVPRTVKEDDQICGYPVPAGATVILSQYVMHHDPAFWEAPAEFDPERF
TPERSASRPRYSYMPFGGGPRQCIGNLFSIMEAQIVIAVLLRRLRMRLVPGHPVSPQAVA
TLRPRHGLKMTLHAV

>sce3065|KEGG|CYP110H1|[Sorangium cellulosum So ce56]|scl|DP

MSQLPRGPRSRLLTTYKFLTRPFEFLTHCAATYGDVFNVPVVSGDVVIAGGPRTVQDVMT
APPETFLPFATRAIAPLVGEHSLLMLSDERHRRERKLLTPPFHGDRMRAYAAAMADTAAR
RLTEAARAPRAVAQELTQAISLDVIIRAVFGVEEPSRTSAFARAVVAMTDALTPALTFLP
PLQRELGGLGPYARFRRRVGELDALFRAQIERARAAPGDDILSLMVSARYDDGSRMSDQA
IFDELRTLLFAGHETTALALAWALDHVHRHPGVLARLRDEIDALGPEPDPERLAALPYLD
AVCKEALRVYPIVTESPRLLAQPFRLGEHELLPGTGVAPCILLVHHHPELYPEPSRFRPE
RFLERKFSPFEYLPFGGGHRRCIGAAFAMFEMKIVLGVALSAWEFRLLDERPPRPVRRNL
TLSPSGGVPLVLRARGRSRLYAAA

>sce4633|KEGG|CYP109D1 |[Sorangium cellulosum So ce56]|scl|DP

METETAPSPSPEQIDLSAPSVIADPYPAYRALRGRSPVLYARVPAGGAAGLGEPIRAYAL
LRHAEVLAALRDPQTFSSNVTDKIRVLPRITLLHDDPPRHTHLRRLVSRSFTPRRIAELE

PWIGRLAASLLEATGDGPSDLMGAYAMPLPMMVIATLLGIPAERYVQFRSWSESVMSYSG
IPAEERASRGKAMVDFFAAELEARRRAPSGDLISALVEAEIDGARLDTPEAVGFCVGLLV
AGNDTTTNLIGNMAHLLSERPELYRRAQQDRSLVGPIIEETLRHSSPVQRLLRVTTRPVD
VSGVMIPAGHLVDVVFGAANRDPAVFEEPDAFRLDRPPAEHLAFGQGTHFCIGAALARME
ARIALNALLDCYESITPGEAPPLRQTRAIMPLGFESLPLVLRRSRATA

>sce4635|KEGG|CYP259A1|[Sorangium cellulosum So ce56]|scl|DP

MLSLGRTDHDSFPTAAGELPVLGHLPVLYRDAIGLLRRSRSALGPLFWVNLGFAQRRLFY
VGADALDLLRAPEVVMDHPEHIKGIVGRSMAAQDGPLHRHMRSAINPSFSPQGFAKEAAA
AIASAIAAKIDAWLQQESFAVHPAMRDLTLDIILRICGVPTGDIRAWSRAYRELFLGVYP
IPGRLPGMPAYRSDRARAWIDDRLRKLLALARGGGAAGSLVEMLSNARDDEGEPLSDQGI
VDNLRAIVLAGHETSASILAWIVIVLSRRSEVWSALCDEHAAAPDAPVPMSAREADRLPV
ANAIFREVLRMYTPVWFIFRTVTETITLSGRRIPRNTPLALSPAQFGYDPSLFPDPDRFD
LSRWRDRSIPPGPLELAAFGGGPHFCLGYNVACVEALQLQFILVRALRRAGLVPHLEGAS
PKHVYFPTGHPTAATRVAFRRC

>sce4806|KEGG|CYP260B1|[Sorangium cellulosum So ce56]|scl|DP

MLPRKNLFSFTSKDPSAFGIHLAAAAREHSVYFDEGLGVPVVLRGADVVAVLRDSETFST
RTYDTGIMKGALVTLGGEAHTRMRRLFNAVLSPRVISRYEEATVTPVARRVVERLVRKER
AELFDDFAISMPMGVTSALFGLPEERIAENDALIRKMIRSVVMPQDPVVVAEGRSAHAAM
EAQLREIAEREVAHPSDTLLGEIARAIVAEGLGGVEACEGVVLTLILGSYETTSWMLANL
LVALLAHPDAMNQLRQQPSLLPQAIEESTRWCSSAAGIVRFVEREATIGGETLAAGTILY
LSLIARHYDEEIYPRPETFDIHRRPVGMLNFGGGLHYCVGAPLARMEARVGVSLLLERFP
ALRADPTVQPTFSTAPRGAAAFGPDQIPALLV

>sce4885|KEGG|CYP263A1|[Sorangium cellulosum So ce56]|scl|DP

MSATLERQPPGPGPLASLRYLANFRTGDMRHYEDLFARHGDVVRLRAPGAEDFVMAFHPN
DIAHVLRTNARNYPKGKRYHELAPVLGWGLVNSEGELWRRQRRHVQPQFNHANTLKFVPI
IVEHMEAVLRRWDAQPGEFERDINDDMMDVTFGIAGEAFFGAALHAHTDTVREAFKYALS
IALKRMYSLVNPPLSWPLPSHLRFRRAMDRVHAVIDGIIDGYQRGAGGEDNVLVRLMNSV
DPETGAKMDRAQLRDEIKTILMVGHETSSVTASWALYQLARHPEVCARLTDEIDRVLGGR
SPTARDIESMPYLTMVFNECLRLLPSVPFILRSPLEDDVIGGYKVAAGSTVAIVPWVTHR
HPEFWSDPLEFKPERFANHRRSADDKLAFIPFGAGQRICLGEFMGQLEGKIMVAMALQRY
RLRLVPGFDPRCRGFISLQPVNGMRMILKRREPSSAVRAAPAGREEPAGAEPVVTEPVAA
AMSAHGCPYSARA

>sce5528|KEGG|CYP117B1|[Sorangium cellulosum So ce56]|scl|DP

MTTALPASFADLPLLQGRNPLARRIETRHGFPILPGRFPVVGHMPAIAVDELGLLREGQR
RFGSLFFWAPGVDAWQLIYASNEAFSLFKSKAVDSSYLTEGGVGHLFGQTLMAHDGASHR
RLRTAMNGPFSPKGLDAAEVGAIVATSVERKVRSWLGRRDVQLLRETRELALEVMFKITG
VEDDELPEWRHYYEDLMLVILSLPFDVPGSPRRRGLRARAWLDERIGRILAGVRARGDAK
GLLPALLTARDEQGEPLSEQDLVGNLRLLLLAGHETSASVMAWCVAHLAESPAVWRALRE
EASSAPDLPRSPADLRRFPFAEAVFREALRLHPPVPHDARRAVADFELDGRAVPAGTHVA
IPLVLLSRDPELYPDPDSYRPERWLGRKEALSPLEIAQFGGGAHFCLGYHLAWMEIVQFL
VALGRELPASGPRLQGGFPASRYLPVLRPSGGTRVRFDG

>sce5624|KEGG|CYP266A1|[Sorangium cellulosum So ce56]|scl|DP

METQVAATQPEINIIAPENLRWPYPMFRRLLLEEPVFYDKNTGSWVVSRYEDVNALLRDA
RMSADRYVALADTVPPEQKEMNSYIVKSLSMFMLNVENPTHFRLRNLTNRSFTPKSIAAM
RPSAHAVVNELLDAVQPRGHMDVVADLAYPMPIKFICGILGMPVEDMGLIKQLSDDVSVY
IGSAGKAAGCIPPAYHAIVEFSKLFRPLVEARRKEPKDDLISSMVTTRVDGDSLSDDEVI
ANCILFLVAGFETVTNLIACGTLALLEHPDQLELLKRDSRLMEGAIDEMLRYYPPVNRTA
RLCVEDIPLRGKVIKKGQIVVLMLGAGNRDPSEYPDPDRFDIARENRSRPLSFGGGHHFC
IGSHLARMEGEVALGALLQRMPNLRLATQEVEWRGNSRFRGLRALPVSF

>sce6323|KEGG|CYP264A1|[Sorangium cellulosum So ce56]|scl|DP

MSERVDIMTPAFRADPYTPYAAMRREAPVCQVDPGGMWAVSRYADVATVLRSPERFSSQG
FRAAWQPAWVGHNPLASSILAMDGPDHARLRGLVSRAFGAPAIARIEQRARDLCERLAGR
LDGEVDFIAAAAAPLPAFVISELLGLDHALEPHFKRWMDDLLSVTPEPASAEHAARVRAT
IAELDRYMADVIAARRRSPSDDLVSELARAGELLGDREIIDLLVSILGGGLETTTHFLGS
SMLLLAERPAELERLRASPQLIPRFIEEMMRYDGPTQSVPRLTTSDVALAGVTIPAGSLV
LALVGSANRDEVRFTDPDRFDLHRGQPSLTFGHGAHFCLGAALARMEAKVALEVLVPRIG
EVTRAPGEIPYNRTLTVRGPVSLPLRFRPA

>sce6424|KEGG|CYP110J1|[Sorangium cellulosum So ce56]|scl|DP

MGNLPPGPKSLSLVDTIRAGFISSEPVFRRYAAEYGPTFRLRSPNGILTITGDPEAIRAV
YTADPDEFDVWGVQLTEPVFGTSSVVVTAGARHRRDRRLLAPPFSAGAMRGYGDAIAEIS
LDVASRWRPGRSFSMLAATQAIALDVIVRVVFGVRGEARVGRTREAVLGLIESLSPSFMI
IPALRRDFAGFGPYARHKRAARALDALLFEEIRARRAEGDASQDILGLMMSARHDDGAGM
SDVEIADQLRALLFAGHETTAMSLGWAMYWLHREPAVLARLLDELDTLGPAPEADALASL

PYLEAVCLEALRLHPPVVDVARVVKRPFRLKGYTIPAGEAIAASPLLLHGREDLYPSPER
FRPSRFLDRKFTPFEFIAFGGGARRCLGAAFAMYEMKVALGTILGRYRLRLESQAPIHHV
RRGLTMGPSGDVAMILEGERERARAEGRPAAVQGSPAEPALRPAAPQPAGRCPMGFS

>sce6630|KEGG|CYP266B1P|[Sorangium cellulosum So ce56]|scl|DP

MGQAPATCTAASPVAAPSSRGVAGRARSTGAYRSCPSPAATDWTGHGHDRRRGGSGVRRN
CHLEPDRFDITRQQSRHLTFGSGAHYCPGASLIRMEVEESVRALLSLPRWELAEETLSYA
GSNLQDRGPSSLRVRFPAACSGRARPIGRRAAGR

>sce7867|KEGG|CYP125E1|[Sorangium cellulosum So ce56]|scl|DP

MTPQTVDILDPDLYLAGAPHDRFELLRREAPVHWHPEPAGRGFWAITRHADVARISRDPA
AFCSGRGLFIEDLPPGDMRDNPDVMIMMDPPRHARFRALVSKGFTPRVIQRLESHVRELV
TRLIDDACERGGCDFASDIAGKLPLSVILEVIGVPREDQEQMLDWTTRFFGASDPAYGVT
PEELNAVLHNMNAYAHQLAEQRRKEPKDDMLSLLMAAEVDGEKLSYTEFGGFFNLLLTAG
HDTTKNLISNGMLALLEHPDQRRRLLDDPSLLPTAIEEMLRFTPPVYYFRRSAVRDIELH
GQRIAAGDKVVLWYVSANRDETVFSDPHRFDVGRTPNEHLSFGIGPHVCLGLVLARLEAR
VAFEELLRRLPDLELAGPAVRLRSNWVSGVKSMPVRCARPAR

>sce8224|KEGG|CYP265A1|[Sorangium cellulosum So ce56]|scl|DP

MSTPHTLQPDDLMEEGGDVVRRAQEACWYAETSMGKAVLRYREVHDLLRDARFRNMGTDV
LTRQGVTEGLVRRMYEGFLLSTEGAAHARLRGLLKKGFTPDAVDALRPRMRERMHALLDE
IGDRTEFDFVTAVAERFPGQVIIELLGLPVSFEDPDFRRWCSDLGYIVGLEVKKHLPRLE
AAFEGLRGYIEPTIAARRAEPRGDLISTLAAAEQNGDRFSEEELVCQIVALLFGGLDTTR
RTIGKAFLVMLEHPDQRRLLQERPDLAEPAAEEILRYAPGTRVAVRVATCDLELHGVPIR
AGEMVIAATGAANRDPRVFEAPETFDITRRGAAPLSLGAGPHHCLGAYLARAELQEAVPI
LTRRLTEAALASPVVVRTAREGSPGPRVIPLRCRVRPR

>sce8551|KEGG|CYP264B1|[Sorangium cellulosum So ce56]|scl|DP

MTRLNLFAPEVRENPYPFYAALRRESPVCQVDPNGMWVVTRYDDIVAAFKNTQVFSSAGL
RMATEPPYLRRQNPLSGSMILADPPRHGQLRSISSRAFTANMVSTLEHHMRSMAVRLTDD
LVHRRVVEFISEFASRAQVSVLAKLIGFDPGLEGHFKRWATDLVIVGVIPPEDHARIAEV
RRTIDEMEQYMLGLLASRRRHLENDLVSELLRSRRDDDGITDQDLVSLLSLLLVAGLETS
TSLMTHMVLILAQRPMWMDRLRAEPALIPHFIEEVMRFEAPVHATMRLTVTETELGGTRL
PAHAVVALLISSGLRDEARFQEPDRFNPERGDQANLAFGHGAHFCLGVFLARVQARIVLE
ELLRRCHRIVLRTDRLEWQAALNTRSPVALPIEVIPVSTTAARESPVVQGIW

>sce8913|KEGG|CYP109C2|[Sorangium cellulosum So ce56]|scl|DP

MNLFSEEMRRNPYPAYDQLRSRTPVLHYPPSDSWLIFDHDGVKRALHDHDAFSSVVSPPG
TRTAEWLIFSDPPRHTKLRALITRAFTPRAVAGLEPRIRRLSRELLDRTIESGQMDVAED
YAVPLPLLVIAEMLGAPAADQPHFKRWSDAILDLSHTVSGSEEAARALDAFTAVTAEMQA
YLRGLVEQRRAAPEDDLLTRLVEAEVDGERLNEDEILGFFQLLLVAGHETTTNLIGNAML
CFLENPDELARLRAAPELLPSAIEEVLRYRSPVQAMFRVTRRDVPMHGQVIPAGKAVLAM
IGSANRDSQQFRDPDRFDIARDPNPHIAFGHGIHFCIGAPLSRLEARVALADLVARLGGL
ERASDAPWEPRRAIHVHGPARLPIRFAPGPRLGGAPG

>SCE1572_00630|KEGG|CYP109C3|[Sorangium cellulosum So0157-2]|scu|DP

MNLFPDEMRRDPYPLYEQMRSSSPALHIAPFDLWMIFDYDGVKRALTDHDAFSSAVTPPT
GKAPEWIVFSDPPRHTKLRGIVQRAFTPRSIAGLEPRIRELSRELLDQRIEHGTMDLAVD
YAGPLPTMVIAEMIGIPAEDRARFMRWSEAIVNLSHTLSGGEEAARVVSEHAAVKEEMKA
YVADLLEARRGAPEDDLLTRLVEAEVDGERLNEDELLNFFQFLLAAGTETTTNLIDNAIL
CLLESPSELARVRAAPDLVPSAIEEVLRYRSPLQMVFRETRRAVEVHGQAIPAGKLVLPV
IGSANRDSRQFRDPGRFDVARDPNPHVGFGHGIHFCIGASLARLEARIALPDLLTRLRGL
ALASDEPWEPRKALIVHGPARLPVCFEPGRRAGAGAAGAHAASP

>SCE1572_04555|KEGG|CYP267A2|[Sorangium cellulosum So0157-2]|scu|DP

MNSPDAPKPDATPVATPAADADLDPFRLQSPETFANPYPVYAKLREQAPVYFSAAYNGWL
ITRYDQVAAGFRDPRLSAKRSGAFVTKLPEDVRQRLEPLRRNLASWALLLDPPEHTRIRS
LINKAFVPRLVEGLRSRVEALVNELLDAVAPTGRIDVLRDLGDALPLLVIGEVLGVPTQD
RHRLKGWSNALGGFLGSGRPTLEIAGGALSAVAEMEDYFRGVIAARRQNPGSDLLSQLLV
AEEQGMILGEQELLSTCCMLLFGGHETTKNLIGNGLLALLLHPAEREALRESPALIGPAV
EELLRYDSPVQWMSRVALDDIELDGVRIAKGDRAFLVLGAANRDPSQFPDPDRLDFRRTD
IRHISFGLGVHYCAGAALARVEAQAAISTFLRRFPDAALSSAPLTYRQSPGIRSLTAMPV
ELGAQSSAS

>SCE1572_16700|KEGG|CYP110AD1|[Sorangium cellulosum So0157-2]|scu|DP

MTTLPKGPSNNILVNIQGMLDPIGYTLRMRERYGDPMSCPKMNGKPVLATGSVEGLRSVF
AVPPENLDQMLGESFTAAVGESSLFSLSGARHAAMRKLLMPPFHGQRMRLYGKQICDLTL
QCSQDFKPGLQLVAQDLMHKISLQTIIHLVFGVTTPAEAARLEELLEELRKSFSFGLTIT

LIIPWLRREFRGFGPWARRQRATRDLRSFLDREVARRRAEPAERTDILSLLLEARQEDGT
ALDDQQIFEQLHTLLFAGHSTTAGALSWVLYFLGHAPAALRRLQDELTALGADPEPEALA
KAPYLEAVCNETLRLRPPSPGVGRKLNAPMQIAGYELPAGAGVFAQIIWAHHNPAVFPEP
AVFRPERFLERTYSPFEFLPFGGGNRRCIGAAFALYEMKLILGTLLRRYSFELVSKKPVR
VGLQGLPGSPVRVIVR

>SCE1572_18480|KEGG|CYP110H2|[Sorangium cellulosum So0157-2]|scu|DP

MAPDAAAAPPRRPHRWIDPGEAPRATGIELLPRCAMSQLPRGPQGRLLTTYQFLTRPIEL
LSRCAATYGDVFNVPMYNGDVVMAGSPASVQDVMTAPPDALVTYATKSIAPLVGEHSLLM
LSGERHRRERKLLTPPFHGDRMRAYAAAMADVAARRFAEAARAPRAVAQDITQAISLDVI
LRAVFGVEEPSRMSAFARAVVAMTDALTPALTFLPVLQRELGGLGPYARFRRRVDHLEAL
FREQIERARAAPGDDILSLLVAARYDDGSSMSDQAIFDELRTLLFAGHETTALALAWALD
HAHRHPAVLARLRDEIDALGPEPDPERLAALPYLDAVCKEALRLHPIVTEAPRMAAQPFR
LGEHTLPPGTGVAPCILLVHHRAELYPEPSRFRPERFLERKYSPFEYLPFGGGNRRCIGA
AFAMFEMKIVLGVALSAWEFRLLDERPPRPVRRNLTLGPAGGVPLALRARGRAALRTAA

>SCE1572_24990|KEGG|CYP167A1|[Sorangium cellulosum So0157-2]|scu|DP

MTQEQANQSETKPAFDFKPFAPGYAEDPFPAIERLREATPIFYWDEGRSWVLTRYHDVSA
VFRDERFAVSREEWESSAEYSSTIPELSDMKKYGLFGLPPEDHARVRKLVNPSFTSRAID
LLRAEIQRTVDQLLDARSGQEEFDVVRDYAEGIPMRAISALLKVPAECDEKFRRFGSATA
RALGVGLVPQVDEETKTLVASVTEGLALLHGVLDERRRNPLENDVLTMLLQAEADGSRLS
TKELVALVGAIIAAGTDTTIYLIAFAVLNLLRSPEALELVKAEPGLMRNALDEVLRFDNI
LRIGTVRFARQDLEYCGASIKKGEMVFLLIPSALRDGTVFSRPDVFDVRRDTSASLAYGR
GPHVCPGVSLARLEAEIAVGTILRRFPDMKLKETPVFGYHPAFRNIESLNVILRPSKAG

>SCE1572_32890|KEGG|CYP105AC39|[Sorangium cellulosum So0157-2]|scu|DP

MIMTESVHTVGLLPTAREPGCPFDPPNELRQAREHGPISRYPFPGGYEGWLITGYDLVRS
VLADSRFSSRKELMRHPLIDLGDMKIPPAPPGEFLLMDEPEHGRYRKLLTGKFTVRRMQL
LTERVEQVTTEHLDAMEKAGPPVDLVTAFAKPIPTIMICDLLGVPYETRDSFQEHVDAIF
SGVSKQDQVAGYVATQQYIAQLVAAKRANPTDDVLSDLTDTDLTDEELMGVGLLLLAAGI
DTTANMLALGTFALLSNPAQWATLRARPEIADLAVEELLRYLSVAKTFQRTALEDVELGG
QTIKAGTTVILSYNTANRDPERFDNPHALDIRRQAIGHLAFGHGIHVCLGQQLARIEMRV
ALPALVQRFPTLRLDVAAEDVDLRPETADIFGVNSLPVTWNA

>SCE1572_38035|KEGG|CYP110J2|[Sorangium cellulosum So0157-2]|scu|DP

MGSLPPGPKSFSLVDYIRSGFISSEPVFKRYAAQHGPTFRVKSPNGMLTITADPEAIRAI
YTADPDTFGVWAAELVEPVFGTTSVVVTTGARHRRDRKLLGPPFGAGAMRGYGEAIADIS
LGEASRWRPGRSFSMLAATQAIALDVIVRVVFGVRGEERVGRTREAVLALIDSLSPSFFV
LPALRREFGGFGPYARNRRAARALEALLVEEVQRRRAAGDASQDILGLMMSARHDDGGGM
SDVEIADQLRALLFAGHETTAMSLGWAMYWLHREPAVLARLLAEIDALGPSPAPDALASL
PYLEAVCLEALRIHPPVVDVGRVAKRPFQLGRYTIPAGESVAASPLLLHGREDLYPSPER
FRPSRFLERRFSPFEYIAFGGGARRCLGAAFAMYEMKVVLGTILGRYRLRLESQAPIQHV
RRGLTMGPSGGMAMILEGERERPRAAGGGAAAEGSQAEPALQGVASGPAARCPMGFS

>SCE1572_50855|KEGG|CYP109C4|[Sorangium cellulosum So0157-2]|scu|DP

MNLFSDEMRRNPYPLYDQLRSGSPVLHYPPSDTWMIFDYDGVKRALHDPEAFSSVVSPPG
NRTSEWLLFMDPPRHAKLRALLQRAFTPKAVAALEPRVRELSRGLLDRAIERGEMDLVED
YTVPLPLLVISEMLGAPIEDQPRFKRWSDVILDLSTTVAGTEEGARAIAAFTAVSAEMQA
YLRGLIAERRAAPRDDLLTRLVDAEVDGERLGEDEILGFFQLLLVAGHETTTNLIGNAVL
CLLEDRAALARVRAEPALLPAAIEEVLRYRSPVQAMFRVTKRDVPMHGQVIPAGKPALVM
IGSANRDPQQFRDPHRFDIARDPNPHVAFGHGLHFCLGAPLSRLEARIALSDLVTRLADL
RLASDAPWEPRRAIHVLGPTRLPIRFAPGPRLDRSET

>CMC5_001820|KEGG|CYP102M3|[Chondromyces crocatus]|ccro|DP

MSDLPAQSVIPQPPVRPLLGNAPDIDTDSPVMSMVQLSERLGPIYKLAFPGRSMLVLGNH
ALAAEAGDEARFGKYVGGVLNELRALGGDGLFTADSNEENWGKAHRVLMPAFGPAAMRNY
FDDMVEIADQMLVKWERFGPETDIDVADNMTRLTLDTIALCGFNYRFNSFYQREMHPFVA
SMVRALLEAGYRAKRLPLQNQLMFLSRRQFEADNAFMHGLTDELIEKRRKLDQREAPRDL
LGLMLNAKDPLTGEGLDDKNIRYQLVTFLVAGHETTSGLLSFALHLLLDNPEVLEKAQAE
VDRVLGDEAPRHEHLAQLVYVDQILRETLRLHPTAPIFAVHAREETTLGGRYPIHPEQAL
MVLLPSVHRDPAIWKHPERFMPERFAPGVRQEIPHRAWMPFGNGQRSCIGRAFSLQEATL
VLAMLLQRFEITRPRRYRLVIKETLTMKPEGLVLRARARKPAVRGLAARPAPRAEPREDT
KTKVRAPAHGTPLLLLFGSNSGASEAFARRIASDGSARGYATHVAPLDDHAGKLPREGAV
AIITASYNGQPPDNARQFCTWLDGLPEGALSGVRYAVFGCGNRDWAATYQNVPRRVDEQL
ARAGATVLLPRGEGDARADFFGDFEQWYAPFWGTLGEALGVAPQEIDMSPLYEVEAVPSG
SAELLAQNQLALATVIANRELVDLSSPFGRSKHHIEFTLPEGASYAAGDYLAVLPENHPD
LVERAARRFGVRTDAAVVLRSTRGAMAASLPLGKPVSIQELLGRHVELSAPATRKDLERL
AEKNPCPPHRAHLAALVGDLDRYKAEILDKRVSVLDLLETYPSCIVSFAEFLEMLPAMRV
RQYSISSSPRLDPTRCTLTVSVLSAPAYSGNGQFHGTCSSYLARLRPGDRIPVAVRTPNV

PFHPPASNETPVVMICAGTGLAPFRGFLQERADRYAHGEAVGEAVLYFGCDHPDVDFIYR
DELMQWERDGIVDVRPAFFRKPDGEVTFVQHRVWQERERIRDLYEQGAIFFLCGDGQRMA
PAVRATLVRIRQEAVGCTEEDATAWLTSMEREGRYVPDVFA

>CMC5_008210|KEGG|CYP110R2|[Chondromyces crocatus]|ccro|DP

MVALPPGPPLAPLHTLFAGLDTPRFFKSCVSRYGDPFTVTLPAGKVVVTGHPDGIREIFT
AEPSTFGALTQIPLLPVLGKSSMLLLEGQAHKRERRLLMPPFHGDRMRAYGTLMRDITLR
LAATLPSHGAFTAMQLTQAISLEVIIQAVFGVKQPERVKAFQDTIASYFSSYTVPLMFVP
PLRRSFHGVSPWDRFQQTAHRFDILLDEEIAARRRSSVEHEDILSLLLSARDEEGTPMTD
EEIKDELRTMLIAGHETTAITMAWALYHVHRLPAVKARLLDELDALGPDPSPDALARASY
LGAVCDEALRLDPVVILVPRRLKAPMTLRGVALPAGIGVMGAIVLAHHDPTIFSEPDQFK
PERFLDRKYSPFEYLPFGGGARRCLGAAFALYEMKIALGSLLLAHRFSLDEQGPVKAVRR
NVTFGPKTGVRLRHDGVRPRPASPANPGLKGAGEGAAA

>CMC5_010050|KEGG|CYP107HV2|[Chondromyces crocatus]|ccro|DP

MRIHVPDLCSAQHKSNPYPVYARLRAEAPVMRIVFGDKKPAWLLTRYDDVAAALKDPRLA
KNAYRTLTPEEQAKAMPWTPGFLRPLTTSMLDQDPPDHTRLRTLVHKAFTPRAVESLRER
IEVLTAELIARARARGSIDLLEELAVPLPITVICEMLGVPEADRARFRAMSNRLIGIVTP
TDMLLAMPSMWMLMRYLRGLVESRRRSGGNDMLAALIDAEEAGDRLGEEELLSMVMLLLL
AGHETTVNLIASGMLSLFDDHEALERLRSEPSLIKPAVEELLRHTSPVDLATERYTTEEV
AFSGHVIPKGQRVFAVLGSANHDEQMFAQPERLQLDRDPNRHLAFGSGIHYCVGAPLARL
EAQIAFTAILKELPGLRLAVPRASLRWKPATILRGLDQLPVHCTPAPTCSIPRAKPTAAA
TAPLG

>CMC5_013000|KEGG|CYP264H1|[Chondromyces crocatus]|ccro|DP

MNQQIDLFMDQNTRRNPYPVYAELRRNRPVCQVKGGIWVAARHADVMRIAKDVTSFSSSG
FRVILSPPTIGPNPVAESILALDPPNHTQLRALVSRAFTPRNIGRLEGRVTDIANEIGDR
LRDMGEADLVAELAGPLPARIIAEILGLDPALHREFKRWGEHLAVITPATVDPLASAIRS
SIGEMERYFKEVIAARRRNPREDIVTDLTRAEVDGVKLSDPQIISFLFLLLSGGFETTVN
LIAHAMMILSARPQDHDRLRAEPHMIPAFVEEVLRYEPPLQGLPRVCSADVVIGGQFIPA

GSLVVGLIGSANRDESLYHDPDQFDMDREQHGALAFGHGIHFCLGAALARLEGRLSLEAL
LSRFRRLQQLPGEITYTCAISLRGPIAVPMRFIPG

>CMC5_016940|KEGG|CYP183AH2|[Chondromyces crocatus]|ccro|DP

MDATSEIGIAPGGLPFLGHALQMLRRPLDFVASLRGQGDVVKIRVGAEWAYLVRHPDLVR
QVLTDIFTFDKGGALFDKAKQIVGNGLSTCPASDHLRQRRLLQPAFSRERLENYAATMSE
VTREMVDAWQPGDIVDVHKAMGKLALSIATQTLVSTSAVSASQETLKDSIQVLLDGIYWR
MIAPFELLQKIPTRHNREFALAQQRMGAVVDQMAEEYRKSGVDRGDLLSALLAARDTDGT
TGMTNAEIRDQVLTLFLAGVESNGAAMAWAFYLLGRHPEVERQLHAEVDEVLGGRVPSFA
DIPKLAVTHRIVQETLRLYPPIWMVSRRTTAAVKLGSCAIPAGAGILFSAYAMHHDPDLF
DEPERFDPDRWLPERARSLPREAMLPFSSGKRKCIGEAFSVIEATIAVATVARRFRLRSV
RAGTIKGIPRVTLIPGPLPMRVEGRKPKTMA

>CMC5_016950|KEGG|CYP183AH1|[Chondromyces crocatus]|ccro|DP

MDTSSEIGVAPGGLPLLGHALQMLRHPLDFLTSLRGTGDVVKIRLGPEWAYLVRHPDLVK
QVLADAYTFDKGGIVFDKARNILGNGLATCPAGDHLRQRRLLQPAFSRERLINYAVCMRD
AARTRVESWHDGQVVEVDREMGSLALTVATRTLFSMDSVAESPEEIQASIQTILSGIYRR
VITPFEVVLRVPTRKNREFDQAMARMYSVIHQITEDYRTAGIDRGDLLSALLAARDEDGT
TGLTNREIRDQILALFLAGIETTAAAMTWSFHLMGHHPEVEARLHAEVDTLLGDKPPGFE
DVPKLTHAHRVITEALRLYPPLWLLTRRTTAEVKLGNHLLQPGTAILFSSYAMHRDATYF
EHPERFDPDRWLPERAKAVPRGAMIPFSAGKRKCLGDTFGMTEATIALATVARRWRLRPV
PGKRISSIARITLAPGAVPMRVERRS

> CMC5_019510|KEGG|CYP101P1|[Chondromyces crocatus]|ccro|DP

MNNELESERGVPAHVPVDLVVDLDVFHPAGMEDDLHLAWRKVQVSGPDIFWTPRNGGHWV
ATRAEDIVVIQTDHERFSHRDVSIPTARPKGAPTALPVELDPPQHGAYRGLISPAFSIRA
VGAMEAKIREVTVALVEAIAPRGECEFVGDFAKELPVVVFLDLVDLPRADRETLLPWAED
LIRPKSAETAGLAYQNLARYVAGWVAQRTERPGNDLISHIAGSRVHGRPITQDEAIRLCM
LVLVGGLDTVASMLGFVARFMAQHPVHRRQLAEDPTLLPQAVEELIRRHGLVNTTRTITH
DFVFKGVQLKEGERILVPNHLAGLDERTVEAPLTVDFLRPKPAPHAAFGNGPHRCPGSTL
ARTELRIFLEEWLKRIPEFGIKPGTKPVFASGAVNTVHELHLGWTPARG

>CMC5_023260|KEGG|CYP264G1|[Chondromyces crocatus]|ccro|DP

MSEVHLNLLSPEVAVDPFPFYAALRRDSPVCRVEPGGFWAVSRYEDAAAVVADPVRFSSR
GVAPAMDPPWLQLEFNAGAHSMVVQDPPEHTRLRSMVNRAFGPALLAATEGPLRALAERL
ADGLGGGDEVDFVSAFATPLVASVIGGVLGLDPTRHAQFKRWSDVLGSAGPVSQGPEHDE
LVRSAYREMHRHCDEVIEARRREPGSDLMSALLQIRAGEQALTDRELHSFFHLLFIAGLE
TTVHLIAKSVMRFADHPEEMARLRAEPAMIPRYLEEMLRYDPSVHFIFRQAPAEATLAGG
TIPAGSFILVMLASANRDEARFPKADVFDMDRDTRGHLAFGHGPHTCLGLALARMEARIA
LETLLTRFQRFERRPGPPEWMHTIIVRGLARLPVRAIPA

>CMC5_023750|KEGG|**CYP263A3**|[Chondromyces crocatus]|ccro|DP

MTEMREEKRPPGPGPLTSLRYLANFRTGNMRHYEALFARYGDVVRLRAPGAEDFVLAFHP
RDIGHILRTSSRNYPKGKRYHELVPVLGWGLVNSEGDLWRRQRRLVQPQFNHAGTLGFVP
LIIAHTEALLRRWDARPDEFERDINDDMMDVTFGIAGEAFFGAALHAHTDTVRTAFKFAL
SMALKRMYSLVNLPLSWPLPSHARFHHAMGQVHAVIDEIIDGYQAGDGSPDNVLVRLMKA
VDPETGEAMDRAQLRDEIKTILMVGHETSSVTATWALYLLSQHREACERLTEEIDRVLSG
RTPTVEDLDAMPYLGMVFNECLRLMPSVPFILRSPLEDDVVGGYRVEAGSTVAIVPWVTH
RHPAFWNAPEKFMPERFAESRKSAEEKLAFIPFGAGQRICLGEFMGQLEGKIMVTMLLQR
YRFQLIPGFDPRCRGFISLQPLNGMRMICRRREQTRSASVRRGGDGVRESVARPSRACPF
SAGS

>CMC5_027320|KEGG|**CYP109Q2**|[Chondromyces crocatus]|ccro|DP

METRVFAELDVTSPEFIRHPYPSYARARRASPLCRIQPNQFLGVSRYHDVVRVLHDSKRF
SNSGYTASRPAQRQDLDIMPPMLVELDPPRHGKVRALIVRALSPRTVAEFEPRLRKITDE
LMASLHGESEFELLSALAIPLPMIVIAELLGVGSERRDDFRRWADDFAGAQAIGAALETE
KLQRSTQEFYHYFGERMEERRREPRGDLLSQLVHAEVDGERLSAEELLSVVTALLIGGNE
TTTSLVANTIVTLTDHPEQLAEVQENLALIPALVEEALRFEPPAHCIFRQATTDVELEGA
VIPRGTVVLPLLASANRDESQFPDPDRFDIHRDTKGHLAFGLDIHFCLGAALARMEGRVM
LEALLSRTKNLKRAERDVAWTPSFFIRAPQKLVLRAQVD

>CMC5_027330|KEGG|**CYP109Q3**|[Chondromyces crocatus]|ccro|DP

MSTGSADSAELDVTTPDFIRHPYPGYARARRTASLCRILPNQFLGVSRYHDVVRVLHDSK
RFSNSGYTASRATQQQNLDIMPPNLVELDPPRHGKLRGLIVRALSPRSVAEFEPWLRKIA
DGLLESLHGASEFELLSTLAIPLPMIVIVEMLGVGTERRDEFRRWVDDFAAAHAIGSTLK
REQLQRSTQAFCHYFSAQLEERRREPREDLLSQLVHAEVDGERVTAEELLSVVTALLIGG
NETTTSLIANALVTLTDHPDQLAEVQENPALIPAFIEEVLRFESPAHIVFRQTTTDVELD
GVTIPRGAMVLPMLASANRDERQFPDPDRFDIHRDTKGHVAFGLDIHFCPGSALARLEAR
VMLDALLSRAKNLRRPEREVAWAPSFFLRTPQKLVLCAQIA

>CMC5_027340|KEGG|CYP109Q4|[Chondromyces crocatus]|ccro|DP

MFAELDVTSPEFIRHPYPGYARVRRSSPLCRIQPNQFLGVSRYHDVVRVLHDSKRFSNSA
YTTSRPAQRQGLDTMPPMLLEMDPPRHGKVRALLVHAFSPRIVAEFEPRLRQLTDELMAT
LHGAQEFELLSTLAVPLPILVIAELLGIGSERRDDFRRWSDDFADALSIGATVETEQLQR
STQEFYHYFGERMEERRREPRGDLLSLLVHAEVDGERLSARELLSAVTSLLVGGNETTTS
LVANALVTLTDHPEQLAEVQENLALIPALIEEVLRFEPPAHCLFRQTTRDVEVAEMVIPG
DTIVLPLLASANRDASHFPDPERFDIHRDTRGHLAFGLNIHFCLGAALARMEARVMLEAL
LSRTKNLKRAEREVAWSSSFLIRSPRRLVLRAQIG

>CMC5_027350|KEGG|CYP109Q1|[Chondromyces crocatus]|ccro|DP

MIADFNITSPEVARNPYALFTEMRRASPVCRLQPIQLLSVGRYHDVVRVLHDSKRFSNSG
YNASVPKEMQENSTMPPSIVQVDPPRHGQLRALVTKAFTPRTVAQLEPRIRELSRELIDG
LRGQSTFELTSAITIPLPMIVIAELLGVGPERRGDFKRWSDDFVSTLALVEPGNRERVRR
STEEFYGYFGQVLEERRKEPRDDLISRLLLADVDGSRLTPEEVLSFANTLLIGGNETTTS
LIGNAMVALTDNPDQLAEVQENPALIPALIEEVLRFESPAQCIFRQTTTDVDLDGVTIPR
GAVVLPLLASANHDESRFPDPDRFDIHRDTKGQIAFGIDIHFCIGAALARLEARVLLEEM
LGRMKNIQRAEKDVAWTPSFFIRSPQSLPLRARID

>CMC5_030120|KEGG|CYP1011F1|[Chondromyces crocatus]|ccro|DP

MSQRLHFDPLSSEFLADPYSLYARMRAEDPVHCNAMGSWLLTRHDDVVAVLRDHRFGVHS
MAANLRSKGRFLGPGQKLDALADTVGQWLMFDNPPEHTRLRRLVSRSFTTGSVELLRAQV

EERVAACLDAARERGELDVIHDLAVPLAVGTISSILGVPAKDRARVLAWTEGLSHIVDPL
RSLEEYLAMEQVAEEFMEYFRALFRERRRNPEDDLVSALVAKEKDKGVTEVELLSMCTNL
FTAGYKTTVNFIGNGVLALLRNPAQCALLREAPEAVPRAMEELLRYDSPVQLITRLACEE
VSLRGRTIPAGSMVFLALGGANRDPEQFANPDRLDLTRTEVRHVAFAPGMHHCLGAMLAQ
LEGQVAILGLVQRFEDLALRTEGVAMESDVIFRGPRSLPVSFDARTQALRRGRGG

>CMC5_034440|KEGG|CYP210B1|[Chondromyces crocatus]|ccro|DP

MALPPGPRTPALVTTYHWIRRPQPFLTECRDTFGPAFTIRLPNLPPVAIFANPDDVKDIF
TGDSDTMLAGRFNLSLRAFLGDHSVLMLDGREHLRQRRLLLPPFHGERMTAYGRIMIDAA
HDAIDRFPFRSPFAVHGYMQEITLHVILRAVFGLEEGPRLDALAEAVTNLLEIAVWPPLL
LPAMQRDLGPWSPWGRFLHYKRQTHGMLLDQIRRRRAEQATRPEGLEGDDVLTLLLQARD
EEGQPLTDEELRDELMTLLVAGHETTATGLAWALHWILTTPGIEARLRRELADGHFTPER
IARLELLDAVVRETLRLQPVLPFVGRILDKPVRVGGWDLPAGVGAVCSIYLAHKRPEVYT
EPDRFNPDRFLGTKFSPYEFFPFGGGIRRCIGMAFALHEMKMVLATVLARTILRPAHDRA
IRAVRRNITLTPSDGCSVVLKRRAPRSTAAHAA

>CMC5_036230|KEGG|CYP147L1|[Chondromyces crocatus]|ccro|DP

MPRNDLFQQATRPAHRADPYPLYAELRQKPVWLQDDGSYVVSTYREVSALLYDPRVSADP
MKRSDLPPIYTDPRMPRDQPSFIQLDEPKHGAMRRLTMKHFGPPRSAGRVSGMGARIGQI
VDRLLDAHRDARQLDVVQDLSYPLPVMVICDLLGVPLDDEPLFREWSEVLTSSLDPDPAL
QNDPAFLERIGSAAVGIRTYMTNLLQGHRRTPNDSMLSAFVNDPEAREHITDGEFLSIAI
LMLIAGHETTVNLISNATLTLLRHPDQLARLRDDPTLMPGAIEEFLRYEPPVQYRERTTL
TDISIAGTTIPKGASVNLVLAAANRDPLRFSEPDQFLLEREDNQHLSFGTGVHYCFGAPL
ARVEAQIALNELLRRLDNPELTADPPPYRFNPALRGPSHLVVAFDGMRA

>CMC5_041820|KEGG|CYP107HW1|[Chondromyces crocatus]|ccro|DP
MSSPTIAEVMATPAFQLDPYPLYARLRDEQPLYRSPHGVAYLSRHADIEKALRDPRLSND
RDRIIRAMQAQKNDTQLMTRLMRKLGRVMTNSDPPYHARLRKLVGKAFNAGRIRDFRPQV
QALTDDLLDAAQARGKGMDLIAALAYPLTSTVICALIGLPRDDQARFLAWFRQLEDPIGA
GLSIGETEALVDTLYGALRALIHLREREPGDDFLSALVHAEEGGERLDEDELLSACFVLI
GSGYETTMNLIANSVFTLLRHPDQLCALQEKPELLPQAIEEVLRYESPSLQVIRVVAEPV
PIADGTLEEGEMVSLLLGAANRDPLRFPDAERFDITRTDSRHVSLGSGIHFCLGAPLARL
EAAVAVGTVLRRFPSLRFEDSTPAWRPNPALRGLAHLNVAWS

>CMC5_048430|KEGG|CYP107HU1|[Chondromyces crocatus]|ccro|DP

METRASQEQPRALRAVTEDVSRRDAQGGESIPVLNVGDPEFLGGAYEAYEALREKGPVVR

VAIPVSTGSDEEENPEGRTAFQEIYGKEVFFLSRYAEVFEALRDERFSSCPHALKSAEEL
AEEPVVAEELKPLRDSLLTTDAPDHTRLRKLVQPSFTAQAMEALRPRIVQLAQGLLDQAE
AAAAARGEKAGERRMDLVEAFAYPLPVTVISELLGIPEADRDRVRHWTEDLLINRGSRGL
DKAALGKLQEFSTYLRALFVERRDRPAEDLISRMVHAKEEGDALSEDELLSMVFLIYVAG
HVTTVNLIGNGVFALLTHPEQLERFKASPSLSRGVVEETLRFWGPVDTVSGRIAKEDVEI
AGTVIPKGSRVMCGLAAASHDPARFDHPEAFDITRPDAGRHVAFGRGIHLCLGAPLARLE
GQIAFEVLFDRFPSLRLSRPAEEMSWGHKVLRGFDEIPVLF

>CMC5_053220|KEGG|**CYP1007F1|**[Chondromyces crocatus**]|**ccro|DP

MANPSRDTALHALPGLRPLPLLGGRPHLVRFLRDPLTTVRALHHEYGDVAVLCAEKPAMV
CLFGGQNNRQVLTDAKLFVNYSKSLVETAPDSPLTRLTTSLPSLNGEMHKRRRRLMMPAF
SKGRVEAYRDQMVEVTERYLARRPSGQIVDIASEMTELTMCIALQCLFGLDVSNEAEDLG
RLGLQFLEGLTSLGVMALPFEIPGLPYRRFMQRCAQLEARISALIQRKRSSGEETQDLLS
ILLRTHDEDGGALTDAELLSESTGLFIAGHDTTANTLSWTLFLLSQHPEIYAALVDQLTS
VLGGEAPTVEQLSQLPLLDAVIKESMRLLPAAPLLFLRNSSEKAKLDGCTLPPDTTVILS
PIISHHAPEIFPEPRRFRPDRWSTLRPSPYDYLPFGAGPRTCIGASFAALELRIALAILL
QRRRFTLSPDAVVSHHMRSILMGPRKGLRMVWTRQDGHFARPGGVRGTIHDLVDLS

>CMC5_054910|KEGG|**CYP1224J1|**[Chondromyces crocatus**]|**ccro|DP

MKSSLPPGPRLPGFIQTSLYMSRPLHFLKRWSQQHGDTFTVHMTGSGDFVFITSPEDIRR
VFTASIDVIYAGESNSLVRPFVGDSSVVVLDGEAHIRSRRQLLPPFQNERMQTYATIMRD
VADASLDRWPVGRPFPLLSKMTEIAMELMLRNIFGLEDPREIATFLERFTSVLDEATSPM
RVMASFAGLDLYKLLPFLHVSKLKRQLDDSIYELIARRRAAPRDPTRQDVLTLILESKHE
DGQAMTDRELRDALVTLIAAGYETSAIGMTFAVERLLAEPWALAKVHEELDRVLGQETIV
AEHLPALEYLDAAIKESLRLRPLVPLISRRTKAPFELSRHTLPAETMLIPALVLTHLRED
LYPDPERFDPARFLGTKPDPYAWLPFGGGARRCLGMAFAMYEMRMVLATVLRRASLELAS
NRPVRMVNRHIMLAPSDGAPVVLKARRPKPQTKNSAAA

>CMC5_054920|KEGG|CYP1286B1|[Chondromyces crocatus]|ccro|DP

MLPSGPTLPGRVQLFWWMFDPTSFMERCTREHGDTFTTRFTSYPPSVYVTDPEVIKQVFV
ASAEDLSAGQANTGLMGFLFGERSVLSLDGAQHLRSRRLVLPPFHNERLGRYGRLMREIT
DQHIDRWPIGKSFSVSKAMHELTFDVILRLLFGVHERAFYEPFSKLVAELVRQTTSPLFH

GLFAMLPPEWVMSMLTRGPTKVALGPLGERDLSLFMPGGAILQAKREIGALIHAEISLRR
ASSQRGDDVLSMLLDARDENGEGMSDDELHDQLITLFIAGHDTTANALSWAVHHLLQHRE
VRGKLDDELSGVKGEGAGWLQEVAELAYLDAFIKETMRLTPVAPVILRILQRPLRFGQYD
LPAGSYVCPCVYLTHRRADLWPEPERFLPERFIGRRVSPYEFFPFGGGARRCLGAGFSMY
EMKVILARLVARAALRPVGGGEVRPERRGIFLAPSSGVPVVLEGRREA

>CMC5_063310|KEGG|CYP107AM2|[Chondromyces crocatus]|ccro|DP

MNTDRDEQVREYPFRAPGPLEPAEEWAPLRKGCPVAHVRLPSGDQAVLLTRYDDVRQVLS
DPRFTHNLSAEGAARTTANEAGGVFERQEASMLTDADRHQRWRRMIMKSFTVKHISAMQQ
RIEAMADQLIDDMVKQGPPLDLASALGFPLPVWVICELLGIPGVDRDKLAYWSNTMLSVT
QYGQQEIDAAQAEFKAYLTAHIVAKRANPGDDLLSELLAAGDEGQGLSDMELLLTAQGLL
VAGHETTSNMIGKMVAMLLSDRRRWEQLLADPSLVRTAVEEVLRFDANAGFGLPRFITED
IEVAGQTLPRGTTVICAMASANRDERTFAEAEEMNLARSPNAHLTFGAGPHSCLGQTLAR
TELQTVLRVLLRRLPTLALAVPTEALSRRAGLLVGGLEQVLVRW

>CMC5_067690|KEGG|CYP1496A1|[Chondromyces crocatus]|ccro|DP

MDRRSSFEVTSTSFFSDPYPTLRRLHDERPVYYHEPINAWFLVKYEHCEAVVRDPRFSSV
RARELLHTLLPALRGTEGLDALVAQFSRLLWFVDPPRHTQLRSMVNRGFSASAIERMREK
IRTVVQRALSRIGDGGEVDVVGDFADPIAIDVLCALFGLPEADSDVLRAWTSDLGKVAGA
SWADGTGGTDAKDASSAFAAYLKDLIADRIAHPGEDMVSRFLEGAGSAPEDIEGVIDQCY
QVIGASYLPTCNQIGNAVLTLLQHPGELQRLRTTPELFRSAVEEILRFEPSTLTTNRIAS
EDIELGGERILRGQLVFPVLAGANRDPAVFPEPERFDIGRRGARHLTFSVGPHYCAGGAL
VRMQLEEALQALLTFDTWELAGACDFRGSGLQDRGLATLPVRFSRRPRSPRAAHGALESD
LALLEV

>CMC5_068540|KEGG|CYP125E2|[Chondromyces crocatus]|ccro|DP

MELADIRIADPDLYLTGVPHDRFERLRREAPVFWHEEEGGTGFWAITRHADAIQVISDAK
RFSSARGGIWLEDYPPDDLRSNRDGLITLDPPRQTRYRGLVSKGFVPRLMQAIEPHAREI
VTAMIDRVEERGSGDFVSELAGDLPLRIILQLLGIPLEDRAQVAAWAYQFLLAADKPKEE
VEALVRDLVGYAARLAEERRASPRDDMLSVLMEGELDGEKLSSFEFGLFFSLLLSAGTVT
THQLIAQGMLTLLERPETQRRLEEDPSLIPSAVEEMLRFVPSVGYMRRTATCDTEIQGQR
IAEGDKVVAWLVSANRDEEVFPDAQTFDVTRSPNEHLSFGRGPHFCLGATLARLEARVAF
EEILRRLPGMQLAGPVQRMRSNWLMGLTHLPVRWSPSLRARP

>CMC5_069130|KEGG|CYP167B1|[Chondromyces crocatus]|ccro|DP

MLDHTINHGQQPLKFCPFSTEYSANPYPTLERLREGSPLMFWEEGRCWVVSRHADVFGLL
RDPRFTTDRRTWEFAKPSEVEARCPEYAEMSKHSLFSLDQAAHARVRKLVSPSFTPRAIE
RLRPAIQHIVDEVLDAAAGKEILDVTRDFAEQIPIRAIGAMLNVPPERQAAFHRYADAMI
RQLFPMLIRPEEVDGNFASIREGIAMIDDILEERRQEPREGDLMSSLLQAEEQGDRLSRR
ELQALVSSLIVGGSETTVHLIGFMVLNLLRRPALMEQAKDEPELVKSILEEVLRFDNFTR
LGLTRYARQDMDFNGSHIRKGQMMILLLNSALRDPSAFPLPDEFDPHREAVTSIAFGNGA
HFCLGASLARLEGQVAVSTLLRRFPDMHLVGTPIFGPHPVIRKLDSLKVALHASAR

>CMC5_069160|KEGG|CYP167B2|[Chondromyces crocatus]|ccro|DP

MLEQNRENAPQKVDFDPFTNDYAKDPYPTLERLRANSPLLYWEEGRCWVASRYATVVVLL
RDPRFTTDRSVWEHAGHGQAAALCPEFVEMNRNGLFSLDQVAHARVRKLVSPSFTPRAIE
RLRPAVQGIVDEVLDAAEGKDVLDVTRDFADQIPVRVIGALLNIPADRQAQFHHFADAVV
RQLMPMLIKPSEIEENFAAIREGIAMIEGVIEERRQHPIEGDILTSLIQAEEQGDRLSKG
ELVSLVSGLVVGGSETTVHLIGFMVLNLLQRPELLAEAKANPELIKGVLEEVLRFDNFGR
LGITRYAKEDIELDGARIKKGQMVILMLNSAMRDAAAIPMPDTFDPHRDTVTSLAFGNGA
HFCLGASLARLEGQIAVGTLLSRFPDLRQEKGAVFGPHPLIRKLDSLQVRLRPAVS

>DB32_001278|KEGG|CYP110T1|[Sandaracinus amylolyticus]|samy|DP

MKTTLPPGPRWTLPATLRLIRDPYATLRELRAEYGDLVTFPSPNGRVVLAMTPELVKPIF
TASPDHFGAWAVGTTSVVLGPRSLIVSEGETHKRDRKLLTPAFHGARMRAYGDAMRTLAR
RRFETAMQPGATVTMQDVTTDITMDVILRTVFGVGEGAAFDEGRALMGEIVRGMSPLLFF
SKASHTPLFPPWRRFVRIRERFRAWLDARIAEARARGDEGDDVLAMMLAARYDDGSAMSD
DDVAAQLVTLLFAGHETTAIALAWAMHWLGRHPDALALVRAEIASVGIDADPEVIAKLPY
LSAVCDETLRLHPIVTENLRMLRKPLALGGYTIPPGIGVAAAIGAIHADPAIYPEPDAFR
PERFLDRKYGAFEHLPFGGGHRRCIGAAFAEYEMRLALAVLVDGWDVELVHRHERPVRRS
VTLGPAHGVPVKVIGRRAGLRAAA

>DB32_004988|KEGG|CYP253M1|[Sandaracinus amylolyticus]|samy|DP

MDARPYESMPTLPGGLPLLGHWPEIIHDVFGFLERAAERADVVRVRFVHERAVITHDPAM
IQHVLQQSPRLYAKSRNYAGMKKVVGEGLLTSEGDFWKRQRKLAQPAFHHAKLRGITRTM
ASATEDMLARWRTWEDGRPFDLHEEMMRVTLRIAGLSLFGADLDGESREIGAALGVILPW
VNGIIQEPFRPPLWIPTRENRALREALATLDRLVYRIVEERRRSDPEHQRDDLLSMLMAA
TDDEGGGGMSDRQLRDEILTAVLAGHETTANALAWTGMLLARHPEIGARVEREASAVLGD
RTPSVDDLSKLELCDRVVSESMRLYPPAWEFEREALVDDVAAGWRIPKGTVVMIAPWTLH

RSPRFWDEPARFDPDRFLPERSAGRPRYAYLPFGDGPRVCIGKAFAMMESKLLLAMMARE
VRFELEPGAHVRPEPSVTLRARGGVPMRFRRTAPAPFVDQTTSA

>DB32_006288|KEGG|CYP51B3|[Sandaracinus amylolyticus]|samy|DP

MDETVRRATRRRDEAPSGLLPAPRLSGGLPVVGHSVEFVRATIELLFRANRELGEVAAFS
VFNREMVAMFGPEAHEAVFRAPDAVLSPTEAYKIMTPVFGKDVVYDATPEKMAEQLKMLL
PALKDRRMRTYGEAVVHETAASTREWGTSGVVDFVEFCRVLTNFTSSRCLLGKEFREGMS
EEFARVYHDLEGGVTPLAYIDPHLPVPAFRKRDKARVRMVEMITGIVRDRRAHQRTGEDF
LQTLMESNYADGRPLSEHEITGLLLAGIFAGHHTSSVTTAWTLLELLQNPTQLQRCVEEV
DRVFGGGRPVSHAALRELTTIENAVKEALRLHPPLFMLVRVVKQDFTYKQFFIPKGTWIV
VSPTVSHRIPTVFRDPDAFDPDRYLPGREEDKKDFAYIAFGGGRHKCLGNAFAILQIKAI
LALLLGQFEFGLCGDAIAPSFQGLVIGPKEPCRVRYRRRAQPSVTMEMGTELAAAAKDAI
AHELNGAKEVPQAVQQAAAAAGCPAHAHVAATPSTSAGSSLRVKVDRDLCQGHAVCATEA
PEVFAVSPKDHKVMLKVARPPAELHDKVRKAAQYCPNHVIRIEETD

>DB32_006357|KEGG|CYP117D1|[Sandaracinus amylolyticus]|samy|DP

MPVFRGAFPALGHLPALASDPLGLDRIAEREVGPLYWTDTGFGRWQLRCLSPESFDLFKN
VQTSSDYVGETLPDLFGRSVITLDGKPHRHARSVMGPAFTPGGLSAARVGSLFADVIERH
VHEWIDRGVITTLASTRELVLALILRMLGVPERDLDVWRRDYEDHMLLALGVPLDLPGTP
RRRGLTAKARLDERIGALVREARAGSDTTVLAQLSRAKDEHGEPLADAELLDNLRLLILA
GHETTATVIAWLIAYLAESPSLLERLQSEATDVPRAPGDLRAFRFAEALFREVLRLQPPV
ARDARRAVVDLEIAGRRVPAGSIVAISIELLSRSPRTYEDPDSFVPERWMGKTAPPTALE
LVQFGAGPHFCLGYHVAWLEVVQTSVILARALRARGARLALDGRFPRVRHLPLRHPDASS
RVRFVRG

>DB32_008533|KEGG|CYP152Z1|[Sandaracinus amylolyticus]|samy|DP

MGSIPRDPAIDGSLALLREGYLFLPRRFERLDTDVLELRLMGLRFIALRGRDAAALFYDP
TRFVRRGAVPMAVQRTLLGERGVQTLDGDMHAKRKKMFMSLMSRERIDALMGEMWKQWYA
ALDRWQQREEIVLLEEAQDVMCRAACAWAGVPLEEREVRLRAEDFGAMVHSFATIGPRHF
AGRIARARTERWMGGVIQRIRRGELRPSPTQASYVVASHVDANDRPLDEHEASVELMNVL
RPIVAIGTYVAFIGHALHAHPDHRAMLRAPVDETTRAERVEAFVQEVRRFYPFTPFLGAR
VRESFEWRGYLFPKGRRVLLDVFGTDHDRRLFDDPDAFRPDRFLGWAGGPYDFIPQGGGR

YEGGHRCAGEWLTIEAMKVALTFLTRAVELRFAPQDAHIDLSRIPTLPRSGMILTDVRAV
ASDAPRPPVTRPDGAAARI

>DB32_008796|KEGG|CYP253J1|[Sandaracinus amylolyticus]|samy|DP

MPLLGHLPLVRPDRLDYLMRVALETGDVVRFEFPHVTAHLVAHPDHVQQVLVDQHRLFTK
QTRGYAKLRAFLGNGLVTSDGEFWLRQRRIAQPAFHKKRIAGFADAMVRAAEDTVQRWAA
PAREGTPIDVAAEMMRLTLRIAGETLLSTDPSDRANAVSTALTTVLHEANTRINTLWSPP
AHWPTPRNRAYAAATRELDRIVLEIIEQRRRGREHRDDLLQMLLEARDPETGAAMDDKQL
RDEVMTMFLAGHETTANALAWTFVLLSRYPAVARALHEEACDVLGDRPATEADLPKLDLA
RRVLNESMRLYPPVWIIGRSPAEPVEIGGYDIPAKTIVFTSQWVTHRHPRFWDDPEGFDP
DRWLPERAKTMHRHQFFPFAAGPRMCIGAGFAMMEGQLVLATLARRYRVDLLPGHPIVPE
PLITLRPKHGVRATVHRID

>AKJ09_00712|KEGG|CYP1373B2|[Labilithrix luteola]|llu|DP

MRAIAHKMDIMTNATSEAMPSSKSARKSVHAFPRLPGGHLLFGHLPDLKRDRAGLQFRVG
REFPDAVSLRFGVVRALMIGAPALANEVLSTKNASFIKAPGLTVFLRPVLGNGLLTSDRS
FHARQRKLIAPAFAHKRVASYADTMAERARSFASGVRSDEVLDVSDAMMRLTLEIVGKTL
FDAEVGSDADEIGEAVTTAMECAIGQLESFLPLPPIVPSPSNVRIRKAVARLDAIIYRLI
RARRDQGGDRGDLLSMLLAAQDEDGTAMNDRQVRDEAMTLFLAGHETTANALAWTLYLLA
KNPEARAKVEAEVDTLGRAPSYEDLVRLPYTLAVLKEAMRLYPPAYILARRAVEDVSLGG
HLVRRNTIVLVNVLGLHRRPDLFPEPERFVPERFLGDEEKKLPRCAYMPFGAGPRVCIGN
HFALMEGHVLLATMLGRLRFELAGGADVELEPLVTLRPRGLRMRVHVREAQN

>AKJ09_02861|KEGG|CYP210A2|[Labilithrix luteola]|llu|DP

MLDNAGSRFGDIFTLRLPVGANGIVLVANPEAVKEIFALGPDEGHAGKANIVLKPFLGRH
SLLVLDGAEHGRQRKMMMPAFHGERMQAYGQAMLDLAHDSVDGWPIGRSFVVHRPMQRIT
TQVILRTVFGVAAGPQFEELADLLAKTLDAAAWPGLLFPFMQKDLGPLSPWGRWRRLRDR
ASSILHEEIRQARSNHESRNDVLAMMVAARDESGRALSDDEIHDELITLLVAGHETTATA
LTWALRWIVPNRELTRRLQDEIAGADDDPAKIARLELLDATVKETLRLQPVIPIVGRVLQ
EPMRVLGWDLPTGTWLAPAIALIHQRASLYPNPRRFDPDRFVGARPAPWEWLPFGGGLRR
CIGAAFAIYEMKMVLAAVLSRVSMRLVKNEVRTVRRSITLAPDEGLPIVALERRPRKARL
RAA

>AKJ09_06094|KEGG|CYP1007B1|[Labilithrix luteola]|llu|DP

MGTVFHLIRYGRDPIGVTDRLFREYGPIVSLVRGPVRIFSPAGCAMVFATGAGVNRQILT

EHDRYHMCALPGALYPSDEVLAEAAAADKRWRATPERLQPIRRTLTGLFHVNGAEHRRHR
RLLMPAFHKTRIDAYRDEMVALTTKVLDEWRDDQVRDVLEDMTELTLRIATKTLFGEDAG
EQGVRLARMFQEWLVTKFSPAMLLRFDAGPTVFRRWLDLTRDIDQCTLDIVRAKVARSKS
QRDVAPDMLSSLIAARDEDGSALDEDELVGHAGVIFAAGHETSTNALAWTLLLLSQHPDI
ATDLVDELRGVLRGEAPTVEQLGSLPLLDAVVKESLRILPPVPIHPRLVAKDSELEGHSL
PAGTELFLSIYHMHHDPDVFPRPQRFDPSRWSKIKPTVYEYNPFSAGPRMCIGASFATME
IKIVLAMLLQRFRIELLPGARVDRRVTITMAPKNGLPMRIRRADDAWGGGPRDLRGNLRE
LVDFS

>AKJ09_06238|KEGG|CYP1007G1|[Labilithrix luteola]|llu|DP

MTMLRLHGFASVDIRGPRALPLVGTARRFISFLDDPVGVVLALRRMGDVVAVVDRNPAIV
CVFGPERNREVLSDPDTFHNPEDFFTGPDGSAREKMRAMVVTSNGADHRRRRRLMMPAFH
RSALDGYAEEIVTLTRSLVGAWPRGEVVSLDGLCRELSLCIAVQCFYGVDVKRGASVLGQ
TMAEFVRLVTSPANILFPFDLPGTPYRRGVQIGEDLASRMLALVESKRQSGGQRRDAMGL
LLDARDEHGNGLTADEIVALAVELFIAGSETSSMALTWCLLLLDQHPRVLEALAAELSDV
LGERDPSPADLPKLRLLDRVVKETLRVLPAAPILFLRSATRDTMLGGKAIPKGANLVVSP
LATHHDASLYPDPDRFLPDRWKEIEPSTYAYFPFGAGPRMCAGALFASQSVRLMLAMILQ
RFHPRTVDAGPIDRRVRANILNAQHGVHARLCPVRANAAPAAPVRGNIHELVKLESRGRS

>AKJ09_10261|KEGG|CYP1366A1|[Labilithrix luteola]|llu|DP

MASNILVTDVSGIPSAGPLRRLPTKFALPLVGDSFAFLKDPAGFLATRAHELGPAFQINV
FGHRTACFWGPEAFHFFLDERYFTRASASPAHVQKILDAKAVPFLDGARFKRRKALLMQA
FTPDALDAYLGVLERVLTRYARRWANLGTFAWVPEMTAFGMTVATSLFLGTNPDEDDPLL
ARAFMTAFEGFLSVPINLPFTKYGRALQARDALRERIEHAIDEHEASTSTDALARLIDAR
LPNGDKLSREEIRIETFHFVGAYLAVIGALSSLAMFLGRRPDVKERLRQEIVDTLGTGPV
TLRGLAGLEYLGRVTRESRRAEPVLPMTFFANVKEPCAYGGMRIPAGIKAIGCIGATLQD
ERTFADPRSFDPDRWLPERATDRQHAAWVPHGGGGPFTHHRCAGERLADLMLSLFSVVML
RDYDWSFPRQDFSPTAAALFATPRAGLQVQLQRRTSDRSPRV

>A7982_00879|KEGG|CYP51B2|[Minicystis rosea]|mrm|DP

MNLEPIVETLLRRKKAPPAGLREPPVLSGALPVVGHTVEFVRSTIGLLSRAQRELGEVAA
IEIVGKRMVAVFGPDAHEAVFRAPDSQLNPSEAYKIMTPIFGKDVVYDAPPERMNEQLKM
LLPALKDRRMRTYGEIVVSEVEQSIAGWGDSGEIDLVEYCRVLTNYTSSHCLLGREFRKG
MTEEFAAVYHSLERGVTPIAYLNAHLPLPSFRERDRARARLEQMVTQIIQERRRAGREGE
DFLQTLMDAKYKSGAPLTEHEITGMLVAAMFAGHHTSSVTTAWAIIELCNHPQYLRRVVE
ELDNAFAGGEEVGFASLRNIPHTERTVLEVLRLHPPLFMLVRVAMEDFTYKGFFIPKGTW

VLTSPTVAHQIPAVFRNPESFDPDRFAPGRDEDKRDFAYIPFGGGRHKCMGNAFALLQIK
AILAILLRRYELELVNPHVPSDFHGLVVGPKEPCRIRYRRRKAVSAGARPVAAAAVAAAP
EKRYRVSVDLDLCQGHAACVGEAPEVFRVDGLGKVRVIDTTPDASLSAKVEKAAQFCPTK
TIKIEAI

>A7982_00887|KEGG|CYP263A2|[Minicystis rosea]|mrm|DP

MSSIRDQKPPGPGPLGSLRYLANFRTGNMKHYEDLFARHGNVVRLRSPGVEDFILAFHPN
DIAHVLRDNARNYPKGKRYHELVPVLGWGLVNSEGDLWRRQRRHVQPQFNHARTLEFVPI
IVAHMEALLQRWDAEAGEFERDINDDMMDVTFGIAGEAFFGAALQAHTDTVRQAFKFALS
IALTRMYSVVNPPLSWPLPSHVRFRRAMDRVHAVIDSIIDGYQQDAGDTDNVLVRLMKSV
DPETGAKMDRVQLRDEIKTILMVGHETSSVTATWALYLLAQHPEVYAKLTDEIDRVLGGR
SPTAEDIESMPYLTMVFNECLRLLPSVPFILRSPSEDDTIGGYDVAAGSTIAIVPWVTHR
HPAFWREPHVFMPERFVDYRRNVEERFAFIPFGAGQRICLGEFMGQLEGKIMVAMALQRY
RLRLVPGFDPRCRGFISLQPLNGMRMLCRRRETVSASRAKPIAAAQSPAVAPERRGCPHA
AGG

>A7982_01273|KEGG|CYP183AH3|[Minicystis rosea]|mrm|DP

MTTQVPTTSCKLGIAPGRFPSIRHPLQMLTSPLEFLTSLRDAGDLVKVRLGTEWAYFVRH
PDLLRQVLLGGPQTFDKGGPFFDKAQTVAGNGILTCPGKDHARQRRMMQPLFSRERLVRY
AAVMHEEAERVAASWRPGQQLSVNKAMSALTLAVTTRTLFSTEAPAGIVAEAQDALIVIF
AGLYRRMIVPLDLVHDLPTPDNRRFDQAVTRLPEIIDQIIERYRRAGIDHGDLLSTLLAA
RDPETGEGLSDRELREQVMALFTAGTETTASALTSTFYLLDRHPDVERRLHAEADAVLHG
RAARHEDLEALDYTTRVFTETLRLYPPGWILTRRTNAAVDLGGHRVPAGATILFSPYALH
RDPEFFTDPERFDPDRWLPDRAKAVPRFAMLPFSAGQRKCMGDTFAMNMATIILATLAGR
WRLRSGADTKVRSIAKMTLSVEATPMRVEARAVG

>A7982_01853|KEGG|CYP1373D1|[Minicystis rosea]|mrm|DP

MDFEAMPIAPGAQGLLGHLPGFRKERLKTLGLLSRQSTPMMRLDIPVAGIRALALNHPDV
VQEALVEKVKHFHKSDMLRFGLYDLAGEGLFTSNGELWRRQRKLMAPLFTPRALEAYATD
MVACTRRTMEGWRDGEELPLSRETTRLTMGIAGKTLFDADTFSEADEIGHALTIALDWTG
WIVGRPFAFAHVVLKRMAERLAAHATGNVREALTRAEQRFSRPVVHVGTRGRELSRAIAF
LDEHVQRMIDDRRRTAGTGRADLLSRLLEAHDEDDGAKMSDRQVRDEVLTLFVAGHETTA
TGLAWTIYLLCKHPEVLAATVREVDAVGDDPSVSDLPRLDLCLRVFKEALRLYPPVYVFG
RDSKDPITLGGYDLPAPTNVLTSPWVLHHAARFWPDPERFDPDRFRPENEAARHRYAYLP
FGAGPRICLGNHFAYMEAQLALATMLRRYTFELLAEDEPEPGATLRPKHGVRVRVRRRTH
ARAFVGEAVME

>A7982_01903|KEGG|CYP1493A1|[Minicystis rosea]|mrm|DP

MLEGFAAKWRDIGLLPAPSPKQKALVFVNAPELIAEVLIGKSACFEKSEFQRSVIGAPDR
LGSGLLTSSNEDHRSQQRLIGEAFKPTRLQDYTQILAECARDLQASWADGAEIDVPAEAM
RLATRTLGLSLFSTDMSPDGLDDRVDRATATVADSITSRYVGSARQQMRAESLENASAWL
RTYLAGLIRSRVDAPRRGRDDLLDVLLSARASTAGVDPWDDEQLIYDIGTILLAGTENPK
NSTAWALHLLAAHPEVRARLVAEVDGVLEGRLPTHADLGRLTYTQQVYKEVLRLWPPGYA
FSRTAVQPVTVGGIDIPVGAEVLVSPYVLHRRADLYPNPTVFDPDRFAPSTERRLHRMAY
LPFGAGPRACIGGGFAMIQGPITLAVLAQSVLLDAVPGQHVEPEPMVTMRPSAFTMRVRR
RPEAPEVR

>A7982_01917|KEGG|CYP1500A1|[Minicystis rosea]|mrm|DP

MSTQTVDDFRGASTLPEPPLDRGPMIVGHLPEFMWARGPLLARLYRVNGPCFRMRMGLET
PVVMAGPEMAEFTTKDAGRILHAGPSNMGFVREFGGTNMMLGADMDPHFHRRRNLMGQFT
PGAVMKMVPRFAAVTVEMASAWSTGAPLDLLPCLKRLVTRQTGLVLAGRDAEAIYDDLCR
FFDLAVAVCFAQVPRPVMKLLGYAEARERVLGFASSILDDRAAMPADARPGDLIDAILAA
REPDGSPLDRGEQVGLVLAPFIGGLDTVSHTLSFLLYEVLTRPELQARCQREADAVFDGD
TLDPARVRNLETIAATVSETLRVHPIAPVGQRVAAESFDFAGYRIQKGTKLLLGWSVGNQ
VASRYAHPERFDVDRFLAPRNESTSGIGAFGFGPHKCIGAKLGEVQLAVTLAALLRKTTM
RLDPPGARLRTTSYTTLKPVGLRVRFERRAAPERAAS

>A7982_02131|KEGG|CYP1069G1|[Minicystis rosea]|mrm|DP

MIARARPLPRRSRLTLPWTLHEEPPMSTATTIAFNHLAPPDLHDPYPLYERLRLEGPAVF
NPIFDLWFVTRHEDVVAVVKDPARFSSADILKPLHPLSPEAVAALGEHSDVYPLLSGDPP
MHTRVRNLVGKAFTPQRVAALEPRVRAIATELVAGVAPEGRADLIRRFAYPLPMRLTADM
FGMRAADMDDVKRWCDEETLFLMAPLPPERQVECARSVAAYRRYLRDLVEDHRAAPKGDL
VDALLDAQHEGEVPLTTEEIVGALCVLIFAGHQTTTNMIGTTLLHLLRRPGAWQTLRDDP
GLIPGVVEEALRFDAPVQGMTRTVTEETDLGGVTLPKGAKVFVVFASANRDPACAAEPDT
FDVRRAPPAQHLAFGRGPHFCVGASLARLEGRVALEVLTERLPNARLVSDEALEYLPNLV
HRGPSALPVAWDVP

>A7982_02356|KEGG|CYP1499A1|[Minicystis rosea]|mrm|DP

MPAHDAIHSDLPSERVAAHRTTSPRALPRLGTLARFANRIRTFIDPLTASQRVLRAHGDL
VAVDDRVICTDAPAIAKEVLLDHRTFTKDFSQVYDVLSTSFWGSGFAVSNQDAWLRKRRV
VQAALSADHLRAYADVVTAGASRLLDTWRSGETRDVQADLMRLTLENTARVCAGSDLDAA

LPQIEAAVVAAMRWFENVELHAIGAETREEVGFKEAVARMNEQLAGVIQRRRSAPEPRKD
VLSAMMSTPCEDGSLMTDDELRDELVTMFRAGHRNTATTLAWAIGLLAAHPRAQEELAGE
LARVLGGRAPALTDVPRLSYATRVVQETMRCYPLYPFILREARQGGTLAGRRIPASSKVI
ISIWSMHRDGRTFDRPDAFEPDRWAAPLDDAREIYAFLPFGGGPRRCPFKSYAMVEAVLL
LASLAQRFRFRLAPGHRLRPMNTANGLMPRDGMKIVLEAR

>A7982_03064|KEGG|CYP1069D1|[Minicystis rosea]|mrm|DP

MLEDPRPFYARARREEPVFWSESLGLWVVTRHDDVCAVAKDAVRFSSMDSITPQVAPPPP
EMLAELVKGFPLLPSLVTSDPPAHTHQRALVTKALSLRRLATFEPILRGIADGLVDGFAA
KGRAEIVEAFAIALPGNFIVDLLGLPREHLAQVNHWTTDSAEVFAGHKPLDQLVVHARGF
VEYQHYLAAAIEERQKSPREDALSDIVTGAAALNPPLGMPEIINMLLQVIFAGYETTAGL
IAASALELARDPELFAAARRDPSIIPPIIEEVLRVASPIHAMYRTANEDVEIGGVRIQKG
ERLQIAYISANHDERRFEDPLRFDIHRTTPHLAFGHGIHFCIGAPLARLEGRIALEVLTQ
RMPGLRLVPGQKLSYFPSATARRLDSLLLEWP

>A7982_04313|KEGG|CYP1069F1|[Minicystis rosea]|mrm|DP

MSRSTVIPFNPFIPPSLDDPYPALEAARRQASVFFNPVLEMWMVTRYADVIAVVRDPKTF
ASHDTVRYPQDLSPRTRELLATGYEEMSILVNDDPPHHTRMRGLVGKAFLPERIHAMTPH
IRAIASTILDRIAGAGEADIVAELAYPLPAKVICAMSGVPDDDMDKVKRWSDDWLLLSLG
RLPEEKQLACAEGVLAFQRYTTEIVEARRRAPQDDITTSLVQARFEGGEMSTPEIAIFMM
GLLLAGHETTTNLIASTLLHVLRDPRVKQQIVDDPDLIPLAIEETLRFDAPIQGMIRTVR
AEATVGGVSLPPGERLFLSYAAANRDEEQFTDPNRWNPHRSASERHLGFGHGIHFCIGAP
LARLEARLVLEQLFERLPNLRLVPDQTLEYVPNLLHRGPRSLRLAWG

>A7982_04652|KEGG|CYP1373C1|[Minicystis rosea]|mrm|DP

MRLEDIPLLSGANRLGHMEEIRDDRFNFFHRLNRECGDIGRVHALGTPLIFANSPALLHE
ILVEKAKSFVKSPGLRGPLRPLAGEGLFTSDGDLWKRQRKLMAPLFTHGMVAGYAPTMAH
CAREAVAPLRTGEVVDVARLTTHIAMRIAGKTLFDSDTLDEADELGEALTVALRWANDQS
SSLPYAAQLRLSGVFADVAEELPEPLRARAKTLAESLVEPIHWPGEQTRRIEHSTAVIER
RVERMIAERRAAGLDRRDLLTALLTAYDEDGGMSDKQVRDEIITLFVAGHETTATALAWS
LYLLARHPEAYARARDEARQLGGRAATIEDLPKLGYCLKVFKESMRLYPPVYFFGRQASA
DVRIGAYDLPRGAIILISLYAMQRRPEVWPDPERFDPERFTPAAEEARPKLAWAPFSGGP
RTCIGNHFALMEGPIVLATILDRLDLALTSNAEVLPDDSATLRPKGGVAMRVTAVHHRD

>A7982_04960|KEGG|CYP120G2|[Minicystis rosea]|mrm|DP

MTAPVSSVIYPQPSAGDIARLPLPPGSFGLPLLGETLEYLRSPPEFIGRRYRRHGNVFRT
HLLGAPMVFLIGSDALRFSFAGENKYLQQRWPYSMRRLFGAEGTNLLVGEVHKERRRVLG
PHFAFNAMRGFVPTMEAIAKKHLARWSEQSGVLTIVPAVRAMILEILSTLILSDEPVDQA
RLGRLCHDWFAGLVVPIPLDLPGTAFGRALAAKREIGALLGAVVDRRMKLATQPDDVLAG
MLSVRDDANRPLSRQAIVDEIMNLMIAGQDTTISATGNLLLLLAQHPDVLRRCREEQDGI
AIEGGLTLDHLKSMPYLNQTVQEAMRFITPVANATRATTQDVACAGYRIPKDWTVALCLA
GTHHASTPWTDPERFDPDRFGPERAEHKREACPYVPFGGGPRVCLGQHLAMVEMSVIVAL
MLRGYRWELLPDQDLRMNHIPVPSPKSGIKVRFSRR

>A7982_05203|KEGG|CYP197H1|[Minicystis rosea]|mrm|DP

MYGVRNIIGFARDQLVLLRDLADRYGDVVEMKVLGRPFFLISHPDDIEAVLVKHARIMLR
DEYAVVLERVLGKGLLTSDGDLWKRQRKLMSQAFVPKRIRSYGEAMVRVTDAALRPWHDG
QAINLHQEISRVTMEVVADVLFGSGIDHEDVRTVREAMEIVNEFLANSPEAVLKLPAWVP
TPRNVATVRAVKQLDELIYRIIQKRRAGEPRDDLLGTLLAAQDDDGTKMSDAQLRDEAIT
LFLAGHETTALALAHTFYLLSKHPDIERRLHAEIASVLGGRLPTADDVRALPYTERVLKE
AMRLYPPAWTTGREVAEEVEIGGYVLPKGAQILTSQWVVHRDPRWFPNPEGFDPDRFAPE
RAKDLPRFAYFPFGGGPRICIGNHFAMMEATLLLAVIVQRFRIELLPGQQLSLKPSVTLR
QAGPGLRVRVRERKAISPETSVREGIAIAAP

>A7982_06089|KEGG|CYP1448B1|[Minicystis rosea]|mrm|DP

MDTTPDTTAPISAVANADLRAAAFRFFTDPAARADPIPLYRRLVREAPILELGPMWLVSG
FDELVGLSARHELRSTPSVRGRALPMTPIPSLAGWLALTFPMRDGADHRRLKGLAATAFS
PARVAELRGRIEESIDALLAQATERGAMDVVADLALPLPVAISSAMLDVPAGDREQVHAW
AMLVRRQLLRYDQSADEIARIEAEMEQFAAFVHALCDERRRRPGNDLLSDLSVAADAGRL
SRDELVAFVLLLFVNGLETLTSGLTMAVWELLQHPEERLAIAGNRAYAEAMFDEVLRLHC
PVRFSARVLVGDVQLDGHRLREGDVVTLCFGAANRDPRRFVDPDRFDPRRPRSRHLGFGH
GAHYCLGAQLSLTTGACVLERIAKHGDRLTTRVTPATAAWSRSLAFTTLDTLPIEIAPPA
RTT

>A7982_06635|KEGG|CYP1373C2|[Minicystis rosea]|mrm|DP

MQLMNIDDLPVLSGADRLGHLRELAHERLSLFARFNRECGAIGRIFALRTPIVIVNAPEL
IHGVLIGEAKSFIKPTAVLGPVRPLVGDGLSTSDGDLWSTQRKLMAPLFTQAHIEAYADT
MVEHAWSEARALREGQVVDALEEMMRITMRVAGKTLFDAEVRDEADDLTAAITIALCWAD
EQIGSIPWAVRLYLASLLQALAERVPASMRAHTSTLTEAVVRPIHWPTVKRRRADAALAT
IERRIEQMIADRRRAGLDRPDLLSLLLATHDEHGDRMSDKQVRDEIVTLFIAGHETTATA
LTWSLYLLGRHPEAHARAQAEARALGDRRPRAADLPSLRYCLQVFKEAMRLYPPIYLFGR

QAANDVRIGDVDLPKGTIVLVSAYALHRRPELWPDPERFDPSRFEPQAEHRRHRLAYLPF
SAGPRTCIGNHFAMMEGPLVLATILARVDFALGTSDPIEPRPFTTLRPWRVPLEVTAVRP

>A7982_06764|KEGG|CYP1373B1|[Minicystis rosea]|mrm|DP

MSTPTSSIKTVPFFERLRFRRGNRVDSLLEMARHHGDVVRVPWFGGIVKANIISSADMAH
EVLVEQADAFVKGYGLSYFARPLLGNGLLTSERDFHRRQRRMMAPAFVHKRIADYATVIG
QRAEATQQGFRDGAVVDFSAAMMRMTLEIVGATLFGAEVGEEAEEIHEALTAAMEQATSA
IRAIIPIPPSWPTPGNRRGHRAIARLDRTVYRLIEERRREGGDRGDFLSMLLLAQDEDDG
SVMTDKQVRDEAMNIFLAGHETTANALAWGFYLLAQHPEIRERLEREVDAALEGRTPTLA
DLPRLPFALQVFKEAMRLYPPAFVVARRATRDVIIGGHRMAKNELAIINIIGMHHRPAYY
VDPERFDPDRFAPEHEKAMNKRAFLPFGAGPRVCIGNHFAMLEGHLAVAALAQRVRLDLA
PGSARVEPEPLITLRPRGGVPMIVRRRATVPHARPAAA

>A7982_07556|KEGG|CYP167D1|[Minicystis rosea]|mrm|DP

MSMSESNQRVPIFHYDPSAPAIDVDPYPTFTRLREGAPVHYWPEARAYVVSRFEDVAMVF
RDPRFSPDPVAAGQPSLDDALPDAIRGPLEQNLLRLGPKDHARVRRAVSPAFTPRAVERL
RVEVERLVDAGLAPLSGQSEIDVAPFADFIPLRVIASMLAIPLAHEVTFRRFGEALIRRA
DPRLDREAVAALAPVLAPGMDLIRRLVAERRQNLGDDLLSTLIRAEQAGDKLSEDEMLGL
VSILITAGSETTVHFICFAVKSLLCVPERVAEVRADPGILRSALEEVLRFDGFGKMGAFR
YALEDLEIGGVHLPRGTRVIGFSGAAQRDPRAYPNADHYDPRREPAEILTFGSGMHFCLG
AALARLEGETAIRALLDRYTDFRLLGEPVYATHPVMRKIASLRIGVTPRGRAS

>A7982_10186|KEGG|CYP159A21|[Minicystis rosea]|mrm|DP

MTAPDILSPEFARDPYPLYRVMRDDHPLFFHEPTKSYVLSRYEDVERAFKDPAFSSKNYE
WQLEPVHGRTILQMEGREHATHRNLLTPAFRGRDLQERFVPVIERNARELIDAFRARGEV
DLVEAFATRFPINVIVEMLGLPREDHARFHAWYTSIMAFLSNLTGDPAVVERGLATREAL
ASYMLPIIAERRRSPGADLLSTLCSAEIDGVRMTDDEIRAFVSLLLTAGGETTDKAVASL
FKNLVENPDQLEAVRRDRSLIDAAFAETLRYSPPVHMIMRTPLQDVTMTGGVIPANSTVT
CLIGAANRDERRFASPDRFDIFRKDLDTERAFSGGANHAAFALGRHFCVGAILAKTELQI
ATGLLLDAMPDVRLDGGSPPEFGIFTRAPKSLKLRFTPAAS

>A7982_10283|KEGG|CYP110W1|[Minicystis rosea]|mrm|DP

MALPPGPPLPASVQTALYLTKPIEFLEGCHRRYGDLVTIETLLFGREVAVVDPEHVKQVF
TSDPSDARAGEANAFLEPLVGARSVLLLDGQAHLRERRRMMPPFHGERMTAYARVMREIT

EQVVDTFPTDRPFALHPYMQRITLDIILRTVFGVEQGADLDALRASLIRLLDRQASTIGA
LSTIPMFRRSLFGLSPWDGFLRDRDRADALIHAQIARRRADRSPRSDVLSMLLEARDEEG
RPMTDAELRDELMTLLAAGHETTAGMLCWAFELILGDGRVAARLRNEARDVGPEAAARLE
YTDATVKEALRLHPVIPAVGRRLHAPMTLGKYVIPAGMLVVPSVYLTHRLASLYPEPTAF
RPERFLDKKPDPYAWYPFGGGVRRCLGMAFALYEMKIVIATILSRVRLRKAHPAPERTVI
RAISFVPERGAEVVLEAKLGAGATAAAAA

>A7982_11355|KEGG|CYP253K1|[Minicystis rosea]|mrm|DP

MPARRNDVPPGPPGYPLVGALPAFARDPLGFLSGATRRHGDMLFLGKVLGRGVYVLTRPD
HLKYILQENNRNYIKGNGLAALKLVVGEGLVVAEGERWHRQRRLVQPAFHRQRVAALVDQ
MADVIEKAHARWREKTAQEPVPVLFEMMRLTQRVLLSTMFGVDIESKADSLCKAWDVVVA
VLTERLLSPPIHLPISVPTPKHLRFRRAMQTLDDAIYGILRDPESADRGLLSMLRAARDE
VTGEGMSDRELRDEVMTIFAGGFETSAVALSWVWHVLGTHPEVEAKLHAEIDEVLGDRRP
RMEDLPKLRYTKMVVEETLRLYPSAWIFARMNLAADEIDGYPIPPRSLLFVVPYLTHRHL
GFWPDPETFDPERFTPDRSASRPRFAYVPFGGGPRLCVGEAFAMTEMQTVVAMTARRFRF
RPVPGREVRERGMFTLRARPDIIMSIEERRPAQRSIEEPTAAI

>A7982_11494|KEGG|CYP1069H1|[Minicystis rosea]|mrm|DP

MTRAFSYFAPETQQDPYPQYAAARAEEPIFFCEDLGMWVATRHADVTAILRDTDRFSSRL
TTVAVKEPPPEVVAVMRRGFPRTGSIIHLDPPEHTQVRRRMNAAFTAERVAAMEGAVTTL
ANELVDAFEAEGRADLVRRFCGPLPVSVIADILGVSRADAGKFGQWANDAARLLMSGNLA
TDEWVRAAESVVTMQHHLADLITARKSAPTDDLLTALVQTATDANGQLDMAKAVGYATAF
VFAGHKTTTDLLGNALRLLLLHPEQLAALVRDSSLASAVVEETLRRDCPVPGTARVANVD
VKIGDVTIPKDARILALLGSANHDENLFEASSKFDIGRTDVGDHLAFGRGVHFCLGAPLA
RLQGRVALTVLTQRLRGLRFASEQPVKFWQVTMFRGPVSLDVAWDVAK

>A7982_11629|KEGG|CYP110S1|[Minicystis rosea]|mrm|DP

MPSLPPGPRSTLISSFDLGLGPVHALARAHARYGDPFALPTFNGSLILTARPELIGEVLT
QKDTELFEPFADDTLSPFFGTHSLLLIKGETHRRERRLLMPPFHGERMRAYGATMAEVAR
EAVAALAPGQAFRAIDLGMEISLEVIVRAVFGVEGRAQVDEHLAALKATLDATSPFFLFG
KAFQRAPFGLGPWARYEASSARVDRLLFDQIERVRPRAAERTDILSLMVAARYDDGSSMT
PQQIRDELRTLLIAGHETTAITLAWAFELIHRHPHVRDRLLAELDAAGDAPDELAKLPYL
TAIVDETLRLRPVVEMVARKLRRPWTFAGYDLPTGATIAPAISLVHMRPDLYPDPLQFKP
ERMLEKKPGPFEYMPFGGGNRRCLGAAMSHYESCIVLATALRGFRFALREPEPVPFARRT
FTVGPKTGVRMEMLGVRREAALRNVA

>A7982_12497|KEGG|CYP109P1|[Minicystis rosea]|mrm|DP

MADCDAPETRDLYRAMREKRPVFFDPRYRSFCVFRYDDVRTVLTDWERFSADYGAMAPGS
ALDRIHQGSMNASDPPRHQRLRGLVSRAFTPKSIAEMAPEIARTTHALLDELAPRGACEL
MEDFAGVLPLLIIADILGIPRADHAHFRRLTLELLETFDTIVSGAPTSNAAQEAMEAYFS
RIIEERRVDPRDDLTSRLIAAELDGERLDDREILTFMKLLLVAGNSTTSRLVGNAVVTLL
EHPGELERLRADPKLVPSAVEEVLRFRPPINTWFRIAAKDVTLGGEAIAARQQVVAFIGS
ANRDEAYFTDPDRFDITRSPNPHIALSSGIHFCLGASLARLEAQIALQAILERLPDLALA
GSEPLEVTKGLQSNGFVRLPVRYRAA

>A7982_13464|KEGG|CYP1007E1|[Minicystis rosea]|mrm|DP

MTTRSPFASLAGPPPMPFFGWRGNMVRFFQDTIGYMAPLARRYGDVVSFAAGGSGAVLVR
QPESRGAVFAFGPACHQAVLGQMSVFHSTRVPGPPESRSFARITSGLFNMNDDKHRQQRR
LMQPAFHRKRLDGYLDTMVRFTADVLDGFRVGETRDLVREMERLTLAVANKTLFGLEPTP
GVISLGERIQEVIGRSMSPGALVPINLPGSPRRKLIEAADRVDAELRAVIATKRASGIDG
DDVLSTLIATRDEAGDALADDELIGQTFILFLAGHDTTKSALAWTLFLLEQHPRIFADVL
DELRGVLRGAAPTVAQLGELPLLERVIKESLRLLPPAPLTARLTMQPTALGGVELPAGTE
VIISPYCLHRYPDLYTEAQRFLPDRWASIERSPFEYAPFGAGARMCIGAAFAMMELKTVL
SMFLQRFGVGLAKGATIDRRTTVVMAPRYGMPMILRPPGAPAEPGVARGNVREMIDLPQ

>Hoch_0067|KEGG|CYP107HZ1|[Haliangium ochraceum]|hoh|DP

MTDSRDAAKPATSAAGAPIFNPLDPAFLRNPYPVYRGLQDQAPVLLTPLGVYVVTRYQDV
QQVLRGPGMHHRFEDMARRRVGDKAKTSPAFRSLGQWILTGNPPDHTRIRGLMVKAFSAA
RVESMRPRVQQLVDDLIDQMLAAADSAAPRADLVGALARPLPVTVICELLGIPREDWPRF
TGDSYLPRFLVEPRPLSPSELDAVDSVVSEIGEYLGALCDSRRAAPQDDLISALVQASEG
DDGKLTHEELVANLIMLFFAGHETTVNLIGNGMLALLGHPAERDKLIADSSLIPNAIDEM
LRYDNSVQLAGVRCNEAAVTLSGVEVPAGSQLIPIIGAANHDPRAFDEPERFDVSRSFAN
KPVSFGGGIHFCLGAQLARLEASIAVETLLRRLPDLRLAEGYEPAYFPSFVLRGLRSLEI
AWS

>Hoch_0804|KEGG|CYP107JA1|[Haliangium ochraceum]|hoh|DP

MTTIAHFNPQRADVWTDPYPWYEQYRREDPVHWTLDAERGDAGVWYVFRHEDVLAARVNL
SLSRRYRHASLMKKQLPSYMKPYSDMLGYWMVEMDAPDHTRLRSLVNKAFTPRMIAPLGH
HIRASVNALLDRAAETRELDLIGEFAYPLTLRTIAGVLGAPPEDWPRFKQWSVVFAALEN
RVPTIELYKQASAAMVEFAAYLRELFHRRRGEPLGDDIVSTLIQAEEHDSVLEENEFIGT
CMLLLFAGHETSTNLIGNAFYTLLKHPEQLALLRSEPARVPSVVEEVLRYESPVQTVSIG

HAIEPTAIGGREILPGQKVITAFGSANRDPEVFAEPDRFDITRDAKRHCAFGTGFHNCLG
NQLARLEAQIALEALLERLPDLRLSGPEPQFKEGLMLRGLDQIAVTF

>Hoch_0812|KEGG|CYP262C1|[Haliangium ochraceum]|hoh|DP

MPKAKGYPLVGPLPNLIANSFDFFLKAQETYGDIYLLDVGLMQTVVCNHPRHIRHVYVTK
GQSYRKAGALWEQMKLITGNGLAVSEGDTWRRQRTMLQPVFNKRRLRALGGLMSAAVSEE
LPHWRETIGRERTMDVSPLFARTTMKMTIKLVFGTSLHQDEIDKVQPELQYAVRYLLPAM
LADKVPHWIPIPGRRRYRQAIDTLGSVLMDVIARRRGESGEEGDLIAQLSRLVDESGQPM
SQQQLLDETLTLFLAGYETTANAITWAAHFLSQHSEVMDKLHGELDAVLGGRIPTYEDLA
QLTYTNRVFRETLRLCPPIWWNPRDIEEEDEIDGFYIPPGTTVTPVTYVIQRHPEFWEQP
HVFDPDRFTPERSEGRDRYAWAPFGYGRRSCIGQEMAMMEGVFILAGILQHLQIEPIPGR
EAKIALLGTLRPKNGVHLRLRPRHGGV

>Hoch_0813|KEGG|CYP1490A1|[Haliangium ochraceum]|hoh|DP

MASVAGLPPAAPLARSLAQLARDPLAFLTEGRERYGDIYRLGLGGMHVVLLHHPRHAHHV
FREHYKNYGKGGALWEALRDYLGNGLLVTEGDLWLRQRRLLQPLFKRTNIDMRMSSMYEI
VTRVLDSWGEECAQTGSLDLVSACARLSMGVSTGSMFGSALTHEDTHALMEEVRVVVDSM
VWNMLTRRIPQRLRPGYARYRKAMDHIHSALDGLIDHYQRSEDGDDGLLAMFSYIEDSAT
GARMSRELMRDETLDLLLGAYETTAQALAWTFYCILQHAEVERRLRAEIGAVLGARRPEL
EDLKQLPYVVRVIREALRVYPPGAWITRTTREDDEIDGHHIAAGTTVAVVTYAIHHHPAI
WEQPERFEPERFLPEADAARERCAWIPFGSGPRVCMGMDFAMLELALAVILALQRYDIQR
VTSGPIAPRLRTTLTPAEPLEVRLHLRPGDA

>Hoch_0814|KEGG|CYP1504A1|[Haliangium ochraceum]|hoh|DP

MKHAHPPRLSPFEHTRQLLASPFQWLVDSQTRTGDIARLGIGHQSVLLSSHPEHADHVFR
RHARRYARAGRFWDALPGIFGRGLLMTADETWLRHRRLLQRFFRRDHLLSIAEPLLATIE
DAVARHLGTVRGPRVLDVHPVLEAIVLECMITLVFGTRLSDADRAQVRSQITWLTRRVPQ
ALLYSTLPASLARVVRNRYAENLLAFRRRIAAMAADCRREPSVSDDMLSELVWAASPGGA
VLPDAEIEDEIINVFIAGLDTSVLGVTWAVALTATHPAEGEGISSEGRALGAHRRLRPEH
IDALTRSLWVFQEGLRLYPPIWFSPRLALADDEFGGCELAAGSTVIRMTHVLHRHPQFWA
ESERFLPERFEPGRCPARHRAAWVPYGLGPHVCIGRHLAAILSQAIITILFARYRLELSG
RMPRPLLRASLAPQGGVPVRISPI

>Hoch_1303|KEGG|CYP1489A2|[Haliangium ochraceum]|hoh|DP

MLNDPLKLMTRAFEQHGRISYLPGINSYMISDPEFMRLILLDGENKFIKSPEVMSKIQVA

VGNGLSTLNGGEWKRQRRMSNSAFKPRNIAAFEPIFHEHLGEVMHQWEQRLGQRFDIAQE
MKRLTLRIVLKGLLSLDVTDRADMLIEHLDVLQDYAVYILWSLFPLPENVPTRRNRQYAE
SKRVMDEEIYRIIEQRRRDGEAAGKGDLLATYMHAVDDAGSGMGNTQLRHELMNLFLGGH
DTTANSIAFTLYLLSRNPGCRERLERELDEVLGGRLPTVEDIPKLHYLECVYNESLRLYP
PSSAMSRRTLEPIEYEGYEIPAGADLLLSQWAMHRDPTLWENPDVFDPDRFTPERSANRH
KFAFVPFGAGPRICIGAKLARMEASMILAALLQKYRFESPPGYKLKLQSRLFVNAVPGVF
LRLQKREVSEKM

>Hoch_2132|KEGG|CYP167C1|[Haliangium ochraceum]|hoh|DP

MTTTPTSSEFPASPETFDLDMSSEAFVRAPAETYAYLRDRAPAYYWPRHQAWMMTRYDDV
DALLRDPRFSTDRRKWRYAHTLPHASSPELAALFENDLFTMHADDHRRVRKLVAPAFSPR
SIARLEDEVRALVNSLLDDAKIDQRETFDVSQELAEPLPIRVISTMLDIPAEKDEDFRVW
GTSVIRLALPYLPREVLMEAAEKATAGVSMVRAIIAERRQNPGEDILSKLISAQEEGERL
SEDELMSLVAGLLVAGSETTVHLITFATYQFLTHEDSRRRLQDDPSLLGNALEESLRHDN
FGKSGVTRYALEDVELHGKTIEKGDMVILNLASALLDERVWPDAERFDVTRDPVSNITFG
RGAHFCLGAHLARLEGRVAMQTLLERFPEMQLAGPPSFDYAHPFIRRIDKLEVRLRA

>Hoch_2513|KEGG|CYP107HY1|[Haliangium ochraceum]|hoh|DP

MSTEQPIAPAPDSHTAPPTHPDATPAALFADAAFHHHPYPRLARLREAAPVCAITMPDGA
RAWLITRYDEALQVLKDARVTNEPPGGMHPGAMHSGTLERLMSPNMIFMDKADHRRLRGL
LAKAFTPRFAEGLRPAVQQLADELLDAVAARGRMEFISDFAFPLPITVIADMLGLPQEDH
QRLRDWSAVLFDSIGAHDGADREQQANEFAGYVKELIAHKRREPADDLISKLVQAEQDGE
RLDEQELRAMIVLLIFAGHETTVNLLGNGLLALFRQPEQLEALKRDPSLVPNAVEEMLRF
CGPATMLAPRYAREDMQIGGVTIAKDELILLSVAGADRDPAAFSDPESIDLRRPTPQHLA
FGQGMHICIGAPLARMEAQVAFTTLLKRFPELRLTVPVDEVVWRGNFALRGVEALPLAF

>Hoch_2966|KEGG|CYP1486B2|[Haliangium ochraceum]|hoh|DP

MAATSKSQPSPEVAPFITQRVGDVARPLVGMPTTMDLSHLPGEEGPVATVRNTLGWIRSG
VDHLLAQHERFGPVYRTGFAGYRVVCAADPDMVLSIARDNQSWSTSLAWLTFFQGIDSKI
NRAQLDSPLFLDFEPHRNARKVLQPAFGHSATSDYFDAALSLYEAAAERWVARGQVSFKD
AIRSLMVEVSTRIFLGEDDAAPEFERALIEYWEGPLALVRDPRFSRKWRRSIRGHRRLCE
LLRARIASRRASGGDDLFSRLCAMTGTSDGLVDDDGLVRLVIGVMSAAFATTSSGLSSMV
YLLAEHPAWQQRMREELLALDSPRMTYADSQSLDVCMRVWKETMRLYPIAPYCARRALHD
VDLGSYRIPAGTFVFGLISAVLRDSALWTQPERFDPDRFTEERAEDKRHKAAFLPFGIGV
HTCTGMHLANAEVMAFCHAMLTRCRFSLERPYRARHTYAPAGIVSGPVKLRVERL

>Hoch_2967|KEGG|CYP126B3|[Haliangium ochraceum]|hoh|DP

MSVDSTNRPPASETAHSSPTAGALPAVLEGFDLSDQPRFADGFPYEVFARLRREAPVLFH
PPGQTKDGEGFWVLSRHADICEAAASPAFSSQGGGGRPHGGTHIDDARPELPGVLINMMD
DPRHADLKDVLSPAVGRQALVALEGALRPYVNELVDGLLARGEAEFAADVGAAVGARAIS
LLLGIPREDWPLFATWTSALMGFDDRETAEPSERSQKIHMDLFGYGARLLVARRAAPQED
LGSLLANAQLRRDSERPLTELERQTAFCLMVLAGTESTRNMIAGGVLALAQHPAQWQALR
DERSLLPSAIDEILRWTTPTPYNRRTATRDVTLGDAHIRAGDKVTLWWTSANRDESVFKD
PMAFDVRRDPNPHLAFGYGTHCCFGDQLGKLEMRLVLDAMLERVAPLELSGPVVWAASNK
HTVVMDMPVAVRAR

>Hoch_2973|KEGG|CYP110U1|[Haliangium ochraceum]|hoh|DP

MPETVDPSSAQTAGDLGRGPSSAFLTTLRYSIDPVSLYRTLARDYGDGHTVHMPMLLGDV
VGAISPQSAQDILTADAASFDIFSPESLAMVFRPRSVVMLSGQEHARERKLLMPPFSPRQ
VLANYAGTMQETALAYAAEVADGRPFVMQELAQRVLLRVVVRDVFGVTEDAELDELELRI
RELFEASSPALIFFPPLRHRFGGVGPYATWERADRRLTRLIHDLIARRRAEPRGDRVDVL
SLMLSARYPDGSAPSDEVLHDELMALFFAGHAATATSIAWVFYWAHRHPEVLHKLRDELA
ALPWDAEPARYTELPYLDAVCNETLRIYPPVADLYRKLRVPLRVGGRTVPAGTGVAVLVT
CIHARPELFPEPLRFRPERFAERRYSPFEFLPYGAGARRCLGASFAHQALQVVVATILRR
WELALVRREQKAVRQGVGVGPKHGVPMRVVRSLVPERSAAQPAGGRP

>Hoch_3012|KEGG|CYP262D1|[Haliangium ochraceum]|hoh|DP

MNATTTQQPSAEKRFPIEKGLPLVGTLPQLLSSGTQLVERVSRKHPGQITVLKAGPTQVY
LITHPDHVKHVLQHSERGFSKGPMWDPLRRVFGDGIGTSEGSEWLRSRRMAQPLFSTKNI
DSLVGSMSEIVARHVARLAPLVGSGESVDMNVEMMRMVQDVLVATLFGVDVPAQQMTTIA
DAIQRILSSSQLELLLGMVLPHRLLQPMDLLVRRPRQVLDEVIFDLIAQARASDSDSANF
LSWFLRARDIDTGKGLDDQQLRNELISIYVAGLETTMCSLLWLWYHLDQNPEIDGKMRAE
VDAVMGASSITAAKLEQLSYTKMVIQESMRRLPTIWMMPRYSREDNVIDGYKIPADSLIF
LSPYATHRDPKFWERPEAFYPEHFTPERVAARPRYAYYPFGGGPHQCIGKYLAMTDQLLI
VAMMVQRFRPRLLLGSTVDPRPSLFLRPRHAMRMTLERR

>Hoch_4349|KEGG|CYP155H1|[Haliangium ochraceum]|hoh|DP

MSDASAAERPLQGRCPIDHDSATQQKTARAVELPEVPLDCDAEGVWHVRAFELVRELLRS
ADTKQAGFNAESFAEQQQIINAPILFLDGNEHHVQRKQTARFFTPRTVSEKHRSMMESFA
DQLMREFRARKRIELSSVSMKMAVQVAAAVIGLTDSLLPGLSGRLEAFFDDHEPPETLSP
PRRMLAHARSNARTFAFFYLDVKPAIRARRKRPQDDVISHLLERQYSDVEILSECLTYGA

AGMVTTREFISVATWHLLEQPALLEQYLGGDESARQRLLHEILRLEPVVTHLRRRATADI
EIAADGRRYTIPEGALIDLHIGAANLDSAATGPCPLSVQPERELQVPRVGAEVLSFGDGA
HRCPGAYIAIQETDIFLQRLLRLPGLRFAQKPSVSWHPLVTGYELRNFWLELEG

>Hoch_4741|KEGG|CYP1069B2|[Haliangium ochraceum]|hoh|DP

MSHDPTPSLDFDPFSGAQLDDPVPFLNQARATTPVFFSPPLGTWVVTRYDHVVEILRDPA
RFSSAASTTNVPGPPPPPVLEVLSRGVAYAPNSVDLDPPRHTLFRRLINKAFTPRRIESM
GPEIERFAHQLIDDFTGDQCELVAAFAHPLPTLVIASVLGVPRQDLGAFRRWSDEWVVLL
AQRGDMARLVQAAGEVVAFQRYVADLVAQRRRAPQDDLISAIVEAAAELEEAPTEAELVS
LLMTVMFAGHETTTSLIVNTVKQLGLHPVERDRALADRGLIPAAISESLRLDPPVPSMYR
TATEAVEIGGVALPAGAHIQLNFAAANRDPARFDEPERFDLERAEPERHLSFGRGIHYCV
GAALALLEGRIAIAALYDRLPGLELVPEQRIRIRPSATVRIPERVLARWRAEV

>Hoch_5488|KEGG|CYP110H3|[Haliangium ochraceum]|hoh|DP

MSTLPPGPSGKLRPTYRLLTKPRTAIPAWSARYGDPFTLKSLTGHVVVTGNPEGNRAIFS
ADPDTFDTFAANALEPFVGQYSMLLLSGEVHRRHRKLLMPPFHGERMRAYAAVMAEVARR
KLREAGAGPVRAIEVAQEISIDVIVRAIFGIHDDDRAAELCDAVRRAMGALHPALAFMPF
LQRRFGGIGPYARMRRYLDYSDSLVAEQMKRARTQPGDDILSLMLAARDDEGQPMSEVEI
LDELRTLVFAGHETTALLLAWGIDFIHRHPAVLKRLLAEIDQVDDSADDAAERYAQLPYL
DAVCKETLRLQPPVLEVLRTLRVPFELCGYTVPPGMGVAASVQLTHSRPDLYPEPEAFRP
ERFLERKFSPFEYFPFGGGNRRCIGAAFSSFEAKVVLATLLANWQVRLLDPEPPVPVRAS
VVITPKGGVPIELTSRQRPN

>Hoch_5532|KEGG|CYP107HV4|[Haliangium ochraceum]|hoh|DP

MIDIPDITRPRHKANPYPLYARLRAEAPAVRVIFGDKRPAWLITRHADVSAALKDPRLSK
NPFVTLSAEEQRKQLPWIPAFARPLSKNMLDQDPPDHGRLRRLVHKVFTPRMVAELRTRI
EAITGDLLGAARARGRFDLLEDFAMPLPITVICEMLGVPEHERRRFRILSNRMVSIVTPS
EMLRGLPAVWLLVRYLRGLIARRRREGGEDMLAVLVRAEEDGDRLDEDELLAMTLLLLVA
GHETTMNLIASGTLALLDAPEEFARLAGEPTLMASAVEELLRFTSPVDVATERYASTELQ
VGGQVIPRGERVFAVLGSANRDGSVFDHPDALMLDRSPNRHVAFGHGPHYCLGGPLARLE
GEIAFAAICNELRGLALDVPRDSLRWKRSPVLRGLRSLPVRCDGAPAYSLPRAA

>Hoch_6014|KEGG|CYP1220C1|[Haliangium ochraceum]|hoh|DP

MHRLPGPRRLPLVGNLHQIELSRLHLILEDWARRYGSAYRIGLGRRPNLVLSDPELIRAV

LRDRPEGFRRNRTLETVLSEMGISGVFSAEGAAWRPQRRLATAALSRSHIQGFFPTLRLG
GERLLRRWQRAADQGAIVNILTDFQRFAVDVMTSLSFGRDVNTIEDGGGGVHELLETVFP
VLARRLNARVPIWRWLPSDADRALDDALVELRAWLGAIIAEARSELAANPDGVTKNFLEA
MLHARDEDGAAFDEAALFGNALTMLLAGEDTTASSLAWAVHELSDDAEVVARLRAELDGE
ISSAVPKTLREVARLPYALAVAEETLRLRPAAPVLLMESNRDAVLGEDIAVPAGTRVLLL
TRLAAISEAYVPEPQRFLPARWLDRERASELLRRGVHLPFGSGPRLCPGRNLAFIEMQMM
LAVLYRNFEVERIGPRSAVAENYGFTMTPHRLRVRLRRRAHAPAPHAAAAPRMEQR

>DN745_16145|KEGG|CYP197AB1|[Bradymonas sediminis]|bsed|DP

MKLRSLIREKVAQKLDPSAGVARPPGPGALETLKTHLSFAGDPIGSLRKLTDKHGPTAVF
RLGPYTNYLFTNPEDIEEVLLRKNDAFHKDTMLHELDAVLGQGLLTAEGDYWRGQRKLIS
PTLRRKHIESYAEVMVSHTKDMMADWKTGEVRDFHADIMGVTLRIVVKTLFNLELEADYH
EVGEQLEAALDYFFERNNTMWRFMPEPLPTPMRAKNKRAIEGLDRLIYELIESRRRQARA
DTRAGVDEGNDLLYRLLIAVDDEGNKMDNRQVRDEALTFLLAGHETTALAVTYAWHLMAA
HPDAAARLREEVDAVLDGRAPTAEDAKKLPYTRALLNETLRLYPPAWIIGREAIEDVQIG
DWTVPRGAQVLTPQALVHRDPRWFDAPDTFRPERWLGDLEKTLPRFAFFPFGGGPRVCIG
NYFAIMEATLIIATMAQQIELENVSTEPLRIRPSVTQRPLSEIKMKVTRR

>FRD00_08280|KEGG|CYP110AU1|[Persicimonas caeni]|pcay|DP

MPMPPSPSMSPREQTFGWMTRPYELLARCRQEVGDVFTIDFRGHGQYVLFSDPEHIHQIL
TGDPAVFHAANGLLEPFLGAHSLLLLEEERHKRERRLMMPHFSRRQIHGWGDQIEEVADR
MIDGWRVGEEMPMRETAQSVALGLIDRLVFGLDGEANHDRHDALHRCFDEILSHPHFNIA
LIGQFGERLAGSPAWQQLQESLAEMDRLVREEIAERRATGRRGDDIMSLLMDAEYEDGES
LSDDELRDELVTLLATGHETTATSLAWAVHWVWSHPPVLERLRAELATLGPDASPSQIAE
LDYLQSVVLETLRINPVVPIIARELQEPVQIAGYELPAGVTVAPVIYLLHRREDLYDEPG
QFRPSRFLERDYKHNEFMPFGGGVRRCLGMHLALYELQILLARIATRVELRLCDEEEVRP
VRRMVTIAPSGGVRFEVERVGA

>FRD00_16705|KEGG|CYP197AB2|[Persicimonas caeni]|pcay|DP

MFGLKDGLNLVDRFKSGESAPARPPGPRGWQNLKVLVDFATDQIGCFRRITRRYGSASFF
KLGTFDAYLFTDPEAIEEVLLTKSSSFEKDALTHELDVLLGKGLLTSEGQMWRHQRRLIS
PNLRRKQIAHYADVMVERTRQMLDDWEDGQVRPLHRDAMEVTLRIVVDTLFNLEMDSDIH
RVGQALDTAMEGFHEQAHTLWRFVPEPLPTPMRAKFEKALEEFDALIYKLIDQRRQDATE
GDDLLYRLIAAVDDEGNQMTDGQLRDEVITLFLAGHETTALAIMYAWYLMSDHPWVMDKV
HAEVDEVLGERVATADDVSELPYTEAVIQETMRLYPPAWTIGREAIEDVEIAGWTVTKGA
QVLLPQSLVHRDRRWFDNPDLFRPERWLDGLEDRIPRFAYFPFGGGPRICIGNYFAMMEA

ILVVATMAQQVRLENVSRQALRTQPSVTQRPATAIEMKVRRR

Epsilonproteobacteria

>A0Z60_06705|KEGG|CYP172A|[Helicobacter winghamensis]hwi|EP
MGVCPFHPKPYKTKASTFATFLFKRRSWLDGLYERSYKMMMGRVKMPGFDLYVVNDPKEV
RHVMVDDVKNFPKSQLLHELLEPLLGMSIFTTNDKMWKKQRELLRPSFEMTRISKVFNLM
RDATDDMMRKLASCQNGAVIEVDAIMTFVTADVIFRTIMSSKLDEAKGKLVLEAFGTVQE
ETTKTALRTMFCFPKWLSRLLGENKRLKAASVIRKVLSDIIAPRYHNAQSDLGKYEDILS
SLLQVVDAETHQRFTFEEILDQVSMLFLAGHETTASSLTWTLYILSIAPNEQEKAYKEII
RVAGDGVFEIEHIKEMHYVASIFKEALRLYPPVGFFARETREDRKMRDKLVKKGSGVVVA
PWLIHRHERFWKNPHEFDPTRHESKSNIKKDTYMPFGMGERICIGQGFAMQESVLILANI
IRKYKLELEENFVPDIVGRLTIRSANGMRIKFIKRNQ

>Cj1411c|KEGG|CYP172A1|[Campylobacter jejuni subsp. jejuni NCTC 11168 = ATCC 700819]cje|EP

MSECPFFPKPYKNKASTLLTFLLKRRSWLDGLYERSYKMQTGYVKMPNFDLYVINDTKEV
KRMMVDEVREFPKSAFLHELLSPLLGESIFTTNGEVWKKQRELLRPSFEMTRINKVFNLM
SEAVADMMDRFSKYPNHAVIEVDEAMTFITADVIFRTIMSSKLDEEKGKKILNAFVTFQE
QSVHTAMRRMFRFPKWLSYVLGDCKRAKAGDVIRQVLSDIIKPRYDMADNAEFEDILGSL
LLVVDADTNKRFSFEEILDQVAMLFLAGHETTASSLTWTLYLLSLYPKEQEKAYEEITQV
LQGGVIEISHLRQFKYLTNIFKESLRLYPPVGFFAREAKKDTQVRDKLIKKGSGVVIAPW
LIHRHEEFWTNPHGFNPSRFEGEYKKDAYLPFGVGERICIGQGFAMQEAILILANILKTY
KLELEEGFVPDVVGRLTVRSANGMRIKFSKRKL

>BN148_1411c|KEGG|CYP172A1|[Campylobacter jejuni subsp. jejuni NCTC 11168-BN148]cjb|EP

MSECPFFPKPYKNKASTLLTFLLKRRSWLDGLYERSYKMQTGYVKMPNFDLYVINDTKEV
KRMMVDEVREFPKSAFLHELLSPLLGESIFTTNGEVWKKQRELLRPSFEMTRINKVFNLM
SEAVADMMDRFSKYPNHAVIEVDEAMTFITADVIFRTIMSSKLDEEKGKKILNAFVTFQE
QSVHTAMRRMFRFPKWLSYVLGDCKRAKAGDVIRQVLSDIIKPRYDMADNAEFEDILGSL
LLVVDADTNKRFSFEEILDQVAMLFLAGHETTASSLTWTLYLLSLYPKEQEKAYEEITQV
LQGGVIEISHLRQFKYLTNIFKESLRLYPPVGFFAREAKKDTQVRDKLIKKGSGVVIAPW
LIHRHEEFWTNPHGFNPSRFEGEYKKDAYLPFGVGERICIGQGFAMQEAILILANILKTY
KLELEEGFVPDVVGRLTVRSANGMRIKFSKRKL

>CJJ81176_1410|KEGG|CYP172A|[Campylobacter jejuni subsp. jejuni 81-176]cjj|EP

MSECPFFPKPYKNKASTLLTFLLKRRSWLDGLYERSYKMQTGYVKMPNFDLYVINDTKEV
KRMMVDEVREFPKSAFLHELLSPLLGESIFTTNGEVWKKQRELLRPSFEMTRINKVFNLM
SEAVADMMDRFSKYPNHAVIEVDEAMTFITADVIFRTIMSSKLNEEKGKKILNAFVTFQE
QSVHTAMRRMFRFPKWLSYVLGDRKRAKAGDVIRQVLSDIIKPRYDMADNAEFEDILGSL
LLVVDADTNKRFSFEEILDQVAMLFLAGHETTASSLTWTLYLLSLYPKEQEKAYEEITQV
LQGGAIEISHLRQFKYLINIFKESLRLYPPVGFFAREAKKDTQVRDKLIKKGSGVVIAPW
LIHRHEEFWTNPHGFNPSRFEGEYKKDAYLPFGVGERICIGQGFAMQEAILILANILKTY
KLELEEGFVPDVVGRLTVRSANGMRIKFSKREL

>C8J_1325|KEGG|CYP172A|[Campylobacter jejuni subsp. jejuni 81116]cju|EP

MSECPFFPKPYKNKASTLLTFLLKRRSWLDGLYERSYKMQTGYVKMPNFDLYVINDTKEV
KRMMVDEVREFPKSAFLHELLSPLLGESIFTTNGEVWKKQRELLRPSFEMTRINKVFNLM
SEAVADMMDRFSKYPNHAVIEVDEAMTFITADVIFRTIMSSKLDEEKGKKILNAFVTFQE
QSVHTAMRRMFRFPKWLSYILGDRKRAKAGDVIRQVLSDIIKPRYDMADNAEFEDILGSL
LLVVDADTNKRFSFEEILDQVAMLFLAGHETTASSLTWTLYLLSLYPKEQEKAYEEITQV
LQGGAIEISHLRQFKYLTNIFKESLRLYPPVGFFAREAKKDTQVRDKLIKKGSGVVIAPW
LIHRHEEFWTNPHGFNPSRFEGEYKKDAYLPFGVGERICIGQGFAMQEAILILANILKTY
KLELEEDFVPDVVGRLTVRSANGMRIKFSKREL

>ICDCCJ07001_1342|KEGG|CYP172A|[Campylobacter jejuni subsp. jejuni ICDCCJ07001]cjn|EP

MQTGYVKMPNFDLYVINDTKEVKRMMVDEVREFPKSAFLHELLSPLLGESIFTTNGEVWK
KQRELLRPSFEMTRINKVFNLMSEAVADMMDRFSKYPNHAIIEVDEAMTFITADVIFRTI
MSSKLDEEKGKKILNAFVTFQEQSVHTAMRRMFRFPKWLSYVLGDRKRAKAGDVIRQVLS
DIIKPRYDMADNAEFEDILGSLLLVVDADTNKRFSFEEILDQVAMLFLAGHETTASSLTW
TLYLLSLYPKEQEKAYEEITQVLQGGAIEISHLRQFKYLTNIFKESLRLYPPVGFFAREA
KKDTQVRDKLIKKDSGVVIAPWLIHRHEEFWTNPHGFNPSRFEGEYKKDAYLPFGVGERI
CIGQGFAMQEAILILANILKTYKLELEEGFVPDVVGRLTVRSANGMRIKFSKREL

>CJSA_1342|KEGG|CYP172A|[Campylobacter jejuni subsp. jejuni IA3902]cji|EP

MSECPFFPKPYKNKASTLLTFLLKRRSWLDGLYERSYKMQTGYVKMPNFDLYVINDTKEV
KRMMVDEVREFPKSAFLHELLSPLLGESIFTTNGEVWKKQRELLRPSFEMTRINKVFNLM
SEAVADMMDRFSKYPNHAVIEVDEAMTFITADVIFRTIMSSKLDEEKGKKILNAFVTFQE
QSVHTAMRRMFRFPKWLSYVLGDRKRAKAGDVIRQVLSDIIKPRYDMADNAEFEDILGSL
LLVVDADTNKRFSFEEILDQVAMLFLAGHETTASSLTWTLYLLSLYPKEQEKAYEEITQV
LQGGAIEISHLRQFKYLTNIFKESLRLYPPVGFFAREAKKDTQVRDKLIKKGSGVVIAPW
LIHRHEEFWTNPHGFNPSRFEGEYKKDAYLPFGVGERICIGQGFAMQEAILILANILKTY
KLELEEGFVPDVVGRLTVRSANGMRIKFSKRKL

>CJM1_1368|KEGG|CYP172A|[Campylobacter jejuni subsp. jejuni M1]cjm|EP

MSECPFFPKPYKNKASTLLTFLLKRRSWLDGLYERSYKMQTGYVKMPNFDLYVINDTKEV
KRMMVDEVREFPKSAFLHELLSPLLGESIFTTNGEVWKKQRELLRPSFEMTRINKVFNLM
SEAVADMMDRFSKYPNHAVIEVDEAMTFITADVIFRTIMSSKLDEGKGKKILNAFVTFQE
QSVHTAMRRMFRFPKWLSYVLGDRKRTKAGDVIRQVLSDIIKPRYDMADNAEFEDILGSL
LLVVDADTNKRFSFEEILDQVAMLFLAGHETTASSLTWTLYLLSLYPKEQEKAYEEITQV
LQGGAIEISHLRQFKYLTNIFKESLRLYPPVGFFAREAKKDTQVRDKLIKKGSGVVIAPW
LIHRHEEFWTNPHGFNPSRFEGEYKKDAYLPFGVGERICIGQGFAMQEAILILANILKTY
KLELEEGFVPDVVGRLTVRSANGMRIKFSKRKL

>CJS3_1506|KEGG|CYP172A1|[Campylobacter jejuni subsp. jejuni S3]cjs|EP

MSECPFFPKPYKNKASTLLTFLLKRRSWLDGLYERSYKMQTGYVKMPNFDLYVINDTKEV
KRMMVDEVREFPKSAFLHELLSPLLGESIFTTNGEVWKKQRELLRPSFEMTRINKVFNLM
SEAVADMMDRFSKYPNHAVIEVDEAMTFITADVIFRTIMSSKLDEEKGKKILNAFVTFQE
QSVHTAMRRMFRFPKWLSYVLGDCKRAKAGDVIRQVLSDIIKPRYDMADNAEFEDILGSL
LLVVDADTNKRFSFEEILDQVAMLFLAGHETTASSLTWTLYLLSLYPKEQEKAYEEITQV
LQGGVIEISHLRQFKYLTNIFKESLRLYPPVGFFAREAKKDTQVRDKLIKKGSGVVIAPW
LIHRHEEFWTNPHGFNPSRFEGEYKKDAYLPFGVGERICIGQGFAMQEAILILANILKTY
KLELEEGFVPDVVGRLTVRSANGMRIKFSKRKL

>A911_06865|KEGG|CYP172A|[Campylobacter jejuni subsp. jejuni PT14]cjp|EP

MSECPFFPKPYKNKASTLLTFLLKRRSWLDGLYERSYKMQTGYVKMPNFDLYVINDTKEV
KRMMVDEVREFPKSAFLHELLSPLLGESIFTTNGEVWKKQRELLRPSFEMTRINKVFNLM
SEAVADMMDRFSKYPNHAVIEVDEAMTFITADVIFRTIMSSKLDEEKGKKILNAFVTFQE
QSVHTAMRRMFRFPKWLSYVLGDRKRAKAGDVIRQVLSDIIKPRYDMADNAEFEDILGSL
LLVVDADTNKRFSFEEILDQVAMLFLAGHETTASSLTWTLYLLSLYPKEQEKAYEEITQV
LQGGAIEISHLRQFKYLTNIFKESLRLYPPVGFFAREAKKDTQVRDKLIKKGSGVVIAPW
LIHRHEEFWTNPHGFNPSRFEGEYKKDAYLPFGVGERICIGQGFAMQEAILILANILKTY
KLELEEGFVPDVVGRLTVRSANGMRIKFSKRKL

>N564_01408|KEGG|CYP172A1|[Campylobacter jejuni subsp. jejuni 00-2426]cjej|EP

MSECPFFPKPYKNKASTLLTFLLKRRSWLDGLYERSYKMQTGYVKMPNFDLYVINDTKEV
KRMMVDEVREFPKSAFLHELLSPLLGESIFTTNGEVWKKQRELLRPSFEMTRINKVFNLM
SEAVADMMDRFSKYPNHAVIEVDEAMTFITADVIFRTIMSSKLDEEKGKKILNAFVTFQE
QSVHTAMRRMFRFPKWLSYVLGDCKRAKAGDVIRQVLSDIIKPRYDMADNAEFEDILGSL
LLVVDADTNKRFSFEEILDQVAMLFLAGHETTASSLTWTLYLLSLYPKEQEKAYEEITQV

LQGGVIEISHLRQFKYLTNIFKESLRLYPPVGFFAREAKKDTQVRDKLIKKGSGVVIAPW
LIHRHEEFWTNPHGFNPSRFEGEYKKDAYLPFGVGERICIGQGFAMQEAILILANILKTY
KLELEEGFVPDVVGRLTVRSANGMRIKFSKRKL

>N565_01448|KEGG|CYP172A1|[Campylobacter jejuni subsp. jejuni 00-2538]cjeu|EP

MSECPFFPKPYKNKASTLLTFLLKRRSWLDGLYERSYKMQTGYVKMPNFDLYVINDTKEV
KRMMVDEVREFPKSAFLHELLSPLLGESIFTTNGEVWKKQRELLRPSFEMTRINKVFNLM
SEAVADMMDRFSKYPNHAVIEVDEAMTFITADVIFRTIMSSKLDEEKGKKILNAFVTFQE
QSVHTAMRRMFRFPKWLSYVLGDCKRAKAGDVIRQVLSDIIKPRYDMADNAEFEDILGSL
LLVVDADTNKRFSFEEILDQVAMLFLAGHETTASSLTWTLYLLSLYPKEQEKAYEEITQV
LQGGVIEISHLRQFKYLTNIFKESLRLYPPVGFFAREAKKDTQVRDKLIKKGSGVVIAPW
LIHRHEEFWTNPHGFNPSRFEGEYKKDAYLPFGVGERICIGQGFAMQEAILILANILKTY
KLELEEGFVPDVVGRLTVRSANGMRIKFSKRKL

>N755_01445|KEGG|CYP172A1|[Campylobacter jejuni subsp. jejuni 00-2544]cjen|EP

MSECPFFPKPYKNKASTLLTFLLKRRSWLDGLYERSYKMQTGYVKMPNFDLYVINDTKEV
KRMMVDEVREFPKSAFLHELLSPLLGESIFTTNGEVWKKQRELLRPSFEMTRINKVFNLM
SEAVADMMDRFSKYPNHAVIEVDEAMTFITADVIFRTIMSSKLDEEKGKKILNAFVTFQE
QSVHTAMRRMFRFPKWLSYVLGDCKRAKAGDVIRQVLSDIIKPRYDMADNAEFEDILGSL
LLVVDADTNKRFSFEEILDQVAMLFLAGHETTASSLTWTLYLLSLYPKEQEKAYEEITQV
LQGGVIEISHLRQFKYLTNIFKESLRLYPPVGFFAREAKKDTQVRDKLIKKGSGVVIAPW
LIHRHEEFWTNPHGFNPSRFEGEYKKDAYLPFGVGERICIGQGFAMQEAILILANILKTY
KLELEEGFVPDVVGRLTVRSANGMRIKFSKRKL

>N135_01500|KEGG|CYP172A1|[Campylobacter jejuni subsp. jejuni 00-2425]cjei|EP

MSECPFFPKPYKNKASTLLTFLLKRRSWLDGLYERSYKMQTGYVKMPNFDLYVINDTKEV
KRMMVDEVREFPKSAFLHELLSPLLGESIFTTNGEVWKKQRELLRPSFEMTRINKVFNLM
SEAVADMMDRFSKYPNHAVIEVDEAMTFITADVIFRTIMSSKLDEEKGKKILNAFVTFQE

QSVHTAMRRMFRFPKWLSYVLGDCKRAKAGDVIRQVLSDIIKPRYDMADNAEFEDILGSL
LLVVDADTNKRFSFEEILDQVAMLFLAGHETTASSLTWTLYLLSLYPKEQEKAYEEITQV
LQGGVIEISHLRQFKYLTNIFKESLRLYPPVGFFAREAKKDTQVRDKLIKKGSGVVIAPW
LIHRHEEFWTNPHGFNPSRFEGEYKKDAYLPFGVGERICIGQGFAMQEAILILANILKTY
KLELEEGFVPDVVGRLTVRSANGMRIKFSKRKL

>H730_08010|KEGG|CYP172A1|[Campylobacter jejuni subsp. jejuni R14]cjer|EP

MSECPFFPKPYKNKASTLLTFLLKRRSWLDGLYERSYKMQTGYVKMPNFDLYVINDTKEV
KRMMVDEVREFPKSAFLHELLSPLLGESIFTTNGEVWKKQRELLRPSFEMTRINKVFNLM
SEAVADMMDRFSKYPNHAVIEVDEAMTFITADVIFRTIMSSKLDEEKGKKILNAFVTFQE
QSVHTAMRRMFRFPKWLSYVLGDCKRAKAGDVIRQVLSDIIKPRYDMADNAEFEDILGSL
LLVVDADTNKRFSFEEILDQVAMLFLAGHETTASSLTWTLYLLSLYPKEQEKAYEEITQV
LQGGVIEISHLRQFKYLTNIFKESLRLYPPVGFFAREAKKDTQVRDKLIKKGSGVVIAPW
LIHRHEEFWTNPHGFNPSRFEGEYKKDAYLPFGVGERICIGQGFAMQEAILILANILKTY
KLELEEGFVPDVVGRLTVRSANGMRIKFSKRKL

>MTVDSCj20_1387|KEGG|CYP172A|[Campylobacter jejuni subsp. jejuni MTVDSCj20]cjv|EP

MSECPFFPKPYKNKASTLLTFLLKRRSWLDGLYERSYKMQTGYVKMPNFDLYVINDTKEV
KRMMVDEVREFPKSAFLHELLSPLLGESIFTTNGEVWKKQRELLRPSFEMTRINKVFNLM
SEAVADMMDRFSKYPNHAVIEVDEAMTFITADVIFRTIMSSKLDEGKGKKILNAFVTFQE
QSVHTAMRRMFRFPKWLSYVLGDRKRTKAGDVIRQVLSDIIKPRYDMADNAEFEDILGSL
LLVVDADTNKRFSFEEILDQVAMLFLAGHETTASSLTWTLYLLSLYPKEQEKAYEEITQV
LQGGAIEISHLRQFKYLTNIFKESLRLYPPVGFFAREAKKDTQVRDKLIKKGSGVVIAPW
LIHRHEEFWTNPHGFNPSRFEGEYKKDAYLPFGVGERICIGQGFAMQEAILILANILKTY
KLELEEGFVPDVVGRLTVRSANGMRIKFSKRKL

>PJ17_07425|KEGG|CYP172A|[Campylobacter jejuni subsp. jejuni 00-1597]cjl|EP

MSECPFFPKPYKNKASTLLTFLLKRRSWLDGLYERSYKMQTGYVKMPNFDLYVINDTKEV

KRMMVDKVREFPKSAFLHELLSPLLGESIFTTNGEVWKKQRELLRPSFEMTRINKVFNLM
SEAVADMMDRFGKYPNHAVIEVDEAMTFITADVIFRTIMSSKLDEGKGKKILNAFVTFQE
QSVHTAMRRMFRFPKWLSYVLGDRKRAKAGDVIRQVLSDIIKPRYDMADNAEFEDILGSL
LLVVDADTNKRFSFEEILDQVAMLFLAGHETTASSLTWTLYLLSLYPKEQEKAYEEITQV
LQGGVVEISHLRQFKYLTNIFKESLRLYPPVGFFAREAKKDTQVRDKLIKKGSGVVIAPW
LIHRHEEFWTNPHGFNPSRFEGEYKKDAYLPFGVGERICIGQGFAMQEAILILANILKTY
KLELEEGFVPDVVGRLTVRSANGMRIKFSKRKL

>PJ18_07210|KEGG|CYP172A|[Campylobacter jejuni subsp. jejuni 00-6200]cjw|EP

MSECPFFPKPYKNKASTLLTFLLKRRSWLDGLYERSYKMQTGYVKMPNFDLYVINDTKEV
KRMMVDEVREFPKSAFLHELLSPLLGESIFTTNGEVWKKQRELLRPSFEMTRINKVFNLM
SEAVADMMDRFSKYPNHAIIEVDEAMTFITADVIFRTIMSSKLDEEKGKKILNAFVTFQE
QSVHTAMRRMFRFPKWLSYVLGDRKRAKAGDVIRQVLSDIIKLRYDMADNAEFEDILGSL
LLVVDADTNKRFSFEEILDQVAMLFLAGHETTASSLTWTLYLLSLYPKEQEKAYEEITQV
LQGGAIEISHLRQFKYLTNIFKESLRLYPPVGFFAREAKKDTQVRDKLIKKDSGVVIAPW
LIHRHEEFWTNPHGFNPSRFEGEYKKDAYLPFGVGERICIGQGFAMQEAILILANILKTY
KLELEEGFVPDVVGRLTVRSANGMRIKFSKREL

>cjr:CJE1598|KEGG|CYP172A1|[Campylobacter jejuni RM1221]cjr|EP

MSECPFFPKPYKNKASTLLTFLLKRRSWLDGLYERSYKMQTGYVKMPNFDLYVINDTKEV
KRMMVDEVREFPKSAFLHELLSPLLGESIFTTNGEVWKKQRELLRPSFEMTRINKVFNLM
SEAVADMMDRFSKYPNHAVIEVDEAMTFITADVIFRTIMSSKLDEEKGKKILNAFVTFQE
QSVHTAMRRMFRFPKWLSYVLGDCKRAKAGDVIRQVLSDIIKPRYDMADNAEFEDILGSL
LLVVDADTNKRFSFEEILDQVAMLFLAGHETTASSLTWTLYLLSLYPKEQEKAYEEITQV
LQGGVIEISHLRQFKYLTNIFKESLRLYPPVGFFAREAKKDTQVRDKLIKKGSGVVIAPW
LIHRHEEFWTNPHGFNPSRFEGEYKKDAYLPFGVGERICIGQGFAMQEAILILANILKTY
KLELEEGFVPDVVGRLTVRSANGMRIKFSKRKL

>JJD26997_1744|KEGG|CYP172A|[Campylobacter jejuni subsp. doylei 269.97]cjd|EP

MSECPFFPKPHKNKASTLLTFLLKRRSWLDGLYERSYKMQTGYVKMPNFDLYVINDTKEV
KRMMVDEVREFPKSAFLHELLSPLLGESIFTTNGEVWKKQRELLRPSFEMTRINKVFNLM
SEAVADMMDRFSKYPNHAIIEVDEAMTFITADVIFRTIMSSKLDEEKGKKILNAFVTFQE
QSVHTAMRRMFRFPKWLSYVLGDRKRAKAGDVIRQVLSNIIKPRYDMADNAEFEDILGSL
LLVVDADTNKRFSFEEILDQVAMLFLAGHETTASSLTWTLYLLSLYPKEQEKAYEEITQI
LQGGAIEISHLRQFKYLTNIFKESLRLYPPVGFFAREAKKDTQVRDKLIKKGSGVVIAPW
LIHRHEEFWTNPHGFNPSRFEGEYKKDAYLPFGVGERICIGQGFAMQEAILILANILKTY
KLELEEGFVPDVVGRLTVRSANGMRIKFSKREL

>M635_02730|KEGG|CYP172A1|[Campylobacter jejuni 32488]cjz|EP

MSECPFFPKPYKNKASTLLTFLLKRRSWLDGLYERSYKMQTGYVKMPNFDLYVINDTKEV
KRMMVDEVREFPKSAFLHELLSPLLGESIFTTNGEVWKKQRELLRPSFEMTRINKVFNLM
SEAVADMMDRFSKYPNHAVIEVDEAMTFITADVIFRTIMSSKLDEEKGKKILNAFVTFQE
QSVHTAMRRMFRFPKWLSYVLGDCKRAKAGDVIRQVLSDIIKPRYDMADNAEFEDILGSL
LLVVDADTNKRFSFEEILDQVAMLFLAGHETTASSLTWTLYLLSLYPKEQEKAYEEITQV
LQGGVIEISHLRQFKYLTNIFKESLRLYPPVGFFAREAKKDTQVRDKLIKKGSGVVIAPW
LIHRHEEFWTNPHGFNPSRFEGEYKKDAYLPFGVGERICIGQGFAMQEAILILANILKTY
KLELEEGFVPDVVGRLTVRSANGMRIKFSKRKL

>BN867_13910|KEGG|CYP172A|[Campylobacter jejuni 4031]cjx|EP

MSECPFFPKPYKNKASTLLTFLLKRRSWLDGLYERSYKMQTGYVKMPNFDLYVINDTKEV
KRMMVDEVREFPKSAFLHELLSPLLGESIFTTNGEVWKKQRELLRPSFEMTRINKVFNLM
SEAVADMMDRFSKYPNHAVIEVDEAMTFITADVIFRTIMSSKLDEGKGKKILNAFVTFQE
QSVHTAMRRMFRFPKWLSYVLGDRKRTKAGDVIRQVLSDIIKPRYDMADNAEFEDILGSL
LLVVDADTNKRFSFEEILDQVAMLFLAGHETTASSLTWTLYLLSLYPKEQEKAYEEITQV
LQGGAIEISHLRQFKYLTNIFKESLRLYPPVGFFAREAKKDTQVRDKLIKKGSGVVIAPW
LIHRHEEFWTNPHGFNPSRFEGEYKKDAYLPFGVGERICIGQGFAMQEAILILANILKTY
KLELEEGFVPDVVGRLTVRSANGMRIKFSKRKL

>CLA_0322|KEGG|CYP172A|[Campylobacter lari RM2100]cla|EP

MGQCPFHPKPYKNKASTLTTFLLKRRSWLDGLYERSYKMMMGRVKMPGFDLYVVNDPKEV
RRIMVDEVREYPKSQLLHELLEPLLGISIFTTNDRVWEKQRELLRPSFEMTRISKVFNLM
SEAASDMMARFAKYEDKAIVEVDEAMTFVTADVIFRTIMSSKLDEQKGKLVLDAFVTVQE
ETVKTAMRRMFRFPTWLSNLLGERKRLKAGGVIRKVLSDIIKPRYDNATNDQGKYEDILS
SLLMVVDADTNERFSFNEILDQVAMLFLAGHETTASSLTWTLYILSISPNEQQKAYEEIM
QVAGDEEFKIEHIRAMKYLTNVFKESLRLYPPVGFFAREARNDNKMRDKLIKKGSGVVVA
PWLIHRHDSFWENPHEFDPSRHEDKSKIKKDTYMPFGMGERVCIGQGFAMQETVLILANI
LRTYKLELEENFIPDIVGRLTIRSANGMNIRFIKRQK

>UPTC16701_0323|KEGG|CYP172A|[Campylobacter lari RM16701]clr|EP

MGQCPFHPKPYKNKASTLTTFLLKRRSWLDGLYERSYKMMMGRVKMPGFDLYVVNDPKEV
RRIMVDEVREYPKSQLLHELLEPLLGISIFTTNDRVWEKQRELLRPSFEMTRISKVFNLM
SEAASDMMARFAKYEDKAIVEVDEAMTFVTADVIFRTIMSSKLDEQKGKLVLDAFVTVQE
ETVKTAMRRMFRFPTWLSNLLGERKRLKAGTTIRKVLSDIIKPRYDNAINDQGKYEDILS
SLLMVVDADTNERFSFNEILDQVAMLFLAGHETTASSLTWTLYILSISPNEQQKAYEEIM
QVAGDEEFKIEHIRAMKYVTNVFKESLRLYPPVGFFAREARNDNKMRDKLIKKGSGVVVA
PWLIHRHDSFWENPHEFDPSRHEDKSKIKKDTYMPFGMGERVCIGQGFAMQEAVLILANI
LRTYKLELEENFVPDIVGRLTIRSANGMNIRFIKRQK

>UPTC16712_0326|KEGG|CYP172A|[Campylobacter lari RM16712]clm|EP

MGQCPFHPKPYKNKASTLTTFLLKRRSWLDGLYERSYKMMMGRVKMPGFDLYVVNDPKEV
RRIMVDEVREYPKSQLLHELLEPLLGISIFTTNDRVWEKQRELLRPSFEMTRISKVFNLM
SEAASDMMARFAKYEDKAIIEVDEAMTFVTADVIFRTIMSSKLDEQKGKLVLDAFVTVQE
ETVKTAMRRMFRFPTWLSNLLGERKRLKAGGVIRKVLSDIIKPRYDNALNDQGKYEDILS
SLLMVVDVDTNERFSFNEILDQVAMLFLAGHETTASSLTWTLYILSISPNEQQKAYEEIM
QVTGDGEFKIEHIRAMKYLTNVFKESLRLYPPVGFFAREARNESKMRDKLIKKGSGVVVA
PWLIHRHDSFWKNPHEFDPSRHEDKSKIKKDTYMPFGMGERVCIGQGFAMQEAVLILANI
LRTYKLELEENFVPDIVGRLTIRSANGMNIRFIKRQK

>UPTC3659_0339|KEGG|CYP172A|[Campylobacter lari NCTC 11845]cln|EP

MGQCPFHPKPYKNKASTLTTFLLKRRSWLDGLYERSYKMMMGRVKMPGFDLYVVNDPKEV
RRIMVDEVREYPKSQLLHELLEPLLGISIFTTNDRVWEKQRELLRPSFEMTRISKVFNLM
SEAASDMMARFAKYEDKAIIEVDEAMTFVTADVIFRTIMSSKLDEQKGKLVLDAFVTVQE
ETVKTAMRRMFRFPTWLSNLLGERKRLKAGGVIRKVLSDIIKPRYDNTTNDQGKYEDILS
SLLMVVDADTNERFSFNEILDQVAMLFLAGHETTASSLTWTLYILSISPDEQQKAYEEIM
QVAGDEEFKIEHIRAMKYLTNVFKESLRLYPPVGFFAREARNDNKMRDKLIKKGSGVVVA
PWLIHRHDSFWENPHEFDPSRHEDKSKIKKDTYMPFGMGERVCIGQGFAMQEAVLILANI
LRTYKLELEENFVPDIVGRLTIRSANGMNIRFIKRQK

>CONCH_0330|KEGG|CYP172A|[Campylobacter lari subsp. concheus LMG 11760]cll|EP

MGQCPFHPKPYKNKASTLTTFLLKRRSWLDGLYERSYKMMMGRVKMPGFDLYVVNDPKEV
RRIMVDEVREYPKSQLLHELLEPLLGISIFTTNDRVWEKQRELLRPSFEMTRISKVFNLM
SEAASDMMARFAKYEDKAIVEVDEAMTFVTADVIFRTIMSSKLDEQKGKLVLDAFVTVQE
ETVKTAMRRMFRFPTWLSNLLGERKRLKAGGVIRKVLSDIIKPRYDNATNDQGKYEDILS
SLLMVVDADTNERFSFNEILDQVAMLFLAGHETTASSLTWTLYILSISPNEQQKAYEEIM
QVAGNEEFKIEHIRAMKYLTNVFKESLRLYPPVGFFAREARNESKMRDKLIKKGSGVVVA
PWLIHRHDSFWENPHEFDPSRHEDKSKIKKDTYMPFGMGERVCIGQGFAMQEAVLILANI
LRTYKLELEENFVPDIVGRLTIRSANGMNIRFIKRQK

>BN865_07260|KEGG|CYP172A|[Campylobacter coli 76339]ccol|EP

MSQCPFFPKPYKNKASTLLTFLLKRRSWLDGLYERSYKMQTGYVKMPNFDLYVINDTKEV
KRMMVDEVKEFPKSAFLHELLSPLLGESIFTTNGEVWKKQRELLRPSFEMTRISKVFNLM
SEAVEDMMKRFEKYPNGSIIEVDEAMTFITADVIFRTIMSSKLDEEQGKKILDAFVTFQE
QSVHTAMRRMFRFPKWLSYVLGDRKRAKAGDVIRQVLSDIIKPRYDAVSSGKAQSFEDIL
GSLLLVVDAQTNQRFSFEEILDQVAMLFLAGHETTASSLTWTLYLLSLYPDEQEKAYKEI
IQVLQGENIQISHLRQFKYLTNIFKESLRLYPPVGFFAREAKKDTKIRDKMVKKGSGVVV
APWLIHRHESFWNNPHEFKPSRFESEYKKDAYLPFGAGERICIGQGFAMQEAILILASIL

RKYKLELEEGFVPDVVGRLTVRSANGMNIKFTKRES

>G157_01790|KEGG|CYP172A|[Campylobacter coli CVM N29710]ccc|EP

MSQCPFFPKPYKNKASTLLTFLLKRRSWLDGLYERSYKMQTGYVKMPNFDLYVINDTKEV
KRMMVDEVKEFPKSAFLHELLSPLLGESIFTTNGEVWKKQRELLRPSFEMTRISKVFNLM
SEAVSDMMKRFEKYPNASIIEVDEAMTFITADVIFRTIMSSKLDEEQGKKILDAFVTFQE
QSVHTAMRRMFRFPKWLSYVLGDRKRAKAGDVIRQALSDIIKPRYDAVSSGKAENFEDIL
GSLLLVVDAQTNQRFSFEEILDQVAMLFLAGHETTASSLTWTLYLLSLYPDEQEKAYKEI
IQVLQGENIQISHLKQFRYLTHIFKESLRLYPPVGFFAREAKKDTKVRDKMIKKGSGVVI
APWLIHRHEGFWANPHEFKPSRFEGEYKKDAYLPFGVGERICIGQGFAMQEAILILANIL
KKYKLELEEGFVPDVVGRLTIRSANGMRIKFSKREP

>N149_1372|KEGG|CYP172A|[Campylobacter coli 15-537360]ccq|EP

MSQCPFFPKPYKNKASTLLTFLLKRRSWLDGLYERSYKMQTGYVKMPNFDLYVINDTKEV
KRMMVDEVKEFPKSAFLHELLSPLLGESIFTTNGEVWKKQRELLRPSFEMTRISKVFNLM
SEAVSDMMKRFEKYPNASIIEVDEAMTFITADVIFRTIMSSKLDEEQGKKILDAFVTFQE
QSVHTAMRRMFRFPKWLSYVLGDRKRAKAGDVIRQALSDIIKPRYDAVSSGKAENFEDIL
GSLLLVVDAQTNQRFSFEEILDQVAMLFLAGHETTASSLTWTLYLLSLYPDEQEKAYKEI
IQVLQGENIQISHLKQFRYLTHIFKESLRLYPPVGFFAREAKKDTKVRDKMIKKGSGVVI
APWLIHRHEGFWANPHEFKPSRFEGEYKKDAYLPFGVGERICIGQGFAMQEAILILANIL
KKYKLELEEGFVPDVVGRLTIRSANGMRIKFSKREP

>YSQ_01815|KEGG|CYP172A|[Campylobacter coli RM1875]ccf|EP

MSQCPFFPKPYKNKASTLLTFLLKRRSWLDGLYERSYKMQTGYVKMPNFDLYVINDTKEV
KRMMVDEVKEFPKSAFLHELLSPLLGESIFTTNGEVWKKQRELLRPSFEMTRISKVFNLM
SEAVSDMMKRFEKYPNASIIEVDEAMTFITADVIFRTIMSSKLDEEQGKKILDAFVTFQE
QSVHTAMRRMFRFPKWLSYVLGDRKRAKAGDVIRQVLSDIIKPRYDAVNSEKAQSFEDIL
GSLLLVVDAQTNQRFSFEEILDQVAMLFLAGHETTASSLTWTLYLLSLYPDEQEKAYKEI

IQVLQGENIQISHLRQFKYLTNIFKESLRLYPPVGFFAREAKKDTKIRDKMIKKGSGVVI
APWLIHRHEGFWANPHEFKPSRFESEYKKDAYLPFGVGERICIGQGFAMQEAILILANIL
KKYKLELEEGFVPDVVGRLTVRSANGMRIKFSKREP

>YSS_07600|KEGG|CYP172A|[Campylobacter coli RM4661]ccy|EP

MSQCPFFPKPYKNKASTLLTFLLKRRSWLDGLYERSYKMQTGYVKMPNFDLYVINDTKEV
KRMMVDEVKEFPKSAFLHELLSPLLGESIFTTNGEVWKKQRELLRPSFEMTRISKVFNLM
SEAVSDMMKRFEKYPNASIIEVDETMTFITADVIFRTIMSSKLDEEQGKKILDAFVTFQE
QSVHTAMRRMFRFPKWLSYVLGDRKRAKAGDVIRQALSDIIKPRYDAVSSGKAENFEDIL
GSLLLVVDAQTNQRFSFEEILDQVAMLFLAGHETTASSLTWTLYLLSLYPDEQEKAYKEI
IQVLQGENIQISHLKQFRYLTHIFKESLRLYPPVGFFAREAKKDTKVRDKMIKKGSGVVI
APWLIHRHEGFWANPHEFKPSRFEGEYKKDAYLPFGVGERICIGQGFAMQEAILILANIL
KKYKLELEEGFVPDVVGRLTIRSANGMRIKFSKRES

>YSU_01845|KEGG|CYP172A|[Campylobacter coli RM5611]ccoi|EP

MSQCPFFPKPYKNKASTLLTFLLKRRSWLDGLYERSYKMQTGYVKMPNFDLYVINDTKEV
KRMMVDEVKEFPKSAFLHELLSPLLGESIFTTNGEVWKKQRELLRPSFEMTRISKVFNLM
SEAVSDMMKRFEKYPNASIIEVDEAMTFITADVIFRTIMSSKLDEEQGKKILDAFVTFQE
QSVHTAMRRMFRFPKWLSYVLGDRKRAKAGDVIRQALSDIIKPRYDAVSSGKAENFEDIL
GSLLLVVDAQTNQRFSFEEILDQVAMLFLAGHETTASSLTWTLYLLSLYPDEQEKAYKEI
IQVLQGENIQISHLKQFRYLTHIFKESLRLYPPVGFFAREAKKDTKVRDKMIKKGSGVVI
APWLIHRHEGFWANPHEFKPSRFEGEYKKDAYLPFGVGERICIGQGFAMQEAILILANIL
KKYKLELEEGFVPDVVGRLTIRSANGMRIKFSKRES

>VC76_07055|KEGG|CYP172A|[Campylobacter coli FB1]ccof|EP

MSQCPFFPKPYKNKASTLLTFLLKRRSWLDGLYERSYKMQTGYVKMPNFDLYVINDTKEV
KRMMVDEVKEFPKSAFLHELLSPLLGESIFTTNGEVWKKQRELLRPSFEMTRISKVFNLM
SEAVSDMMKRFEKYPNASIIEVDEAMTFITADVIFRTIMSSKLDEEQGKKILDAFVTFQE

QSVHTAMRRMFRFPKWLSYVLGDRKRAKAGDVIRQALSDIIKPRYDAVSSGKAENFEDIL
GSLLLVVDAQTNQRFSFEEILDQVAMLFLAGHETTASSLTWTLYLLSLYPKEQEKAYEEI
TQVLQGGVIEISHLRQFKYLTNIFKESLRLYPPVGFFAREAKKDTKVRDKMIKKGSGVVI
APWLIHRHEGFWANPHEFKPSRFEGEYKKDAYLPFGVGERICIGQGFAMQEAILILANIL
KKYKLELEEGFVPDVVGRLTIRSANGMRIKFSKREP

>ATE51_00744|KEGG|CYP172A|[Campylobacter coli OR12]ccoo|EP

MSQCPFFPKPYKNKASTLLTFLLKRRSWLDGLYERSYKMQTGYVKMPNFDLYVINDTKEV
KRMMVDEVKEFPKSAFLHELLSPLLGESIFTTNGEVWKKQRELLRPSFEMTRISKVFNLM
SEAVSDMMKRFEKYPNASIIEVDEAMTFITADVIFRTIMSSKLDEEQGKKILDAFVTFQE
QSVHTAMRRMFRFPKWLSYVLGDRKRAKAGDVIRQALSDIIKPRYDAVSSGKAENFEDIL
GSLLLVVDAQTNQRFSFEEILDQVAMLFLAGHETTASSLTWTLYLLSLYPKEQEKAYEEI
TQVLQGGVIEISHLRQFKYLTNIFKESLRLYPPVGFFAREAKKDTKVRDKMIKKGSGVVI
APWLIHRHEGFWANPHEFKPSRFEGEYKKDAYLPFGVGERICIGQGFAMQEAILILANIL
KKYKLELEEGFVPDVVGRLTIRSANGMRIKFSKREP

>CINS_0316|KEGG|CYP172A|[Campylobacter insulaenigrae]cis|EP

MGQCPFYPKPYKNKASTLATFLFKRRSWLDGLYERSYKMMMGRVKMPGFDLYVVNDPKEV
RRIMVDEVREYPKSQLLHELLEPLLGVSIFTTNDRVWEKQRELLRPSFEMTRISKVFDLM
SQAANDMMIRFAKCDDGKIIEVDEAMTFVTADVIFRTIMSSKLDEQKGKIVLDAFVTVQE
ETIKTAMRKMFRFPNWLSNFLGEKKRLKAGSTIRKVLSDIIKPRYDNALADQGKYKDILS
SLLMVVDEDTNERFSFNEILDQVAMLFLAGHETTASSLTWTLYILSISQNEQQKAYEEII
QVANFNDFKIEHIKEMKYLTNVFKESLRLYPPVGFFAREARHESKMRDKLIQEGSGVVVA
PWLIHRHDNFWENPHEFDPTRYEDKGKIKKDTYMPFGMGERVCIGQGFAMQEAILILANI
LRTYKLELEENFIPDIVGRLTIRSANGMRIKFIKRKK

>CVOL_0316|KEGG|CYP172A|[Campylobacter volucris]cvo|EP

MGQCPFHPKPYKNKASTLMTFLLKRRSWLDGLYERSYKMMMGRVQMPGFDLYVVNDPKEV

RRIMVDEVREYPKSQLLHELLEPLLGVSIFTTNGRVWEKQRELLRPSFEMTRISKVFGLM
SEAASDMMARFDKYEDGAIIEVDEAMTFVTADVIFRTIMSSKLDEVKGKIVLDAFVTVQE
QTVKTAMRRMFRFPTWLSNLLGEKKRLKAGMTIRKVLSDIIKPRYENALADKGKYEDILS
SLLMVVDADTNERFSFNEILDQVAMLFLAGHETTASSLTWTLYILSISEKEQEKAYEEIM
QVAGDEKFTIEHIKSMKYLTNIFKESLRLYPPVGFFAREAKHESKMRDKLIKEGSGVVVA
PWLIHRHDNFWENPHEFDPSRHEDKSKIKKDTYMPFGMGERICIGQGFAMQEAILILANI
LRTYKLQLEENFTPDVVGRLTIRSANGMNIKFIKRKK

>CPEL_0328|KEGG|CYP172A|[Campylobacter peloridis]cpel|EP

MGQCPFHPKPYKNKASTLTTFLLKRRSWLDGLYDRSYKMMMGRVKMPGFDLYVVNDPKEV
RRIMIDEVREYPKSQLLHELLEPLLGVSIFTTNDRVWEKQRELLRPSFEMTRISKVFNLM
SEAAADMMARFAKYEDKAIIEVDEAMTFVTADVIFRTIMSSKLDEQKGKLVLDAFVTVQE
ETVKTAMRRMFRFPTWLSNLLGERKRLKAGGVIRKVLSDIIKPRYDNAINDQGKYEDILS
SLLMVVDADTNERFSFNEILDQVAMLFLAGHETTASSLTWTLYILSISPKEQQKAYEEIM
QIAGNDEFKIEHIKAMKYLTNVFKESLRLYPPVGFFAREARNESKMRDKLIKKGSGVVVA
PWLIHRHDNFWENPHEFDPTRHEDKSKIKKDTYMPFGMGERVCIGQGFAMQEAILILANI
LRTYKLELQENFVPDIVGRLTIRSANGMNIRFIKRQK

>CAQ16704_0328|KEGG|CYP172A|[Campylobacter sp. RM16704]camr|EP

MGQCPFHPKPYKNKASTLTTFLLKRRSWLDGLYERSYKMMMGRVKMPGFDLYVVNDPKEV
RRIMVDEVREYPKSQLLHELLEPLLGISIFTTNDRVWEKQRELLRPSFEMTRISKVFDLM
SDAASDMMARFNKYEDGATIEVDEAMTFVTADVIFRTIMSSKLDEQKGKIVLDAFVTVQE
QTVKTAMRRMFRFPTWLSNLLGEKKRLKAGTTIRKVLSDIIKPRYDNALNDQGKYEDILS
SLLMVVDADTNERFSFNEILDQVAMLFLAGHETTASSLTWTLYILSMSPNEQQKAYEEIM
QIAGKEEFKIEHIKAMKYLTNIFKESLRLYPPVGFFAREARNESKMRDKLIKKGSGVVVA
PWLIHRHDNFWENPHEFDPSRHDDKSKIKKDTYMPFGMGERVCIGQGFAMQEAILILANI
LRTYKLELEENFVPDIVGRLTIRSANGMNIRFIKR

>CSUB8521_0354|KEGG|CYP172A|[Campylobacter subantarcticus LMG 24374]csm|EP

MGQCPFHPKPYKNKASTLTTFLLKRRSWLDGLYERSYKMMMGRVKMPGFDLYVVNDPKEV
RRIMVDEVREYPKSQLLHELLEPLLGISIFTTNDRVWEKQRELLRPSFEMTRISKVFNLM
NEAASDMMARFAKHENGAVVEVDEAMTFVTADVIFRTIMSSKLDEQKGKLVLDAFVTVQE
ETVKTAMRRMFRFPTWLSNLLGERKRLKAGATIRQVLSDIIKPRYDNALNDQGKYEDILS
SLLAVVDADTNERFSFNEILDQVAMLFLAGHETTASSLTWTLYILSISPKEQQKAYEEIM
QVAGNEEFKIEHIRAMKYVANVFKESLRLYPPVGFFAREAKNESKMRDKLIKKGSGVVVA
PWLIHRHDSFWENPHEFDPSRHEGKSKIKKDTYMPFGMGERVCIGQGFAMQEAVLILANI
LRTYRLELEENFVPDVVGRLTIRSANGMNIRFIKREK

>CSUB8523_0336|KEGG|CYP172A|[Campylobacter subantarcticus LMG 24377]csf|EP

MGQCPFHPKPYKNKASTLTTFLLKRRSWLDGLYERSYKMMMGRVKMPGFDLYVVNDPKEV
RRIMVDEVREYPKSQLLHELLEPLLGISIFTTNDRVWEKQRELLRPSFEMTRISKVFNLM
NEAASDMMARFAKHENGAVVEVDEAMTFVTADVIFRTIMSSKLDEQKGKLVLDAFVTVQE
ETVKTAMRRMFRFPTWLSNLLGERKRLKAGATIRQVLSDIIKPRYDNALNDQGKYEDILS
SLLIVVDADTNERFSFNEILDQVAMLFLAGHETTASSLTWTLYILSISPKEQQKAYEEIM
QVAGNEEFKIEHIRAMKYVANVFKESLRLYPPVGFFAREAKNESKMRDKLIKKGSGVVVA
PWLIHRHDSFWENPHEFDPSRHEGKSKIKKDTYMPFGMGERVCIGQGFAMQEAVLILANI
LRTYRLELEENFVPDVVGRLTIRSANGMNIRFIKREK

>CHELV3228_0153|KEGG|CYP172A|[Campylobacter helveticus]chv|EP

MSGCPFFPKPYKNKASTLLTFLLKRRSWLDGLYERSYKMQTGYVKMPNFDLYVINDTKEV
KRMMVDEVREFPKSELLHRLLSPLLGESIFTTNGEVWHRQRELLKPSFEMTRISKVFNLM
SEAVADLMARFEKYPNHSYIEVDEMMTFVTADVIFRTIMSSKLDKNEGRKILEAFVTFQE
ESVRTAMRGMFYFPKWLSYLLGDRKRAKAGEVIRRALSDIIKPRYDEANLEKSSYEDILA
SLLLVVDAKTNERFSFEEILDQVAMLFLAGHETTASSLTWTLYLLSLYPKEQEKAFKEIC
EVLQDSEEIKITHLRQFKFLTNVFKEALRLYPPVGFFAREAKKDTQVRDKLVKKGSGVVI
APWLIHRHELFWQNPHGFDPSRFEREYKKDAYLPFGMGERICIGQGFAMQEAILILANIL
RIYKLGLKENFVPDVVGRLTIRSANGMWIKFSKR

>CCUN_1453|KEGG|CYP172A|[Campylobacter cuniculorum]ccun|EP

MSQCPFHPKPYKNKASTLLTFLLKRRSWLDGLYERSYEMQTGYVKMPNFDLYVINDPKEV
KRIMVDEVREFPKSAFLHELLKPLLGESIFTTNGEIWSKQRELLRPSFEMTRISKVFNLM
SEAVKDMMKRFEKYPNDSIIEVDEMMTFVTADVIFRTIMSSKLDEQKGKKILHAFVNFQE
QSVHTAMRKMFRFPKWFSYILGDRKRLKSGEIIREILSDIIKPRYNAVSEGKNLGNEDIL
ESLLLVVDAKTNQRFSFEEILDQVAMLFLAGHETTASSLTWTLYLLSLYPQEQEKAYEEV
RQILNGEEIQISHLRQFKFLTNVFKESLRLYPPVGFFAREAKKDTKIRDKNIKKGSGVVV
APWLIHRHKHFWDNPNDFNPSRFDKEYRKDAYLPFGMGERICIGQGFAMQEAILILASIL
KIYKLELESGFVPDVVGRLTIRSANGMNIKFSKR

>CLAN_0140|KEGG|CYP172A|[Campylobacter lanienae]clx|EP

MGVCPFHPKPHSSKAGLITTFFLKRRSWLDGLYEKSYKMRVGRVKMPGFDLFVVNDPKEV
RKIMVDNVKEFPKSDMLHELLKPLLGVSIFTTNGEVWKKQRELLRPSFEMTRISKVFDLM
SSAAADMMERFRKYEDGAVVEVDEHMTFVTADVIFRTIMSAKLDEEKGKEILDAFVTFQE
ETAKTAIRKFFCVPQWLSNLLGENKRIKAGAVIRKNLSNIIKPRYDNYANDTHCDILSSL
LHTTDADSGERFSFEEILDQVSMLFLAGHETTASSLTWTLYCLSLDQDAQQKAYNEIISI
NKDSKFEISDIRDMKYLTNVFKEALRLYPPVGFFARQNKNEIKIRDKVLQKGAGVVVAPW
LIHRHDDYWEAPHEFRPERHNENIPKERYMPFGLGERVCIGHGFAMQEAIIILANILREF
KLELKDGFIPDVVGRLTIRSANGMMIKMTQRH

>CAV_1397|KEGG|CYP172A|[Campylobacter avium]cavi|EP

MACPFHPKPKKTKASTLMTFLLKRRSWLDGLYEKSYSMQSGRVKMPGFDLYVVNDVREVK
RIMVDEVKEFPKSELLHRLLEPLLGVSIFTTNGDVWKKQRELLMPSFEMTRINKVFELMK
DAANDLLIRLEKKDKDFIDADEEMTFVTADVIFRTIMSAKLDEEKGKEILKAFSIMQEET
IRTGIREMFCFPKWLSKLLGENKRMKAGACIRDNLAQIIKPRYDEQREGKADYRQDILAS
LLRVIDADTNEPFSFKEILDQVSMLFLAGHETTASSLTWTLYCLCVEPSWQEKAYDEIMQ

ITKGEELSIAHVKAFKVLNNIFKEALRLYPPVGFFPRTAKKDTKIRDKEIKAGSGVVVAP
WLIHRHSKLWQDPHEFKPQRFDEQINKDAYMPFGMGERICIGQGFAMQEAMIILASILKK
YKLQLKENFTPDVVGRLTVRSANGMSIKFIRR

>A2J15_004785|KEGG|CYP172A|[Campylobacter hepaticus]chw|EP

MNQCPFFPKPYKNKASTLLTFLLKRRSWLDGLYERSYKMQTGYVKMPNFDLYVVNNTKEV
KRIMVNEVRQFPKSAFLHELLSPLLGESIFTTNGDVWKKQRELLRPSFEMTGINKVFNLM
KDAAADMMQRFDKYPNNAIIEVDEAMTFVTADVIFRTIMSSKLDEEQGKKILNAFVTFQE
QTVHTAMRRMFHFPKWFSYILGDRKRAKAGELIRQVLKDIVKPRYDKANEANQEEFKDIL
SSLLLVVDAQTNKRFSFEEILDQVAMLFLAGHETTASSLTWTLYLLSLYPKDQEKAYEEL
CEILQNKDIEIVHLKQFKYLINIFKESLRLYPPVGFFAREAKQDTQIRDKFIKKGSGVVI
APWLIHRHEKFWSDPNGFNPSRFEHEYQKDAYLPFGVGERICIGQGFAMQEAILILANIL
RVYKLELEEGFVPDVVGRLTVRSANGMRIKFTKRKA

>CVIC12175_1523|KEGG|CYP172A|[Campylobacter sp. RM12175]camz|EP

MGVCPFHPKPHNSKAGLITTFLLKRRSWLDGLYAKSYKMRAGRVKMPGFDLFVVNDPKEV
RKIMIDNVKEFPKSDMLHELLKPLLGVSIFTTNGEVWKKQRELLKPSFEMTRISKVFDLM
SSAAADMMERFRKYPDGSIVEVDEHMTFVTADVIFRTIMSSKLDEIKGKEILHAFVTFQE
ETARTAIRKFFCVPQWLSNILGENKRMKAGAVIRKSLSDIIQPRYDSYKSDNYSDILSSL
LHTVDASSGERFSFEEILDQVSMLFLAGHETTASSLTWTLYCLSLDQDAQQKAYNEIISI
NKDGKFSISDIKEMKYLTNVFKEALRLYPPVGFFARQSKGEAKIRDKVLQKGSGVVVAPW
LIHRHDDFWEAPHEFRPDRHNENIPKEKYMPFGLGERICIGQGFAMQEAIIILANILREF
KLELKDGFVPDVVGRLTVRSANGMMIKMSKRQ

>CSUIS_0153|KEGG|CYP172A|[Campylobacter sp. RM6137]camy|EP

MGVCPFHPKPHNNKAGLITTFLLKRRSWLDGLYVKSYKMRSGRVKMPGFDLFVVNDPNEV
RKIMIDNVKEFPKSDLLHELLKPLLGVSIFTTNGEVWKKQRELLRPSFEMTRISKVFDLM
SSAAADMMDRFRKYEDGSIIEVDEHMTFVTADVIFRTIMSAKLDEEKGKEILHAFVTFQE

ETARTAIRKFFCVPQWLSNILGENKRMKAGEIIRQNLSNIIKPRYDNYANDTHCDILSSL
LHTIDADSNQRFSFEEILDQVSMLFLAGHETTASSLTWTLYCLSLDQDAQQKAYNEVISI
NKDGKFSISDIKNMRYLTNVFKEALRLYPPVGFFARQSKNDTKIRDKVLKKGSGVVVAPW
LIHRHEDFWDAPHEFRPDRHNDDIPKEKYMPFGLGERICIGQGFAMQEAIIILANILREF
KLELKDGFIPDVVGRLTVRSANGMMIKMTRRQ

>CORN_0326|KEGG|CYP172A|[Campylobacter ornithocola]coj|EP

MGQCPFHPKPYKNKASTLTTFLLKRRSWLDGLYERSYKMMMGRVKMPGFDLYVVNDPKEV
RRIMVDEVREYPKSQLLHELLEPLLGVSIFTTNDKVWEKQRELLKPSFEMTRISKVFDLM
SEAASDMMGRFAKYENKAVIEVDEAMTFVTADVIFRTIMSSKLDEQKGKLVLDAFVTVQE
ETVKTAMRRMFRFPTWLSNLLGEKKRLKAGGVIRKVLSDIIKPRYDNALNDQGKYEDILS
SLLMVVDADTNKRFSFNEILDQVAMLFLAGHETTASSLTWTLYILSISPNEQQKAYEEII
QVAGNGEFKIEHIRAMKYVTNVFKESLRLYPPVGFFAREARNESKMRDKLIKKGSGVVVA
PWLIHRHDNFWENPHEFDPSRHEDKGKIKKDTYMPFGMGERICIGQGFAMQEAVLILANI
LRTYKLELEENFVPDIVGRLTIRSANGMNIRFIKRQK

>CUP3940_1510|KEGG|CYP172A|[Campylobacter upsaliensis]cux|EP

MSECPFFPKPYKNKASTLLTFLLKRRSWLDGLYERSYQMQTGYVKMPNFDLYVINDTKEV
KRMMVDEVREFPKSELLHRLLSPLLGESIFTTNGEVWHKQRELLKPSFELTRISKVFNLM
SEAVADLMRRFEKYPDNSYIEVDEMMTFVTADVIFRTIMSSKLDEVQGRQILEAFATFQE
ESVRTAMRGMFYFPKWLSYLLGDRKRAKAGELIRKALSDIIKPRYEANLEKSDYEDILSS
LLLVVDAKTNQRFSFEEILDQVAMLFLAGHETTASSLTWTLYLLSLYPEEQERAYNEICE
ILQDSKEIKIAHLRQFKFLTNVFKESLRLYPPVGFFAREAKKDTQVRDKLIKQGSGVVIA
PWLIHRHELFWQNPHGFDPSRFEREYKKEAYLPFGMGERICIGQGFAMQEAILILANILK
TYRLELKEDFVPDVVGRLTIRSLNGMWIKFSKR

>CARM_0293|KEGG|CYP172A|[Campylobacter armoricus]carm|EP

MGQCPFHPKPYKNKASTLTTFLFKRRSWLDGLYERSYKMMMGRVQMPGFDLYVVNDPKEV

RRIMVDEVREYPKSQLLHELLEPLLGVSIFTTNGRVWEKQRELLRPSFEMTRISKVFGLM
SEAASDMMARFAKYEDKAIIEVDEAMTFVTADVIFRTIMSSKLDEQKGKIVLDAFVTVQE
QTVKTAMRRMFRFPTWLSNLLGERKRLKAGGVIRKVLSDIIKPRYENALNDQGKYEDILS
SLLMVVDADTNERFSFDEILDQVAMLFLAGHETTASSLTWTLYILSMSPNEQQKAYEEIM
QVAGDEKFTIEHIKSMKYLTNIFKESLRLYPPVGFFAREARNESKMRDKLIKKGSGVVVA
PWLIHRHDSFWENPHEFDPSRHEDKSKIKKDTYMPFGMGERICIGQGFAMQEAILILANI
LRTYKLELEENFVPDIVGRLTIRSANGMNIRFIKR

>CNZW441b_1292|KEGG|CYP172A|[Campylobacter novaezeelandiae]cnv|EP

MSQCPFFPKPYKTKASTLLTFLLKRRSWLDGLYERSYKMQTGYVKMPNFDLYVINDTKEV
KRIMIDEVKEFPKSEFLHQLLSPLLGESIFTTNGEVWRKQRELLRPSFEMTRINKVFNLM
SEAVADMMSRFEKYHDESIIEVDEAMTFVTADVIFRTIMSSKLDEKKGKKVLDAFVTFQE
QSVHSAMRRMFRFPKWISYILGDKKRLQAGETIREVLSEIIRPRYESFDPQNTNEFDILE
SLLCVIDANTNKRFSFEEILDQVAMLFLAGHETTASSLTWTLYLLSSFEDEQEKAYREVE
EVLQGKDIEITHLKQFKILTNIFKESLRLYPPVGFFAREAKQDTKIRDKFVKKGSGVVVA
PWLIQRHENYWEKPNEFNPSRFEGDYKKDAYLPFGMGERICIGQAFAMQEAILILANILR
KYKLQLQEGFIPDVVGRLTVRSANGMNIKFIKRKK

>AMYT_2277|KEGG|CYP2724A1|[Helicobacter winghamensis]amyt|EP
MKSLIPPQLKFQPLKNIKATSALRAMLYAEEFWPEEILNKPSVSLKTTTGELLFLSDPFL
VKEVLTRNDGTIPRSRLQQRFAGHGTGRENVITDIGKRSAMHRKTLAPLFSPDRFNTYFP
FIKMTLEEAIKPIFDAIEKKEPIDIGRVCVNGTFGVIWQILFGEDKKLTPPLIVEQMANT
LYEAGLSGELTQTSKAVKQAKEISLSLRPLVPLVSDTPFAKNPKINLQLTNKELEDNAQF
LLTSGHESTALTITWALFLLAHYQDEQELIANEIKNILGNKELTLDILHKLPRLNNLFNE
TMRLYPSAILVNRETIQDVTVGELSIKKGTQIAVCFYSMHRHKNYWKEPDSFNPDRFNTL
NKEQLRAFMPFSTGHHSCLGGKLAWLEAMTILTTILKDVKILSCKNSIKPIARYTLRPDG
PVMLEVQKRN

Table S4. Identification of P450s that are part of the secondary metabolite biosynthetic gene clusters (sm-BGCs) in alphaproteobacterial species. Reference cluster information was obtained by performing BLAST at the anti-SMASH database [3], as indicated in the materials and methods section. The cluster type and most similar known cluster names available in the anti-SMASH database[3], were listed in the table.

Betaproteobacteria

**Species name**	**Species code**	**No of P450s part of cluster**	**Region**	**Type**	**Most similar known cluster**	**Similarit y**	**P450**
Chromobacterium phragmitis IIBBL 112-1	chri	1	Region 4	transAT-PKS,NRPS,NRPS-like	sorangicin A	8%	CYP261D1
Aquitalea magnusonii	amah	1	Region 3	butyrolactone			CYP1200A5
Ralstonia syzygii subsp. *celebesensis*	rsg	1	Region 5	transAT-PKS,NRPS-like,siderophore	rhizoxin A	100%	CYP1464A2
Cupriavidus basilensis	cbw	1	Region 2	NRPS-like	iturin	22%	CYP107DG5
Burkholderia mallei ATCC 23344	bma 2	2	Region 1	RiPP-like			CYP1488A1
			Region 1 1	hglE-KS,terpene	lipopolysaccharide	16%	CYP1013A3
Burkholderia mallei SAVP1	bmv 2	2	Region 6	hglE-KS,terpene	myxochromide D	20%	CYP1013A3
			Region 7	RiPP-like			CYP1488A1
Burkholderia mallei NCTC 10229	bml 2	2	Region 7	hglE-KS,terpene	myxochromide D	20%	CYP1013A3

			Region 8	RiPP-like			CYP1488A1
Burkholderia mallei NCTC 10247	bmn 2	2	Region 1	RiPP-like			CYP1488A1
			Region 1 1	hglE-KS,terpene	myxochromide D	20%	CYP1013A3
Burkholderia mallei 23344	bmal 2	2	Region 2	RiPP-like			CYP1488A1
			Region 3	terpene,hglE-KS	myxochromide D	20%	CYP1013A3
Burkholderia mallei 6	bmae 2	1	Region 7	RiPP-like			CYP1488A1
Burkholderia mallei BMQ	bmaq 2	2	Region 6	RiPP-like			CYP1488A1
			Region 7	terpene,hglE-KS	myxochromide D	20%	CYP1013A3
Burkholderia mallei 2000031063	bmai 2	2	Region 1	RiPP-like			CYP1488A1
			Region 1 1	hglE-KS,terpene	myxochromide D	20%	CYP1013A3
Burkholderia mallei FMH 23344	bmaf 2	2	Region 5	hglE-KS,terpene	N-myristoyl-D-asparagine / cis-7-tetradecenoyl-D-asparagine / (R)-N1-((S)-5-oxohexan-2-yl)-2-tetradecanamidosuccinamide	8%	CYP1013A3
			Region 6	RiPP-like			CYP1488A1
Burkholderia mallei NCTC 10247	bmaz 2	2	Region 1	NRPS	glidopeptin	62%	CYP1488A1
			Region 1 1	hserlactone	L-2-amino-4-methoxy-trans-3-butenoic acid	20%	CYP1013A3
Burkholderia mallei 2002734299	bmab 2	2	Region 8	RiPP-like			CYP1488A1
			Region 9	terpene,hglE-KS	myxochromide D	20%	CYP1013A3
Burkholderia pseudomallei 1710b	bpm 2	2	Region 1 0	hglE-KS,terpene	myxochromide D	20%	CYP1013A3
			Region 1 1	RiPP-like			CYP1488A1

Burkholderia pseudomallei 1106a	bpl 2	2	Region 1	RiPP-like			CYP1488A1
			Region 17	hglE-KS,terpene	myxochromide D	20%	CYP1013A3
Burkholderia pseudomallei 668	bpd 2	2	Region 1	RiPP-like			CYP1488A1
			Region 17	hglE-KS,terpene	myxochromide D	20%	CYP1013A3
Burkholderia pseudomallei MSHR305	bpse 1	2	Region 13	hglE-KS,terpene	myxochromide D	20%	CYP1013A3
			Region 14	RiPP-like			CYP1488A1
Burkholderia pseudomallei MSHR511	bpsm 2	2	Region 2	RiPP-like			CYP1488A1
			Region 3	terpene,hglE-KS	myxochromide D	20%	CYP1013A3
Burkholderia pseudomallei MSHR146	bpsu 2	2	Region 14	hglE-KS,terpene	myxochromide D	20%	CYP1013A3
			Region 15	RiPP-like			CYP1488A1
Burkholderia pseudomallei MSHR520	bpsd 2	2	Region 7	hglE-KS,terpene	myxochromide D	20%	CYP1013A3
			Region 8	RiPP-like			CYP1488A1
Burkholderia pseudomallei 1026b	bpz 2	2	Region 1	RiPP-like			CYP1488A1
			Region 15	hglE-KS,terpene	myxochromide D	20%	CYP1013A3
Burkholderia pseudomallei BPC006	bpq 2	2	Region 1	RiPP-like			CYP1488A1
			Region 17	hglE-KS,terpene	myxochromide D	20%	CYP1013A3
Burkholderia pseudomallei NCTC 13179	bpk 2	2	Region 8	hglE-KS,terpene	myxochromide D	20%	CYP1013A3
			Region 9	RiPP-like			CYP1488A1

Burkholderia pseudomallei HBPUB10134a	bpsh 2	2	Region 1 4	RiPP-like				CYP1488A1
			Region 1 5	terpene,hglE-KS		myxochromide D	20%	CYP1013A3
Burkholderia pseudomallei NAU35A-3	bpsa 2	2	Region 2	RiPP-like				CYP1488A1
			Region 3	terpene,hglE-KS		myxochromide D	20%	CYP1013A3
Burkholderia pseudomallei A79A	bpso 2	2	Region 1 2	RiPP-like				CYP1488A1
			Region 1 3	terpene,hglE-KS		myxochromide D	20%	CYP1013A3
Burkholderia pseudomallei TSV202	but 2	2	Region 7	RiPP-like				CYP1488A1
			Region 8	terpene,hglE-KS		myxochromide D	20%	CYP1013A3
Burkholderia thailandensis E264	bte 2	2	Region 1	Ripp-Like				CYP1488A2
			Region 1 4	hglE-KS,terpene		myxochromide D	20%	CYP1013A3
Burkholderia thailandensis 2002721723	btq 2	2	Region 2	RiPP-like				CYP1488A2
			Region 1 5	hglE-KS		myxochromide D	20%	CYP1013A3
Burkholderia thailandensis E444	btj 2	2	Region 7	hglE-KS,terpene		myxochromide D	20%	CYP1013A3
			Region 8	RiPP-like				CYP1488A2
Burkholderia thailandensis H0587	btz 2	1	Region 1 3	RiPP-like				CYP1488A2
Burkholderia thailandensis MSMB121	btd 2	2	Region 2	transAT-PKS,PKS-like,NRPS,NRPS-like		thailanstatin A	100%	CYP1486A1
			Region 7	RiPP-like				CYP1488A2
Burkholderia thailandensis MSMB59	btv 2	2	Region 7	RiPP-like				CYP1488A2
			Region 8	terpene,hglE-KS		myxochromide D	20%	CYP1013A3

Burkholderia thailandensis E254	bthe 2	2	Region 10	hglE-KS,terpene	myxochromide D	20%	CYP1013A3
			Region 11	RiPP-like			CYP1488A2
Burkholderia thailandensis USAMRU Malaysia #20	bthm 2	2	Region 10	hglE-KS,terpene	myxochromide D	20%	CYP1013A3
			Region 11	RiPP-like			CYP1488A2
Burkholderia thailandensis 2003015869	btha 2	2	Region 13	hglE-KS,terpene	myxochromide D	20%	CYP1013A3
			Region 14	RiPP-like			CYP1488A2
Burkholderia thailandensis 2002721643	bthl 2	2	Region 11	hglE-KS,terpene	myxochromide D	20%	CYP1013A3
			Region 12	RiPP-like			CYP1488A2
Burkholderia oklahomensis C6786	boc 2	1	Region 5	hglE-KS,terpene	myxochromide D	20%	CYP1013A3
Burkholderia mayonis	buu 2	2	Region 9	terpene	sodorifen	50%	CYP117C2
			Region 17	hglE-KS,terpene	myxochromide D	20%	CYP1013A3
Burkholderia multivorans ATCC 17616 (Tohoku)	bmj 1	1	Region 2	hglE-KS	myxochromide D	20%	CYP1013A4
Burkholderia multivorans ATCC 17616 (Tohoku)	bmj 3	2	Region 2	redox-cofactor			CYP116B113
			Region 3	terpene	sodorifen	75%	CYP117C1
Burkholderia multivorans ATCC 17616 (JGI)	bmu 1	1	Region 3	hglE-KS	myxochromide D	20%	CYP1013A4
Burkholderia multivorans ATCC 17616 (JGI)	bmu 3	2	Region 2	terpene	sodorifen	75%	CYP117C1
			Region 3	redox-cofactor			CYP116B113

Burkholderia multivorans DDS 15A-1	bmk 1	1	Region 2	hglE-KS	myxochromide D	20%	CYP1013A4
Burkholderia multivorans DDS 15A-1	bmk 3	2	Region 2	terpene	sodorifen	75%	CYP117C1
			Region 3	redox-cofactor			CYP116B113
Burkholderia multivorans ATCC BAA-247	bmul 3	1	Region 2	redox-cofactor	lankacidin C	13%	CYP116B113
Burkholderia gladioli BSR3	bgd 1	1	Region 3	transAT-PKS,T3PKS,PKS-like	isobongkrekic acid / bongkrekic acid	85%	CYP267C1
Burkholderia gladioli ATCC 10248	bgo 2	1	Region 12	terpene	desotamide	9%	CYP183AG1
Burkholderia sp. 2002721687	bul 2	3	Region 1	transAT-PKS,PKS-like,NRPS,NRPS-like	thailanstatin A	100%	CYP1486A1
			Region 5	RiPP-like			CYP1488A2
			Region 6	terpene,hglE-KS	myxochromide D	20%	CYP1013A3
Burkholderia plantarii PG1	bgp 2	1	Region 11	blactam			CYP107DN1
Burkholderia plantarii ATCC 43733	bpla 1	1	Region 9	CDPS	bicyclomycin	75%	CYP1481A2
Burkholderia plantarii ATCC 43733	bpla 2	1	Region 12	blactam			CYP107DN1
Burkholderia sp. Bp5365	bud 2	3	Region 1	RiPP-like			CYP1488A2
			Region 5	NRPS-like,transAT-PKS,NRPS,PKS-like	thailanstatin A	100%	CYP1486A1
			Region 13	hglE-KS,terpene	myxochromide D	20%	CYP1013A3
Paraburkholderia phymatum	bph 2	1	Region 5	terpene			CYP133C2
Paraburkholderia caribensis	bcai 2	1	Region 1	terpene			CYP133C2
Paraburkholderia hospita	phs 2	1	Region 5	terpene			CYP133C2
Paraburkholderia terrae	pter 2	1	Region 5	terpene			CYP133C2
Paraburkholderia megapolitana	pmeg 2	1	Region 4	NRPS-like			CYP1686A1

Mycetohabitans rhizoxinica	brh	1	Region 6	transAT-PKS,NRPS-like	rhizoxin A	88%	CYP1464A3
Achromobacter sp. AONIH1	achr	1	Region 6	NRPS-like			CYP2308A1
Paralcaligenes sp. KSB-10	park	1	Region 5	NRPS			CYP107DG11
Acidovorax sp. JS42	ajs	1	Region 2	NRPS-like			CYP1318A1
Massilia umbonata	mum	1	Region 6	linaridin			CYP1104B1
Methyloversatilis sp. RAC08	metr	1	Region 7	terpene			CYP1246A

Deltaproteobacteria

**Species name**	**Species code**	**Number of P450s part of clusters**	**Region**	**Type**	**Most similar known cluster**	**Similarity**	**P450s**
Archangium violaceum	avm	4	Region 26	T1PKS,NRPS,lanthipeptide-class-ii,lanthipeptide-class-i	ajudazol A	84%	CYP1224J3
			Region 31	NRPS-like	glidobactin	10%	CYP109R10
			Region 39	NRPS,T1PKS,NRPS-like	puwainaphycin A / puwainaphycin B / puwainaphycin C / puwainaphycin D	50%	CYP242A6
			Region 40	terpene			CYP1329A2

Melittangium boletus DSM 14713	mbd	4	Region 1	T1PKS	microsclerodermin M	18%	CYP253D
			Region 3	proteusin	haliamide	29%	CYP262F1
			Region 5	terpene			CYP167B10 , CYP251V1
Cystobacter fuscus	cfus	6	Region 16	NRPS			CYP105EK1
			Region 17	proteusin,RiPP-like			CYP120G3
			Region 18	terpene			CYP251V1
			Region 19	terpene			CYP262B4
			Region 28	NRPS,T1PKS,NRPS-like	minutissamide A / minutissamide C / minutissamide D	30%	CYP1011E3
			Region 40	terpene			CYP183BE1
Sorangium cellulosum So ce56	scl	3	Region 13	T3PKS			CYP262A1
			Region 20	hglE-KS,T1PKS			CYP263A1

			Region 34	terpene	eremophilene	100%	CYP264B1
Chondromyces crocatus	ccro	6	Region 4	T1PKS,NRPS,RiPP-like			CYP110R2
			Region 9	T1PKS			CYP263A3
			Region 13	T1PKS,NRPS-like,NRPS	crocacin	100%	CYP1011F1
			Region 15	NRPS,T1PKS			CYP147L1
			Region 17	NRPS,RiPP-like			CYP107HW1
			Region 20	T1PKS,NRPS-like	ajudazol A	38%	CYP107HU1
Sandaracinus amylolyticus	samy	2	Region 2	terpene			CYP110T1
			Region 15	terpene			CYP51B3
Minicystis rosea	mrm	6	Region 5	hglE-KS,T1PKS,terpene			CYP51B2, CYP263A2
			Region 8	terpene			CYP183AH3

			Region 14	NRPS-like	ralsolamycin	40%	CYP1499A1
			Region 26	lanthipeptide-class-ii,LAP			CYP1448B1
			Region 42	hglE-KS,T1PKS			CYP1069H1
Haliangium ochraceum DSM 14365	hoh	10	Region 1	RRE-containing			CYP107HZ1
			Region 2	RRE-containing			CYP107JA1, CYP262C1, CYP1490A1, CYP1504A1, CYP1489A2
			Region 18	NRPS,T1PKS,phenazine	aurafuron A	71%	CYP1486B2, CYP126B3, CYP110U1
			Region 23	RiPP-like			CYP1069B2
Archangium gephyra	age	6	Region 9	NRPS-like,T1PKS,NRPS	crocacin	38%	CYP107DP4, CYP1011E
			Region 14	terpene,lanthipeptide-class-ii			CYP1329A1
			Region 19	terpene			CYP1298A1, CYP1347B3
			Region 35	T1PKS,NRPS	microsclerodermin	21%	CYP253G3

Stigmatella aurantiaca DW4/3-1	sur	10	Region 1	T1PKS,NRPS	nostopeptolide A2	25%	CYP1494A1
			Region 6	NRPS,T1PKS,NRPS-like	minutissamide A / minutissamide C / minutissamide D	30%	CYP1011E2
			Region 11	T1PKS	dawenol	100%	CYP107DP2
			Region 13	indole			CYP120H1
			Region 18	NRPS,furan,terpene	myxochelin A / myxochelin B	100%	CYP1498A1
			Region 29	T1PKS	aurafuron A	100%	CYP1486B1, CYP126B2, CYP264J1, CYP1495A1
			Region 38	microviridin	microviridin K	25%	CYP264J1
Desulfocurvibacter africanus subsp. *africanus* str. Walvis Bay	daf	1	Region 3	NRPS-like,RiPP-like			CYP152E2
Myxococcus xanthus DK 1622	mxa	1	Region 13	transAT-PKS,NRPS,PKS-like	myxovirescin A1	78%	CYP209A1
Myxococcus stipitatus DSM 14675	msd	7	Region 11	NRPS,T1PKS	pyridomycin	7%	CYP253L1
			Region 16	NRPS,T1PKS	phenalamide A2	100%	CYP1491A1

			Region 19	T1PKS,other	dawenol	77%	CYP1497A1, CYP120F1, CYP1503A1, CYP1495A1
			Region 28	T3PKS	alkylpyrone-407 / alkylpyrone-393	25%	CYP229H1
Myxococcus hansupus	mym	1	Region 3	terpene	geosmin	100 %	CYP109S1
Corallococcus coralloides DSM 2259	ccx	2	Region 16	NRPS			CYP264D1
			Region 21	NRPS			CYP264D6

Epsilonproteobacteria

Species names	Species code	No of p450s	Cluster Count	Region	Type	From	To	Most similar known cluster	Similarity	P450 in Clusters

Helicobacter winghamensis	hwi	1								
Campylobacter jejuni subsp. *jejuni* NCTC 11168 = ATCC 700819	cje	1								
Campylobacter jejuni subsp. *jejuni* NCTC 11168-BN148	cjb	1								
Campylobacter jejuni subsp. *jejuni* 81-176	cjj	1								
Campylobacter jejuni subsp. *jejuni* 81116	cju	1								

Campylobacter jejuni subsp. *jejuni* ICDCCJ07001	cjn	1								
Campylobacter jejuni subsp. *jejuni* IA3902	cji	1								
Campylobacter jejuni subsp. *jejuni* M1	cjm	1								
Campylobacter jejuni subsp. *jejuni* S3	cjs	1								
Campylobacter jejuni subsp. *jejuni* PT14	cjp	1	1	Region 1	betalactone	858,63	888,467			

Campylobacter jejuni subsp. *jejuni* 00-2426	cjej	1	1	Region 1	betalactone	859,152	888,989			
Campylobacter jejuni subsp. *jejuni* 00-2538	cjeu	1	1	Region 1	betalactone	897,707	927,544			
Campylobacter jejuni subsp. *jejuni* 00-2544	cjen	1	1	Region 1	betalactone	897,708	927,545			
Campylobacter jejuni subsp. *jejuni* 00-2425	cjei	1	1	Region 1	betalactone	897,706	927,543			
Campylobacter jejuni subsp. *jejuni* R14	cjer	1	1	Region 1	betalactone	963,554	993,393			

Campylobacter jejuni subsp. *jejuni* MTVDSCj20	cjv									
Campylobacter jejuni subsp. *jejuni* 00-1597	cjl	1	1	Region 1	betalactone	856,759	886,598			
Campylobacter jejuni subsp. *jejuni* 00-6200	cjw	1	1	Region 1	betalactone	857,862	887,699			
Campylobacter jejuni RM1221	cjr	1								
Campylobacter jejuni subsp. *doylei* 269.97	cjd	1	1	Region 1	betalactone	935,951	965,789			
Campylobacter jejuni 32488	cjz	1	1	Region 1	betalactone	10,63	40,467			

Campylobacter jejuni 4031	cjx	1	2	Region 1	thioamide-NRP	181,841	244,976			
				Region 2	arylpolyene	1,433,255	1,476,717	APE Vf	20%	
Campylobacter lari RM2100	cla	1								
Campylobacter lari RM16701	clr	1								
Campylobacter lari RM16712	clm	1								
Campylobacter lari NCTC 11845	cln	1								
Campylobacter lari subsp. *concheus* LMG 11760	cll	1								
Campylobacter coli 76339	ccol	1								
Campylobacter coli CVM N29710	ccc	1								

Campylobacter coli 15-537360	ccq	1								
Campylobacter coli RM1875	ccf	1								
Campylobacter coli RM4661	ccy	1								
Campylobacter coli RM5611	ccoi	1								
Campylobacter coli FB1	ccof	1								
Campylobacter coli OR12	ccoo	1								
Campylobacter insulaenigrae	cis	1								
Campylobacter volucris	cvo	1								
Campylobacter peloridis	cpel	1								
Campylobacter sp. RM16704	camr	1								

Campylobacter subantarcticus LMG 24374	csm	1								
Campylobacter subantarcticus LMG 24377	csf	1								
Campylobacter helveticus	chv	1								
Campylobacter cuniculorum	ccun	1								
Campylobacter lanienae	clx	1								
Campylobacter avium	cavi	1								
Campylobacter hepaticus	chw	1								
Campylobacter sp. RM12175	camz	1								
Campylobacter sp. RM6137	camy	1								

Campylobacter ornithocola	coj	1								
Campylobacter upsaliensis	cux	1								
Campylobacter armoricus	carm	1								
Campylobacter novaezeelandiae	cnv	1								
Malaciobacter mytili	amyt	1								

1. Nzuza, N.; Padayachee, T.; Syed, P. R.; Kryś, J. D.; Chen, W.; Gront, D.; Nelson, D. R.; Syed, K., Ancient Bacterial Class Alphaproteobacteria Cytochrome P450 Monooxygenases Can Be Found in Other Bacterial Species. *International journal of molecular sciences* **2021,** 22, (11), 5542.
2. Msomi, N. N.; Padayachee, T.; Nzuza, N.; Syed, P. R.; Kryś, J. D.; Chen, W.; Gront, D.; Nelson, D. R.; Syed, K., In silico analysis of P450s and their role in secondary metabolism in the bacterial class Gammaproteobacteria. *Molecules (Basel, Switzerland)* **2021,** 26, (6), 1538.
3. Blin, K.; Shaw, S.; Steinke, K.; Villebro, R.; Ziemert, N.; Lee, S. Y.; Medema, M. H.; Weber, T., antiSMASH 5.0: updates to the secondary metabolite genome mining pipeline. *Nucleic acids research* **2019**.
